# Supplementary material for: ERK2-topoisomerase II regulatory axis is important for gene activation in immediate early genes
Source: Nat Commun. 2023 Dec 14;14:8341. doi: 10.1038/s41467-023-44089-y (PMC10721843; doi:10.1038/s41467-023-44089-y)
Supplement: Supplementary file 3 — Description of Additional Supplementary Files [file 41467_2023_44089_MOESM3_ESM.pdf]

Description of Additional Supplementary Files

File Name: Supplementary Data 1
Description: Mass spectrometry data- deTOP2B control

File Name: Supplementary Data 2
Description: Mass spectrometry data- deTOP2B with ERK1

File Name: Supplementary Data 3
Description: Mass spectrometry data- deTOP2B with ERK2

File Name: Supplementary Data 4
Description: Mass spectrometry data- deTOP2B with ERK1m

File Name: Supplementary Data 5
Description: Mass spectrometry data- deTOP2B with ERK2m

File Name: Supplementary Data 6
Description: Mass spectrometry data- spectrum

File Name: Supplementary Data 7
Description: Mass spectrometry data-CTRL reaction against *E. coli* proteome

File Name: Supplementary Data 8
Description: Mass spectrometry data-ERK1m reaction against *E. coli* proteome

File Name: Supplementary Data 9
Description: Mass spectrometry data-ERK2m reaction against *E. coli* proteome
