# Supplementary material for: ERK2-topoisomerase II regulatory axis is important for gene activation in immediate early genes
Source: Nat Commun. 2023 Dec 14;14:8341. doi: 10.1038/s41467-023-44089-y (PMC10721843; doi:10.1038/s41467-023-44089-y)

MASCOT Search Results

Protein View: Q02880

DNA topoisomerase 2-beta OS=Homo sapiens OX=9606 GN=TOP2B PE=1 SV=3

**Database:**	Uniprot_Proteome_Human
**Score:**	18972
**Monoisotopic mass (M_r):**	184122
**Calculated pI:**	8.14

Sequence similarity is available as an NCBI BLAST search of Q02880 against nr.

Search parameters

**MS data file:**	File Name: Z:\SN22\SN221362_deTOP2B_control_1ul.raw;File Path: ;File Time: 3/1/2023 7:06:29 AM;File Size: 352609374 [Byte]
**Enzyme:**	Trypsin: cuts C-term side of KR unless next residue is P.
**Fixed modifications:**	Carbamidomethyl (C)
**Variable modifications:**	Acetyl (Protein N-term), Oxidation (M), Phospho (ST), Phospho (Y)

Protein sequence coverage: 65%

Matched peptides shown in ***bold red***.

```
   1 MAKSGGCGAG AGVGGGNGAL TWVTLFDQNN AAKKEESETA NKNDSSKKLS
  51 VERVYQKKTQ LEHILLRPDT YIGSVEPLTQ FMWVYDEDVG MNCREVTFVP
 101 GLYKIFDEIL VNAADNKQRD KNMTCIKVSI DPESNIISIW NNGKGIPVVE
 151 HKVEKVYVPA LIFGQLLTSS NYDDDEKKVT GGRNGYGAKL CNIFSTKFTV
 201 ETACKEYKHS FKQTWMNNMM KTSEAKIKHF DGEDYTCITF QPDLSKFKME
 251 KLDKDIVALM TRRAYDLAGS CRGVKVMFNG KKLPVNGFRS YVDLYVKDKL
 301 DETGVALKVI HELANERWDV CLTLSEKGFQ QISFVNSIAT TKGGRHVDYV
 351 VDQVVGKLIE VVKKKNKAGV SVKPFQVKNH IWVFINCLIE NPTFDSQTKE
 401 NMTLQPKSFG SKCQLSEKFF KAASNCGIVE SILNWVKFKA QTQLNKKCSS
 451 VKYSKIKGIP KLDDANDAGG KHSLECTLIL TEGDSAKSLA VSGLGVIGRD
 501 RYGVFPLRGK ILNVREASHK QIMENAEINN IIKIVGLQYK KSYDDAESLK
 551 TLRYGKIMIM TDQDQDGSHI KGLLINFIHH NWPSLLKHGF LEEFITPIVK
 601 ASKNKQELSF YSIPEFDEWK KHIENQKAWK IKYYKGLGTS TAKEAKEYFA
 651 DMERHRILFR YAGPEDDAAI TLAFSKKKID DRKEWLTNFM EDRRQRRLHG
 701 LPEQFLYGTA TKHLTYNDFI NKELILFSNS DNERSIPSLV DGFKPGQRKV
 751 LFTCFKRNDK REVKVAQLAG SVAEMSAYHH GEQALMMTIV NLAQNFVGSN
 801 NINLLQPIGQ FGTRLHGGKD AASPRYIFTM LSTLARLLFP AVDDNLLKFL
 851 YDDNQRVEPE WYIPIIPMVL INGAEGIGTG WACKLPNYDA REIVNNVRRM
 901 LDGLDPHPML PNYKNFKGTI QELGQNQYAV SGEIFVVDRN TVEITELPVR
 951 TWTQVYKEQV LEPMLNGTDK TPALISDYKE YHTDTTVKFV VKMTEEKLAQ
1001 AEAAGLHKVF KLQTTLTCNS MVLFDHMGCL KKYETVQDIL KEFFDLRLSY
1051 YGLRKEWLVG MLGAESTKLN NQARFILEKI QGKITIENRS KKDLIQMLVQ
1101 RGYESDPVKA WKEAQEKAAE EDETQNQHDD SSSDSGTPSG PDFNYILNMS
1151 LWSLTKEKVE ELIKQRDAKG REVNDLKRKS PSDLWKEDLA AFVEELDKVE
1201 SQEREDVLAG MSGKAIKGKV GKPKVKKLQL EETMPSPYGR RIIPEITAMK
1251 ADASKKLLKK KKGDLDTAAV KVEFDEEFSG APVEGAGEEA LTPSVPINKG
1301 PKPKREKKEP GTRVRKTPTS SGKPSAKKVK KRNPWSDDES KSESDLEETE
1351 PVVIPRDSLL RRAAAERPKY TFDFSEEEDD DADDDDDDNN DLEELKVKAS
1401 PITNDGEDEF VPSDGLDKDE YTFSPGKSKA TPEKSLHDKK SQDFGNLFSF
1451 PSYSQKSEDD SAKFDSNEED SASVFSPSFG LKQTDKVPSK TVAAKKGKPS
1501 SDTVPKPKRA PKQKKVVEAV NSDSDSEFGI PKKTTTPKGK GRGAKKRKAS
1551 GSENEGDYNP GRKTSKTTSK KPKKTSFDQD SDVDIFPSDF PTEPPSLPRT
1601 GRARKEVKYF AESDEEEDDV DFAMFN
```

Unformatted sequence string: 1626 residues (for pasting into other applications).

Sort by (•) residue number ( ) increasing mass ( ) decreasing mass
Show (•) matched peptides only ( ) predicted peptides also

Query	Start – End	Observed	Mr(expt)	Mr(calc)	ppm	M	Score	Expect	Rank	U	Peptide
1779	34 – 42	518.2515	1034.4885	1034.4880	0.48	1	42	0.0034	1	U	K.KEESETANK.N
6439	34 – 47	783.8663	1565.7180	1565.7169	0.72	2	32	0.003	1	U	K.KEESETANKNDSSK.K
7502	34 – 48	847.9134	1693.8123	1693.8118	0.30	3	47	0.00022	1	U	K.KEESETANKNDSSKK.L
7503	34 – 48	565.6116	1693.8128	1693.8118	0.60	3	25	0.026	1	U	K.KEESETANKNDSSKK.L
7504	34 – 48	847.9140	1693.8135	1693.8118	0.99	3	30	0.0016	1	U	K.KEESETANKNDSSKK.L
5258	105 – 117	487.9234	1460.7485	1460.7511	-1.77	0	35	0.0043	1		K.IFDEILVNAADNK.Q
5259	105 – 117	731.3818	1460.7490	1460.7511	-1.41	0	109	9.6e-10	1		K.IFDEILVNAADNK.Q
5260	105 – 117	487.9239	1460.7500	1460.7511	-0.75	0	31	0.013	1		K.IFDEILVNAADNK.Q
5263	105 – 117	487.9241	1460.7505	1460.7511	-0.42	0	27	0.017	1		K.IFDEILVNAADNK.Q
5264	105 – 117	731.3825	1460.7505	1460.7511	-0.39	0	67	2.7e-06	1		K.IFDEILVNAADNK.Q
5265	105 – 117	487.9241	1460.7505	1460.7511	-0.38	0	22	0.018	1		K.IFDEILVNAADNK.Q
5266	105 – 117	487.9241	1460.7506	1460.7511	-0.36	0	25	0.024	1		K.IFDEILVNAADNK.Q
5267	105 – 117	487.9241	1460.7506	1460.7511	-0.34	0	27	0.021	1		K.IFDEILVNAADNK.Q
5268	105 – 117	487.9242	1460.7507	1460.7511	-0.30	0	22	0.043	1		K.IFDEILVNAADNK.Q
5269	105 – 117	487.9242	1460.7507	1460.7511	-0.25	0	25	0.013	1		K.IFDEILVNAADNK.Q
5271	105 – 117	731.3827	1460.7508	1460.7511	-0.17	0	71	1.3e-06	1		K.IFDEILVNAADNK.Q
5272	105 – 117	731.3827	1460.7508	1460.7511	-0.16	0	51	7.6e-05	1		K.IFDEILVNAADNK.Q
5273	105 – 117	487.9242	1460.7509	1460.7511	-0.13	0	43	0.0012	1		K.IFDEILVNAADNK.Q
5274	105 – 117	487.9242	1460.7509	1460.7511	-0.11	0	35	0.0045	1		K.IFDEILVNAADNK.Q

Query	Start - End	Observed	Mr(expt)	Mr(calc)	ppm	M	Score	Expect	Rank	U	Peptide
5275	105 - 117	731.3828	1460.7510	1460.7511	-0.051	0	73	8.7e-07	1		K.IFDEILVNAADNK.Q
5276	105 - 117	487.9243	1460.7510	1460.7511	-0.049	0	40	0.0029	1		K.IFDEILVNAADNK.Q
5277	105 - 117	487.9243	1460.7510	1460.7511	-0.049	0	27	0.026	1		K.IFDEILVNAADNK.Q
5278	105 - 117	487.9243	1460.7511	1460.7511	-0.0075	0	38	0.012	1		K.IFDEILVNAADNK.Q
5279	105 - 117	487.9243	1460.7511	1460.7511	-0.0075	0	32	0.0044	1		K.IFDEILVNAADNK.Q
5280	105 - 117	731.3829	1460.7512	1460.7511	0.086	0	66	6.8e-06	1		K.IFDEILVNAADNK.Q
5281	105 - 117	487.9244	1460.7512	1460.7511	0.095	0	43	0.00063	1		K.IFDEILVNAADNK.Q
5282	105 - 117	487.9244	1460.7513	1460.7511	0.14	0	49	0.0007	1		K.IFDEILVNAADNK.Q
5283	105 - 117	731.3829	1460.7513	1460.7511	0.15	0	83	4.7e-07	1		K.IFDEILVNAADNK.Q
5284	105 - 117	731.3830	1460.7513	1460.7511	0.18	0	69	2.4e-06	1		K.IFDEILVNAADNK.Q
5285	105 - 117	731.3830	1460.7514	1460.7511	0.21	0	75	4.1e-07	1		K.IFDEILVNAADNK.Q
5286	105 - 117	731.3830	1460.7515	1460.7511	0.26	0	65	7.8e-06	1		K.IFDEILVNAADNK.Q
5287	105 - 117	487.9245	1460.7516	1460.7511	0.34	0	21	0.037	1		K.IFDEILVNAADNK.Q
5289	105 - 117	487.9246	1460.7518	1460.7511	0.51	0	33	0.011	1		K.IFDEILVNAADNK.Q
5290	105 - 117	731.3832	1460.7518	1460.7511	0.52	0	66	4e-06	1		K.IFDEILVNAADNK.Q
5291	105 - 117	731.3832	1460.7518	1460.7511	0.52	0	63	1e-05	1		K.IFDEILVNAADNK.Q
5293	105 - 117	487.9246	1460.7519	1460.7511	0.59	0	37	0.0036	1		K.IFDEILVNAADNK.Q
5294	105 - 117	731.3833	1460.7520	1460.7511	0.61	0	76	1.7e-06	1		K.IFDEILVNAADNK.Q
5295	105 - 117	487.9247	1460.7522	1460.7511	0.79	0	31	0.016	1		K.IFDEILVNAADNK.Q
5296	105 - 117	731.3838	1460.7531	1460.7511	1.37	0	78	5.9e-07	1		K.IFDEILVNAADNK.Q
7934	105 - 119	582.6445	1744.9117	1744.9108	0.52	1	30	0.014	1		K.IFDEILVNAADNKQR.D
1697	120 - 127	513.2411	1024.4677	1024.4681	-0.40	1	28	0.04	1	U	R.DKNMTCIK.V + Oxidation (M)
9003	128 - 144	943.4857	1884.9568	1884.9581	-0.71	0	62	3.6e-06	1	U	K.VSIDPESNIISIWNNGK.G
9004	128 - 144	943.4863	1884.9581	1884.9581	-0.0037	0	69	1.2e-06	1	U	K.VSIDPESNIISIWNNGK.G
9005	128 - 144	629.3268	1884.9586	1884.9581	0.24	0	59	2.2e-05	1	U	K.VSIDPESNIISIWNNGK.G
9006	128 - 144	943.4870	1884.9594	1884.9581	0.68	0	59	9.6e-06	1	U	K.VSIDPESNIISIWNNGK.G
779	145 - 152	439.7585	877.5024	877.5022	0.27	0	28	0.043	1		K.GIPVVEHK.V
12009	153 - 177	948.4820	2842.4241	2842.4276	-1.24	1	54	1.7e-05	1	U	K.VEKVYVPALIFGQLLTSSNYDDDEK.K
11199	156 - 177	1244.1171	2486.2197	2486.2217	-0.79	0	53	3.4e-05	1	U	K.VYVPALIFGQLLTSSNYDDDEK.K
11200	156 - 177	829.7473	2486.2200	2486.2217	-0.67	0	31	0.011	1	U	K.VYVPALIFGQLLTSSNYDDDEK.K
11201	156 - 177	1244.1174	2486.2202	2486.2217	-0.60	0	24	0.012	1	U	K.VYVPALIFGQLLTSSNYDDDEK.K
11202	156 - 177	829.7476	2486.2211	2486.2217	-0.22	0	25	0.036	1	U	K.VYVPALIFGQLLTSSNYDDDEK.K
11203	156 - 177	829.7477	2486.2212	2486.2217	-0.21	0	53	7e-05	1	U	K.VYVPALIFGQLLTSSNYDDDEK.K
11204	156 - 177	1244.1187	2486.2228	2486.2217	0.45	0	95	9.2e-09	1	U	K.VYVPALIFGQLLTSSNYDDDEK.K
11205	156 - 177	829.7482	2486.2229	2486.2217	0.49	0	36	0.0021	1	U	K.VYVPALIFGQLLTSSNYDDDEK.K
11206	156 - 177	829.7483	2486.2231	2486.2217	0.56	0	35	0.0073	1	U	K.VYVPALIFGQLLTSSNYDDDEK.K
11207	156 - 177	1244.1190	2486.2233	2486.2217	0.67	0	46	0.00036	1	U	K.VYVPALIFGQLLTSSNYDDDEK.K
11209	156 - 177	1244.1200	2486.2255	2486.2217	1.56	0	29	0.0033	1	U	K.VYVPALIFGQLLTSSNYDDDEK.K
11509	156 - 178	872.4442	2614.3107	2614.3166	-2.27	1	39	0.011	1	U	K.VYVPALIFGQLLTSSNYDDDEKK.V
11513	156 - 178	872.4460	2614.3162	2614.3166	-0.17	1	36	0.028	1	U	K.VYVPALIFGQLLTSSNYDDDEKK.V
11514	156 - 178	1308.1656	2614.3166	2614.3166	0.0050	1	58	7.4e-05	1	U	K.VYVPALIFGQLLTSSNYDDDEKK.V
11516	156 - 178	654.5866	2614.3172	2614.3166	0.22	1	39	0.013	1	U	K.VYVPALIFGQLLTSSNYDDDEKK.V
11517	156 - 178	872.4464	2614.3173	2614.3166	0.24	1	32	0.028	1	U	K.VYVPALIFGQLLTSSNYDDDEKK.V
11519	156 - 178	872.4464	2614.3173	2614.3166	0.27	1	40	0.011	1	U	K.VYVPALIFGQLLTSSNYDDDEKK.V
11521	156 - 178	872.4466	2614.3178	2614.3166	0.45	1	38	0.0098	1	U	K.VYVPALIFGQLLTSSNYDDDEKK.V
11522	156 - 178	1308.1664	2614.3183	2614.3166	0.65	1	61	9.5e-05	1	U	K.VYVPALIFGQLLTSSNYDDDEKK.V
11524	156 - 178	872.4468	2614.3185	2614.3166	0.72	1	61	8.7e-05	1	U	K.VYVPALIFGQLLTSSNYDDDEKK.V
11526	156 - 178	654.5870	2614.3188	2614.3166	0.83	1	26	0.034	1	U	K.VYVPALIFGQLLTSSNYDDDEKK.V
1394	190 - 197	491.7550	981.4954	981.4953	0.084	0	42	0.0018	1		K.LCNIFSTK.F
1200	198 - 205	478.2307	954.4468	954.4481	-1.35	0	19	0.046	1	U	K.FTVETACK.E
3212	213 - 221	600.2544	1198.4942	1198.4933	0.74	0	32	0.026	1	U	K.QTWMNNMMK.T + Oxidation (M)
3331	213 - 221	608.2515	1214.4885	1214.4882	0.20	0	33	0.011	1	U	K.QTWMNNMMK.T + 2 Oxidation (M)
10569	229 - 246	1126.9722	2251.9298	2251.9133	7.31	0	46	9.8e-05	1	U	K.HFDGEDYTCITFQPDLSK.F + Phospho (ST)
3850	252 - 262	637.8602	1273.7058	1273.7064	-0.47	1	35	0.0036	1	U	K.LDKDIVALMTR.R
3854	252 - 262	637.8603	1273.7060	1273.7064	-0.33	1	25	0.012	1	U	K.LDKDIVALMTR.R
3855	252 - 262	637.8603	1273.7060	1273.7064	-0.27	1	71	4.1e-07	1	U	K.LDKDIVALMTR.R
3856	252 - 262	637.8603	1273.7061	1273.7064	-0.25	1	38	0.00096	1	U	K.LDKDIVALMTR.R
3857	252 - 262	637.8603	1273.7061	1273.7064	-0.21	1	37	0.0057	1	U	K.LDKDIVALMTR.R
3860	252 - 262	637.8606	1273.7066	1273.7064	0.16	1	57	8.5e-05	1	U	K.LDKDIVALMTR.R
3862	252 - 262	637.8606	1273.7067	1273.7064	0.25	1	64	1e-05	1	U	K.LDKDIVALMTR.R
3864	252 - 262	425.5762	1273.7069	1273.7064	0.39	1	39	0.0055	1	U	K.LDKDIVALMTR.R
3865	252 - 262	637.8608	1273.7070	1273.7064	0.45	1	28	0.011	1	U	K.LDKDIVALMTR.R
3866	252 - 262	637.8608	1273.7070	1273.7064	0.45	1	25	0.0048	1	U	K.LDKDIVALMTR.R
3867	252 - 262	637.8608	1273.7071	1273.7064	0.53	1	38	0.00066	1	U	K.LDKDIVALMTR.R
3868	252 - 262	637.8609	1273.7073	1273.7064	0.74	1	34	0.005	1	U	K.LDKDIVALMTR.R
3981	252 - 262	645.8572	1289.6999	1289.7013	-1.06	1	49	0.00012	1	U	K.LDKDIVALMTR.R + Oxidation (M)
3982	252 - 262	645.8574	1289.7003	1289.7013	-0.81	1	40	0.0015	1	U	K.LDKDIVALMTR.R + Oxidation (M)
3984	252 - 262	645.8576	1289.7006	1289.7013	-0.53	1	43	0.001	1	U	K.LDKDIVALMTR.R + Oxidation (M)
3985	252 - 262	430.9075	1289.7006	1289.7013	-0.52	1	29	0.015	1	U	K.LDKDIVALMTR.R + Oxidation (M)
3986	252 - 262	430.9075	1289.7007	1289.7013	-0.47	1	45	0.00056	1	U	K.LDKDIVALMTR.R + Oxidation (M)
3988	252 - 262	430.9076	1289.7010	1289.7013	-0.24	1	41	0.0037	1	U	K.LDKDIVALMTR.R + Oxidation (M)
3989	252 - 262	430.9076	1289.7010	1289.7013	-0.24	1	37	0.0019	1	U	K.LDKDIVALMTR.R + Oxidation (M)
3990	252 - 262	645.8578	1289.7011	1289.7013	-0.17	1	43	0.0006	1	U	K.LDKDIVALMTR.R + Oxidation (M)
3991	252 - 262	430.9076	1289.7011	1289.7013	-0.14	1	39	0.0038	1	U	K.LDKDIVALMTR.R + Oxidation (M)
3992	252 - 262	645.8578	1289.7011	1289.7013	-0.14	1	65	1e-05	1	U	K.LDKDIVALMTR.R + Oxidation (M)
3993	252 - 262	430.9077	1289.7012	1289.7013	-0.098	1	29	0.022	1	U	K.LDKDIVALMTR.R + Oxidation (M)
3994	252 - 262	430.9077	1289.7012	1289.7013	-0.075	1	30	0.012	1	U	K.LDKDIVALMTR.R + Oxidation (M)
3995	252 - 262	645.8579	1289.7013	1289.7013	-0.032	1	58	8.9e-06	1	U	K.LDKDIVALMTR.R

Query	Start – End	Observed	Mr(expt)	Mr(calc)	ppm	M	Score	Expect	Rank	U	Peptide
											+ Oxidation (M)
3996	252 – 262	645.8580	1289.7014	1289.7013	0.077	1	57	2.5e-05	1	U	K.LDKDIVALMTR.R<br>+ Oxidation (M)
988	255 – 262	459.7574	917.5003	917.5004	-0.086	0	42	0.0016	1	U	K.DIVALMTR.R
1089	255 – 262	467.7547	933.4948	933.4953	-0.55	0	31	0.043	1	U	K.DIVALMTR.R<br>+ Oxidation (M)
1090	255 – 262	467.7548	933.4950	933.4953	-0.40	0	31	0.042	1	U	K.DIVALMTR.R<br>+ Oxidation (M)
1093	255 – 262	467.7552	933.4958	933.4953	0.49	0	32	0.034	1	U	K.DIVALMTR.R<br>+ Oxidation (M)
2994	263 – 272	584.7797	1167.5449	1167.5455	-0.48	1	41	0.0007	1	U	R.RAYDLAGSCR.G
1596	264 – 272	506.7296	1011.4446	1011.4444	0.24	0	59	1.4e-05	1	U	R.AYDLAGSCR.G
1419	290 – 297	493.7634	985.5122	985.5120	0.13	0	29	0.01	1	U	R.SYVDLYVK.D
1420	290 – 297	493.7634	985.5123	985.5120	0.25	0	27	0.018	1	U	R.SYVDLYVK.D
1421	290 – 297	493.7637	985.5128	985.5120	0.80	0	43	0.0006	1	U	R.SYVDLYVK.D
3440	290 – 299	615.3242	1228.6338	1228.6339	-0.13	1	20	0.041	1	U	R.SYVDLYVKDK.L
3143	298 – 308	594.8268	1187.6391	1187.6398	-0.56	1	50	0.00032	1	U	K.DKLDETGVALK.V
3144	298 – 308	594.8268	1187.6391	1187.6398	-0.51	1	44	0.003	1	U	K.DKLDETGVALK.V
3146	298 – 308	594.8270	1187.6395	1187.6398	-0.17	1	27	0.025	1	U	K.DKLDETGVALK.V
10560	298 – 317	563.3082	2249.2038	2249.2015	1.00	2	24	0.026	1	U	K.DKLDETGVALKVIHELANER.W
1154	300 – 308	473.2664	944.5183	944.5179	0.46	0	51	0.00026	1	U	K.LDETGVALK.V
2158	309 – 317	540.7923	1079.5700	1079.5723	-2.19	0	39	0.0027	1	U	K.VIHELANER.W
2159	309 – 317	360.8641	1079.5704	1079.5723	-1.75	0	25	0.028	1	U	K.VIHELANER.W
2162	309 – 317	360.8646	1079.5721	1079.5723	-0.25	0	29	0.0075	1	U	K.VIHELANER.W
2163	309 – 317	540.7933	1079.5721	1079.5723	-0.24	0	26	0.018	1	U	K.VIHELANER.W
2164	309 – 317	540.7933	1079.5721	1079.5723	-0.22	0	30	0.027	1	U	K.VIHELANER.W
2165	309 – 317	360.8647	1079.5722	1079.5723	-0.16	0	20	0.032	1	U	K.VIHELANER.W
2167	309 – 317	360.8647	1079.5723	1079.5723	0.0046	0	29	0.013	1	U	K.VIHELANER.W
2168	309 – 317	540.7934	1079.5723	1079.5723	0.019	0	25	0.023	1	U	K.VIHELANER.W
2169	309 – 317	540.7935	1079.5724	1079.5723	0.094	0	34	0.0023	1	U	K.VIHELANER.W
2172	309 – 317	360.8648	1079.5725	1079.5723	0.20	0	30	0.014	1	U	K.VIHELANER.W
2173	309 – 317	540.7936	1079.5726	1079.5723	0.22	0	51	0.00035	1	U	K.VIHELANER.W
2174	309 – 317	360.8648	1079.5726	1079.5723	0.23	0	44	0.0018	1	U	K.VIHELANER.W
2175	309 – 317	540.7937	1079.5728	1079.5723	0.41	0	28	0.035	1	U	K.VIHELANER.W
2176	309 – 317	360.8649	1079.5729	1079.5723	0.56	0	34	0.018	1	U	K.VIHELANER.W
3624	318 – 327	625.8079	1249.6012	1249.6013	-0.052	0	57	1.8e-05	1	U	R.WDVCLTLSEK.G
7018	328 – 342	820.9341	1639.8536	1639.8570	-2.03	0	60	3.5e-05	1	U	K.GFQQISFVNSIATTK.G
7019	328 – 342	820.9342	1639.8539	1639.8570	-1.87	0	38	0.00083	1	U	K.GFQQISFVNSIATTK.G
7020	328 – 342	820.9343	1639.8540	1639.8570	-1.81	0	38	0.00048	1	U	K.GFQQISFVNSIATTK.G
7021	328 – 342	820.9345	1639.8544	1639.8570	-1.57	0	38	0.00027	1	U	K.GFQQISFVNSIATTK.G
7022	328 – 342	820.9347	1639.8548	1639.8570	-1.35	0	43	0.00019	1	U	K.GFQQISFVNSIATTK.G
7023	328 – 342	820.9347	1639.8548	1639.8570	-1.34	0	51	2.1e-05	1	U	K.GFQQISFVNSIATTK.G
7024	328 – 342	820.9349	1639.8553	1639.8570	-1.04	0	61	6.6e-06	1	U	K.GFQQISFVNSIATTK.G
7026	328 – 342	820.9353	1639.8560	1639.8570	-0.59	0	53	9.7e-05	1	U	K.GFQQISFVNSIATTK.G
7027	328 – 342	820.9353	1639.8561	1639.8570	-0.53	0	43	0.0012	1	U	K.GFQQISFVNSIATTK.G
7028	328 – 342	820.9353	1639.8561	1639.8570	-0.52	0	67	1.1e-05	1	U	K.GFQQISFVNSIATTK.G
7029	328 – 342	820.9354	1639.8563	1639.8570	-0.41	0	28	0.0032	1	U	K.GFQQISFVNSIATTK.G
7030	328 – 342	820.9355	1639.8565	1639.8570	-0.30	0	39	0.01	1	U	K.GFQQISFVNSIATTK.G
7031	328 – 342	547.6261	1639.8565	1639.8570	-0.29	0	48	0.00021	1	U	K.GFQQISFVNSIATTK.G
7032	328 – 342	820.9356	1639.8565	1639.8570	-0.26	0	49	0.00067	1	U	K.GFQQISFVNSIATTK.G
7033	328 – 342	820.9356	1639.8566	1639.8570	-0.23	0	33	0.0028	1	U	K.GFQQISFVNSIATTK.G
7035	328 – 342	820.9356	1639.8567	1639.8570	-0.14	0	24	0.0057	1	U	K.GFQQISFVNSIATTK.G
7036	328 – 342	820.9357	1639.8568	1639.8570	-0.10	0	58	2.1e-05	1	U	K.GFQQISFVNSIATTK.G
7037	328 – 342	820.9357	1639.8568	1639.8570	-0.080	0	58	1.2e-05	1	U	K.GFQQISFVNSIATTK.G
7038	328 – 342	820.9357	1639.8569	1639.8570	-0.043	0	27	0.0039	1	U	K.GFQQISFVNSIATTK.G
7039	328 – 342	820.9357	1639.8569	1639.8570	-0.031	0	42	0.0015	1	U	K.GFQQISFVNSIATTK.G
7040	328 – 342	547.6263	1639.8570	1639.8570	-0.016	0	26	0.026	1	U	K.GFQQISFVNSIATTK.G
7041	328 – 342	820.9358	1639.8570	1639.8570	-0.0067	0	61	1.4e-05	1	U	K.GFQQISFVNSIATTK.G
7042	328 – 342	547.6263	1639.8570	1639.8570	0.0018	0	23	0.024	1	U	K.GFQQISFVNSIATTK.G
7043	328 – 342	547.6263	1639.8570	1639.8570	0.020	0	44	0.0002	1	U	K.GFQQISFVNSIATTK.G
7044	328 – 342	820.9358	1639.8571	1639.8570	0.066	0	45	0.0023	1	U	K.GFQQISFVNSIATTK.G
7045	328 – 342	820.9358	1639.8571	1639.8570	0.079	0	22	0.048	1	U	K.GFQQISFVNSIATTK.G
7046	328 – 342	820.9360	1639.8575	1639.8570	0.30	0	39	0.0069	1	U	K.GFQQISFVNSIATTK.G
7047	328 – 342	820.9361	1639.8576	1639.8570	0.40	0	78	1.1e-06	1	U	K.GFQQISFVNSIATTK.G
7048	328 – 342	820.9361	1639.8577	1639.8570	0.43	0	50	0.00029	1	U	K.GFQQISFVNSIATTK.G
7049	328 – 342	820.9362	1639.8578	1639.8570	0.52	0	52	4.2e-05	1	U	K.GFQQISFVNSIATTK.G
7050	328 – 342	547.6266	1639.8579	1639.8570	0.53	0	39	0.00028	1	U	K.GFQQISFVNSIATTK.G
7051	328 – 342	820.9363	1639.8581	1639.8570	0.66	0	47	0.0002	1	U	K.GFQQISFVNSIATTK.G
7054	328 – 342	547.6267	1639.8583	1639.8570	0.79	0	31	0.003	1	U	K.GFQQISFVNSIATTK.G
7056	328 – 342	820.9366	1639.8587	1639.8570	1.07	0	31	0.0071	1	U	K.GFQQISFVNSIATTK.G
7057	328 – 342	820.9369	1639.8592	1639.8570	1.33	0	17	0.025	1	U	K.GFQQISFVNSIATTK.G
4486	346 – 357	679.3587	1356.7028	1356.7038	-0.74	0	52	5.1e-05	1	U	R.HVDYVVDQVVGK.L
4487	346 – 357	679.3587	1356.7029	1356.7038	-0.67	0	79	8.5e-08	1	U	R.HVDYVVDQVVGK.L
4488	346 – 357	679.3588	1356.7030	1356.7038	-0.55	0	44	6.9e-05	1	U	R.HVDYVVDQVVGK.L
4489	346 – 357	679.3588	1356.7031	1356.7038	-0.51	0	32	0.0023	1	U	R.HVDYVVDQVVGK.L
4491	346 – 357	679.3590	1356.7034	1356.7038	-0.30	0	80	1.2e-07	1	U	R.HVDYVVDQVVGK.L
4492	346 – 357	679.3591	1356.7036	1356.7038	-0.14	0	52	4.8e-05	1	U	R.HVDYVVDQVVGK.L
4493	346 – 357	679.3591	1356.7037	1356.7038	-0.080	0	79	8.7e-08	1	U	R.HVDYVVDQVVGK.L
4494	346 – 357	679.3591	1356.7037	1356.7038	-0.065	0	49	7.2e-05	1	U	R.HVDYVVDQVVGK.L
4495	346 – 357	679.3592	1356.7038	1356.7038	0.0088	0	76	2.4e-07	1	U	R.HVDYVVDQVVGK.L
4496	346 – 357	679.3592	1356.7038	1356.7038	0.0088	0	60	3.6e-06	1	U	R.HVDYVVDQVVGK.L
4498	346 – 357	679.3593	1356.7041	1356.7038	0.24	0	47	8.1e-05	1	U	R.HVDYVVDQVVGK.L
4500	346 – 357	453.2421	1356.7044	1356.7038	0.45	0	34	0.024	1	U	R.HVDYVVDQVVGK.L
4502	346 – 357	679.3596	1356.7046	1356.7038	0.57	0	72	3.9e-07	1	U	R.HVDYVVDQVVGK.L
4503	346 – 357	679.3596	1356.7046	1356.7038	0.57	0	58	5.6e-06	1	U	R.HVDYVVDQVVGK.L
4504	346 – 357	679.3601	1356.7057	1356.7038	1.42	0	75	1.2e-07	1	U	R.HVDYVVDQVVGK.L
521	358 – 364	414.7815	827.5484	827.5480	0.47	1	26	0.018	1	U	K.LIEVVKK.K
2916	368 – 378	387.2320	1158.6743	1158.6761	-1.54	0	43	0.0011	1	U	K.AGVSVKPFQVK.N
2917	368 – 378	580.3448	1158.6750	1158.6761	-0.91	0	33	0.0034	1	U	K.AGVSVKPFQVK.N
2921	368 – 378	387.2326	1158.6759	1158.6761	-0.16	0	31	0.019	1	U	K.AGVSVKPFQVK.N
2923	368 – 378	580.3453	1158.6760	1158.6761	-0.12	0	40	0.0025	1	U	K.AGVSVKPFQVK.N
2926	368 – 378	580.3454	1158.6763	1158.6761	0.18	0	21	0.049	1	U	K.AGVSVKPFQVK.N
2927	368 – 378	580.3456	1158.6767	1158.6761	0.56	0	51	0.00022	1	U	K.AGVSVKPFQVK.N

Query	Start – End	Observed	Mr(expt)	Mr(calc)	ppm	M	Score	Expect	Rank	U	Peptide
2928	368 – 378	387.2329	1158.6769	1158.6761	0.66	0	27	0.0095	1	U	K.AGVSVKPFQVK.N
2932	368 – 378	580.3465	1158.6784	1158.6761	2.01	0	23	0.05	1	U	K.AGVSVKPFQVK.N
11429	379 – 399	859.4247	2575.2522	2575.2529	-0.27	0	63	4e-05	1	U	K.NHIWVFINCLIENPTFDSQTK.E
11430	379 – 399	859.4248	2575.2526	2575.2529	-0.13	0	58	0.00026	1	U	K.NHIWVFINCLIENPTFDSQTK.E
2136	438 – 446	539.3060	1076.5974	1076.5978	-0.39	1	26	0.018	1	U	K.FKAQTQLNK.K
406	440 – 446	401.7242	801.4339	801.4344	-0.67	0	32	0.03	1	U	K.AQTQLNK.K
410	440 – 446	401.7252	801.4359	801.4344	1.80	0	36	0.01	1	U	K.AQTQLNK.K
1061	440 – 447	465.7718	929.5291	929.5294	-0.30	1	32	0.0096	1	U	K.AQTQLNKK.C
1337	462 – 471	488.2224	974.4301	974.4305	-0.36	0	59	5.6e-05	1	U	K.LDDANDAGGK.H
1338	462 – 471	488.2224	974.4303	974.4305	-0.17	0	65	1.6e-05	1	U	K.LDDANDAGGK.H
1339	462 – 471	488.2226	974.4306	974.4305	0.11	0	65	1.3e-05	1	U	K.LDDANDAGGK.H
1340	462 – 471	488.2228	974.4311	974.4305	0.63	0	36	0.0029	1	U	K.LDDANDAGGK.H
1341	462 – 471	488.2230	974.4314	974.4305	0.94	0	26	0.026	1	U	K.LDDANDAGGK.H
1342	462 – 471	488.2236	974.4326	974.4305	2.17	0	34	0.0037	1	U	K.LDDANDAGGK.H
8179	472 – 487	887.4379	1772.8613	1772.8614	-0.069	0	97	8.9e-09	1	U	K.HSLECTLILTEGDSAK.S
8180	472 – 487	591.9612	1772.8616	1772.8614	0.097	0	31	0.016	1	U	K.HSLECTLILTEGDSAK.S
2601	488 – 499	564.8399	1127.6653	1127.6663	-0.88	0	33	0.0034	1	U	K.SLAVSGLGVIGR.D
2602	488 – 499	564.8400	1127.6653	1127.6663	-0.81	0	48	0.00011	1	U	K.SLAVSGLGVIGR.D
2603	488 – 499	564.8401	1127.6656	1127.6663	-0.56	0	60	3.2e-05	1	U	K.SLAVSGLGVIGR.D
2604	488 – 499	564.8401	1127.6657	1127.6663	-0.49	0	69	9.1e-07	1	U	K.SLAVSGLGVIGR.D
2605	488 – 499	564.8402	1127.6658	1127.6663	-0.44	0	67	8.3e-06	1	U	K.SLAVSGLGVIGR.D
2606	488 – 499	564.8402	1127.6658	1127.6663	-0.40	0	54	0.00013	1	U	K.SLAVSGLGVIGR.D
2608	488 – 499	564.8402	1127.6659	1127.6663	-0.33	0	50	0.00014	1	U	K.SLAVSGLGVIGR.D
2609	488 – 499	564.8403	1127.6660	1127.6663	-0.21	0	35	0.00053	1	U	K.SLAVSGLGVIGR.D
2610	488 – 499	564.8403	1127.6661	1127.6663	-0.13	0	69	1.8e-06	1	U	K.SLAVSGLGVIGR.D
2611	488 – 499	564.8403	1127.6661	1127.6663	-0.12	0	65	3.7e-06	1	U	K.SLAVSGLGVIGR.D
2612	488 – 499	564.8404	1127.6663	1127.6663	0.025	0	45	0.00024	1	U	K.SLAVSGLGVIGR.D
2613	488 – 499	564.8404	1127.6663	1127.6663	0.025	0	67	6.7e-06	1	U	K.SLAVSGLGVIGR.D
2614	488 – 499	376.8960	1127.6663	1127.6663	0.028	0	46	0.00091	1	U	K.SLAVSGLGVIGR.D
2615	488 – 499	564.8404	1127.6663	1127.6663	0.043	0	65	4.4e-06	1	U	K.SLAVSGLGVIGR.D
2616	488 – 499	564.8406	1127.6666	1127.6663	0.27	0	53	5.2e-05	1	U	K.SLAVSGLGVIGR.D
2617	488 – 499	564.8406	1127.6667	1127.6663	0.36	0	62	5.7e-06	1	U	K.SLAVSGLGVIGR.D
2618	488 – 499	564.8406	1127.6667	1127.6663	0.43	0	57	1.7e-05	1	U	K.SLAVSGLGVIGR.D
2619	488 – 499	564.8408	1127.6669	1127.6663	0.61	0	41	0.00021	1	U	K.SLAVSGLGVIGR.D
2620	488 – 499	564.8410	1127.6675	1127.6663	1.09	0	29	0.0018	1	U	K.SLAVSGLGVIGR.D
2621	488 – 499	564.8411	1127.6677	1127.6663	1.30	0	40	0.00088	1	U	K.SLAVSGLGVIGR.D
2547	500 – 508	561.8068	1121.5990	1121.5982	0.76	1	32	0.038	1	U	R.DRYGVFPLR.G
641	502 – 508	426.2422	850.4698	850.4701	-0.39	0	35	0.0097	1		R.YGVFPLR.G
645	502 – 508	426.2423	850.4700	850.4701	-0.16	0	30	0.01	1		R.YGVFPLR.G
646	502 – 508	426.2425	850.4704	850.4701	0.38	0	19	0.037	1		R.YGVFPLR.G
647	502 – 508	426.2425	850.4705	850.4701	0.48	0	19	0.047	1		R.YGVFPLR.G
6010	521 – 533	765.4036	1528.7926	1528.7919	0.51	0	90	7.5e-08	1		K.QIMENAEINNIIK.I
6011	521 – 533	510.6049	1528.7928	1528.7919	0.64	0	47	0.0016	1		K.QIMENAEINNIIK.I
6209	521 – 533	773.4001	1544.7857	1544.7868	-0.70	0	71	4.3e-06	1		K.QIMENAEINNIIK.I + Oxidation (M)
6210	521 – 533	773.4005	1544.7864	1544.7868	-0.27	0	73	2.2e-06	1		K.QIMENAEINNIIK.I + Oxidation (M)
6211	521 – 533	773.4005	1544.7865	1544.7868	-0.17	0	52	0.0001	1		K.QIMENAEINNIIK.I + Oxidation (M)
6212	521 – 533	773.4007	1544.7868	1544.7868	0.025	0	61	2.1e-05	1		K.QIMENAEINNIIK.I + Oxidation (M)
6213	521 – 533	773.4010	1544.7875	1544.7868	0.44	0	87	1.1e-07	1		K.QIMENAEINNIIK.I + Oxidation (M)
477	534 – 540	410.7499	819.4852	819.4854	-0.22	0	20	0.032	1		K.IVGLQYK.K
480	534 – 540	410.7506	819.4866	819.4854	1.44	0	32	0.0072	1		K.IVGLQYK.K
2880	541 – 550	578.2797	1154.5449	1154.5455	-0.55	1	31	0.0012	1	U	K.KSYDDAESLK.T
2883	541 – 550	578.2801	1154.5457	1154.5455	0.20	1	33	0.001	1	U	K.KSYDDAESLK.T
2884	541 – 550	578.2808	1154.5471	1154.5455	1.43	1	30	0.0067	1	U	K.KSYDDAESLK.T
1713	542 – 550	514.2323	1026.4501	1026.4505	-0.43	0	19	0.026	1	U	K.SYDDAESLK.T
1715	542 – 550	514.2325	1026.4504	1026.4505	-0.095	0	30	0.0035	1	U	K.SYDDAESLK.T
1716	542 – 550	514.2326	1026.4507	1026.4505	0.18	0	43	0.00022	1	U	K.SYDDAESLK.T
1717	542 – 550	514.2327	1026.4508	1026.4505	0.22	0	25	0.041	1	U	K.SYDDAESLK.T
4773	542 – 553	699.3488	1396.6830	1396.6834	-0.29	1	55	1.4e-05	1	U	K.SYDDAESLKTLR.Y
4774	542 – 553	699.3490	1396.6833	1396.6834	-0.034	1	48	9.1e-05	1	U	K.SYDDAESLKTLR.Y
7798	557 – 571	866.4054	1730.7963	1730.7968	-0.28	0	53	7.9e-05	1		K.IMIMTDQDQDGSHIK.G
7800	557 – 571	577.9395	1730.7968	1730.7968	0.023	0	31	0.013	1		K.IMIMTDQDQDGSHIK.G
7806	557 – 571	866.4064	1730.7983	1730.7968	0.89	0	80	3.2e-07	1		K.IMIMTDQDQDGSHIK.G
7807	557 – 571	866.4067	1730.7988	1730.7968	1.16	0	73	6e-06	1		K.IMIMTDQDQDGSHIK.G
7944	557 – 571	874.4024	1746.7903	1746.7917	-0.76	0	56	6.5e-05	1		K.IMIMTDQDQDGSHIK.G + Oxidation (M)
7945	557 – 571	583.2707	1746.7904	1746.7917	-0.75	0	40	0.011	1		K.IMIMTDQDQDGSHIK.G + Oxidation (M)
7948	557 – 571	874.4028	1746.7911	1746.7917	-0.33	0	77	2.2e-06	1		K.IMIMTDQDQDGSHIK.G + Oxidation (M)
7949	557 – 571	583.2710	1746.7911	1746.7917	-0.32	0	47	0.0021	1		K.IMIMTDQDQDGSHIK.G + Oxidation (M)
7950	557 – 571	874.4030	1746.7913	1746.7917	-0.19	0	57	3.7e-05	1		K.IMIMTDQDQDGSHIK.G + Oxidation (M)
7951	557 – 571	583.2711	1746.7914	1746.7917	-0.17	0	41	0.0082	1		K.IMIMTDQDQDGSHIK.G + Oxidation (M)
7952	557 – 571	874.4030	1746.7914	1746.7917	-0.14	0	82	1.7e-07	1		K.IMIMTDQDQDGSHIK.G + Oxidation (M)
7953	557 – 571	583.2711	1746.7916	1746.7917	-0.063	0	42	0.0074	1		K.IMIMTDQDQDGSHIK.G + Oxidation (M)
7954	557 – 571	874.4031	1746.7916	1746.7917	-0.060	0	79	1.3e-06	1		K.IMIMTDQDQDGSHIK.G + Oxidation (M)
7956	557 – 571	583.2712	1746.7916	1746.7917	-0.029	0	36	0.027	1		K.IMIMTDQDQDGSHIK.G + Oxidation (M)
7958	557 – 571	583.2713	1746.7922	1746.7917	0.28	0	32	0.0045	1		K.IMIMTDQDQDGSHIK.G + Oxidation (M)
7960	557 – 571	583.2717	1746.7934	1746.7917	0.98	0	47	0.00092	1		K.IMIMTDQDQDGSHIK.G + Oxidation (M)
8086	557 – 571	588.6018	1762.7834	1762.7866	-1.80	0	31	0.0053	1		K.IMIMTDQDQDGSHIK.G + 2 Oxidation (M)

Query	Start – End	Observed	Mr(expt)	Mr(calc)	ppm	M	Score	Expect	Rank	U	Peptide
8087	557 – 571	588.6020	1762.7843	1762.7866	-1.32	0	29	0.0091	1		K.IMIMTDQDQDGSHIK.G + 2 Oxidation (M)
8089	557 – 571	882.3997	1762.7848	1762.7866	-0.99	0	55	4.3e-05	1		K.IMIMTDQDQDGSHIK.G + 2 Oxidation (M)
8090	557 – 571	588.6023	1762.7852	1762.7866	-0.81	0	55	9.4e-05	1		K.IMIMTDQDQDGSHIK.G + 2 Oxidation (M)
8091	557 – 571	882.3999	1762.7852	1762.7866	-0.80	0	45	0.00042	1		K.IMIMTDQDQDGSHIK.G + 2 Oxidation (M)
8092	557 – 571	588.6024	1762.7853	1762.7866	-0.73	0	38	0.00091	1		K.IMIMTDQDQDGSHIK.G + 2 Oxidation (M)
8093	557 – 571	882.4000	1762.7855	1762.7866	-0.63	0	56	0.00026	1		K.IMIMTDQDQDGSHIK.G + 2 Oxidation (M)
8094	557 – 571	588.6026	1762.7858	1762.7866	-0.44	0	51	0.00021	1		K.IMIMTDQDQDGSHIK.G + 2 Oxidation (M)
8095	557 – 571	882.4005	1762.7864	1762.7866	-0.10	0	62	5.1e-05	1		K.IMIMTDQDQDGSHIK.G + 2 Oxidation (M)
8096	557 – 571	882.4006	1762.7866	1762.7866	-0.0017	0	71	7.2e-06	1		K.IMIMTDQDQDGSHIK.G + 2 Oxidation (M)
8097	557 – 571	882.4006	1762.7867	1762.7866	0.044	0	67	1.2e-05	1		K.IMIMTDQDQDGSHIK.G + 2 Oxidation (M)
8098	557 – 571	588.6028	1762.7867	1762.7866	0.074	0	58	6.3e-05	1		K.IMIMTDQDQDGSHIK.G + 2 Oxidation (M)
8099	557 – 571	882.4007	1762.7868	1762.7866	0.12	0	60	0.00011	1		K.IMIMTDQDQDGSHIK.G + 2 Oxidation (M)
8101	557 – 571	588.6029	1762.7870	1762.7866	0.21	0	39	0.0012	1		K.IMIMTDQDQDGSHIK.G + 2 Oxidation (M)
8102	557 – 571	588.6030	1762.7870	1762.7866	0.24	0	22	0.015	1		K.IMIMTDQDQDGSHIK.G + 2 Oxidation (M)
8103	557 – 571	882.4008	1762.7870	1762.7866	0.26	0	69	9.3e-06	1		K.IMIMTDQDQDGSHIK.G + 2 Oxidation (M)
8105	557 – 571	588.6031	1762.7876	1762.7866	0.55	0	32	0.0054	1		K.IMIMTDQDQDGSHIK.G + 2 Oxidation (M)
8106	557 – 571	588.6032	1762.7877	1762.7866	0.64	0	30	0.0069	1		K.IMIMTDQDQDGSHIK.G + 2 Oxidation (M)
9118	572 – 587	634.6954	1901.0644	1901.0676	-1.67	0	22	0.012	1	U	K.GLLINFIHHNWPSLLK.H
9119	572 – 587	951.5400	1901.0653	1901.0676	-1.16	0	39	0.00024	1	U	K.GLLINFIHHNWPSLLK.H
9123	572 – 587	634.6964	1901.0673	1901.0676	-0.14	0	19	0.025	1	U	K.GLLINFIHHNWPSLLK.H
9125	572 – 587	634.6964	1901.0673	1901.0676	-0.11	0	25	0.033	1	U	K.GLLINFIHHNWPSLLK.H
9126	572 – 587	951.5410	1901.0674	1901.0676	-0.065	0	38	0.00033	1	U	K.GLLINFIHHNWPSLLK.H
9132	572 – 587	634.6967	1901.0683	1901.0676	0.41	0	33	0.015	1	U	K.GLLINFIHHNWPSLLK.H
9133	572 – 587	634.6968	1901.0685	1901.0676	0.49	0	23	0.01	1	U	K.GLLINFIHHNWPSLLK.H
6012	588 – 600	510.6162	1528.8267	1528.8290	-1.49	0	47	0.00018	1	U	K.HGFLEEFITPIVK.A
6013	588 – 600	765.4209	1528.8272	1528.8290	-1.14	0	49	2.3e-05	1	U	K.HGFLEEFITPIVK.A
6015	588 – 600	765.4212	1528.8278	1528.8290	-0.77	0	51	1.7e-05	1	U	K.HGFLEEFITPIVK.A
6016	588 – 600	510.6167	1528.8283	1528.8290	-0.41	0	21	0.026	1	U	K.HGFLEEFITPIVK.A
6017	588 – 600	765.4215	1528.8284	1528.8290	-0.34	0	72	2.7e-07	1	U	K.HGFLEEFITPIVK.A
6018	588 – 600	510.6168	1528.8285	1528.8290	-0.28	0	40	0.0042	1	U	K.HGFLEEFITPIVK.A
6019	588 – 600	765.4216	1528.8286	1528.8290	-0.26	0	29	0.023	1	U	K.HGFLEEFITPIVK.A
6020	588 – 600	510.6168	1528.8286	1528.8290	-0.26	0	49	0.00015	1	U	K.HGFLEEFITPIVK.A
6021	588 – 600	765.4216	1528.8286	1528.8290	-0.25	0	84	1.5e-08	1	U	K.HGFLEEFITPIVK.A
6022	588 – 600	510.6168	1528.8286	1528.8290	-0.24	0	43	0.0013	1	U	K.HGFLEEFITPIVK.A
6023	588 – 600	510.6168	1528.8286	1528.8290	-0.24	0	38	0.0054	1	U	K.HGFLEEFITPIVK.A
6024	588 – 600	765.4216	1528.8286	1528.8290	-0.21	0	50	2.7e-05	1	U	K.HGFLEEFITPIVK.A
6026	588 – 600	765.4217	1528.8288	1528.8290	-0.10	0	65	2.4e-06	1	U	K.HGFLEEFITPIVK.A
6027	588 – 600	765.4217	1528.8289	1528.8290	-0.063	0	64	9.5e-07	1	U	K.HGFLEEFITPIVK.A
6028	588 – 600	510.6169	1528.8289	1528.8290	-0.060	0	25	0.041	1	U	K.HGFLEEFITPIVK.A
6029	588 – 600	765.4217	1528.8289	1528.8290	-0.037	0	51	0.00019	1	U	K.HGFLEEFITPIVK.A
6030	588 – 600	510.6170	1528.8290	1528.8290	0.038	0	45	0.001	1	U	K.HGFLEEFITPIVK.A
6032	588 – 600	510.6170	1528.8291	1528.8290	0.058	0	46	0.00031	1	U	K.HGFLEEFITPIVK.A
6033	588 – 600	765.4218	1528.8291	1528.8290	0.094	0	20	0.031	1	U	K.HGFLEEFITPIVK.A
6034	588 – 600	765.4218	1528.8291	1528.8290	0.094	0	61	4.4e-06	1	U	K.HGFLEEFITPIVK.A
6035	588 – 600	510.6170	1528.8291	1528.8290	0.12	0	49	0.00066	1	U	K.HGFLEEFITPIVK.A
6037	588 – 600	765.4219	1528.8292	1528.8290	0.13	0	82	2.6e-08	1	U	K.HGFLEEFITPIVK.A
6038	588 – 600	765.4219	1528.8292	1528.8290	0.19	0	87	8.4e-09	1	U	K.HGFLEEFITPIVK.A
6039	588 – 600	510.6170	1528.8293	1528.8290	0.19	0	36	0.011	1	U	K.HGFLEEFITPIVK.A
6040	588 – 600	765.4219	1528.8293	1528.8290	0.21	0	45	0.001	1	U	K.HGFLEEFITPIVK.A
6041	588 – 600	765.4219	1528.8293	1528.8290	0.23	0	87	1.4e-08	1	U	K.HGFLEEFITPIVK.A
6042	588 – 600	765.4219	1528.8293	1528.8290	0.23	0	87	2.8e-08	1	U	K.HGFLEEFITPIVK.A
6043	588 – 600	765.4220	1528.8294	1528.8290	0.28	0	72	2.6e-07	1	U	K.HGFLEEFITPIVK.A
6044	588 – 600	765.4220	1528.8294	1528.8290	0.29	0	27	0.012	1	U	K.HGFLEEFITPIVK.A
6045	588 – 600	765.4220	1528.8294	1528.8290	0.29	0	35	0.0005	1	U	K.HGFLEEFITPIVK.A
6046	588 – 600	510.6171	1528.8294	1528.8290	0.31	0	28	0.014	1	U	K.HGFLEEFITPIVK.A
6047	588 – 600	510.6171	1528.8295	1528.8290	0.33	0	36	0.0034	1	U	K.HGFLEEFITPIVK.A
6049	588 – 600	765.4220	1528.8295	1528.8290	0.38	0	28	0.01	1	U	K.HGFLEEFITPIVK.A
6050	588 – 600	765.4221	1528.8296	1528.8290	0.40	0	35	0.0019	1	U	K.HGFLEEFITPIVK.A
6052	588 – 600	510.6173	1528.8301	1528.8290	0.72	0	21	0.032	1	U	K.HGFLEEFITPIVK.A
6053	588 – 600	765.4225	1528.8304	1528.8290	0.97	0	56	5.2e-06	1	U	K.HGFLEEFITPIVK.A
10220	604 – 620	1080.5165	2159.0184	2159.0211	-1.25	1	96	1.3e-08	1	U	K.NKQELSFYSIPEFDEWK.K
10221	604 – 620	1080.5173	2159.0201	2159.0211	-0.45	1	61	1.4e-05	1	U	K.NKQELSFYSIPEFDEWK.K
10222	604 – 620	720.6808	2159.0204	2159.0211	-0.32	1	29	0.024	1	U	K.NKQELSFYSIPEFDEWK.K
10655	604 – 621	1144.5648	2287.1151	2287.1161	-0.42	2	70	4.5e-07	1	U	K.NKQELSFYSIPEFDEWKK.H
10657	604 – 621	572.7863	2287.1161	2287.1161	0.034	2	29	0.045	1	U	K.NKQELSFYSIPEFDEWKK.H
10658	604 – 621	763.3795	2287.1167	2287.1161	0.28	2	53	0.00015	1	U	K.NKQELSFYSIPEFDEWKK.H
9254	606 – 620	959.4488	1916.8830	1916.8832	-0.13	0	55	6.1e-05	1	U	K.QELSFYSIPEFDEWK.K
9791	606 – 621	1023.4936	2044.9726	2044.9782	-2.71	1	19	0.02	1	U	K.QELSFYSIPEFDEWKK.H
9792	606 – 621	682.6665	2044.9777	2044.9782	-0.23	1	31	0.0059	1	U	K.QELSFYSIPEFDEWKK.H
9793	606 – 621	1023.4964	2044.9782	2044.9782	0.014	1	37	0.0014	1	U	K.QELSFYSIPEFDEWKK.H
857	621 – 627	448.7512	895.4877	895.4875	0.24	1	31	0.024	1	U	K.KHIENQK.A
1973	647 – 654	530.7231	1059.4316	1059.4331	-1.39	0	24	0.006	1	U	K.EYFADMER.H
1975	647 – 654	530.7237	1059.4329	1059.4331	-0.18	0	19	0.026	1	U	K.EYFADMER.H
1976	647 – 654	530.7239	1059.4332	1059.4331	0.12	0	19	0.035	1	U	K.EYFADMER.H
1977	647 – 654	530.7242	1059.4339	1059.4331	0.76	0	20	0.029	1	U	K.EYFADMER.H
1978	647 – 654	530.7246	1059.4347	1059.4331	1.48	0	30	0.0053	1	U	K.EYFADMER.H
2122	647 – 654	538.7210	1075.4275	1075.4280	-0.53	0	18	0.034	1	U	K.EYFADMER.H

Query	Start – End	Observed	Mr(expt)	Mr(calc)	ppm	M	Score	Expect	Rank	U	Peptide
											+ Oxidation (M)
2126	647 – 654	538.7214	1075.4282	1075.4280	0.19	0	22	0.036	1	U	K.EYFADMER.H + Oxidation (M)
7237	661 – 676	834.9090	1667.8035	1667.8042	-0.45	0	75	2.8e-07	1	U	R.YAGPEDDAAITLAFSK.K
7238	661 – 676	834.9090	1667.8035	1667.8042	-0.44	0	52	1.9e-05	1	U	R.YAGPEDDAAITLAFSK.K
7239	661 – 676	834.9091	1667.8036	1667.8042	-0.36	0	58	4e-06	1	U	R.YAGPEDDAAITLAFSK.K
7240	661 – 676	834.9091	1667.8037	1667.8042	-0.32	0	58	4.8e-06	1	U	R.YAGPEDDAAITLAFSK.K
7241	661 – 676	834.9092	1667.8039	1667.8042	-0.23	0	25	0.0054	1	U	R.YAGPEDDAAITLAFSK.K
7242	661 – 676	834.9092	1667.8039	1667.8042	-0.19	0	84	7.5e-08	1	U	R.YAGPEDDAAITLAFSK.K
7243	661 – 676	834.9094	1667.8042	1667.8042	-0.010	0	84	1.6e-08	1	U	R.YAGPEDDAAITLAFSK.K
7244	661 – 676	834.9094	1667.8042	1667.8042	0.0018	0	84	2.8e-08	1	U	R.YAGPEDDAAITLAFSK.K
7245	661 – 676	834.9094	1667.8043	1667.8042	0.014	0	69	8.8e-07	1	U	R.YAGPEDDAAITLAFSK.K
7247	661 – 676	834.9095	1667.8044	1667.8042	0.12	0	59	5e-06	1	U	R.YAGPEDDAAITLAFSK.K
7248	661 – 676	556.9421	1667.8046	1667.8042	0.21	0	48	0.00023	1	U	R.YAGPEDDAAITLAFSK.K
7249	661 – 676	834.9096	1667.8047	1667.8042	0.30	0	79	3.7e-08	1	U	R.YAGPEDDAAITLAFSK.K
7250	661 – 676	834.9097	1667.8048	1667.8042	0.33	0	91	4.4e-09	1	U	R.YAGPEDDAAITLAFSK.K
7251	661 – 676	834.9097	1667.8049	1667.8042	0.40	0	72	1.7e-07	1	U	R.YAGPEDDAAITLAFSK.K
7252	661 – 676	834.9097	1667.8049	1667.8042	0.41	0	63	1.8e-06	1	U	R.YAGPEDDAAITLAFSK.K
7253	661 – 676	834.9098	1667.8050	1667.8042	0.45	0	68	6e-07	1	U	R.YAGPEDDAAITLAFSK.K
7254	661 – 676	834.9098	1667.8050	1667.8042	0.45	0	74	2.4e-07	1	U	R.YAGPEDDAAITLAFSK.K
7255	661 – 676	834.9098	1667.8050	1667.8042	0.46	0	70	5.4e-07	1	U	R.YAGPEDDAAITLAFSK.K
7256	661 – 676	834.9098	1667.8050	1667.8042	0.48	0	54	1e-05	1	U	R.YAGPEDDAAITLAFSK.K
7257	661 – 676	834.9100	1667.8055	1667.8042	0.77	0	66	7.2e-07	1	U	R.YAGPEDDAAITLAFSK.K
7258	661 – 676	834.9114	1667.8082	1667.8042	2.35	0	72	6.2e-07	1	U	R.YAGPEDDAAITLAFSK.K
5348	683 – 693	490.2348	1467.6825	1467.6816	0.60	1	29	0.013	1		R.KEWLTNFMEDR.R
5476	683 – 693	495.5661	1483.6764	1483.6765	-0.13	1	26	0.017	1		R.KEWLTNFMEDR.R + Oxidation (M)
5478	683 – 693	495.5662	1483.6768	1483.6765	0.15	1	26	0.011	1		R.KEWLTNFMEDR.R + Oxidation (M)
5479	683 – 693	742.8457	1483.6769	1483.6765	0.22	1	54	2.8e-05	1		R.KEWLTNFMEDR.R + Oxidation (M)
5480	683 – 693	495.5663	1483.6770	1483.6765	0.30	1	41	0.00074	1		R.KEWLTNFMEDR.R + Oxidation (M)
5481	683 – 693	495.5663	1483.6770	1483.6765	0.32	1	33	0.0028	1		R.KEWLTNFMEDR.R + Oxidation (M)
5482	683 – 693	742.8458	1483.6771	1483.6765	0.39	1	24	0.014	1		R.KEWLTNFMEDR.R + Oxidation (M)
5483	683 – 693	495.5663	1483.6771	1483.6765	0.40	1	31	0.016	1		R.KEWLTNFMEDR.R + Oxidation (M)
5484	683 – 693	742.8459	1483.6772	1483.6765	0.43	1	28	0.0076	1		R.KEWLTNFMEDR.R + Oxidation (M)
5485	683 – 693	742.8460	1483.6774	1483.6765	0.56	1	38	0.0014	1		R.KEWLTNFMEDR.R + Oxidation (M)
5486	683 – 693	742.8463	1483.6781	1483.6765	1.04	1	30	0.014	1		R.KEWLTNFMEDR.R + Oxidation (M)
5487	683 – 693	742.8469	1483.6793	1483.6765	1.88	1	23	0.016	1		R.KEWLTNFMEDR.R + Oxidation (M)
6924	683 – 694	812.8984	1623.7821	1623.7827	-0.36	2	38	0.0036	1		R.KEWLTNFMEDRR.Q
4394	684 – 693	670.8006	1339.5866	1339.5867	-0.016	0	39	0.00032	1		K.EWLTNFMEDR.R
4395	684 – 693	670.8007	1339.5868	1339.5867	0.10	0	49	0.00025	1		K.EWLTNFMEDR.R
4479	684 – 693	678.7978	1355.5811	1355.5816	-0.33	0	33	0.0025	1		K.EWLTNFMEDR.R + Oxidation (M)
4480	684 – 693	678.7980	1355.5815	1355.5816	-0.075	0	27	0.0054	1		K.EWLTNFMEDR.R + Oxidation (M)
4482	684 – 693	678.7983	1355.5820	1355.5816	0.32	0	30	0.0051	1		K.EWLTNFMEDR.R + Oxidation (M)
4483	684 – 693	678.7985	1355.5824	1355.5816	0.62	0	36	0.0078	1		K.EWLTNFMEDR.R + Oxidation (M)
8671	697 – 712	610.9991	1829.9754	1829.9788	-1.84	1	56	5.7e-05	1	U	R.RLHGLPEQFLYGTATK.H
8672	697 – 712	610.9994	1829.9764	1829.9788	-1.31	1	48	0.00034	1	U	R.RLHGLPEQFLYGTATK.H
8673	697 – 712	610.9995	1829.9766	1829.9788	-1.23	1	44	0.00027	1	U	R.RLHGLPEQFLYGTATK.H
8674	697 – 712	610.9995	1829.9767	1829.9788	-1.15	1	33	0.0023	1	U	R.RLHGLPEQFLYGTATK.H
8675	697 – 712	610.9996	1829.9770	1829.9788	-1.00	1	23	0.016	1	U	R.RLHGLPEQFLYGTATK.H
8678	697 – 712	610.9998	1829.9776	1829.9788	-0.64	1	27	0.018	1	U	R.RLHGLPEQFLYGTATK.H
8679	697 – 712	915.9963	1829.9780	1829.9788	-0.46	1	88	1e-08	1	U	R.RLHGLPEQFLYGTATK.H
8680	697 – 712	611.0000	1829.9780	1829.9788	-0.43	1	35	0.0052	1	U	R.RLHGLPEQFLYGTATK.H
8682	697 – 712	611.0002	1829.9788	1829.9788	0.014	1	33	0.0064	1	U	R.RLHGLPEQFLYGTATK.H
8683	697 – 712	611.0002	1829.9789	1829.9788	0.031	1	48	0.00019	1	U	R.RLHGLPEQFLYGTATK.H
8685	697 – 712	611.0004	1829.9793	1829.9788	0.24	1	65	1.5e-05	1	U	R.RLHGLPEQFLYGTATK.H
8687	697 – 712	611.0005	1829.9796	1829.9788	0.41	1	30	0.0044	1	U	R.RLHGLPEQFLYGTATK.H
8688	697 – 712	611.0005	1829.9798	1829.9788	0.54	1	22	0.017	1	U	R.RLHGLPEQFLYGTATK.H
8689	697 – 712	915.9972	1829.9798	1829.9788	0.55	1	82	6e-08	1	U	R.RLHGLPEQFLYGTATK.H
7292	698 – 712	837.9453	1673.8760	1673.8777	-1.01	0	39	0.00026	1	U	R.LHGLPEQFLYGTATK.H
7293	698 – 712	837.9453	1673.8761	1673.8777	-0.95	0	24	0.007	1	U	R.LHGLPEQFLYGTATK.H
7294	698 – 712	558.9660	1673.8763	1673.8777	-0.86	0	35	0.0023	1	U	R.LHGLPEQFLYGTATK.H
7295	698 – 712	837.9454	1673.8763	1673.8777	-0.86	0	18	0.031	1	U	R.LHGLPEQFLYGTATK.H
7296	698 – 712	837.9456	1673.8766	1673.8777	-0.65	0	19	0.018	1	U	R.LHGLPEQFLYGTATK.H
7298	698 – 712	837.9456	1673.8767	1673.8777	-0.58	0	28	0.0025	1	U	R.LHGLPEQFLYGTATK.H
7299	698 – 712	558.9662	1673.8767	1673.8777	-0.57	0	37	0.0022	1	U	R.LHGLPEQFLYGTATK.H
7300	698 – 712	837.9458	1673.8770	1673.8777	-0.41	0	20	0.014	1	U	R.LHGLPEQFLYGTATK.H
7301	698 – 712	837.9458	1673.8770	1673.8777	-0.40	0	28	0.0025	1	U	R.LHGLPEQFLYGTATK.H
7304	698 – 712	837.9458	1673.8771	1673.8777	-0.34	0	27	0.0031	1	U	R.LHGLPEQFLYGTATK.H
7305	698 – 712	837.9459	1673.8772	1673.8777	-0.32	0	69	1.1e-06	1	U	R.LHGLPEQFLYGTATK.H
7306	698 – 712	837.9459	1673.8773	1673.8777	-0.23	0	39	0.00032	1	U	R.LHGLPEQFLYGTATK.H
7307	698 – 712	837.9459	1673.8773	1673.8777	-0.23	0	57	4.4e-06	1	U	R.LHGLPEQFLYGTATK.H
7308	698 – 712	837.9459	1673.8773	1673.8777	-0.23	0	32	0.00092	1	U	R.LHGLPEQFLYGTATK.H
7309	698 – 712	837.9459	1673.8773	1673.8777	-0.22	0	61	3.8e-06	1	U	R.LHGLPEQFLYGTATK.H
7312	698 – 712	558.9665	1673.8776	1673.8777	-0.089	0	27	0.031	1	U	R.LHGLPEQFLYGTATK.H
7313	698 – 712	558.9665	1673.8776	1673.8777	-0.089	0	22	0.019	1	U	R.LHGLPEQFLYGTATK.H
7314	698 – 712	837.9461	1673.8776	1673.8777	-0.068	0	25	0.0048	1	U	R.LHGLPEQFLYGTATK.H
7316	698 – 712	558.9665	1673.8776	1673.8777	-0.053	0	28	0.012	1	U	R.LHGLPEQFLYGTATK.H
7317	698 – 712	558.9665	1673.8777	1673.8777	-0.017	0	38	0.0027	1	U	R.LHGLPEQFLYGTATK.H
7318	698 – 712	558.9666	1673.8779	1673.8777	0.11	0	45	0.00077	1	U	R.LHGLPEQFLYGTATK.H
7319	698 – 712	558.9666	1673.8779	1673.8777	0.13	0	48	0.00032	1	U	R.LHGLPEQFLYGTATK.H

Query	Start – End	Observed	Mr(expt)	Mr(calc)	ppm	M	Score	Expect	Rank	U	Peptide
7320	698 – 712	558.9666	1673.8780	1673.8777	0.16	0	32	0.012	1	U	R.LHGLPEQFLYGTATK.H
7321	698 – 712	558.9666	1673.8780	1673.8777	0.16	0	34	0.013	1	U	R.LHGLPEQFLYGTATK.H
7323	698 – 712	837.9463	1673.8780	1673.8777	0.17	0	26	0.0038	1	U	R.LHGLPEQFLYGTATK.H
7324	698 – 712	837.9463	1673.8780	1673.8777	0.20	0	16	0.029	1	U	R.LHGLPEQFLYGTATK.H
7325	698 – 712	558.9666	1673.8781	1673.8777	0.22	0	44	0.00084	1	U	R.LHGLPEQFLYGTATK.H
7326	698 – 712	558.9666	1673.8781	1673.8777	0.23	0	26	0.026	1	U	R.LHGLPEQFLYGTATK.H
7327	698 – 712	558.9667	1673.8782	1673.8777	0.27	0	49	0.001	1	U	R.LHGLPEQFLYGTATK.H
7328	698 – 712	558.9667	1673.8782	1673.8777	0.27	0	32	0.019	1	U	R.LHGLPEQFLYGTATK.H
7329	698 – 712	558.9667	1673.8783	1673.8777	0.34	0	26	0.022	1	U	R.LHGLPEQFLYGTATK.H
7330	698 – 712	558.9667	1673.8784	1673.8777	0.39	0	49	0.00017	1	U	R.LHGLPEQFLYGTATK.H
7331	698 – 712	558.9667	1673.8784	1673.8777	0.39	0	37	0.0037	1	U	R.LHGLPEQFLYGTATK.H
7332	698 – 712	558.9668	1673.8785	1673.8777	0.45	0	35	0.0055	1	U	R.LHGLPEQFLYGTATK.H
7333	698 – 712	837.9467	1673.8789	1673.8777	0.70	0	21	0.011	1	U	R.LHGLPEQFLYGTATK.H
7334	698 – 712	837.9468	1673.8791	1673.8777	0.82	0	25	0.0049	1	U	R.LHGLPEQFLYGTATK.H
7335	698 – 712	837.9469	1673.8793	1673.8777	0.96	0	25	0.0044	1	U	R.LHGLPEQFLYGTATK.H
7336	698 – 712	558.9671	1673.8794	1673.8777	0.99	0	29	0.0061	1	U	R.LHGLPEQFLYGTATK.H
7338	698 – 712	837.9476	1673.8806	1673.8777	1.74	0	17	0.026	1	U	R.LHGLPEQFLYGTATK.H
3726	713 – 722	632.8189	1263.6232	1263.6248	-1.24	0	37	0.014	1	U	K.HLTYNDFINK.E
3727	713 – 722	632.8193	1263.6241	1263.6248	-0.54	0	24	0.036	1	U	K.HLTYNDFINK.E
3729	713 – 722	632.8194	1263.6242	1263.6248	-0.45	0	23	0.023	1	U	K.HLTYNDFINK.E
3730	713 – 722	632.8194	1263.6242	1263.6248	-0.45	0	24	0.0077	1	U	K.HLTYNDFINK.E
3731	713 – 722	632.8194	1263.6243	1263.6248	-0.38	0	24	0.009	1	U	K.HLTYNDFINK.E
3732	713 – 722	422.2154	1263.6243	1263.6248	-0.36	0	34	0.0054	1	U	K.HLTYNDFINK.E
3733	713 – 722	632.8195	1263.6244	1263.6248	-0.32	0	28	0.02	1	U	K.HLTYNDFINK.E
3734	713 – 722	632.8195	1263.6244	1263.6248	-0.27	0	23	0.038	1	U	K.HLTYNDFINK.E
3735	713 – 722	632.8195	1263.6245	1263.6248	-0.19	0	27	0.032	1	U	K.HLTYNDFINK.E
3736	713 – 722	422.2155	1263.6246	1263.6248	-0.15	0	27	0.014	1	U	K.HLTYNDFINK.E
3738	713 – 722	632.8196	1263.6246	1263.6248	-0.097	0	36	0.0055	1	U	K.HLTYNDFINK.E
3739	713 – 722	632.8196	1263.6247	1263.6248	-0.066	0	39	0.00041	1	U	K.HLTYNDFINK.E
3741	713 – 722	632.8197	1263.6248	1263.6248	0.013	0	25	0.018	1	U	K.HLTYNDFINK.E
3742	713 – 722	632.8197	1263.6248	1263.6248	0.061	0	24	0.0088	1	U	K.HLTYNDFINK.E
3743	713 – 722	632.8198	1263.6250	1263.6248	0.17	0	15	0.044	1	U	K.HLTYNDFINK.E
3744	713 – 722	632.8199	1263.6252	1263.6248	0.33	0	30	0.0026	1	U	K.HLTYNDFINK.E
3745	713 – 722	632.8199	1263.6252	1263.6248	0.36	0	30	0.0029	1	U	K.HLTYNDFINK.E
3746	713 – 722	632.8199	1263.6253	1263.6248	0.39	0	30	0.0036	1	U	K.HLTYNDFINK.E
3747	713 – 722	632.8199	1263.6253	1263.6248	0.44	0	39	0.00034	1	U	K.HLTYNDFINK.E
3748	713 – 722	632.8199	1263.6253	1263.6248	0.44	0	22	0.021	1	U	K.HLTYNDFINK.E
3749	713 – 722	632.8200	1263.6254	1263.6248	0.50	0	25	0.0058	1	U	K.HLTYNDFINK.E
3750	713 – 722	632.8201	1263.6256	1263.6248	0.63	0	27	0.0077	1	U	K.HLTYNDFINK.E
3751	713 – 722	632.8201	1263.6256	1263.6248	0.65	0	30	0.0038	1	U	K.HLTYNDFINK.E
3752	713 – 722	632.8201	1263.6256	1263.6248	0.68	0	23	0.0081	1	U	K.HLTYNDFINK.E
3753	713 – 722	632.8201	1263.6256	1263.6248	0.68	0	35	0.0016	1	U	K.HLTYNDFINK.E
3755	713 – 722	632.8202	1263.6258	1263.6248	0.84	0	26	0.005	1	U	K.HLTYNDFINK.E
3756	713 – 722	632.8202	1263.6259	1263.6248	0.87	0	31	0.0037	1	U	K.HLTYNDFINK.E
11724	713 – 734	894.7768	2681.3087	2681.3085	0.082	1	39	0.0011	1	U	K.HLTYNDFINKELILFSNSDNER.S
11725	713 – 734	894.7772	2681.3098	2681.3085	0.48	1	49	9.4e-05	1	U	K.HLTYNDFINKELILFSNSDNER.S
5033	723 – 734	718.8537	1435.6928	1435.6943	-1.02	0	32	0.0081	1		K.ELILFSNSDNER.S
5034	723 – 734	718.8537	1435.6929	1435.6943	-0.96	0	33	0.0063	1		K.ELILFSNSDNER.S
5036	723 – 734	718.8542	1435.6939	1435.6943	-0.25	0	36	0.0042	1		K.ELILFSNSDNER.S
5037	723 – 734	718.8543	1435.6940	1435.6943	-0.21	0	27	0.024	1		K.ELILFSNSDNER.S
5039	723 – 734	718.8544	1435.6943	1435.6943	0.040	0	28	0.033	1		K.ELILFSNSDNER.S
5040	723 – 734	718.8544	1435.6943	1435.6943	0.040	0	40	0.0081	1		K.ELILFSNSDNER.S
5041	723 – 734	718.8545	1435.6945	1435.6943	0.14	0	31	0.0073	1		K.ELILFSNSDNER.S
5042	723 – 734	718.8546	1435.6947	1435.6943	0.26	0	52	0.00016	1		K.ELILFSNSDNER.S
5043	723 – 734	718.8547	1435.6948	1435.6943	0.39	0	36	0.0025	1		K.ELILFSNSDNER.S
5044	723 – 734	718.8547	1435.6948	1435.6943	0.39	0	36	0.0032	1		K.ELILFSNSDNER.S
5045	723 – 734	718.8547	1435.6949	1435.6943	0.42	0	31	0.0063	1		K.ELILFSNSDNER.S
5047	723 – 734	718.8548	1435.6951	1435.6943	0.57	0	34	0.0052	1		K.ELILFSNSDNER.S
5048	723 – 734	718.8549	1435.6952	1435.6943	0.65	0	31	0.013	1		K.ELILFSNSDNER.S
5050	723 – 734	718.8550	1435.6954	1435.6943	0.75	0	36	0.018	1		K.ELILFSNSDNER.S
5052	723 – 734	718.8551	1435.6957	1435.6943	1.00	0	33	0.0084	1		K.ELILFSNSDNER.S
5053	723 – 734	718.8552	1435.6958	1435.6943	1.07	0	28	0.019	1		K.ELILFSNSDNER.S
5054	723 – 734	718.8552	1435.6959	1435.6943	1.13	0	36	0.0015	1		K.ELILFSNSDNER.S
5055	723 – 734	718.8554	1435.6963	1435.6943	1.41	0	31	0.0084	1		K.ELILFSNSDNER.S
5057	723 – 734	718.8569	1435.6992	1435.6943	3.45	0	30	0.0067	1		K.ELILFSNSDNER.S
5629	735 – 748	750.9114	1499.8082	1499.8096	-0.95	0	48	0.00031	1	U	R.SIPSLVDGFKPGQR.K
5638	735 – 748	750.9120	1499.8094	1499.8096	-0.12	0	52	4.9e-05	1	U	R.SIPSLVDGFKPGQR.K
5644	735 – 748	750.9123	1499.8100	1499.8096	0.26	0	41	0.00056	1	U	R.SIPSLVDGFKPGQR.K
5645	735 – 748	750.9123	1499.8100	1499.8096	0.28	0	49	3.6e-05	1	U	R.SIPSLVDGFKPGQR.K
5646	735 – 748	750.9124	1499.8103	1499.8096	0.45	0	36	0.0021	1	U	R.SIPSLVDGFKPGQR.K
5652	735 – 748	750.9129	1499.8112	1499.8096	1.02	0	35	0.00076	1	U	R.SIPSLVDGFKPGQR.K
2409	815 – 825	554.7966	1107.5787	1107.5785	0.20	1	62	1.1e-05	1	U	R.LHGGKDAASPR.Y
4190	826 – 836	658.3567	1314.6989	1314.7006	-1.25	0	43	0.0011	1	U	R.YIFTMLSTLAR.L
4191	826 – 836	658.3570	1314.6994	1314.7006	-0.87	0	39	0.0014	1	U	R.YIFTMLSTLAR.L
4192	826 – 836	658.3572	1314.6999	1314.7006	-0.54	0	38	0.00067	1	U	R.YIFTMLSTLAR.L
4193	826 – 836	658.3574	1314.7003	1314.7006	-0.20	0	25	0.022	1	U	R.YIFTMLSTLAR.L
4194	826 – 836	658.3576	1314.7007	1314.7006	0.100	0	47	0.00011	1	U	R.YIFTMLSTLAR.L
4195	826 – 836	658.3577	1314.7009	1314.7006	0.22	0	46	0.00018	1	U	R.YIFTMLSTLAR.L
4196	826 – 836	658.3577	1314.7009	1314.7006	0.25	0	50	7.1e-05	1	U	R.YIFTMLSTLAR.L
4197	826 – 836	658.3578	1314.7011	1314.7006	0.37	0	34	0.0031	1	U	R.YIFTMLSTLAR.L
4198	826 – 836	658.3579	1314.7012	1314.7006	0.50	0	47	0.00093	1	U	R.YIFTMLSTLAR.L
4199	826 – 836	658.3579	1314.7013	1314.7006	0.53	0	34	0.0049	1	U	R.YIFTMLSTLAR.L
4200	826 – 836	658.3580	1314.7015	1314.7006	0.72	0	50	5.3e-05	1	U	R.YIFTMLSTLAR.L
4201	826 – 836	658.3581	1314.7016	1314.7006	0.75	0	45	0.0003	1	U	R.YIFTMLSTLAR.L
4325	826 – 836	666.3540	1330.6934	1330.6955	-1.57	0	31	0.006	1	U	R.YIFTMLSTLAR.L<br>+ Oxidation (M)
4326	826 – 836	666.3548	1330.6949	1330.6955	-0.41	0	31	0.0026	1	U	R.YIFTMLSTLAR.L<br>+ Oxidation (M)
4329	826 – 836	666.3548	1330.6951	1330.6955	-0.26	0	50	8.5e-05	1	U	R.YIFTMLSTLAR.L<br>+ Oxidation (M)
4330	826 – 836	666.3549	1330.6952	1330.6955	-0.19	0	18	0.045	1	U	R.YIFTMLSTLAR.L<br>+ Oxidation (M)
4331	826 – 836	666.3550	1330.6954	1330.6955	-0.066	0	49	7.9e-05	1	U	R.YIFTMLSTLAR.L

Query	Start - End	Observed	Mr(expt)	Mr(calc)	ppm	M	Score	Expect	Rank	U	Peptide
											+ Oxidation (M)
4332	826 - 836	666.3550	1330.6955	1330.6955	-0.0060	0	35	0.0014	1	U	R.YIFTMLSTLAR.L + Oxidation (M)
4333	826 - 836	666.3550	1330.6955	1330.6955	0.039	0	39	0.00092	1	U	R.YIFTMLSTLAR.L + Oxidation (M)
4334	826 - 836	666.3551	1330.6956	1330.6955	0.099	0	27	0.0069	1	U	R.YIFTMLSTLAR.L + Oxidation (M)
4335	826 - 836	666.3551	1330.6957	1330.6955	0.14	0	54	3.8e-05	1	U	R.YIFTMLSTLAR.L + Oxidation (M)
4336	826 - 836	666.3552	1330.6957	1330.6955	0.19	0	42	0.00025	1	U	R.YIFTMLSTLAR.L + Oxidation (M)
4337	826 - 836	666.3552	1330.6959	1330.6955	0.32	0	20	0.014	1	U	R.YIFTMLSTLAR.L + Oxidation (M)
4339	826 - 836	666.3554	1330.6961	1330.6955	0.49	0	16	0.031	1	U	R.YIFTMLSTLAR.L + Oxidation (M)
4341	826 - 836	666.3555	1330.6964	1330.6955	0.70	0	50	6.7e-05	1	U	R.YIFTMLSTLAR.L + Oxidation (M)
4342	826 - 836	666.3555	1330.6964	1330.6955	0.72	0	32	0.0035	1	U	R.YIFTMLSTLAR.L + Oxidation (M)
4506	837 - 848	679.3895	1356.7644	1356.7653	-0.68	0	23	0.036	1	U	R.LLFPAVDDNLLK.F
4508	837 - 848	679.3896	1356.7647	1356.7653	-0.41	0	36	0.0021	1	U	R.LLFPAVDDNLLK.F
4509	837 - 848	679.3897	1356.7648	1356.7653	-0.35	0	28	0.023	1	U	R.LLFPAVDDNLLK.F
4510	837 - 848	679.3897	1356.7649	1356.7653	-0.27	0	33	0.0042	1	U	R.LLFPAVDDNLLK.F
4511	837 - 848	679.3898	1356.7650	1356.7653	-0.19	0	58	3.5e-05	1	U	R.LLFPAVDDNLLK.F
4512	837 - 848	679.3898	1356.7650	1356.7653	-0.18	0	41	0.0032	1	U	R.LLFPAVDDNLLK.F
4513	837 - 848	679.3899	1356.7652	1356.7653	-0.045	0	24	0.015	1	U	R.LLFPAVDDNLLK.F
4514	837 - 848	679.3899	1356.7652	1356.7653	-0.030	0	46	0.0011	1	U	R.LLFPAVDDNLLK.F
4515	837 - 848	679.3899	1356.7653	1356.7653	-0.015	0	19	0.032	1	U	R.LLFPAVDDNLLK.F
4517	837 - 848	679.3899	1356.7653	1356.7653	0.014	0	41	0.0016	1	U	R.LLFPAVDDNLLK.F
4518	837 - 848	679.3900	1356.7654	1356.7653	0.073	0	36	0.0023	1	U	R.LLFPAVDDNLLK.F
4519	837 - 848	679.3900	1356.7654	1356.7653	0.073	0	48	0.0003	1	U	R.LLFPAVDDNLLK.F
4520	837 - 848	679.3900	1356.7655	1356.7653	0.13	0	41	0.0013	1	U	R.LLFPAVDDNLLK.F
4521	837 - 848	679.3901	1356.7656	1356.7653	0.24	0	25	0.006	1	U	R.LLFPAVDDNLLK.F
4522	837 - 848	679.3901	1356.7656	1356.7653	0.26	0	32	0.0022	1	U	R.LLFPAVDDNLLK.F
4523	837 - 848	679.3901	1356.7657	1356.7653	0.28	0	34	0.0076	1	U	R.LLFPAVDDNLLK.F
4525	837 - 848	679.3903	1356.7660	1356.7653	0.52	0	58	2.4e-05	1	U	R.LLFPAVDDNLLK.F
4526	837 - 848	679.3904	1356.7662	1356.7653	0.66	0	26	0.012	1	U	R.LLFPAVDDNLLK.F
4527	837 - 848	679.3904	1356.7662	1356.7653	0.71	0	28	0.0031	1	U	R.LLFPAVDDNLLK.F
4528	837 - 848	679.3908	1356.7670	1356.7653	1.24	0	48	0.00011	1	U	R.LLFPAVDDNLLK.F
2075	849 - 856	535.7489	1069.4833	1069.4829	0.40	0	35	0.01	1		K.FLYDDNQR.V
629	885 - 891	424.7166	847.4186	847.4188	-0.27	0	36	0.0038	1	U	K.LPNYDAR.E
630	885 - 891	424.7169	847.4193	847.4188	0.63	0	36	0.009	1	U	K.LPNYDAR.E
7905	900 - 914	870.9260	1739.8375	1739.8375	0.017	0	76	5.7e-07	1	U	R.MLDGLDPHPMLPNYK.N
7908	900 - 914	870.9263	1739.8381	1739.8375	0.36	0	46	0.00039	1	U	R.MLDGLDPHPMLPNYK.N
8039	900 - 914	586.2842	1755.8308	1755.8324	-0.89	0	35	0.016	1	U	R.MLDGLDPHPMLPNYK.N + Oxidation (M)
8040	900 - 914	878.9231	1755.8316	1755.8324	-0.43	0	36	0.0013	1	U	R.MLDGLDPHPMLPNYK.N + Oxidation (M)
8041	900 - 914	878.9232	1755.8319	1755.8324	-0.29	0	28	0.014	1	U	R.MLDGLDPHPMLPNYK.N + Oxidation (M)
8043	900 - 914	878.9237	1755.8329	1755.8324	0.30	0	59	3e-05	1	U	R.MLDGLDPHPMLPNYK.N + Oxidation (M)
8167	900 - 914	886.9202	1771.8259	1771.8273	-0.82	0	27	0.0049	1	U	R.MLDGLDPHPMLPNYK.N + 2 Oxidation (M)
8169	900 - 914	886.9207	1771.8268	1771.8273	-0.28	0	31	0.0022	1	U	R.MLDGLDPHPMLPNYK.N + 2 Oxidation (M)
8172	900 - 914	886.9212	1771.8278	1771.8273	0.28	0	27	0.012	1	U	R.MLDGLDPHPMLPNYK.N + 2 Oxidation (M)
8174	900 - 914	886.9213	1771.8280	1771.8273	0.40	0	26	0.0065	1	U	R.MLDGLDPHPMLPNYK.N + 2 Oxidation (M)
8176	900 - 914	886.9216	1771.8286	1771.8273	0.74	0	30	0.0035	1	U	R.MLDGLDPHPMLPNYK.N + 2 Oxidation (M)
11939	915 - 939	938.1473	2811.4200	2811.4192	0.30	1	45	0.00013	1	U	K.NFKGTIQELGQNQYAVSGEIFVVDR.N
10938	918 - 939	1212.1120	2422.2094	2422.2129	-1.41	0	129	1.5e-12	1	U	K.GTIQELGQNQYAVSGEIFVVDR.N
10944	918 - 939	1212.1133	2422.2121	2422.2129	-0.31	0	18	0.029	1	U	K.GTIQELGQNQYAVSGEIFVVDR.N
10946	918 - 939	808.4114	2422.2124	2422.2129	-0.20	0	24	0.036	1	U	K.GTIQELGQNQYAVSGEIFVVDR.N
10949	918 - 939	808.4116	2422.2129	2422.2129	0.034	0	21	0.032	1	U	K.GTIQELGQNQYAVSGEIFVVDR.N
10950	918 - 939	808.4117	2422.2132	2422.2129	0.16	0	33	0.0026	1	U	K.GTIQELGQNQYAVSGEIFVVDR.N
10952	918 - 939	808.4117	2422.2133	2422.2129	0.19	0	31	0.005	1	U	K.GTIQELGQNQYAVSGEIFVVDR.N
10955	918 - 939	606.5608	2422.2139	2422.2129	0.44	0	33	0.0072	1	U	K.GTIQELGQNQYAVSGEIFVVDR.N
10958	918 - 939	1212.1144	2422.2143	2422.2129	0.58	0	68	6.8e-07	1	U	K.GTIQELGQNQYAVSGEIFVVDR.N
10961	918 - 939	808.4121	2422.2144	2422.2129	0.64	0	28	0.031	1	U	K.GTIQELGQNQYAVSGEIFVVDR.N
10963	918 - 939	1212.1148	2422.2150	2422.2129	0.88	0	64	8.3e-06	1	U	K.GTIQELGQNQYAVSGEIFVVDR.N
10965	918 - 939	808.4124	2422.2153	2422.2129	1.01	0	21	0.032	1	U	K.GTIQELGQNQYAVSGEIFVVDR.N
13593	918 - 950	1225.6388	3673.8947	3673.8952	-0.12	1	87	1.8e-07	1	U	K.GTIQELGQNQYAVSGEIFVVDRNTVEITELPVR.T
3798	940 - 950	635.8534	1269.6923	1269.6929	-0.43	0	27	0.041	1	U	R.NTVEITELPVR.T
3800	940 - 950	635.8535	1269.6925	1269.6929	-0.26	0	26	0.016	1	U	R.NTVEITELPVR.T
3801	940 - 950	635.8536	1269.6926	1269.6929	-0.23	0	52	0.00018	1	U	R.NTVEITELPVR.T
3803	940 - 950	424.2382	1269.6928	1269.6929	-0.043	0	57	0.00011	1	U	R.NTVEITELPVR.T
3804	940 - 950	635.8538	1269.6930	1269.6929	0.088	0	67	9.4e-06	1	U	R.NTVEITELPVR.T
3805	940 - 950	635.8538	1269.6930	1269.6929	0.14	0	25	0.023	1	U	R.NTVEITELPVR.T
3806	940 - 950	635.8538	1269.6931	1269.6929	0.18	0	32	0.0015	1	U	R.NTVEITELPVR.T
3807	940 - 950	635.8539	1269.6932	1269.6929	0.26	0	67	8.1e-06	1	U	R.NTVEITELPVR.T
1020	951 - 957	463.2422	924.4698	924.4705	-0.77	0	32	0.034	1	U	R.TWTQVYK.E
5408	958 - 970	737.3664	1472.7182	1472.7181	0.088	0	37	0.004	1	U	K.EQVLEPMLNGTDK.T
5528	958 - 970	745.3636	1488.7127	1488.7130	-0.18	0	52	0.00011	1	U	K.EQVLEPMLNGTDK.T + Oxidation (M)
11171	958 - 979	826.7533	2477.2380	2477.2359	0.83	1	42	0.00079	1	U	K.EQVLEPMLNGTDKTPALISDYK.E + Oxidation (M)
1561	971 - 979	504.2737	1006.5328	1006.5335	-0.66	0	30	0.0089	1	U	K.TPALISDYK.E
1562	971 - 979	504.2740	1006.5334	1006.5335	-0.084	0	33	0.0064	1	U	K.TPALISDYK.E
1563	971 - 979	504.2740	1006.5334	1006.5335	-0.084	0	31	0.031	1	U	K.TPALISDYK.E
1564	971 - 979	504.2740	1006.5334	1006.5335	-0.045	0	36	0.003	1	U	K.TPALISDYK.E
9933	971 - 988	694.6837	2081.0292	2081.0317	-1.18	1	37	0.00069	1	U	K.TPALISDYKEYHTDTTVK.F

Query	Start - End	Observed	Mr(expt)	Mr(calc)	ppm	M	Score	Expect	Rank	U	Peptide
9934	971 - 988	694.6841	2081.0305	2081.0317	-0.56	1	26	0.0043	1	U	K.TPALISDYKEYHTDTTVK.F
9939	971 - 988	694.6847	2081.0323	2081.0317	0.28	1	23	0.014	1	U	K.TPALISDYKEYHTDTTVK.F
9940	971 - 988	694.6848	2081.0325	2081.0317	0.41	1	30	0.0085	1	U	K.TPALISDYKEYHTDTTVK.F
2291	980 - 988	547.2616	1092.5087	1092.5088	-0.010	0	22	0.021	1		K.EYHTDTTVK.F
7764	993 - 1008	576.2981	1725.8726	1725.8719	0.37	1	30	0.0028	1	U	K.MTEEKLAQAEAAGLHK.V
2413	998 - 1008	554.8088	1107.6029	1107.6036	-0.61	0	57	5.7e-05	1	U	K.LAQAEAAGLHK.V
2415	998 - 1008	554.8089	1107.6033	1107.6036	-0.27	0	29	0.0099	1	U	K.LAQAEAAGLHK.V
2416	998 - 1008	370.2084	1107.6034	1107.6036	-0.23	0	44	0.00065	1	U	K.LAQAEAAGLHK.V
2417	998 - 1008	370.2085	1107.6036	1107.6036	0.018	0	42	0.0014	1	U	K.LAQAEAAGLHK.V
2418	998 - 1008	554.8091	1107.6036	1107.6036	0.023	0	55	7.2e-05	1	U	K.LAQAEAAGLHK.V
2419	998 - 1008	370.2086	1107.6039	1107.6036	0.21	0	44	0.0011	1	U	K.LAQAEAAGLHK.V
2420	998 - 1008	554.8095	1107.6044	1107.6036	0.67	0	25	0.013	1	U	K.LAQAEAAGLHK.V
3486	1032 - 1041	618.8453	1235.6761	1235.6761	-0.052	1	58	2.4e-05	1	U	K.KYETVQDILK.E
3487	1032 - 1041	618.8453	1235.6761	1235.6761	-0.0032	1	41	0.00089	1	U	K.KYETVQDILK.E
3491	1032 - 1041	618.8454	1235.6762	1235.6761	0.078	1	31	0.015	1	U	K.KYETVQDILK.E
3492	1032 - 1041	618.8454	1235.6762	1235.6761	0.078	1	38	0.0011	1	U	K.KYETVQDILK.E
3493	1032 - 1041	412.8994	1235.6765	1235.6761	0.27	1	37	0.0084	1	U	K.KYETVQDILK.E
3494	1032 - 1041	618.8456	1235.6766	1235.6761	0.42	1	46	0.00019	1	U	K.KYETVQDILK.E
2412	1033 - 1041	554.7983	1107.5820	1107.5812	0.71	0	35	0.018	1	U	K.YETVQDILK.E
742	1048 - 1054	436.2373	870.4600	870.4599	0.067	0	38	0.0047	1	U	R.LSYYGLR.K
6248	1055 - 1068	516.9410	1547.8010	1547.8017	-0.47	1	23	0.013	1	U	R.KEWLVGMLGAESTK.L
6249	1055 - 1068	516.9412	1547.8018	1547.8017	0.058	1	30	0.0092	1	U	R.KEWLVGMLGAESTK.L
6250	1055 - 1068	774.9082	1547.8019	1547.8017	0.13	1	76	1.9e-06	1	U	R.KEWLVGMLGAESTK.L
6251	1055 - 1068	516.9414	1547.8024	1547.8017	0.43	1	25	0.04	1	U	R.KEWLVGMLGAESTK.L
6410	1055 - 1068	522.2723	1563.7950	1563.7967	-1.04	1	43	0.00048	1	U	R.KEWLVGMLGAESTK.L + Oxidation (M)
6411	1055 - 1068	782.9050	1563.7954	1563.7967	-0.79	1	54	5.5e-05	1	U	R.KEWLVGMLGAESTK.L + Oxidation (M)
6412	1055 - 1068	522.2726	1563.7959	1563.7967	-0.52	1	21	0.048	1	U	R.KEWLVGMLGAESTK.L + Oxidation (M)
6413	1055 - 1068	782.9052	1563.7959	1563.7967	-0.46	1	64	6.2e-06	1	U	R.KEWLVGMLGAESTK.L + Oxidation (M)
6414	1055 - 1068	522.2726	1563.7960	1563.7967	-0.40	1	46	0.00014	1	U	R.KEWLVGMLGAESTK.L + Oxidation (M)
6415	1055 - 1068	782.9055	1563.7965	1563.7967	-0.12	1	69	5.6e-06	1	U	R.KEWLVGMLGAESTK.L + Oxidation (M)
6416	1055 - 1068	782.9057	1563.7968	1563.7967	0.068	1	49	0.00015	1	U	R.KEWLVGMLGAESTK.L + Oxidation (M)
6417	1055 - 1068	522.2729	1563.7970	1563.7967	0.19	1	36	0.00054	1	U	R.KEWLVGMLGAESTK.L + Oxidation (M)
6418	1055 - 1068	782.9059	1563.7972	1563.7967	0.32	1	67	1.6e-05	1	U	R.KEWLVGMLGAESTK.L + Oxidation (M)
6419	1055 - 1068	522.2732	1563.7978	1563.7967	0.71	1	36	0.0005	1	U	R.KEWLVGMLGAESTK.L + Oxidation (M)
6420	1055 - 1068	522.2739	1563.7998	1563.7967	2.00	1	45	0.00025	1	U	R.KEWLVGMLGAESTK.L + Oxidation (M)
4914	1056 - 1068	710.8602	1419.7059	1419.7068	-0.62	0	15	0.044	1	U	K.EWLVGMLGAESTK.L
4915	1056 - 1068	710.8606	1419.7066	1419.7068	-0.15	0	21	0.031	1	U	K.EWLVGMLGAESTK.L
4916	1056 - 1068	710.8608	1419.7069	1419.7068	0.11	0	46	5.8e-05	1	U	K.EWLVGMLGAESTK.L
4917	1056 - 1068	474.2430	1419.7072	1419.7068	0.29	0	25	0.013	1	U	K.EWLVGMLGAESTK.L
4919	1056 - 1068	710.8610	1419.7075	1419.7068	0.49	0	17	0.034	1	U	K.EWLVGMLGAESTK.L
4920	1056 - 1068	710.8611	1419.7076	1419.7068	0.59	0	36	0.00043	1	U	K.EWLVGMLGAESTK.L
5058	1056 - 1068	718.8572	1435.6999	1435.7017	-1.25	0	38	0.0045	1	U	K.EWLVGMLGAESTK.L + Oxidation (M)
5059	1056 - 1068	718.8574	1435.7003	1435.7017	-0.99	0	36	0.0039	1	U	K.EWLVGMLGAESTK.L + Oxidation (M)
5060	1056 - 1068	718.8578	1435.7011	1435.7017	-0.39	0	35	0.0019	1	U	K.EWLVGMLGAESTK.L + Oxidation (M)
5061	1056 - 1068	718.8580	1435.7014	1435.7017	-0.24	0	37	0.018	1	U	K.EWLVGMLGAESTK.L + Oxidation (M)
5062	1056 - 1068	718.8581	1435.7017	1435.7017	0.0014	0	34	0.015	1	U	K.EWLVGMLGAESTK.L + Oxidation (M)
2120	1075 - 1083	538.3287	1074.6429	1074.6437	-0.78	1	32	0.0028	1	U	R.FILEKIQGK.I
3561	1092 - 1101	415.2443	1242.7111	1242.7118	-0.54	1	39	0.0056	1	U	K.KDLIQMLVQR.G
3562	1092 - 1101	622.3629	1242.7113	1242.7118	-0.43	1	61	3.3e-05	1	U	K.KDLIQMLVQR.G
3700	1092 - 1101	630.3608	1258.7070	1258.7067	0.21	1	51	0.00031	1	U	K.KDLIQMLVQR.G + Oxidation (M)
3701	1092 - 1101	630.3609	1258.7072	1258.7067	0.41	1	52	0.0004	1	U	K.KDLIQMLVQR.G + Oxidation (M)
3702	1092 - 1101	420.5766	1258.7081	1258.7067	1.11	1	38	0.0077	1	U	K.KDLIQMLVQR.G + Oxidation (M)
2484	1093 - 1101	558.3153	1114.6161	1114.6169	-0.69	0	58	9e-05	1	U	K.DLIQMLVQR.G
2485	1093 - 1101	558.3157	1114.6168	1114.6169	-0.039	0	41	0.004	1	U	K.DLIQMLVQR.G
2486	1093 - 1101	558.3157	1114.6169	1114.6169	0.014	0	51	0.00041	1	U	K.DLIQMLVQR.G
2487	1093 - 1101	558.3158	1114.6170	1114.6169	0.10	0	27	0.029	1	U	K.DLIQMLVQR.G
2488	1093 - 1101	558.3158	1114.6170	1114.6169	0.18	0	58	9.7e-05	1	U	K.DLIQMLVQR.G
2489	1093 - 1101	558.3159	1114.6172	1114.6169	0.30	0	54	0.00021	1	U	K.DLIQMLVQR.G
2490	1093 - 1101	558.3162	1114.6179	1114.6169	0.97	0	32	0.01	1	U	K.DLIQMLVQR.G
2491	1093 - 1101	558.3163	1114.6180	1114.6169	1.00	0	31	0.027	1	U	K.DLIQMLVQR.G
2650	1093 - 1101	566.3123	1130.6100	1130.6118	-1.58	0	44	0.00086	1	U	K.DLIQMLVQR.G + Oxidation (M)
2651	1093 - 1101	566.3127	1130.6108	1130.6118	-0.85	0	45	0.0022	1	U	K.DLIQMLVQR.G + Oxidation (M)
2652	1093 - 1101	566.3129	1130.6112	1130.6118	-0.50	0	54	0.00022	1	U	K.DLIQMLVQR.G + Oxidation (M)
2653	1093 - 1101	566.3131	1130.6117	1130.6118	-0.038	0	48	0.0011	1	U	K.DLIQMLVQR.G + Oxidation (M)
2654	1093 - 1101	566.3132	1130.6118	1130.6118	0.015	0	61	5.9e-05	1	U	K.DLIQMLVQR.G + Oxidation (M)
2655	1093 - 1101	566.3132	1130.6119	1130.6118	0.12	0	49	0.00052	1	U	K.DLIQMLVQR.G + Oxidation (M)
2656	1093 - 1101	566.3133	1130.6121	1130.6118	0.30	0	33	0.031	1	U	K.DLIQMLVQR.G + Oxidation (M)
2657	1093 - 1101	566.3134	1130.6122	1130.6118	0.35	0	39	0.0033	1	U	K.DLIQMLVQR.G + Oxidation (M)

Query	Start – End	Observed	Mr(expt)	Mr(calc)	ppm	M	Score	Expect	Rank	U	Peptide
2658	1093 – 1101	566.3134	1130.6122	1130.6118	0.37	0	49	0.00083	1	U	K.DLIQMLVQR.G + Oxidation (M)
2659	1093 – 1101	566.3134	1130.6123	1130.6118	0.44	0	27	0.042	1	U	K.DLIQMLVQR.G + Oxidation (M)
836	1102 – 1109	447.7136	893.4126	893.4131	-0.48	0	18	0.048	1	U	R.GYESDPVK.A
837	1102 – 1109	447.7137	893.4129	893.4131	-0.21	0	27	0.028	1	U	R.GYESDPVK.A
838	1102 – 1109	447.7138	893.4129	893.4131	-0.12	0	42	0.00023	1	U	R.GYESDPVK.A
1428	1157 – 1164	494.2896	986.5647	986.5648	-0.11	1	36	0.013	1	U	K.EKVEELIK.Q
1431	1157 – 1164	494.2899	986.5652	986.5648	0.48	1	46	0.0011	1	U	K.EKVEELIK.Q
2228	1170 – 1178	543.8044	1085.5942	1085.5941	0.053	2	42	0.0034	1	U	K.GREVNDLKR.K
2229	1170 – 1178	362.8721	1085.5946	1085.5941	0.42	2	41	0.005	1	U	K.GREVNDLKR.K
754	1172 – 1178	437.2428	872.4711	872.4716	-0.47	1	42	0.0037	1	U	R.EVNDLKR.K
755	1172 – 1178	437.2431	872.4717	872.4716	0.15	1	42	0.0039	1	U	R.EVNDLKR.K
1253	1179 – 1186	480.7617	959.5088	959.5076	1.20	1	25	0.042	1		R.KSPSDLWK.E
532	1180 – 1186	416.7137	831.4129	831.4127	0.25	0	26	0.039	1		K.SPSDLWK.E
4654	1187 – 1198	689.8402	1377.6658	1377.6664	-0.37	0	50	0.00059	1	U	K.EDLAAFVEELDK.V
4655	1187 – 1198	689.8402	1377.6659	1377.6664	-0.31	0	35	0.0026	1	U	K.EDLAAFVEELDK.V
4656	1187 – 1198	689.8405	1377.6664	1377.6664	0.070	0	36	0.0013	1	U	K.EDLAAFVEELDK.V
4658	1187 – 1198	689.8407	1377.6669	1377.6664	0.39	0	64	8.7e-06	1	U	K.EDLAAFVEELDK.V
10054	1187 – 1204	1054.0107	2106.0068	2106.0117	-2.29	1	80	3.2e-07	1	U	K.EDLAAFVEELDKVESQER.E
10055	1187 – 1204	703.0103	2106.0090	2106.0117	-1.26	1	42	0.0015	1	U	K.EDLAAFVEELDKVESQER.E
10056	1187 – 1204	1054.0121	2106.0097	2106.0117	-0.92	1	36	0.0014	1	U	K.EDLAAFVEELDKVESQER.E
10057	1187 – 1204	703.0108	2106.0105	2106.0117	-0.53	1	32	0.0089	1	U	K.EDLAAFVEELDKVESQER.E
10058	1187 – 1204	703.0110	2106.0113	2106.0117	-0.19	1	39	0.0064	1	U	K.EDLAAFVEELDKVESQER.E
10059	1187 – 1204	703.0110	2106.0113	2106.0117	-0.17	1	28	0.01	1	U	K.EDLAAFVEELDKVESQER.E
10060	1187 – 1204	703.0112	2106.0118	2106.0117	0.054	1	44	0.0014	1	U	K.EDLAAFVEELDKVESQER.E
10061	1187 – 1204	703.0112	2106.0119	2106.0117	0.097	1	34	0.003	1	U	K.EDLAAFVEELDKVESQER.E
10062	1187 – 1204	1054.0132	2106.0119	2106.0117	0.11	1	68	1.8e-06	1	U	K.EDLAAFVEELDKVESQER.E
10063	1187 – 1204	703.0112	2106.0119	2106.0117	0.11	1	43	0.0025	1	U	K.EDLAAFVEELDKVESQER.E
10064	1187 – 1204	703.0112	2106.0119	2106.0117	0.11	1	37	0.0099	1	U	K.EDLAAFVEELDKVESQER.E
10065	1187 – 1204	703.0112	2106.0119	2106.0117	0.13	1	42	0.0016	1	U	K.EDLAAFVEELDKVESQER.E
10066	1187 – 1204	703.0113	2106.0120	2106.0117	0.14	1	38	0.0046	1	U	K.EDLAAFVEELDKVESQER.E
10067	1187 – 1204	703.0113	2106.0120	2106.0117	0.18	1	38	0.0014	1	U	K.EDLAAFVEELDKVESQER.E
10068	1187 – 1204	1054.0133	2106.0121	2106.0117	0.20	1	79	1.9e-07	1	U	K.EDLAAFVEELDKVESQER.E
10069	1187 – 1204	703.0113	2106.0121	2106.0117	0.23	1	45	0.00028	1	U	K.EDLAAFVEELDKVESQER.E
10070	1187 – 1204	703.0114	2106.0124	2106.0117	0.37	1	30	0.031	1	U	K.EDLAAFVEELDKVESQER.E
10071	1187 – 1204	703.0115	2106.0126	2106.0117	0.44	1	35	0.0047	1	U	K.EDLAAFVEELDKVESQER.E
10072	1187 – 1204	1054.0137	2106.0128	2106.0117	0.53	1	62	9.3e-06	1	U	K.EDLAAFVEELDKVESQER.E
10073	1187 – 1204	1054.0137	2106.0128	2106.0117	0.54	1	64	3.1e-06	1	U	K.EDLAAFVEELDKVESQER.E
10074	1187 – 1204	1054.0138	2106.0130	2106.0117	0.63	1	53	7.3e-05	1	U	K.EDLAAFVEELDKVESQER.E
10075	1187 – 1204	703.0117	2106.0132	2106.0117	0.74	1	33	0.0055	1	U	K.EDLAAFVEELDKVESQER.E
10076	1187 – 1204	1054.0145	2106.0145	2106.0117	1.33	1	48	8.9e-05	1	U	K.EDLAAFVEELDKVESQER.E
10077	1187 – 1204	1054.0146	2106.0147	2106.0117	1.46	1	60	2.2e-05	1	U	K.EDLAAFVEELDKVESQER.E
10078	1187 – 1204	1054.0148	2106.0150	2106.0117	1.61	1	50	9.2e-05	1	U	K.EDLAAFVEELDKVESQER.E
10079	1187 – 1204	1054.0151	2106.0156	2106.0117	1.86	1	61	8.7e-06	1	U	K.EDLAAFVEELDKVESQER.E
10080	1187 – 1204	1054.0155	2106.0164	2106.0117	2.25	1	50	7e-05	1	U	K.EDLAAFVEELDKVESQER.E
12593	1187 – 1214	1032.1676	3093.4809	3093.4812	-0.089	2	65	1.5e-05	1	U	K.EDLAAFVEELDKVESQEREDVLAGMSGK.A
12595	1187 – 1214	1032.1683	3093.4831	3093.4812	0.62	2	46	0.0012	1	U	K.EDLAAFVEELDKVESQEREDVLAGMSGK.A
12635	1187 – 1214	1037.4983	3109.4730	3109.4761	-0.99	2	47	0.00062	1	U	K.EDLAAFVEELDKVESQEREDVLAGMSGK.A + Oxidation (M)
12636	1187 – 1214	1037.4988	3109.4746	3109.4761	-0.48	2	43	0.0016	1	U	K.EDLAAFVEELDKVESQEREDVLAGMSGK.A + Oxidation (M)
12637	1187 – 1214	1037.4989	3109.4748	3109.4761	-0.42	2	38	0.0013	1	U	K.EDLAAFVEELDKVESQEREDVLAGMSGK.A + Oxidation (M)
12640	1187 – 1214	1037.4994	3109.4763	3109.4761	0.067	2	56	3.7e-05	1	U	K.EDLAAFVEELDKVESQEREDVLAGMSGK.A + Oxidation (M)
12644	1187 – 1214	1037.5003	3109.4790	3109.4761	0.94	2	44	0.00084	1	U	K.EDLAAFVEELDKVESQEREDVLAGMSGK.A + Oxidation (M)
7846	1199 – 1214	867.9198	1733.8251	1733.8254	-0.14	1	59	1.9e-05	1	U	K.VESQEREDVLAGMSGK.A
7847	1199 – 1214	578.9490	1733.8253	1733.8254	-0.042	1	36	0.015	1	U	K.VESQEREDVLAGMSGK.A
7991	1199 – 1214	875.9174	1749.8202	1749.8203	-0.037	1	75	4.8e-07	1	U	K.VESQEREDVLAGMSGK.A + Oxidation (M)
7992	1199 – 1214	584.2808	1749.8206	1749.8203	0.17	1	33	0.0026	1	U	K.VESQEREDVLAGMSGK.A + Oxidation (M)
7994	1199 – 1214	875.9178	1749.8210	1749.8203	0.37	1	63	1.7e-05	1	U	K.VESQEREDVLAGMSGK.A + Oxidation (M)
1552	1205 – 1214	503.7474	1005.4803	1005.4801	0.20	0	60	1.6e-05	1	U	R.EDVLAGMSGK.A
1668	1205 – 1214	511.7444	1021.4742	1021.4750	-0.74	0	54	9.6e-05	1	U	R.EDVLAGMSGK.A + Oxidation (M)
1669	1205 – 1214	511.7445	1021.4745	1021.4750	-0.51	0	51	0.00012	1	U	R.EDVLAGMSGK.A + Oxidation (M)
1670	1205 – 1214	511.7448	1021.4750	1021.4750	0.0020	0	47	0.0004	1	U	R.EDVLAGMSGK.A + Oxidation (M)
1671	1205 – 1214	511.7448	1021.4751	1021.4750	0.061	0	50	0.00021	1	U	R.EDVLAGMSGK.A + Oxidation (M)
1672	1205 – 1214	511.7448	1021.4751	1021.4750	0.12	0	60	2.6e-05	1	U	R.EDVLAGMSGK.A + Oxidation (M)
1673	1205 – 1214	511.7449	1021.4753	1021.4750	0.32	0	58	3.6e-05	1	U	R.EDVLAGMSGK.A + Oxidation (M)
1674	1205 – 1214	511.7451	1021.4756	1021.4750	0.55	0	47	0.00041	1	U	R.EDVLAGMSGK.A + Oxidation (M)
7110	1227 – 1240	824.9215	1647.8284	1647.8290	-0.38	1	88	3.4e-08	1	U	K.KLQLEETMPSPYGR.R
7207	1227 – 1240	832.9187	1663.8229	1663.8239	-0.61	1	76	1.7e-07	1	U	K.KLQLEETMPSPYGR.R + Oxidation (M)
7208	1227 – 1240	832.9190	1663.8234	1663.8239	-0.30	1	60	5.1e-06	1	U	K.KLQLEETMPSPYGR.R + Oxidation (M)
7209	1227 – 1240	555.6151	1663.8236	1663.8239	-0.22	1	31	0.004	1	U	K.KLQLEETMPSPYGR.R + Oxidation (M)
7210	1227 – 1240	555.6152	1663.8236	1663.8239	-0.18	1	31	0.0057	1	U	K.KLQLEETMPSPYGR.R + Oxidation (M)
7211	1227 – 1240	555.6152	1663.8237	1663.8239	-0.11	1	17	0.036	1	U	K.KLQLEETMPSPYGR.R + Oxidation (M)
7212	1227 – 1240	832.9192	1663.8238	1663.8239	-0.073	1	47	0.00012	1	U	K.KLQLEETMPSPYGR.R + Oxidation (M)
7213	1227 – 1240	832.9192	1663.8239	1663.8239	-0.037	1	76	2.2e-07	1	U	K.KLQLEETMPSPYGR.R

Query	Start – End	Observed	Mr(expt)	Mr(calc)	ppm	M	Score	Expect	Rank	U	Peptide
											+ Oxidation (M)
7214	1227 – 1240	555.6152	1663.8239	1663.8239	-0.022	1	32	0.0029	1	U	K.KLQLEETMPSPYGR.R
											+ Oxidation (M)
7215	1227 – 1240	832.9192	1663.8239	1663.8239	-0.00060	1	46	0.00015	1	U	K.KLQLEETMPSPYGR.R
											+ Oxidation (M)
7216	1227 – 1240	832.9193	1663.8241	1663.8239	0.084	1	76	2.7e-07	1	U	K.KLQLEETMPSPYGR.R
											+ Oxidation (M)
7217	1227 – 1240	555.6154	1663.8243	1663.8239	0.23	1	28	0.0071	1	U	K.KLQLEETMPSPYGR.R
											+ Oxidation (M)
7218	1227 – 1240	832.9199	1663.8253	1663.8239	0.84	1	76	1.9e-07	1	U	K.KLQLEETMPSPYGR.R
											+ Oxidation (M)
7219	1227 – 1240	832.9205	1663.8264	1663.8239	1.47	1	71	4.6e-07	1	U	K.KLQLEETMPSPYGR.R
											+ Oxidation (M)
5883	1228 – 1240	760.8738	1519.7330	1519.7341	-0.66	0	60	1e-05	1	U	K.LQLEETMPSPYGR.R
5885	1228 – 1240	760.8743	1519.7340	1519.7341	-0.044	0	68	1.3e-06	1	U	K.LQLEETMPSPYGR.R
6104	1228 – 1240	768.8713	1535.7281	1535.7290	-0.55	0	66	1.4e-06	1	U	K.LQLEETMPSPYGR.R
											+ Oxidation (M)
6105	1228 – 1240	768.8716	1535.7287	1535.7290	-0.15	0	67	1.2e-06	1	U	K.LQLEETMPSPYGR.R
											+ Oxidation (M)
6106	1228 – 1240	768.8717	1535.7289	1535.7290	-0.068	0	58	2e-05	1	U	K.LQLEETMPSPYGR.R
											+ Oxidation (M)
6107	1228 – 1240	768.8719	1535.7292	1535.7290	0.18	0	61	1.1e-05	1	U	K.LQLEETMPSPYGR.R
											+ Oxidation (M)
6108	1228 – 1240	768.8720	1535.7294	1535.7290	0.27	0	62	6.5e-06	1	U	K.LQLEETMPSPYGR.R
											+ Oxidation (M)
3021	1241 – 1250	586.3470	1170.6794	1170.6794	-0.051	1	34	0.0023	1	U	R.RIIPEITAMK.A
3022	1241 – 1250	391.2337	1170.6794	1170.6794	-0.031	1	36	0.0017	1	U	R.RIIPEITAMK.A
3023	1241 – 1250	586.3471	1170.6796	1170.6794	0.19	1	41	0.00098	1	U	R.RIIPEITAMK.A
3131	1241 – 1250	594.3438	1186.6731	1186.6743	-1.06	1	26	0.044	1	U	R.RIIPEITAMK.A
											+ Oxidation (M)
3132	1241 – 1250	396.5652	1186.6738	1186.6743	-0.46	1	26	0.008	1	U	R.RIIPEITAMK.A
											+ Oxidation (M)
3133	1241 – 1250	594.3443	1186.6740	1186.6743	-0.32	1	37	0.0022	1	U	R.RIIPEITAMK.A
											+ Oxidation (M)
3134	1241 – 1250	594.3445	1186.6745	1186.6743	0.10	1	31	0.007	1	U	R.RIIPEITAMK.A
											+ Oxidation (M)
3135	1241 – 1250	396.5654	1186.6745	1186.6743	0.15	1	16	0.042	1	U	R.RIIPEITAMK.A
											+ Oxidation (M)
3136	1241 – 1250	396.5655	1186.6746	1186.6743	0.25	1	25	0.027	1	U	R.RIIPEITAMK.A
											+ Oxidation (M)
3137	1241 – 1250	594.3446	1186.6747	1186.6743	0.34	1	36	0.00075	1	U	R.RIIPEITAMK.A
											+ Oxidation (M)
3138	1241 – 1250	594.3448	1186.6750	1186.6743	0.56	1	49	5.5e-05	1	U	R.RIIPEITAMK.A
											+ Oxidation (M)
1622	1242 – 1250	508.2967	1014.5788	1014.5783	0.50	0	58	6.9e-05	1	U	R.IIPEITAMK.A
2786	1261 – 1271	573.3293	1144.6440	1144.6452	-0.98	2	57	5.2e-05	1	U	K.KKGDLDTAAVK.V
2788	1261 – 1271	382.5558	1144.6455	1144.6452	0.32	2	30	0.022	1	U	K.KKGDLDTAAVK.V
1640	1262 – 1271	509.2822	1016.5499	1016.5502	-0.30	1	36	0.015	1	U	K.KGDLDTAAVK.V
1641	1262 – 1271	509.2824	1016.5503	1016.5502	0.11	1	65	1.7e-05	1	U	K.KGDLDTAAVK.V
1642	1262 – 1271	509.2825	1016.5504	1016.5502	0.15	1	43	0.0031	1	U	K.KGDLDTAAVK.V
1643	1262 – 1271	509.2825	1016.5504	1016.5502	0.23	1	42	0.0034	1	U	K.KGDLDTAAVK.V
824	1263 – 1271	445.2350	888.4554	888.4553	0.14	0	34	0.022	1	U	K.GDLDTAAVK.V
13694	1263 – 1299	947.9651	3787.8312	3787.8316	-0.10	1	50	0.00019	1	U	K.GDLDTAAVKVEFDEEFSGAPVEGAGEEALTPSVPINK.G
12101	1272 – 1299	973.4688	2917.3844	2917.3869	-0.85	0	42	0.00079	1	U	K.VEFDEEFSGAPVEGAGEEALTPSVPINK.G
12104	1272 – 1299	973.4692	2917.3858	2917.3869	-0.37	0	33	0.0028	1	U	K.VEFDEEFSGAPVEGAGEEALTPSVPINK.G
12108	1272 – 1299	973.4695	2917.3866	2917.3869	-0.099	0	49	0.0014	1	U	K.VEFDEEFSGAPVEGAGEEALTPSVPINK.G
12109	1272 – 1299	973.4696	2917.3869	2917.3869	0.0034	0	25	0.012	1	U	K.VEFDEEFSGAPVEGAGEEALTPSVPINK.G
12111	1272 – 1299	973.4698	2917.3875	2917.3869	0.21	0	34	0.0035	1	U	K.VEFDEEFSGAPVEGAGEEALTPSVPINK.G
12112	1272 – 1299	730.3542	2917.3875	2917.3869	0.22	0	52	0.00027	1	U	K.VEFDEEFSGAPVEGAGEEALTPSVPINK.G
12113	1272 – 1299	973.4699	2917.3878	2917.3869	0.30	0	38	0.0045	1	U	K.VEFDEEFSGAPVEGAGEEALTPSVPINK.G
12117	1272 – 1299	973.4702	2917.3889	2917.3869	0.67	0	51	0.00015	1	U	K.VEFDEEFSGAPVEGAGEEALTPSVPINK.G
12119	1272 – 1299	973.4703	2917.3892	2917.3869	0.77	0	28	0.014	1	U	K.VEFDEEFSGAPVEGAGEEALTPSVPINK.G
12123	1272 – 1299	973.4713	2917.3920	2917.3869	1.73	0	25	0.016	1	U	K.VEFDEEFSGAPVEGAGEEALTPSVPINK.G
12124	1272 – 1299	973.4717	2917.3932	2917.3869	2.14	0	33	0.0038	1	U	K.VEFDEEFSGAPVEGAGEEALTPSVPINK.G
5935	1314 – 1327	508.6026	1522.7859	1522.7868	-0.61	2	23	0.022	1	U	R.VRKTPTSSGKPSAK.K
											+ Phospho (ST)
3150	1316 – 1327	594.8326	1187.6506	1187.6510	-0.34	1	42	0.0017	1	U	R.KTPTSSGKPSAK.K
3151	1316 – 1327	396.8908	1187.6506	1187.6510	-0.30	1	27	0.04	1	U	R.KTPTSSGKPSAK.K
3156	1316 – 1327	396.8910	1187.6511	1187.6510	0.13	1	26	0.027	1	U	R.KTPTSSGKPSAK.K
3157	1316 – 1327	594.8329	1187.6512	1187.6510	0.20	1	53	0.00032	1	U	R.KTPTSSGKPSAK.K
3158	1316 – 1327	594.8330	1187.6514	1187.6510	0.32	1	53	6.2e-05	1	U	R.KTPTSSGKPSAK.K
1982	1317 – 1327	354.1925	1059.5556	1059.5560	-0.39	0	26	0.0098	1	U	K.TPTSSGKPSAK.K
1983	1317 – 1327	530.7851	1059.5557	1059.5560	-0.34	0	41	0.00073	1	U	K.TPTSSGKPSAK.K
1984	1317 – 1327	530.7851	1059.5557	1059.5560	-0.32	0	32	0.003	1	U	K.TPTSSGKPSAK.K
1985	1317 – 1327	530.7853	1059.5561	1059.5560	0.074	0	28	0.0063	1	U	K.TPTSSGKPSAK.K
1986	1317 – 1327	354.1927	1059.5562	1059.5560	0.12	0	37	0.0051	1	U	K.TPTSSGKPSAK.K
12094	1332 – 1356	729.3480	2913.3627	2913.3628	-0.020	2	43	0.0011	1	U	K.RNPWSDDESKSESDLEETEPVVIPR.D
12095	1332 – 1356	972.1285	2913.3637	2913.3628	0.32	2	71	1.4e-06	1	U	K.RNPWSDDESKSESDLEETEPVVIPR.D
12096	1332 – 1356	972.1285	2913.3637	2913.3628	0.32	2	74	6.1e-07	1	U	K.RNPWSDDESKSESDLEETEPVVIPR.D
2130	1333 – 1341	539.2279	1076.4413	1076.4411	0.24	0	39	0.00061	1	U	R.NPWSDDESK.S
11809	1333 – 1356	920.0942	2757.2609	2757.2617	-0.30	1	41	0.0015	1	U	R.NPWSDDESKSESDLEETEPVVIPR.D
11810	1333 – 1356	920.0943	2757.2611	2757.2617	-0.20	1	57	0.00027	1	U	R.NPWSDDESKSESDLEETEPVVIPR.D
7540	1342 – 1356	850.4230	1698.8315	1698.8312	0.16	0	63	4.4e-05	1	U	K.SESDLEETEPVVIPR.D
145	1363 – 1369	371.7138	741.4131	741.4133	-0.31	0	33	0.0027	1	U	R.AAAERPK.Y
12930	1370 – 1396	1076.7351	3227.1835	3227.1858	-0.73	0	29	0.0051	1	U	K.YTFDFSEEEDDDADDDDDDNNDLEELK.V
10905	1397 – 1418	805.0310	2412.0713	2412.0734	-0.87	1	35	0.0022	1	U	K.VKASPITNDGEDEFVPSDGLDK.D
											+ Phospho (ST)
10906	1397 – 1418	805.0313	2412.0720	2412.0734	-0.57	1	35	0.013	1	U	K.VKASPITNDGEDEFVPSDGLDK.D
											+ Phospho (ST)
13320	1397 – 1427	860.1351	3436.5112	3436.5236	-3.60	2	30	0.0084	1	U	K.VKASPITNDGEDEFVPSDGLDKDEYTFSPGK.S
											+ Phospho (ST)
13321	1397 – 1427	1146.5135	3436.5188	3436.5236	-1.39	2	46	0.0012	1	U	K.VKASPITNDGEDEFVPSDGLDKDEYTFSPGK.S
											+ Phospho (ST)
13322	1397 – 1427	1146.5139	3436.5198	3436.5236	-1.10	2	78	8e-07	1	U	K.VKASPITNDGEDEFVPSDGLDKDEYTFSPGK.S
											+ Phospho (ST)

Query	Start – End	Observed	Mr(expt)	Mr(calc)	ppm	M	Score	Expect	Rank	U	Peptide
13323	1397 – 1427	860.1379	3436.5224	3436.5236	-0.34	2	58	7.2e-05	1	U	K.VKASPITNDGEDEFVPSDGLDKDEYTFSPGK.S + Phospho (ST)
13324	1397 – 1427	860.1381	3436.5232	3436.5236	-0.11	2	56	0.00023	1	U	K.VKASPITNDGEDEFVPSDGLDKDEYTFSPGK.S + Phospho (ST)
13325	1397 – 1427	1146.5150	3436.5232	3436.5236	-0.095	2	61	3.5e-05	1	U	K.VKASPITNDGEDEFVPSDGLDKDEYTFSPGK.S + Phospho (ST)
13326	1397 – 1427	1146.5157	3436.5252	3436.5236	0.49	2	79	1.3e-06	1	U	K.VKASPITNDGEDEFVPSDGLDKDEYTFSPGK.S + Phospho (ST)
10044	1399 – 1418	1053.4786	2104.9426	2104.9437	-0.50	0	96	1.1e-08	1	U	K.ASPITNDGEDEFVPSDGLDK.D
12703	1399 – 1427	1044.1382	3129.3929	3129.3939	-0.32	1	85	1.6e-07	1	U	K.ASPITNDGEDEFVPSDGLDKDEYTFSPGK.S
12704	1399 – 1427	1044.1389	3129.3949	3129.3939	0.33	1	69	5.1e-06	1	U	K.ASPITNDGEDEFVPSDGLDKDEYTFSPGK.S
12705	1399 – 1427	1044.1393	3129.3960	3129.3939	0.69	1	76	1.2e-06	1	U	K.ASPITNDGEDEFVPSDGLDKDEYTFSPGK.S
12706	1399 – 1427	1044.1393	3129.3962	3129.3939	0.75	1	78	2.8e-07	1	U	K.ASPITNDGEDEFVPSDGLDKDEYTFSPGK.S
12707	1399 – 1427	783.3565	3129.3968	3129.3939	0.93	1	37	0.012	1	U	K.ASPITNDGEDEFVPSDGLDKDEYTFSPGK.S
12897	1399 – 1427	1070.7928	3209.3567	3209.3602	-1.09	1	35	0.009	1	U	K.ASPITNDGEDEFVPSDGLDKDEYTFSPGK.S + Phospho (ST)
12898	1399 – 1427	1070.7932	3209.3578	3209.3602	-0.74	1	79	4.3e-07	1	U	K.ASPITNDGEDEFVPSDGLDKDEYTFSPGK.S + Phospho (ST)
1825	1419 – 1427	522.2374	1042.4603	1042.4607	-0.45	0	35	0.0045	1	U	K.DEYTFSPGK.S
9568	1440 – 1456	990.4787	1978.9428	1978.9425	0.18	1	90	1.7e-08	1	U	K.KSQDFGNLFSFPSYSQK.S
8830	1441 – 1456	926.4302	1850.8459	1850.8475	-0.90	0	18	0.019	1	U	K.SQDFGNLFSFPSYSQK.S
8833	1441 – 1456	926.4304	1850.8463	1850.8475	-0.67	0	55	1.1e-05	1	U	K.SQDFGNLFSFPSYSQK.S
8834	1441 – 1456	926.4306	1850.8466	1850.8475	-0.50	0	93	5.1e-09	1	U	K.SQDFGNLFSFPSYSQK.S
8835	1441 – 1456	926.4307	1850.8469	1850.8475	-0.35	0	42	0.00011	1	U	K.SQDFGNLFSFPSYSQK.S
8836	1441 – 1456	617.9564	1850.8473	1850.8475	-0.15	0	26	0.031	1	U	K.SQDFGNLFSFPSYSQK.S
8837	1441 – 1456	926.4310	1850.8475	1850.8475	-0.025	0	60	5.8e-06	1	U	K.SQDFGNLFSFPSYSQK.S
8838	1441 – 1456	926.4311	1850.8477	1850.8475	0.094	0	79	9.9e-08	1	U	K.SQDFGNLFSFPSYSQK.S
8839	1441 – 1456	617.9565	1850.8478	1850.8475	0.14	0	38	0.0011	1	U	K.SQDFGNLFSFPSYSQK.S
8840	1441 – 1456	617.9566	1850.8478	1850.8475	0.16	0	56	0.00012	1	U	K.SQDFGNLFSFPSYSQK.S
8842	1441 – 1456	926.4313	1850.8481	1850.8475	0.30	0	74	1.2e-07	1	U	K.SQDFGNLFSFPSYSQK.S
8843	1441 – 1456	617.9567	1850.8482	1850.8475	0.37	0	31	0.0042	1	U	K.SQDFGNLFSFPSYSQK.S
8844	1441 – 1456	926.4316	1850.8486	1850.8475	0.57	0	86	2.2e-08	1	U	K.SQDFGNLFSFPSYSQK.S
8845	1441 – 1456	926.4319	1850.8493	1850.8475	0.95	0	78	2.1e-07	1	U	K.SQDFGNLFSFPSYSQK.S
8846	1441 – 1456	926.4319	1850.8493	1850.8475	0.95	0	62	2e-06	1	U	K.SQDFGNLFSFPSYSQK.S
8847	1441 – 1456	926.4320	1850.8495	1850.8475	1.08	0	29	0.015	1	U	K.SQDFGNLFSFPSYSQK.S
8848	1441 – 1456	926.4325	1850.8505	1850.8475	1.59	0	62	1.6e-06	1	U	K.SQDFGNLFSFPSYSQK.S
11863	1457 – 1482	932.4094	2794.2063	2794.2093	-1.09	1	42	0.0034	1	U	K.SEDDSAKFDSNEEDSASVFSPSFGLK.Q
11866	1457 – 1482	932.4099	2794.2078	2794.2093	-0.57	1	56	5.3e-05	1	U	K.SEDDSAKFDSNEEDSASVFSPSFGLK.Q
11868	1457 – 1482	932.4100	2794.2080	2794.2093	-0.47	1	30	0.0077	1	U	K.SEDDSAKFDSNEEDSASVFSPSFGLK.Q
11869	1457 – 1482	932.4102	2794.2088	2794.2093	-0.19	1	52	8.7e-05	1	U	K.SEDDSAKFDSNEEDSASVFSPSFGLK.Q
11870	1457 – 1482	699.5596	2794.2092	2794.2093	-0.057	1	30	0.018	1	U	K.SEDDSAKFDSNEEDSASVFSPSFGLK.Q
11871	1457 – 1482	932.4104	2794.2094	2794.2093	0.013	1	75	1.5e-06	1	U	K.SEDDSAKFDSNEEDSASVFSPSFGLK.Q
11873	1457 – 1482	932.4108	2794.2105	2794.2093	0.41	1	41	0.0011	1	U	K.SEDDSAKFDSNEEDSASVFSPSFGLK.Q
11874	1457 – 1482	932.4110	2794.2112	2794.2093	0.68	1	87	1.1e-07	1	U	K.SEDDSAKFDSNEEDSASVFSPSFGLK.Q
11876	1457 – 1482	932.4114	2794.2124	2794.2093	1.11	1	45	0.00036	1	U	K.SEDDSAKFDSNEEDSASVFSPSFGLK.Q
12048	1457 – 1482	959.0650	2874.1732	2874.1757	-0.87	1	29	0.018	1	U	K.SEDDSAKFDSNEEDSASVFSPSFGLK.Q + Phospho (ST)
12049	1457 – 1482	959.0655	2874.1746	2874.1757	-0.39	1	43	0.0025	1	U	K.SEDDSAKFDSNEEDSASVFSPSFGLK.Q + Phospho (ST)
9858	1464 – 1482	1031.9638	2061.9129	2061.9167	-1.84	0	79	2.1e-07	1	U	K.FDSNEEDSASVFSPSFGLK.Q
9859	1464 – 1482	1031.9640	2061.9134	2061.9167	-1.61	0	35	0.0066	1	U	K.FDSNEEDSASVFSPSFGLK.Q
9861	1464 – 1482	1031.9642	2061.9139	2061.9167	-1.36	0	25	0.019	1	U	K.FDSNEEDSASVFSPSFGLK.Q
9862	1464 – 1482	1031.9648	2061.9151	2061.9167	-0.77	0	76	3.3e-07	1	U	K.FDSNEEDSASVFSPSFGLK.Q
9865	1464 – 1482	1031.9651	2061.9156	2061.9167	-0.54	0	105	2.3e-09	1	U	K.FDSNEEDSASVFSPSFGLK.Q
9866	1464 – 1482	1031.9651	2061.9156	2061.9167	-0.53	0	22	0.013	1	U	K.FDSNEEDSASVFSPSFGLK.Q
9867	1464 – 1482	1031.9652	2061.9158	2061.9167	-0.46	0	68	2.9e-06	1	U	K.FDSNEEDSASVFSPSFGLK.Q
9868	1464 – 1482	1031.9652	2061.9159	2061.9167	-0.39	0	25	0.011	1	U	K.FDSNEEDSASVFSPSFGLK.Q
9870	1464 – 1482	1031.9654	2061.9162	2061.9167	-0.25	0	83	1.6e-08	1	U	K.FDSNEEDSASVFSPSFGLK.Q
9871	1464 – 1482	1031.9654	2061.9163	2061.9167	-0.21	0	73	5e-07	1	U	K.FDSNEEDSASVFSPSFGLK.Q
9872	1464 – 1482	1031.9654	2061.9163	2061.9167	-0.20	0	53	0.00023	1	U	K.FDSNEEDSASVFSPSFGLK.Q
9873	1464 – 1482	1031.9656	2061.9165	2061.9167	-0.090	0	59	7.9e-06	1	U	K.FDSNEEDSASVFSPSFGLK.Q
9874	1464 – 1482	688.3128	2061.9166	2061.9167	-0.078	0	28	0.015	1	U	K.FDSNEEDSASVFSPSFGLK.Q
9875	1464 – 1482	688.3128	2061.9166	2061.9167	-0.064	0	40	0.0045	1	U	K.FDSNEEDSASVFSPSFGLK.Q
9876	1464 – 1482	1031.9657	2061.9168	2061.9167	0.036	0	51	4e-05	1	U	K.FDSNEEDSASVFSPSFGLK.Q
9877	1464 – 1482	1031.9657	2061.9168	2061.9167	0.046	0	36	0.0023	1	U	K.FDSNEEDSASVFSPSFGLK.Q
9878	1464 – 1482	1031.9657	2061.9168	2061.9167	0.056	0	105	5.5e-10	1	U	K.FDSNEEDSASVFSPSFGLK.Q
9879	1464 – 1482	688.3130	2061.9170	2061.9167	0.14	0	26	0.0074	1	U	K.FDSNEEDSASVFSPSFGLK.Q
9880	1464 – 1482	1031.9660	2061.9175	2061.9167	0.37	0	52	4.1e-05	1	U	K.FDSNEEDSASVFSPSFGLK.Q
9882	1464 – 1482	1031.9661	2061.9176	2061.9167	0.43	0	55	9e-05	1	U	K.FDSNEEDSASVFSPSFGLK.Q
9883	1464 – 1482	688.3132	2061.9179	2061.9167	0.56	0	21	0.037	1	U	K.FDSNEEDSASVFSPSFGLK.Q
9884	1464 – 1482	1031.9663	2061.9180	2061.9167	0.63	0	27	0.0065	1	U	K.FDSNEEDSASVFSPSFGLK.Q
9885	1464 – 1482	1031.9667	2061.9189	2061.9167	1.05	0	29	0.0082	1	U	K.FDSNEEDSASVFSPSFGLK.Q
9886	1464 – 1482	1031.9667	2061.9189	2061.9167	1.05	0	59	7e-05	1	U	K.FDSNEEDSASVFSPSFGLK.Q
9887	1464 – 1482	1031.9673	2061.9200	2061.9167	1.60	0	27	0.0097	1	U	K.FDSNEEDSASVFSPSFGLK.Q
10176	1464 – 1482	1071.9475	2141.8804	2141.8831	-1.24	0	57	2.2e-05	1	U	K.FDSNEEDSASVFSPSFGLK.Q + Phospho (ST)
10177	1464 – 1482	1071.9480	2141.8814	2141.8831	-0.79	0	68	2.8e-06	1	U	K.FDSNEEDSASVFSPSFGLK.Q + Phospho (ST)
10178	1464 – 1482	1071.9485	2141.8824	2141.8831	-0.29	0	60	8.3e-06	1	U	K.FDSNEEDSASVFSPSFGLK.Q + Phospho (ST)
4575	1496 – 1508	456.9330	1367.7772	1367.7773	-0.015	1	46	8.4e-05	1	U	K.KGKPSSDTVPKPK.R
4576	1496 – 1508	684.8961	1367.7776	1367.7773	0.25	1	40	0.0015	1	U	K.KGKPSSDTVPKPK.R
4577	1496 – 1508	456.9332	1367.7778	1367.7773	0.40	1	26	0.007	1	U	K.KGKPSSDTVPKPK.R
3530	1497 – 1508	414.2345	1239.6817	1239.6823	-0.48	0	27	0.016	1	U	K.GKPSSDTVPKPK.R
3534	1497 – 1508	620.8484	1239.6823	1239.6823	-0.023	0	33	0.0012	1	U	K.GKPSSDTVPKPK.R
3535	1497 – 1508	414.2348	1239.6827	1239.6823	0.29	0	24	0.022	1	U	K.GKPSSDTVPKPK.R
3536	1497 – 1508	414.2349	1239.6828	1239.6823	0.44	0	39	0.003	1	U	K.GKPSSDTVPKPK.R
4238	1497 – 1508	440.8899	1319.6479	1319.6486	-0.54	0	36	0.0063	1	U	K.GKPSSDTVPKPK.R + Phospho (ST)
4239	1497 – 1508	440.8900	1319.6481	1319.6486	-0.41	0	29	0.0091	1	U	K.GKPSSDTVPKPK.R + Phospho (ST)
9284	1515 – 1532	640.9894	1919.9464	1919.9476	-0.64	1	24	0.019	1	U	K.KVVEAVNSDSDSEFGIPK.K
9285	1515 – 1532	640.9895	1919.9467	1919.9476	-0.48	1	59	5e-05	1	U	K.KVVEAVNSDSDSEFGIPK.K
9287	1515 – 1532	960.9813	1919.9480	1919.9476	0.20	1	107	2.4e-10	1	U	K.KVVEAVNSDSDSEFGIPK.K

Query	Start - End	Observed	Mr(expt)	Mr(calc)	ppm	M	Score	Expect	Rank	U	Peptide
9619	1515 - 1532	667.6450	1999.9131	1999.9140	-0.45	1	27	0.026	1	U	K.KVVEAVNSDSDSEFGIPK.K + Phospho (ST)
9621	1515 - 1532	1000.9647	1999.9148	1999.9140	0.41	1	46	0.0018	1	U	K.KVVEAVNSDSDSEFGIPK.K + Phospho (ST)
9622	1515 - 1532	1000.9647	1999.9149	1999.9140	0.47	1	48	7.6e-05	1	U	K.KVVEAVNSDSDSEFGIPK.K + Phospho (ST)
9623	1515 - 1532	667.6456	1999.9150	1999.9140	0.51	1	36	0.0047	1	U	K.KVVEAVNSDSDSEFGIPK.K + Phospho (ST)
9624	1515 - 1532	667.6465	1999.9176	1999.9140	1.83	1	23	0.043	1	U	K.KVVEAVNSDSDSEFGIPK.K + Phospho (ST)
8347	1516 - 1532	896.9336	1791.8525	1791.8527	-0.071	0	70	3.8e-07	1	U	K.VVEAVNSDSDSEFGIPK.K
8348	1516 - 1532	896.9341	1791.8536	1791.8527	0.51	0	65	2.5e-06	1	U	K.VVEAVNSDSDSEFGIPK.K
8946	1516 - 1532	936.9156	1871.8166	1871.8190	-1.28	0	60	9.6e-06	1	U	K.VVEAVNSDSDSEFGIPK.K + Phospho (ST)
8947	1516 - 1532	936.9170	1871.8195	1871.8190	0.28	0	71	1.4e-06	1	U	K.VVEAVNSDSDSEFGIPK.K + Phospho (ST)
8579	1547 - 1562	606.2584	1815.7533	1815.7537	-0.23	2	54	0.0003	1	U	K.RKASGSENEGDYNPGR.K + Phospho (ST)
9408	1547 - 1563	648.9568	1943.8484	1943.8486	-0.11	3	34	0.0065	1	U	K.RKASGSENEGDYNPGRK.T + Phospho (ST)
6557	1548 - 1562	527.5688	1579.6847	1579.6862	-0.99	1	40	0.001	1	U	R.KASGSENEGDYNPGR.K
6559	1548 - 1562	790.8503	1579.6860	1579.6862	-0.18	1	35	0.0018	1	U	R.KASGSENEGDYNPGR.K
7185	1548 - 1562	830.8333	1659.6520	1659.6526	-0.32	1	32	0.002	1	U	R.KASGSENEGDYNPGR.K + Phospho (ST)
7187	1548 - 1562	830.8342	1659.6538	1659.6526	0.72	1	45	0.00017	1	U	R.KASGSENEGDYNPGR.K + Phospho (ST)
7188	1548 - 1562	554.2253	1659.6541	1659.6526	0.92	1	39	0.00094	1	U	R.KASGSENEGDYNPGR.K + Phospho (ST)
7893	1548 - 1562	870.8167	1739.6189	1739.6189	0.0069	1	27	0.005	1	U	R.KASGSENEGDYNPGR.K + 2 Phospho (ST)
7894	1548 - 1562	870.8172	1739.6199	1739.6189	0.57	1	36	0.0017	1	U	R.KASGSENEGDYNPGR.K + 2 Phospho (ST)
7895	1548 - 1562	870.8175	1739.6204	1739.6189	0.85	1	30	0.021	1	U	R.KASGSENEGDYNPGR.K + 2 Phospho (ST)
7600	1548 - 1563	570.2678	1707.7816	1707.7812	0.23	2	53	7.6e-05	1	U	R.KASGSENEGDYNPGRK.T
7601	1548 - 1563	854.8982	1707.7819	1707.7812	0.41	2	59	2.1e-05	1	U	R.KASGSENEGDYNPGRK.T
7602	1548 - 1563	854.8999	1707.7853	1707.7812	2.41	2	46	0.00017	1	U	R.KASGSENEGDYNPGRK.T
8275	1548 - 1563	894.8809	1787.7473	1787.7475	-0.15	2	34	0.0037	1	U	R.KASGSENEGDYNPGRK.T + Phospho (ST)
8276	1548 - 1563	596.9233	1787.7480	1787.7475	0.25	2	43	0.00087	1	U	R.KASGSENEGDYNPGRK.T + Phospho (ST)
5178	1549 - 1562	726.8020	1451.5894	1451.5913	-1.31	0	44	0.00047	1	U	K.ASGSENEGDYNPGR.K
5179	1549 - 1562	726.8028	1451.5910	1451.5913	-0.20	0	40	0.0012	1	U	K.ASGSENEGDYNPGR.K
5180	1549 - 1562	726.8029	1451.5912	1451.5913	-0.045	0	40	0.0004	1	U	K.ASGSENEGDYNPGR.K
5181	1549 - 1562	484.8715	1451.5927	1451.5913	0.97	0	44	0.0008	1	U	K.ASGSENEGDYNPGR.K
6077	1549 - 1562	766.7861	1531.5576	1531.5576	-0.032	0	51	0.0002	1	U	K.ASGSENEGDYNPGR.K + Phospho (ST)
6078	1549 - 1562	766.7861	1531.5577	1531.5576	0.033	0	55	0.0001	1	U	K.ASGSENEGDYNPGR.K + Phospho (ST)
6079	1549 - 1562	766.7863	1531.5581	1531.5576	0.29	0	38	0.0016	1	U	K.ASGSENEGDYNPGR.K + Phospho (ST)
6080	1549 - 1562	766.7867	1531.5589	1531.5576	0.86	0	54	3.1e-05	1	U	K.ASGSENEGDYNPGR.K + Phospho (ST)
6561	1549 - 1563	790.8504	1579.6862	1579.6862	-0.051	1	59	5.6e-05	1	U	K.ASGSENEGDYNPGRK.T
7184	1549 - 1563	830.8330	1659.6513	1659.6526	-0.74	1	39	0.0012	1	U	K.ASGSENEGDYNPGRK.T + Phospho (ST)
7186	1549 - 1563	554.2250	1659.6531	1659.6526	0.29	1	26	0.0064	1	U	K.ASGSENEGDYNPGRK.T + Phospho (ST)
12179	1574 - 1599	979.7966	2936.3680	2936.3716	-1.23	1	26	0.017	1	U	K.KTSFDQDSDVDIFPSDFPTEPPSLPR.T
12180	1574 - 1599	979.7966	2936.3681	2936.3716	-1.20	1	25	0.033	1	U	K.KTSFDQDSDVDIFPSDFPTEPPSLPR.T
12183	1574 - 1599	979.7969	2936.3688	2936.3716	-0.98	1	21	0.028	1	U	K.KTSFDQDSDVDIFPSDFPTEPPSLPR.T
12185	1574 - 1599	979.7971	2936.3696	2936.3716	-0.69	1	39	0.0035	1	U	K.KTSFDQDSDVDIFPSDFPTEPPSLPR.T
12186	1574 - 1599	979.7972	2936.3697	2936.3716	-0.65	1	44	0.0023	1	U	K.KTSFDQDSDVDIFPSDFPTEPPSLPR.T
12188	1574 - 1599	979.7974	2936.3703	2936.3716	-0.46	1	29	0.018	1	U	K.KTSFDQDSDVDIFPSDFPTEPPSLPR.T
12191	1574 - 1599	979.7980	2936.3721	2936.3716	0.15	1	50	0.00014	1	U	K.KTSFDQDSDVDIFPSDFPTEPPSLPR.T
12193	1574 - 1599	979.7988	2936.3746	2936.3716	1.00	1	35	0.0067	1	U	K.KTSFDQDSDVDIFPSDFPTEPPSLPR.T
12194	1574 - 1599	735.1012	2936.3755	2936.3716	1.32	1	37	0.022	1	U	K.KTSFDQDSDVDIFPSDFPTEPPSLPR.T
11927	1575 - 1599	937.1001	2808.2784	2808.2767	0.61	0	21	0.042	1	U	K.TSFDQDSDVDIFPSDFPTEPPSLPR.T
11928	1575 - 1599	937.1002	2808.2788	2808.2767	0.75	0	28	0.045	1	U	K.TSFDQDSDVDIFPSDFPTEPPSLPR.T
10265	1609 - 1626	1086.9138	2171.8131	2171.8154	-1.03	0	36	0.0013	1	U	K.YFAESDEEEDDVDFAMFN.-
10268	1609 - 1626	1086.9141	2171.8136	2171.8154	-0.81	0	18	0.022	1	U	K.YFAESDEEEDDVDFAMFN.-
10273	1609 - 1626	1086.9148	2171.8150	2171.8154	-0.18	0	32	0.0012	1	U	K.YFAESDEEEDDVDFAMFN.-
10326	1609 - 1626	1094.9114	2187.8082	2187.8103	-0.93	0	53	3.4e-05	1	U	K.YFAESDEEEDDVDFAMFN.- + Oxidation (M)
10327	1609 - 1626	1094.9120	2187.8095	2187.8103	-0.34	0	40	0.00025	1	U	K.YFAESDEEEDDVDFAMFN.- + Oxidation (M)
10328	1609 - 1626	1094.9122	2187.8098	2187.8103	-0.21	0	55	1.1e-05	1	U	K.YFAESDEEEDDVDFAMFN.- + Oxidation (M)
10330	1609 - 1626	1094.9122	2187.8099	2187.8103	-0.16	0	55	7.3e-06	1	U	K.YFAESDEEEDDVDFAMFN.- + Oxidation (M)
10331	1609 - 1626	1094.9124	2187.8102	2187.8103	-0.038	0	55	9.5e-06	1	U	K.YFAESDEEEDDVDFAMFN.- + Oxidation (M)
10332	1609 - 1626	1094.9125	2187.8104	2187.8103	0.053	0	44	0.00011	1	U	K.YFAESDEEEDDVDFAMFN.- + Oxidation (M)

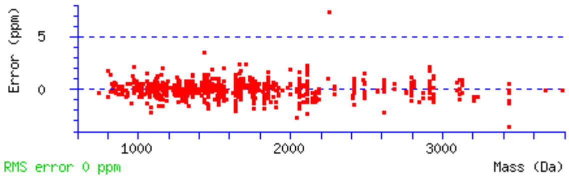

Supplement: Supplementary file 4 — Supplementary Data 1 [file 41467_2023_44089_MOESM4_ESM.pdf]
