# Supplementary material for: ERK2-topoisomerase II regulatory axis is important for gene activation in immediate early genes
Source: Nat Commun. 2023 Dec 14;14:8341. doi: 10.1038/s41467-023-44089-y (PMC10721843; doi:10.1038/s41467-023-44089-y)

MASCOT Search Results

Protein View: Q02880

DNA topoisomerase 2-beta OS=Homo sapiens OX=9606 GN=TOP2B PE=1 SV=3

**Database:**	Uniprot_Proteome_Human
**Score:**	20719
**Monoisotopic mass (M_r):**	184122
**Calculated pI:**	8.14

Sequence similarity is available as **an NCBI BLAST search of Q02880 against nr**.

Search parameters

**MS data file:**	File Name: Z:\SN22\SN221363_deTOP2B_Erk1_1ul.raw;File Path: ;File Time: 3/1/2023 9:56:14 AM;File Size: 347522226 [Byte]
**Enzyme:**	Trypsin: cuts C-term side of KR unless next residue is P.
**Fixed modifications:**	**Carbamidomethyl (C)**
**Variable modifications:**	**Acetyl (Protein N-term)**, **Oxidation (M)**, **Phospho (ST)**, **Phospho (Y)**

Protein sequence coverage: 65%

Matched peptides shown in ***bold red***.

```
   1 MAKSGGCGAG AGVGGGNGAL TWVTLFDQNN AAKKEESETA NKNDSSKKLS
  51 VERVYQKKTQ LEHILLRPDT YIGSVEPLTQ FMWVYDEDVG MNCREVTFVP
 101 GLYKIFDEIL VNAADNKQRD KNMTCIKVSI DPESNIISIW NNGKGIPVVE
 151 HKVEKVYVPA LIFGQLLTSS NYDDDEKKVT GGRNGYGAKL CNIFSTKFTV
 201 ETACKEYKHS FKQTWMNNMM KTSEAKIKHF DGEDYTCITF QPDLSKFKME
 251 KLDKDIVALM TRRAYDLAGS CRGVKVMFNG KKLPVNGFRS YVDLYVKDKL
 301 DETGVALKVI HELANERWDV CLTLSEKGFQ QISFVNSIAT TKGGRHVDYV
 351 VDQVVGKLIE VVKKKNKAGV SVKPFQVKNH IWVFINCLIE NPTFDSQTKE
 401 NMTLQPKSFG SKCQLSEKFF KAASNCGIVE SILNWVKFKA QTQLNKKCSS
 451 VKYSKIKGIP KLDDANDAGG KHSLECTLIL TEGDSAKSLA VSGLGVIGRD
 501 RYGVFPLRGK ILNVREASHK QIMENAEINN IIKIVGLQYK KSYDDAESLK
 551 TLRYGKIMIM TDQDQDGSHI KGLLINFIHH NWPSLLKHGF LEEFITPIVK
 601 ASKNKQELSF YSIPEFDEWK KHIENQKAWK IKYYKGLGTS TAKEAKEYFA
 651 DMERHRILFR YAGPEDDAAI TLAFSKKKID DRKEWLTNFM EDRRQRRLHG
 701 LPEQFLYGTA TKHLTYNDFI NKELILFSNS DNERSIPSLV DGFKPGQRKV
 751 LFTCFKRNDK REVKVAQLAG SVAEMSAYHH GEQALMMTIV NLAQNFVGSN
 801 NINLLQPIGQ FGTRLHGGKD AASPRYIFTM LSTLARLLFP AVDDNLLKFL
 851 YDDNQRVEPE WYIPIIPMVL INGAEGIGTG WACKLPNYDA REIVNNVRRM
 901 LDGLDPHPML PNYKNFKGTI QELGQNQYAV SGEIFVVDRN TVEITELPVR
 951 TWTQVYKEQV LEPMLNGTDK TPALISDYKE YHTDTTVKFV VKMTEEKLAQ
1001 AEAAGLHKVF KLQTTLTCNS MVLFDHMGCL KKYETVQDIL KEFFDLRLSY
1051 YGLRKEWLVG MLGAESTKLN NQARFILEKI QGKITIENRS KKDLIQMLVQ
1101 RGYESDPVKA WKEAQEKAAE EDETQNQHDD SSSDSGTPSG PDFNYILNMS
1151 LWSLTKEKVE ELIKQRDAKG REVNDLKRKS PSDLWKEDLA AFVEELDKVE
1201 SQEREDVLAG MSGKAIKGKV GKPKVKKLQL EETMPSPYGR RIIPEITAMK
1251 ADASKKLLKK KKGDLDTAAV KVEFDEEFSG APVEGAGEEA LTPSVPINKG
1301 PKPKREKKEP GTRVRKTPTS SGKPSAKKVK KRNPWSDDES KSESDLEETE
1351 PVVIPRDSLL RRAAAERPKY TFDFSEEEDD DADDDDDDNN DLEELKVKAS
1401 PITNDGEDEF VPSDGLDKDE YTFSPGKSKA TPEKSLHDKK SQDFGNLFSF
1451 PSYSQKSEDD SAKFDSNEED SASVFSPSFG LKQTDKVPSK TVAAKKGKPS
1501 SDTVPKPKRA PKQKKVVEAV NSDSDSEFGI PKKTTTPKGK GRGAKKRKAS
1551 GSENEGDYNP GRKTSKTTSK KPKKTSFDQD SDVDIFPSDF PTEPPSLPRT
1601 GRARKEVKYF AESDEEEDDV DFAMFN
```

Unformatted sequence string: **1626 residues** (for pasting into other applications).

Sort by ◉ residue number ○ increasing mass ○ decreasing mass
Show ◉ matched peptides only ○ predicted peptides also

Query	Start – End	Observed	Mr(expt)	Mr(calc)	ppm	M	Score	Expect	Rank	U	Peptide
1783	34 – 42	518.2516	1034.4885	1034.4880	0.55	1	39	0.0078	1	U	K.KEESETANK.N
6329	34 – 47	783.8656	1565.7166	1565.7169	-0.17	2	41	0.00034	1	U	K.KEESETANKNDSSK.K
6330	34 – 47	522.9129	1565.7168	1565.7169	-0.068	2	31	0.04	1	U	K.KEESETANKNDSSK.K
6332	34 – 47	783.8659	1565.7172	1565.7169	0.24	2	43	0.00024	1	U	K.KEESETANKNDSSK.K
6333	34 – 47	783.8659	1565.7173	1565.7169	0.29	2	34	0.0075	1	U	K.KEESETANKNDSSK.K
7336	34 – 48	565.6114	1693.8123	1693.8118	0.26	3	31	0.007	1	U	K.KEESETANKNDSSKK.L
7337	34 – 48	847.9137	1693.8129	1693.8118	0.65	3	38	0.0021	1	U	K.KEESETANKNDSSKK.L
2831	95 – 104	576.8188	1151.6230	1151.6227	0.26	0	18	0.026	1		R.EVTFVPGLYK.I
5188	105 – 117	731.3816	1460.7487	1460.7511	-1.64	0	50	0.00082	1		K.IFDEILVNAADNK.Q
5189	105 – 117	487.9235	1460.7487	1460.7511	-1.63	0	46	0.00048	1		K.IFDEILVNAADNK.Q
5190	105 – 117	731.3822	1460.7498	1460.7511	-0.90	0	90	8.3e-08	1		K.IFDEILVNAADNK.Q
5191	105 – 117	487.9240	1460.7500	1460.7511	-0.73	0	27	0.021	1		K.IFDEILVNAADNK.Q
5193	105 – 117	731.3824	1460.7502	1460.7511	-0.60	0	51	9.3e-05	1		K.IFDEILVNAADNK.Q
5195	105 – 117	487.9241	1460.7504	1460.7511	-0.44	0	33	0.0079	1		K.IFDEILVNAADNK.Q
5196	105 – 117	487.9241	1460.7505	1460.7511	-0.42	0	27	0.018	1		K.IFDEILVNAADNK.Q
5197	105 – 117	487.9241	1460.7505	1460.7511	-0.40	0	27	0.028	1		K.IFDEILVNAADNK.Q
5198	105 – 117	487.9241	1460.7505	1460.7511	-0.40	0	38	0.0026	1		K.IFDEILVNAADNK.Q
5199	105 – 117	731.3826	1460.7506	1460.7511	-0.34	0	66	5.1e-06	1		K.IFDEILVNAADNK.Q
5200	105 – 117	487.9241	1460.7506	1460.7511	-0.34	0	66	1.5e-05	1		K.IFDEILVNAADNK.Q

Query	Start - End	Observed	Mr(expt)	Mr(calc)	ppm	M	Score	Expect	Rank	U	Peptide
5201	105 - 117	487.9242	1460.7507	1460.7511	-0.23	0	33	0.0047	1		K.IFDEILVNAADNK.Q
5202	105 - 117	731.3827	1460.7508	1460.7511	-0.16	0	74	1.7e-06	1		K.IFDEILVNAADNK.Q
5203	105 - 117	487.9242	1460.7509	1460.7511	-0.11	0	38	0.0034	1		K.IFDEILVNAADNK.Q
5204	105 - 117	731.3828	1460.7509	1460.7511	-0.092	0	58	2.1e-05	1		K.IFDEILVNAADNK.Q
5205	105 - 117	487.9243	1460.7510	1460.7511	-0.090	0	33	0.0085	1		K.IFDEILVNAADNK.Q
5206	105 - 117	487.9243	1460.7510	1460.7511	-0.069	0	39	0.0036	1		K.IFDEILVNAADNK.Q
5207	105 - 117	731.3828	1460.7510	1460.7511	-0.065	0	81	6e-07	1		K.IFDEILVNAADNK.Q
5208	105 - 117	487.9243	1460.7510	1460.7511	-0.049	0	29	0.012	1		K.IFDEILVNAADNK.Q
5209	105 - 117	487.9243	1460.7511	1460.7511	0.034	0	27	0.028	1		K.IFDEILVNAADNK.Q
5210	105 - 117	487.9244	1460.7513	1460.7511	0.12	0	35	0.0039	1		K.IFDEILVNAADNK.Q
5211	105 - 117	487.9244	1460.7513	1460.7511	0.14	0	27	0.013	1		K.IFDEILVNAADNK.Q
5212	105 - 117	731.3829	1460.7513	1460.7511	0.17	0	83	2.8e-07	1		K.IFDEILVNAADNK.Q
5213	105 - 117	731.3830	1460.7513	1460.7511	0.18	0	83	1e-07	1		K.IFDEILVNAADNK.Q
5214	105 - 117	731.3830	1460.7515	1460.7511	0.26	0	75	6.9e-07	1		K.IFDEILVNAADNK.Q
5215	105 - 117	487.9245	1460.7517	1460.7511	0.42	0	32	0.0078	1		K.IFDEILVNAADNK.Q
5217	105 - 117	731.3834	1460.7523	1460.7511	0.82	0	70	2.9e-06	1		K.IFDEILVNAADNK.Q
5218	105 - 117	487.9248	1460.7525	1460.7511	0.96	0	61	3.1e-05	1		K.IFDEILVNAADNK.Q
5219	105 - 117	731.3835	1460.7525	1460.7511	0.96	0	71	1.6e-06	1		K.IFDEILVNAADNK.Q
7742	105 - 119	582.6440	1744.9102	1744.9108	-0.31	1	28	0.016	1		K.IFDEILVNAADNKQR.D
8763	128 - 144	943.4867	1884.9589	1884.9581	0.42	0	73	7.4e-07	1	U	K.VSIDPESNIISIWNNGK.G
8764	128 - 144	943.4868	1884.9590	1884.9581	0.46	0	57	6.7e-06	1	U	K.VSIDPESNIISIWNNGK.G
8765	128 - 144	629.3272	1884.9597	1884.9581	0.82	0	56	5.6e-05	1	U	K.VSIDPESNIISIWNNGK.G
11599	153 - 177	948.4836	2842.4291	2842.4276	0.52	1	34	0.0023	1	U	K.VEKVYVPALIFGQLLTSSNYDDDEK.K
10836	156 - 177	829.7460	2486.2163	2486.2217	-2.16	0	32	0.002	1	U	K.VYVPALIFGQLLTSSNYDDDEK.K
10837	156 - 177	829.7467	2486.2182	2486.2217	-1.42	0	30	0.0027	1	U	K.VYVPALIFGQLLTSSNYDDDEK.K
10840	156 - 177	829.7480	2486.2222	2486.2217	0.21	0	35	0.0048	1	U	K.VYVPALIFGQLLTSSNYDDDEK.K
10841	156 - 177	829.7481	2486.2224	2486.2217	0.27	0	32	0.008	1	U	K.VYVPALIFGQLLTSSNYDDDEK.K
10842	156 - 177	1244.1186	2486.2226	2486.2217	0.37	0	80	6.4e-08	1	U	K.VYVPALIFGQLLTSSNYDDDEK.K
10843	156 - 177	829.7482	2486.2227	2486.2217	0.42	0	59	1.2e-05	1	U	K.VYVPALIFGQLLTSSNYDDDEK.K
10845	156 - 177	1244.1189	2486.2233	2486.2217	0.65	0	53	2.2e-05	1	U	K.VYVPALIFGQLLTSSNYDDDEK.K
10846	156 - 177	1244.1191	2486.2236	2486.2217	0.78	0	62	4e-06	1	U	K.VYVPALIFGQLLTSSNYDDDEK.K
10847	156 - 177	1244.1196	2486.2246	2486.2217	1.18	0	38	0.00067	1	U	K.VYVPALIFGQLLTSSNYDDDEK.K
11116	156 - 178	872.4460	2614.3162	2614.3166	-0.18	1	44	0.0016	1	U	K.VYVPALIFGQLLTSSNYDDDEKK.V
11117	156 - 178	654.5863	2614.3162	2614.3166	-0.16	1	47	0.0025	1	U	K.VYVPALIFGQLLTSSNYDDDEKK.V
11118	156 - 178	872.4460	2614.3163	2614.3166	-0.12	1	39	0.013	1	U	K.VYVPALIFGQLLTSSNYDDDEKK.V
11122	156 - 178	654.5866	2614.3171	2614.3166	0.19	1	26	0.049	1	U	K.VYVPALIFGQLLTSSNYDDDEKK.V
11123	156 - 178	654.5866	2614.3172	2614.3166	0.21	1	34	0.014	1	U	K.VYVPALIFGQLLTSSNYDDDEKK.V
11124	156 - 178	872.4464	2614.3172	2614.3166	0.22	1	55	0.00016	1	U	K.VYVPALIFGQLLTSSNYDDDEKK.V
11125	156 - 178	872.4464	2614.3173	2614.3166	0.24	1	42	0.0035	1	U	K.VYVPALIFGQLLTSSNYDDDEKK.V
11126	156 - 178	1308.1659	2614.3173	2614.3166	0.27	1	73	5.6e-06	1	U	K.VYVPALIFGQLLTSSNYDDDEKK.V
1392	190 - 197	491.7552	981.4958	981.4953	0.45	0	49	0.00016	1		K.LCNIFSTK.F
1201	198 - 205	478.2310	954.4475	954.4481	-0.61	0	21	0.046	1	U	K.FTVETACK.E
3073	213 - 221	592.2559	1182.4972	1182.4984	-1.03	0	25	0.018	1	U	K.QTWMNNMMK.T
3180	213 - 221	600.2537	1198.4928	1198.4933	-0.41	0	31	0.0066	1	U	K.QTWMNNMMK.T + Oxidation (M)
3181	213 - 221	600.2539	1198.4932	1198.4933	-0.061	0	28	0.038	1	U	K.QTWMNNMMK.T + Oxidation (M)
3182	213 - 221	600.2539	1198.4933	1198.4933	-0.011	0	30	0.0059	1	U	K.QTWMNNMMK.T + Oxidation (M)
3283	213 - 221	608.2511	1214.4876	1214.4882	-0.49	0	26	0.0062	1	U	K.QTWMNNMMK.T + 2 Oxidation (M)
9938	229 - 246	724.9894	2171.9463	2171.9470	-0.32	0	28	0.015	1	U	K.HFDGEDYTCITFQPDLSK.F
3795	252 - 262	637.8592	1273.7038	1273.7064	-2.06	1	50	0.00013	1	U	K.LDKDIVALMTR.R
3797	252 - 262	425.5755	1273.7046	1273.7064	-1.37	1	18	0.042	1	U	K.LDKDIVALMTR.R
3798	252 - 262	637.8598	1273.7051	1273.7064	-0.99	1	50	0.00025	1	U	K.LDKDIVALMTR.R
3799	252 - 262	637.8599	1273.7052	1273.7064	-0.90	1	35	0.0037	1	U	K.LDKDIVALMTR.R
3803	252 - 262	637.8602	1273.7058	1273.7064	-0.44	1	26	0.0041	1	U	K.LDKDIVALMTR.R
3806	252 - 262	637.8604	1273.7061	1273.7064	-0.19	1	46	0.00093	1	U	K.LDKDIVALMTR.R
3809	252 - 262	637.8604	1273.7063	1273.7064	-0.033	1	74	4.7e-07	1	U	K.LDKDIVALMTR.R
3811	252 - 262	637.8605	1273.7064	1273.7064	0.030	1	48	0.00014	1	U	K.LDKDIVALMTR.R
3812	252 - 262	637.8605	1273.7064	1273.7064	0.046	1	35	0.00078	1	U	K.LDKDIVALMTR.R
3813	252 - 262	637.8607	1273.7068	1273.7064	0.34	1	30	0.0067	1	U	K.LDKDIVALMTR.R
3814	252 - 262	637.8611	1273.7077	1273.7064	1.02	1	29	0.02	1	U	K.LDKDIVALMTR.R
3815	252 - 262	637.8617	1273.7088	1273.7064	1.90	1	77	7.5e-07	1	U	K.LDKDIVALMTR.R
3816	252 - 262	425.5771	1273.7094	1273.7064	2.37	1	35	0.013	1	U	K.LDKDIVALMTR.R
3929	252 - 262	645.8572	1289.6998	1289.7013	-1.18	1	38	0.0017	1	U	K.LDKDIVALMTR.R + Oxidation (M)
3930	252 - 262	430.9072	1289.6998	1289.7013	-1.17	1	25	0.017	1	U	K.LDKDIVALMTR.R + Oxidation (M)
3931	252 - 262	430.9075	1289.7007	1289.7013	-0.49	1	23	0.029	1	U	K.LDKDIVALMTR.R + Oxidation (M)
3932	252 - 262	430.9076	1289.7009	1289.7013	-0.35	1	26	0.032	1	U	K.LDKDIVALMTR.R + Oxidation (M)
3933	252 - 262	645.8579	1289.7012	1289.7013	-0.094	1	41	0.0009	1	U	K.LDKDIVALMTR.R + Oxidation (M)
3934	252 - 262	430.9077	1289.7012	1289.7013	-0.052	1	24	0.032	1	U	K.LDKDIVALMTR.R + Oxidation (M)
3935	252 - 262	645.8579	1289.7013	1289.7013	-0.032	1	30	0.0077	1	U	K.LDKDIVALMTR.R + Oxidation (M)
3936	252 - 262	430.9077	1289.7014	1289.7013	0.064	1	37	0.013	1	U	K.LDKDIVALMTR.R + Oxidation (M)
3938	252 - 262	645.8580	1289.7015	1289.7013	0.14	1	43	0.00018	1	U	K.LDKDIVALMTR.R + Oxidation (M)
3939	252 - 262	645.8580	1289.7015	1289.7013	0.19	1	42	0.00022	1	U	K.LDKDIVALMTR.R + Oxidation (M)
3940	252 - 262	645.8581	1289.7017	1289.7013	0.28	1	55	3e-05	1	U	K.LDKDIVALMTR.R + Oxidation (M)
3943	252 - 262	645.8583	1289.7021	1289.7013	0.62	1	64	4.6e-06	1	U	K.LDKDIVALMTR.R + Oxidation (M)
995	255 - 262	459.7579	917.5012	917.5004	0.89	0	49	0.00038	1	U	K.DIVALMTR.R
1093	255 - 262	467.7547	933.4948	933.4953	-0.55	0	37	0.011	1	U	K.DIVALMTR.R + Oxidation (M)
2968	263 - 272	584.7804	1167.5463	1167.5455	0.72	1	40	0.0011	1	U	R.RAYDLAGSCR.G
1601	264 - 272	506.7295	1011.4445	1011.4444	0.14	0	42	0.00046	1	U	R.AYDLAGSCR.G

Query	Start – End	Observed	Mr(expt)	Mr(calc)	ppm	M	Score	Expect	Rank	U	Peptide
1421	290 – 297	493.7638	985.5131	985.5120	1.04	0	43	0.00061	1	U	R.SYVDLYVK.D
1422	290 – 297	493.7638	985.5131	985.5120	1.04	0	28	0.023	1	U	R.SYVDLYVK.D
3401	290 – 299	615.3239	1228.6333	1228.6339	-0.55	1	28	0.014	1	U	R.SYVDLYVKDK.L
3112	298 – 308	594.8266	1187.6386	1187.6398	-0.97	1	51	0.00028	1	U	K.DKLDETGVALK.V
3113	298 – 308	396.8869	1187.6389	1187.6398	-0.72	1	36	0.0044	1	U	K.DKLDETGVALK.V
3115	298 – 308	594.8268	1187.6390	1187.6398	-0.61	1	28	0.016	1	U	K.DKLDETGVALK.V
3118	298 – 308	594.8272	1187.6399	1187.6398	0.15	1	33	0.014	1	U	K.DKLDETGVALK.V
3119	298 – 308	594.8272	1187.6399	1187.6398	0.15	1	37	0.0029	1	U	K.DKLDETGVALK.V
1156	300 – 308	473.2665	944.5184	944.5179	0.57	0	59	7.6e-05	1	U	K.LDETGVALK.V
2158	309 – 317	540.7930	1079.5715	1079.5723	-0.80	0	38	0.0014	1	U	K.VIHELANER.W
2162	309 – 317	360.8647	1079.5722	1079.5723	-0.11	0	24	0.023	1	U	K.VIHELANER.W
2163	309 – 317	360.8648	1079.5725	1079.5723	0.12	0	39	0.006	1	U	K.VIHELANER.W
2164	309 – 317	360.8648	1079.5726	1079.5723	0.23	0	26	0.031	1	U	K.VIHELANER.W
2167	309 – 317	540.7936	1079.5727	1079.5723	0.39	0	23	0.024	1	U	K.VIHELANER.W
2168	309 – 317	540.7937	1079.5728	1079.5723	0.45	0	58	7.4e-05	1	U	K.VIHELANER.W
2170	309 – 317	360.8650	1079.5733	1079.5723	0.87	0	21	0.048	1	U	K.VIHELANER.W
2171	309 – 317	540.7940	1079.5735	1079.5723	1.09	0	53	0.00027	1	U	K.VIHELANER.W
3582	318 – 327	625.8081	1249.6016	1249.6013	0.30	0	57	1.9e-05	1	U	R.WDVCLTLSEK.G
6862	328 – 342	547.6250	1639.8533	1639.8570	-2.23	0	45	6.5e-05	1	U	K.GFQQISFVNSIATTK.G
6863	328 – 342	820.9342	1639.8539	1639.8570	-1.87	0	53	2.4e-05	1	U	K.GFQQISFVNSIATTK.G
6864	328 – 342	820.9343	1639.8541	1639.8570	-1.74	0	47	7.4e-05	1	U	K.GFQQISFVNSIATTK.G
6865	328 – 342	547.6255	1639.8548	1639.8570	-1.35	0	21	0.035	1	U	K.GFQQISFVNSIATTK.G
6867	328 – 342	820.9352	1639.8558	1639.8570	-0.70	0	34	0.00065	1	U	K.GFQQISFVNSIATTK.G
6868	328 – 342	820.9352	1639.8558	1639.8570	-0.69	0	38	0.00032	1	U	K.GFQQISFVNSIATTK.G
6869	328 – 342	820.9352	1639.8559	1639.8570	-0.63	0	28	0.013	1	U	K.GFQQISFVNSIATTK.G
6870	328 – 342	820.9353	1639.8560	1639.8570	-0.59	0	33	0.0043	1	U	K.GFQQISFVNSIATTK.G
6871	328 – 342	820.9354	1639.8563	1639.8570	-0.40	0	50	3.7e-05	1	U	K.GFQQISFVNSIATTK.G
6872	328 – 342	547.6261	1639.8564	1639.8570	-0.36	0	44	0.0001	1	U	K.GFQQISFVNSIATTK.G
6873	328 – 342	820.9355	1639.8564	1639.8570	-0.36	0	42	0.0048	1	U	K.GFQQISFVNSIATTK.G
6874	328 – 342	820.9356	1639.8565	1639.8570	-0.26	0	43	0.0043	1	U	K.GFQQISFVNSIATTK.G
6876	328 – 342	820.9356	1639.8567	1639.8570	-0.17	0	35	0.0065	1	U	K.GFQQISFVNSIATTK.G
6877	328 – 342	820.9357	1639.8568	1639.8570	-0.13	0	20	0.013	1	U	K.GFQQISFVNSIATTK.G
6878	328 – 342	820.9357	1639.8568	1639.8570	-0.12	0	60	5.9e-05	1	U	K.GFQQISFVNSIATTK.G
6879	328 – 342	820.9357	1639.8568	1639.8570	-0.10	0	54	8.6e-05	1	U	K.GFQQISFVNSIATTK.G
6880	328 – 342	820.9358	1639.8569	1639.8570	-0.019	0	17	0.025	1	U	K.GFQQISFVNSIATTK.G
6881	328 – 342	820.9358	1639.8569	1639.8570	-0.019	0	44	0.00034	1	U	K.GFQQISFVNSIATTK.G
6882	328 – 342	547.6263	1639.8570	1639.8570	-0.016	0	37	0.00074	1	U	K.GFQQISFVNSIATTK.G
6884	328 – 342	547.6264	1639.8573	1639.8570	0.20	0	35	0.00074	1	U	K.GFQQISFVNSIATTK.G
6885	328 – 342	547.6264	1639.8573	1639.8570	0.22	0	29	0.0028	1	U	K.GFQQISFVNSIATTK.G
6886	328 – 342	547.6264	1639.8574	1639.8570	0.24	0	60	1.8e-05	1	U	K.GFQQISFVNSIATTK.G
6888	328 – 342	820.9360	1639.8575	1639.8570	0.30	0	49	0.00048	1	U	K.GFQQISFVNSIATTK.G
6890	328 – 342	820.9360	1639.8575	1639.8570	0.35	0	71	5.3e-06	1	U	K.GFQQISFVNSIATTK.G
6892	328 – 342	820.9361	1639.8576	1639.8570	0.41	0	62	3.2e-06	1	U	K.GFQQISFVNSIATTK.G
6893	328 – 342	820.9361	1639.8577	1639.8570	0.43	0	51	0.00039	1	U	K.GFQQISFVNSIATTK.G
6895	328 – 342	820.9362	1639.8578	1639.8570	0.49	0	35	0.0014	1	U	K.GFQQISFVNSIATTK.G
6896	328 – 342	820.9362	1639.8578	1639.8570	0.53	0	33	0.021	1	U	K.GFQQISFVNSIATTK.G
6897	328 – 342	820.9363	1639.8580	1639.8570	0.63	0	53	2.4e-05	1	U	K.GFQQISFVNSIATTK.G
6898	328 – 342	820.9364	1639.8581	1639.8570	0.71	0	43	0.00098	1	U	K.GFQQISFVNSIATTK.G
6899	328 – 342	547.6267	1639.8582	1639.8570	0.77	0	30	0.0056	1	U	K.GFQQISFVNSIATTK.G
6900	328 – 342	820.9364	1639.8583	1639.8570	0.79	0	57	0.00011	1	U	K.GFQQISFVNSIATTK.G
6901	328 – 342	820.9368	1639.8589	1639.8570	1.20	0	57	3.1e-05	1	U	K.GFQQISFVNSIATTK.G
6902	328 – 342	820.9370	1639.8594	1639.8570	1.48	0	41	0.0057	1	U	K.GFQQISFVNSIATTK.G
6903	328 – 342	820.9370	1639.8594	1639.8570	1.49	0	15	0.036	1	U	K.GFQQISFVNSIATTK.G
6905	328 – 342	820.9376	1639.8607	1639.8570	2.26	0	72	1.3e-06	1	U	K.GFQQISFVNSIATTK.G
4396	346 – 357	679.3583	1356.7021	1356.7038	-1.27	0	70	3.9e-07	1	U	R.HVDYVVDQVVGK.L
4397	346 – 357	679.3588	1356.7030	1356.7038	-0.55	0	49	0.00012	1	U	R.HVDYVVDQVVGK.L
4398	346 – 357	679.3588	1356.7030	1356.7038	-0.55	0	39	0.00065	1	U	R.HVDYVVDQVVGK.L
4400	346 – 357	679.3589	1356.7032	1356.7038	-0.40	0	81	6e-08	1	U	R.HVDYVVDQVVGK.L
4403	346 – 357	679.3590	1356.7034	1356.7038	-0.30	0	76	2e-07	1	U	R.HVDYVVDQVVGK.L
4406	346 – 357	679.3592	1356.7039	1356.7038	0.068	0	83	9e-08	1	U	R.HVDYVVDQVVGK.L
4407	346 – 357	679.3593	1356.7040	1356.7038	0.14	0	56	2.2e-05	1	U	R.HVDYVVDQVVGK.L
4408	346 – 357	679.3593	1356.7040	1356.7038	0.14	0	39	0.00076	1	U	R.HVDYVVDQVVGK.L
4409	346 – 357	679.3593	1356.7040	1356.7038	0.16	0	66	1.1e-06	1	U	R.HVDYVVDQVVGK.L
4410	346 – 357	679.3593	1356.7041	1356.7038	0.24	0	44	0.00019	1	U	R.HVDYVVDQVVGK.L
4411	346 – 357	679.3594	1356.7043	1356.7038	0.35	0	56	8.4e-06	1	U	R.HVDYVVDQVVGK.L
4412	346 – 357	679.3594	1356.7043	1356.7038	0.41	0	72	4.3e-07	1	U	R.HVDYVVDQVVGK.L
4413	346 – 357	679.3594	1356.7043	1356.7038	0.41	0	50	5.4e-05	1	U	R.HVDYVVDQVVGK.L
4414	346 – 357	679.3595	1356.7045	1356.7038	0.52	0	62	3.2e-06	1	U	R.HVDYVVDQVVGK.L
4415	346 – 357	453.2423	1356.7050	1356.7038	0.87	0	34	0.022	1	U	R.HVDYVVDQVVGK.L
525	358 – 364	414.7814	827.5482	827.5480	0.23	1	25	0.024	1	U	K.LIEVVKK.K
2886	368 – 378	580.3447	1158.6749	1158.6761	-1.07	0	47	0.00047	1	U	K.AGVSVKPFQVK.N
2887	368 – 378	580.3448	1158.6751	1158.6761	-0.86	0	55	6.8e-05	1	U	K.AGVSVKPFQVK.N
2888	368 – 378	387.2324	1158.6754	1158.6761	-0.60	0	35	0.0078	1	U	K.AGVSVKPFQVK.N
2890	368 – 378	387.2326	1158.6759	1158.6761	-0.14	0	50	0.00027	1	U	K.AGVSVKPFQVK.N
2891	368 – 378	580.3453	1158.6761	1158.6761	-0.029	0	52	0.00018	1	U	K.AGVSVKPFQVK.N
2893	368 – 378	387.2327	1158.6762	1158.6761	0.043	0	27	0.024	1	U	K.AGVSVKPFQVK.N
2895	368 – 378	580.3455	1158.6765	1158.6761	0.33	0	31	0.0085	1	U	K.AGVSVKPFQVK.N
2899	368 – 378	580.3460	1158.6774	1158.6761	1.13	0	26	0.019	1	U	K.AGVSVKPFQVK.N
11046	379 – 399	859.4235	2575.2488	2575.2529	-1.61	0	42	0.01	1	U	K.NHIWVFINCLIENPTFDSQTK.E
11047	379 – 399	859.4246	2575.2521	2575.2529	-0.33	0	48	0.0013	1	U	K.NHIWVFINCLIENPTFDSQTK.E
2141	438 – 446	539.3062	1076.5979	1076.5978	0.059	1	26	0.008	1	U	K.FKAQTQLNK.K
411	440 – 446	401.7248	801.4351	801.4344	0.87	0	38	0.0074	1	U	K.AQTQLNK.K
1063	440 – 447	465.7720	929.5294	929.5294	-0.017	1	36	0.012	1	U	K.AQTQLNKK.C
4507	458 – 471	685.8488	1369.6830	1369.6837	-0.54	1	53	5.5e-05	1	U	K.GIPKLDDANDAGGK.H
1342	462 – 471	488.2225	974.4305	974.4305	-0.029	0	58	8.1e-05	1	U	K.LDDANDAGGK.H
1343	462 – 471	488.2226	974.4306	974.4305	0.14	0	53	0.00016	1	U	K.LDDANDAGGK.H
1344	462 – 471	488.2226	974.4307	974.4305	0.22	0	23	0.013	1	U	K.LDDANDAGGK.H
1345	462 – 471	488.2229	974.4312	974.4305	0.73	0	27	0.015	1	U	K.LDDANDAGGK.H
7953	472 – 487	591.9612	1772.8618	1772.8614	0.22	0	30	0.015	1	U	K.HSLECTLILTEGDSAK.S
7954	472 – 487	887.4382	1772.8619	1772.8614	0.26	0	100	6.8e-10	1	U	K.HSLECTLILTEGDSAK.S
2583	488 – 499	564.8401	1127.6657	1127.6663	-0.52	0	49	9.7e-05	1	U	K.SLAVSGLGVIGR.D
2584	488 – 499	564.8402	1127.6659	1127.6663	-0.33	0	43	0.00071	1	U	K.SLAVSGLGVIGR.D
2585	488 – 499	564.8402	1127.6659	1127.6663	-0.33	0	54	4.4e-05	1	U	K.SLAVSGLGVIGR.D

Query	Start – End	Observed	Mr(expt)	Mr(calc)	ppm	M	Score	Expect	Rank	U	Peptide
2586	488 – 499	564.8402	1127.6659	1127.6663	-0.31	0	67	4.4e-06	1	U	K.SLAVSGLGVIGR.D
2587	488 – 499	564.8403	1127.6660	1127.6663	-0.21	0	58	7.7e-06	1	U	K.SLAVSGLGVIGR.D
2588	488 – 499	564.8403	1127.6660	1127.6663	-0.19	0	53	0.00011	1	U	K.SLAVSGLGVIGR.D
2589	488 – 499	564.8403	1127.6661	1127.6663	-0.17	0	31	0.0041	1	U	K.SLAVSGLGVIGR.D
2590	488 – 499	564.8403	1127.6661	1127.6663	-0.17	0	38	0.00027	1	U	K.SLAVSGLGVIGR.D
2592	488 – 499	564.8404	1127.6662	1127.6663	-0.064	0	61	2.2e-05	1	U	K.SLAVSGLGVIGR.D
2593	488 – 499	564.8405	1127.6665	1127.6663	0.18	0	47	0.00021	1	U	K.SLAVSGLGVIGR.D
2594	488 – 499	564.8405	1127.6665	1127.6663	0.22	0	65	7.8e-06	1	U	K.SLAVSGLGVIGR.D
2595	488 – 499	564.8405	1127.6665	1127.6663	0.24	0	57	3.4e-05	1	U	K.SLAVSGLGVIGR.D
2596	488 – 499	564.8405	1127.6665	1127.6663	0.24	0	41	0.00058	1	U	K.SLAVSGLGVIGR.D
2597	488 – 499	564.8406	1127.6667	1127.6663	0.42	0	53	7.5e-05	1	U	K.SLAVSGLGVIGR.D
2598	488 – 499	564.8406	1127.6667	1127.6663	0.42	0	50	6.3e-05	1	U	K.SLAVSGLGVIGR.D
2599	488 – 499	564.8406	1127.6667	1127.6663	0.43	0	41	0.0017	1	U	K.SLAVSGLGVIGR.D
2600	488 – 499	564.8407	1127.6669	1127.6663	0.54	0	69	2.4e-06	1	U	K.SLAVSGLGVIGR.D
2601	488 – 499	376.8963	1127.6671	1127.6663	0.72	0	37	0.0034	1	U	K.SLAVSGLGVIGR.D
2602	488 – 499	564.8410	1127.6675	1127.6663	1.11	0	64	8.2e-06	1	U	K.SLAVSGLGVIGR.D
2603	488 – 499	564.8411	1127.6677	1127.6663	1.30	0	43	0.00021	1	U	K.SLAVSGLGVIGR.D
2604	488 – 499	564.8412	1127.6678	1127.6663	1.41	0	69	4e-06	1	U	K.SLAVSGLGVIGR.D
2531	500 – 508	561.8063	1121.5981	1121.5982	-0.095	1	33	0.013	1	U	R.DRYGVFPLR.G
638	502 – 508	426.2421	850.4696	850.4701	-0.61	0	30	0.0072	1		R.YGVFPLR.G
642	502 – 508	426.2425	850.4705	850.4701	0.48	0	17	0.038	1		R.YGVFPLR.G
643	502 – 508	426.2426	850.4707	850.4701	0.66	0	30	0.013	1		R.YGVFPLR.G
5938	521 – 533	765.4045	1528.7945	1528.7919	1.74	0	96	1.7e-08	1		K.QIMENAEINNIIK.I
5939	521 – 533	510.6058	1528.7955	1528.7919	2.36	0	49	0.0011	1		K.QIMENAEINNIIK.I
6130	521 – 533	773.4007	1544.7869	1544.7868	0.090	0	58	2.3e-05	1		K.QIMENAEINNIIK.I + Oxidation (M)
6131	521 – 533	773.4008	1544.7871	1544.7868	0.19	0	50	0.00039	1		K.QIMENAEINNIIK.I + Oxidation (M)
6132	521 – 533	773.4012	1544.7879	1544.7868	0.70	0	62	1.5e-05	1		K.QIMENAEINNIIK.I + Oxidation (M)
6133	521 – 533	773.4012	1544.7879	1544.7868	0.72	0	71	3.5e-06	1		K.QIMENAEINNIIK.I + Oxidation (M)
483	534 – 540	410.7503	819.4860	819.4854	0.71	0	21	0.043	1		K.IVGLQYK.K
484	534 – 540	410.7504	819.4862	819.4854	0.95	0	45	0.00038	1		K.IVGLQYK.K
2850	541 – 550	385.8556	1154.5449	1154.5455	-0.56	1	24	0.02	1	U	K.KSYDDAESLK.T
2851	541 – 550	578.2799	1154.5453	1154.5455	-0.20	1	36	0.0012	1	U	K.KSYDDAESLK.T
2853	541 – 550	578.2802	1154.5458	1154.5455	0.28	1	21	0.039	1	U	K.KSYDDAESLK.T
2854	541 – 550	578.2808	1154.5470	1154.5455	1.32	1	34	0.0018	1	U	K.KSYDDAESLK.T
1714	542 – 550	514.2325	1026.4504	1026.4505	-0.17	0	25	0.0081	1	U	K.SYDDAESLK.T
1715	542 – 550	514.2326	1026.4506	1026.4505	0.060	0	39	0.00075	1	U	K.SYDDAESLK.T
1716	542 – 550	514.2326	1026.4507	1026.4505	0.20	0	45	0.00017	1	U	K.SYDDAESLK.T
1717	542 – 550	514.2328	1026.4511	1026.4505	0.57	0	27	0.012	1	U	K.SYDDAESLK.T
4691	542 – 553	699.3495	1396.6844	1396.6834	0.70	1	60	1.4e-05	1	U	K.SYDDAESLKTLR.Y
7623	557 – 571	866.4054	1730.7963	1730.7968	-0.28	0	87	7.3e-08	1		K.IMIMTDQDQDGSHIK.G
7629	557 – 571	866.4058	1730.7971	1730.7968	0.18	0	70	2.2e-06	1		K.IMIMTDQDQDGSHIK.G
7630	557 – 571	577.9397	1730.7974	1730.7968	0.37	0	35	0.0059	1		K.IMIMTDQDQDGSHIK.G
7631	557 – 571	866.4061	1730.7976	1730.7968	0.52	0	75	2.1e-06	1		K.IMIMTDQDQDGSHIK.G
7633	557 – 571	866.4067	1730.7989	1730.7968	1.22	0	59	0.00012	1		K.IMIMTDQDQDGSHIK.G
7751	557 – 571	583.2706	1746.7900	1746.7917	-0.96	0	48	0.0017	1		K.IMIMTDQDQDGSHIK.G + Oxidation (M)
7752	557 – 571	583.2707	1746.7903	1746.7917	-0.80	0	28	0.02	1		K.IMIMTDQDQDGSHIK.G + Oxidation (M)
7754	557 – 571	874.4026	1746.7906	1746.7917	-0.62	0	64	4e-05	1		K.IMIMTDQDQDGSHIK.G + Oxidation (M)
7755	557 – 571	874.4027	1746.7908	1746.7917	-0.49	0	68	2.2e-06	1		K.IMIMTDQDQDGSHIK.G + Oxidation (M)
7756	557 – 571	874.4027	1746.7909	1746.7917	-0.45	0	91	1.4e-08	1		K.IMIMTDQDQDGSHIK.G + Oxidation (M)
7757	557 – 571	583.2710	1746.7912	1746.7917	-0.29	0	28	0.01	1		K.IMIMTDQDQDGSHIK.G + Oxidation (M)
7760	557 – 571	583.2711	1746.7915	1746.7917	-0.097	0	43	0.0016	1		K.IMIMTDQDQDGSHIK.G + Oxidation (M)
7761	557 – 571	874.4033	1746.7920	1746.7917	0.19	0	71	9.3e-06	1		K.IMIMTDQDQDGSHIK.G + Oxidation (M)
7762	557 – 571	583.2713	1746.7920	1746.7917	0.19	0	23	0.023	1		K.IMIMTDQDQDGSHIK.G + Oxidation (M)
7763	557 – 571	583.2713	1746.7921	1746.7917	0.26	0	34	0.045	1		K.IMIMTDQDQDGSHIK.G + Oxidation (M)
7764	557 – 571	874.4034	1746.7922	1746.7917	0.28	0	72	7.4e-06	1		K.IMIMTDQDQDGSHIK.G + Oxidation (M)
7765	557 – 571	583.2714	1746.7924	1746.7917	0.42	0	49	0.0013	1		K.IMIMTDQDQDGSHIK.G + Oxidation (M)
7766	557 – 571	583.2715	1746.7926	1746.7917	0.50	0	51	0.00052	1		K.IMIMTDQDQDGSHIK.G + Oxidation (M)
7767	557 – 571	583.2719	1746.7939	1746.7917	1.29	0	26	0.0099	1		K.IMIMTDQDQDGSHIK.G + Oxidation (M)
7870	557 – 571	588.6019	1762.7840	1762.7866	-1.47	0	29	0.011	1		K.IMIMTDQDQDGSHIK.G + 2 Oxidation (M)
7871	557 – 571	588.6021	1762.7845	1762.7866	-1.19	0	50	0.00052	1		K.IMIMTDQDQDGSHIK.G + 2 Oxidation (M)
7873	557 – 571	588.6023	1762.7852	1762.7866	-0.81	0	51	0.00012	1		K.IMIMTDQDQDGSHIK.G + 2 Oxidation (M)
7874	557 – 571	588.6024	1762.7853	1762.7866	-0.76	0	38	0.0046	1		K.IMIMTDQDQDGSHIK.G + 2 Oxidation (M)
7877	557 – 571	882.4004	1762.7862	1762.7866	-0.23	0	73	5.7e-06	1		K.IMIMTDQDQDGSHIK.G + 2 Oxidation (M)
7878	557 – 571	882.4004	1762.7863	1762.7866	-0.17	0	41	0.0012	1		K.IMIMTDQDQDGSHIK.G + 2 Oxidation (M)
7879	557 – 571	882.4004	1762.7863	1762.7866	-0.15	0	71	9.5e-06	1		K.IMIMTDQDQDGSHIK.G + 2 Oxidation (M)
7880	557 – 571	588.6027	1762.7864	1762.7866	-0.13	0	24	0.0096	1		K.IMIMTDQDQDGSHIK.G + 2 Oxidation (M)
7881	557 – 571	882.4005	1762.7864	1762.7866	-0.12	0	62	5e-05	1		K.IMIMTDQDQDGSHIK.G + 2 Oxidation (M)
7882	557 – 571	588.6028	1762.7864	1762.7866	-0.096	0	58	0.00021	1		K.IMIMTDQDQDGSHIK.G

Query	Start – End	Observed	Mr(expt)	Mr(calc)	ppm	M	Score	Expect	Rank	U	Peptide
											+ 2 Oxidation (M)
7883	557 – 571	882.4006	1762.7866	1762.7866	-0.0017	0	70	1.2e-05	1		K.IMIMTDQDQDGSHIK.G + 2 Oxidation (M)
7884	557 – 571	588.6028	1762.7866	1762.7866	0.023	0	44	0.0016	1		K.IMIMTDQDQDGSHIK.G + 2 Oxidation (M)
7886	557 – 571	882.4007	1762.7869	1762.7866	0.16	0	62	7.3e-05	1		K.IMIMTDQDQDGSHIK.G + 2 Oxidation (M)
7887	557 – 571	588.6030	1762.7870	1762.7866	0.24	0	21	0.031	1		K.IMIMTDQDQDGSHIK.G + 2 Oxidation (M)
7888	557 – 571	588.6031	1762.7876	1762.7866	0.55	0	28	0.013	1		K.IMIMTDQDQDGSHIK.G + 2 Oxidation (M)
7890	557 – 571	882.4025	1762.7904	1762.7866	2.18	0	62	4.1e-05	1		K.IMIMTDQDQDGSHIK.G + 2 Oxidation (M)
8870	572 – 587	634.6956	1901.0649	1901.0676	-1.37	0	20	0.015	1	U	K.GLLINFIHHNWPSLLK.H
8874	572 – 587	951.5410	1901.0674	1901.0676	-0.054	0	41	0.00013	1	U	K.GLLINFIHHNWPSLLK.H
8875	572 – 587	951.5411	1901.0676	1901.0676	0.030	0	52	1.4e-05	1	U	K.GLLINFIHHNWPSLLK.H
8878	572 – 587	634.6966	1901.0681	1901.0676	0.28	0	27	0.018	1	U	K.GLLINFIHHNWPSLLK.H
8879	572 – 587	634.6967	1901.0682	1901.0676	0.32	0	18	0.028	1	U	K.GLLINFIHHNWPSLLK.H
5940	588 – 600	765.4198	1528.8251	1528.8290	-2.51	0	71	1.9e-06	1	U	K.HGFLEEFITPIVK.A
5941	588 – 600	510.6158	1528.8254	1528.8290	-2.32	0	38	0.0095	1	U	K.HGFLEEFITPIVK.A
5942	588 – 600	765.4210	1528.8274	1528.8290	-0.99	0	58	3.5e-06	1	U	K.HGFLEEFITPIVK.A
5944	588 – 600	765.4211	1528.8276	1528.8290	-0.90	0	70	1e-06	1	U	K.HGFLEEFITPIVK.A
5945	588 – 600	765.4212	1528.8279	1528.8290	-0.70	0	71	2.3e-07	1	U	K.HGFLEEFITPIVK.A
5947	588 – 600	510.6167	1528.8282	1528.8290	-0.47	0	28	0.026	1	U	K.HGFLEEFITPIVK.A
5948	588 – 600	510.6168	1528.8286	1528.8290	-0.26	0	46	0.0017	1	U	K.HGFLEEFITPIVK.A
5949	588 – 600	765.4216	1528.8286	1528.8290	-0.22	0	66	6.7e-07	1	U	K.HGFLEEFITPIVK.A
5950	588 – 600	510.6168	1528.8286	1528.8290	-0.22	0	44	0.0026	1	U	K.HGFLEEFITPIVK.A
5952	588 – 600	510.6168	1528.8287	1528.8290	-0.20	0	43	0.0032	1	U	K.HGFLEEFITPIVK.A
5953	588 – 600	765.4217	1528.8288	1528.8290	-0.12	0	64	9.1e-07	1	U	K.HGFLEEFITPIVK.A
5954	588 – 600	510.6169	1528.8288	1528.8290	-0.099	0	34	0.0019	1	U	K.HGFLEEFITPIVK.A
5955	588 – 600	510.6169	1528.8288	1528.8290	-0.080	0	30	0.0096	1	U	K.HGFLEEFITPIVK.A
5956	588 – 600	765.4217	1528.8289	1528.8290	-0.063	0	90	5.9e-09	1	U	K.HGFLEEFITPIVK.A
5957	588 – 600	510.6169	1528.8290	1528.8290	-0.0013	0	38	0.003	1	U	K.HGFLEEFITPIVK.A
5958	588 – 600	765.4218	1528.8290	1528.8290	0.016	0	76	8.2e-08	1	U	K.HGFLEEFITPIVK.A
5959	588 – 600	765.4218	1528.8291	1528.8290	0.081	0	38	0.0013	1	U	K.HGFLEEFITPIVK.A
5960	588 – 600	765.4218	1528.8291	1528.8290	0.081	0	58	1.3e-05	1	U	K.HGFLEEFITPIVK.A
5961	588 – 600	765.4218	1528.8291	1528.8290	0.094	0	56	1.5e-05	1	U	K.HGFLEEFITPIVK.A
5962	588 – 600	765.4218	1528.8291	1528.8290	0.094	0	84	4.3e-08	1	U	K.HGFLEEFITPIVK.A
5963	588 – 600	765.4218	1528.8291	1528.8290	0.11	0	24	0.02	1	U	K.HGFLEEFITPIVK.A
5964	588 – 600	510.6170	1528.8292	1528.8290	0.16	0	44	0.0023	1	U	K.HGFLEEFITPIVK.A
5965	588 – 600	765.4219	1528.8292	1528.8290	0.16	0	87	1.3e-08	1	U	K.HGFLEEFITPIVK.A
5967	588 – 600	765.4219	1528.8293	1528.8290	0.23	0	20	0.012	1	U	K.HGFLEEFITPIVK.A
5968	588 – 600	510.6171	1528.8295	1528.8290	0.33	0	22	0.025	1	U	K.HGFLEEFITPIVK.A
5969	588 – 600	765.4220	1528.8295	1528.8290	0.34	0	65	8.4e-07	1	U	K.HGFLEEFITPIVK.A
5970	588 – 600	510.6171	1528.8295	1528.8290	0.35	0	26	0.039	1	U	K.HGFLEEFITPIVK.A
5971	588 – 600	765.4221	1528.8296	1528.8290	0.41	0	46	0.00022	1	U	K.HGFLEEFITPIVK.A
5972	588 – 600	765.4221	1528.8296	1528.8290	0.45	0	68	6.9e-07	1	U	K.HGFLEEFITPIVK.A
5973	588 – 600	765.4221	1528.8297	1528.8290	0.49	0	40	0.00035	1	U	K.HGFLEEFITPIVK.A
5975	588 – 600	765.4222	1528.8299	1528.8290	0.64	0	56	6e-06	1	U	K.HGFLEEFITPIVK.A
5976	588 – 600	765.4225	1528.8305	1528.8290	1.00	0	22	0.025	1	U	K.HGFLEEFITPIVK.A
9880	604 – 620	1080.5171	2159.0196	2159.0211	-0.69	1	102	7.1e-09	1	U	K.NKQELSFYSIPEFDEWK.K
9881	604 – 620	1080.5172	2159.0199	2159.0211	-0.57	1	87	1.2e-07	1	U	K.NKQELSFYSIPEFDEWK.K
9882	604 – 620	720.6813	2159.0220	2159.0211	0.41	1	26	0.039	1	U	K.NKQELSFYSIPEFDEWK.K
10288	604 – 621	572.7859	2287.1146	2287.1161	-0.65	2	25	0.045	1	U	K.NKQELSFYSIPEFDEWKK.H
10289	604 – 621	1144.5649	2287.1152	2287.1161	-0.36	2	67	6.3e-07	1	U	K.NKQELSFYSIPEFDEWKK.H
10290	604 – 621	763.3794	2287.1163	2287.1161	0.085	2	44	0.00074	1	U	K.NKQELSFYSIPEFDEWKK.H
8985	606 – 620	959.4480	1916.8814	1916.8832	-0.97	0	58	1.6e-05	1	U	K.QELSFYSIPEFDEWK.K
8987	606 – 620	959.4498	1916.8850	1916.8832	0.92	0	38	0.0019	1	U	K.QELSFYSIPEFDEWK.K
9496	606 – 621	1023.4963	2044.9781	2044.9782	-0.045	1	38	0.0008	1	U	K.QELSFYSIPEFDEWKK.H
9497	606 – 621	682.6668	2044.9786	2044.9782	0.19	1	39	0.0026	1	U	K.QELSFYSIPEFDEWKK.H
1986	647 – 654	530.7240	1059.4334	1059.4331	0.25	0	18	0.027	1	U	K.EYFADMER.H
1988	647 – 654	530.7240	1059.4335	1059.4331	0.40	0	33	0.0053	1	U	K.EYFADMER.H
2127	647 – 654	538.7210	1075.4273	1075.4280	-0.64	0	17	0.034	1	U	K.EYFADMER.H + Oxidation (M)
2128	647 – 654	538.7211	1075.4277	1075.4280	-0.35	0	26	0.015	1	U	K.EYFADMER.H + Oxidation (M)
2130	647 – 654	538.7213	1075.4280	1075.4280	-0.048	0	33	0.0075	1	U	K.EYFADMER.H + Oxidation (M)
7075	661 – 676	834.9068	1667.7989	1667.8042	-3.18	0	77	1.9e-07	1	U	R.YAGPEDDAAITLAFSK.K
7076	661 – 676	834.9089	1667.8032	1667.8042	-0.60	0	68	1.1e-06	1	U	R.YAGPEDDAAITLAFSK.K
7077	661 – 676	834.9089	1667.8032	1667.8042	-0.60	0	59	1.5e-05	1	U	R.YAGPEDDAAITLAFSK.K
7078	661 – 676	834.9089	1667.8033	1667.8042	-0.59	0	79	7.3e-08	1	U	R.YAGPEDDAAITLAFSK.K
7079	661 – 676	834.9090	1667.8034	1667.8042	-0.49	0	80	3e-08	1	U	R.YAGPEDDAAITLAFSK.K
7080	661 – 676	834.9091	1667.8037	1667.8042	-0.33	0	57	9.3e-06	1	U	R.YAGPEDDAAITLAFSK.K
7081	661 – 676	834.9092	1667.8039	1667.8042	-0.18	0	74	2.3e-07	1	U	R.YAGPEDDAAITLAFSK.K
7082	661 – 676	834.9094	1667.8042	1667.8042	-0.046	0	71	2.2e-07	1	U	R.YAGPEDDAAITLAFSK.K
7083	661 – 676	834.9094	1667.8042	1667.8042	-0.022	0	91	5.6e-09	1	U	R.YAGPEDDAAITLAFSK.K
7084	661 – 676	834.9094	1667.8043	1667.8042	0.050	0	52	1.7e-05	1	U	R.YAGPEDDAAITLAFSK.K
7085	661 – 676	834.9095	1667.8044	1667.8042	0.098	0	71	2.1e-07	1	U	R.YAGPEDDAAITLAFSK.K
7086	661 – 676	834.9096	1667.8046	1667.8042	0.21	0	64	4.4e-06	1	U	R.YAGPEDDAAITLAFSK.K
7087	661 – 676	834.9096	1667.8047	1667.8042	0.25	0	74	1.2e-07	1	U	R.YAGPEDDAAITLAFSK.K
7088	661 – 676	834.9096	1667.8047	1667.8042	0.28	0	86	3.3e-08	1	U	R.YAGPEDDAAITLAFSK.K
7089	661 – 676	834.9097	1667.8048	1667.8042	0.33	0	90	1.9e-08	1	U	R.YAGPEDDAAITLAFSK.K
7090	661 – 676	834.9097	1667.8048	1667.8042	0.35	0	74	1.2e-07	1	U	R.YAGPEDDAAITLAFSK.K
7091	661 – 676	834.9097	1667.8049	1667.8042	0.40	0	73	1.5e-07	1	U	R.YAGPEDDAAITLAFSK.K
7092	661 – 676	556.9423	1667.8052	1667.8042	0.57	0	45	0.00045	1	U	R.YAGPEDDAAITLAFSK.K
7093	661 – 676	834.9099	1667.8052	1667.8042	0.58	0	80	1.4e-07	1	U	R.YAGPEDDAAITLAFSK.K
7094	661 – 676	834.9100	1667.8055	1667.8042	0.76	0	83	5.7e-08	1	U	R.YAGPEDDAAITLAFSK.K
7095	661 – 676	834.9101	1667.8056	1667.8042	0.81	0	74	5.7e-07	1	U	R.YAGPEDDAAITLAFSK.K
7096	661 – 676	834.9101	1667.8057	1667.8042	0.85	0	88	6.1e-09	1	U	R.YAGPEDDAAITLAFSK.K
7097	661 – 676	834.9104	1667.8062	1667.8042	1.20	0	62	1.7e-06	1	U	R.YAGPEDDAAITLAFSK.K
7098	661 – 676	834.9107	1667.8068	1667.8042	1.51	0	70	5.7e-07	1	U	R.YAGPEDDAAITLAFSK.K
7099	661 – 676	834.9113	1667.8080	1667.8042	2.24	0	63	1.3e-06	1	U	R.YAGPEDDAAITLAFSK.K
7100	661 – 676	834.9114	1667.8082	1667.8042	2.39	0	73	3.4e-06	1	U	R.YAGPEDDAAITLAFSK.K

Query	Start – End	Observed	Mr(expt)	Mr(calc)	ppm	M	Score	Expect	Rank	U	Peptide
7101	661 – 676	834.9115	1667.8084	1667.8042	2.52	0	58	3.7e-06	1	U	R.YAGPEDDAAITLAFSK.K
5267	683 – 693	734.8459	1467.6773	1467.6816	-2.97	1	23	0.012	1		R.KEWLTNFMEDR.R
5270	683 – 693	490.2346	1467.6819	1467.6816	0.22	1	18	0.049	1		R.KEWLTNFMEDR.R
5396	683 – 693	495.5660	1483.6761	1483.6765	-0.31	1	28	0.011	1		R.KEWLTNFMEDR.R + Oxidation (M)
5397	683 – 693	742.8453	1483.6761	1483.6765	-0.29	1	27	0.0048	1		R.KEWLTNFMEDR.R + Oxidation (M)
5398	683 – 693	742.8456	1483.6766	1483.6765	0.016	1	27	0.0083	1		R.KEWLTNFMEDR.R + Oxidation (M)
5399	683 – 693	742.8458	1483.6770	1483.6765	0.33	1	49	0.00018	1		R.KEWLTNFMEDR.R + Oxidation (M)
5400	683 – 693	495.5663	1483.6771	1483.6765	0.38	1	26	0.009	1		R.KEWLTNFMEDR.R + Oxidation (M)
5401	683 – 693	495.5664	1483.6772	1483.6765	0.46	1	28	0.0059	1		R.KEWLTNFMEDR.R + Oxidation (M)
5402	683 – 693	742.8463	1483.6780	1483.6765	1.01	1	28	0.015	1		R.KEWLTNFMEDR.R + Oxidation (M)
5403	683 – 693	495.5667	1483.6782	1483.6765	1.14	1	30	0.009	1		R.KEWLTNFMEDR.R + Oxidation (M)
5404	683 – 693	742.8466	1483.6785	1483.6765	1.35	1	32	0.0014	1		R.KEWLTNFMEDR.R + Oxidation (M)
5405	683 – 693	495.5673	1483.6800	1483.6765	2.30	1	31	0.022	1		R.KEWLTNFMEDR.R + Oxidation (M)
6784	683 – 694	542.2680	1623.7821	1623.7827	-0.36	2	40	0.0026	1		R.KEWLTNFMEDRR.Q
4305	684 – 693	670.8006	1339.5867	1339.5867	0.043	0	61	2.3e-06	1		K.EWLTNFMEDR.R
4306	684 – 693	670.8009	1339.5872	1339.5867	0.39	0	49	0.0002	1		K.EWLTNFMEDR.R
4388	684 – 693	678.7975	1355.5804	1355.5816	-0.86	0	28	0.011	1		K.EWLTNFMEDR.R + Oxidation (M)
4389	684 – 693	678.7980	1355.5814	1355.5816	-0.10	0	38	0.0053	1		K.EWLTNFMEDR.R + Oxidation (M)
4390	684 – 693	678.7982	1355.5818	1355.5816	0.13	0	38	0.0017	1		K.EWLTNFMEDR.R + Oxidation (M)
4391	684 – 693	678.7982	1355.5818	1355.5816	0.18	0	25	0.026	1		K.EWLTNFMEDR.R + Oxidation (M)
8424	697 – 712	610.9995	1829.9768	1829.9788	-1.12	1	48	0.00049	1	U	R.RLHGLPEQFLYGTATK.H
8425	697 – 712	610.9997	1829.9772	1829.9788	-0.85	1	43	0.00043	1	U	R.RLHGLPEQFLYGTATK.H
8426	697 – 712	610.9998	1829.9777	1829.9788	-0.63	1	58	1.3e-05	1	U	R.RLHGLPEQFLYGTATK.H
8428	697 – 712	915.9963	1829.9780	1829.9788	-0.46	1	83	5.9e-08	1	U	R.RLHGLPEQFLYGTATK.H
8429	697 – 712	611.0000	1829.9780	1829.9788	-0.43	1	57	5.3e-05	1	U	R.RLHGLPEQFLYGTATK.H
8430	697 – 712	915.9964	1829.9782	1829.9788	-0.33	1	78	9.2e-08	1	U	R.RLHGLPEQFLYGTATK.H
8432	697 – 712	611.0003	1829.9792	1829.9788	0.19	1	27	0.0085	1	U	R.RLHGLPEQFLYGTATK.H
8433	697 – 712	611.0004	1829.9793	1829.9788	0.29	1	40	0.001	1	U	R.RLHGLPEQFLYGTATK.H
8435	697 – 712	611.0005	1829.9796	1829.9788	0.42	1	40	0.00046	1	U	R.RLHGLPEQFLYGTATK.H
8436	697 – 712	611.0006	1829.9798	1829.9788	0.56	1	46	0.0004	1	U	R.RLHGLPEQFLYGTATK.H
8437	697 – 712	611.0010	1829.9811	1829.9788	1.24	1	31	0.0049	1	U	R.RLHGLPEQFLYGTATK.H
8438	697 – 712	611.0010	1829.9811	1829.9788	1.24	1	24	0.017	1	U	R.RLHGLPEQFLYGTATK.H
7139	698 – 712	837.9447	1673.8748	1673.8777	-1.74	0	37	0.00031	1	U	R.LHGLPEQFLYGTATK.H
7140	698 – 712	837.9447	1673.8748	1673.8777	-1.73	0	28	0.019	1	U	R.LHGLPEQFLYGTATK.H
7141	698 – 712	837.9447	1673.8749	1673.8777	-1.67	0	22	0.018	1	U	R.LHGLPEQFLYGTATK.H
7142	698 – 712	558.9658	1673.8755	1673.8777	-1.34	0	28	0.015	1	U	R.LHGLPEQFLYGTATK.H
7143	698 – 712	837.9456	1673.8767	1673.8777	-0.61	0	26	0.0048	1	U	R.LHGLPEQFLYGTATK.H
7144	698 – 712	837.9456	1673.8767	1673.8777	-0.59	0	40	0.0002	1	U	R.LHGLPEQFLYGTATK.H
7146	698 – 712	837.9457	1673.8768	1673.8777	-0.55	0	28	0.0024	1	U	R.LHGLPEQFLYGTATK.H
7147	698 – 712	837.9457	1673.8769	1673.8777	-0.47	0	25	0.0042	1	U	R.LHGLPEQFLYGTATK.H
7149	698 – 712	837.9458	1673.8770	1673.8777	-0.41	0	33	0.0015	1	U	R.LHGLPEQFLYGTATK.H
7151	698 – 712	558.9663	1673.8770	1673.8777	-0.41	0	19	0.038	1	U	R.LHGLPEQFLYGTATK.H
7154	698 – 712	837.9459	1673.8772	1673.8777	-0.29	0	25	0.0048	1	U	R.LHGLPEQFLYGTATK.H
7155	698 – 712	837.9460	1673.8773	1673.8777	-0.21	0	54	9.4e-06	1	U	R.LHGLPEQFLYGTATK.H
7156	698 – 712	558.9665	1673.8776	1673.8777	-0.089	0	34	0.0016	1	U	R.LHGLPEQFLYGTATK.H
7158	698 – 712	558.9665	1673.8776	1673.8777	-0.053	0	29	0.0037	1	U	R.LHGLPEQFLYGTATK.H
7160	698 – 712	558.9665	1673.8778	1673.8777	0.036	0	50	6.8e-05	1	U	R.LHGLPEQFLYGTATK.H
7161	698 – 712	837.9462	1673.8778	1673.8777	0.040	0	41	0.00021	1	U	R.LHGLPEQFLYGTATK.H
7162	698 – 712	419.4767	1673.8778	1673.8777	0.045	0	25	0.019	1	U	R.LHGLPEQFLYGTATK.H
7163	698 – 712	558.9666	1673.8779	1673.8777	0.14	0	52	0.00013	1	U	R.LHGLPEQFLYGTATK.H
7164	698 – 712	558.9666	1673.8780	1673.8777	0.18	0	34	0.0038	1	U	R.LHGLPEQFLYGTATK.H
7165	698 – 712	558.9666	1673.8780	1673.8777	0.20	0	20	0.047	1	U	R.LHGLPEQFLYGTATK.H
7166	698 – 712	837.9464	1673.8781	1673.8777	0.27	0	56	6.7e-06	1	U	R.LHGLPEQFLYGTATK.H
7167	698 – 712	558.9667	1673.8782	1673.8777	0.27	0	21	0.046	1	U	R.LHGLPEQFLYGTATK.H
7168	698 – 712	837.9464	1673.8782	1673.8777	0.33	0	41	0.00013	1	U	R.LHGLPEQFLYGTATK.H
7169	698 – 712	837.9464	1673.8783	1673.8777	0.34	0	34	0.00063	1	U	R.LHGLPEQFLYGTATK.H
7170	698 – 712	558.9667	1673.8783	1673.8777	0.34	0	29	0.011	1	U	R.LHGLPEQFLYGTATK.H
7171	698 – 712	837.9464	1673.8783	1673.8777	0.37	0	39	0.00021	1	U	R.LHGLPEQFLYGTATK.H
7172	698 – 712	558.9667	1673.8784	1673.8777	0.39	0	39	0.0048	1	U	R.LHGLPEQFLYGTATK.H
7173	698 – 712	558.9668	1673.8785	1673.8777	0.45	0	44	0.0028	1	U	R.LHGLPEQFLYGTATK.H
7174	698 – 712	558.9668	1673.8785	1673.8777	0.47	0	42	0.00081	1	U	R.LHGLPEQFLYGTATK.H
7176	698 – 712	837.9469	1673.8793	1673.8777	0.96	0	55	7.3e-06	1	U	R.LHGLPEQFLYGTATK.H
7177	698 – 712	558.9671	1673.8794	1673.8777	0.99	0	28	0.027	1	U	R.LHGLPEQFLYGTATK.H
7178	698 – 712	558.9671	1673.8796	1673.8777	1.11	0	33	0.0047	1	U	R.LHGLPEQFLYGTATK.H
3685	713 – 722	632.8183	1263.6220	1263.6248	-2.20	0	15	0.039	1	U	K.HLTYNDFINK.E
3686	713 – 722	632.8190	1263.6235	1263.6248	-1.00	0	28	0.0076	1	U	K.HLTYNDFINK.E
3687	713 – 722	632.8191	1263.6236	1263.6248	-0.95	0	25	0.013	1	U	K.HLTYNDFINK.E
3688	713 – 722	632.8192	1263.6238	1263.6248	-0.76	0	16	0.033	1	U	K.HLTYNDFINK.E
3689	713 – 722	632.8192	1263.6238	1263.6248	-0.75	0	37	0.0096	1	U	K.HLTYNDFINK.E
3690	713 – 722	632.8192	1263.6239	1263.6248	-0.68	0	24	0.011	1	U	K.HLTYNDFINK.E
3691	713 – 722	632.8193	1263.6240	1263.6248	-0.59	0	26	0.018	1	U	K.HLTYNDFINK.E
3692	713 – 722	632.8194	1263.6241	1263.6248	-0.49	0	31	0.0049	1	U	K.HLTYNDFINK.E
3693	713 – 722	632.8195	1263.6244	1263.6248	-0.30	0	31	0.0038	1	U	K.HLTYNDFINK.E
3694	713 – 722	422.2154	1263.6245	1263.6248	-0.20	0	35	0.0052	1	U	K.HLTYNDFINK.E
3695	713 – 722	632.8196	1263.6246	1263.6248	-0.13	0	44	0.003	1	U	K.HLTYNDFINK.E
3696	713 – 722	632.8196	1263.6246	1263.6248	-0.11	0	29	0.026	1	U	K.HLTYNDFINK.E
3697	713 – 722	632.8196	1263.6247	1263.6248	-0.082	0	18	0.036	1	U	K.HLTYNDFINK.E
3699	713 – 722	632.8196	1263.6247	1263.6248	-0.050	0	36	0.0012	1	U	K.HLTYNDFINK.E
3700	713 – 722	632.8196	1263.6247	1263.6248	-0.018	0	32	0.0022	1	U	K.HLTYNDFINK.E
3701	713 – 722	632.8197	1263.6249	1263.6248	0.077	0	37	0.001	1	U	K.HLTYNDFINK.E

Query	Start – End	Observed	Mr(expt)	Mr(calc)	ppm	M	Score	Expect	Rank	U	Peptide
3702	713 – 722	632.8197	1263.6249	1263.6248	0.093	0	32	0.0013	1	U	K.HLTYNDFINK.E
3703	713 – 722	632.8197	1263.6249	1263.6248	0.093	0	25	0.023	1	U	K.HLTYNDFINK.E
3705	713 – 722	632.8198	1263.6249	1263.6248	0.14	0	28	0.0066	1	U	K.HLTYNDFINK.E
3707	713 – 722	632.8198	1263.6250	1263.6248	0.20	0	23	0.0093	1	U	K.HLTYNDFINK.E
3708	713 – 722	632.8198	1263.6251	1263.6248	0.27	0	25	0.02	1	U	K.HLTYNDFINK.E
3709	713 – 722	632.8198	1263.6251	1263.6248	0.28	0	34	0.00072	1	U	K.HLTYNDFINK.E
3710	713 – 722	632.8200	1263.6254	1263.6248	0.52	0	28	0.0098	1	U	K.HLTYNDFINK.E
3711	713 – 722	632.8200	1263.6255	1263.6248	0.55	0	44	0.00022	1	U	K.HLTYNDFINK.E
3712	713 – 722	632.8201	1263.6257	1263.6248	0.74	0	34	0.00086	1	U	K.HLTYNDFINK.E
3713	713 – 722	632.8201	1263.6257	1263.6248	0.77	0	22	0.034	1	U	K.HLTYNDFINK.E
3715	713 – 722	632.8202	1263.6259	1263.6248	0.92	0	22	0.028	1	U	K.HLTYNDFINK.E
3717	713 – 722	632.8205	1263.6265	1263.6248	1.37	0	36	0.0012	1	U	K.HLTYNDFINK.E
11319	713 – 734	894.7795	2681.3166	2681.3085	3.04	1	48	0.00017	1	U	K.HLTYNDFINKELILFSNSDNER.S
4959	723 – 734	718.8534	1435.6921	1435.6943	-1.49	0	25	0.016	1		K.ELILFSNSDNER.S
4960	723 – 734	718.8536	1435.6926	1435.6943	-1.14	0	28	0.048	1		K.ELILFSNSDNER.S
4962	723 – 734	718.8540	1435.6934	1435.6943	-0.63	0	33	0.0084	1		K.ELILFSNSDNER.S
4963	723 – 734	718.8541	1435.6936	1435.6943	-0.45	0	33	0.0093	1		K.ELILFSNSDNER.S
4964	723 – 734	718.8542	1435.6938	1435.6943	-0.31	0	41	0.00047	1		K.ELILFSNSDNER.S
4965	723 – 734	718.8542	1435.6939	1435.6943	-0.24	0	33	0.005	1		K.ELILFSNSDNER.S
4967	723 – 734	718.8544	1435.6942	1435.6943	-0.029	0	45	0.00038	1		K.ELILFSNSDNER.S
4969	723 – 734	718.8545	1435.6945	1435.6943	0.12	0	38	0.0039	1		K.ELILFSNSDNER.S
4970	723 – 734	718.8545	1435.6945	1435.6943	0.15	0	43	0.0031	1		K.ELILFSNSDNER.S
4973	723 – 734	718.8548	1435.6950	1435.6943	0.51	0	26	0.027	1		K.ELILFSNSDNER.S
4974	723 – 734	718.8548	1435.6951	1435.6943	0.57	0	32	0.01	1		K.ELILFSNSDNER.S
4975	723 – 734	718.8551	1435.6956	1435.6943	0.92	0	40	0.0024	1		K.ELILFSNSDNER.S
4976	723 – 734	718.8552	1435.6958	1435.6943	1.07	0	45	0.00058	1		K.ELILFSNSDNER.S
4977	723 – 734	718.8557	1435.6969	1435.6943	1.82	0	32	0.0044	1		K.ELILFSNSDNER.S
5565	735 – 748	750.9114	1499.8082	1499.8096	-0.96	0	53	7.9e-05	1	U	R.SIPSLVDGFKPGQR.K
5572	735 – 748	750.9119	1499.8092	1499.8096	-0.26	0	34	0.0052	1	U	R.SIPSLVDGFKPGQR.K
5582	735 – 748	750.9123	1499.8101	1499.8096	0.30	0	57	3.8e-05	1	U	R.SIPSLVDGFKPGQR.K
5586	735 – 748	750.9128	1499.8110	1499.8096	0.89	0	35	0.00099	1	U	R.SIPSLVDGFKPGQR.K
5587	735 – 748	750.9129	1499.8112	1499.8096	1.06	0	42	0.00029	1	U	R.SIPSLVDGFKPGQR.K
2391	815 – 825	554.7966	1107.5785	1107.5785	0.051	1	36	0.0011	1	U	R.LHGGKDAASPR.Y
2392	815 – 825	554.7966	1107.5785	1107.5785	0.051	1	54	3.1e-05	1	U	R.LHGGKDAASPR.Y
2393	815 – 825	370.2001	1107.5786	1107.5785	0.064	1	33	0.0048	1	U	R.LHGGKDAASPR.Y
4128	826 – 836	658.3570	1314.6994	1314.7006	-0.89	0	46	0.00011	1	U	R.YIFTMLSTLAR.L
4129	826 – 836	658.3576	1314.7007	1314.7006	0.084	0	33	0.0013	1	U	R.YIFTMLSTLAR.L
4130	826 – 836	658.3577	1314.7008	1314.7006	0.15	0	46	0.00012	1	U	R.YIFTMLSTLAR.L
4131	826 – 836	658.3577	1314.7008	1314.7006	0.19	0	32	0.005	1	U	R.YIFTMLSTLAR.L
4132	826 – 836	658.3577	1314.7008	1314.7006	0.21	0	37	0.002	1	U	R.YIFTMLSTLAR.L
4133	826 – 836	658.3577	1314.7009	1314.7006	0.27	0	50	6.7e-05	1	U	R.YIFTMLSTLAR.L
4134	826 – 836	658.3578	1314.7011	1314.7006	0.39	0	37	0.0044	1	U	R.YIFTMLSTLAR.L
4135	826 – 836	658.3579	1314.7012	1314.7006	0.45	0	39	0.00022	1	U	R.YIFTMLSTLAR.L
4136	826 – 836	658.3579	1314.7013	1314.7006	0.54	0	47	0.00014	1	U	R.YIFTMLSTLAR.L
4138	826 – 836	658.3587	1314.7028	1314.7006	1.67	0	33	0.0011	1	U	R.YIFTMLSTLAR.L
4254	826 – 836	666.3544	1330.6943	1330.6955	-0.91	0	46	0.00013	1	U	R.YIFTMLSTLAR.L + Oxidation (M)
4255	826 – 836	666.3550	1330.6954	1330.6955	-0.096	0	21	0.0098	1	U	R.YIFTMLSTLAR.L + Oxidation (M)
4256	826 – 836	666.3550	1330.6954	1330.6955	-0.096	0	24	0.02	1	U	R.YIFTMLSTLAR.L + Oxidation (M)
4257	826 – 836	666.3550	1330.6954	1330.6955	-0.066	0	40	0.00037	1	U	R.YIFTMLSTLAR.L + Oxidation (M)
4258	826 – 836	666.3551	1330.6956	1330.6955	0.069	0	57	2.1e-05	1	U	R.YIFTMLSTLAR.L + Oxidation (M)
4260	826 – 836	666.3552	1330.6958	1330.6955	0.23	0	42	0.00061	1	U	R.YIFTMLSTLAR.L + Oxidation (M)
4261	826 – 836	666.3552	1330.6958	1330.6955	0.26	0	39	0.002	1	U	R.YIFTMLSTLAR.L + Oxidation (M)
4262	826 – 836	666.3552	1330.6959	1330.6955	0.31	0	34	0.00064	1	U	R.YIFTMLSTLAR.L + Oxidation (M)
4263	826 – 836	666.3552	1330.6959	1330.6955	0.31	0	46	0.00026	1	U	R.YIFTMLSTLAR.L + Oxidation (M)
4264	826 – 836	666.3552	1330.6959	1330.6955	0.34	0	53	4e-05	1	U	R.YIFTMLSTLAR.L + Oxidation (M)
4265	826 – 836	666.3554	1330.6962	1330.6955	0.50	0	49	9.2e-05	1	U	R.YIFTMLSTLAR.L + Oxidation (M)
4269	826 – 836	666.3567	1330.6988	1330.6955	2.52	0	50	0.00052	1	U	R.YIFTMLSTLAR.L + Oxidation (M)
4416	837 – 848	679.3894	1356.7642	1356.7653	-0.77	0	60	2e-05	1	U	R.LLFPAVDDNLLK.F
4417	837 – 848	679.3897	1356.7649	1356.7653	-0.28	0	47	0.00029	1	U	R.LLFPAVDDNLLK.F
4418	837 – 848	679.3898	1356.7650	1356.7653	-0.21	0	25	0.022	1	U	R.LLFPAVDDNLLK.F
4419	837 – 848	679.3898	1356.7650	1356.7653	-0.21	0	33	0.0054	1	U	R.LLFPAVDDNLLK.F
4420	837 – 848	679.3898	1356.7651	1356.7653	-0.10	0	32	0.007	1	U	R.LLFPAVDDNLLK.F
4421	837 – 848	679.3898	1356.7651	1356.7653	-0.10	0	33	0.0043	1	U	R.LLFPAVDDNLLK.F
4422	837 – 848	679.3899	1356.7652	1356.7653	-0.060	0	38	0.0037	1	U	R.LLFPAVDDNLLK.F
4423	837 – 848	679.3899	1356.7652	1356.7653	-0.030	0	45	0.00023	1	U	R.LLFPAVDDNLLK.F
4424	837 – 848	679.3899	1356.7653	1356.7653	-0.015	0	42	0.00077	1	U	R.LLFPAVDDNLLK.F
4425	837 – 848	679.3900	1356.7654	1356.7653	0.088	0	37	0.0039	1	U	R.LLFPAVDDNLLK.F
4426	837 – 848	679.3900	1356.7655	1356.7653	0.16	0	40	0.00085	1	U	R.LLFPAVDDNLLK.F
4427	837 – 848	679.3901	1356.7656	1356.7653	0.24	0	31	0.0024	1	U	R.LLFPAVDDNLLK.F
4428	837 – 848	679.3901	1356.7657	1356.7653	0.28	0	42	0.0027	1	U	R.LLFPAVDDNLLK.F
4430	837 – 848	679.3901	1356.7657	1356.7653	0.29	0	40	0.0032	1	U	R.LLFPAVDDNLLK.F
4432	837 – 848	679.3902	1356.7658	1356.7653	0.41	0	24	0.037	1	U	R.LLFPAVDDNLLK.F
4433	837 – 848	679.3903	1356.7660	1356.7653	0.53	0	37	0.0012	1	U	R.LLFPAVDDNLLK.F
4434	837 – 848	679.3903	1356.7660	1356.7653	0.56	0	33	0.015	1	U	R.LLFPAVDDNLLK.F
4435	837 – 848	679.3904	1356.7662	1356.7653	0.68	0	54	3.1e-05	1	U	R.LLFPAVDDNLLK.F
4436	837 – 848	679.3904	1356.7663	1356.7653	0.77	0	21	0.011	1	U	R.LLFPAVDDNLLK.F
4437	837 – 848	679.3905	1356.7664	1356.7653	0.82	0	33	0.0035	1	U	R.LLFPAVDDNLLK.F
4438	837 – 848	679.3905	1356.7664	1356.7653	0.84	0	25	0.0083	1	U	R.LLFPAVDDNLLK.F
4439	837 – 848	679.3905	1356.7665	1356.7653	0.90	0	18	0.031	1	U	R.LLFPAVDDNLLK.F
4440	837 – 848	679.3907	1356.7668	1356.7653	1.09	0	29	0.0065	1	U	R.LLFPAVDDNLLK.F
4441	837 – 848	679.3910	1356.7673	1356.7653	1.52	0	25	0.0044	1	U	R.LLFPAVDDNLLK.F
2074	849 – 856	535.7488	1069.4830	1069.4829	0.13	0	33	0.032	1		K.FLYDDNQR.V

Query	Start - End	Observed	Mr(expt)	Mr(calc)	ppm	M	Score	Expect	Rank	U	Peptide
630	885 - 891	424.7169	847.4192	847.4188	0.51	0	37	0.007	1	U	K.LPNYDAR.E
7713	900 - 914	870.9256	1739.8365	1739.8375	-0.54	0	66	7.4e-06	1	U	R.MLDGLDPHPMLPNYK.N
7715	900 - 914	580.9530	1739.8373	1739.8375	-0.11	0	35	0.013	1	U	R.MLDGLDPHPMLPNYK.N
7720	900 - 914	870.9270	1739.8395	1739.8375	1.17	0	46	0.00037	1	U	R.MLDGLDPHPMLPNYK.N
7832	900 - 914	878.9226	1755.8307	1755.8324	-0.96	0	31	0.003	1	U	R.MLDGLDPHPMLPNYK.N + Oxidation (M)
7834	900 - 914	878.9232	1755.8319	1755.8324	-0.27	0	53	7.3e-05	1	U	R.MLDGLDPHPMLPNYK.N + Oxidation (M)
7838	900 - 914	878.9241	1755.8337	1755.8324	0.72	0	34	0.0013	1	U	R.MLDGLDPHPMLPNYK.N + Oxidation (M)
7944	900 - 914	886.9206	1771.8267	1771.8273	-0.34	0	41	0.00024	1	U	R.MLDGLDPHPMLPNYK.N + 2 Oxidation (M)
7946	900 - 914	886.9209	1771.8272	1771.8273	-0.072	0	17	0.041	1	U	R.MLDGLDPHPMLPNYK.N + 2 Oxidation (M)
7948	900 - 914	886.9214	1771.8282	1771.8273	0.53	0	36	0.00089	1	U	R.MLDGLDPHPMLPNYK.N + 2 Oxidation (M)
7949	900 - 914	886.9215	1771.8284	1771.8273	0.59	0	36	0.0013	1	U	R.MLDGLDPHPMLPNYK.N + 2 Oxidation (M)
10582	918 - 939	1212.1115	2422.2084	2422.2129	-1.85	0	49	0.00027	1	U	K.GTIQELGQNQYAVSGEIFVVDR.N
10583	918 - 939	808.4103	2422.2091	2422.2129	-1.54	0	48	9.2e-05	1	U	K.GTIQELGQNQYAVSGEIFVVDR.N
10584	918 - 939	1212.1120	2422.2094	2422.2129	-1.44	0	34	0.00078	1	U	K.GTIQELGQNQYAVSGEIFVVDR.N
10586	918 - 939	1212.1121	2422.2097	2422.2129	-1.30	0	79	2.7e-07	1	U	K.GTIQELGQNQYAVSGEIFVVDR.N
10587	918 - 939	1212.1126	2422.2107	2422.2129	-0.90	0	38	0.00028	1	U	K.GTIQELGQNQYAVSGEIFVVDR.N
10588	918 - 939	1212.1127	2422.2108	2422.2129	-0.86	0	82	4.6e-08	1	U	K.GTIQELGQNQYAVSGEIFVVDR.N
10589	918 - 939	1212.1129	2422.2112	2422.2129	-0.67	0	26	0.0076	1	U	K.GTIQELGQNQYAVSGEIFVVDR.N
10591	918 - 939	1212.1130	2422.2114	2422.2129	-0.60	0	130	4e-12	1	U	K.GTIQELGQNQYAVSGEIFVVDR.N
10592	918 - 939	1212.1132	2422.2118	2422.2129	-0.43	0	61	1.3e-05	1	U	K.GTIQELGQNQYAVSGEIFVVDR.N
10593	918 - 939	1212.1132	2422.2118	2422.2129	-0.42	0	66	2.6e-06	1	U	K.GTIQELGQNQYAVSGEIFVVDR.N
10594	918 - 939	1212.1132	2422.2119	2422.2129	-0.38	0	83	1.1e-07	1	U	K.GTIQELGQNQYAVSGEIFVVDR.N
10595	918 - 939	808.4113	2422.2120	2422.2129	-0.37	0	24	0.045	1	U	K.GTIQELGQNQYAVSGEIFVVDR.N
10596	918 - 939	808.4113	2422.2122	2422.2129	-0.29	0	57	4.3e-05	1	U	K.GTIQELGQNQYAVSGEIFVVDR.N
10598	918 - 939	1212.1134	2422.2122	2422.2129	-0.26	0	36	0.001	1	U	K.GTIQELGQNQYAVSGEIFVVDR.N
10599	918 - 939	808.4114	2422.2123	2422.2129	-0.24	0	26	0.024	1	U	K.GTIQELGQNQYAVSGEIFVVDR.N
10600	918 - 939	808.4114	2422.2123	2422.2129	-0.21	0	19	0.023	1	U	K.GTIQELGQNQYAVSGEIFVVDR.N
10601	918 - 939	1212.1135	2422.2124	2422.2129	-0.19	0	46	0.00053	1	U	K.GTIQELGQNQYAVSGEIFVVDR.N
10602	918 - 939	1212.1135	2422.2124	2422.2129	-0.18	0	30	0.0014	1	U	K.GTIQELGQNQYAVSGEIFVVDR.N
10603	918 - 939	1212.1135	2422.2125	2422.2129	-0.15	0	101	4.7e-09	1	U	K.GTIQELGQNQYAVSGEIFVVDR.N
10604	918 - 939	808.4114	2422.2125	2422.2129	-0.14	0	18	0.046	1	U	K.GTIQELGQNQYAVSGEIFVVDR.N
10607	918 - 939	808.4115	2422.2126	2422.2129	-0.10	0	27	0.028	1	U	K.GTIQELGQNQYAVSGEIFVVDR.N
10608	918 - 939	808.4115	2422.2127	2422.2129	-0.065	0	26	0.023	1	U	K.GTIQELGQNQYAVSGEIFVVDR.N
10613	918 - 939	1212.1140	2422.2134	2422.2129	0.23	0	59	2e-05	1	U	K.GTIQELGQNQYAVSGEIFVVDR.N
10615	918 - 939	1212.1141	2422.2136	2422.2129	0.29	0	91	1.3e-08	1	U	K.GTIQELGQNQYAVSGEIFVVDR.N
10618	918 - 939	1212.1144	2422.2142	2422.2129	0.55	0	23	0.018	1	U	K.GTIQELGQNQYAVSGEIFVVDR.N
10619	918 - 939	808.4120	2422.2142	2422.2129	0.55	0	34	0.0034	1	U	K.GTIQELGQNQYAVSGEIFVVDR.N
10622	918 - 939	1212.1146	2422.2147	2422.2129	0.76	0	82	1.2e-07	1	U	K.GTIQELGQNQYAVSGEIFVVDR.N
10623	918 - 939	808.4122	2422.2149	2422.2129	0.85	0	56	0.00026	1	U	K.GTIQELGQNQYAVSGEIFVVDR.N
10624	918 - 939	808.4123	2422.2150	2422.2129	0.90	0	31	0.01	1	U	K.GTIQELGQNQYAVSGEIFVVDR.N
10626	918 - 939	808.4124	2422.2154	2422.2129	1.04	0	37	0.025	1	U	K.GTIQELGQNQYAVSGEIFVVDR.N
10627	918 - 939	1212.1150	2422.2154	2422.2129	1.06	0	43	0.00052	1	U	K.GTIQELGQNQYAVSGEIFVVDR.N
10628	918 - 939	808.4125	2422.2157	2422.2129	1.17	0	31	0.0067	1	U	K.GTIQELGQNQYAVSGEIFVVDR.N
10629	918 - 939	606.5612	2422.2159	2422.2129	1.25	0	32	0.0056	1	U	K.GTIQELGQNQYAVSGEIFVVDR.N
10630	918 - 939	1212.1153	2422.2160	2422.2129	1.28	0	52	4.4e-05	1	U	K.GTIQELGQNQYAVSGEIFVVDR.N
10631	918 - 939	606.5613	2422.2161	2422.2129	1.35	0	36	0.0053	1	U	K.GTIQELGQNQYAVSGEIFVVDR.N
10633	918 - 939	808.4128	2422.2167	2422.2129	1.59	0	26	0.016	1	U	K.GTIQELGQNQYAVSGEIFVVDR.N
10634	918 - 939	808.4139	2422.2198	2422.2129	2.86	0	31	0.011	1	U	K.GTIQELGQNQYAVSGEIFVVDR.N
13173	918 - 950	919.4792	3673.8875	3673.8952	-2.09	1	44	0.0037	1	U	K.GTIQELGQNQYAVSGEIFVVDRNTVEITELPVR.T
13174	918 - 950	919.4807	3673.8938	3673.8952	-0.37	1	52	7.1e-05	1	U	K.GTIQELGQNQYAVSGEIFVVDRNTVEITELPVR.T
13175	918 - 950	1225.6392	3673.8957	3673.8952	0.15	1	78	1.4e-06	1	U	K.GTIQELGQNQYAVSGEIFVVDRNTVEITELPVR.T
13176	918 - 950	1225.6393	3673.8961	3673.8952	0.26	1	89	3.9e-08	1	U	K.GTIQELGQNQYAVSGEIFVVDRNTVEITELPVR.T
3750	940 - 950	635.8526	1269.6907	1269.6929	-1.68	0	38	0.0018	1	U	R.NTVEITELPVR.T
3751	940 - 950	635.8532	1269.6918	1269.6929	-0.84	0	67	1.1e-05	1	U	R.NTVEITELPVR.T
3752	940 - 950	635.8534	1269.6922	1269.6929	-0.54	0	24	0.027	1	U	R.NTVEITELPVR.T
3753	940 - 950	635.8534	1269.6922	1269.6929	-0.49	0	54	0.00013	1	U	R.NTVEITELPVR.T
3754	940 - 950	424.2381	1269.6925	1269.6929	-0.28	0	46	0.0012	1	U	R.NTVEITELPVR.T
3756	940 - 950	635.8537	1269.6929	1269.6929	0.041	0	54	0.0002	1	U	R.NTVEITELPVR.T
3757	940 - 950	635.8538	1269.6929	1269.6929	0.072	0	58	2.7e-05	1	U	R.NTVEITELPVR.T
3759	940 - 950	635.8538	1269.6931	1269.6929	0.20	0	55	0.00018	1	U	R.NTVEITELPVR.T
3760	940 - 950	635.8538	1269.6931	1269.6929	0.23	0	42	0.00012	1	U	R.NTVEITELPVR.T
3762	940 - 950	635.8541	1269.6937	1269.6929	0.64	0	39	0.00096	1	U	R.NTVEITELPVR.T
3763	940 - 950	635.8542	1269.6939	1269.6929	0.83	0	48	0.00022	1	U	R.NTVEITELPVR.T
3764	940 - 950	635.8544	1269.6942	1269.6929	1.05	0	56	4e-05	1	U	R.NTVEITELPVR.T
5331	958 - 970	737.3663	1472.7181	1472.7181	0.033	0	40	0.0042	1	U	K.EQVLEPMLNGTDK.T
5448	958 - 970	745.3634	1488.7122	1488.7130	-0.56	0	42	0.00088	1	U	K.EQVLEPMLNGTDK.T + Oxidation (M)
5449	958 - 970	745.3638	1488.7131	1488.7130	0.048	0	57	4.2e-05	1	U	K.EQVLEPMLNGTDK.T + Oxidation (M)
10809	958 - 979	826.7517	2477.2332	2477.2359	-1.09	1	24	0.044	1	U	K.EQVLEPMLNGTDKTPALISDYK.E + Oxidation (M)
10810	958 - 979	826.7530	2477.2373	2477.2359	0.56	1	39	0.0016	1	U	K.EQVLEPMLNGTDKTPALISDYK.E + Oxidation (M)
1559	971 - 979	504.2730	1006.5313	1006.5335	-2.13	0	18	0.034	1	U	K.TPALISDYK.E
1561	971 - 979	504.2743	1006.5340	1006.5335	0.47	0	27	0.016	1	U	K.TPALISDYK.E
1562	971 - 979	504.2744	1006.5341	1006.5335	0.65	0	36	0.0019	1	U	K.TPALISDYK.E
1563	971 - 979	504.2744	1006.5342	1006.5335	0.75	0	31	0.006	1	U	K.TPALISDYK.E
1564	971 - 979	504.2746	1006.5346	1006.5335	1.09	0	33	0.0064	1	U	K.TPALISDYK.E
9622	971 - 988	694.6839	2081.0298	2081.0317	-0.90	1	48	0.00022	1	U	K.TPALISDYKEYHTDTTVK.F
9625	971 - 988	694.6843	2081.0310	2081.0317	-0.36	1	30	0.0077	1	U	K.TPALISDYKEYHTDTTVK.F
9627	971 - 988	521.2652	2081.0318	2081.0317	0.059	1	34	0.0059	1	U	K.TPALISDYKEYHTDTTVK.F
2284	980 - 988	547.2613	1092.5080	1092.5088	-0.69	0	27	0.041	1		K.EYHTDTTVK.F
2289	980 - 988	547.2635	1092.5124	1092.5088	3.34	0	23	0.031	1		K.EYHTDTTVK.F
7582	993 - 1008	576.2980	1725.8722	1725.8719	0.18	1	35	0.00092	1	U	K.MTEEKLAQAEAAGLHK.V
2397	998 - 1008	554.8087	1107.6029	1107.6036	-0.64	0	55	8.4e-05	1	U	K.LAQAEAAGLHK.V
2398	998 - 1008	370.2083	1107.6030	1107.6036	-0.52	0	36	0.011	1	U	K.LAQAEAAGLHK.V
2399	998 - 1008	554.8089	1107.6032	1107.6036	-0.37	0	42	0.0012	1	U	K.LAQAEAAGLHK.V

Query	Start - End	Observed	Mr(expt)	Mr(calc)	ppm	M	Score	Expect	Rank	U	Peptide
2400	998 - 1008	554.8090	1107.6034	1107.6036	-0.18	0	25	0.015	1	U	K.LAQAEAAGLHK.V
2401	998 - 1008	370.2085	1107.6036	1107.6036	0.018	0	38	0.0063	1	U	K.LAQAEAAGLHK.V
2402	998 - 1008	554.8091	1107.6037	1107.6036	0.060	0	54	7.5e-05	1	U	K.LAQAEAAGLHK.V
2403	998 - 1008	370.2085	1107.6037	1107.6036	0.099	0	42	0.0011	1	U	K.LAQAEAAGLHK.V
2404	998 - 1008	370.2086	1107.6039	1107.6036	0.21	0	39	0.003	1	U	K.LAQAEAAGLHK.V
2407	998 - 1008	370.2088	1107.6046	1107.6036	0.88	0	26	0.03	1	U	K.LAQAEAAGLHK.V
3445	1032 - 1041	618.8449	1235.6753	1235.6761	-0.65	1	66	2.9e-06	1	U	K.KYETVQDILK.E
3448	1032 - 1041	618.8452	1235.6758	1235.6761	-0.29	1	33	0.0021	1	U	K.KYETVQDILK.E
3449	1032 - 1041	412.8992	1235.6758	1235.6761	-0.27	1	23	0.02	1	U	K.KYETVQDILK.E
3450	1032 - 1041	618.8453	1235.6761	1235.6761	-0.036	1	43	0.00028	1	U	K.KYETVQDILK.E
3451	1032 - 1041	618.8454	1235.6762	1235.6761	0.094	1	34	0.0033	1	U	K.KYETVQDILK.E
3452	1032 - 1041	412.8994	1235.6764	1235.6761	0.24	1	40	0.0044	1	U	K.KYETVQDILK.E
3453	1032 - 1041	618.8455	1235.6765	1235.6761	0.30	1	40	0.0014	1	U	K.KYETVQDILK.E
3454	1032 - 1041	618.8456	1235.6765	1235.6761	0.34	1	51	6.6e-05	1	U	K.KYETVQDILK.E
9473	1032 - 1047	682.0298	2043.0676	2043.0677	-0.049	2	28	0.013	1	U	K.KYETVQDILKEFFDLR.L
2394	1033 - 1041	554.7975	1107.5805	1107.5812	-0.59	0	39	0.0063	1	U	K.YETVQDILK.E
742	1048 - 1054	436.2372	870.4598	870.4599	-0.19	0	28	0.029	1	U	R.LSYYGLR.K
6166	1055 - 1068	516.9409	1547.8010	1547.8017	-0.50	1	25	0.011	1	U	R.KEWLVGMLGAESTK.L
6167	1055 - 1068	516.9410	1547.8013	1547.8017	-0.27	1	26	0.032	1	U	R.KEWLVGMLGAESTK.L
6168	1055 - 1068	516.9411	1547.8014	1547.8017	-0.25	1	36	0.0013	1	U	R.KEWLVGMLGAESTK.L
6170	1055 - 1068	774.9085	1547.8024	1547.8017	0.44	1	76	2e-06	1	U	R.KEWLVGMLGAESTK.L
6304	1055 - 1068	782.9034	1563.7923	1563.7967	-2.76	1	57	5.8e-05	1	U	R.KEWLVGMLGAESTK.L + Oxidation (M)
6305	1055 - 1068	522.2720	1563.7942	1563.7967	-1.57	1	29	0.0032	1	U	R.KEWLVGMLGAESTK.L + Oxidation (M)
6306	1055 - 1068	522.2726	1563.7958	1563.7967	-0.54	1	29	0.006	1	U	R.KEWLVGMLGAESTK.L + Oxidation (M)
6307	1055 - 1068	522.2726	1563.7959	1563.7967	-0.52	1	20	0.027	1	U	R.KEWLVGMLGAESTK.L + Oxidation (M)
6308	1055 - 1068	782.9055	1563.7965	1563.7967	-0.12	1	46	5.8e-05	1	U	R.KEWLVGMLGAESTK.L + Oxidation (M)
6309	1055 - 1068	782.9055	1563.7965	1563.7967	-0.11	1	64	4.9e-06	1	U	R.KEWLVGMLGAESTK.L + Oxidation (M)
6310	1055 - 1068	782.9056	1563.7967	1563.7967	0.0045	1	68	7e-06	1	U	R.KEWLVGMLGAESTK.L + Oxidation (M)
6311	1055 - 1068	522.2729	1563.7968	1563.7967	0.058	1	35	0.0032	1	U	R.KEWLVGMLGAESTK.L + Oxidation (M)
6312	1055 - 1068	522.2729	1563.7968	1563.7967	0.12	1	49	0.00021	1	U	R.KEWLVGMLGAESTK.L + Oxidation (M)
6313	1055 - 1068	782.9058	1563.7970	1563.7967	0.21	1	44	0.0004	1	U	R.KEWLVGMLGAESTK.L + Oxidation (M)
6314	1055 - 1068	782.9059	1563.7972	1563.7967	0.32	1	63	6.3e-06	1	U	R.KEWLVGMLGAESTK.L + Oxidation (M)
6315	1055 - 1068	522.2731	1563.7974	1563.7967	0.46	1	31	0.0035	1	U	R.KEWLVGMLGAESTK.L + Oxidation (M)
6316	1055 - 1068	782.9065	1563.7984	1563.7967	1.09	1	46	0.00082	1	U	R.KEWLVGMLGAESTK.L + Oxidation (M)
4823	1056 - 1068	710.8609	1419.7073	1419.7068	0.35	0	21	0.04	1	U	K.EWLVGMLGAESTK.L
4825	1056 - 1068	710.8609	1419.7073	1419.7068	0.37	0	48	5.6e-05	1	U	K.EWLVGMLGAESTK.L
4826	1056 - 1068	710.8610	1419.7075	1419.7068	0.48	0	33	0.0038	1	U	K.EWLVGMLGAESTK.L
4827	1056 - 1068	474.2433	1419.7082	1419.7068	0.97	0	31	0.0036	1	U	K.EWLVGMLGAESTK.L
4828	1056 - 1068	710.8614	1419.7082	1419.7068	1.00	0	26	0.0036	1	U	K.EWLVGMLGAESTK.L
4829	1056 - 1068	710.8619	1419.7092	1419.7068	1.69	0	22	0.014	1	U	K.EWLVGMLGAESTK.L
4982	1056 - 1068	718.8579	1435.7013	1435.7017	-0.28	0	37	0.002	1	U	K.EWLVGMLGAESTK.L + Oxidation (M)
4983	1056 - 1068	718.8581	1435.7016	1435.7017	-0.096	0	34	0.034	1	U	K.EWLVGMLGAESTK.L + Oxidation (M)
4985	1056 - 1068	718.8583	1435.7020	1435.7017	0.21	0	35	0.0022	1	U	K.EWLVGMLGAESTK.L + Oxidation (M)
2124	1075 - 1083	538.3292	1074.6439	1074.6437	0.22	1	37	0.00046	1	U	R.FILEKIQGK.I
3523	1092 - 1101	415.2444	1242.7115	1242.7118	-0.25	1	44	0.0015	1	U	K.KDLIQMLVQR.G
3524	1092 - 1101	622.3630	1242.7115	1242.7118	-0.24	1	59	4.8e-05	1	U	K.KDLIQMLVQR.G
3660	1092 - 1101	630.3607	1258.7069	1258.7067	0.14	1	55	0.00013	1	U	K.KDLIQMLVQR.G + Oxidation (M)
3661	1092 - 1101	630.3608	1258.7071	1258.7067	0.30	1	51	0.00047	1	U	K.KDLIQMLVQR.G + Oxidation (M)
3662	1092 - 1101	420.5769	1258.7090	1258.7067	1.80	1	39	0.0071	1	U	K.KDLIQMLVQR.G + Oxidation (M)
2468	1093 - 1101	558.3152	1114.6159	1114.6169	-0.83	0	48	0.00098	1	U	K.DLIQMLVQR.G
2469	1093 - 1101	558.3153	1114.6161	1114.6169	-0.69	0	48	0.00096	1	U	K.DLIQMLVQR.G
2470	1093 - 1101	558.3157	1114.6168	1114.6169	-0.039	0	64	2.2e-05	1	U	K.DLIQMLVQR.G
2472	1093 - 1101	558.3158	1114.6171	1114.6169	0.27	0	50	0.00053	1	U	K.DLIQMLVQR.G
2473	1093 - 1101	558.3159	1114.6173	1114.6169	0.41	0	39	0.0073	1	U	K.DLIQMLVQR.G
2474	1093 - 1101	558.3162	1114.6177	1114.6169	0.80	0	51	0.00047	1	U	K.DLIQMLVQR.G
2628	1093 - 1101	566.3118	1130.6090	1130.6118	-2.46	0	33	0.028	1	U	K.DLIQMLVQR.G + Oxidation (M)
2629	1093 - 1101	566.3122	1130.6099	1130.6118	-1.61	0	31	0.033	1	U	K.DLIQMLVQR.G + Oxidation (M)
2630	1093 - 1101	566.3130	1130.6114	1130.6118	-0.32	0	49	0.00038	1	U	K.DLIQMLVQR.G + Oxidation (M)
2631	1093 - 1101	566.3130	1130.6115	1130.6118	-0.25	0	48	0.0007	1	U	K.DLIQMLVQR.G + Oxidation (M)
2632	1093 - 1101	566.3132	1130.6118	1130.6118	0.068	0	37	0.0029	1	U	K.DLIQMLVQR.G + Oxidation (M)
2634	1093 - 1101	566.3134	1130.6122	1130.6118	0.40	0	54	0.00026	1	U	K.DLIQMLVQR.G + Oxidation (M)
2635	1093 - 1101	566.3134	1130.6123	1130.6118	0.44	0	51	0.00035	1	U	K.DLIQMLVQR.G + Oxidation (M)
2636	1093 - 1101	566.3135	1130.6124	1130.6118	0.53	0	50	0.00064	1	U	K.DLIQMLVQR.G + Oxidation (M)
2638	1093 - 1101	566.3138	1130.6130	1130.6118	1.11	0	44	0.0012	1	U	K.DLIQMLVQR.G + Oxidation (M)
847	1102 - 1109	447.7137	893.4128	893.4131	-0.28	0	23	0.011	1	U	R.GYESDPVK.A
851	1102 - 1109	447.7140	893.4134	893.4131	0.37	0	25	0.041	1	U	R.GYESDPVK.A
1428	1157 - 1164	494.2895	986.5645	986.5648	-0.25	1	39	0.0072	1	U	K.EKVEELIK.Q

Query	Start – End	Observed	Mr(expt)	Mr(calc)	ppm	M	Score	Expect	Rank	U	Peptide
1430	1157 – 1164	494.2898	986.5651	986.5648	0.30	1	46	0.0013	1	U	K.EKVEELIK.Q
2229	1170 – 1178	543.8043	1085.5941	1085.5941	-0.040	2	42	0.0034	1	U	K.GREVNDLKR.K
2230	1170 – 1178	362.8722	1085.5947	1085.5941	0.48	2	39	0.0075	1	U	K.GREVNDLKR.K
756	1172 – 1178	437.2428	872.4711	872.4716	-0.54	1	44	0.0022	1	U	R.EVNDLKR.K
533	1180 – 1186	416.7135	831.4124	831.4127	-0.28	0	27	0.04	1		K.SPSDLWK.E
4573	1187 – 1198	689.8402	1377.6659	1377.6664	-0.32	0	63	1.1e-05	1	U	K.EDLAAFVEELDK.V
4574	1187 – 1198	689.8404	1377.6661	1377.6664	-0.15	0	53	8.5e-05	1	U	K.EDLAAFVEELDK.V
4575	1187 – 1198	689.8414	1377.6683	1377.6664	1.45	0	20	0.028	1	U	K.EDLAAFVEELDK.V
9727	1187 – 1204	703.0101	2106.0084	2106.0117	-1.54	1	65	1.2e-05	1	U	K.EDLAAFVEELDKVESQER.E
9728	1187 – 1204	703.0105	2106.0096	2106.0117	-1.00	1	50	0.0003	1	U	K.EDLAAFVEELDKVESQER.E
9729	1187 – 1204	1054.0125	2106.0104	2106.0117	-0.57	1	57	1.5e-05	1	U	K.EDLAAFVEELDKVESQER.E
9730	1187 – 1204	703.0109	2106.0110	2106.0117	-0.32	1	36	0.0019	1	U	K.EDLAAFVEELDKVESQER.E
9731	1187 – 1204	703.0109	2106.0110	2106.0117	-0.32	1	36	0.013	1	U	K.EDLAAFVEELDKVESQER.E
9732	1187 – 1204	703.0110	2106.0111	2106.0117	-0.27	1	33	0.0067	1	U	K.EDLAAFVEELDKVESQER.E
9733	1187 – 1204	1054.0128	2106.0111	2106.0117	-0.25	1	75	1.3e-06	1	U	K.EDLAAFVEELDKVESQER.E
9734	1187 – 1204	1054.0129	2106.0113	2106.0117	-0.19	1	61	6.4e-06	1	U	K.EDLAAFVEELDKVESQER.E
9736	1187 – 1204	1054.0130	2106.0115	2106.0117	-0.090	1	61	3.8e-06	1	U	K.EDLAAFVEELDKVESQER.E
9737	1187 – 1204	1054.0131	2106.0117	2106.0117	0.033	1	74	4e-07	1	U	K.EDLAAFVEELDKVESQER.E
9738	1187 – 1204	703.0112	2106.0117	2106.0117	0.040	1	45	0.00057	1	U	K.EDLAAFVEELDKVESQER.E
9739	1187 – 1204	703.0112	2106.0117	2106.0117	0.040	1	48	0.00042	1	U	K.EDLAAFVEELDKVESQER.E
9740	1187 – 1204	703.0112	2106.0118	2106.0117	0.054	1	50	0.00016	1	U	K.EDLAAFVEELDKVESQER.E
9741	1187 – 1204	703.0112	2106.0119	2106.0117	0.097	1	27	0.011	1	U	K.EDLAAFVEELDKVESQER.E
9742	1187 – 1204	1054.0133	2106.0121	2106.0117	0.19	1	76	3.4e-07	1	U	K.EDLAAFVEELDKVESQER.E
9743	1187 – 1204	703.0113	2106.0121	2106.0117	0.20	1	46	0.0012	1	U	K.EDLAAFVEELDKVESQER.E
9744	1187 – 1204	703.0113	2106.0121	2106.0117	0.23	1	68	5e-06	1	U	K.EDLAAFVEELDKVESQER.E
9745	1187 – 1204	703.0113	2106.0122	2106.0117	0.25	1	42	0.0023	1	U	K.EDLAAFVEELDKVESQER.E
9746	1187 – 1204	703.0114	2106.0123	2106.0117	0.28	1	47	0.00049	1	U	K.EDLAAFVEELDKVESQER.E
9748	1187 – 1204	1054.0138	2106.0130	2106.0117	0.63	1	65	6.7e-06	1	U	K.EDLAAFVEELDKVESQER.E
9749	1187 – 1204	703.0118	2106.0135	2106.0117	0.85	1	32	0.0076	1	U	K.EDLAAFVEELDKVESQER.E
9750	1187 – 1204	1054.0209	2106.0272	2106.0117	7.36	1	64	2.7e-05	1	U	K.EDLAAFVEELDKVESQER.E
12217	1187 – 1214	1032.1675	3093.4808	3093.4812	-0.13	2	65	2.5e-05	1	U	K.EDLAAFVEELDKVESQEREDVLAGMSGK.A
12218	1187 – 1214	1032.1680	3093.4823	3093.4812	0.35	2	46	0.0004	1	U	K.EDLAAFVEELDKVESQEREDVLAGMSGK.A
12220	1187 – 1214	1032.1686	3093.4841	3093.4812	0.94	2	32	0.0068	1	U	K.EDLAAFVEELDKVESQEREDVLAGMSGK.A
12257	1187 – 1214	1037.4990	3109.4752	3109.4761	-0.30	2	41	0.0013	1	U	K.EDLAAFVEELDKVESQEREDVLAGMSGK.A + Oxidation (M)
12258	1187 – 1214	1037.4991	3109.4755	3109.4761	-0.20	2	46	0.0011	1	U	K.EDLAAFVEELDKVESQEREDVLAGMSGK.A + Oxidation (M)
12259	1187 – 1214	1037.4992	3109.4759	3109.4761	-0.069	2	41	0.0012	1	U	K.EDLAAFVEELDKVESQEREDVLAGMSGK.A + Oxidation (M)
12260	1187 – 1214	1037.4995	3109.4767	3109.4761	0.20	2	58	2.1e-05	1	U	K.EDLAAFVEELDKVESQEREDVLAGMSGK.A + Oxidation (M)
12262	1187 – 1214	1037.5002	3109.4788	3109.4761	0.86	2	34	0.0066	1	U	K.EDLAAFVEELDKVESQEREDVLAGMSGK.A + Oxidation (M)
7661	1199 – 1214	867.9201	1733.8257	1733.8254	0.18	1	57	1.9e-05	1	U	K.VESQEREDVLAGMSGK.A
7662	1199 – 1214	578.9494	1733.8263	1733.8254	0.53	1	40	0.0033	1	U	K.VESQEREDVLAGMSGK.A
7663	1199 – 1214	867.9207	1733.8269	1733.8254	0.88	1	74	5.5e-07	1	U	K.VESQEREDVLAGMSGK.A
7789	1199 – 1214	584.2806	1749.8201	1749.8203	-0.14	1	49	0.00026	1	U	K.VESQEREDVLAGMSGK.A + Oxidation (M)
7790	1199 – 1214	875.9174	1749.8203	1749.8203	-0.0023	1	51	0.00016	1	U	K.VESQEREDVLAGMSGK.A + Oxidation (M)
7793	1199 – 1214	875.9177	1749.8208	1749.8203	0.28	1	62	1.5e-05	1	U	K.VESQEREDVLAGMSGK.A + Oxidation (M)
7794	1199 – 1214	584.2812	1749.8219	1749.8203	0.89	1	27	0.03	1	U	K.VESQEREDVLAGMSGK.A + Oxidation (M)
1551	1205 – 1214	503.7475	1005.4805	1005.4801	0.44	0	56	3.6e-05	1	U	R.EDVLAGMSGK.A
1672	1205 – 1214	511.7441	1021.4737	1021.4750	-1.25	0	54	9.8e-05	1	U	R.EDVLAGMSGK.A + Oxidation (M)
1673	1205 – 1214	511.7444	1021.4742	1021.4750	-0.76	0	53	0.00011	1	U	R.EDVLAGMSGK.A + Oxidation (M)
1674	1205 – 1214	511.7446	1021.4747	1021.4750	-0.27	0	58	2.8e-05	1	U	R.EDVLAGMSGK.A + Oxidation (M)
1675	1205 – 1214	511.7446	1021.4747	1021.4750	-0.25	0	38	0.0032	1	U	R.EDVLAGMSGK.A + Oxidation (M)
1676	1205 – 1214	511.7447	1021.4749	1021.4750	-0.096	0	52	0.00016	1	U	R.EDVLAGMSGK.A + Oxidation (M)
1677	1205 – 1214	511.7448	1021.4750	1021.4750	0.0020	0	46	0.00032	1	U	R.EDVLAGMSGK.A + Oxidation (M)
1678	1205 – 1214	511.7448	1021.4751	1021.4750	0.080	0	52	0.00015	1	U	R.EDVLAGMSGK.A + Oxidation (M)
6952	1227 – 1240	824.9216	1647.8286	1647.8290	-0.27	1	79	2.3e-07	1	U	K.KLQLEETMPSPYGR.R
7043	1227 – 1240	832.9186	1663.8225	1663.8239	-0.83	1	54	3.2e-05	1	U	K.KLQLEETMPSPYGR.R + Oxidation (M)
7044	1227 – 1240	832.9187	1663.8228	1663.8239	-0.66	1	71	5.9e-07	1	U	K.KLQLEETMPSPYGR.R + Oxidation (M)
7045	1227 – 1240	832.9188	1663.8231	1663.8239	-0.49	1	67	1.1e-06	1	U	K.KLQLEETMPSPYGR.R + Oxidation (M)
7046	1227 – 1240	832.9189	1663.8232	1663.8239	-0.42	1	62	4.9e-06	1	U	K.KLQLEETMPSPYGR.R + Oxidation (M)
7047	1227 – 1240	555.6151	1663.8235	1663.8239	-0.26	1	23	0.016	1	U	K.KLQLEETMPSPYGR.R + Oxidation (M)
7049	1227 – 1240	555.6153	1663.8242	1663.8239	0.14	1	29	0.014	1	U	K.KLQLEETMPSPYGR.R + Oxidation (M)
7050	1227 – 1240	832.9194	1663.8243	1663.8239	0.20	1	59	6.9e-06	1	U	K.KLQLEETMPSPYGR.R + Oxidation (M)
7051	1227 – 1240	832.9195	1663.8244	1663.8239	0.28	1	52	5.5e-05	1	U	K.KLQLEETMPSPYGR.R + Oxidation (M)
7052	1227 – 1240	555.6155	1663.8247	1663.8239	0.45	1	23	0.023	1	U	K.KLQLEETMPSPYGR.R + Oxidation (M)
7054	1227 – 1240	555.6156	1663.8251	1663.8239	0.70	1	33	0.0047	1	U	K.KLQLEETMPSPYGR.R + Oxidation (M)
7055	1227 – 1240	832.9199	1663.8252	1663.8239	0.78	1	76	4.4e-07	1	U	K.KLQLEETMPSPYGR.R + Oxidation (M)
7056	1227 – 1240	832.9201	1663.8257	1663.8239	1.07	1	78	2.8e-07	1	U	K.KLQLEETMPSPYGR.R + Oxidation (M)
5803	1228 – 1240	507.5850	1519.7331	1519.7341	-0.64	0	27	0.018	1	U	K.LQLEETMPSPYGR.R

Query	Start - End	Observed	Mr(expt)	Mr(calc)	ppm	M	Score	Expect	Rank	U	Peptide
5804	1228 - 1240	760.8738	1519.7331	1519.7341	-0.62	0	65	1.3e-06	1	U	K.LQLEETMPSPYGR.R
5806	1228 - 1240	760.8771	1519.7396	1519.7341	3.64	0	41	0.00015	1	U	K.LQLEETMPSPYGR.R
5807	1228 - 1240	760.8771	1519.7397	1519.7341	3.72	0	37	0.00065	1	U	K.LQLEETMPSPYGR.R
6018	1228 - 1240	768.8703	1535.7260	1535.7290	-1.90	0	66	3.6e-06	1	U	K.LQLEETMPSPYGR.R + Oxidation (M)
6019	1228 - 1240	768.8707	1535.7268	1535.7290	-1.44	0	41	0.00055	1	U	K.LQLEETMPSPYGR.R + Oxidation (M)
6020	1228 - 1240	768.8714	1535.7283	1535.7290	-0.43	0	55	4.6e-05	1	U	K.LQLEETMPSPYGR.R + Oxidation (M)
6021	1228 - 1240	768.8714	1535.7283	1535.7290	-0.42	0	48	0.00017	1	U	K.LQLEETMPSPYGR.R + Oxidation (M)
6022	1228 - 1240	768.8717	1535.7289	1535.7290	-0.029	0	61	5.1e-06	1	U	K.LQLEETMPSPYGR.R + Oxidation (M)
6024	1228 - 1240	768.8718	1535.7290	1535.7290	0.036	0	64	2.5e-06	1	U	K.LQLEETMPSPYGR.R + Oxidation (M)
6025	1228 - 1240	768.8719	1535.7292	1535.7290	0.18	0	83	7.5e-08	1	U	K.LQLEETMPSPYGR.R + Oxidation (M)
2998	1241 - 1250	586.3470	1170.6794	1170.6794		1	50	0.00012	1	U	R.RIIPEITAMK.A
2999	1241 - 1250	391.2338	1170.6797	1170.6794	0.25	1	39	0.00074	1	U	R.RIIPEITAMK.A
3000	1241 - 1250	586.3475	1170.6804	1170.6794	0.82	1	22	0.0081	1	U	R.RIIPEITAMK.A
3099	1241 - 1250	594.3434	1186.6722	1186.6743	-1.82	1	21	0.015	1	U	R.RIIPEITAMK.A + Oxidation (M)
3101	1241 - 1250	594.3441	1186.6736	1186.6743	-0.61	1	29	0.0035	1	U	R.RIIPEITAMK.A + Oxidation (M)
3102	1241 - 1250	396.5652	1186.6738	1186.6743	-0.48	1	34	0.0015	1	U	R.RIIPEITAMK.A + Oxidation (M)
3103	1241 - 1250	594.3445	1186.6744	1186.6743	0.051	1	35	0.0011	1	U	R.RIIPEITAMK.A + Oxidation (M)
3104	1241 - 1250	396.5655	1186.6746	1186.6743	0.20	1	25	0.0055	1	U	R.RIIPEITAMK.A + Oxidation (M)
3105	1241 - 1250	594.3446	1186.6746	1186.6743	0.24	1	37	0.0005	1	U	R.RIIPEITAMK.A + Oxidation (M)
3106	1241 - 1250	396.5655	1186.6747	1186.6743	0.32	1	15	0.045	1	U	R.RIIPEITAMK.A + Oxidation (M)
3107	1241 - 1250	594.3446	1186.6747	1186.6743	0.34	1	36	0.00088	1	U	R.RIIPEITAMK.A + Oxidation (M)
1753	1242 - 1250	516.2937	1030.5728	1030.5732	-0.42	0	36	0.018	1	U	R.IIPEITAMK.A + Oxidation (M)
2760	1261 - 1271	382.5557	1144.6452	1144.6452	-0.017	2	22	0.04	1	U	K.KKGDLDTAAVK.V
2762	1261 - 1271	573.3301	1144.6456	1144.6452	0.36	2	57	6.5e-05	1	U	K.KKGDLDTAAVK.V
1646	1262 - 1271	509.2820	1016.5495	1016.5502	-0.66	1	31	0.05	1	U	K.KGDLDTAAVK.V
1647	1262 - 1271	509.2825	1016.5505	1016.5502	0.27	1	39	0.0041	1	U	K.KGDLDTAAVK.V
1648	1262 - 1271	509.2825	1016.5505	1016.5502	0.29	1	42	0.0032	1	U	K.KGDLDTAAVK.V
1649	1262 - 1271	509.2826	1016.5506	1016.5502	0.42	1	53	0.00019	1	U	K.KGDLDTAAVK.V
1650	1262 - 1271	509.2826	1016.5507	1016.5502	0.50	1	58	8e-05	1	U	K.KGDLDTAAVK.V
1651	1262 - 1271	509.2828	1016.5511	1016.5502	0.86	1	53	0.00023	1	U	K.KGDLDTAAVK.V
834	1263 - 1271	445.2344	888.4542	888.4553	-1.19	0	50	0.00066	1	U	K.GDLDTAAVK.V
13271	1263 - 1299	947.9647	3787.8296	3787.8316	-0.54	1	29	0.022	1	U	K.GDLDTAAVKVEFDEEFSGAPVEGAGEEALTPSVPINK.G
11697	1272 - 1299	973.4682	2917.3826	2917.3869	-1.47	0	58	1.6e-05	1	U	K.VEFDEEFSGAPVEGAGEEALTPSVPINK.G
11701	1272 - 1299	973.4687	2917.3843	2917.3869	-0.90	0	45	0.0003	1	U	K.VEFDEEFSGAPVEGAGEEALTPSVPINK.G
11704	1272 - 1299	973.4691	2917.3854	2917.3869	-0.52	0	26	0.022	1	U	K.VEFDEEFSGAPVEGAGEEALTPSVPINK.G
11705	1272 - 1299	973.4691	2917.3854	2917.3869	-0.51	0	21	0.024	1	U	K.VEFDEEFSGAPVEGAGEEALTPSVPINK.G
11709	1272 - 1299	973.4693	2917.3860	2917.3869	-0.32	0	24	0.029	1	U	K.VEFDEEFSGAPVEGAGEEALTPSVPINK.G
11712	1272 - 1299	973.4694	2917.3865	2917.3869	-0.15	0	57	9.2e-05	1	U	K.VEFDEEFSGAPVEGAGEEALTPSVPINK.G
11716	1272 - 1299	973.4697	2917.3874	2917.3869	0.16	0	53	5.5e-05	1	U	K.VEFDEEFSGAPVEGAGEEALTPSVPINK.G
11717	1272 - 1299	973.4698	2917.3874	2917.3869	0.18	0	33	0.012	1	U	K.VEFDEEFSGAPVEGAGEEALTPSVPINK.G
11718	1272 - 1299	973.4698	2917.3876	2917.3869	0.24	0	44	0.00087	1	U	K.VEFDEEFSGAPVEGAGEEALTPSVPINK.G
11719	1272 - 1299	973.4699	2917.3878	2917.3869	0.29	0	36	0.0026	1	U	K.VEFDEEFSGAPVEGAGEEALTPSVPINK.G
11721	1272 - 1299	973.4700	2917.3881	2917.3869	0.41	0	26	0.036	1	U	K.VEFDEEFSGAPVEGAGEEALTPSVPINK.G
11724	1272 - 1299	973.4703	2917.3890	2917.3869	0.72	0	50	0.0001	1	U	K.VEFDEEFSGAPVEGAGEEALTPSVPINK.G
11730	1272 - 1299	973.4709	2917.3910	2917.3869	1.40	0	23	0.038	1	U	K.VEFDEEFSGAPVEGAGEEALTPSVPINK.G
11731	1272 - 1299	1459.7034	2917.3923	2917.3869	1.87	0	80	5.4e-07	1	U	K.VEFDEEFSGAPVEGAGEEALTPSVPINK.G
11732	1272 - 1299	973.4714	2917.3925	2917.3869	1.93	0	46	0.00076	1	U	K.VEFDEEFSGAPVEGAGEEALTPSVPINK.G
5865	1314 - 1327	508.6025	1522.7857	1522.7868	-0.74	2	20	0.027	1	U	R.VRKTPTSSGKPSAK.K + Phospho (ST)
3122	1316 - 1327	396.8909	1187.6508	1187.6510	-0.15	1	35	0.0061	1	U	R.KTPTSSGKPSAK.K
3124	1316 - 1327	594.8328	1187.6511	1187.6510	0.14	1	42	0.0025	1	U	R.KTPTSSGKPSAK.K
3125	1316 - 1327	594.8334	1187.6521	1187.6510	0.98	1	41	0.0016	1	U	R.KTPTSSGKPSAK.K
1990	1317 - 1327	354.1924	1059.5555	1059.5560	-0.48	0	26	0.042	1	U	K.TPTSSGKPSAK.K
1992	1317 - 1327	530.7853	1059.5561	1059.5560	0.055	0	34	0.0053	1	U	K.TPTSSGKPSAK.K
1993	1317 - 1327	530.7854	1059.5562	1059.5560	0.17	0	42	0.0017	1	U	K.TPTSSGKPSAK.K
1994	1317 - 1327	354.1927	1059.5563	1059.5560	0.23	0	31	0.013	1	U	K.TPTSSGKPSAK.K
3431	1332 - 1341	617.2786	1232.5426	1232.5422	0.38	1	33	0.0063	1	U	K.RNPWSDDESK.S
4109	1332 - 1341	657.2611	1312.5077	1312.5085	-0.58	1	29	0.0057	1	U	K.RNPWSDDESK.S + Phospho (ST)
11691	1332 - 1356	972.1285	2913.3636	2913.3628	0.26	2	81	8.2e-08	1	U	K.RNPWSDDESKSESDLEETEPVVIPR.D
12001	1332 - 1356	998.7823	2993.3251	2993.3291	-1.35	2	67	4.3e-06	1	U	K.RNPWSDDESKSESDLEETEPVVIPR.D + Phospho (ST)
12002	1332 - 1356	998.7832	2993.3278	2993.3291	-0.43	2	49	0.00033	1	U	K.RNPWSDDESKSESDLEETEPVVIPR.D + Phospho (ST)
12003	1332 - 1356	998.7844	2993.3314	2993.3291	0.76	2	52	0.00025	1	U	K.RNPWSDDESKSESDLEETEPVVIPR.D + Phospho (ST)
2135	1333 - 1341	539.2280	1076.4415	1076.4411	0.38	0	35	0.00058	1	U	R.NPWSDDESK.S
11396	1333 - 1356	920.0943	2757.2611	2757.2617	-0.22	1	49	0.00013	1	U	R.NPWSDDESKSESDLEETEPVVIPR.D
11397	1333 - 1356	690.3229	2757.2624	2757.2617	0.25	1	49	0.00022	1	U	R.NPWSDDESKSESDLEETEPVVIPR.D
11398	1333 - 1356	920.0948	2757.2627	2757.2617	0.35	1	29	0.008	1	U	R.NPWSDDESKSESDLEETEPVVIPR.D
11586	1333 - 1356	946.7492	2837.2258	2837.2280	-0.78	1	34	0.0099	1	U	R.NPWSDDESKSESDLEETEPVVIPR.D + Phospho (ST)
11587	1333 - 1356	946.7494	2837.2263	2837.2280	-0.60	1	44	0.0013	1	U	R.NPWSDDESKSESDLEETEPVVIPR.D + Phospho (ST)
11588	1333 - 1356	946.7506	2837.2301	2837.2280	0.72	1	60	7e-05	1	U	R.NPWSDDESKSESDLEETEPVVIPR.D + Phospho (ST)
7375	1342 - 1356	850.4221	1698.8296	1698.8312	-0.93	0	70	9.6e-06	1	U	K.SESDLEETEPVVIPR.D
7376	1342 - 1356	567.2839	1698.8298	1698.8312	-0.83	0	31	0.026	1	U	K.SESDLEETEPVVIPR.D
878	1362 - 1369	449.7647	897.5148	897.5144	0.49	1	25	0.044	1	U	R.RAAAERPK.Y

Query	Start – End	Observed	Mr(expt)	Mr(calc)	ppm	M	Score	Expect	Rank	U	Peptide
160	1363 – 1369	371.7141	741.4136	741.4133	0.42	0	35	0.0018	1	U	R.AAAERPK.Y
12534	1370 – 1396	1076.7362	3227.1867	3227.1858	0.26	0	45	0.00014	1	U	K.YTFDFSEEEDDDADDDDDDNNDLEELK.V
12535	1370 – 1396	1076.7363	3227.1870	3227.1858	0.37	0	45	0.00024	1	U	K.YTFDFSEEEDDDADDDDDDNNDLEELK.V
10543	1397 – 1418	805.0304	2412.0694	2412.0734	-1.65	1	41	0.00056	1	U	K.VKASPITNDGEDEFVPSDGLDK.D + Phospho (ST)
10544	1397 – 1418	805.0310	2412.0712	2412.0734	-0.89	1	39	0.001	1	U	K.VKASPITNDGEDEFVPSDGLDK.D + Phospho (ST)
12912	1397 – 1427	860.1374	3436.5205	3436.5236	-0.90	2	49	0.00032	1	U	K.VKASPITNDGEDEFVPSDGLDKDEYTFSPGK.S + Phospho (ST)
12913	1397 – 1427	860.1376	3436.5215	3436.5236	-0.60	2	39	0.0051	1	U	K.VKASPITNDGEDEFVPSDGLDKDEYTFSPGK.S + Phospho (ST)
12914	1397 – 1427	1146.5148	3436.5226	3436.5236	-0.28	2	67	2.7e-05	1	U	K.VKASPITNDGEDEFVPSDGLDKDEYTFSPGK.S + Phospho (ST)
12915	1397 – 1427	860.1379	3436.5226	3436.5236	-0.27	2	45	0.0014	1	U	K.VKASPITNDGEDEFVPSDGLDKDEYTFSPGK.S + Phospho (ST)
12916	1397 – 1427	860.1381	3436.5233	3436.5236	-0.077	2	54	0.00022	1	U	K.VKASPITNDGEDEFVPSDGLDKDEYTFSPGK.S + Phospho (ST)
12917	1397 – 1427	1146.5153	3436.5240	3436.5236	0.12	2	46	0.00081	1	U	K.VKASPITNDGEDEFVPSDGLDKDEYTFSPGK.S + Phospho (ST)
12918	1397 – 1427	860.1386	3436.5251	3436.5236	0.45	2	55	0.00015	1	U	K.VKASPITNDGEDEFVPSDGLDKDEYTFSPGK.S + Phospho (ST)
12919	1397 – 1427	860.1387	3436.5257	3436.5236	0.61	2	38	0.0042	1	U	K.VKASPITNDGEDEFVPSDGLDKDEYTFSPGK.S + Phospho (ST)
12920	1397 – 1427	860.1387	3436.5257	3436.5236	0.63	2	53	0.0002	1	U	K.VKASPITNDGEDEFVPSDGLDKDEYTFSPGK.S + Phospho (ST)
9717	1399 – 1418	1053.4788	2104.9431	2104.9437	-0.26	0	86	1.9e-07	1	U	K.ASPITNDGEDEFVPSDGLDK.D
12325	1399 – 1427	1044.1378	3129.3916	3129.3939	-0.71	1	80	1.5e-06	1	U	K.ASPITNDGEDEFVPSDGLDKDEYTFSPGK.S
12328	1399 – 1427	1044.1388	3129.3946	3129.3939	0.23	1	84	1.1e-07	1	U	K.ASPITNDGEDEFVPSDGLDKDEYTFSPGK.S
12499	1399 – 1427	1070.7929	3209.3568	3209.3602	-1.07	1	71	3.6e-06	1	U	K.ASPITNDGEDEFVPSDGLDKDEYTFSPGK.S + Phospho (ST)
12500	1399 – 1427	1070.7938	3209.3597	3209.3602	-0.16	1	86	6.3e-08	1	U	K.ASPITNDGEDEFVPSDGLDKDEYTFSPGK.S + Phospho (ST)
12501	1399 – 1427	1070.7943	3209.3610	3209.3602	0.25	1	28	0.024	1	U	K.ASPITNDGEDEFVPSDGLDKDEYTFSPGK.S + Phospho (ST)
1830	1419 – 1427	522.2374	1042.4601	1042.4607	-0.57	0	36	0.0016	1	U	K.DEYTFSPGK.S
9292	1440 – 1456	990.4790	1978.9435	1978.9425	0.50	1	100	2.3e-09	1	U	K.KSQDFGNLFSFPSYSQK.S
9293	1440 – 1456	660.6551	1978.9435	1978.9425	0.53	1	23	0.045	1	U	K.KSQDFGNLFSFPSYSQK.S
8575	1441 – 1456	926.4304	1850.8462	1850.8475	-0.73	0	83	7.5e-08	1	U	K.SQDFGNLFSFPSYSQK.S
8576	1441 – 1456	617.9560	1850.8462	1850.8475	-0.72	0	20	0.044	1	U	K.SQDFGNLFSFPSYSQK.S
8577	1441 – 1456	926.4306	1850.8467	1850.8475	-0.42	0	70	5.5e-07	1	U	K.SQDFGNLFSFPSYSQK.S
8578	1441 – 1456	926.4307	1850.8468	1850.8475	-0.39	0	63	3.5e-06	1	U	K.SQDFGNLFSFPSYSQK.S
8579	1441 – 1456	926.4307	1850.8469	1850.8475	-0.33	0	57	4.2e-06	1	U	K.SQDFGNLFSFPSYSQK.S
8581	1441 – 1456	617.9564	1850.8475	1850.8475	-0.0065	0	27	0.012	1	U	K.SQDFGNLFSFPSYSQK.S
8582	1441 – 1456	926.4311	1850.8476	1850.8475	0.040	0	48	3.3e-05	1	U	K.SQDFGNLFSFPSYSQK.S
8583	1441 – 1456	926.4312	1850.8479	1850.8475	0.19	0	75	1.5e-07	1	U	K.SQDFGNLFSFPSYSQK.S
8584	1441 – 1456	926.4312	1850.8479	1850.8475	0.19	0	74	1.1e-07	1	U	K.SQDFGNLFSFPSYSQK.S
8585	1441 – 1456	926.4314	1850.8482	1850.8475	0.36	0	92	5.5e-09	1	U	K.SQDFGNLFSFPSYSQK.S
8586	1441 – 1456	926.4314	1850.8482	1850.8475	0.39	0	74	2e-07	1	U	K.SQDFGNLFSFPSYSQK.S
8587	1441 – 1456	926.4314	1850.8483	1850.8475	0.41	0	49	4.1e-05	1	U	K.SQDFGNLFSFPSYSQK.S
8588	1441 – 1456	617.9567	1850.8483	1850.8475	0.43	0	53	7.4e-05	1	U	K.SQDFGNLFSFPSYSQK.S
8589	1441 – 1456	926.4316	1850.8486	1850.8475	0.60	0	74	1.1e-07	1	U	K.SQDFGNLFSFPSYSQK.S
8590	1441 – 1456	926.4317	1850.8489	1850.8475	0.74	0	74	1.2e-07	1	U	K.SQDFGNLFSFPSYSQK.S
8592	1441 – 1456	926.4325	1850.8504	1850.8475	1.55	0	20	0.015	1	U	K.SQDFGNLFSFPSYSQK.S
8593	1441 – 1456	926.4330	1850.8514	1850.8475	2.11	0	27	0.0028	1	U	K.SQDFGNLFSFPSYSQK.S
11450	1457 – 1482	932.4085	2794.2036	2794.2093	-2.06	1	68	1.5e-06	1	U	K.SEDDSAKFDSNEEDSASVFSPSFGLK.Q
11452	1457 – 1482	932.4098	2794.2076	2794.2093	-0.63	1	53	9.8e-05	1	U	K.SEDDSAKFDSNEEDSASVFSPSFGLK.Q
11453	1457 – 1482	932.4101	2794.2084	2794.2093	-0.34	1	56	3.1e-05	1	U	K.SEDDSAKFDSNEEDSASVFSPSFGLK.Q
11455	1457 – 1482	932.4104	2794.2094	2794.2093	0.024	1	49	0.00037	1	U	K.SEDDSAKFDSNEEDSASVFSPSFGLK.Q
11457	1457 – 1482	932.4107	2794.2102	2794.2093	0.31	1	45	0.00034	1	U	K.SEDDSAKFDSNEEDSASVFSPSFGLK.Q
11458	1457 – 1482	932.4107	2794.2102	2794.2093	0.31	1	73	1.1e-06	1	U	K.SEDDSAKFDSNEEDSASVFSPSFGLK.Q
11459	1457 – 1482	699.5598	2794.2102	2794.2093	0.32	1	39	0.0062	1	U	K.SEDDSAKFDSNEEDSASVFSPSFGLK.Q
11461	1457 – 1482	932.4108	2794.2106	2794.2093	0.44	1	38	0.0014	1	U	K.SEDDSAKFDSNEEDSASVFSPSFGLK.Q
11462	1457 – 1482	932.4108	2794.2107	2794.2093	0.50	1	65	5.9e-06	1	U	K.SEDDSAKFDSNEEDSASVFSPSFGLK.Q
11463	1457 – 1482	932.4111	2794.2115	2794.2093	0.76	1	21	0.042	1	U	K.SEDDSAKFDSNEEDSASVFSPSFGLK.Q
11464	1457 – 1482	932.4114	2794.2124	2794.2093	1.09	1	35	0.0045	1	U	K.SEDDSAKFDSNEEDSASVFSPSFGLK.Q
11651	1457 – 1482	959.0660	2874.1761	2874.1757	0.17	1	42	0.00074	1	U	K.SEDDSAKFDSNEEDSASVFSPSFGLK.Q + Phospho (ST)
9547	1464 – 1482	1031.9639	2061.9132	2061.9167	-1.70	0	66	1.5e-06	1	U	K.FDSNEEDSASVFSPSFGLK.Q
9548	1464 – 1482	1031.9641	2061.9136	2061.9167	-1.53	0	28	0.011	1	U	K.FDSNEEDSASVFSPSFGLK.Q
9549	1464 – 1482	1031.9642	2061.9139	2061.9167	-1.36	0	101	1.7e-09	1	U	K.FDSNEEDSASVFSPSFGLK.Q
9550	1464 – 1482	1031.9647	2061.9148	2061.9167	-0.94	0	134	6.6e-13	1	U	K.FDSNEEDSASVFSPSFGLK.Q
9551	1464 – 1482	688.3123	2061.9150	2061.9167	-0.85	0	27	0.016	1	U	K.FDSNEEDSASVFSPSFGLK.Q
9552	1464 – 1482	1031.9648	2061.9150	2061.9167	-0.83	0	109	2.7e-10	1	U	K.FDSNEEDSASVFSPSFGLK.Q
9553	1464 – 1482	1031.9649	2061.9152	2061.9167	-0.75	0	122	9.3e-12	1	U	K.FDSNEEDSASVFSPSFGLK.Q
9554	1464 – 1482	1031.9651	2061.9156	2061.9167	-0.56	0	53	0.00018	1	U	K.FDSNEEDSASVFSPSFGLK.Q
9555	1464 – 1482	1031.9652	2061.9158	2061.9167	-0.46	0	31	0.0036	1	U	K.FDSNEEDSASVFSPSFGLK.Q
9556	1464 – 1482	1031.9652	2061.9158	2061.9167	-0.43	0	101	5e-09	1	U	K.FDSNEEDSASVFSPSFGLK.Q
9557	1464 – 1482	688.3126	2061.9160	2061.9167	-0.37	0	22	0.024	1	U	K.FDSNEEDSASVFSPSFGLK.Q
9558	1464 – 1482	1031.9654	2061.9162	2061.9167	-0.24	0	31	0.019	1	U	K.FDSNEEDSASVFSPSFGLK.Q
9559	1464 – 1482	1031.9655	2061.9165	2061.9167	-0.12	0	63	1.6e-05	1	U	K.FDSNEEDSASVFSPSFGLK.Q
9560	1464 – 1482	1031.9657	2061.9169	2061.9167	0.075	0	76	4.8e-07	1	U	K.FDSNEEDSASVFSPSFGLK.Q
9561	1464 – 1482	1031.9658	2061.9170	2061.9167	0.12	0	115	6.1e-11	1	U	K.FDSNEEDSASVFSPSFGLK.Q
9562	1464 – 1482	688.3130	2061.9172	2061.9167	0.24	0	49	0.00013	1	U	K.FDSNEEDSASVFSPSFGLK.Q
9564	1464 – 1482	1031.9659	2061.9173	2061.9167	0.27	0	47	0.00022	1	U	K.FDSNEEDSASVFSPSFGLK.Q
9566	1464 – 1482	1031.9660	2061.9175	2061.9167	0.39	0	101	9.7e-10	1	U	K.FDSNEEDSASVFSPSFGLK.Q
9567	1464 – 1482	1031.9662	2061.9177	2061.9167	0.49	0	45	0.0001	1	U	K.FDSNEEDSASVFSPSFGLK.Q
9568	1464 – 1482	1031.9665	2061.9185	2061.9167	0.84	0	53	9.8e-05	1	U	K.FDSNEEDSASVFSPSFGLK.Q
9570	1464 – 1482	1031.9670	2061.9195	2061.9167	1.33	0	25	0.044	1	U	K.FDSNEEDSASVFSPSFGLK.Q
9571	1464 – 1482	1031.9677	2061.9209	2061.9167	2.01	0	40	0.00035	1	U	K.FDSNEEDSASVFSPSFGLK.Q
9572	1464 – 1482	1031.9680	2061.9215	2061.9167	2.33	0	38	0.00098	1	U	K.FDSNEEDSASVFSPSFGLK.Q
9573	1464 – 1482	1031.9684	2061.9221	2061.9167	2.63	0	33	0.0012	1	U	K.FDSNEEDSASVFSPSFGLK.Q
9836	1464 – 1482	1071.9477	2141.8809	2141.8831	-1.00	0	51	0.00019	1	U	K.FDSNEEDSASVFSPSFGLK.Q + Phospho (ST)
9837	1464 – 1482	1071.9478	2141.8809	2141.8831	-0.98	0	71	4e-07	1	U	K.FDSNEEDSASVFSPSFGLK.Q

Query	Start - End	Observed	Mr(expt)	Mr(calc)	ppm	M	Score	Expect	Rank	U	Peptide
											+ Phospho (ST)
9838	1464 - 1482	1071.9482	2141.8819	2141.8831	-0.56	0	51	9.1e-05	1	U	K.FDSNEEDSASVFSPSFGLK.Q
											+ Phospho (ST)
4493	1496 - 1508	684.8952	1367.7759	1367.7773	-1.01	1	55	2e-05	1	U	K.KGKPSSDTVPKPK.R
4494	1496 - 1508	456.9331	1367.7776	1367.7773	0.25	1	54	4e-05	1	U	K.KGKPSSDTVPKPK.R
5075	1496 - 1508	483.5890	1447.7450	1447.7436	1.00	1	32	0.045	1	U	K.KGKPSSDTVPKPK.R
											+ Phospho (ST)
3493	1497 - 1508	414.2343	1239.6810	1239.6823	-1.08	0	26	0.024	1	U	K.GKPSSDTVPKPK.R
3496	1497 - 1508	414.2346	1239.6819	1239.6823	-0.36	0	15	0.045	1	U	K.GKPSSDTVPKPK.R
3498	1497 - 1508	414.2346	1239.6820	1239.6823	-0.21	0	22	0.025	1	U	K.GKPSSDTVPKPK.R
3499	1497 - 1508	414.2346	1239.6821	1239.6823	-0.14	0	47	0.00019	1	U	K.GKPSSDTVPKPK.R
3501	1497 - 1508	620.8488	1239.6829	1239.6823	0.53	0	38	0.0024	1	U	K.GKPSSDTVPKPK.R
3502	1497 - 1508	414.2350	1239.6830	1239.6823	0.58	0	30	0.0023	1	U	K.GKPSSDTVPKPK.R
9023	1515 - 1532	640.9896	1919.9471	1919.9476	-0.28	1	59	4.6e-05	1	U	K.KVVEAVNSDSDSEFGIPK.K
9025	1515 - 1532	960.9810	1919.9475	1919.9476	-0.085	1	83	1.9e-08	1	U	K.KVVEAVNSDSDSEFGIPK.K
9344	1515 - 1532	1000.9642	1999.9139	1999.9140	-0.014	1	67	3e-06	1	U	K.KVVEAVNSDSDSEFGIPK.K
											+ Phospho (ST)
9345	1515 - 1532	667.6453	1999.9141	1999.9140	0.073	1	30	0.01	1	U	K.KVVEAVNSDSDSEFGIPK.K
											+ Phospho (ST)
9346	1515 - 1532	667.6455	1999.9148	1999.9140	0.40	1	24	0.039	1	U	K.KVVEAVNSDSDSEFGIPK.K
											+ Phospho (ST)
9347	1515 - 1532	1000.9647	1999.9148	1999.9140	0.41	1	48	0.00019	1	U	K.KVVEAVNSDSDSEFGIPK.K
											+ Phospho (ST)
9348	1515 - 1532	667.6457	1999.9153	1999.9140	0.67	1	24	0.039	1	U	K.KVVEAVNSDSDSEFGIPK.K
											+ Phospho (ST)
9349	1515 - 1532	667.6458	1999.9157	1999.9140	0.85	1	21	0.048	1	U	K.KVVEAVNSDSDSEFGIPK.K
											+ Phospho (ST)
9350	1515 - 1532	1000.9654	1999.9162	1999.9140	1.15	1	55	2.4e-05	1	U	K.KVVEAVNSDSDSEFGIPK.K
											+ Phospho (ST)
8113	1516 - 1532	896.9322	1791.8498	1791.8527	-1.61	0	47	8e-05	1	U	K.VVEAVNSDSDSEFGIPK.K
8114	1516 - 1532	896.9333	1791.8521	1791.8527	-0.33	0	63	2.1e-06	1	U	K.VVEAVNSDSDSEFGIPK.K
8115	1516 - 1532	896.9335	1791.8524	1791.8527	-0.15	0	67	7.7e-07	1	U	K.VVEAVNSDSDSEFGIPK.K
8699	1516 - 1532	936.9169	1871.8192	1871.8190	0.11	0	42	0.00014	1	U	K.VVEAVNSDSDSEFGIPK.K
											+ Phospho (ST)
8700	1516 - 1532	936.9170	1871.8195	1871.8190	0.28	0	71	2.6e-07	1	U	K.VVEAVNSDSDSEFGIPK.K
											+ Phospho (ST)
9024	1516 - 1533	640.9897	1919.9473	1919.9476	-0.19	1	28	0.021	1	U	K.VVEAVNSDSDSEFGIPKK.T
8336	1547 - 1562	606.2589	1815.7549	1815.7537	0.66	2	53	0.0002	1	U	K.RKASGSENEGDYNPGR.K
											+ Phospho (ST)
9138	1547 - 1563	648.9568	1943.8485	1943.8486	-0.062	3	29	0.01	1	U	K.RKASGSENEGDYNPGRK.T
											+ Phospho (ST)
6455	1548 - 1562	790.8502	1579.6858	1579.6862	-0.25	1	30	0.0031	1	U	R.KASGSENEGDYNPGR.K
7019	1548 - 1562	830.8336	1659.6526	1659.6526	-0.0030	1	42	0.00061	1	U	R.KASGSENEGDYNPGR.K
											+ Phospho (ST)
7022	1548 - 1562	554.2251	1659.6535	1659.6526	0.54	1	25	0.024	1	U	R.KASGSENEGDYNPGR.K
											+ Phospho (ST)
7024	1548 - 1562	830.8350	1659.6554	1659.6526	1.70	1	44	0.00075	1	U	R.KASGSENEGDYNPGR.K
											+ Phospho (ST)
7702	1548 - 1562	870.8171	1739.6197	1739.6189	0.47	1	29	0.0089	1	U	R.KASGSENEGDYNPGR.K
											+ 2 Phospho (ST)
7432	1548 - 1563	570.2674	1707.7805	1707.7812	-0.40	2	46	0.00059	1	U	R.KASGSENEGDYNPGRK.T
7433	1548 - 1563	570.2676	1707.7811	1707.7812	-0.066	2	53	9.6e-05	1	U	R.KASGSENEGDYNPGRK.T
7434	1548 - 1563	854.8983	1707.7821	1707.7812	0.51	2	52	8.6e-05	1	U	R.KASGSENEGDYNPGRK.T
7435	1548 - 1563	570.2681	1707.7825	1707.7812	0.78	2	27	0.049	1	U	R.KASGSENEGDYNPGRK.T
8045	1548 - 1563	596.9235	1787.7487	1787.7475	0.66	2	44	0.0003	1	U	R.KASGSENEGDYNPGRK.T
											+ Phospho (ST)
5099	1549 - 1562	726.8024	1451.5902	1451.5913	-0.73	0	32	0.0043	1	U	K.ASGSENEGDYNPGR.K
5100	1549 - 1562	726.8028	1451.5910	1451.5913	-0.18	0	47	0.00032	1	U	K.ASGSENEGDYNPGR.K
5101	1549 - 1562	726.8028	1451.5911	1451.5913	-0.13	0	31	0.01	1	U	K.ASGSENEGDYNPGR.K
5102	1549 - 1562	484.8712	1451.5919	1451.5913	0.41	0	41	0.0016	1	U	K.ASGSENEGDYNPGR.K
5991	1549 - 1562	766.7861	1531.5576	1531.5576	-0.032	0	31	0.008	1	U	K.ASGSENEGDYNPGR.K
											+ Phospho (ST)
5992	1549 - 1562	766.7863	1531.5580	1531.5576	0.26	0	48	0.00022	1	U	K.ASGSENEGDYNPGR.K
											+ Phospho (ST)
5993	1549 - 1562	766.7865	1531.5585	1531.5576	0.58	0	53	0.00012	1	U	K.ASGSENEGDYNPGR.K
											+ Phospho (ST)
6453	1549 - 1563	790.8501	1579.6856	1579.6862	-0.41	1	62	2.5e-05	1	U	K.ASGSENEGDYNPGRK.T
6454	1549 - 1563	527.5692	1579.6858	1579.6862	-0.30	1	32	0.026	1	U	K.ASGSENEGDYNPGRK.T
7021	1549 - 1563	830.8338	1659.6530	1659.6526	0.27	1	62	3.6e-05	1	U	K.ASGSENEGDYNPGRK.T
											+ Phospho (ST)
7023	1549 - 1563	554.2252	1659.6536	1659.6526	0.63	1	40	0.00062	1	U	K.ASGSENEGDYNPGRK.T
											+ Phospho (ST)
11780	1574 - 1599	979.7948	2936.3625	2936.3716	-3.12	1	36	0.0042	1	U	K.KTSFDQDSDVDIFPSDFPTEPPSLPR.T
11784	1574 - 1599	979.7963	2936.3672	2936.3716	-1.51	1	32	0.021	1	U	K.KTSFDQDSDVDIFPSDFPTEPPSLPR.T
11786	1574 - 1599	979.7969	2936.3689	2936.3716	-0.94	1	32	0.0035	1	U	K.KTSFDQDSDVDIFPSDFPTEPPSLPR.T
11787	1574 - 1599	979.7970	2936.3692	2936.3716	-0.83	1	54	0.0001	1	U	K.KTSFDQDSDVDIFPSDFPTEPPSLPR.T
11788	1574 - 1599	979.7971	2936.3695	2936.3716	-0.73	1	27	0.018	1	U	K.KTSFDQDSDVDIFPSDFPTEPPSLPR.T
11790	1574 - 1599	979.7974	2936.3703	2936.3716	-0.47	1	40	0.0045	1	U	K.KTSFDQDSDVDIFPSDFPTEPPSLPR.T
11791	1574 - 1599	588.2814	2936.3705	2936.3716	-0.39	1	29	0.014	1	U	K.KTSFDQDSDVDIFPSDFPTEPPSLPR.T
11794	1574 - 1599	979.7978	2936.3714	2936.3716	-0.070	1	26	0.046	1	U	K.KTSFDQDSDVDIFPSDFPTEPPSLPR.T
11795	1574 - 1599	735.1002	2936.3715	2936.3716	-0.045	1	45	0.0014	1	U	K.KTSFDQDSDVDIFPSDFPTEPPSLPR.T
11796	1574 - 1599	979.7978	2936.3717	2936.3716	0.032	1	49	0.0006	1	U	K.KTSFDQDSDVDIFPSDFPTEPPSLPR.T
11797	1574 - 1599	979.7979	2936.3720	2936.3716	0.11	1	61	1.7e-05	1	U	K.KTSFDQDSDVDIFPSDFPTEPPSLPR.T
11498	1575 - 1599	937.0990	2808.2752	2808.2767	-0.52	0	21	0.041	1	U	K.TSFDQDSDVDIFPSDFPTEPPSLPR.T
11499	1575 - 1599	937.0992	2808.2756	2808.2767	-0.37	0	28	0.022	1	U	K.TSFDQDSDVDIFPSDFPTEPPSLPR.T
11503	1575 - 1599	937.1001	2808.2785	2808.2767	0.66	0	27	0.012	1	U	K.TSFDQDSDVDIFPSDFPTEPPSLPR.T
11504	1575 - 1599	937.1002	2808.2789	2808.2767	0.79	0	27	0.019	1	U	K.TSFDQDSDVDIFPSDFPTEPPSLPR.T
9925	1609 - 1626	1086.9135	2171.8124	2171.8154	-1.36	0	29	0.0044	1	U	K.YFAESDEEEDDVDFAMFN.-
9933	1609 - 1626	1086.9148	2171.8151	2171.8154	-0.12	0	33	0.00077	1	U	K.YFAESDEEEDDVDFAMFN.-
9935	1609 - 1626	724.9461	2171.8164	2171.8154	0.46	0	41	0.00015	1	U	K.YFAESDEEEDDVDFAMFN.-
9975	1609 - 1626	1094.9108	2187.8071	2187.8103	-1.45	0	79	6.2e-08	1	U	K.YFAESDEEEDDVDFAMFN.-
											+ Oxidation (M)
9976	1609 - 1626	1094.9118	2187.8091	2187.8103	-0.54	0	53	1.7e-05	1	U	K.YFAESDEEEDDVDFAMFN.-
											+ Oxidation (M)
9977	1609 - 1626	1094.9121	2187.8096	2187.8103	-0.32	0	40	0.00039	1	U	K.YFAESDEEEDDVDFAMFN.-

Query	Start – End	Observed	Mr(expt)	Mr(calc)	ppm	M	Score	Expect	Rank	U	Peptide
											+ Oxidation (M)
9979	1609 – 1626	1094.9126	2187.8107	2187.8103	0.18	0	65	8.4e-07	1	U	K.YFAESDEEEDDVDFAMFN.- + Oxidation (M)
9981	1609 – 1626	1094.9132	2187.8119	2187.8103	0.76	0	17	0.028	1	U	K.YFAESDEEEDDVDFAMFN.- + Oxidation (M)

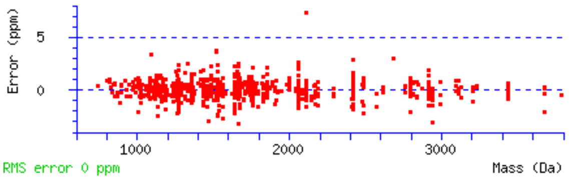

Supplement: Supplementary file 5 — Supplementary Data 2 [file 41467_2023_44089_MOESM5_ESM.pdf]
