# Supplementary material for: ERK2-topoisomerase II regulatory axis is important for gene activation in immediate early genes
Source: Nat Commun. 2023 Dec 14;14:8341. doi: 10.1038/s41467-023-44089-y (PMC10721843; doi:10.1038/s41467-023-44089-y)

MASCOT Search Results

Protein View: Q02880

DNA topoisomerase 2-beta OS=Homo sapiens OX=9606 GN=TOP2B PE=1 SV=3

**Database:**	Uniprot_Proteome_Human
**Score:**	19255
**Monoisotopic mass (M_r):**	184122
**Calculated pI:**	8.14

Sequence similarity is available as **an NCBI BLAST search of Q02880 against nr**.

Search parameters

**MS data file:**	File Name: Z:\SN22\SN221364_deTOP2B_Erk2_1ul.raw;File Path: ;File Time: 3/1/2023 12:45:59 PM;File Size: 347857146 [Byte]
**Enzyme:**	Trypsin: cuts C-term side of KR unless next residue is P.
**Fixed modifications:**	**Carbamidomethyl (C)**
**Variable modifications:**	**Acetyl (Protein N-term)**, **Oxidation (M)**, **Phospho (ST)**, **Phospho (Y)**

Protein sequence coverage: 62%

Matched peptides shown in ***bold red***.

```
   1 MAKSGGCGAG AGVGGGNGAL TWVTLFDQNN AAKKEESETA NKNDSSKKLS
  51 VERVYQKKTQ LEHILLRPDT YIGSVEPLTQ FMWVYDEDVG MNCREVTFVP
 101 GLYKIFDEIL VNAADNKQRD KNMTCIKVSI DPESNIISIW NNGKGIPVVE
 151 HKVEKVYVPA LIFGQLLTSS NYDDDEKKVT GGRNGYGAKL CNIFSTKFTV
 201 ETACKEYKHS FKQTWMNNMM KTSEAKIKHF DGEDYTCITF QPDLSKFKME
 251 KLDKDIVALM TRRAYDLAGS CRGVKVMFNG KKLPVNGFRS YVDLYVKDKL
 301 DETGVALKVI HELANERWDV CLTLSEKGFQ QISFVNSIAT TKGGRHVDYV
 351 VDQVVGKLIE VVKKKNKAGV SVKPFQVKNH IWVFINCLIE NPTFDSQTKE
 401 NMTLQPKSFG SKCQLSEKFF KAASNCGIVE SILNWVKFKA QTQLNKKCSS
 451 VKYSKIKGIP KLDDANDAGG KHSLECTLIL TEGDSAKSLA VSGLGVIGRD
 501 RYGVFPLRGK ILNVREASHK QIMENAEINN IIKIVGLQYK KSYDDAESLK
 551 TLRYGKIMIM TDQDQDGSHI KGLLINFIHH NWPSLLKHGF LEEFITPIVK
 601 ASKNKQELSF YSIPEFDEWK KHIENQKAWK IKYYKGLGTS TAKEAKEYFA
 651 DMERHRILFR YAGPEDDAAI TLAFSKKKID DRKEWLTNFM EDRRQRRLHG
 701 LPEQFLYGTA TKHLTYNDFI NKELILFSNS DNERSIPSLV DGFKPGQRKV
 751 LFTCFKRNDK REVKVAQLAG SVAEMSAYHH GEQALMMTIV NLAQNFVGSN
 801 NINLLQPIGQ FGTRLHGGKD AASPRYIFTM LSTLARLLFP AVDDNLLKFL
 851 YDDNQRVEPE WYIPIIPMVL INGAEGIGTG WACKLPNYDA REIVNNVRRM
 901 LDGLDPHPML PNYKNFKGTI QELGQNQYAV SGEIFVVDRN TVEITELPVR
 951 TWTQVYKEQV LEPMLNGTDK TPALISDYKE YHTDTTVKFV VKMTEEKLAQ
1001 AEAAGLHKVF KLQTTLTCNS MVLFDHMGCL KKYETVQDIL KEFFDLRLSY
1051 YGLRKEWLVG MLGAESTKLN NQARFILEKI QGKITIENRS KKDLIQMLVQ
1101 RGYESDPVKA WKEAQEKAAE EDETQNQHDD SSSDSGTPSG PDFNYILNMS
1151 LWSLTKEKVE ELIKQRDAKG REVNDLKRKS PSDLWKEDLA AFVEELDKVE
1201 SQEREDVLAG MSGKAIKGKV GKPKVKKLQL EETMPSPYGR RIIPEITAMK
1251 ADASKKLLKK KKGDLDTAAV KVEFDEEFSG APVEGAGEEA LTPSVPINKG
1301 PKPKREKKEP GTRVRKTPTS SGKPSAKKVK KRNPWSDDES KSESDLEETE
1351 PVVIPRDSLL RRAAAERPKY TFDFSEEEDD DADDDDDDNN DLEELKVKAS
1401 PITNDGEDEF VPSDGLDKDE YTFSPGKSKA TPEKSLHDKK SQDFGNLFSF
1451 PSYSQKSEDD SAKFDSNEED SASVFSPSFG LKQTDKVPSK TVAAKKGKPS
1501 SDTVPKPKRA PKQKKVVEAV NSDSDSEFGI PKKTTTPKGK GRGAKKRKAS
1551 GSENEGDYNP GRKTSKTTSK KPKKTSFDQD SDVDIFPSDF PTEPPSLPRT
1601 GRARKEVKYF AESDEEEDDV DFAMFN
```

Unformatted sequence string: **1626 residues** (for pasting into other applications).

Sort by (●) residue number ( ) increasing mass ( ) decreasing mass
Show (●) matched peptides only ( ) predicted peptides also

Query	Start – End	Observed	Mr(expt)	Mr(calc)	ppm	M	Score	Expect	Rank	U	Peptide
1755	34 – 42	518.2515	1034.4884	1034.4880	0.46	1	37	0.012	1	U	K.KEESETANK.N
6141	34 – 47	522.9126	1565.7159	1565.7169	-0.62	2	24	0.02	1	U	K.KEESETANKNDSSK.K
6142	34 – 47	783.8665	1565.7184	1565.7169	0.96	2	55	4.1e-05	1	U	K.KEESETANKNDSSK.K
6143	34 – 47	783.8665	1565.7184	1565.7169	0.96	2	54	2.6e-05	1	U	K.KEESETANKNDSSK.K
7142	34 – 48	565.6111	1693.8114	1693.8118	-0.27	3	25	0.017	1	U	K.KEESETANKNDSSKK.L
7143	34 – 48	565.6115	1693.8127	1693.8118	0.51	3	34	0.0022	1	U	K.KEESETANKNDSSKK.L
2777	95 – 104	576.8185	1151.6225	1151.6227	-0.15	0	14	0.045	1		R.EVTFVPGLYK.I
2779	95 – 104	576.8187	1151.6228	1151.6227	0.11	0	18	0.026	1		R.EVTFVPGLYK.I
2780	95 – 104	576.8188	1151.6230	1151.6227	0.30	0	19	0.026	1		R.EVTFVPGLYK.I
2781	95 – 104	576.8188	1151.6230	1151.6227	0.32	0	21	0.018	1		R.EVTFVPGLYK.I
5070	105 – 117	487.9233	1460.7482	1460.7511	-2.00	0	30	0.005	1		K.IFDEILVNAADNK.Q
5071	105 – 117	731.3821	1460.7496	1460.7511	-0.98	0	50	0.00025	1		K.IFDEILVNAADNK.Q
5072	105 – 117	487.9240	1460.7502	1460.7511	-0.62	0	46	0.00075	1		K.IFDEILVNAADNK.Q
5073	105 – 117	731.3824	1460.7502	1460.7511	-0.61	0	51	0.00011	1		K.IFDEILVNAADNK.Q
5075	105 – 117	731.3824	1460.7503	1460.7511	-0.56	0	68	3.1e-06	1		K.IFDEILVNAADNK.Q

Query	Start - End	Observed	Mr(expt)	Mr(calc)	ppm	M	Score	Expect	Rank	U	Peptide
5076	105 - 117	731.3825	1460.7505	1460.7511	-0.41	0	70	2.6e-06	1		K.IFDEILVNAADNK.Q
5079	105 - 117	731.3826	1460.7507	1460.7511	-0.28	0	71	1.6e-06	1		K.IFDEILVNAADNK.Q
5080	105 - 117	731.3826	1460.7507	1460.7511	-0.24	0	53	0.00011	1		K.IFDEILVNAADNK.Q
5081	105 - 117	487.9242	1460.7507	1460.7511	-0.23	0	33	0.0088	1		K.IFDEILVNAADNK.Q
5082	105 - 117	487.9242	1460.7507	1460.7511	-0.23	0	37	0.006	1		K.IFDEILVNAADNK.Q
5083	105 - 117	487.9242	1460.7507	1460.7511	-0.23	0	44	0.001	1		K.IFDEILVNAADNK.Q
5084	105 - 117	731.3826	1460.7507	1460.7511	-0.23	0	39	0.001	1		K.IFDEILVNAADNK.Q
5085	105 - 117	487.9242	1460.7508	1460.7511	-0.19	0	37	0.0023	1		K.IFDEILVNAADNK.Q
5086	105 - 117	731.3827	1460.7509	1460.7511	-0.13	0	75	2.4e-06	1		K.IFDEILVNAADNK.Q
5087	105 - 117	487.9243	1460.7510	1460.7511	-0.090	0	25	0.043	1		K.IFDEILVNAADNK.Q
5088	105 - 117	487.9243	1460.7510	1460.7511	-0.028	0	31	0.021	1		K.IFDEILVNAADNK.Q
5090	105 - 117	731.3828	1460.7511	1460.7511	0.031	0	37	0.003	1		K.IFDEILVNAADNK.Q
5091	105 - 117	487.9243	1460.7512	1460.7511	0.075	0	34	0.0074	1		K.IFDEILVNAADNK.Q
5093	105 - 117	731.3829	1460.7513	1460.7511	0.14	0	88	9.5e-08	1		K.IFDEILVNAADNK.Q
5094	105 - 117	487.9244	1460.7513	1460.7511	0.16	0	48	0.00076	1		K.IFDEILVNAADNK.Q
5095	105 - 117	731.3829	1460.7513	1460.7511	0.17	0	73	7.1e-07	1		K.IFDEILVNAADNK.Q
5096	105 - 117	731.3830	1460.7514	1460.7511	0.24	0	83	7.4e-08	1		K.IFDEILVNAADNK.Q
5097	105 - 117	487.9244	1460.7514	1460.7511	0.24	0	31	0.011	1		K.IFDEILVNAADNK.Q
5098	105 - 117	731.3830	1460.7514	1460.7511	0.25	0	70	3.1e-06	1		K.IFDEILVNAADNK.Q
5101	105 - 117	487.9245	1460.7516	1460.7511	0.34	0	29	0.011	1		K.IFDEILVNAADNK.Q
5102	105 - 117	731.3831	1460.7516	1460.7511	0.37	0	70	3.4e-06	1		K.IFDEILVNAADNK.Q
5104	105 - 117	731.3832	1460.7519	1460.7511	0.56	0	30	0.0032	1		K.IFDEILVNAADNK.Q
5105	105 - 117	731.3832	1460.7519	1460.7511	0.58	0	29	0.016	1		K.IFDEILVNAADNK.Q
5106	105 - 117	731.3832	1460.7519	1460.7511	0.59	0	55	6.4e-05	1		K.IFDEILVNAADNK.Q
5107	105 - 117	731.3833	1460.7520	1460.7511	0.61	0	65	3.9e-06	1		K.IFDEILVNAADNK.Q
5108	105 - 117	731.3834	1460.7523	1460.7511	0.82	0	74	2e-06	1		K.IFDEILVNAADNK.Q
5109	105 - 117	731.3834	1460.7523	1460.7511	0.82	0	78	3.1e-07	1		K.IFDEILVNAADNK.Q
5110	105 - 117	731.3834	1460.7523	1460.7511	0.84	0	71	2.8e-06	1		K.IFDEILVNAADNK.Q
5111	105 - 117	487.9248	1460.7527	1460.7511	1.12	0	47	0.002	1		K.IFDEILVNAADNK.Q
5112	105 - 117	487.9249	1460.7528	1460.7511	1.20	0	24	0.025	1		K.IFDEILVNAADNK.Q
5113	105 - 117	731.3838	1460.7530	1460.7511	1.29	0	27	0.044	1		K.IFDEILVNAADNK.Q
1679	120 - 127	513.2409	1024.4672	1024.4681	-0.87	1	31	0.037	1	U	K.DKNMTCIK.V + Oxidation (M)
8513	128 - 144	943.4860	1884.9575	1884.9581	-0.31	0	59	1.2e-05	1	U	K.VSIDPESNIISIWNNGK.G
8514	128 - 144	629.3269	1884.9589	1884.9581	0.43	0	48	0.00011	1	U	K.VSIDPESNIISIWNNGK.G
10580	156 - 177	829.7476	2486.2209	2486.2217	-0.31	0	34	0.015	1	U	K.VYVPALIFGQLLTSSNYDDDEK.K
10583	156 - 177	829.7479	2486.2218	2486.2217	0.029	0	38	0.0016	1	U	K.VYVPALIFGQLLTSSNYDDDEK.K
10584	156 - 177	1244.1185	2486.2225	2486.2217	0.33	0	52	9.6e-05	1	U	K.VYVPALIFGQLLTSSNYDDDEK.K
10585	156 - 177	829.7482	2486.2227	2486.2217	0.39	0	60	3.4e-05	1	U	K.VYVPALIFGQLLTSSNYDDDEK.K
10586	156 - 177	1244.1190	2486.2235	2486.2217	0.72	0	58	8.9e-06	1	U	K.VYVPALIFGQLLTSSNYDDDEK.K
10587	156 - 177	1244.1190	2486.2235	2486.2217	0.73	0	87	5.4e-08	1	U	K.VYVPALIFGQLLTSSNYDDDEK.K
10588	156 - 177	1244.1192	2486.2238	2486.2217	0.86	0	86	2.8e-08	1	U	K.VYVPALIFGQLLTSSNYDDDEK.K
10589	156 - 177	1244.1195	2486.2245	2486.2217	1.14	0	70	5.9e-07	1	U	K.VYVPALIFGQLLTSSNYDDDEK.K
10591	156 - 177	1244.1240	2486.2335	2486.2217	4.74	0	80	8.6e-08	1	U	K.VYVPALIFGQLLTSSNYDDDEK.K
10839	156 - 178	872.4444	2614.3114	2614.3166	-1.99	1	43	0.0027	1	U	K.VYVPALIFGQLLTSSNYDDDEKK.V
10844	156 - 178	654.5864	2614.3165	2614.3166	-0.038	1	38	0.0046	1	U	K.VYVPALIFGQLLTSSNYDDDEKK.V
10846	156 - 178	872.4462	2614.3167	2614.3166	0.029	1	65	2.5e-05	1	U	K.VYVPALIFGQLLTSSNYDDDEKK.V
10847	156 - 178	872.4462	2614.3169	2614.3166	0.11	1	42	0.0027	1	U	K.VYVPALIFGQLLTSSNYDDDEKK.V
10849	156 - 178	872.4464	2614.3173	2614.3166	0.27	1	39	0.014	1	U	K.VYVPALIFGQLLTSSNYDDDEKK.V
10850	156 - 178	1308.1660	2614.3174	2614.3166	0.30	1	66	2.7e-05	1	U	K.VYVPALIFGQLLTSSNYDDDEKK.V
10853	156 - 178	654.5868	2614.3180	2614.3166	0.51	1	42	0.0074	1	U	K.VYVPALIFGQLLTSSNYDDDEKK.V
10855	156 - 178	654.5868	2614.3181	2614.3166	0.57	1	37	0.0029	1	U	K.VYVPALIFGQLLTSSNYDDDEKK.V
10856	156 - 178	872.4467	2614.3182	2614.3166	0.60	1	33	0.028	1	U	K.VYVPALIFGQLLTSSNYDDDEKK.V
1361	190 - 197	491.7550	981.4954	981.4953	0.043	0	46	0.00039	1		K.LCNIFSTK.F
3007	213 - 221	592.2567	1182.4988	1182.4984	0.33	0	26	0.028	1	U	K.QTWMNNMMK.T
3100	213 - 221	600.2539	1198.4932	1198.4933	-0.094	0	29	0.027	1	U	K.QTWMNNMMK.T + Oxidation (M)
3101	213 - 221	600.2541	1198.4936	1198.4933	0.26	0	26	0.016	1	U	K.QTWMNNMMK.T + Oxidation (M)
3102	213 - 221	600.2543	1198.4941	1198.4933	0.67	0	34	0.0022	1	U	K.QTWMNNMMK.T + Oxidation (M)
3199	213 - 221	608.2511	1214.4877	1214.4882	-0.44	0	15	0.048	1	U	K.QTWMNNMMK.T + 2 Oxidation (M)
3203	213 - 221	608.2515	1214.4884	1214.4882	0.15	0	19	0.048	1	U	K.QTWMNNMMK.T + 2 Oxidation (M)
3204	213 - 221	608.2515	1214.4885	1214.4882	0.25	0	25	0.043	1	U	K.QTWMNNMMK.T + 2 Oxidation (M)
9693	229 - 246	724.9894	2171.9465	2171.9470	-0.22	0	28	0.014	1	U	K.HFDGEDYTCITFQPDLSK.F
3692	252 - 262	637.8598	1273.7051	1273.7064	-1.04	1	38	0.0012	1	U	K.LDKDIVALMTR.R
3693	252 - 262	637.8601	1273.7057	1273.7064	-0.55	1	29	0.003	1	U	K.LDKDIVALMTR.R
3694	252 - 262	637.8601	1273.7057	1273.7064	-0.54	1	34	0.01	1	U	K.LDKDIVALMTR.R
3699	252 - 262	637.8605	1273.7064	1273.7064	-0.0016	1	35	0.012	1	U	K.LDKDIVALMTR.R
3700	252 - 262	637.8605	1273.7064	1273.7064	-0.0016	1	75	1.3e-06	1	U	K.LDKDIVALMTR.R
3701	252 - 262	425.5761	1273.7064	1273.7064	0.041	1	32	0.016	1	U	K.LDKDIVALMTR.R
3702	252 - 262	637.8605	1273.7064	1273.7064	0.046	1	32	0.013	1	U	K.LDKDIVALMTR.R
3705	252 - 262	637.8606	1273.7066	1273.7064	0.19	1	34	0.0057	1	U	K.LDKDIVALMTR.R
3706	252 - 262	637.8607	1273.7069	1273.7064	0.39	1	49	0.00013	1	U	K.LDKDIVALMTR.R
3707	252 - 262	637.8608	1273.7070	1273.7064	0.47	1	31	0.0054	1	U	K.LDKDIVALMTR.R
3708	252 - 262	637.8608	1273.7071	1273.7064	0.56	1	45	0.0003	1	U	K.LDKDIVALMTR.R
3821	252 - 262	645.8569	1289.6993	1289.7013	-1.54	1	46	0.00015	1	U	K.LDKDIVALMTR.R + Oxidation (M)
3824	252 - 262	645.8577	1289.7008	1289.7013	-0.42	1	33	0.0055	1	U	K.LDKDIVALMTR.R + Oxidation (M)
3825	252 - 262	645.8577	1289.7008	1289.7013	-0.39	1	32	0.002	1	U	K.LDKDIVALMTR.R + Oxidation (M)
3826	252 - 262	430.9076	1289.7009	1289.7013	-0.35	1	32	0.0071	1	U	K.LDKDIVALMTR.R + Oxidation (M)
3828	252 - 262	430.9076	1289.7009	1289.7013	-0.33	1	32	0.036	1	U	K.LDKDIVALMTR.R + Oxidation (M)
3829	252 - 262	645.8577	1289.7009	1289.7013	-0.33	1	39	0.0011	1	U	K.LDKDIVALMTR.R

Query	Start – End	Observed	Mr(expt)	Mr(calc)	ppm	M	Score	Expect	Rank	U	Peptide
											+ Oxidation (M)
3830	252 – 262	645.8577	1289.7009	1289.7013	-0.30	1	64	5.3e-06	1	U	K.LDKDIVALMTR.R + Oxidation (M)
3831	252 – 262	430.9076	1289.7011	1289.7013	-0.14	1	25	0.048	1	U	K.LDKDIVALMTR.R + Oxidation (M)
3833	252 – 262	430.9077	1289.7013	1289.7013	-0.029	1	23	0.019	1	U	K.LDKDIVALMTR.R + Oxidation (M)
3834	252 – 262	645.8581	1289.7016	1289.7013	0.25	1	62	1e-05	1	U	K.LDKDIVALMTR.R + Oxidation (M)
3835	252 – 262	430.9078	1289.7016	1289.7013	0.25	1	31	0.024	1	U	K.LDKDIVALMTR.R + Oxidation (M)
3836	252 – 262	645.8582	1289.7018	1289.7013	0.40	1	55	2.3e-05	1	U	K.LDKDIVALMTR.R + Oxidation (M)
938	255 – 262	459.7580	917.5015	917.5004	1.20	0	49	0.0002	1	U	K.DIVALMTR.R
1057	255 – 262	467.7551	933.4956	933.4953	0.28	0	38	0.0092	1	U	K.DIVALMTR.R + Oxidation (M)
1060	255 – 262	467.7553	933.4960	933.4953	0.75	0	37	0.012	1	U	K.DIVALMTR.R + Oxidation (M)
1581	264 – 272	506.7295	1011.4444	1011.4444	0.022	0	37	0.0009	1	U	R.AYDLAGSCR.G
1390	290 – 297	493.7635	985.5124	985.5120	0.35	0	23	0.043	1	U	R.SYVDLYVK.D
1391	290 – 297	493.7635	985.5125	985.5120	0.50	0	32	0.011	1	U	R.SYVDLYVK.D
1392	290 – 297	493.7638	985.5131	985.5120	1.06	0	41	0.00085	1	U	R.SYVDLYVK.D
3039	298 – 308	594.8269	1187.6393	1187.6398	-0.36	1	31	0.0075	1	U	K.DKLDETGVALK.V
3040	298 – 308	396.8871	1187.6394	1187.6398	-0.34	1	26	0.023	1	U	K.DKLDETGVALK.V
3041	298 – 308	396.8871	1187.6394	1187.6398	-0.31	1	27	0.038	1	U	K.DKLDETGVALK.V
3042	298 – 308	594.8270	1187.6394	1187.6398	-0.29	1	53	0.00016	1	U	K.DKLDETGVALK.V
3043	298 – 308	594.8270	1187.6395	1187.6398	-0.24	1	30	0.0099	1	U	K.DKLDETGVALK.V
1116	300 – 308	473.2668	944.5191	944.5179	1.31	0	56	0.00015	1	U	K.LDETGVALK.V
2123	309 – 317	540.7924	1079.5702	1079.5723	-1.98	0	31	0.015	1	U	K.VIHELANER.W
2124	309 – 317	540.7931	1079.5716	1079.5723	-0.67	0	19	0.024	1	U	K.VIHELANER.W
2125	309 – 317	540.7932	1079.5719	1079.5723	-0.43	0	26	0.0037	1	U	K.VIHELANER.W
2126	309 – 317	360.8646	1079.5720	1079.5723	-0.30	0	25	0.018	1	U	K.VIHELANER.W
2128	309 – 317	540.7933	1079.5721	1079.5723	-0.18	0	32	0.0019	1	U	K.VIHELANER.W
2131	309 – 317	540.7934	1079.5723	1079.5723	0.019	0	48	0.00064	1	U	K.VIHELANER.W
2132	309 – 317	540.7935	1079.5724	1079.5723	0.075	0	42	0.0022	1	U	K.VIHELANER.W
2133	309 – 317	540.7935	1079.5724	1079.5723	0.075	0	31	0.012	1	U	K.VIHELANER.W
2134	309 – 317	360.8648	1079.5725	1079.5723	0.14	0	30	0.02	1	U	K.VIHELANER.W
2135	309 – 317	360.8648	1079.5725	1079.5723	0.14	0	15	0.05	1	U	K.VIHELANER.W
2136	309 – 317	360.8648	1079.5726	1079.5723	0.28	0	29	0.0092	1	U	K.VIHELANER.W
2137	309 – 317	540.7936	1079.5727	1079.5723	0.32	0	46	0.00059	1	U	K.VIHELANER.W
2139	309 – 317	360.8649	1079.5728	1079.5723	0.39	0	44	0.0018	1	U	K.VIHELANER.W
2140	309 – 317	360.8650	1079.5731	1079.5723	0.67	0	25	0.042	1	U	K.VIHELANER.W
3468	318 – 327	625.8082	1249.6019	1249.6013	0.51	0	57	2.4e-05	1	U	R.WDVCLTLSEK.G
6635	328 – 342	547.6251	1639.8535	1639.8570	-2.10	0	26	0.0058	1	U	K.GFQQISFVNSIATTK.G
6636	328 – 342	820.9348	1639.8551	1639.8570	-1.13	0	37	0.0024	1	U	K.GFQQISFVNSIATTK.G
6637	328 – 342	820.9350	1639.8554	1639.8570	-0.96	0	60	4.3e-06	1	U	K.GFQQISFVNSIATTK.G
6638	328 – 342	820.9351	1639.8557	1639.8570	-0.76	0	49	3.1e-05	1	U	K.GFQQISFVNSIATTK.G
6639	328 – 342	820.9352	1639.8558	1639.8570	-0.73	0	60	1.7e-05	1	U	K.GFQQISFVNSIATTK.G
6640	328 – 342	820.9352	1639.8559	1639.8570	-0.65	0	49	0.00098	1	U	K.GFQQISFVNSIATTK.G
6641	328 – 342	820.9353	1639.8561	1639.8570	-0.56	0	30	0.0084	1	U	K.GFQQISFVNSIATTK.G
6642	328 – 342	820.9354	1639.8562	1639.8570	-0.48	0	72	5.4e-06	1	U	K.GFQQISFVNSIATTK.G
6643	328 – 342	820.9355	1639.8565	1639.8570	-0.31	0	39	0.00044	1	U	K.GFQQISFVNSIATTK.G
6644	328 – 342	820.9355	1639.8565	1639.8570	-0.29	0	46	5e-05	1	U	K.GFQQISFVNSIATTK.G
6645	328 – 342	547.6261	1639.8565	1639.8570	-0.27	0	38	0.00028	1	U	K.GFQQISFVNSIATTK.G
6646	328 – 342	820.9356	1639.8566	1639.8570	-0.25	0	52	0.00045	1	U	K.GFQQISFVNSIATTK.G
6647	328 – 342	820.9356	1639.8566	1639.8570	-0.23	0	51	0.0003	1	U	K.GFQQISFVNSIATTK.G
6648	328 – 342	820.9356	1639.8566	1639.8570	-0.20	0	57	0.00013	1	U	K.GFQQISFVNSIATTK.G
6649	328 – 342	820.9356	1639.8567	1639.8570	-0.19	0	32	0.0018	1	U	K.GFQQISFVNSIATTK.G
6650	328 – 342	820.9356	1639.8567	1639.8570	-0.17	0	68	1.3e-05	1	U	K.GFQQISFVNSIATTK.G
6652	328 – 342	820.9357	1639.8568	1639.8570	-0.12	0	39	0.0065	1	U	K.GFQQISFVNSIATTK.G
6653	328 – 342	820.9357	1639.8569	1639.8570	-0.068	0	38	0.00066	1	U	K.GFQQISFVNSIATTK.G
6654	328 – 342	547.6263	1639.8570	1639.8570	-0.016	0	53	2.5e-05	1	U	K.GFQQISFVNSIATTK.G
6655	328 – 342	820.9358	1639.8571	1639.8570	0.079	0	47	0.00024	1	U	K.GFQQISFVNSIATTK.G
6656	328 – 342	820.9358	1639.8571	1639.8570	0.091	0	50	4.9e-05	1	U	K.GFQQISFVNSIATTK.G
6657	328 – 342	547.6263	1639.8572	1639.8570	0.13	0	41	0.00057	1	U	K.GFQQISFVNSIATTK.G
6658	328 – 342	820.9359	1639.8572	1639.8570	0.15	0	67	2.9e-06	1	U	K.GFQQISFVNSIATTK.G
6659	328 – 342	820.9359	1639.8572	1639.8570	0.16	0	75	2.3e-06	1	U	K.GFQQISFVNSIATTK.G
6660	328 – 342	547.6264	1639.8573	1639.8570	0.18	0	53	6.3e-05	1	U	K.GFQQISFVNSIATTK.G
6661	328 – 342	547.6264	1639.8573	1639.8570	0.18	0	50	0.00013	1	U	K.GFQQISFVNSIATTK.G
6662	328 – 342	820.9359	1639.8573	1639.8570	0.19	0	69	4.6e-06	1	U	K.GFQQISFVNSIATTK.G
6663	328 – 342	820.9359	1639.8573	1639.8570	0.20	0	42	0.0048	1	U	K.GFQQISFVNSIATTK.G
6665	328 – 342	547.6264	1639.8575	1639.8570	0.33	0	39	0.00085	1	U	K.GFQQISFVNSIATTK.G
6666	328 – 342	547.6265	1639.8576	1639.8570	0.37	0	52	4.6e-05	1	U	K.GFQQISFVNSIATTK.G
6667	328 – 342	820.9361	1639.8577	1639.8570	0.42	0	96	1.7e-08	1	U	K.GFQQISFVNSIATTK.G
6668	328 – 342	820.9361	1639.8577	1639.8570	0.44	0	53	8.5e-05	1	U	K.GFQQISFVNSIATTK.G
6669	328 – 342	820.9362	1639.8578	1639.8570	0.53	0	79	1e-06	1	U	K.GFQQISFVNSIATTK.G
6670	328 – 342	547.6266	1639.8579	1639.8570	0.55	0	63	5.7e-06	1	U	K.GFQQISFVNSIATTK.G
6671	328 – 342	820.9363	1639.8580	1639.8570	0.63	0	64	2.9e-05	1	U	K.GFQQISFVNSIATTK.G
6672	328 – 342	547.6266	1639.8581	1639.8570	0.68	0	44	0.00012	1	U	K.GFQQISFVNSIATTK.G
6673	328 – 342	820.9363	1639.8581	1639.8570	0.70	0	36	0.003	1	U	K.GFQQISFVNSIATTK.G
6674	328 – 342	820.9364	1639.8582	1639.8570	0.73	0	58	2e-05	1	U	K.GFQQISFVNSIATTK.G
6675	328 – 342	820.9364	1639.8583	1639.8570	0.79	0	75	2.7e-06	1	U	K.GFQQISFVNSIATTK.G
6676	328 – 342	820.9367	1639.8588	1639.8570	1.10	0	42	0.00089	1	U	K.GFQQISFVNSIATTK.G
6677	328 – 342	547.6269	1639.8589	1639.8570	1.17	0	25	0.0045	1	U	K.GFQQISFVNSIATTK.G
6678	328 – 342	820.9369	1639.8592	1639.8570	1.33	0	46	0.00034	1	U	K.GFQQISFVNSIATTK.G
6680	328 – 342	820.9376	1639.8607	1639.8570	2.25	0	52	7.6e-05	1	U	K.GFQQISFVNSIATTK.G
4300	346 – 357	679.3586	1356.7027	1356.7038	-0.79	0	81	1.3e-07	1	U	R.HVDYVVDQVVGK.L
4302	346 – 357	679.3588	1356.7031	1356.7038	-0.48	0	59	5.4e-06	1	U	R.HVDYVVDQVVGK.L
4303	346 – 357	679.3589	1356.7033	1356.7038	-0.34	0	83	4.3e-08	1	U	R.HVDYVVDQVVGK.L
4304	346 – 357	679.3591	1356.7036	1356.7038	-0.15	0	77	1.2e-07	1	U	R.HVDYVVDQVVGK.L

Query	Start - End	Observed	Mr(expt)	Mr(calc)	ppm	M	Score	Expect	Rank	U	Peptide
4305	346 - 357	679.3591	1356.7036	1356.7038	-0.12	0	50	4.3e-05	1	U	R.HVDYVVDQVVGK.L
4306	346 - 357	453.2418	1356.7037	1356.7038	-0.055	0	40	0.007	1	U	R.HVDYVVDQVVGK.L
4307	346 - 357	679.3592	1356.7038	1356.7038	-0.021	0	35	0.0011	1	U	R.HVDYVVDQVVGK.L
4308	346 - 357	679.3592	1356.7038	1356.7038	0.038	0	56	2.6e-05	1	U	R.HVDYVVDQVVGK.L
4309	346 - 357	679.3593	1356.7041	1356.7038	0.24	0	79	8.8e-08	1	U	R.HVDYVVDQVVGK.L
4310	346 - 357	679.3594	1356.7042	1356.7038	0.27	0	70	3.7e-07	1	U	R.HVDYVVDQVVGK.L
4312	346 - 357	679.3594	1356.7042	1356.7038	0.33	0	48	3.5e-05	1	U	R.HVDYVVDQVVGK.L
4313	346 - 357	679.3595	1356.7044	1356.7038	0.48	0	36	0.0018	1	U	R.HVDYVVDQVVGK.L
4314	346 - 357	679.3596	1356.7045	1356.7038	0.55	0	36	0.0016	1	U	R.HVDYVVDQVVGK.L
4316	346 - 357	679.3596	1356.7047	1356.7038	0.67	0	50	6.4e-05	1	U	R.HVDYVVDQVVGK.L
4317	346 - 357	679.3596	1356.7047	1356.7038	0.70	0	42	0.00031	1	U	R.HVDYVVDQVVGK.L
4319	346 - 357	679.3599	1356.7052	1356.7038	1.01	0	60	4e-06	1	U	R.HVDYVVDQVVGK.L
483	358 - 364	414.7814	827.5482	827.5480	0.23	1	28	0.011	1	U	K.LIEVVKK.K
2834	368 - 378	387.2324	1158.6754	1158.6761	-0.63	0	27	0.046	1	U	K.AGVSVKPFQVK.N
2835	368 - 378	387.2326	1158.6759	1158.6761	-0.19	0	48	0.00043	1	U	K.AGVSVKPFQVK.N
2836	368 - 378	580.3453	1158.6760	1158.6761	-0.064	0	44	0.00093	1	U	K.AGVSVKPFQVK.N
2838	368 - 378	387.2326	1158.6761	1158.6761	0.017	0	47	0.00055	1	U	K.AGVSVKPFQVK.N
2839	368 - 378	580.3454	1158.6762	1158.6761	0.13	0	33	0.0047	1	U	K.AGVSVKPFQVK.N
2845	368 - 378	580.3457	1158.6769	1158.6761	0.68	0	61	1.8e-05	1	U	K.AGVSVKPFQVK.N
2846	368 - 378	580.3457	1158.6769	1158.6761	0.71	0	61	2e-05	1	U	K.AGVSVKPFQVK.N
10766	379 - 399	859.4257	2575.2554	2575.2529	0.95	0	51	0.0012	1	U	K.NHIWVFINCLIENPTFDSQTK.E
384	440 - 446	401.7244	801.4342	801.4344	-0.32	0	42	0.0027	1	U	K.AQTQLNK.K
1025	440 - 447	465.7720	929.5294	929.5294	-0.017	1	43	0.0014	1	U	K.AQTQLNKK.C
1308	462 - 471	488.2217	974.4289	974.4305	-1.63	0	19	0.023	1	U	K.LDDANDAGGK.H
1310	462 - 471	488.2224	974.4303	974.4305	-0.23	0	56	0.00011	1	U	K.LDDANDAGGK.H
1311	462 - 471	488.2224	974.4303	974.4305	-0.21	0	28	0.0066	1	U	K.LDDANDAGGK.H
1312	462 - 471	488.2225	974.4304	974.4305	-0.090	0	55	0.00011	1	U	K.LDDANDAGGK.H
1313	462 - 471	488.2227	974.4308	974.4305	0.28	0	65	1.7e-05	1	U	K.LDDANDAGGK.H
7731	472 - 487	591.9611	1772.8614	1772.8614	-0.038	0	34	0.0041	1	U	K.HSLECTLILTEGDSAK.S
7732	472 - 487	887.4381	1772.8616	1772.8614	0.078	0	93	6.3e-09	1	U	K.HSLECTLILTEGDSAK.S
2524	488 - 499	564.8394	1127.6642	1127.6663	-1.86	0	70	1.3e-06	1	U	K.SLAVSGLGVIGR.D
2525	488 - 499	376.8956	1127.6649	1127.6663	-1.20	0	26	0.026	1	U	K.SLAVSGLGVIGR.D
2527	488 - 499	564.8400	1127.6654	1127.6663	-0.74	0	43	9.7e-05	1	U	K.SLAVSGLGVIGR.D
2528	488 - 499	564.8401	1127.6656	1127.6663	-0.58	0	60	1.1e-05	1	U	K.SLAVSGLGVIGR.D
2529	488 - 499	564.8401	1127.6657	1127.6663	-0.47	0	63	8.2e-06	1	U	K.SLAVSGLGVIGR.D
2530	488 - 499	564.8402	1127.6657	1127.6663	-0.45	0	68	5.6e-06	1	U	K.SLAVSGLGVIGR.D
2531	488 - 499	564.8402	1127.6658	1127.6663	-0.44	0	47	0.00022	1	U	K.SLAVSGLGVIGR.D
2532	488 - 499	564.8402	1127.6659	1127.6663	-0.33	0	40	0.00075	1	U	K.SLAVSGLGVIGR.D
2533	488 - 499	564.8402	1127.6659	1127.6663	-0.31	0	50	0.00016	1	U	K.SLAVSGLGVIGR.D
2534	488 - 499	564.8403	1127.6660	1127.6663	-0.26	0	52	2.6e-05	1	U	K.SLAVSGLGVIGR.D
2535	488 - 499	564.8403	1127.6660	1127.6663	-0.21	0	61	2.2e-06	1	U	K.SLAVSGLGVIGR.D
2537	488 - 499	564.8403	1127.6661	1127.6663	-0.13	0	69	1.9e-06	1	U	K.SLAVSGLGVIGR.D
2538	488 - 499	564.8404	1127.6662	1127.6663	-0.082	0	79	2e-07	1	U	K.SLAVSGLGVIGR.D
2539	488 - 499	564.8404	1127.6662	1127.6663	-0.046	0	63	4.8e-06	1	U	K.SLAVSGLGVIGR.D
2540	488 - 499	564.8404	1127.6662	1127.6663	-0.028	0	43	0.00045	1	U	K.SLAVSGLGVIGR.D
2541	488 - 499	564.8405	1127.6664	1127.6663	0.096	0	39	0.0015	1	U	K.SLAVSGLGVIGR.D
2542	488 - 499	564.8405	1127.6664	1127.6663	0.11	0	60	8.5e-06	1	U	K.SLAVSGLGVIGR.D
2543	488 - 499	564.8405	1127.6664	1127.6663	0.13	0	39	0.0015	1	U	K.SLAVSGLGVIGR.D
2544	488 - 499	564.8406	1127.6666	1127.6663	0.31	0	34	0.0061	1	U	K.SLAVSGLGVIGR.D
2545	488 - 499	564.8406	1127.6666	1127.6663	0.34	0	37	0.00058	1	U	K.SLAVSGLGVIGR.D
2547	488 - 499	564.8408	1127.6671	1127.6663	0.72	0	40	0.00055	1	U	K.SLAVSGLGVIGR.D
2548	488 - 499	564.8416	1127.6686	1127.6663	2.08	0	28	0.0025	1	U	K.SLAVSGLGVIGR.D
2472	500 - 508	561.8064	1121.5981	1121.5982	-0.024	1	28	0.048	1	U	R.DRYGVFPLR.G
603	502 - 508	426.2422	850.4698	850.4701	-0.37	0	24	0.026	1		R.YGVFPLR.G
606	502 - 508	426.2424	850.4703	850.4701	0.24	0	29	0.015	1		R.YGVFPLR.G
607	502 - 508	426.2425	850.4704	850.4701	0.29	0	25	0.016	1		R.YGVFPLR.G
5768	521 - 533	765.4031	1528.7916	1528.7919	-0.16	0	84	3e-07	1		K.QIMENAEINNIIK.I
5769	521 - 533	510.6048	1528.7925	1528.7919	0.44	0	42	0.0052	1		K.QIMENAEINNIIK.I
5940	521 - 533	773.3997	1544.7848	1544.7868	-1.28	0	52	6.8e-05	1		K.QIMENAEINNIIK.I + Oxidation (M)
5941	521 - 533	773.4005	1544.7865	1544.7868	-0.18	0	63	1.4e-05	1		K.QIMENAEINNIIK.I + Oxidation (M)
5942	521 - 533	773.4008	1544.7871	1544.7868	0.23	0	75	2.8e-06	1		K.QIMENAEINNIIK.I + Oxidation (M)
5943	521 - 533	773.4011	1544.7876	1544.7868	0.52	0	66	6e-06	1		K.QIMENAEINNIIK.I + Oxidation (M)
5944	521 - 533	773.4017	1544.7888	1544.7868	1.29	0	50	0.00012	1		K.QIMENAEINNIIK.I + Oxidation (M)
452	534 - 540	410.7505	819.4864	819.4854	1.15	0	39	0.0018	1		K.IVGLQYK.K
2804	541 - 550	578.2796	1154.5447	1154.5455	-0.67	1	30	0.021	1	U	K.KSYDDAESLK.T
2805	541 - 550	578.2797	1154.5448	1154.5455	-0.60	1	24	0.019	1	U	K.KSYDDAESLK.T
2807	541 - 550	578.2802	1154.5457	1154.5455	0.21	1	35	0.001	1	U	K.KSYDDAESLK.T
1689	542 - 550	514.2324	1026.4502	1026.4505	-0.33	0	19	0.022	1	U	K.SYDDAESLK.T
1690	542 - 550	514.2326	1026.4506	1026.4505	0.041	0	31	0.003	1	U	K.SYDDAESLK.T
1691	542 - 550	514.2327	1026.4508	1026.4505	0.29	0	26	0.0094	1	U	K.SYDDAESLK.T
1692	542 - 550	514.2328	1026.4510	1026.4505	0.47	0	55	0.00019	1	U	K.SYDDAESLK.T
7412	557 - 571	866.4053	1730.7960	1730.7968	-0.44	0	67	3.4e-06	1		K.IMIMTDQDQDGSHIK.G
7414	557 - 571	577.9394	1730.7963	1730.7968	-0.25	0	26	0.025	1		K.IMIMTDQDQDGSHIK.G
7417	557 - 571	577.9396	1730.7971	1730.7968	0.20	0	23	0.038	1		K.IMIMTDQDQDGSHIK.G
7420	557 - 571	577.9400	1730.7980	1730.7968	0.73	0	33	0.03	1		K.IMIMTDQDQDGSHIK.G
7421	557 - 571	866.4065	1730.7984	1730.7968	0.94	0	86	1.8e-07	1		K.IMIMTDQDQDGSHIK.G
7529	557 - 571	874.4023	1746.7900	1746.7917	-0.96	0	76	5.2e-07	1		K.IMIMTDQDQDGSHIK.G + Oxidation (M)
7530	557 - 571	583.2708	1746.7906	1746.7917	-0.60	0	46	0.002	1		K.IMIMTDQDQDGSHIK.G + Oxidation (M)
7531	557 - 571	583.2709	1746.7909	1746.7917	-0.44	0	47	0.00066	1		K.IMIMTDQDQDGSHIK.G + Oxidation (M)
7532	557 - 571	874.4028	1746.7910	1746.7917	-0.38	0	53	0.0005	1		K.IMIMTDQDQDGSHIK.G + Oxidation (M)
7533	557 - 571	583.2710	1746.7911	1746.7917	-0.30	0	25	0.05	1		K.IMIMTDQDQDGSHIK.G

Query	Start – End	Observed	Mr(expt)	Mr(calc)	ppm	M	Score	Expect	Rank	U	Peptide
											+ Oxidation (M)
7535	557 – 571	874.4029	1746.7913	1746.7917	-0.23	0	76	4.5e-07	1		K.IMIMTDQDQDGSHIK.G + Oxidation (M)
7537	557 – 571	583.2712	1746.7918	1746.7917	0.074	0	51	0.00085	1		K.IMIMTDQDQDGSHIK.G + Oxidation (M)
7538	557 – 571	583.2713	1746.7921	1746.7917	0.25	0	47	0.002	1		K.IMIMTDQDQDGSHIK.G + Oxidation (M)
7539	557 – 571	874.4034	1746.7922	1746.7917	0.31	0	69	1.4e-05	1		K.IMIMTDQDQDGSHIK.G + Oxidation (M)
7540	557 – 571	583.2717	1746.7932	1746.7917	0.86	0	52	0.00062	1		K.IMIMTDQDQDGSHIK.G + Oxidation (M)
7541	557 – 571	583.2721	1746.7944	1746.7917	1.59	0	37	0.0034	1		K.IMIMTDQDQDGSHIK.G + Oxidation (M)
7650	557 – 571	588.6021	1762.7846	1762.7866	-1.13	0	42	0.0024	1		K.IMIMTDQDQDGSHIK.G + 2 Oxidation (M)
7651	557 – 571	882.3996	1762.7847	1762.7866	-1.08	0	65	1.6e-05	1		K.IMIMTDQDQDGSHIK.G + 2 Oxidation (M)
7654	557 – 571	588.6025	1762.7856	1762.7866	-0.56	0	52	0.0003	1		K.IMIMTDQDQDGSHIK.G + 2 Oxidation (M)
7655	557 – 571	882.4001	1762.7856	1762.7866	-0.55	0	52	0.00058	1		K.IMIMTDQDQDGSHIK.G + 2 Oxidation (M)
7656	557 – 571	588.6025	1762.7857	1762.7866	-0.50	0	35	0.0047	1		K.IMIMTDQDQDGSHIK.G + 2 Oxidation (M)
7658	557 – 571	588.6026	1762.7859	1762.7866	-0.42	0	30	0.016	1		K.IMIMTDQDQDGSHIK.G + 2 Oxidation (M)
7659	557 – 571	588.6026	1762.7859	1762.7866	-0.40	0	55	0.00011	1		K.IMIMTDQDQDGSHIK.G + 2 Oxidation (M)
7661	557 – 571	588.6027	1762.7862	1762.7866	-0.25	0	36	0.0011	1		K.IMIMTDQDQDGSHIK.G + 2 Oxidation (M)
7662	557 – 571	588.6027	1762.7862	1762.7866	-0.23	0	41	0.0018	1		K.IMIMTDQDQDGSHIK.G + 2 Oxidation (M)
7663	557 – 571	882.4004	1762.7863	1762.7866	-0.18	0	62	6.2e-05	1		K.IMIMTDQDQDGSHIK.G + 2 Oxidation (M)
7665	557 – 571	882.4005	1762.7865	1762.7866	-0.036	0	55	0.00034	1		K.IMIMTDQDQDGSHIK.G + 2 Oxidation (M)
7666	557 – 571	882.4006	1762.7866	1762.7866	-0.0017	0	67	1.2e-05	1		K.IMIMTDQDQDGSHIK.G + 2 Oxidation (M)
7667	557 – 571	882.4006	1762.7866	1762.7866	0.021	0	54	4e-05	1		K.IMIMTDQDQDGSHIK.G + 2 Oxidation (M)
7668	557 – 571	882.4006	1762.7867	1762.7866	0.055	0	64	1.2e-05	1		K.IMIMTDQDQDGSHIK.G + 2 Oxidation (M)
7669	557 – 571	882.4006	1762.7867	1762.7866	0.078	0	66	3.3e-05	1		K.IMIMTDQDQDGSHIK.G + 2 Oxidation (M)
7670	557 – 571	588.6029	1762.7870	1762.7866	0.23	0	58	7.3e-05	1		K.IMIMTDQDQDGSHIK.G + 2 Oxidation (M)
7673	557 – 571	882.4011	1762.7876	1762.7866	0.55	0	44	0.00094	1		K.IMIMTDQDQDGSHIK.G + 2 Oxidation (M)
7674	557 – 571	588.6032	1762.7878	1762.7866	0.67	0	31	0.0061	1		K.IMIMTDQDQDGSHIK.G + 2 Oxidation (M)
8622	572 – 587	951.5398	1901.0650	1901.0676	-1.32	0	36	0.00038	1	U	K.GLLINFIHHNWPSLLK.H
8628	572 – 587	951.5410	1901.0675	1901.0676	-0.033	0	53	3.4e-05	1	U	K.GLLINFIHHNWPSLLK.H
8630	572 – 587	951.5412	1901.0678	1901.0676	0.16	0	54	0.00013	1	U	K.GLLINFIHHNWPSLLK.H
8631	572 – 587	634.6966	1901.0681	1901.0676	0.28	0	21	0.016	1	U	K.GLLINFIHHNWPSLLK.H
8633	572 – 587	634.6967	1901.0683	1901.0676	0.41	0	30	0.0055	1	U	K.GLLINFIHHNWPSLLK.H
5770	588 – 600	765.4204	1528.8262	1528.8290	-1.83	0	73	5.2e-07	1	U	K.HGFLEEFITPIVK.A
5771	588 – 600	510.6162	1528.8267	1528.8290	-1.47	0	25	0.018	1	U	K.HGFLEEFITPIVK.A
5772	588 – 600	765.4206	1528.8267	1528.8290	-1.46	0	86	1.6e-08	1	U	K.HGFLEEFITPIVK.A
5773	588 – 600	510.6163	1528.8271	1528.8290	-1.24	0	44	0.0021	1	U	K.HGFLEEFITPIVK.A
5774	588 – 600	765.4214	1528.8282	1528.8290	-0.52	0	54	8.5e-06	1	U	K.HGFLEEFITPIVK.A
5775	588 – 600	510.6167	1528.8282	1528.8290	-0.47	0	21	0.031	1	U	K.HGFLEEFITPIVK.A
5776	588 – 600	510.6167	1528.8283	1528.8290	-0.43	0	22	0.019	1	U	K.HGFLEEFITPIVK.A
5777	588 – 600	765.4214	1528.8283	1528.8290	-0.42	0	73	3.8e-07	1	U	K.HGFLEEFITPIVK.A
5779	588 – 600	765.4215	1528.8284	1528.8290	-0.36	0	62	3.6e-06	1	U	K.HGFLEEFITPIVK.A
5780	588 – 600	765.4215	1528.8284	1528.8290	-0.35	0	53	1.9e-05	1	U	K.HGFLEEFITPIVK.A
5781	588 – 600	510.6168	1528.8285	1528.8290	-0.32	0	39	0.0017	1	U	K.HGFLEEFITPIVK.A
5782	588 – 600	510.6168	1528.8285	1528.8290	-0.28	0	30	0.048	1	U	K.HGFLEEFITPIVK.A
5783	588 – 600	510.6168	1528.8285	1528.8290	-0.28	0	37	0.0071	1	U	K.HGFLEEFITPIVK.A
5784	588 – 600	510.6168	1528.8286	1528.8290	-0.26	0	49	0.00075	1	U	K.HGFLEEFITPIVK.A
5785	588 – 600	765.4216	1528.8286	1528.8290	-0.23	0	72	3e-07	1	U	K.HGFLEEFITPIVK.A
5786	588 – 600	510.6169	1528.8288	1528.8290	-0.14	0	47	0.00015	1	U	K.HGFLEEFITPIVK.A
5787	588 – 600	510.6169	1528.8288	1528.8290	-0.099	0	35	0.018	1	U	K.HGFLEEFITPIVK.A
5788	588 – 600	765.4217	1528.8288	1528.8290	-0.089	0	65	7.1e-06	1	U	K.HGFLEEFITPIVK.A
5789	588 – 600	765.4217	1528.8289	1528.8290	-0.024	0	71	2.4e-07	1	U	K.HGFLEEFITPIVK.A
5790	588 – 600	510.6169	1528.8289	1528.8290	-0.021	0	25	0.014	1	U	K.HGFLEEFITPIVK.A
5791	588 – 600	765.4218	1528.8289	1528.8290	-0.010	0	68	1.6e-06	1	U	K.HGFLEEFITPIVK.A
5792	588 – 600	765.4218	1528.8290	1528.8290	0.0026	0	84	1.9e-08	1	U	K.HGFLEEFITPIVK.A
5793	588 – 600	510.6169	1528.8290	1528.8290	0.018	0	27	0.027	1	U	K.HGFLEEFITPIVK.A
5795	588 – 600	510.6170	1528.8292	1528.8290	0.14	0	44	0.002	1	U	K.HGFLEEFITPIVK.A
5796	588 – 600	765.4220	1528.8295	1528.8290	0.34	0	87	1e-08	1	U	K.HGFLEEFITPIVK.A
5797	588 – 600	765.4220	1528.8295	1528.8290	0.36	0	63	3.2e-06	1	U	K.HGFLEEFITPIVK.A
5798	588 – 600	510.6171	1528.8296	1528.8290	0.39	0	26	0.04	1	U	K.HGFLEEFITPIVK.A
5799	588 – 600	765.4221	1528.8296	1528.8290	0.43	0	72	9.1e-07	1	U	K.HGFLEEFITPIVK.A
5800	588 – 600	510.6172	1528.8298	1528.8290	0.53	0	32	0.015	1	U	K.HGFLEEFITPIVK.A
5801	588 – 600	765.4222	1528.8298	1528.8290	0.58	0	66	7.9e-07	1	U	K.HGFLEEFITPIVK.A
9640	604 – 620	1080.5174	2159.0202	2159.0211	-0.43	1	100	1.2e-08	1	U	K.NKQELSFYSIPEFDEWK.K
9642	604 – 620	720.6813	2159.0220	2159.0211	0.43	1	35	0.004	1	U	K.NKQELSFYSIPEFDEWK.K
10064	604 – 621	1144.5645	2287.1145	2287.1161	-0.69	2	67	1.1e-06	1	U	K.NKQELSFYSIPEFDEWKK.H
10065	604 – 621	572.7859	2287.1146	2287.1161	-0.65	2	26	0.024	1	U	K.NKQELSFYSIPEFDEWKK.H
10067	604 – 621	763.3794	2287.1163	2287.1161	0.11	2	45	0.00036	1	U	K.NKQELSFYSIPEFDEWKK.H
8741	606 – 620	959.4487	1916.8828	1916.8832	-0.21	0	50	7.3e-05	1	U	K.QELSFYSIPEFDEWK.K
9269	606 – 621	1023.4965	2044.9784	2044.9782	0.12	1	38	0.002	1	U	K.QELSFYSIPEFDEWKK.H

Query	Start - End	Observed	Mr(expt)	Mr(calc)	ppm	M	Score	Expect	Rank	U	Peptide
9270	606 - 621	682.6668	2044.9785	2044.9782	0.15	1	29	0.019	1	U	K.QELSFYSIPEFDEWKK.H
1950	647 - 654	530.7238	1059.4329	1059.4331	-0.16	0	22	0.04	1	U	K.EYFADMER.H
1951	647 - 654	530.7240	1059.4335	1059.4331	0.33	0	30	0.0068	1	U	K.EYFADMER.H
1952	647 - 654	530.7243	1059.4340	1059.4331	0.87	0	20	0.036	1	U	K.EYFADMER.H
2089	647 - 654	538.7210	1075.4274	1075.4280	-0.61	0	33	0.0091	1	U	K.EYFADMER.H + Oxidation (M)
2090	647 - 654	538.7211	1075.4276	1075.4280	-0.38	0	22	0.036	1	U	K.EYFADMER.H + Oxidation (M)
2091	647 - 654	538.7213	1075.4281	1075.4280	0.063	0	26	0.027	1	U	K.EYFADMER.H + Oxidation (M)
2092	647 - 654	538.7214	1075.4282	1075.4280	0.19	0	31	0.0067	1	U	K.EYFADMER.H + Oxidation (M)
6872	661 - 676	834.9087	1667.8028	1667.8042	-0.84	0	30	0.003	1	U	R.YAGPEDDAAITLAFSK.K
6873	661 - 676	834.9088	1667.8031	1667.8042	-0.69	0	68	5.5e-07	1	U	R.YAGPEDDAAITLAFSK.K
6874	661 - 676	834.9089	1667.8032	1667.8042	-0.63	0	68	5.3e-07	1	U	R.YAGPEDDAAITLAFSK.K
6875	661 - 676	834.9089	1667.8032	1667.8042	-0.60	0	65	3.2e-06	1	U	R.YAGPEDDAAITLAFSK.K
6876	661 - 676	834.9090	1667.8034	1667.8042	-0.49	0	38	0.00027	1	U	R.YAGPEDDAAITLAFSK.K
6877	661 - 676	834.9090	1667.8034	1667.8042	-0.48	0	76	7.5e-08	1	U	R.YAGPEDDAAITLAFSK.K
6878	661 - 676	834.9091	1667.8036	1667.8042	-0.41	0	55	2.5e-05	1	U	R.YAGPEDDAAITLAFSK.K
6879	661 - 676	834.9091	1667.8036	1667.8042	-0.37	0	75	4.1e-07	1	U	R.YAGPEDDAAITLAFSK.K
6880	661 - 676	834.9091	1667.8036	1667.8042	-0.37	0	29	0.004	1	U	R.YAGPEDDAAITLAFSK.K
6881	661 - 676	834.9092	1667.8038	1667.8042	-0.24	0	75	2.8e-07	1	U	R.YAGPEDDAAITLAFSK.K
6882	661 - 676	834.9092	1667.8039	1667.8042	-0.23	0	81	8.7e-08	1	U	R.YAGPEDDAAITLAFSK.K
6883	661 - 676	834.9093	1667.8040	1667.8042	-0.13	0	41	0.00072	1	U	R.YAGPEDDAAITLAFSK.K
6884	661 - 676	834.9094	1667.8043	1667.8042	0.062	0	63	1.1e-06	1	U	R.YAGPEDDAAITLAFSK.K
6885	661 - 676	834.9095	1667.8044	1667.8042	0.074	0	77	7e-08	1	U	R.YAGPEDDAAITLAFSK.K
6886	661 - 676	834.9095	1667.8045	1667.8042	0.16	0	79	4.7e-08	1	U	R.YAGPEDDAAITLAFSK.K
6887	661 - 676	834.9096	1667.8047	1667.8042	0.28	0	28	0.0031	1	U	R.YAGPEDDAAITLAFSK.K
6888	661 - 676	834.9097	1667.8048	1667.8042	0.35	0	75	3.1e-07	1	U	R.YAGPEDDAAITLAFSK.K
6889	661 - 676	834.9097	1667.8048	1667.8042	0.36	0	72	2.8e-07	1	U	R.YAGPEDDAAITLAFSK.K
6890	661 - 676	834.9097	1667.8049	1667.8042	0.37	0	62	5.4e-06	1	U	R.YAGPEDDAAITLAFSK.K
6891	661 - 676	834.9098	1667.8049	1667.8042	0.42	0	61	4.7e-06	1	U	R.YAGPEDDAAITLAFSK.K
6892	661 - 676	834.9098	1667.8050	1667.8042	0.43	0	69	1.7e-06	1	U	R.YAGPEDDAAITLAFSK.K
6893	661 - 676	834.9098	1667.8050	1667.8042	0.46	0	76	1.5e-07	1	U	R.YAGPEDDAAITLAFSK.K
6894	661 - 676	556.9423	1667.8051	1667.8042	0.51	0	51	0.00015	1	U	R.YAGPEDDAAITLAFSK.K
6895	661 - 676	834.9101	1667.8057	1667.8042	0.87	0	61	4.3e-06	1	U	R.YAGPEDDAAITLAFSK.K
6896	661 - 676	834.9101	1667.8057	1667.8042	0.88	0	56	8.2e-06	1	U	R.YAGPEDDAAITLAFSK.K
6898	661 - 676	834.9102	1667.8058	1667.8042	0.94	0	38	0.00029	1	U	R.YAGPEDDAAITLAFSK.K
6899	661 - 676	834.9103	1667.8060	1667.8042	1.06	0	29	0.0024	1	U	R.YAGPEDDAAITLAFSK.K
6900	661 - 676	834.9103	1667.8061	1667.8042	1.13	0	67	5.8e-07	1	U	R.YAGPEDDAAITLAFSK.K
6901	661 - 676	834.9103	1667.8061	1667.8042	1.13	0	48	0.00011	1	U	R.YAGPEDDAAITLAFSK.K
6902	661 - 676	834.9105	1667.8064	1667.8042	1.32	0	74	3.9e-07	1	U	R.YAGPEDDAAITLAFSK.K
6903	661 - 676	834.9106	1667.8067	1667.8042	1.46	0	89	6.7e-09	1	U	R.YAGPEDDAAITLAFSK.K
6904	661 - 676	834.9118	1667.8091	1667.8042	2.93	0	83	1.7e-08	1	U	R.YAGPEDDAAITLAFSK.K
5161	683 - 693	490.2345	1467.6817	1467.6816	0.073	1	32	0.012	1		R.KEWLTNFMEDR.R
5279	683 - 693	742.8454	1483.6762	1483.6765	-0.21	1	38	0.0015	1		R.KEWLTNFMEDR.R + Oxidation (M)
5280	683 - 693	495.5661	1483.6764	1483.6765	-0.11	1	31	0.0068	1		R.KEWLTNFMEDR.R + Oxidation (M)
5281	683 - 693	495.5661	1483.6765	1483.6765	-0.0081	1	25	0.011	1		R.KEWLTNFMEDR.R + Oxidation (M)
5282	683 - 693	742.8456	1483.6767	1483.6765	0.11	1	26	0.0091	1		R.KEWLTNFMEDR.R + Oxidation (M)
5283	683 - 693	495.5662	1483.6767	1483.6765	0.11	1	45	0.00023	1		R.KEWLTNFMEDR.R + Oxidation (M)
5284	683 - 693	742.8456	1483.6767	1483.6765	0.12	1	56	1.9e-05	1		R.KEWLTNFMEDR.R + Oxidation (M)
5285	683 - 693	742.8457	1483.6768	1483.6765	0.15	1	45	0.0002	1		R.KEWLTNFMEDR.R + Oxidation (M)
5286	683 - 693	495.5662	1483.6768	1483.6765	0.17	1	28	0.0078	1		R.KEWLTNFMEDR.R + Oxidation (M)
5287	683 - 693	742.8458	1483.6771	1483.6765	0.41	1	26	0.0088	1		R.KEWLTNFMEDR.R + Oxidation (M)
6556	683 - 694	542.2685	1623.7836	1623.7827	0.51	2	29	0.025	1		R.KEWLTNFMEDRR.Q
4210	684 - 693	670.8007	1339.5869	1339.5867	0.19	0	28	0.0024	1		K.EWLTNFMEDR.R
4211	684 - 693	670.8010	1339.5874	1339.5867	0.57	0	65	7.4e-06	1		K.EWLTNFMEDR.R
4292	684 - 693	678.7979	1355.5812	1355.5816	-0.27	0	23	0.02	1		K.EWLTNFMEDR.R + Oxidation (M)
4293	684 - 693	678.7980	1355.5814	1355.5816	-0.13	0	28	0.0094	1		K.EWLTNFMEDR.R + Oxidation (M)
4295	684 - 693	678.7983	1355.5820	1355.5816	0.31	0	26	0.0092	1		K.EWLTNFMEDR.R + Oxidation (M)
8191	697 - 712	610.9992	1829.9758	1829.9788	-1.64	1	37	0.0015	1	U	R.RLHGLPEQFLYGTATK.H
8192	697 - 712	610.9992	1829.9759	1829.9788	-1.59	1	47	0.00022	1	U	R.RLHGLPEQFLYGTATK.H
8193	697 - 712	610.9993	1829.9762	1829.9788	-1.44	1	44	0.00046	1	U	R.RLHGLPEQFLYGTATK.H
8194	697 - 712	610.9994	1829.9762	1829.9788	-1.41	1	37	0.003	1	U	R.RLHGLPEQFLYGTATK.H
8195	697 - 712	915.9960	1829.9774	1829.9788	-0.79	1	76	1.7e-07	1	U	R.RLHGLPEQFLYGTATK.H
8196	697 - 712	610.9997	1829.9774	1829.9788	-0.77	1	28	0.0066	1	U	R.RLHGLPEQFLYGTATK.H
8197	697 - 712	610.9998	1829.9775	1829.9788	-0.69	1	67	8.8e-06	1	U	R.RLHGLPEQFLYGTATK.H
8198	697 - 712	458.5018	1829.9780	1829.9788	-0.45	1	36	0.0046	1	U	R.RLHGLPEQFLYGTATK.H
8199	697 - 712	611.0001	1829.9785	1829.9788	-0.17	1	51	9.8e-05	1	U	R.RLHGLPEQFLYGTATK.H
8200	697 - 712	611.0001	1829.9786	1829.9788	-0.13	1	17	0.043	1	U	R.RLHGLPEQFLYGTATK.H
8202	697 - 712	611.0002	1829.9789	1829.9788	0.047	1	33	0.0024	1	U	R.RLHGLPEQFLYGTATK.H
8203	697 - 712	611.0003	1829.9791	1829.9788	0.15	1	38	0.00081	1	U	R.RLHGLPEQFLYGTATK.H
8205	697 - 712	611.0006	1829.9800	1829.9788	0.67	1	20	0.041	1	U	R.RLHGLPEQFLYGTATK.H
8206	697 - 712	611.0007	1829.9804	1829.9788	0.85	1	32	0.0086	1	U	R.RLHGLPEQFLYGTATK.H
6940	698 - 712	558.9653	1673.8741	1673.8777	-2.15	0	21	0.016	1	U	R.LHGLPEQFLYGTATK.H
6941	698 - 712	837.9444	1673.8742	1673.8777	-2.06	0	20	0.019	1	U	R.LHGLPEQFLYGTATK.H
6942	698 - 712	837.9445	1673.8744	1673.8777	-1.96	0	28	0.0026	1	U	R.LHGLPEQFLYGTATK.H
6943	698 - 712	837.9449	1673.8752	1673.8777	-1.51	0	31	0.0011	1	U	R.LHGLPEQFLYGTATK.H

Query	Start - End	Observed	Mr(expt)	Mr(calc)	ppm	M	Score	Expect	Rank	U	Peptide
6945	698 - 712	837.9454	1673.8761	1673.8777	-0.93	0	22	0.0093	1	U	R.LHGLPEQFLYGTATK.H
6947	698 - 712	837.9457	1673.8769	1673.8777	-0.49	0	32	0.001	1	U	R.LHGLPEQFLYGTATK.H
6948	698 - 712	837.9457	1673.8769	1673.8777	-0.49	0	28	0.0023	1	U	R.LHGLPEQFLYGTATK.H
6949	698 - 712	837.9458	1673.8770	1673.8777	-0.44	0	21	0.012	1	U	R.LHGLPEQFLYGTATK.H
6950	698 - 712	837.9458	1673.8771	1673.8777	-0.35	0	26	0.0035	1	U	R.LHGLPEQFLYGTATK.H
6951	698 - 712	558.9663	1673.8772	1673.8777	-0.32	0	36	0.013	1	U	R.LHGLPEQFLYGTATK.H
6953	698 - 712	837.9459	1673.8772	1673.8777	-0.29	0	36	0.00041	1	U	R.LHGLPEQFLYGTATK.H
6954	698 - 712	558.9664	1673.8773	1673.8777	-0.27	0	26	0.033	1	U	R.LHGLPEQFLYGTATK.H
6955	698 - 712	558.9664	1673.8773	1673.8777	-0.23	0	41	0.0011	1	U	R.LHGLPEQFLYGTATK.H
6956	698 - 712	837.9459	1673.8773	1673.8777	-0.22	0	31	0.0038	1	U	R.LHGLPEQFLYGTATK.H
6957	698 - 712	837.9460	1673.8773	1673.8777	-0.21	0	27	0.0034	1	U	R.LHGLPEQFLYGTATK.H
6958	698 - 712	558.9664	1673.8774	1673.8777	-0.20	0	32	0.0084	1	U	R.LHGLPEQFLYGTATK.H
6959	698 - 712	837.9460	1673.8774	1673.8777	-0.15	0	49	3e-05	1	U	R.LHGLPEQFLYGTATK.H
6960	698 - 712	558.9664	1673.8775	1673.8777	-0.12	0	29	0.0062	1	U	R.LHGLPEQFLYGTATK.H
6962	698 - 712	558.9665	1673.8777	1673.8777	0.00060	0	43	0.00027	1	U	R.LHGLPEQFLYGTATK.H
6963	698 - 712	837.9461	1673.8777	1673.8777	0.0042	0	72	2.1e-07	1	U	R.LHGLPEQFLYGTATK.H
6965	698 - 712	558.9665	1673.8778	1673.8777	0.054	0	44	0.00052	1	U	R.LHGLPEQFLYGTATK.H
6966	698 - 712	837.9462	1673.8778	1673.8777	0.076	0	20	0.015	1	U	R.LHGLPEQFLYGTATK.H
6967	698 - 712	558.9666	1673.8779	1673.8777	0.090	0	25	0.036	1	U	R.LHGLPEQFLYGTATK.H
6970	698 - 712	558.9666	1673.8780	1673.8777	0.18	0	35	0.0083	1	U	R.LHGLPEQFLYGTATK.H
6972	698 - 712	837.9463	1673.8780	1673.8777	0.18	0	28	0.0037	1	U	R.LHGLPEQFLYGTATK.H
6973	698 - 712	837.9463	1673.8780	1673.8777	0.20	0	46	0.00036	1	U	R.LHGLPEQFLYGTATK.H
6974	698 - 712	558.9666	1673.8780	1673.8777	0.20	0	31	0.012	1	U	R.LHGLPEQFLYGTATK.H
6975	698 - 712	558.9666	1673.8781	1673.8777	0.22	0	28	0.011	1	U	R.LHGLPEQFLYGTATK.H
6976	698 - 712	558.9666	1673.8781	1673.8777	0.23	0	43	0.0008	1	U	R.LHGLPEQFLYGTATK.H
6977	698 - 712	558.9667	1673.8782	1673.8777	0.29	0	43	0.00041	1	U	R.LHGLPEQFLYGTATK.H
6979	698 - 712	558.9668	1673.8785	1673.8777	0.47	0	50	0.0007	1	U	R.LHGLPEQFLYGTATK.H
6980	698 - 712	558.9668	1673.8787	1673.8777	0.59	0	34	0.034	1	U	R.LHGLPEQFLYGTATK.H
6983	698 - 712	837.9471	1673.8796	1673.8777	1.16	0	70	7.8e-07	1	U	R.LHGLPEQFLYGTATK.H
6984	698 - 712	558.9672	1673.8797	1673.8777	1.22	0	41	0.0017	1	U	R.LHGLPEQFLYGTATK.H
6986	698 - 712	558.9672	1673.8798	1673.8777	1.27	0	42	0.00099	1	U	R.LHGLPEQFLYGTATK.H
3570	713 - 722	632.8182	1263.6217	1263.6248	-2.39	0	26	0.0045	1	U	K.HLTYNDFINK.E
3571	713 - 722	632.8189	1263.6232	1263.6248	-1.21	0	24	0.023	1	U	K.HLTYNDFINK.E
3572	713 - 722	422.2152	1263.6238	1263.6248	-0.77	0	28	0.018	1	U	K.HLTYNDFINK.E
3573	713 - 722	632.8192	1263.6239	1263.6248	-0.71	0	30	0.016	1	U	K.HLTYNDFINK.E
3574	713 - 722	632.8193	1263.6240	1263.6248	-0.62	0	30	0.0078	1	U	K.HLTYNDFINK.E
3575	713 - 722	632.8193	1263.6240	1263.6248	-0.60	0	30	0.013	1	U	K.HLTYNDFINK.E
3576	713 - 722	632.8193	1263.6241	1263.6248	-0.56	0	36	0.00092	1	U	K.HLTYNDFINK.E
3577	713 - 722	632.8193	1263.6241	1263.6248	-0.54	0	33	0.003	1	U	K.HLTYNDFINK.E
3578	713 - 722	632.8193	1263.6241	1263.6248	-0.51	0	33	0.0047	1	U	K.HLTYNDFINK.E
3579	713 - 722	632.8194	1263.6242	1263.6248	-0.41	0	37	0.0075	1	U	K.HLTYNDFINK.E
3580	713 - 722	632.8195	1263.6244	1263.6248	-0.32	0	31	0.0073	1	U	K.HLTYNDFINK.E
3582	713 - 722	422.2155	1263.6247	1263.6248	-0.055	0	26	0.021	1	U	K.HLTYNDFINK.E
3583	713 - 722	632.8196	1263.6247	1263.6248	-0.050	0	27	0.015	1	U	K.HLTYNDFINK.E
3585	713 - 722	632.8197	1263.6249	1263.6248	0.11	0	35	0.0035	1	U	K.HLTYNDFINK.E
3586	713 - 722	632.8198	1263.6249	1263.6248	0.14	0	23	0.0089	1	U	K.HLTYNDFINK.E
3587	713 - 722	632.8198	1263.6250	1263.6248	0.22	0	27	0.011	1	U	K.HLTYNDFINK.E
3588	713 - 722	632.8198	1263.6251	1263.6248	0.25	0	40	0.00059	1	U	K.HLTYNDFINK.E
3589	713 - 722	632.8198	1263.6251	1263.6248	0.30	0	26	0.015	1	U	K.HLTYNDFINK.E
3590	713 - 722	632.8198	1263.6251	1263.6248	0.30	0	31	0.005	1	U	K.HLTYNDFINK.E
3591	713 - 722	632.8198	1263.6251	1263.6248	0.30	0	24	0.014	1	U	K.HLTYNDFINK.E
3592	713 - 722	632.8199	1263.6252	1263.6248	0.33	0	27	0.011	1	U	K.HLTYNDFINK.E
3593	713 - 722	632.8199	1263.6252	1263.6248	0.38	0	19	0.021	1	U	K.HLTYNDFINK.E
3594	713 - 722	632.8200	1263.6255	1263.6248	0.57	0	16	0.034	1	U	K.HLTYNDFINK.E
3595	713 - 722	632.8200	1263.6255	1263.6248	0.61	0	24	0.0059	1	U	K.HLTYNDFINK.E
3596	713 - 722	632.8202	1263.6258	1263.6248	0.82	0	27	0.0055	1	U	K.HLTYNDFINK.E
3597	713 - 722	632.8202	1263.6258	1263.6248	0.84	0	30	0.0014	1	U	K.HLTYNDFINK.E
3598	713 - 722	632.8202	1263.6259	1263.6248	0.93	0	27	0.0043	1	U	K.HLTYNDFINK.E
4842	723 - 734	718.8541	1435.6937	1435.6943	-0.41	0	36	0.0035	1		K.ELILFSNSDNER.S
4844	723 - 734	718.8543	1435.6941	1435.6943	-0.14	0	40	0.0016	1		K.ELILFSNSDNER.S
4845	723 - 734	718.8544	1435.6942	1435.6943	-0.057	0	32	0.0083	1		K.ELILFSNSDNER.S
4846	723 - 734	718.8544	1435.6942	1435.6943	-0.043	0	36	0.0094	1		K.ELILFSNSDNER.S
4847	723 - 734	718.8545	1435.6945	1435.6943	0.12	0	34	0.0072	1		K.ELILFSNSDNER.S
4848	723 - 734	718.8546	1435.6947	1435.6943	0.28	0	35	0.0019	1		K.ELILFSNSDNER.S
4849	723 - 734	718.8546	1435.6947	1435.6943	0.28	0	35	0.026	1		K.ELILFSNSDNER.S
4850	723 - 734	718.8546	1435.6947	1435.6943	0.32	0	30	0.0036	1		K.ELILFSNSDNER.S
4851	723 - 734	718.8547	1435.6948	1435.6943	0.35	0	51	0.0003	1		K.ELILFSNSDNER.S
4852	723 - 734	718.8547	1435.6949	1435.6943	0.42	0	27	0.047	1		K.ELILFSNSDNER.S
4854	723 - 734	718.8548	1435.6949	1435.6943	0.46	0	20	0.041	1		K.ELILFSNSDNER.S
4856	723 - 734	718.8549	1435.6952	1435.6943	0.67	0	51	0.00049	1		K.ELILFSNSDNER.S
4857	723 - 734	718.8550	1435.6954	1435.6943	0.76	0	40	0.003	1		K.ELILFSNSDNER.S
4858	723 - 734	718.8550	1435.6954	1435.6943	0.78	0	31	0.017	1		K.ELILFSNSDNER.S
4860	723 - 734	718.8551	1435.6957	1435.6943	1.00	0	30	0.0086	1		K.ELILFSNSDNER.S
4861	723 - 734	718.8554	1435.6963	1435.6943	1.43	0	28	0.016	1		K.ELILFSNSDNER.S
5425	735 - 748	750.9115	1499.8085	1499.8096	-0.76	0	39	0.0012	1	U	R.SIPSLVDGFKPGQR.K
5426	735 - 748	750.9117	1499.8088	1499.8096	-0.52	0	47	0.00011	1	U	R.SIPSLVDGFKPGQR.K
5434	735 - 748	750.9121	1499.8097	1499.8096	0.025	0	51	3.9e-05	1	U	R.SIPSLVDGFKPGQR.K
5437	735 - 748	750.9122	1499.8099	1499.8096	0.20	0	53	6.8e-05	1	U	R.SIPSLVDGFKPGQR.K
5449	735 - 748	750.9133	1499.8121	1499.8096	1.64	0	53	9.6e-05	1	U	R.SIPSLVDGFKPGQR.K
5450	735 - 748	750.9137	1499.8128	1499.8096	2.10	0	15	0.036	1	U	R.SIPSLVDGFKPGQR.K
4015	826 - 836	658.3563	1314.6980	1314.7006	-1.92	0	31	0.0012	1	U	R.YIFTMLSTLAR.L
4016	826 - 836	658.3566	1314.6985	1314.7006	-1.54	0	23	0.018	1	U	R.YIFTMLSTLAR.L
4017	826 - 836	658.3573	1314.7000	1314.7006	-0.40	0	45	0.00019	1	U	R.YIFTMLSTLAR.L
4018	826 - 836	658.3573	1314.7001	1314.7006	-0.37	0	46	0.00044	1	U	R.YIFTMLSTLAR.L
4019	826 - 836	658.3574	1314.7002	1314.7006	-0.28	0	36	0.00045	1	U	R.YIFTMLSTLAR.L
4020	826 - 836	658.3574	1314.7002	1314.7006	-0.25	0	46	0.00025	1	U	R.YIFTMLSTLAR.L
4021	826 - 836	658.3574	1314.7003	1314.7006	-0.24	0	38	0.00098	1	U	R.YIFTMLSTLAR.L
4022	826 - 836	658.3575	1314.7005	1314.7006	-0.052	0	31	0.0047	1	U	R.YIFTMLSTLAR.L
4023	826 - 836	658.3576	1314.7006	1314.7006	-0.0068	0	50	0.00012	1	U	R.YIFTMLSTLAR.L

Query	Start - End	Observed	Mr(expt)	Mr(calc)	ppm	M	Score	Expect	Rank	U	Peptide
4024	826 - 836	658.3576	1314.7007	1314.7006	0.069	0	49	0.00011	1	U	R.YIFTMLSTLAR.L
4025	826 - 836	658.3577	1314.7008	1314.7006	0.21	0	50	7.2e-05	1	U	R.YIFTMLSTLAR.L
4026	826 - 836	658.3577	1314.7009	1314.7006	0.24	0	40	0.00067	1	U	R.YIFTMLSTLAR.L
4027	826 - 836	658.3578	1314.7009	1314.7006	0.28	0	38	0.00028	1	U	R.YIFTMLSTLAR.L
4028	826 - 836	658.3585	1314.7024	1314.7006	1.36	0	48	0.00036	1	U	R.YIFTMLSTLAR.L
4029	826 - 836	658.3585	1314.7025	1314.7006	1.44	0	30	0.0081	1	U	R.YIFTMLSTLAR.L
4144	826 - 836	666.3540	1330.6934	1330.6955	-1.54	0	24	0.026	1	U	R.YIFTMLSTLAR.L + Oxidation (M)
4145	826 - 836	666.3544	1330.6942	1330.6955	-0.94	0	54	8.2e-05	1	U	R.YIFTMLSTLAR.L + Oxidation (M)
4148	826 - 836	666.3548	1330.6950	1330.6955	-0.35	0	44	0.00026	1	U	R.YIFTMLSTLAR.L + Oxidation (M)
4149	826 - 836	666.3549	1330.6952	1330.6955	-0.22	0	41	0.00056	1	U	R.YIFTMLSTLAR.L + Oxidation (M)
4150	826 - 836	666.3550	1330.6954	1330.6955	-0.096	0	51	9.2e-05	1	U	R.YIFTMLSTLAR.L + Oxidation (M)
4151	826 - 836	666.3551	1330.6957	1330.6955	0.16	0	52	6.2e-05	1	U	R.YIFTMLSTLAR.L + Oxidation (M)
4152	826 - 836	666.3551	1330.6957	1330.6955	0.16	0	38	0.0012	1	U	R.YIFTMLSTLAR.L + Oxidation (M)
4155	826 - 836	666.3552	1330.6959	1330.6955	0.31	0	24	0.017	1	U	R.YIFTMLSTLAR.L + Oxidation (M)
4156	826 - 836	666.3553	1330.6960	1330.6955	0.37	0	23	0.016	1	U	R.YIFTMLSTLAR.L + Oxidation (M)
4157	826 - 836	666.3553	1330.6960	1330.6955	0.40	0	55	2.8e-05	1	U	R.YIFTMLSTLAR.L + Oxidation (M)
4158	826 - 836	666.3554	1330.6962	1330.6955	0.50	0	51	5.3e-05	1	U	R.YIFTMLSTLAR.L + Oxidation (M)
4159	826 - 836	666.3555	1330.6964	1330.6955	0.72	0	42	0.00025	1	U	R.YIFTMLSTLAR.L + Oxidation (M)
4160	826 - 836	666.3555	1330.6965	1330.6955	0.73	0	35	0.0024	1	U	R.YIFTMLSTLAR.L + Oxidation (M)
4161	826 - 836	666.3559	1330.6972	1330.6955	1.26	0	39	0.00093	1	U	R.YIFTMLSTLAR.L + Oxidation (M)
4320	837 - 848	679.3895	1356.7644	1356.7653	-0.62	0	31	0.0095	1	U	R.LLFPAVDDNLLK.F
4321	837 - 848	679.3898	1356.7649	1356.7653	-0.25	0	35	0.0075	1	U	R.LLFPAVDDNLLK.F
4322	837 - 848	679.3898	1356.7650	1356.7653	-0.24	0	36	0.0047	1	U	R.LLFPAVDDNLLK.F
4323	837 - 848	679.3898	1356.7651	1356.7653	-0.15	0	41	0.00064	1	U	R.LLFPAVDDNLLK.F
4324	837 - 848	679.3898	1356.7651	1356.7653	-0.15	0	32	0.024	1	U	R.LLFPAVDDNLLK.F
4325	837 - 848	679.3898	1356.7651	1356.7653	-0.13	0	39	0.0022	1	U	R.LLFPAVDDNLLK.F
4326	837 - 848	679.3898	1356.7651	1356.7653	-0.12	0	53	8.8e-05	1	U	R.LLFPAVDDNLLK.F
4328	837 - 848	679.3900	1356.7654	1356.7653	0.058	0	33	0.011	1	U	R.LLFPAVDDNLLK.F
4329	837 - 848	679.3900	1356.7654	1356.7653	0.058	0	39	0.0031	1	U	R.LLFPAVDDNLLK.F
4330	837 - 848	679.3900	1356.7655	1356.7653	0.16	0	50	0.00021	1	U	R.LLFPAVDDNLLK.F
4331	837 - 848	679.3900	1356.7655	1356.7653	0.18	0	27	0.013	1	U	R.LLFPAVDDNLLK.F
4332	837 - 848	679.3901	1356.7657	1356.7653	0.31	0	42	0.00071	1	U	R.LLFPAVDDNLLK.F
2043	849 - 856	535.7489	1069.4832	1069.4829	0.28	0	39	0.0082	1		K.FLYDDNQR.V
2044	849 - 856	535.7489	1069.4833	1069.4829	0.42	0	25	0.041	1		K.FLYDDNQR.V
590	885 - 891	424.7166	847.4187	847.4188	-0.053	0	25	0.042	1	U	K.LPNYDAR.E
592	885 - 891	424.7168	847.4190	847.4188	0.25	0	36	0.0064	1	U	K.LPNYDAR.E
7496	900 - 914	870.9260	1739.8375	1739.8375	0.040	0	81	3.9e-07	1	U	R.MLDGLDPHPMLPNYK.N
7608	900 - 914	878.9237	1755.8328	1755.8324	0.22	0	24	0.01	1	U	R.MLDGLDPHPMLPNYK.N + Oxidation (M)
7609	900 - 914	878.9238	1755.8330	1755.8324	0.37	0	37	0.001	1	U	R.MLDGLDPHPMLPNYK.N + Oxidation (M)
7612	900 - 914	878.9241	1755.8336	1755.8324	0.69	0	59	3e-05	1	U	R.MLDGLDPHPMLPNYK.N + Oxidation (M)
7615	900 - 914	878.9250	1755.8353	1755.8324	1.68	0	27	0.0088	1	U	R.MLDGLDPHPMLPNYK.N + Oxidation (M)
7722	900 - 914	886.9206	1771.8266	1771.8273	-0.41	0	22	0.026	1	U	R.MLDGLDPHPMLPNYK.N + 2 Oxidation (M)
7723	900 - 914	886.9208	1771.8270	1771.8273	-0.16	0	31	0.0063	1	U	R.MLDGLDPHPMLPNYK.N + 2 Oxidation (M)
7728	900 - 914	591.6170	1771.8292	1771.8273	1.08	0	31	0.016	1	U	R.MLDGLDPHPMLPNYK.N + 2 Oxidation (M)
7729	900 - 914	886.9221	1771.8296	1771.8273	1.30	0	25	0.038	1	U	R.MLDGLDPHPMLPNYK.N + 2 Oxidation (M)
10332	918 - 939	1212.1072	2422.1999	2422.2129	-5.35	0	112	9.6e-11	1	U	K.GTIQELGQNQYAVSGEIFVVDR.N
10333	918 - 939	808.4094	2422.2062	2422.2129	-2.74	0	34	0.0042	1	U	K.GTIQELGQNQYAVSGEIFVVDR.N
10339	918 - 939	1212.1133	2422.2121	2422.2129	-0.31	0	142	7.2e-13	1	U	K.GTIQELGQNQYAVSGEIFVVDR.N
10342	918 - 939	1212.1137	2422.2128	2422.2129	-0.021	0	64	8.4e-06	1	U	K.GTIQELGQNQYAVSGEIFVVDR.N
10343	918 - 939	808.4116	2422.2128	2422.2129	-0.016	0	21	0.028	1	U	K.GTIQELGQNQYAVSGEIFVVDR.N
10344	918 - 939	808.4116	2422.2129	2422.2129	-0.0033	0	23	0.024	1	U	K.GTIQELGQNQYAVSGEIFVVDR.N
10345	918 - 939	808.4116	2422.2129	2422.2129	0.021	0	22	0.022	1	U	K.GTIQELGQNQYAVSGEIFVVDR.N
10346	918 - 939	808.4116	2422.2131	2422.2129	0.11	0	25	0.044	1	U	K.GTIQELGQNQYAVSGEIFVVDR.N
10348	918 - 939	808.4117	2422.2133	2422.2129	0.19	0	41	0.0013	1	U	K.GTIQELGQNQYAVSGEIFVVDR.N
10349	918 - 939	606.5606	2422.2134	2422.2129	0.23	0	35	0.02	1	U	K.GTIQELGQNQYAVSGEIFVVDR.N
10352	918 - 939	808.4118	2422.2137	2422.2129	0.33	0	27	0.016	1	U	K.GTIQELGQNQYAVSGEIFVVDR.N
10354	918 - 939	808.4122	2422.2147	2422.2129	0.75	0	33	0.014	1	U	K.GTIQELGQNQYAVSGEIFVVDR.N
10355	918 - 939	606.5612	2422.2156	2422.2129	1.14	0	39	0.0028	1	U	K.GTIQELGQNQYAVSGEIFVVDR.N
10357	918 - 939	1212.1162	2422.2178	2422.2129	2.03	0	42	0.00051	1	U	K.GTIQELGQNQYAVSGEIFVVDR.N
3640	940 - 950	635.8524	1269.6902	1269.6929	-2.09	0	35	0.013	1	U	R.NTVEITELPVR.T
3641	940 - 950	635.8532	1269.6918	1269.6929	-0.81	0	67	7.3e-06	1	U	R.NTVEITELPVR.T
3642	940 - 950	635.8532	1269.6919	1269.6929	-0.76	0	32	0.0024	1	U	R.NTVEITELPVR.T
3643	940 - 950	635.8534	1269.6923	1269.6929	-0.42	0	53	0.00027	1	U	R.NTVEITELPVR.T
3644	940 - 950	635.8535	1269.6924	1269.6929	-0.38	0	50	0.00056	1	U	R.NTVEITELPVR.T
3645	940 - 950	635.8536	1269.6926	1269.6929	-0.21	0	55	0.00017	1	U	R.NTVEITELPVR.T
3646	940 - 950	635.8536	1269.6926	1269.6929	-0.18	0	19	0.041	1	U	R.NTVEITELPVR.T
3647	940 - 950	635.8537	1269.6928	1269.6929	-0.038	0	53	6.7e-05	1	U	R.NTVEITELPVR.T
3648	940 - 950	635.8539	1269.6932	1269.6929	0.28	0	23	0.0075	1	U	R.NTVEITELPVR.T
3650	940 - 950	635.8539	1269.6933	1269.6929	0.37	0	47	8.5e-05	1	U	R.NTVEITELPVR.T

Query	Start - End	Observed	Mr(expt)	Mr(calc)	ppm	M	Score	Expect	Rank	U	Peptide
3651	940 - 950	635.8540	1269.6934	1269.6929	0.45	0	22	0.041	1	U	R.NTVEITELPVR.T
3652	940 - 950	424.2385	1269.6936	1269.6929	0.62	0	53	0.00026	1	U	R.NTVEITELPVR.T
5219	958 - 970	737.3661	1472.7176	1472.7181	-0.29	0	40	0.0019	1	U	K.EQVLEPMLNGTDK.T
5324	958 - 970	745.3634	1488.7122	1488.7130	-0.56	0	47	0.0003	1	U	K.EQVLEPMLNGTDK.T + Oxidation (M)
5325	958 - 970	745.3638	1488.7131	1488.7130	0.10	0	44	0.00051	1	U	K.EQVLEPMLNGTDK.T + Oxidation (M)
10561	958 - 979	826.7514	2477.2325	2477.2359	-1.40	1	38	0.0015	1	U	K.EQVLEPMLNGTDKTPALISDYK.E + Oxidation (M)
1541	971 - 979	504.2741	1006.5336	1006.5335	0.094	0	36	0.0031	1	U	K.TPALISDYK.E
1542	971 - 979	504.2743	1006.5341	1006.5335	0.59	0	35	0.0042	1	U	K.TPALISDYK.E
1543	971 - 979	504.2743	1006.5341	1006.5335	0.63	0	26	0.019	1	U	K.TPALISDYK.E
1544	971 - 979	504.2745	1006.5344	1006.5335	0.91	0	27	0.019	1	U	K.TPALISDYK.E
9387	971 - 988	694.6838	2081.0297	2081.0317	-0.98	1	38	0.00076	1	U	K.TPALISDYKEYHTDTTVK.F
9388	971 - 988	694.6842	2081.0308	2081.0317	-0.44	1	30	0.0058	1	U	K.TPALISDYKEYHTDTTVK.F
9390	971 - 988	694.6845	2081.0317	2081.0317	-0.010	1	18	0.045	1	U	K.TPALISDYKEYHTDTTVK.F
2249	980 - 988	547.2617	1092.5088	1092.5088	0.063	0	31	0.0057	1		K.EYHTDTTVK.F
2251	980 - 988	547.2618	1092.5090	1092.5088	0.23	0	19	0.032	1		K.EYHTDTTVK.F
2357	998 - 1008	554.8088	1107.6030	1107.6036	-0.57	0	63	1.9e-05	1	U	K.LAQAEAAGLHK.V
2358	998 - 1008	554.8089	1107.6032	1107.6036	-0.41	0	55	6.6e-05	1	U	K.LAQAEAAGLHK.V
2359	998 - 1008	370.2083	1107.6032	1107.6036	-0.39	0	47	0.00093	1	U	K.LAQAEAAGLHK.V
2361	998 - 1008	370.2084	1107.6035	1107.6036	-0.090	0	32	0.0088	1	U	K.LAQAEAAGLHK.V
2362	998 - 1008	370.2085	1107.6036	1107.6036	-0.063	0	38	0.003	1	U	K.LAQAEAAGLHK.V
2363	998 - 1008	554.8091	1107.6036	1107.6036	-0.013	0	40	0.0015	1	U	K.LAQAEAAGLHK.V
2364	998 - 1008	370.2086	1107.6041	1107.6036	0.40	0	43	0.0023	1	U	K.LAQAEAAGLHK.V
3341	1032 - 1041	618.8448	1235.6750	1235.6761	-0.93	1	21	0.023	1	U	K.KYETVQDILK.E
3343	1032 - 1041	618.8453	1235.6761	1235.6761	-0.019	1	62	4.5e-06	1	U	K.KYETVQDILK.E
3344	1032 - 1041	618.8454	1235.6762	1235.6761	0.029	1	35	0.00091	1	U	K.KYETVQDILK.E
3345	1032 - 1041	618.8454	1235.6762	1235.6761	0.094	1	49	7.3e-05	1	U	K.KYETVQDILK.E
3347	1032 - 1041	412.8994	1235.6763	1235.6761	0.15	1	39	0.0057	1	U	K.KYETVQDILK.E
3348	1032 - 1041	618.8454	1235.6763	1235.6761	0.16	1	33	0.0035	1	U	K.KYETVQDILK.E
2355	1033 - 1041	554.7979	1107.5812	1107.5812	0.046	0	44	0.0022	1	U	K.YETVQDILK.E
5970	1055 - 1068	774.9081	1547.8016	1547.8017	-0.061	1	76	2.1e-06	1	U	R.KEWLVGMLGAESTK.L
5972	1055 - 1068	516.9413	1547.8022	1547.8017	0.29	1	41	0.003	1	U	R.KEWLVGMLGAESTK.L
6116	1055 - 1068	782.9052	1563.7959	1563.7967	-0.49	1	53	5.7e-05	1	U	R.KEWLVGMLGAESTK.L + Oxidation (M)
6117	1055 - 1068	782.9054	1563.7962	1563.7967	-0.29	1	43	0.00053	1	U	R.KEWLVGMLGAESTK.L + Oxidation (M)
6118	1055 - 1068	782.9055	1563.7965	1563.7967	-0.098	1	68	7.6e-06	1	U	R.KEWLVGMLGAESTK.L + Oxidation (M)
6119	1055 - 1068	782.9055	1563.7965	1563.7967	-0.098	1	70	8.2e-06	1	U	R.KEWLVGMLGAESTK.L + Oxidation (M)
6120	1055 - 1068	522.2730	1563.7973	1563.7967	0.42	1	29	0.0036	1	U	R.KEWLVGMLGAESTK.L + Oxidation (M)
6121	1055 - 1068	522.2731	1563.7975	1563.7967	0.56	1	28	0.006	1	U	R.KEWLVGMLGAESTK.L + Oxidation (M)
6122	1055 - 1068	522.2732	1563.7977	1563.7967	0.63	1	37	0.00081	1	U	R.KEWLVGMLGAESTK.L + Oxidation (M)
6123	1055 - 1068	522.2732	1563.7977	1563.7967	0.67	1	35	0.00098	1	U	R.KEWLVGMLGAESTK.L + Oxidation (M)
6124	1055 - 1068	782.9065	1563.7985	1563.7967	1.16	1	62	1.2e-05	1	U	R.KEWLVGMLGAESTK.L + Oxidation (M)
4717	1056 - 1068	474.2428	1419.7066	1419.7068	-0.11	0	28	0.012	1	U	K.EWLVGMLGAESTK.L
4718	1056 - 1068	710.8607	1419.7068	1419.7068	0.029	0	52	3.6e-05	1	U	K.EWLVGMLGAESTK.L
4724	1056 - 1068	710.8612	1419.7079	1419.7068	0.79	0	32	0.0034	1	U	K.EWLVGMLGAESTK.L
4726	1056 - 1068	710.8618	1419.7091	1419.7068	1.65	0	29	0.0018	1	U	K.EWLVGMLGAESTK.L
4865	1056 - 1068	718.8577	1435.7008	1435.7017	-0.63	0	37	0.0081	1	U	K.EWLVGMLGAESTK.L + Oxidation (M)
4866	1056 - 1068	718.8578	1435.7010	1435.7017	-0.51	0	39	0.0055	1	U	K.EWLVGMLGAESTK.L + Oxidation (M)
4867	1056 - 1068	718.8580	1435.7015	1435.7017	-0.15	0	32	0.044	1	U	K.EWLVGMLGAESTK.L + Oxidation (M)
4869	1056 - 1068	718.8580	1435.7015	1435.7017	-0.11	0	20	0.045	1	U	K.EWLVGMLGAESTK.L + Oxidation (M)
2088	1075 - 1083	538.3291	1074.6436	1074.6437	-0.055	1	27	0.0041	1	U	R.FILEKIQGK.I
3414	1092 - 1101	415.2443	1242.7110	1242.7118	-0.62	1	38	0.0061	1	U	K.KDLIQMLVQR.G
3415	1092 - 1101	622.3631	1242.7117	1242.7118	-0.064	1	54	0.00016	1	U	K.KDLIQMLVQR.G
3545	1092 - 1101	420.5761	1258.7065	1258.7067	-0.18	1	51	0.00042	1	U	K.KDLIQMLVQR.G + Oxidation (M)
3546	1092 - 1101	630.3607	1258.7069	1258.7067	0.11	1	40	0.0037	1	U	K.KDLIQMLVQR.G + Oxidation (M)
3547	1092 - 1101	420.5763	1258.7071	1258.7067	0.32	1	33	0.019	1	U	K.KDLIQMLVQR.G + Oxidation (M)
3548	1092 - 1101	630.3610	1258.7073	1258.7067	0.49	1	55	0.00019	1	U	K.KDLIQMLVQR.G + Oxidation (M)
3549	1092 - 1101	420.5769	1258.7088	1258.7067	1.63	1	41	0.0033	1	U	K.KDLIQMLVQR.G + Oxidation (M)
2423	1093 - 1101	558.3156	1114.6167	1114.6169	-0.13	0	58	8.4e-05	1	U	K.DLIQMLVQR.G
2424	1093 - 1101	558.3157	1114.6168	1114.6169	-0.022	0	51	0.00039	1	U	K.DLIQMLVQR.G
2425	1093 - 1101	558.3158	1114.6170	1114.6169	0.18	0	42	0.0034	1	U	K.DLIQMLVQR.G
2426	1093 - 1101	558.3158	1114.6171	1114.6169	0.21	0	48	0.00092	1	U	K.DLIQMLVQR.G
2577	1093 - 1101	566.3119	1130.6093	1130.6118	-2.21	0	35	0.014	1	U	K.DLIQMLVQR.G + Oxidation (M)
2578	1093 - 1101	566.3122	1130.6099	1130.6118	-1.67	0	48	0.001	1	U	K.DLIQMLVQR.G + Oxidation (M)
2579	1093 - 1101	566.3127	1130.6108	1130.6118	-0.83	0	24	0.049	1	U	K.DLIQMLVQR.G + Oxidation (M)
2580	1093 - 1101	566.3129	1130.6112	1130.6118	-0.46	0	32	0.038	1	U	K.DLIQMLVQR.G + Oxidation (M)
2581	1093 - 1101	566.3130	1130.6115	1130.6118	-0.25	0	44	0.0013	1	U	K.DLIQMLVQR.G + Oxidation (M)

Query	Start - End	Observed	Mr(expt)	Mr(calc)	ppm	M	Score	Expect	Rank	U	Peptide
2582	1093 - 1101	566.3132	1130.6119	1130.6118	0.12	0	31	0.015	1	U	K.DLIQMLVQR.G + Oxidation (M)
2583	1093 - 1101	566.3132	1130.6119	1130.6118	0.14	0	48	0.00081	1	U	K.DLIQMLVQR.G + Oxidation (M)
2584	1093 - 1101	566.3133	1130.6120	1130.6118	0.17	0	44	0.0025	1	U	K.DLIQMLVQR.G + Oxidation (M)
2585	1093 - 1101	566.3133	1130.6120	1130.6118	0.19	0	48	0.00061	1	U	K.DLIQMLVQR.G + Oxidation (M)
2586	1093 - 1101	566.3133	1130.6121	1130.6118	0.26	0	51	0.00039	1	U	K.DLIQMLVQR.G + Oxidation (M)
2587	1093 - 1101	566.3133	1130.6121	1130.6118	0.28	0	33	0.016	1	U	K.DLIQMLVQR.G + Oxidation (M)
2589	1093 - 1101	566.3134	1130.6122	1130.6118	0.39	0	51	0.00035	1	U	K.DLIQMLVQR.G + Oxidation (M)
2590	1093 - 1101	566.3134	1130.6122	1130.6118	0.42	0	42	0.0025	1	U	K.DLIQMLVQR.G + Oxidation (M)
2591	1093 - 1101	566.3134	1130.6123	1130.6118	0.49	0	45	0.00074	1	U	K.DLIQMLVQR.G + Oxidation (M)
796	1102 - 1109	447.7138	893.4130	893.4131	-0.030	0	46	8.3e-05	1	U	R.GYESDPVK.A
797	1102 - 1109	447.7138	893.4130	893.4131	-0.030	0	23	0.012	1	U	R.GYESDPVK.A
1398	1157 - 1164	494.2895	986.5644	986.5648	-0.39	1	41	0.0042	1	U	K.EKVEELIK.Q
1400	1157 - 1164	494.2898	986.5649	986.5648	0.18	1	35	0.016	2	U	K.EKVEELIK.Q
2191	1170 - 1178	543.8042	1085.5939	1085.5941	-0.19	2	39	0.0063	1	U	K.GREVNDLKR.K
2192	1170 - 1178	362.8721	1085.5945	1085.5941	0.37	2	34	0.021	1	U	K.GREVNDLKR.K
1226	1179 - 1186	480.7616	959.5087	959.5076	1.16	1	28	0.04	1		R.KSPSDLWK.E
4455	1187 - 1198	689.8400	1377.6655	1377.6664	-0.64	0	52	0.00011	1	U	K.EDLAAFVEELDK.V
4457	1187 - 1198	689.8403	1377.6661	1377.6664	-0.16	0	33	0.0092	1	U	K.EDLAAFVEELDK.V
4458	1187 - 1198	689.8404	1377.6662	1377.6664	-0.075	0	57	4.3e-05	1	U	K.EDLAAFVEELDK.V
4459	1187 - 1198	689.8411	1377.6676	1377.6664	0.88	0	37	0.0023	1	U	K.EDLAAFVEELDK.V
4460	1187 - 1198	689.8412	1377.6678	1377.6664	1.03	0	48	0.0014	1	U	K.EDLAAFVEELDK.V
9493	1187 - 1204	703.0100	2106.0083	2106.0117	-1.58	1	37	0.0051	1	U	K.EDLAAFVEELDKVESQER.E
9494	1187 - 1204	1054.0120	2106.0094	2106.0117	-1.09	1	56	8.1e-06	1	U	K.EDLAAFVEELDKVESQER.E
9495	1187 - 1204	1054.0121	2106.0096	2106.0117	-0.99	1	91	1.7e-08	1	U	K.EDLAAFVEELDKVESQER.E
9496	1187 - 1204	1054.0126	2106.0106	2106.0117	-0.51	1	52	6.7e-05	1	U	K.EDLAAFVEELDKVESQER.E
9497	1187 - 1204	1054.0126	2106.0107	2106.0117	-0.46	1	52	5.4e-05	1	U	K.EDLAAFVEELDKVESQER.E
9498	1187 - 1204	1054.0128	2106.0109	2106.0117	-0.34	1	76	2.1e-07	1	U	K.EDLAAFVEELDKVESQER.E
9499	1187 - 1204	703.0110	2106.0113	2106.0117	-0.16	1	47	0.00065	1	U	K.EDLAAFVEELDKVESQER.E
9500	1187 - 1204	1054.0130	2106.0114	2106.0117	-0.11	1	51	5.4e-05	1	U	K.EDLAAFVEELDKVESQER.E
9501	1187 - 1204	703.0111	2106.0114	2106.0117	-0.10	1	36	0.0034	1	U	K.EDLAAFVEELDKVESQER.E
9502	1187 - 1204	703.0111	2106.0115	2106.0117	-0.088	1	34	0.01	1	U	K.EDLAAFVEELDKVESQER.E
9503	1187 - 1204	703.0112	2106.0117	2106.0117	0.040	1	51	0.00049	1	U	K.EDLAAFVEELDKVESQER.E
9504	1187 - 1204	703.0113	2106.0120	2106.0117	0.15	1	45	0.00082	1	U	K.EDLAAFVEELDKVESQER.E
9505	1187 - 1204	1054.0133	2106.0121	2106.0117	0.20	1	46	8.9e-05	1	U	K.EDLAAFVEELDKVESQER.E
9506	1187 - 1204	1054.0133	2106.0121	2106.0117	0.20	1	72	1.5e-06	1	U	K.EDLAAFVEELDKVESQER.E
9507	1187 - 1204	703.0113	2106.0122	2106.0117	0.24	1	51	0.00012	1	U	K.EDLAAFVEELDKVESQER.E
9508	1187 - 1204	703.0113	2106.0122	2106.0117	0.25	1	38	0.004	1	U	K.EDLAAFVEELDKVESQER.E
9510	1187 - 1204	703.0114	2106.0123	2106.0117	0.28	1	37	0.0043	1	U	K.EDLAAFVEELDKVESQER.E
9511	1187 - 1204	703.0114	2106.0123	2106.0117	0.32	1	29	0.009	1	U	K.EDLAAFVEELDKVESQER.E
9512	1187 - 1204	1054.0134	2106.0123	2106.0117	0.33	1	75	3.9e-07	1	U	K.EDLAAFVEELDKVESQER.E
9513	1187 - 1204	703.0114	2106.0124	2106.0117	0.34	1	30	0.01	1	U	K.EDLAAFVEELDKVESQER.E
9514	1187 - 1204	1054.0135	2106.0124	2106.0117	0.35	1	34	0.0031	1	U	K.EDLAAFVEELDKVESQER.E
9515	1187 - 1204	703.0115	2106.0126	2106.0117	0.47	1	29	0.023	1	U	K.EDLAAFVEELDKVESQER.E
9517	1187 - 1204	703.0119	2106.0139	2106.0117	1.08	1	26	0.017	1	U	K.EDLAAFVEELDKVESQER.E
9518	1187 - 1204	1054.0150	2106.0155	2106.0117	1.83	1	60	1e-05	1	U	K.EDLAAFVEELDKVESQER.E
9519	1187 - 1204	1054.0155	2106.0165	2106.0117	2.31	1	46	0.00019	1	U	K.EDLAAFVEELDKVESQER.E
11909	1187 - 1214	1032.1681	3093.4824	3093.4812	0.41	2	67	1.6e-05	1	U	K.EDLAAFVEELDKVESQEREDVLAGMSGK.A
11959	1187 - 1214	1037.4987	3109.4743	3109.4761	-0.57	2	64	2.9e-05	1	U	K.EDLAAFVEELDKVESQEREDVLAGMSGK.A + Oxidation (M)
11960	1187 - 1214	1037.4990	3109.4753	3109.4761	-0.25	2	36	0.0026	1	U	K.EDLAAFVEELDKVESQEREDVLAGMSGK.A + Oxidation (M)
11964	1187 - 1214	1037.5004	3109.4793	3109.4761	1.03	2	37	0.0024	1	U	K.EDLAAFVEELDKVESQEREDVLAGMSGK.A + Oxidation (M)
7446	1199 - 1214	578.9492	1733.8258	1733.8254	0.22	1	30	0.041	1	U	K.VESQEREDVLAGMSGK.A
7447	1199 - 1214	867.9204	1733.8261	1733.8254	0.43	1	57	2.7e-05	1	U	K.VESQEREDVLAGMSGK.A
7570	1199 - 1214	584.2806	1749.8201	1749.8203	-0.13	1	38	0.0028	1	U	K.VESQEREDVLAGMSGK.A + Oxidation (M)
7572	1199 - 1214	875.9176	1749.8206	1749.8203	0.16	1	56	6.3e-05	1	U	K.VESQEREDVLAGMSGK.A + Oxidation (M)
7573	1199 - 1214	875.9180	1749.8214	1749.8203	0.64	1	62	4.2e-05	1	U	K.VESQEREDVLAGMSGK.A + Oxidation (M)
1531	1205 - 1214	503.7475	1005.4805	1005.4801	0.40	0	59	2e-05	1	U	R.EDVLAGMSGK.A
1651	1205 - 1214	511.7440	1021.4735	1021.4750	-1.51	0	54	0.00013	1	U	R.EDVLAGMSGK.A + Oxidation (M)
1652	1205 - 1214	511.7448	1021.4750	1021.4750	-0.018	0	52	0.00014	1	U	R.EDVLAGMSGK.A + Oxidation (M)
1653	1205 - 1214	511.7448	1021.4750	1021.4750	0.022	0	56	8.6e-05	1	U	R.EDVLAGMSGK.A + Oxidation (M)
1654	1205 - 1214	511.7448	1021.4751	1021.4750	0.14	0	47	0.00042	1	U	R.EDVLAGMSGK.A + Oxidation (M)
1655	1205 - 1214	511.7449	1021.4752	1021.4750	0.18	0	54	8.7e-05	1	U	R.EDVLAGMSGK.A + Oxidation (M)
1656	1205 - 1214	511.7449	1021.4752	1021.4750	0.22	0	54	9.8e-05	1	U	R.EDVLAGMSGK.A + Oxidation (M)
1657	1205 - 1214	511.7452	1021.4759	1021.4750	0.90	0	49	0.00027	1	U	R.EDVLAGMSGK.A + Oxidation (M)
6727	1227 - 1240	824.9219	1647.8293	1647.8290	0.19	1	88	2.4e-08	1	U	K.KLQLEETMPSPYGR.R
6836	1227 - 1240	832.9187	1663.8228	1663.8239	-0.66	1	71	4.7e-07	1	U	K.KLQLEETMPSPYGR.R + Oxidation (M)
6837	1227 - 1240	555.6151	1663.8235	1663.8239	-0.24	1	27	0.0066	1	U	K.KLQLEETMPSPYGR.R + Oxidation (M)
6838	1227 - 1240	555.6152	1663.8237	1663.8239	-0.15	1	29	0.0061	1	U	K.KLQLEETMPSPYGR.R

Query	Start - End	Observed	Mr(expt)	Mr(calc)	ppm	M	Score	Expect	Rank	U	Peptide
											+ Oxidation (M)
6839	1227 - 1240	832.9191	1663.8237	1663.8239	-0.13	1	76	1.4e-06	1	U	K.KLQLEETMPSPYGR.R + Oxidation (M)
6840	1227 - 1240	555.6152	1663.8238	1663.8239	-0.094	1	29	0.016	1	U	K.KLQLEETMPSPYGR.R + Oxidation (M)
6842	1227 - 1240	555.6153	1663.8240	1663.8239	0.068	1	28	0.0073	1	U	K.KLQLEETMPSPYGR.R + Oxidation (M)
6843	1227 - 1240	832.9194	1663.8242	1663.8239	0.19	1	59	8.7e-06	1	U	K.KLQLEETMPSPYGR.R + Oxidation (M)
6844	1227 - 1240	832.9194	1663.8243	1663.8239	0.23	1	55	1.3e-05	1	U	K.KLQLEETMPSPYGR.R + Oxidation (M)
6845	1227 - 1240	832.9197	1663.8249	1663.8239	0.59	1	65	1.8e-06	1	U	K.KLQLEETMPSPYGR.R + Oxidation (M)
6846	1227 - 1240	832.9197	1663.8249	1663.8239	0.60	1	75	3.9e-07	1	U	K.KLQLEETMPSPYGR.R + Oxidation (M)
6847	1227 - 1240	832.9199	1663.8252	1663.8239	0.76	1	66	2.7e-06	1	U	K.KLQLEETMPSPYGR.R + Oxidation (M)
5662	1228 - 1240	760.8741	1519.7337	1519.7341	-0.23	0	62	6.5e-06	1	U	K.LQLEETMPSPYGR.R
5663	1228 - 1240	507.5853	1519.7341	1519.7341	0.011	0	34	0.0015	1	U	K.LQLEETMPSPYGR.R
5664	1228 - 1240	760.8749	1519.7352	1519.7341	0.77	0	55	2.2e-05	1	U	K.LQLEETMPSPYGR.R
5665	1228 - 1240	760.8757	1519.7368	1519.7341	1.82	0	41	0.00075	1	U	K.LQLEETMPSPYGR.R
5842	1228 - 1240	768.8707	1535.7269	1535.7290	-1.34	0	21	0.016	1	U	K.LQLEETMPSPYGR.R + Oxidation (M)
5843	1228 - 1240	768.8714	1535.7282	1535.7290	-0.49	0	60	1.1e-05	1	U	K.LQLEETMPSPYGR.R + Oxidation (M)
5844	1228 - 1240	768.8714	1535.7283	1535.7290	-0.45	0	57	2.5e-05	1	U	K.LQLEETMPSPYGR.R + Oxidation (M)
5845	1228 - 1240	768.8716	1535.7286	1535.7290	-0.24	0	64	2.9e-06	1	U	K.LQLEETMPSPYGR.R + Oxidation (M)
5846	1228 - 1240	768.8718	1535.7289	1535.7290	-0.016	0	84	5.6e-08	1	U	K.LQLEETMPSPYGR.R + Oxidation (M)
5847	1228 - 1240	768.8720	1535.7293	1535.7290	0.24	0	56	1.3e-05	1	U	K.LQLEETMPSPYGR.R + Oxidation (M)
2928	1241 - 1250	586.3471	1170.6797	1170.6794	0.21	1	42	0.0005	1	U	R.RIIPEITAMK.A
2929	1241 - 1250	391.2339	1170.6798	1170.6794	0.35	1	41	0.0014	1	U	R.RIIPEITAMK.A
2930	1241 - 1250	586.3472	1170.6799	1170.6794	0.44	1	20	0.037	1	U	R.RIIPEITAMK.A
3028	1241 - 1250	396.5650	1186.6733	1186.6743	-0.86	1	37	0.0011	1	U	R.RIIPEITAMK.A + Oxidation (M)
3029	1241 - 1250	396.5651	1186.6734	1186.6743	-0.76	1	16	0.034	1	U	R.RIIPEITAMK.A + Oxidation (M)
3030	1241 - 1250	594.3440	1186.6735	1186.6743	-0.71	1	33	0.00077	1	U	R.RIIPEITAMK.A + Oxidation (M)
3031	1241 - 1250	594.3444	1186.6743	1186.6743	-0.067	1	37	0.00075	1	U	R.RIIPEITAMK.A + Oxidation (M)
3032	1241 - 1250	594.3445	1186.6744	1186.6743	0.068	1	43	0.00021	1	U	R.RIIPEITAMK.A + Oxidation (M)
3033	1241 - 1250	396.5654	1186.6744	1186.6743	0.072	1	15	0.04	1	U	R.RIIPEITAMK.A + Oxidation (M)
3034	1241 - 1250	594.3445	1186.6745	1186.6743	0.15	1	49	4.3e-05	1	U	R.RIIPEITAMK.A + Oxidation (M)
1724	1242 - 1250	516.2934	1030.5723	1030.5732	-0.93	0	51	0.00021	1	U	R.IIPEITAMK.A + Oxidation (M)
2703	1261 - 1271	573.3292	1144.6439	1144.6452	-1.07	2	59	6.1e-05	1	U	K.KKGDLDTAAVK.V
1627	1262 - 1271	509.2820	1016.5495	1016.5502	-0.74	1	61	5.2e-05	1	U	K.KGDLDTAAVK.V
1628	1262 - 1271	509.2821	1016.5497	1016.5502	-0.54	1	32	0.041	1	U	K.KGDLDTAAVK.V
1629	1262 - 1271	509.2826	1016.5506	1016.5502	0.40	1	48	0.0008	1	U	K.KGDLDTAAVK.V
1630	1262 - 1271	509.2826	1016.5506	1016.5502	0.42	1	45	0.0017	1	U	K.KGDLDTAAVK.V
1631	1262 - 1271	509.2826	1016.5507	1016.5502	0.46	1	54	0.00023	1	U	K.KGDLDTAAVK.V
783	1263 - 1271	445.2348	888.4550	888.4553	-0.24	0	46	0.0016	1	U	K.GDLDTAAVK.V
11397	1272 - 1299	973.4679	2917.3820	2917.3869	-1.68	0	38	0.0012	1	U	K.VEFDEEFSGAPVEGAGEEALTPSVPINK.G
11398	1272 - 1299	973.4688	2917.3847	2917.3869	-0.76	0	32	0.0041	1	U	K.VEFDEEFSGAPVEGAGEEALTPSVPINK.G
11399	1272 - 1299	973.4689	2917.3848	2917.3869	-0.71	0	49	0.00022	1	U	K.VEFDEEFSGAPVEGAGEEALTPSVPINK.G
11400	1272 - 1299	973.4690	2917.3852	2917.3869	-0.58	0	50	0.00025	1	U	K.VEFDEEFSGAPVEGAGEEALTPSVPINK.G
11402	1272 - 1299	973.4692	2917.3859	2917.3869	-0.36	0	56	5e-05	1	U	K.VEFDEEFSGAPVEGAGEEALTPSVPINK.G
11407	1272 - 1299	973.4694	2917.3864	2917.3869	-0.16	0	40	0.00093	1	U	K.VEFDEEFSGAPVEGAGEEALTPSVPINK.G
11408	1272 - 1299	973.4695	2917.3866	2917.3869	-0.089	0	33	0.0047	1	U	K.VEFDEEFSGAPVEGAGEEALTPSVPINK.G
11411	1272 - 1299	973.4697	2917.3874	2917.3869	0.16	0	22	0.031	1	U	K.VEFDEEFSGAPVEGAGEEALTPSVPINK.G
11412	1272 - 1299	973.4698	2917.3876	2917.3869	0.24	0	28	0.016	1	U	K.VEFDEEFSGAPVEGAGEEALTPSVPINK.G
11414	1272 - 1299	973.4699	2917.3878	2917.3869	0.32	0	40	0.0092	1	U	K.VEFDEEFSGAPVEGAGEEALTPSVPINK.G
11416	1272 - 1299	973.4701	2917.3884	2917.3869	0.52	0	62	1.2e-05	1	U	K.VEFDEEFSGAPVEGAGEEALTPSVPINK.G
11417	1272 - 1299	973.4702	2917.3889	2917.3869	0.68	0	34	0.0018	1	U	K.VEFDEEFSGAPVEGAGEEALTPSVPINK.G
11420	1272 - 1299	973.4704	2917.3894	2917.3869	0.86	0	33	0.029	1	U	K.VEFDEEFSGAPVEGAGEEALTPSVPINK.G
11421	1272 - 1299	730.3547	2917.3897	2917.3869	0.97	0	43	0.0085	1	U	K.VEFDEEFSGAPVEGAGEEALTPSVPINK.G
11423	1272 - 1299	973.4711	2917.3915	2917.3869	1.57	0	44	0.00077	1	U	K.VEFDEEFSGAPVEGAGEEALTPSVPINK.G
3047	1316 - 1327	396.8909	1187.6508	1187.6510	-0.20	1	31	0.023	1	U	R.KTPTSSGKPSAK.K
3048	1316 - 1327	396.8909	1187.6509	1187.6510	-0.096	1	28	0.016	1	U	R.KTPTSSGKPSAK.K
3050	1316 - 1327	396.8909	1187.6510	1187.6510	0.0051	1	40	0.0023	1	U	R.KTPTSSGKPSAK.K
3051	1316 - 1327	594.8329	1187.6513	1187.6510	0.25	1	53	0.00035	1	U	R.KTPTSSGKPSAK.K
3052	1316 - 1327	594.8330	1187.6514	1187.6510	0.34	1	34	0.0066	1	U	R.KTPTSSGKPSAK.K
3614	1316 - 1327	634.8160	1267.6175	1267.6173	0.17	1	46	0.0011	1	U	R.KTPTSSGKPSAK.K + Phospho (ST)
1958	1317 - 1327	530.7853	1059.5561	1059.5560	0.074	0	27	0.0082	1	U	K.TPTSSGKPSAK.K
1959	1317 - 1327	530.7854	1059.5562	1059.5560	0.19	0	43	0.00082	1	U	K.TPTSSGKPSAK.K
11390	1332 - 1356	729.3481	2913.3633	2913.3628	0.16	2	32	0.034	1	U	K.RNPWSDDESKSESDLEETEPVVIPR.D
11391	1332 - 1356	972.1286	2913.3639	2913.3628	0.36	2	73	3.3e-07	1	U	K.RNPWSDDESKSESDLEETEPVVIPR.D
11392	1332 - 1356	729.3485	2913.3650	2913.3628	0.75	2	30	0.0099	1	U	K.RNPWSDDESKSESDLEETEPVVIPR.D
11698	1332 - 1356	998.7837	2993.3292	2993.3291	0.022	2	63	2.4e-05	1	U	K.RNPWSDDESKSESDLEETEPVVIPR.D + Phospho (ST)
11699	1332 - 1356	998.7838	2993.3297	2993.3291	0.20	2	63	1e-05	1	U	K.RNPWSDDESKSESDLEETEPVVIPR.D + Phospho (ST)
2097	1333 - 1341	539.2279	1076.4412	1076.4411	0.14	0	37	0.0012	1	U	R.NPWSDDESK.S

Query	Start - End	Observed	Mr(expt)	Mr(calc)	ppm	M	Score	Expect	Rank	U	Peptide
11130	1333 - 1356	920.0933	2757.2580	2757.2617	-1.32	1	40	0.017	1	U	R.NPWSDDESKSESDLEETEPVVIPR.D
11131	1333 - 1356	920.0943	2757.2612	2757.2617	-0.19	1	76	2.9e-06	1	U	R.NPWSDDESKSESDLEETEPVVIPR.D
11292	1333 - 1356	946.7496	2837.2270	2837.2280	-0.37	1	59	0.00013	1	U	R.NPWSDDESKSESDLEETEPVVIPR.D + Phospho (ST)
11293	1333 - 1356	946.7497	2837.2272	2837.2280	-0.30	1	46	0.0012	1	U	R.NPWSDDESKSESDLEETEPVVIPR.D + Phospho (ST)
11294	1333 - 1356	946.7503	2837.2291	2837.2280	0.38	1	43	0.0038	1	U	R.NPWSDDESKSESDLEETEPVVIPR.D + Phospho (ST)
7173	1342 - 1356	850.4231	1698.8316	1698.8312	0.24	0	89	1.1e-07	1	U	K.SESDLEETEPVVIPR.D
7749	1342 - 1356	890.4108	1778.8070	1778.7975	5.36	0	38	0.019	1	U	K.SESDLEETEPVVIPR.D + Phospho (ST)
139	1363 - 1369	371.7141	741.4136	741.4133	0.47	0	32	0.0049	1	U	R.AAAERPK.Y
12250	1370 - 1396	1076.7357	3227.1852	3227.1858	-0.21	0	46	0.00021	1	U	K.YTFDFSEEEDDDADDDDDDNNDLEELK.V
12251	1370 - 1396	1076.7358	3227.1855	3227.1858	-0.096	0	62	3.7e-06	1	U	K.YTFDFSEEEDDDADDDDDDNNDLEELK.V
10301	1397 - 1418	805.0321	2412.0744	2412.0734	0.44	1	42	0.00069	1	U	K.VKASPITNDGEDEFVPSDGLDK.D + Phospho (ST)
10302	1397 - 1418	805.0322	2412.0748	2412.0734	0.60	1	37	0.0022	1	U	K.VKASPITNDGEDEFVPSDGLDK.D + Phospho (ST)
12656	1397 - 1427	1146.5148	3436.5225	3436.5236	-0.32	2	77	2.6e-06	1	U	K.VKASPITNDGEDEFVPSDGLDKDEYTFSPGK.S + Phospho (ST)
12657	1397 - 1427	860.1381	3436.5233	3436.5236	-0.088	2	69	2.9e-05	1	U	K.VKASPITNDGEDEFVPSDGLDKDEYTFSPGK.S + Phospho (ST)
12658	1397 - 1427	860.1381	3436.5233	3436.5236	-0.088	2	57	5.3e-05	1	U	K.VKASPITNDGEDEFVPSDGLDKDEYTFSPGK.S + Phospho (ST)
12659	1397 - 1427	1146.5152	3436.5239	3436.5236	0.11	2	77	1.1e-06	1	U	K.VKASPITNDGEDEFVPSDGLDKDEYTFSPGK.S + Phospho (ST)
12660	1397 - 1427	860.1383	3436.5239	3436.5236	0.11	2	41	0.0089	1	U	K.VKASPITNDGEDEFVPSDGLDKDEYTFSPGK.S + Phospho (ST)
12661	1397 - 1427	860.1386	3436.5251	3436.5236	0.46	2	46	0.0019	1	U	K.VKASPITNDGEDEFVPSDGLDKDEYTFSPGK.S + Phospho (ST)
12662	1397 - 1427	1146.5198	3436.5377	3436.5236	4.10	2	30	0.021	1	U	K.VKASPITNDGEDEFVPSDGLDKDEYTFSPGK.S + Phospho (ST)
9484	1399 - 1418	1053.4792	2104.9439	2104.9437	0.089	0	69	2.5e-06	1	U	K.ASPITNDGEDEFVPSDGLDK.D
12036	1399 - 1427	1044.1375	3129.3908	3129.3939	-0.98	1	71	5.4e-06	1	U	K.ASPITNDGEDEFVPSDGLDKDEYTFSPGK.S
12037	1399 - 1427	1044.1384	3129.3933	3129.3939	-0.18	1	29	0.021	1	U	K.ASPITNDGEDEFVPSDGLDKDEYTFSPGK.S
12038	1399 - 1427	1044.1384	3129.3933	3129.3939	-0.18	1	80	2.9e-07	1	U	K.ASPITNDGEDEFVPSDGLDKDEYTFSPGK.S
12039	1399 - 1427	783.3558	3129.3943	3129.3939	0.14	1	34	0.014	1	U	K.ASPITNDGEDEFVPSDGLDKDEYTFSPGK.S
12041	1399 - 1427	1044.1393	3129.3960	3129.3939	0.67	1	58	5.4e-05	1	U	K.ASPITNDGEDEFVPSDGLDKDEYTFSPGK.S
12043	1399 - 1427	1044.1399	3129.3978	3129.3939	1.25	1	73	4.4e-06	1	U	K.ASPITNDGEDEFVPSDGLDKDEYTFSPGK.S
12215	1399 - 1427	1070.7933	3209.3581	3209.3602	-0.66	1	72	6.5e-06	1	U	K.ASPITNDGEDEFVPSDGLDKDEYTFSPGK.S + Phospho (ST)
12216	1399 - 1427	1070.7940	3209.3602	3209.3602	0.015	1	33	0.031	1	U	K.ASPITNDGEDEFVPSDGLDKDEYTFSPGK.S + Phospho (ST)
1796	1419 - 1427	522.2377	1042.4608	1042.4607	0.047	0	36	0.003	1	U	K.DEYTFSPGK.S
9055	1440 - 1456	990.4784	1978.9423	1978.9425	-0.072	1	84	1.5e-07	1	U	K.KSQDFGNLFSFPSYSQK.S
8336	1441 - 1456	926.4306	1850.8467	1850.8475	-0.42	0	67	4.7e-07	1	U	K.SQDFGNLFSFPSYSQK.S
8337	1441 - 1456	617.9563	1850.8472	1850.8475	-0.20	0	23	0.019	1	U	K.SQDFGNLFSFPSYSQK.S
8338	1441 - 1456	926.4311	1850.8477	1850.8475	0.10	0	60	3.5e-06	1	U	K.SQDFGNLFSFPSYSQK.S
8339	1441 - 1456	926.4312	1850.8479	1850.8475	0.21	0	87	9.9e-09	1	U	K.SQDFGNLFSFPSYSQK.S
8340	1441 - 1456	926.4313	1850.8480	1850.8475	0.28	0	65	1.6e-06	1	U	K.SQDFGNLFSFPSYSQK.S
8341	1441 - 1456	926.4314	1850.8482	1850.8475	0.37	0	49	3.8e-05	1	U	K.SQDFGNLFSFPSYSQK.S
8342	1441 - 1456	926.4314	1850.8483	1850.8475	0.44	0	40	0.00032	1	U	K.SQDFGNLFSFPSYSQK.S
8344	1441 - 1456	926.4318	1850.8491	1850.8475	0.86	0	74	1.1e-07	1	U	K.SQDFGNLFSFPSYSQK.S
8345	1441 - 1456	617.9570	1850.8491	1850.8475	0.87	0	55	6e-05	1	U	K.SQDFGNLFSFPSYSQK.S
8346	1441 - 1456	926.4320	1850.8493	1850.8475	0.98	0	60	2.5e-06	1	U	K.SQDFGNLFSFPSYSQK.S
8347	1441 - 1456	926.4324	1850.8502	1850.8475	1.42	0	60	2.4e-06	1	U	K.SQDFGNLFSFPSYSQK.S
8348	1441 - 1456	926.4340	1850.8535	1850.8475	3.21	0	65	2.7e-06	1	U	K.SQDFGNLFSFPSYSQK.S
11176	1457 - 1482	932.4094	2794.2064	2794.2093	-1.05	1	35	0.0042	1	U	K.SEDDSAKFDSNEEDSASVFSPSFGLK.Q
11177	1457 - 1482	932.4099	2794.2080	2794.2093	-0.49	1	67	2.4e-06	1	U	K.SEDDSAKFDSNEEDSASVFSPSFGLK.Q
11179	1457 - 1482	699.5595	2794.2089	2794.2093	-0.14	1	34	0.016	1	U	K.SEDDSAKFDSNEEDSASVFSPSFGLK.Q
11180	1457 - 1482	932.4103	2794.2090	2794.2093	-0.12	1	71	7.4e-07	1	U	K.SEDDSAKFDSNEEDSASVFSPSFGLK.Q
11181	1457 - 1482	932.4105	2794.2098	2794.2093	0.16	1	75	8.4e-07	1	U	K.SEDDSAKFDSNEEDSASVFSPSFGLK.Q
11183	1457 - 1482	932.4108	2794.2106	2794.2093	0.46	1	60	2.3e-05	1	U	K.SEDDSAKFDSNEEDSASVFSPSFGLK.Q
11184	1457 - 1482	699.5609	2794.2145	2794.2093	1.86	1	29	0.018	1	U	K.SEDDSAKFDSNEEDSASVFSPSFGLK.Q
11185	1457 - 1482	932.4129	2794.2170	2794.2093	2.73	1	27	0.013	1	U	K.SEDDSAKFDSNEEDSASVFSPSFGLK.Q
11345	1457 - 1482	959.0653	2874.1740	2874.1757	-0.56	1	25	0.026	1	U	K.SEDDSAKFDSNEEDSASVFSPSFGLK.Q + Phospho (ST)
11347	1457 - 1482	959.0659	2874.1759	2874.1757	0.085	1	36	0.0068	1	U	K.SEDDSAKFDSNEEDSASVFSPSFGLK.Q + Phospho (ST)
11348	1457 - 1482	959.0667	2874.1782	2874.1757	0.90	1	35	0.015	1	U	K.SEDDSAKFDSNEEDSASVFSPSFGLK.Q + Phospho (ST)
9320	1464 - 1482	1031.9623	2061.9100	2061.9167	-3.25	0	61	8e-06	1	U	K.FDSNEEDSASVFSPSFGLK.Q
9321	1464 - 1482	1031.9636	2061.9127	2061.9167	-1.97	0	91	2.4e-08	1	U	K.FDSNEEDSASVFSPSFGLK.Q
9322	1464 - 1482	1031.9651	2061.9156	2061.9167	-0.54	0	34	0.0023	1	U	K.FDSNEEDSASVFSPSFGLK.Q
9324	1464 - 1482	1031.9651	2061.9156	2061.9167	-0.53	0	62	3.3e-06	1	U	K.FDSNEEDSASVFSPSFGLK.Q
9325	1464 - 1482	1031.9651	2061.9157	2061.9167	-0.50	0	43	0.00021	1	U	K.FDSNEEDSASVFSPSFGLK.Q
9326	1464 - 1482	688.3126	2061.9159	2061.9167	-0.41	0	23	0.033	1	U	K.FDSNEEDSASVFSPSFGLK.Q
9328	1464 - 1482	1031.9654	2061.9162	2061.9167	-0.24	0	20	0.022	1	U	K.FDSNEEDSASVFSPSFGLK.Q
9329	1464 - 1482	1031.9655	2061.9165	2061.9167	-0.13	0	129	2.6e-12	1	U	K.FDSNEEDSASVFSPSFGLK.Q
9330	1464 - 1482	1031.9656	2061.9166	2061.9167	-0.051	0	68	2e-06	1	U	K.FDSNEEDSASVFSPSFGLK.Q
9331	1464 - 1482	1031.9656	2061.9166	2061.9167	-0.041	0	47	0.0005	1	U	K.FDSNEEDSASVFSPSFGLK.Q
9333	1464 - 1482	1031.9656	2061.9167	2061.9167	-0.022	0	35	0.0035	1	U	K.FDSNEEDSASVFSPSFGLK.Q
9335	1464 - 1482	1031.9656	2061.9167	2061.9167	-0.022	0	97	1.5e-09	1	U	K.FDSNEEDSASVFSPSFGLK.Q
9336	1464 - 1482	1031.9657	2061.9169	2061.9167	0.075	0	92	1.8e-08	1	U	K.FDSNEEDSASVFSPSFGLK.Q
9337	1464 - 1482	1031.9657	2061.9169	2061.9167	0.085	0	34	0.0014	1	U	K.FDSNEEDSASVFSPSFGLK.Q
9338	1464 - 1482	1031.9657	2061.9169	2061.9167	0.095	0	58	1.4e-05	1	U	K.FDSNEEDSASVFSPSFGLK.Q
9339	1464 - 1482	1031.9658	2061.9170	2061.9167	0.15	0	83	6.8e-08	1	U	K.FDSNEEDSASVFSPSFGLK.Q
9340	1464 - 1482	1031.9658	2061.9171	2061.9167	0.18	0	55	0.00011	1	U	K.FDSNEEDSASVFSPSFGLK.Q
9342	1464 - 1482	1031.9658	2061.9171	2061.9167	0.20	0	92	1.7e-08	1	U	K.FDSNEEDSASVFSPSFGLK.Q
9343	1464 - 1482	1031.9659	2061.9172	2061.9167	0.21	0	25	0.0068	1	U	K.FDSNEEDSASVFSPSFGLK.Q

Query	Start - End	Observed	Mr(expt)	Mr(calc)	ppm	M	Score	Expect	Rank	U	Peptide
9344	1464 - 1482	1031.9660	2061.9174	2061.9167	0.32	0	57	3.3e-05	1	U	K.FDSNEEDSASVFSPSFGLK.Q
9345	1464 - 1482	1031.9660	2061.9174	2061.9167	0.33	0	50	0.00041	1	U	K.FDSNEEDSASVFSPSFGLK.Q
9346	1464 - 1482	1031.9660	2061.9174	2061.9167	0.33	0	109	3.6e-10	1	U	K.FDSNEEDSASVFSPSFGLK.Q
9347	1464 - 1482	688.3132	2061.9176	2061.9167	0.43	0	51	0.00028	1	U	K.FDSNEEDSASVFSPSFGLK.Q
9348	1464 - 1482	688.3132	2061.9177	2061.9167	0.46	0	20	0.046	1	U	K.FDSNEEDSASVFSPSFGLK.Q
9349	1464 - 1482	1031.9664	2061.9183	2061.9167	0.77	0	86	3.8e-08	1	U	K.FDSNEEDSASVFSPSFGLK.Q
9602	1464 - 1482	1071.9476	2141.8805	2141.8831	-1.17	0	68	2.7e-06	1	U	K.FDSNEEDSASVFSPSFGLK.Q + Phospho (ST)
9603	1464 - 1482	1071.9482	2141.8818	2141.8831	-0.60	0	86	2.3e-07	1	U	K.FDSNEEDSASVFSPSFGLK.Q + Phospho (ST)
9604	1464 - 1482	1071.9485	2141.8825	2141.8831	-0.27	0	64	4.7e-06	1	U	K.FDSNEEDSASVFSPSFGLK.Q + Phospho (ST)
4376	1496 - 1508	456.9329	1367.7768	1367.7773	-0.37	1	40	0.0011	1	U	K.KGKPSSDTVPKPK.R
4377	1496 - 1508	684.8958	1367.7770	1367.7773	-0.15	1	54	5.1e-05	1	U	K.KGKPSSDTVPKPK.R
3386	1497 - 1508	620.8479	1239.6812	1239.6823	-0.89	0	50	7.7e-05	1	U	K.GKPSSDTVPKPK.R
3388	1497 - 1508	620.8481	1239.6817	1239.6823	-0.51	0	37	0.0012	1	U	K.GKPSSDTVPKPK.R
3393	1497 - 1508	414.2349	1239.6828	1239.6823	0.42	0	48	0.00046	1	U	K.GKPSSDTVPKPK.R
3394	1497 - 1508	414.2350	1239.6832	1239.6823	0.75	0	31	0.0099	1	U	K.GKPSSDTVPKPK.R
4063	1497 - 1508	440.8899	1319.6478	1319.6486	-0.64	0	23	0.034	1	U	K.GKPSSDTVPKPK.R + Phospho (ST)
8780	1515 - 1532	640.9893	1919.9461	1919.9476	-0.80	1	47	0.00026	1	U	K.KVVEAVNSDSDSEFGIPK.K
8782	1515 - 1532	960.9814	1919.9482	1919.9476	0.29	1	90	4.4e-09	1	U	K.KVVEAVNSDSDSEFGIPK.K
9116	1515 - 1532	667.6446	1999.9120	1999.9140	-0.96	1	35	0.0037	1	U	K.KVVEAVNSDSDSEFGIPK.K + Phospho (ST)
9118	1515 - 1532	667.6452	1999.9139	1999.9140	-0.047	1	39	0.0013	1	U	K.KVVEAVNSDSDSEFGIPK.K + Phospho (ST)
9119	1515 - 1532	1000.9650	1999.9155	1999.9140	0.77	1	49	0.00017	1	U	K.KVVEAVNSDSDSEFGIPK.K + Phospho (ST)
7879	1516 - 1532	896.9335	1791.8524	1791.8527	-0.16	0	49	5.1e-05	1	U	K.VVEAVNSDSDSEFGIPK.K
7880	1516 - 1532	896.9337	1791.8529	1791.8527	0.14	0	67	1.2e-06	1	U	K.VVEAVNSDSDSEFGIPK.K
7881	1516 - 1532	896.9340	1791.8535	1791.8527	0.45	0	51	1.6e-05	1	U	K.VVEAVNSDSDSEFGIPK.K
8452	1516 - 1532	936.9165	1871.8185	1871.8190	-0.26	0	52	2.8e-05	1	U	K.VVEAVNSDSDSEFGIPK.K + Phospho (ST)
8453	1516 - 1532	936.9169	1871.8192	1871.8190	0.11	0	79	5.2e-08	1	U	K.VVEAVNSDSDSEFGIPK.K + Phospho (ST)
8454	1516 - 1532	936.9175	1871.8205	1871.8190	0.78	0	48	5.9e-05	1	U	K.VVEAVNSDSDSEFGIPK.K + Phospho (ST)
8111	1547 - 1562	606.2584	1815.7533	1815.7537	-0.18	2	47	0.00037	1	U	K.RKASGSENEGDYNPGR.K + Phospho (ST)
6257	1548 - 1562	790.8496	1579.6847	1579.6862	-0.99	1	35	0.0029	1	U	R.KASGSENEGDYNPGR.K
6795	1548 - 1562	830.8318	1659.6491	1659.6526	-2.06	1	22	0.02	1	U	R.KASGSENEGDYNPGR.K + Phospho (ST)
6797	1548 - 1562	830.8340	1659.6535	1659.6526	0.54	1	49	6.9e-05	1	U	R.KASGSENEGDYNPGR.K + Phospho (ST)
7490	1548 - 1562	870.8170	1739.6195	1739.6189	0.35	1	45	0.00034	1	U	R.KASGSENEGDYNPGR.K + 2 Phospho (ST)
7491	1548 - 1562	870.8181	1739.6216	1739.6189	1.56	1	30	0.0033	1	U	R.KASGSENEGDYNPGR.K + 2 Phospho (ST)
7223	1548 - 1563	570.2679	1707.7818	1707.7812	0.34	2	43	0.00047	1	U	R.KASGSENEGDYNPGRK.T
7817	1548 - 1563	596.9232	1787.7477	1787.7475	0.12	2	24	0.026	1	U	R.KASGSENEGDYNPGRK.T + Phospho (ST)
4984	1549 - 1562	726.8011	1451.5877	1451.5913	-2.47	0	32	0.0047	1	U	K.ASGSENEGDYNPGR.K
4985	1549 - 1562	484.8711	1451.5914	1451.5913	0.061	0	38	0.0014	1	U	K.ASGSENEGDYNPGR.K
4986	1549 - 1562	484.8711	1451.5916	1451.5913	0.19	0	26	0.009	1	U	K.ASGSENEGDYNPGR.K
4987	1549 - 1562	726.8031	1451.5916	1451.5913	0.19	0	62	1.7e-05	1	U	K.ASGSENEGDYNPGR.K
4988	1549 - 1562	726.8032	1451.5918	1451.5913	0.37	0	40	0.0029	1	U	K.ASGSENEGDYNPGR.K
4989	1549 - 1562	726.8032	1451.5918	1451.5913	0.37	0	40	0.00056	1	U	K.ASGSENEGDYNPGR.K
5818	1549 - 1562	766.7843	1531.5541	1531.5576	-2.28	0	45	0.0011	1	U	K.ASGSENEGDYNPGR.K + Phospho (ST)
5819	1549 - 1562	766.7845	1531.5545	1531.5576	-2.04	0	60	3.2e-05	1	U	K.ASGSENEGDYNPGR.K + Phospho (ST)
5820	1549 - 1562	766.7863	1531.5580	1531.5576	0.26	0	44	0.00026	1	U	K.ASGSENEGDYNPGR.K + Phospho (ST)
5821	1549 - 1562	766.7866	1531.5586	1531.5576	0.65	0	28	0.025	1	U	K.ASGSENEGDYNPGR.K + Phospho (ST)
5822	1549 - 1562	766.7867	1531.5589	1531.5576	0.83	0	30	0.0043	1	U	K.ASGSENEGDYNPGR.K + Phospho (ST)
6259	1549 - 1563	790.8505	1579.6864	1579.6862	0.10	1	59	4.9e-05	1	U	K.ASGSENEGDYNPGRK.T
6796	1549 - 1563	830.8339	1659.6532	1659.6526	0.37	1	31	0.043	1	U	K.ASGSENEGDYNPGRK.T + Phospho (ST)
6798	1549 - 1563	554.2251	1659.6535	1659.6526	0.58	1	36	0.0018	1	U	K.ASGSENEGDYNPGRK.T + Phospho (ST)
11470	1574 - 1599	979.7962	2936.3667	2936.3716	-1.67	1	32	0.0067	1	U	K.KTSFDQDSDVDIFPSDFPTEPPSLPR.T
11475	1574 - 1599	979.7970	2936.3691	2936.3716	-0.88	1	44	0.002	1	U	K.KTSFDQDSDVDIFPSDFPTEPPSLPR.T
11476	1574 - 1599	979.7970	2936.3692	2936.3716	-0.84	1	37	0.0035	1	U	K.KTSFDQDSDVDIFPSDFPTEPPSLPR.T
11478	1574 - 1599	979.7973	2936.3702	2936.3716	-0.50	1	34	0.01	1	U	K.KTSFDQDSDVDIFPSDFPTEPPSLPR.T
11482	1574 - 1599	979.7983	2936.3731	2936.3716	0.51	1	26	0.039	1	U	K.KTSFDQDSDVDIFPSDFPTEPPSLPR.T
11483	1574 - 1599	735.1006	2936.3733	2936.3716	0.57	1	30	0.035	1	U	K.KTSFDQDSDVDIFPSDFPTEPPSLPR.T
11226	1575 - 1599	937.0990	2808.2752	2808.2767	-0.53	0	25	0.04	1	U	K.TSFDQDSDVDIFPSDFPTEPPSLPR.T
11232	1575 - 1599	937.1001	2808.2784	2808.2767	0.61	0	24	0.032	1	U	K.TSFDQDSDVDIFPSDFPTEPPSLPR.T
9682	1609 - 1626	1086.9143	2171.8141	2171.8154	-0.59	0	44	8e-05	1	U	K.YFAESDEEEDDVDFAMFN.-
9684	1609 - 1626	1086.9145	2171.8144	2171.8154	-0.42	0	16	0.029	1	U	K.YFAESDEEEDDVDFAMFN.-
9686	1609 - 1626	1086.9150	2171.8155	2171.8154	0.062	0	24	0.026	1	U	K.YFAESDEEEDDVDFAMFN.-
9688	1609 - 1626	724.9461	2171.8165	2171.8154	0.53	0	42	0.0011	1	U	K.YFAESDEEEDDVDFAMFN.-
9735	1609 - 1626	1094.9107	2187.8068	2187.8103	-1.57	0	47	4.8e-05	1	U	K.YFAESDEEEDDVDFAMFN.- + Oxidation (M)
9736	1609 - 1626	1094.9109	2187.8073	2187.8103	-1.37	0	63	1.7e-05	1	U	K.YFAESDEEEDDVDFAMFN.- + Oxidation (M)
9737	1609 - 1626	1094.9123	2187.8100	2187.8103	-0.14	0	52	8.4e-05	1	U	K.YFAESDEEEDDVDFAMFN.- + Oxidation (M)
9739	1609 - 1626	1094.9125	2187.8104	2187.8103	0.053	0	26	0.0036	1	U	K.YFAESDEEEDDVDFAMFN.-

Query	Start - End	Observed	Mr(expt)	Mr(calc)	ppm	M	Score	Expect	Rank	U	Peptide
											+ Oxidation (M)
9741	1609 - 1626	1094.9130	2187.8115	2187.8103	0.55	0	27	0.0035	1	U	K.YFAESDEEEDDVDFAMFN.-<br>+ Oxidation (M)

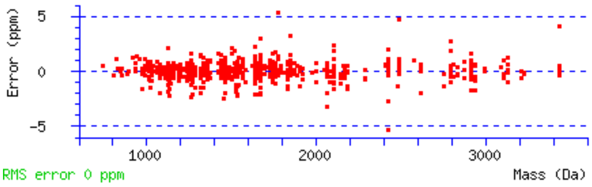

Supplement: Supplementary file 6 — Supplementary Data 3 [file 41467_2023_44089_MOESM6_ESM.pdf]
