# Supplementary material for: ERK2-topoisomerase II regulatory axis is important for gene activation in immediate early genes
Source: Nat Commun. 2023 Dec 14;14:8341. doi: 10.1038/s41467-023-44089-y (PMC10721843; doi:10.1038/s41467-023-44089-y)

MASCOT Search Results

Protein View: Q02880

DNA topoisomerase 2-beta OS=Homo sapiens OX=9606 GN=TOP2B PE=1 SV=3

**Database:**	Uniprot_Proteome_Human
**Score:**	17894
**Monoisotopic mass (M_r):**	184122
**Calculated pI:**	8.14

Sequence similarity is available as **an NCBI BLAST search of Q02880 against nr**.

Search parameters

**MS data file:**	File Name: Z:\SN22\SN221365_deTOP2B_Erk1ml_1ul.raw;File Path: ;File Time: 3/1/2023 3:35:46 PM;File Size: 351320790 [Byte]
**Enzyme:**	Trypsin: cuts C-term side of KR unless next residue is P.
**Fixed modifications:**	**Carbamidomethyl (C)**
**Variable modifications:**	**Acetyl (Protein N-term)**, **Oxidation (M)**, **Phospho (ST)**, **Phospho (Y)**

Protein sequence coverage: 63%

Matched peptides shown in ***bold red***.

```
   1 MAKSGGCGAG AGVGGGNGAL TWVTLFDQNN AAKKEESETA NKNDSSKKLS
  51 VERVYQKKTQ LEHILLRPDT YIGSVEPLTQ FMWVYDEDVG MNCREVTFVP
 101 GLYKIFDEIL VNAADNKQRD KNMTCIKVSI DPESNIISIW NNGKGIPVVE
 151 HKVEKVYVPA LIFGQLLTSS NYDDDEKKVT GGRNGYGAKL CNIFSTKFTV
 201 ETACKEYKHS FKQTWMNNMM KTSEAKIKHF DGEDYTCITF QPDLSKFKME
 251 KLDKDIVALM TRRAYDLAGS CRGVKVMFNG KKLPVNGFRS YVDLYVKDKL
 301 DETGVALKVI HELANERWDV CLTLSEKGFQ QISFVNSIAT TKGGRHVDYV
 351 VDQVVGKLIE VVKKKNKAGV SVKPFQVKNH IWVFINCLIE NPTFDSQTKE
 401 NMTLQPKSFG SKCQLSEKFF KAASNCGIVE SILNWVKFKA QTQLNKKCSS
 451 VKYSKIKGIP KLDDANDAGG KHSLECTLIL TEGDSAKSLA VSGLGVIGRD
 501 RYGVFPLRGK ILNVREASHK QIMENAEINN IIKIVGLQYK KSYDDAESLK
 551 TLRYGKIMIM TDQDQDGSHI KGLLINFIHH NWPSLLKHGF LEEFITPIVK
 601 ASKNKQELSF YSIPEFDEWK KHIENQKAWK IKYYKGLGTS TAKEAKEYFA
 651 DMERHRILFR YAGPEDDAAI TLAFSKKKID DRKEWLTNFM EDRRQRRLHG
 701 LPEQFLYGTA TKHLTYNDFI NKELILFSNS DNERSIPSLV DGFKPGQRKV
 751 LFTCFKRNDK REVKVAQLAG SVAEMSAYHH GEQALMMTIV NLAQNFVGSN
 801 NINLLQPIGQ FGTRLHGGKD AASPRYIFTM LSTLARLLFP AVDDNLLKFL
 851 YDDNQRVEPE WYIPIIPMVL INGAEGIGTG WACKLPNYDA REIVNNVRRM
 901 LDGLDPHPML PNYKNFKGTI QELGQNQYAV SGEIFVVDRN TVEITELPVR
 951 TWTQVYKEQV LEPMLNGTDK TPALISDYKE YHTDTTVKFV VKMTEEKLAQ
1001 AEAAGLHKVF KLQTTLTCNS MVLFDHMGCL KKYETVQDIL KEFFDLRLSY
1051 YGLRKEWLVG MLGAESTKLN NQARFILEKI QGKITIENRS KKDLIQMLVQ
1101 RGYESDPVKA WKEAQEKAAE EDETQNQHDD SSSDSGTPSG PDFNYILNMS
1151 LWSLTKEKVE ELIKQRDAKG REVNDLKRKS PSDLWKEDLA AFVEELDKVE
1201 SQEREDVLAG MSGKAIKGKV GKPKVKKLQL EETMPSPYGR RIIPEITAMK
1251 ADASKKLLKK KKGDLDTAAV KVEFDEEFSG APVEGAGEEA LTPSVPINKG
1301 PKPKREKKEP GTRVRKTPTS SGKPSAKKVK KRNPWSDDES KSESDLEETE
1351 PVVIPRDSLL RRAAAERPKY TFDFSEEEDD DADDDDDDNN DLEELKVKAS
1401 PITNDGEDEF VPSDGLDKDE YTFSPGKSKA TPEKSLHDKK SQDFGNLFSF
1451 PSYSQKSEDD SAKFDSNEED SASVFSPSFG LKQTDKVPSK TVAAKKGKPS
1501 SDTVPKPKRA PKQKKVVEAV NSDSDSEFGI PKKTTTPKGK GRGAKKRKAS
1551 GSENEGDYNP GRKTSKTTSK KPKKTSFDQD SDVDIFPSDF PTEPPSLPRT
1601 GRARKEVKYF AESDEEEDDV DFAMFN
```

Unformatted sequence string: **1626 residues** (for pasting into other applications).

Sort by ◉ residue number ○ increasing mass ○ decreasing mass
Show ◉ matched peptides only ○ predicted peptides also

Query	Start – End	Observed	Mr(expt)	Mr(calc)	ppm	M	Score	Expect	Rank	U	Peptide
1771	34 – 42	518.2516	1034.4887	1034.4880	0.71	1	38	0.0089	1	U	K.KEESETANK.N
2778	95 – 104	576.8184	1151.6222	1151.6227	-0.36	0	16	0.032	1		R.EVTFVPGLYK.I
2779	95 – 104	576.8185	1151.6224	1151.6227	-0.21	0	16	0.031	1		R.EVTFVPGLYK.I
2782	95 – 104	576.8186	1151.6227	1151.6227	0.020	0	19	0.04	1		R.EVTFVPGLYK.I
2784	95 – 104	576.8186	1151.6227	1151.6227	0.055	0	19	0.016	1		R.EVTFVPGLYK.I
2788	95 – 104	576.8188	1151.6231	1151.6227	0.37	0	28	0.013	1		R.EVTFVPGLYK.I
5151	105 – 117	487.9234	1460.7483	1460.7511	-1.88	0	34	0.0044	1		K.IFDEILVNAADNK.Q
5152	105 – 117	487.9235	1460.7486	1460.7511	-1.69	0	27	0.019	1		K.IFDEILVNAADNK.Q
5153	105 – 117	731.3818	1460.7490	1460.7511	-1.39	0	82	3.6e-07	1		K.IFDEILVNAADNK.Q
5155	105 – 117	487.9240	1460.7501	1460.7511	-0.71	0	27	0.036	1		K.IFDEILVNAADNK.Q
5156	105 – 117	487.9240	1460.7501	1460.7511	-0.71	0	25	0.014	1		K.IFDEILVNAADNK.Q
5157	105 – 117	731.3824	1460.7503	1460.7511	-0.53	0	63	1.2e-05	1		K.IFDEILVNAADNK.Q
5158	105 – 117	487.9240	1460.7503	1460.7511	-0.52	0	49	0.00017	1		K.IFDEILVNAADNK.Q
5159	105 – 117	487.9241	1460.7504	1460.7511	-0.44	0	28	0.013	1		K.IFDEILVNAADNK.Q
5160	105 – 117	731.3825	1460.7505	1460.7511	-0.39	0	71	1.6e-06	1		K.IFDEILVNAADNK.Q

Query	Start – End	Observed	Mr(expt)	Mr(calc)	ppm	M	Score	Expect	Rank	U	Peptide
5161	105 – 117	487.9241	1460.7505	1460.7511	-0.38	0	35	0.01	1		K.IFDEILVNAADNK.Q
5163	105 – 117	731.3827	1460.7508	1460.7511	-0.22	0	68	2.3e-06	1		K.IFDEILVNAADNK.Q
5164	105 – 117	487.9242	1460.7508	1460.7511	-0.19	0	46	0.00048	1		K.IFDEILVNAADNK.Q
5165	105 – 117	487.9243	1460.7510	1460.7511	-0.069	0	33	0.0093	1		K.IFDEILVNAADNK.Q
5166	105 – 117	487.9243	1460.7510	1460.7511	-0.049	0	33	0.0031	1		K.IFDEILVNAADNK.Q
5167	105 – 117	487.9243	1460.7511	1460.7511	-0.0075	0	33	0.0065	1		K.IFDEILVNAADNK.Q
5168	105 – 117	487.9243	1460.7511	1460.7511	0.034	0	23	0.027	1		K.IFDEILVNAADNK.Q
5169	105 – 117	731.3828	1460.7511	1460.7511	0.044	0	74	1.7e-06	1		K.IFDEILVNAADNK.Q
5170	105 – 117	487.9243	1460.7512	1460.7511	0.054	0	27	0.031	1		K.IFDEILVNAADNK.Q
5171	105 – 117	487.9243	1460.7512	1460.7511	0.054	0	27	0.016	1		K.IFDEILVNAADNK.Q
5172	105 – 117	731.3829	1460.7512	1460.7511	0.072	0	71	1.6e-06	1		K.IFDEILVNAADNK.Q
5173	105 – 117	487.9244	1460.7512	1460.7511	0.095	0	25	0.03	1		K.IFDEILVNAADNK.Q
5174	105 – 117	731.3829	1460.7512	1460.7511	0.11	0	71	1.4e-06	1		K.IFDEILVNAADNK.Q
5175	105 – 117	487.9244	1460.7513	1460.7511	0.14	0	29	0.026	1		K.IFDEILVNAADNK.Q
5176	105 – 117	487.9244	1460.7513	1460.7511	0.14	0	38	0.002	1		K.IFDEILVNAADNK.Q
5177	105 – 117	487.9244	1460.7513	1460.7511	0.16	0	28	0.031	1		K.IFDEILVNAADNK.Q
5178	105 – 117	731.3829	1460.7513	1460.7511	0.17	0	78	4.6e-07	1		K.IFDEILVNAADNK.Q
5179	105 – 117	731.3830	1460.7514	1460.7511	0.24	0	71	1.7e-06	1		K.IFDEILVNAADNK.Q
5180	105 – 117	487.9244	1460.7515	1460.7511	0.26	0	27	0.014	1		K.IFDEILVNAADNK.Q
5181	105 – 117	731.3830	1460.7515	1460.7511	0.26	0	62	3.2e-05	1		K.IFDEILVNAADNK.Q
5182	105 – 117	731.3830	1460.7515	1460.7511	0.29	0	70	4.1e-06	1		K.IFDEILVNAADNK.Q
5183	105 – 117	731.3831	1460.7516	1460.7511	0.37	0	65	1.8e-05	1		K.IFDEILVNAADNK.Q
5184	105 – 117	487.9245	1460.7518	1460.7511	0.46	0	37	0.018	1		K.IFDEILVNAADNK.Q
5185	105 – 117	487.9245	1460.7518	1460.7511	0.49	0	31	0.004	1		K.IFDEILVNAADNK.Q
5186	105 – 117	731.3832	1460.7519	1460.7511	0.55	0	70	2.5e-06	1		K.IFDEILVNAADNK.Q
5187	105 – 117	731.3833	1460.7521	1460.7511	0.70	0	66	4.3e-06	1		K.IFDEILVNAADNK.Q
1704	120 – 127	513.2408	1024.4670	1024.4681	-1.14	1	30	0.036	1	U	R.DKNMTCIK.V<br>+ Oxidation (M)
8521	128 – 144	629.3266	1884.9581	1884.9581	-0.012	0	59	1.9e-05	1	U	K.VSIDPESNIISIWNNGK.G
8522	128 – 144	943.4865	1884.9584	1884.9581	0.14	0	77	2e-07	1	U	K.VSIDPESNIISIWNNGK.G
758	145 – 152	439.7584	877.5023	877.5022	0.18	0	34	0.011	1		K.GIPVVEHK.V
10538	156 – 177	1244.1162	2486.2178	2486.2217	-1.57	0	74	4.4e-06	1	U	K.VYVPALIFGQLLTSSNYDDDEK.K
10541	156 – 177	829.7480	2486.2220	2486.2217	0.14	0	37	0.0027	1	U	K.VYVPALIFGQLLTSSNYDDDEK.K
10542	156 – 177	829.7480	2486.2221	2486.2217	0.15	0	19	0.035	1	U	K.VYVPALIFGQLLTSSNYDDDEK.K
10543	156 – 177	1244.1184	2486.2222	2486.2217	0.20	0	52	0.0001	1	U	K.VYVPALIFGQLLTSSNYDDDEK.K
10545	156 – 177	1244.1186	2486.2226	2486.2217	0.39	0	86	2.7e-08	1	U	K.VYVPALIFGQLLTSSNYDDDEK.K
10547	156 – 177	829.7482	2486.2228	2486.2217	0.44	0	54	6.1e-05	1	U	K.VYVPALIFGQLLTSSNYDDDEK.K
10548	156 – 177	1244.1187	2486.2229	2486.2217	0.49	0	78	1.6e-07	1	U	K.VYVPALIFGQLLTSSNYDDDEK.K
10550	156 – 177	1244.1192	2486.2239	2486.2217	0.89	0	62	4.7e-06	1	U	K.VYVPALIFGQLLTSSNYDDDEK.K
10551	156 – 177	1244.1208	2486.2270	2486.2217	2.16	0	41	0.0011	1	U	K.VYVPALIFGQLLTSSNYDDDEK.K
10813	156 – 178	872.4447	2614.3124	2614.3166	-1.62	1	40	0.0058	1	U	K.VYVPALIFGQLLTSSNYDDDEKK.V
10815	156 – 178	872.4458	2614.3156	2614.3166	-0.38	1	54	0.00016	1	U	K.VYVPALIFGQLLTSSNYDDDEKK.V
10817	156 – 178	872.4462	2614.3168	2614.3166	0.052	1	46	0.0026	1	U	K.VYVPALIFGQLLTSSNYDDDEKK.V
10818	156 – 178	872.4462	2614.3169	2614.3166	0.11	1	47	0.0013	1	U	K.VYVPALIFGQLLTSSNYDDDEKK.V
10819	156 – 178	654.5865	2614.3169	2614.3166	0.12	1	47	0.0024	1	U	K.VYVPALIFGQLLTSSNYDDDEKK.V
10821	156 – 178	654.5866	2614.3171	2614.3166	0.18	1	22	0.031	1	U	K.VYVPALIFGQLLTSSNYDDDEKK.V
10824	156 – 178	872.4465	2614.3177	2614.3166	0.40	1	44	0.0024	1	U	K.VYVPALIFGQLLTSSNYDDDEKK.V
10826	156 – 178	1308.1666	2614.3185	2614.3166	0.73	1	64	3.8e-05	1	U	K.VYVPALIFGQLLTSSNYDDDEKK.V
1393	190 – 197	491.7548	981.4950	981.4953	-0.36	0	39	0.0011	1		K.LCNIFSTK.F
1201	198 – 205	478.2315	954.4484	954.4481	0.35	0	26	0.016	1	U	K.FTVETACK.E
3118	213 – 221	600.2537	1198.4929	1198.4933	-0.33	0	28	0.012	1	U	K.QTWMNNMMK.T<br>+ Oxidation (M)
3120	213 – 221	600.2541	1198.4937	1198.4933	0.32	0	27	0.028	1	U	K.QTWMNNMMK.T<br>+ Oxidation (M)
3121	213 – 221	600.2543	1198.4940	1198.4933	0.61	0	32	0.008	1	U	K.QTWMNNMMK.T<br>+ Oxidation (M)
3122	213 – 221	600.2544	1198.4943	1198.4933	0.82	0	32	0.008	1	U	K.QTWMNNMMK.T<br>+ Oxidation (M)
3221	213 – 221	608.2513	1214.4881	1214.4882	-0.092	0	20	0.049	1	U	K.QTWMNNMMK.T<br>+ 2 Oxidation (M)
3222	213 – 221	608.2515	1214.4884	1214.4882	0.15	0	18	0.024	1	U	K.QTWMNNMMK.T<br>+ 2 Oxidation (M)
3223	213 – 221	608.2515	1214.4884	1214.4882	0.15	0	31	0.0086	1	U	K.QTWMNNMMK.T<br>+ 2 Oxidation (M)
3224	213 – 221	608.2517	1214.4888	1214.4882	0.45	0	16	0.047	1	U	K.QTWMNNMMK.T<br>+ 2 Oxidation (M)
3225	213 – 221	608.2517	1214.4888	1214.4882	0.48	0	27	0.026	1	U	K.QTWMNNMMK.T<br>+ 2 Oxidation (M)
3226	213 – 221	608.2525	1214.4905	1214.4882	1.85	0	30	0.014	1	U	K.QTWMNNMMK.T<br>+ 2 Oxidation (M)
10285	227 – 246	805.3812	2413.1218	2413.1260	-1.75	1	55	6.9e-05	1	U	K.IKHFDGEDYTCITFQPDLSK.F
10286	227 – 246	805.3821	2413.1245	2413.1260	-0.62	1	45	0.00038	1	U	K.IKHFDGEDYTCITFQPDLSK.F
9649	229 – 246	724.9902	2171.9488	2171.9470	0.84	0	40	0.00089	1	U	K.HFDGEDYTCITFQPDLSK.F
3750	252 – 262	637.8599	1273.7052	1273.7064	-0.94	1	34	0.0034	1	U	K.LDKDIVALMTR.R
3751	252 – 262	637.8599	1273.7053	1273.7064	-0.87	1	36	0.0011	1	U	K.LDKDIVALMTR.R
3755	252 – 262	637.8603	1273.7060	1273.7064	-0.33	1	42	0.00078	1	U	K.LDKDIVALMTR.R
3758	252 – 262	637.8604	1273.7063	1273.7064	-0.033	1	37	0.0015	1	U	K.LDKDIVALMTR.R
3760	252 – 262	637.8606	1273.7065	1273.7064	0.12	1	20	0.041	1	U	K.LDKDIVALMTR.R
3761	252 – 262	637.8606	1273.7066	1273.7064	0.16	1	77	7.3e-07	1	U	K.LDKDIVALMTR.R
3762	252 – 262	637.8606	1273.7066	1273.7064	0.16	1	21	0.018	1	U	K.LDKDIVALMTR.R
3763	252 – 262	637.8606	1273.7067	1273.7064	0.25	1	64	1.6e-05	1	U	K.LDKDIVALMTR.R
3765	252 – 262	637.8607	1273.7069	1273.7064	0.41	1	49	0.00042	1	U	K.LDKDIVALMTR.R
3766	252 – 262	425.5763	1273.7070	1273.7064	0.44	1	33	0.023	1	U	K.LDKDIVALMTR.R
3881	252 – 262	430.9067	1289.6982	1289.7013	-2.38	1	24	0.024	1	U	K.LDKDIVALMTR.R<br>+ Oxidation (M)
3882	252 – 262	645.8573	1289.7000	1289.7013	-1.01	1	55	8.3e-05	1	U	K.LDKDIVALMTR.R<br>+ Oxidation (M)
3883	252 – 262	430.9073	1289.7002	1289.7013	-0.87	1	23	0.017	1	U	K.LDKDIVALMTR.R<br>+ Oxidation (M)
3884	252 – 262	645.8574	1289.7002	1289.7013	-0.85	1	52	8.5e-05	1	U	K.LDKDIVALMTR.R

Query	Start - End	Observed	Mr(expt)	Mr(calc)	ppm	M	Score	Expect	Rank	U	Peptide
											+ Oxidation (M)
3885	252 - 262	645.8577	1289.7008	1289.7013	-0.40	1	34	0.0022	1	U	K.LDKDIVALMTR.R + Oxidation (M)
3886	252 - 262	645.8577	1289.7008	1289.7013	-0.37	1	31	0.0039	1	U	K.LDKDIVALMTR.R + Oxidation (M)
3887	252 - 262	430.9076	1289.7009	1289.7013	-0.33	1	27	0.023	1	U	K.LDKDIVALMTR.R + Oxidation (M)
3889	252 - 262	645.8578	1289.7010	1289.7013	-0.20	1	50	6.2e-05	1	U	K.LDKDIVALMTR.R + Oxidation (M)
3890	252 - 262	430.9076	1289.7011	1289.7013	-0.19	1	32	0.0043	1	U	K.LDKDIVALMTR.R + Oxidation (M)
3891	252 - 262	645.8579	1289.7012	1289.7013	-0.047	1	57	1.9e-05	1	U	K.LDKDIVALMTR.R + Oxidation (M)
3892	252 - 262	430.9077	1289.7013	1289.7013	-0.0054	1	33	0.022	1	U	K.LDKDIVALMTR.R + Oxidation (M)
3894	252 - 262	645.8581	1289.7017	1289.7013	0.29	1	50	6.3e-05	1	U	K.LDKDIVALMTR.R + Oxidation (M)
3895	252 - 262	645.8581	1289.7017	1289.7013	0.31	1	62	5.2e-06	1	U	K.LDKDIVALMTR.R + Oxidation (M)
3896	252 - 262	430.9079	1289.7018	1289.7013	0.39	1	30	0.0084	1	U	K.LDKDIVALMTR.R + Oxidation (M)
975	255 - 262	459.7573	917.5000	917.5004	-0.50	0	49	0.00031	1	U	K.DIVALMTR.R
1083	255 - 262	467.7549	933.4953	933.4953	-0.062	0	40	0.006	1	U	K.DIVALMTR.R + Oxidation (M)
1084	255 - 262	467.7549	933.4953	933.4953	-0.019	0	35	0.018	1	U	K.DIVALMTR.R + Oxidation (M)
1604	264 - 272	506.7295	1011.4444	1011.4444	0.061	0	43	0.0014	1	U	R.AYDLAGSCR.G
1410	290 - 297	493.7634	985.5123	985.5120	0.27	0	30	0.0082	1	U	R.SYVDLYVK.D
1412	290 - 297	493.7636	985.5125	985.5120	0.52	0	43	0.00079	1	U	R.SYVDLYVK.D
3045	298 - 308	396.8870	1187.6392	1187.6398	-0.47	1	30	0.033	1	U	K.DKLDETGVALK.V
3048	298 - 308	594.8270	1187.6394	1187.6398	-0.29	1	50	0.00036	1	U	K.DKLDETGVALK.V
3049	298 - 308	594.8270	1187.6394	1187.6398	-0.29	1	22	0.046	1	U	K.DKLDETGVALK.V
1149	300 - 308	473.2665	944.5184	944.5179	0.59	0	50	0.00058	1	U	K.LDETGVALK.V
2131	309 - 317	540.7921	1079.5696	1079.5723	-2.48	0	30	0.032	1	U	K.VIHELANER.W
2134	309 - 317	360.8642	1079.5709	1079.5723	-1.33	0	25	0.011	1	U	K.VIHELANER.W
2139	309 - 317	360.8646	1079.5720	1079.5723	-0.30	0	31	0.029	1	U	K.VIHELANER.W
2140	309 - 317	540.7933	1079.5720	1079.5723	-0.26	0	35	0.013	1	U	K.VIHELANER.W
2141	309 - 317	360.8646	1079.5721	1079.5723	-0.22	0	19	0.048	1	U	K.VIHELANER.W
2142	309 - 317	360.8647	1079.5723	1079.5723	-0.023	0	33	0.012	1	U	K.VIHELANER.W
2144	309 - 317	540.7935	1079.5724	1079.5723	0.094	0	45	0.0012	1	U	K.VIHELANER.W
2146	309 - 317	540.7935	1079.5725	1079.5723	0.15	0	57	6.6e-05	1	U	K.VIHELANER.W
2147	309 - 317	540.7936	1079.5727	1079.5723	0.32	0	39	0.0056	1	U	K.VIHELANER.W
2148	309 - 317	360.8648	1079.5727	1079.5723	0.34	0	32	0.015	1	U	K.VIHELANER.W
3542	318 - 327	625.8076	1249.6006	1249.6013	-0.52	0	44	0.00033	1	U	R.WDVCLTLSEK.G
6756	328 - 342	547.6252	1639.8539	1639.8570	-1.90	0	21	0.018	1	U	K.GFQQISFVNSIATTK.G
6758	328 - 342	820.9352	1639.8559	1639.8570	-0.68	0	46	0.00016	1	U	K.GFQQISFVNSIATTK.G
6759	328 - 342	820.9354	1639.8562	1639.8570	-0.49	0	23	0.037	1	U	K.GFQQISFVNSIATTK.G
6760	328 - 342	547.6260	1639.8562	1639.8570	-0.46	0	26	0.0059	1	U	K.GFQQISFVNSIATTK.G
6761	328 - 342	820.9355	1639.8564	1639.8570	-0.36	0	44	0.0012	1	U	K.GFQQISFVNSIATTK.G
6762	328 - 342	547.6262	1639.8567	1639.8570	-0.20	0	49	5.5e-05	1	U	K.GFQQISFVNSIATTK.G
6763	328 - 342	820.9356	1639.8567	1639.8570	-0.17	0	57	4.2e-05	1	U	K.GFQQISFVNSIATTK.G
6764	328 - 342	820.9356	1639.8567	1639.8570	-0.15	0	48	0.00045	1	U	K.GFQQISFVNSIATTK.G
6765	328 - 342	820.9357	1639.8568	1639.8570	-0.12	0	71	6.4e-06	1	U	K.GFQQISFVNSIATTK.G
6766	328 - 342	820.9357	1639.8568	1639.8570	-0.092	0	69	1e-05	1	U	K.GFQQISFVNSIATTK.G
6767	328 - 342	820.9357	1639.8569	1639.8570	-0.055	0	54	2.7e-05	1	U	K.GFQQISFVNSIATTK.G
6768	328 - 342	820.9358	1639.8570	1639.8570	-0.0067	0	81	5.6e-07	1	U	K.GFQQISFVNSIATTK.G
6769	328 - 342	820.9358	1639.8570	1639.8570	0.018	0	35	0.027	1	U	K.GFQQISFVNSIATTK.G
6770	328 - 342	547.6263	1639.8570	1639.8570	0.038	0	37	0.0029	1	U	K.GFQQISFVNSIATTK.G
6771	328 - 342	820.9358	1639.8570	1639.8570	0.042	0	27	0.017	1	U	K.GFQQISFVNSIATTK.G
6772	328 - 342	547.6263	1639.8571	1639.8570	0.057	0	56	2.6e-05	1	U	K.GFQQISFVNSIATTK.G
6774	328 - 342	820.9358	1639.8571	1639.8570	0.10	0	55	0.0002	1	U	K.GFQQISFVNSIATTK.G
6775	328 - 342	820.9359	1639.8572	1639.8570	0.13	0	43	0.00048	1	U	K.GFQQISFVNSIATTK.G
6776	328 - 342	820.9359	1639.8572	1639.8570	0.16	0	76	1.3e-06	1	U	K.GFQQISFVNSIATTK.G
6777	328 - 342	820.9360	1639.8573	1639.8570	0.23	0	34	0.0018	1	U	K.GFQQISFVNSIATTK.G
6778	328 - 342	820.9360	1639.8574	1639.8570	0.29	0	37	0.0036	1	U	K.GFQQISFVNSIATTK.G
6779	328 - 342	820.9361	1639.8576	1639.8570	0.40	0	45	0.0002	1	U	K.GFQQISFVNSIATTK.G
6780	328 - 342	820.9361	1639.8577	1639.8570	0.46	0	33	0.0025	1	U	K.GFQQISFVNSIATTK.G
6781	328 - 342	547.6266	1639.8579	1639.8570	0.53	0	26	0.011	1	U	K.GFQQISFVNSIATTK.G
6783	328 - 342	820.9363	1639.8580	1639.8570	0.60	0	59	3.3e-05	1	U	K.GFQQISFVNSIATTK.G
6787	328 - 342	820.9368	1639.8590	1639.8570	1.24	0	17	0.027	1	U	K.GFQQISFVNSIATTK.G
6788	328 - 342	820.9368	1639.8591	1639.8570	1.30	0	54	6.6e-05	1	U	K.GFQQISFVNSIATTK.G
6789	328 - 342	820.9368	1639.8591	1639.8570	1.32	0	32	0.011	1	U	K.GFQQISFVNSIATTK.G
6790	328 - 342	820.9369	1639.8592	1639.8570	1.33	0	22	0.041	1	U	K.GFQQISFVNSIATTK.G
4374	346 - 357	453.2416	1356.7030	1356.7038	-0.56	0	44	0.0029	1	U	R.HVDYVVDQVVGK.L
4375	346 - 357	679.3588	1356.7031	1356.7038	-0.52	0	57	4.7e-06	1	U	R.HVDYVVDQVVGK.L
4376	346 - 357	679.3589	1356.7032	1356.7038	-0.46	0	79	9.7e-08	1	U	R.HVDYVVDQVVGK.L
4378	346 - 357	679.3590	1356.7034	1356.7038	-0.29	0	74	7.1e-07	1	U	R.HVDYVVDQVVGK.L
4380	346 - 357	679.3590	1356.7035	1356.7038	-0.21	0	58	9.6e-06	1	U	R.HVDYVVDQVVGK.L
4381	346 - 357	679.3591	1356.7036	1356.7038	-0.12	0	49	0.00017	1	U	R.HVDYVVDQVVGK.L
4382	346 - 357	679.3591	1356.7037	1356.7038	-0.050	0	81	9.8e-08	1	U	R.HVDYVVDQVVGK.L
4383	346 - 357	679.3592	1356.7038	1356.7038	-0.021	0	58	2.3e-05	1	U	R.HVDYVVDQVVGK.L
4384	346 - 357	679.3592	1356.7038	1356.7038	-0.0059	0	44	0.00022	1	U	R.HVDYVVDQVVGK.L
4385	346 - 357	679.3592	1356.7038	1356.7038	0.024	0	39	0.00065	1	U	R.HVDYVVDQVVGK.L
4386	346 - 357	679.3592	1356.7038	1356.7038	0.038	0	63	2.1e-06	1	U	R.HVDYVVDQVVGK.L
4387	346 - 357	453.2419	1356.7039	1356.7038	0.078	0	24	0.038	1	U	R.HVDYVVDQVVGK.L
4388	346 - 357	679.3592	1356.7039	1356.7038	0.083	0	57	1.6e-05	1	U	R.HVDYVVDQVVGK.L
4389	346 - 357	679.3593	1356.7040	1356.7038	0.14	0	81	1.7e-07	1	U	R.HVDYVVDQVVGK.L
4390	346 - 357	679.3594	1356.7043	1356.7038	0.36	0	56	1.3e-05	1	U	R.HVDYVVDQVVGK.L
4391	346 - 357	679.3595	1356.7044	1356.7038	0.44	0	58	1.5e-05	1	U	R.HVDYVVDQVVGK.L
4392	346 - 357	679.3595	1356.7045	1356.7038	0.54	0	52	3.7e-05	1	U	R.HVDYVVDQVVGK.L

Query	Start - End	Observed	Mr(expt)	Mr(calc)	ppm	M	Score	Expect	Rank	U	Peptide
4394	346 - 357	679.3597	1356.7048	1356.7038	0.78	0	39	0.0012	1	U	R.HVDYVVDQVVGK.L
516	358 - 364	414.7813	827.5480	827.5480	-0.062	1	28	0.012	1	U	K.LIEVVKK.K
2849	368 - 378	580.3449	1158.6752	1158.6761	-0.81	0	39	0.0017	1	U	K.AGVSVKPFQVK.N
2850	368 - 378	387.2325	1158.6756	1158.6761	-0.45	0	37	0.0046	1	U	K.AGVSVKPFQVK.N
2851	368 - 378	580.3451	1158.6757	1158.6761	-0.34	0	30	0.0089	1	U	K.AGVSVKPFQVK.N
2852	368 - 378	580.3452	1158.6758	1158.6761	-0.29	0	48	0.00038	1	U	K.AGVSVKPFQVK.N
2862	368 - 378	580.3464	1158.6782	1158.6761	1.80	0	39	0.0027	1	U	K.AGVSVKPFQVK.N
10737	379 - 399	859.4251	2575.2535	2575.2529	0.22	0	39	0.0041	1	U	K.NHIWVFINCLIENPTFDSQTK.E
397	440 - 446	401.7245	801.4344	801.4344		0	37	0.0092	1	U	K.AQTQLNK.K
1054	440 - 447	465.7720	929.5295	929.5294	0.11	1	27	0.025	1	U	K.AQTQLNKK.C
1341	462 - 471	488.2222	974.4297	974.4305	-0.77	0	54	0.00017	1	U	K.LDDANDAGGK.H
1343	462 - 471	488.2225	974.4305	974.4305	0.012	0	49	0.00054	1	U	K.LDDANDAGGK.H
7763	472 - 487	887.4382	1772.8619	1772.8614	0.27	0	93	1.5e-08	1	U	K.HSLECTLILTEGDSAK.S
2549	488 - 499	564.8392	1127.6638	1127.6663	-2.19	0	69	4.1e-06	1	U	K.SLAVSGLGVIGR.D
2550	488 - 499	564.8397	1127.6648	1127.6663	-1.25	0	50	4.7e-05	1	U	K.SLAVSGLGVIGR.D
2551	488 - 499	564.8401	1127.6656	1127.6663	-0.61	0	42	0.00032	1	U	K.SLAVSGLGVIGR.D
2552	488 - 499	564.8401	1127.6656	1127.6663	-0.60	0	48	0.00063	1	U	K.SLAVSGLGVIGR.D
2554	488 - 499	564.8402	1127.6658	1127.6663	-0.38	0	39	0.0016	1	U	K.SLAVSGLGVIGR.D
2555	488 - 499	564.8403	1127.6660	1127.6663	-0.26	0	35	0.004	1	U	K.SLAVSGLGVIGR.D
2556	488 - 499	564.8403	1127.6660	1127.6663	-0.19	0	67	3e-06	1	U	K.SLAVSGLGVIGR.D
2557	488 - 499	564.8404	1127.6662	1127.6663	-0.064	0	45	0.00021	1	U	K.SLAVSGLGVIGR.D
2558	488 - 499	564.8404	1127.6663	1127.6663	0.0071	0	38	0.00096	1	U	K.SLAVSGLGVIGR.D
2559	488 - 499	564.8406	1127.6665	1127.6663	0.26	0	49	6.9e-05	1	U	K.SLAVSGLGVIGR.D
2560	488 - 499	376.8962	1127.6669	1127.6663	0.53	0	36	0.0094	1	U	K.SLAVSGLGVIGR.D
2561	488 - 499	564.8407	1127.6669	1127.6663	0.56	0	48	5.1e-05	1	U	K.SLAVSGLGVIGR.D
2562	488 - 499	564.8408	1127.6670	1127.6663	0.65	0	52	5.7e-05	1	U	K.SLAVSGLGVIGR.D
2563	488 - 499	564.8408	1127.6671	1127.6663	0.79	0	74	6.5e-07	1	U	K.SLAVSGLGVIGR.D
2564	488 - 499	564.8409	1127.6673	1127.6663	0.91	0	53	9.6e-05	1	U	K.SLAVSGLGVIGR.D
2565	488 - 499	564.8413	1127.6681	1127.6663	1.62	0	48	7.9e-05	1	U	K.SLAVSGLGVIGR.D
2566	488 - 499	564.8416	1127.6686	1127.6663	2.12	0	61	6.2e-06	1	U	K.SLAVSGLGVIGR.D
623	502 - 508	426.2422	850.4699	850.4701	-0.25	0	29	0.021	1		R.YGVFPLR.G
624	502 - 508	426.2423	850.4701	850.4701	-0.018	0	28	0.028	1		R.YGVFPLR.G
5875	521 - 533	765.4037	1528.7929	1528.7919	0.68	0	96	1.8e-08	1		K.QIMENAEINNIIK.I
5877	521 - 533	765.4059	1528.7973	1528.7919	3.56	0	61	6.7e-05	1		K.QIMENAEINNIIK.I
6052	521 - 533	773.4001	1544.7857	1544.7868	-0.70	0	41	0.0012	1		K.QIMENAEINNIIK.I + Oxidation (M)
6053	521 - 533	773.4005	1544.7864	1544.7868	-0.26	0	62	3.4e-05	1		K.QIMENAEINNIIK.I + Oxidation (M)
6054	521 - 533	773.4005	1544.7864	1544.7868	-0.23	0	61	1.6e-05	1		K.QIMENAEINNIIK.I + Oxidation (M)
6055	521 - 533	773.4006	1544.7866	1544.7868	-0.10	0	75	1.7e-06	1		K.QIMENAEINNIIK.I + Oxidation (M)
6056	521 - 533	773.4007	1544.7868	1544.7868	-0.014	0	63	4.7e-05	1		K.QIMENAEINNIIK.I + Oxidation (M)
469	534 - 540	410.7501	819.4856	819.4854	0.17	0	23	0.015	1		K.IVGLQYK.K
473	534 - 540	410.7505	819.4864	819.4854	1.24	0	32	0.0051	1		K.IVGLQYK.K
2814	541 - 550	578.2802	1154.5458	1154.5455	0.28	1	34	0.0028	1	U	K.KSYDDAESLK.T
1714	542 - 550	514.2324	1026.4502	1026.4505	-0.33	0	46	9.1e-05	1	U	K.SYDDAESLK.T
1716	542 - 550	514.2327	1026.4509	1026.4505	0.31	0	22	0.015	1	U	K.SYDDAESLK.T
1717	542 - 550	514.2328	1026.4509	1026.4505	0.39	0	26	0.0081	1	U	K.SYDDAESLK.T
7465	557 - 571	866.4060	1730.7975	1730.7968	0.46	0	52	4.9e-05	1		K.IMIMTDQDQDGSHIK.G
7467	557 - 571	577.9400	1730.7980	1730.7968	0.73	0	21	0.043	1		K.IMIMTDQDQDGSHIK.G
7468	557 - 571	866.4064	1730.7983	1730.7968	0.92	0	69	6e-06	1		K.IMIMTDQDQDGSHIK.G
7565	557 - 571	583.2705	1746.7897	1746.7917	-1.13	0	32	0.0025	1		K.IMIMTDQDQDGSHIK.G + Oxidation (M)
7566	557 - 571	874.4028	1746.7910	1746.7917	-0.40	0	77	1.1e-06	1		K.IMIMTDQDQDGSHIK.G + Oxidation (M)
7567	557 - 571	874.4028	1746.7910	1746.7917	-0.38	0	68	1.6e-05	1		K.IMIMTDQDQDGSHIK.G + Oxidation (M)
7568	557 - 571	874.4028	1746.7910	1746.7917	-0.38	0	64	4.3e-05	1		K.IMIMTDQDQDGSHIK.G + Oxidation (M)
7571	557 - 571	583.2712	1746.7917	1746.7917	-0.011	0	38	0.0059	1		K.IMIMTDQDQDGSHIK.G + Oxidation (M)
7572	557 - 571	583.2712	1746.7919	1746.7917	0.14	0	26	0.012	1		K.IMIMTDQDQDGSHIK.G + Oxidation (M)
7573	557 - 571	583.2713	1746.7921	1746.7917	0.25	0	42	0.0066	1		K.IMIMTDQDQDGSHIK.G + Oxidation (M)
7574	557 - 571	583.2713	1746.7921	1746.7917	0.26	0	28	0.011	1		K.IMIMTDQDQDGSHIK.G + Oxidation (M)
7575	557 - 571	874.4034	1746.7921	1746.7917	0.27	0	67	2.3e-05	1		K.IMIMTDQDQDGSHIK.G + Oxidation (M)
7576	557 - 571	874.4034	1746.7922	1746.7917	0.28	0	71	1e-06	1		K.IMIMTDQDQDGSHIK.G + Oxidation (M)
7578	557 - 571	583.2714	1746.7924	1746.7917	0.44	0	48	0.00081	1		K.IMIMTDQDQDGSHIK.G + Oxidation (M)
7579	557 - 571	583.2717	1746.7934	1746.7917	0.98	0	45	0.0012	1		K.IMIMTDQDQDGSHIK.G + Oxidation (M)
7580	557 - 571	583.2721	1746.7944	1746.7917	1.59	0	29	0.013	1		K.IMIMTDQDQDGSHIK.G + Oxidation (M)
7678	557 - 571	882.3998	1762.7851	1762.7866	-0.84	0	70	1.3e-05	1		K.IMIMTDQDQDGSHIK.G + 2 Oxidation (M)
7679	557 - 571	588.6025	1762.7856	1762.7866	-0.54	0	54	0.00012	1		K.IMIMTDQDQDGSHIK.G + 2 Oxidation (M)
7681	557 - 571	588.6026	1762.7859	1762.7866	-0.37	0	30	0.012	1		K.IMIMTDQDQDGSHIK.G + 2 Oxidation (M)
7682	557 - 571	588.6026	1762.7860	1762.7866	-0.35	0	29	0.012	1		K.IMIMTDQDQDGSHIK.G + 2 Oxidation (M)
7683	557 - 571	588.6026	1762.7860	1762.7866	-0.35	0	54	0.00044	1		K.IMIMTDQDQDGSHIK.G + 2 Oxidation (M)
7685	557 - 571	882.4003	1762.7860	1762.7866	-0.31	0	66	1.2e-05	1		K.IMIMTDQDQDGSHIK.G + 2 Oxidation (M)

Query	Start - End	Observed	Mr(expt)	Mr(calc)	ppm	M	Score	Expect	Rank	U	Peptide
7686	557 - 571	588.6026	1762.7861	1762.7866	-0.28	0	39	0.0025	1		K.IMIMTDQDQDGSHIK.G + 2 Oxidation (M)
7687	557 - 571	882.4004	1762.7861	1762.7866	-0.25	0	56	0.0003	1		K.IMIMTDQDQDGSHIK.G + 2 Oxidation (M)
7688	557 - 571	588.6027	1762.7864	1762.7866	-0.13	0	62	7.5e-05	1		K.IMIMTDQDQDGSHIK.G + 2 Oxidation (M)
7689	557 - 571	588.6028	1762.7867	1762.7866	0.040	0	41	0.0023	1		K.IMIMTDQDQDGSHIK.G + 2 Oxidation (M)
7690	557 - 571	882.4007	1762.7869	1762.7866	0.19	0	55	0.00014	1		K.IMIMTDQDQDGSHIK.G + 2 Oxidation (M)
7691	557 - 571	588.6030	1762.7871	1762.7866	0.26	0	48	0.00025	1		K.IMIMTDQDQDGSHIK.G + 2 Oxidation (M)
7693	557 - 571	588.6031	1762.7874	1762.7866	0.45	0	37	0.0049	1		K.IMIMTDQDQDGSHIK.G + 2 Oxidation (M)
7694	557 - 571	882.4010	1762.7874	1762.7866	0.47	0	64	4.7e-05	1		K.IMIMTDQDQDGSHIK.G + 2 Oxidation (M)
7695	557 - 571	882.4010	1762.7875	1762.7866	0.54	0	40	0.00084	1		K.IMIMTDQDQDGSHIK.G + 2 Oxidation (M)
7696	557 - 571	882.4011	1762.7876	1762.7866	0.60	0	72	7.8e-06	1		K.IMIMTDQDQDGSHIK.G + 2 Oxidation (M)
7697	557 - 571	588.6032	1762.7878	1762.7866	0.69	0	41	0.002	1		K.IMIMTDQDQDGSHIK.G + 2 Oxidation (M)
7698	557 - 571	882.4018	1762.7891	1762.7866	1.43	0	47	0.0016	1		K.IMIMTDQDQDGSHIK.G + 2 Oxidation (M)
7699	557 - 571	882.4019	1762.7893	1762.7866	1.53	0	47	0.00071	1		K.IMIMTDQDQDGSHIK.G + 2 Oxidation (M)
8612	572 - 587	951.5405	1901.0665	1901.0676	-0.57	0	47	4e-05	1	U	K.GLLINFIHHNWPSLLK.H
8613	572 - 587	634.6963	1901.0671	1901.0676	-0.25	0	24	0.018	1	U	K.GLLINFIHHNWPSLLK.H
8615	572 - 587	634.6965	1901.0678	1901.0676	0.13	0	31	0.0067	1	U	K.GLLINFIHHNWPSLLK.H
8618	572 - 587	951.5415	1901.0685	1901.0676	0.51	0	57	5.2e-06	1	U	K.GLLINFIHHNWPSLLK.H
5878	588 - 600	510.6159	1528.8259	1528.8290	-2.02	0	40	0.0028	1	U	K.HGFLEEFITPIVK.A
5879	588 - 600	765.4203	1528.8261	1528.8290	-1.87	0	83	1.3e-07	1	U	K.HGFLEEFITPIVK.A
5880	588 - 600	765.4213	1528.8280	1528.8290	-0.60	0	63	5.8e-06	1	U	K.HGFLEEFITPIVK.A
5881	588 - 600	510.6166	1528.8281	1528.8290	-0.55	0	34	0.0026	1	U	K.HGFLEEFITPIVK.A
5882	588 - 600	510.6166	1528.8281	1528.8290	-0.55	0	33	0.0094	1	U	K.HGFLEEFITPIVK.A
5883	588 - 600	765.4215	1528.8284	1528.8290	-0.36	0	62	1.5e-06	1	U	K.HGFLEEFITPIVK.A
5884	588 - 600	510.6168	1528.8284	1528.8290	-0.35	0	36	0.0024	1	U	K.HGFLEEFITPIVK.A
5885	588 - 600	765.4215	1528.8285	1528.8290	-0.32	0	87	1.6e-08	1	U	K.HGFLEEFITPIVK.A
5887	588 - 600	510.6168	1528.8286	1528.8290	-0.24	0	34	0.0064	1	U	K.HGFLEEFITPIVK.A
5888	588 - 600	765.4216	1528.8286	1528.8290	-0.21	0	61	6.9e-06	1	U	K.HGFLEEFITPIVK.A
5889	588 - 600	765.4216	1528.8287	1528.8290	-0.15	0	69	5.5e-07	1	U	K.HGFLEEFITPIVK.A
5891	588 - 600	765.4218	1528.8290	1528.8290	0.042	0	65	2.3e-06	1	U	K.HGFLEEFITPIVK.A
5892	588 - 600	765.4218	1528.8291	1528.8290	0.11	0	66	1.7e-06	1	U	K.HGFLEEFITPIVK.A
5893	588 - 600	510.6170	1528.8291	1528.8290	0.12	0	42	0.0011	1	U	K.HGFLEEFITPIVK.A
5894	588 - 600	510.6170	1528.8292	1528.8290	0.14	0	48	0.00056	1	U	K.HGFLEEFITPIVK.A
5895	588 - 600	510.6170	1528.8292	1528.8290	0.18	0	29	0.011	1	U	K.HGFLEEFITPIVK.A
5896	588 - 600	510.6170	1528.8293	1528.8290	0.21	0	19	0.026	1	U	K.HGFLEEFITPIVK.A
5897	588 - 600	765.4219	1528.8293	1528.8290	0.23	0	66	7.7e-06	1	U	K.HGFLEEFITPIVK.A
5899	588 - 600	765.4219	1528.8293	1528.8290	0.24	0	45	6.1e-05	1	U	K.HGFLEEFITPIVK.A
5900	588 - 600	765.4220	1528.8294	1528.8290	0.26	0	66	1.1e-06	1	U	K.HGFLEEFITPIVK.A
5901	588 - 600	510.6171	1528.8295	1528.8290	0.33	0	49	0.00046	1	U	K.HGFLEEFITPIVK.A
5902	588 - 600	765.4220	1528.8295	1528.8290	0.34	0	84	2.5e-08	1	U	K.HGFLEEFITPIVK.A
5903	588 - 600	765.4221	1528.8296	1528.8290	0.41	0	39	0.00024	1	U	K.HGFLEEFITPIVK.A
5904	588 - 600	765.4221	1528.8296	1528.8290	0.43	0	70	5.4e-07	1	U	K.HGFLEEFITPIVK.A
5905	588 - 600	510.6172	1528.8297	1528.8290	0.45	0	30	0.0067	1	U	K.HGFLEEFITPIVK.A
5906	588 - 600	765.4222	1528.8298	1528.8290	0.58	0	63	2.8e-06	1	U	K.HGFLEEFITPIVK.A
5907	588 - 600	510.6173	1528.8300	1528.8290	0.69	0	34	0.0052	1	U	K.HGFLEEFITPIVK.A
5908	588 - 600	765.4223	1528.8300	1528.8290	0.71	0	49	2.3e-05	1	U	K.HGFLEEFITPIVK.A
9596	604 - 620	1080.5176	2159.0205	2159.0211	-0.26	1	105	2.1e-09	1	U	K.NKQELSFYSIPEFDEWK.K
9597	604 - 620	720.6815	2159.0227	2159.0211	0.73	1	31	0.039	1	U	K.NKQELSFYSIPEFDEWK.K
9598	604 - 620	1080.5196	2159.0247	2159.0211	1.66	1	96	1.6e-08	1	U	K.NKQELSFYSIPEFDEWK.K
10031	604 - 621	763.3792	2287.1158	2287.1161	-0.099	2	46	0.00023	1	U	K.NKQELSFYSIPEFDEWKK.H
8721	606 - 620	959.4480	1916.8815	1916.8832	-0.87	0	28	0.0064	1	U	K.QELSFYSIPEFDEWK.K
8722	606 - 620	959.4483	1916.8821	1916.8832	-0.57	0	43	0.0014	1	U	K.QELSFYSIPEFDEWK.K
8723	606 - 620	959.4488	1916.8831	1916.8832	-0.050	0	44	0.00059	1	U	K.QELSFYSIPEFDEWK.K
9179	606 - 621	682.6664	2044.9773	2044.9782	-0.44	1	41	0.00043	1	U	K.QELSFYSIPEFDEWKK.H
9180	606 - 621	1023.4962	2044.9778	2044.9782	-0.17	1	46	0.00015	1	U	K.QELSFYSIPEFDEWKK.H
1958	647 - 654	530.7224	1059.4303	1059.4331	-2.64	0	31	0.0043	1	U	K.EYFADMER.H
1959	647 - 654	530.7237	1059.4328	1059.4331	-0.33	0	21	0.038	1	U	K.EYFADMER.H
1960	647 - 654	530.7239	1059.4332	1059.4331	0.044	0	32	0.0022	1	U	K.EYFADMER.H
1961	647 - 654	530.7239	1059.4332	1059.4331	0.10	0	18	0.037	1	U	K.EYFADMER.H
2097	647 - 654	538.7211	1075.4277	1075.4280	-0.29	0	24	0.026	1	U	K.EYFADMER.H + Oxidation (M)
2098	647 - 654	538.7212	1075.4278	1075.4280	-0.20	0	30	0.0067	1	U	K.EYFADMER.H + Oxidation (M)
2101	647 - 654	538.7215	1075.4284	1075.4280	0.32	0	27	0.017	1	U	K.EYFADMER.H + Oxidation (M)
6967	661 - 676	834.9084	1667.8023	1667.8042	-1.19	0	90	4.2e-09	1	U	R.YAGPEDDAAITLAFSK.K
6968	661 - 676	834.9084	1667.8023	1667.8042	-1.15	0	79	6.6e-08	1	U	R.YAGPEDDAAITLAFSK.K
6969	661 - 676	834.9087	1667.8028	1667.8042	-0.89	0	76	1e-07	1	U	R.YAGPEDDAAITLAFSK.K
6970	661 - 676	834.9087	1667.8028	1667.8042	-0.89	0	45	6e-05	1	U	R.YAGPEDDAAITLAFSK.K
6971	661 - 676	834.9090	1667.8033	1667.8042	-0.54	0	50	5.5e-05	1	U	R.YAGPEDDAAITLAFSK.K
6972	661 - 676	834.9090	1667.8035	1667.8042	-0.44	0	90	1.9e-08	1	U	R.YAGPEDDAAITLAFSK.K
6973	661 - 676	834.9091	1667.8036	1667.8042	-0.37	0	78	1.7e-07	1	U	R.YAGPEDDAAITLAFSK.K
6974	661 - 676	834.9092	1667.8037	1667.8042	-0.30	0	96	3.8e-09	1	U	R.YAGPEDDAAITLAFSK.K
6976	661 - 676	834.9092	1667.8039	1667.8042	-0.21	0	82	1.3e-07	1	U	R.YAGPEDDAAITLAFSK.K
6977	661 - 676	834.9093	1667.8040	1667.8042	-0.15	0	46	4.5e-05	1	U	R.YAGPEDDAAITLAFSK.K
6978	661 - 676	834.9093	1667.8040	1667.8042	-0.14	0	69	6.6e-07	1	U	R.YAGPEDDAAITLAFSK.K
6979	661 - 676	834.9093	1667.8040	1667.8042	-0.12	0	56	6e-06	1	U	R.YAGPEDDAAITLAFSK.K
6980	661 - 676	834.9094	1667.8042	1667.8042	-0.046	0	49	4.7e-05	1	U	R.YAGPEDDAAITLAFSK.K

Query	Start - End	Observed	Mr(expt)	Mr(calc)	ppm	M	Score	Expect	Rank	U	Peptide
6981	661 - 676	834.9094	1667.8042	1667.8042	-0.046	0	87	6.8e-09	1	U	R.YAGPEDDAAITLAFSK.K
6982	661 - 676	834.9094	1667.8042	1667.8042	-0.010	0	77	3.2e-07	1	U	R.YAGPEDDAAITLAFSK.K
6983	661 - 676	834.9096	1667.8046	1667.8042	0.23	0	85	2.8e-08	1	U	R.YAGPEDDAAITLAFSK.K
6984	661 - 676	834.9096	1667.8046	1667.8042	0.24	0	86	2e-08	1	U	R.YAGPEDDAAITLAFSK.K
6985	661 - 676	834.9096	1667.8047	1667.8042	0.27	0	78	1.4e-07	1	U	R.YAGPEDDAAITLAFSK.K
6986	661 - 676	834.9097	1667.8048	1667.8042	0.33	0	77	4.5e-07	1	U	R.YAGPEDDAAITLAFSK.K
6987	661 - 676	834.9098	1667.8050	1667.8042	0.46	0	72	5e-07	1	U	R.YAGPEDDAAITLAFSK.K
6988	661 - 676	556.9423	1667.8050	1667.8042	0.46	0	51	9e-05	1	U	R.YAGPEDDAAITLAFSK.K
6989	661 - 676	834.9102	1667.8057	1667.8042	0.90	0	62	1.9e-06	1	U	R.YAGPEDDAAITLAFSK.K
6990	661 - 676	834.9103	1667.8060	1667.8042	1.07	0	20	0.02	1	U	R.YAGPEDDAAITLAFSK.K
6991	661 - 676	834.9111	1667.8076	1667.8042	1.99	0	79	4e-07	1	U	R.YAGPEDDAAITLAFSK.K
6992	661 - 676	834.9112	1667.8078	1667.8042	2.12	0	37	0.00076	1	U	R.YAGPEDDAAITLAFSK.K
5237	683 - 693	490.2344	1467.6815	1467.6816	-0.11	1	27	0.047	1		R.KEWLTNFMEDR.R
5364	683 - 693	742.8450	1483.6754	1483.6765	-0.77	1	18	0.043	1		R.KEWLTNFMEDR.R + Oxidation (M)
5365	683 - 693	495.5660	1483.6762	1483.6765	-0.23	1	24	0.018	1		R.KEWLTNFMEDR.R + Oxidation (M)
5366	683 - 693	495.5660	1483.6763	1483.6765	-0.15	1	19	0.047	1		R.KEWLTNFMEDR.R + Oxidation (M)
5367	683 - 693	742.8455	1483.6764	1483.6765	-0.11	1	50	4e-05	1		R.KEWLTNFMEDR.R + Oxidation (M)
5368	683 - 693	495.5661	1483.6765	1483.6765	-0.049	1	27	0.016	1		R.KEWLTNFMEDR.R + Oxidation (M)
5369	683 - 693	495.5662	1483.6768	1483.6765	0.15	1	28	0.024	1		R.KEWLTNFMEDR.R + Oxidation (M)
5371	683 - 693	742.8464	1483.6782	1483.6765	1.09	1	20	0.031	1		R.KEWLTNFMEDR.R + Oxidation (M)
5373	683 - 693	742.8470	1483.6794	1483.6765	1.92	1	23	0.045	1		R.KEWLTNFMEDR.R + Oxidation (M)
4279	684 - 693	670.8006	1339.5866	1339.5867	-0.031	0	43	0.004	1		K.EWLTNFMEDR.R
4280	684 - 693	670.8006	1339.5866	1339.5867	-0.031	0	24	0.01	1		K.EWLTNFMEDR.R
4281	684 - 693	670.8007	1339.5869	1339.5867	0.16	0	37	0.0034	1		K.EWLTNFMEDR.R
4367	684 - 693	678.7982	1355.5819	1355.5816	0.22	0	24	0.049	1		K.EWLTNFMEDR.R + Oxidation (M)
4368	684 - 693	678.7984	1355.5822	1355.5816	0.46	0	25	0.034	1		K.EWLTNFMEDR.R + Oxidation (M)
8221	697 - 712	610.9994	1829.9764	1829.9788	-1.33	1	51	7e-05	1	U	R.RLHGLPEQFLYGTATK.H
8222	697 - 712	610.9996	1829.9770	1829.9788	-0.99	1	37	0.0016	1	U	R.RLHGLPEQFLYGTATK.H
8226	697 - 712	611.0001	1829.9784	1829.9788	-0.20	1	42	0.0038	1	U	R.RLHGLPEQFLYGTATK.H
8227	697 - 712	915.9966	1829.9785	1829.9788	-0.14	1	79	5.6e-08	1	U	R.RLHGLPEQFLYGTATK.H
8228	697 - 712	611.0002	1829.9786	1829.9788	-0.10	1	52	0.00015	1	U	R.RLHGLPEQFLYGTATK.H
8230	697 - 712	611.0002	1829.9788	1829.9788	-0.019	1	70	6.2e-06	1	U	R.RLHGLPEQFLYGTATK.H
8231	697 - 712	611.0004	1829.9794	1829.9788	0.31	1	35	0.0035	1	U	R.RLHGLPEQFLYGTATK.H
8232	697 - 712	611.0004	1829.9795	1829.9788	0.36	1	28	0.0081	1	U	R.RLHGLPEQFLYGTATK.H
7026	698 - 712	837.9444	1673.8743	1673.8777	-2.04	0	26	0.0083	1	U	R.LHGLPEQFLYGTATK.H
7027	698 - 712	837.9450	1673.8753	1673.8777	-1.41	0	20	0.017	1	U	R.LHGLPEQFLYGTATK.H
7028	698 - 712	837.9452	1673.8759	1673.8777	-1.06	0	20	0.023	1	U	R.LHGLPEQFLYGTATK.H
7030	698 - 712	837.9455	1673.8764	1673.8777	-0.80	0	41	0.00066	1	U	R.LHGLPEQFLYGTATK.H
7032	698 - 712	837.9457	1673.8768	1673.8777	-0.55	0	35	0.00058	1	U	R.LHGLPEQFLYGTATK.H
7033	698 - 712	558.9662	1673.8769	1673.8777	-0.48	0	35	0.0013	1	U	R.LHGLPEQFLYGTATK.H
7034	698 - 712	837.9457	1673.8769	1673.8777	-0.47	0	19	0.018	1	U	R.LHGLPEQFLYGTATK.H
7035	698 - 712	558.9663	1673.8770	1673.8777	-0.45	0	29	0.0068	1	U	R.LHGLPEQFLYGTATK.H
7036	698 - 712	558.9663	1673.8771	1673.8777	-0.36	0	42	0.0005	1	U	R.LHGLPEQFLYGTATK.H
7039	698 - 712	837.9459	1673.8772	1673.8777	-0.28	0	24	0.0054	1	U	R.LHGLPEQFLYGTATK.H
7040	698 - 712	558.9664	1673.8774	1673.8777	-0.20	0	31	0.0064	1	U	R.LHGLPEQFLYGTATK.H
7042	698 - 712	558.9665	1673.8776	1673.8777	-0.089	0	48	0.0001	1	U	R.LHGLPEQFLYGTATK.H
7043	698 - 712	558.9665	1673.8776	1673.8777	-0.089	0	52	3.6e-05	1	U	R.LHGLPEQFLYGTATK.H
7044	698 - 712	558.9665	1673.8776	1673.8777	-0.071	0	30	0.0042	1	U	R.LHGLPEQFLYGTATK.H
7045	698 - 712	558.9665	1673.8776	1673.8777	-0.071	0	29	0.012	1	U	R.LHGLPEQFLYGTATK.H
7046	698 - 712	837.9461	1673.8776	1673.8777	-0.044	0	56	9.9e-06	1	U	R.LHGLPEQFLYGTATK.H
7047	698 - 712	558.9666	1673.8779	1673.8777	0.11	0	30	0.016	1	U	R.LHGLPEQFLYGTATK.H
7048	698 - 712	837.9462	1673.8779	1673.8777	0.11	0	20	0.015	1	U	R.LHGLPEQFLYGTATK.H
7050	698 - 712	837.9463	1673.8780	1673.8777	0.20	0	30	0.0017	1	U	R.LHGLPEQFLYGTATK.H
7051	698 - 712	558.9666	1673.8780	1673.8777	0.20	0	24	0.029	1	U	R.LHGLPEQFLYGTATK.H
7052	698 - 712	558.9666	1673.8781	1673.8777	0.22	0	36	0.019	1	U	R.LHGLPEQFLYGTATK.H
7054	698 - 712	558.9667	1673.8782	1673.8777	0.27	0	34	0.0051	1	U	R.LHGLPEQFLYGTATK.H
7055	698 - 712	837.9464	1673.8782	1673.8777	0.31	0	41	0.00015	1	U	R.LHGLPEQFLYGTATK.H
7056	698 - 712	558.9668	1673.8784	1673.8777	0.43	0	39	0.0036	1	U	R.LHGLPEQFLYGTATK.H
7058	698 - 712	837.9466	1673.8785	1673.8777	0.51	0	33	0.00081	1	U	R.LHGLPEQFLYGTATK.H
7060	698 - 712	837.9467	1673.8788	1673.8777	0.65	0	30	0.0029	1	U	R.LHGLPEQFLYGTATK.H
7061	698 - 712	837.9467	1673.8788	1673.8777	0.69	0	51	2.9e-05	1	U	R.LHGLPEQFLYGTATK.H
7063	698 - 712	558.9671	1673.8795	1673.8777	1.08	0	40	0.0076	1	U	R.LHGLPEQFLYGTATK.H
7064	698 - 712	558.9672	1673.8798	1673.8777	1.24	0	50	0.00017	1	U	R.LHGLPEQFLYGTATK.H
7065	698 - 712	558.9672	1673.8798	1673.8777	1.24	0	25	0.026	1	U	R.LHGLPEQFLYGTATK.H
7066	698 - 712	558.9673	1673.8801	1673.8777	1.45	0	22	0.023	1	U	R.LHGLPEQFLYGTATK.H
3638	713 - 722	632.8187	1263.6228	1263.6248	-1.54	0	18	0.019	1	U	K.HLTYNDFINK.E
3639	713 - 722	632.8188	1263.6231	1263.6248	-1.28	0	24	0.045	1	U	K.HLTYNDFINK.E
3643	713 - 722	632.8192	1263.6239	1263.6248	-0.67	0	38	0.0015	1	U	K.HLTYNDFINK.E
3646	713 - 722	632.8194	1263.6243	1263.6248	-0.37	0	30	0.0024	1	U	K.HLTYNDFINK.E
3648	713 - 722	632.8195	1263.6244	1263.6248	-0.26	0	40	0.0011	1	U	K.HLTYNDFINK.E
3650	713 - 722	632.8196	1263.6246	1263.6248	-0.16	0	37	0.011	1	U	K.HLTYNDFINK.E
3651	713 - 722	632.8196	1263.6246	1263.6248	-0.097	0	32	0.0033	1	U	K.HLTYNDFINK.E
3653	713 - 722	632.8196	1263.6247	1263.6248	-0.082	0	28	0.021	1	U	K.HLTYNDFINK.E
3654	713 - 722	422.2155	1263.6247	1263.6248	-0.078	0	30	0.014	1	U	K.HLTYNDFINK.E
3655	713 - 722	632.8196	1263.6247	1263.6248	-0.066	0	24	0.022	1	U	K.HLTYNDFINK.E
3656	713 - 722	632.8196	1263.6247	1263.6248	-0.018	0	39	0.00035	1	U	K.HLTYNDFINK.E
3658	713 - 722	632.8197	1263.6248	1263.6248	0.029	0	36	0.00068	1	U	K.HLTYNDFINK.E
3659	713 - 722	632.8197	1263.6249	1263.6248	0.077	0	36	0.0013	1	U	K.HLTYNDFINK.E
3660	713 - 722	632.8197	1263.6249	1263.6248	0.077	0	33	0.0019	1	U	K.HLTYNDFINK.E
3661	713 - 722	632.8197	1263.6249	1263.6248	0.093	0	39	0.00098	1	U	K.HLTYNDFINK.E

Query	Start - End	Observed	Mr(expt)	Mr(calc)	ppm	M	Score	Expect	Rank	U	Peptide
3662	713 - 722	632.8198	1263.6250	1263.6248	0.16	0	22	0.0089	1	U	K.HLTYNDFINK.E
3665	713 - 722	632.8198	1263.6250	1263.6248	0.22	0	23	0.022	1	U	K.HLTYNDFINK.E
3666	713 - 722	632.8198	1263.6251	1263.6248	0.28	0	41	0.0004	1	U	K.HLTYNDFINK.E
3667	713 - 722	632.8199	1263.6252	1263.6248	0.31	0	17	0.025	1	U	K.HLTYNDFINK.E
3668	713 - 722	632.8200	1263.6255	1263.6248	0.57	0	46	0.00017	1	U	K.HLTYNDFINK.E
4922	723 - 734	718.8527	1435.6908	1435.6943	-2.44	0	33	0.0048	1		K.ELILFSNSDNER.S
4923	723 - 734	718.8532	1435.6917	1435.6943	-1.77	0	43	0.0043	1		K.ELILFSNSDNER.S
4924	723 - 734	718.8535	1435.6924	1435.6943	-1.30	0	21	0.019	1		K.ELILFSNSDNER.S
4925	723 - 734	718.8535	1435.6925	1435.6943	-1.26	0	31	0.017	1		K.ELILFSNSDNER.S
4926	723 - 734	718.8542	1435.6938	1435.6943	-0.34	0	29	0.022	1		K.ELILFSNSDNER.S
4927	723 - 734	718.8542	1435.6939	1435.6943	-0.28	0	51	9.7e-05	1		K.ELILFSNSDNER.S
4928	723 - 734	718.8542	1435.6939	1435.6943	-0.24	0	20	0.046	1		K.ELILFSNSDNER.S
4929	723 - 734	718.8543	1435.6940	1435.6943	-0.20	0	29	0.0049	1		K.ELILFSNSDNER.S
4932	723 - 734	718.8544	1435.6942	1435.6943	-0.071	0	29	0.033	1		K.ELILFSNSDNER.S
4934	723 - 734	718.8544	1435.6942	1435.6943	-0.029	0	42	0.0024	1		K.ELILFSNSDNER.S
4935	723 - 734	718.8544	1435.6943	1435.6943	-0.015	0	38	0.0047	1		K.ELILFSNSDNER.S
4939	723 - 734	718.8546	1435.6946	1435.6943	0.19	0	26	0.032	1		K.ELILFSNSDNER.S
4942	723 - 734	718.8549	1435.6952	1435.6943	0.65	0	31	0.0045	1		K.ELILFSNSDNER.S
4943	723 - 734	718.8550	1435.6954	1435.6943	0.75	0	46	0.0016	1		K.ELILFSNSDNER.S
4944	723 - 734	718.8550	1435.6954	1435.6943	0.78	0	42	0.0049	1		K.ELILFSNSDNER.S
4947	723 - 734	718.8550	1435.6955	1435.6943	0.86	0	22	0.022	1		K.ELILFSNSDNER.S
4948	723 - 734	718.8551	1435.6957	1435.6943	1.00	0	39	0.0013	1		K.ELILFSNSDNER.S
4949	723 - 734	718.8553	1435.6960	1435.6943	1.17	0	33	0.02	1		K.ELILFSNSDNER.S
4950	723 - 734	718.8555	1435.6964	1435.6943	1.45	0	39	0.0022	1		K.ELILFSNSDNER.S
5520	735 - 748	750.9105	1499.8064	1499.8096	-2.15	0	31	0.0064	1	U	R.SIPSLVDGFKPGQR.K
5525	735 - 748	750.9119	1499.8093	1499.8096	-0.20	0	44	0.00068	1	U	R.SIPSLVDGFKPGQR.K
5527	735 - 748	750.9122	1499.8097	1499.8096	0.078	0	53	9.9e-05	1	U	R.SIPSLVDGFKPGQR.K
5536	735 - 748	750.9126	1499.8105	1499.8096	0.61	0	22	0.012	1	U	R.SIPSLVDGFKPGQR.K
5538	735 - 748	750.9126	1499.8107	1499.8096	0.70	0	43	0.00069	1	U	R.SIPSLVDGFKPGQR.K
5544	735 - 748	750.9130	1499.8114	1499.8096	1.21	0	15	0.041	1	U	R.SIPSLVDGFKPGQR.K
4072	826 - 836	658.3573	1314.7000	1314.7006	-0.43	0	45	0.00053	1	U	R.YIFTMLSTLAR.L
4073	826 - 836	658.3574	1314.7002	1314.7006	-0.30	0	46	0.00012	1	U	R.YIFTMLSTLAR.L
4074	826 - 836	658.3574	1314.7002	1314.7006	-0.28	0	47	0.00012	1	U	R.YIFTMLSTLAR.L
4075	826 - 836	658.3575	1314.7004	1314.7006	-0.16	0	48	0.00014	1	U	R.YIFTMLSTLAR.L
4076	826 - 836	658.3575	1314.7004	1314.7006	-0.098	0	43	0.00089	1	U	R.YIFTMLSTLAR.L
4077	826 - 836	658.3576	1314.7006	1314.7006	0.024	0	35	0.0037	1	U	R.YIFTMLSTLAR.L
4078	826 - 836	658.3576	1314.7006	1314.7006	0.024	0	36	0.0014	1	U	R.YIFTMLSTLAR.L
4079	826 - 836	658.3576	1314.7006	1314.7006	0.024	0	31	0.002	1	U	R.YIFTMLSTLAR.L
4080	826 - 836	658.3577	1314.7009	1314.7006	0.22	0	47	0.00012	1	U	R.YIFTMLSTLAR.L
4081	826 - 836	658.3578	1314.7010	1314.7006	0.30	0	37	0.0021	1	U	R.YIFTMLSTLAR.L
4082	826 - 836	658.3578	1314.7010	1314.7006	0.30	0	45	0.00027	1	U	R.YIFTMLSTLAR.L
4083	826 - 836	658.3579	1314.7012	1314.7006	0.45	0	36	0.0012	1	U	R.YIFTMLSTLAR.L
4084	826 - 836	658.3579	1314.7012	1314.7006	0.45	0	39	0.00078	1	U	R.YIFTMLSTLAR.L
4085	826 - 836	658.3580	1314.7015	1314.7006	0.68	0	45	9.5e-05	1	U	R.YIFTMLSTLAR.L
4206	826 - 836	666.3536	1330.6927	1330.6955	-2.13	0	31	0.005	1	U	R.YIFTMLSTLAR.L<br>+ Oxidation (M)
4207	826 - 836	666.3541	1330.6936	1330.6955	-1.45	0	37	0.0021	1	U	R.YIFTMLSTLAR.L<br>+ Oxidation (M)
4208	826 - 836	666.3546	1330.6947	1330.6955	-0.61	0	23	0.023	1	U	R.YIFTMLSTLAR.L<br>+ Oxidation (M)
4209	826 - 836	666.3549	1330.6953	1330.6955	-0.14	0	33	0.0017	1	U	R.YIFTMLSTLAR.L<br>+ Oxidation (M)
4213	826 - 836	666.3552	1330.6958	1330.6955	0.20	0	53	5.2e-05	1	U	R.YIFTMLSTLAR.L<br>+ Oxidation (M)
4214	826 - 836	666.3552	1330.6958	1330.6955	0.23	0	50	6.2e-05	1	U	R.YIFTMLSTLAR.L<br>+ Oxidation (M)
4215	826 - 836	666.3553	1330.6961	1330.6955	0.43	0	25	0.015	1	U	R.YIFTMLSTLAR.L<br>+ Oxidation (M)
4217	826 - 836	666.3553	1330.6961	1330.6955	0.47	0	33	0.0016	1	U	R.YIFTMLSTLAR.L<br>+ Oxidation (M)
4218	826 - 836	666.3554	1330.6962	1330.6955	0.52	0	24	0.031	1	U	R.YIFTMLSTLAR.L<br>+ Oxidation (M)
4219	826 - 836	666.3555	1330.6964	1330.6955	0.72	0	54	3.1e-05	1	U	R.YIFTMLSTLAR.L<br>+ Oxidation (M)
4220	826 - 836	666.3555	1330.6965	1330.6955	0.73	0	38	0.00055	1	U	R.YIFTMLSTLAR.L<br>+ Oxidation (M)
4223	826 - 836	666.3562	1330.6979	1330.6955	1.78	0	21	0.029	1	U	R.YIFTMLSTLAR.L<br>+ Oxidation (M)
4395	837 - 848	679.3897	1356.7648	1356.7653	-0.37	0	29	0.014	1	U	R.LLFPAVDDNLLK.F
4396	837 - 848	679.3898	1356.7650	1356.7653	-0.21	0	40	0.0038	1	U	R.LLFPAVDDNLLK.F
4397	837 - 848	679.3898	1356.7650	1356.7653	-0.18	0	31	0.011	1	U	R.LLFPAVDDNLLK.F
4398	837 - 848	679.3898	1356.7651	1356.7653	-0.16	0	36	0.0023	1	U	R.LLFPAVDDNLLK.F
4399	837 - 848	679.3898	1356.7651	1356.7653	-0.13	0	41	0.0013	1	U	R.LLFPAVDDNLLK.F
4400	837 - 848	679.3899	1356.7652	1356.7653	-0.089	0	43	0.00071	1	U	R.LLFPAVDDNLLK.F
4401	837 - 848	679.3899	1356.7653	1356.7653	0.014	0	37	0.0015	1	U	R.LLFPAVDDNLLK.F
4402	837 - 848	679.3900	1356.7654	1356.7653	0.073	0	40	0.0011	1	U	R.LLFPAVDDNLLK.F
4403	837 - 848	679.3900	1356.7654	1356.7653	0.10	0	52	0.00016	1	U	R.LLFPAVDDNLLK.F
4404	837 - 848	679.3900	1356.7654	1356.7653	0.10	0	29	0.0054	1	U	R.LLFPAVDDNLLK.F
4405	837 - 848	679.3900	1356.7655	1356.7653	0.18	0	69	3.8e-06	1	U	R.LLFPAVDDNLLK.F
4406	837 - 848	679.3901	1356.7656	1356.7653	0.24	0	35	0.0016	1	U	R.LLFPAVDDNLLK.F
4407	837 - 848	679.3902	1356.7658	1356.7653	0.40	0	23	0.021	1	U	R.LLFPAVDDNLLK.F
4408	837 - 848	679.3902	1356.7659	1356.7653	0.43	0	34	0.0097	1	U	R.LLFPAVDDNLLK.F
4411	837 - 848	679.3907	1356.7669	1356.7653	1.16	0	34	0.00066	1	U	R.LLFPAVDDNLLK.F
2053	849 - 856	535.7490	1069.4835	1069.4829	0.60	0	37	0.011	1		K.FLYDDNQR.V
609	885 - 891	424.7161	847.4176	847.4188	-1.40	0	27	0.024	1	U	K.LPNYDAR.E
612	885 - 891	424.7168	847.4191	847.4188	0.40	0	38	0.007	1	U	K.LPNYDAR.E
594	892 - 898	422.2379	842.4613	842.4610	0.34	0	34	0.016	1	U	R.EIVNNVR.R
7531	900 - 914	870.9240	1739.8334	1739.8375	-2.32	0	40	0.0015	1	U	R.MLDGLDPHPMLPNYK.N
7540	900 - 914	580.9535	1739.8386	1739.8375	0.62	0	40	0.0022	1	U	R.MLDGLDPHPMLPNYK.N
7541	900 - 914	870.9268	1739.8389	1739.8375	0.84	0	68	2.9e-06	1	U	R.MLDGLDPHPMLPNYK.N

Query	Start - End	Observed	Mr(expt)	Mr(calc)	ppm	M	Score	Expect	Rank	U	Peptide
7645	900 - 914	878.9234	1755.8322	1755.8324	-0.11	0	61	1.5e-05	1	U	R.MLDGLDPHPMLPNYK.N + Oxidation (M)
7648	900 - 914	878.9235	1755.8325	1755.8324	0.040	0	28	0.0084	1	U	R.MLDGLDPHPMLPNYK.N + Oxidation (M)
7649	900 - 914	878.9236	1755.8327	1755.8324	0.19	0	26	0.018	1	U	R.MLDGLDPHPMLPNYK.N + Oxidation (M)
7650	900 - 914	878.9238	1755.8330	1755.8324	0.33	0	27	0.018	1	U	R.MLDGLDPHPMLPNYK.N + Oxidation (M)
7749	900 - 914	886.9201	1771.8257	1771.8273	-0.90	0	32	0.0019	1	U	R.MLDGLDPHPMLPNYK.N + 2 Oxidation (M)
7750	900 - 914	886.9203	1771.8261	1771.8273	-0.70	0	23	0.02	1	U	R.MLDGLDPHPMLPNYK.N + 2 Oxidation (M)
7755	900 - 914	591.6165	1771.8278	1771.8273	0.27	0	35	0.023	1	U	R.MLDGLDPHPMLPNYK.N + 2 Oxidation (M)
7757	900 - 914	886.9215	1771.8285	1771.8273	0.66	0	32	0.017	1	U	R.MLDGLDPHPMLPNYK.N + 2 Oxidation (M)
7759	900 - 914	886.9229	1771.8312	1771.8273	2.20	0	34	0.0018	1	U	R.MLDGLDPHPMLPNYK.N + 2 Oxidation (M)
10319	918 - 939	808.4090	2422.2052	2422.2129	-3.15	0	28	0.024	1	U	K.GTIQELGQNQYAVSGEIFVVDR.N
10320	918 - 939	808.4094	2422.2065	2422.2129	-2.62	0	22	0.013	1	U	K.GTIQELGQNQYAVSGEIFVVDR.N
10321	918 - 939	1212.1110	2422.2075	2422.2129	-2.21	0	53	2.2e-05	1	U	K.GTIQELGQNQYAVSGEIFVVDR.N
10323	918 - 939	1212.1119	2422.2092	2422.2129	-1.52	0	115	3e-11	1	U	K.GTIQELGQNQYAVSGEIFVVDR.N
10325	918 - 939	808.4109	2422.2109	2422.2129	-0.82	0	20	0.041	1	U	K.GTIQELGQNQYAVSGEIFVVDR.N
10326	918 - 939	808.4110	2422.2113	2422.2129	-0.65	0	25	0.033	1	U	K.GTIQELGQNQYAVSGEIFVVDR.N
10327	918 - 939	808.4110	2422.2113	2422.2129	-0.65	0	29	0.014	1	U	K.GTIQELGQNQYAVSGEIFVVDR.N
10328	918 - 939	808.4111	2422.2114	2422.2129	-0.59	0	49	0.0011	1	U	K.GTIQELGQNQYAVSGEIFVVDR.N
10329	918 - 939	1212.1130	2422.2115	2422.2129	-0.56	0	129	9.1e-12	1	U	K.GTIQELGQNQYAVSGEIFVVDR.N
10330	918 - 939	808.4114	2422.2122	2422.2129	-0.26	0	32	0.021	1	U	K.GTIQELGQNQYAVSGEIFVVDR.N
10331	918 - 939	606.5604	2422.2123	2422.2129	-0.22	0	36	0.0061	1	U	K.GTIQELGQNQYAVSGEIFVVDR.N
10332	918 - 939	808.4115	2422.2127	2422.2129	-0.065	0	40	0.00093	1	U	K.GTIQELGQNQYAVSGEIFVVDR.N
10336	918 - 939	808.4116	2422.2130	2422.2129	0.046	0	44	0.00041	1	U	K.GTIQELGQNQYAVSGEIFVVDR.N
10338	918 - 939	1212.1139	2422.2133	2422.2129	0.18	0	24	0.024	1	U	K.GTIQELGQNQYAVSGEIFVVDR.N
10344	918 - 939	808.4122	2422.2149	2422.2129	0.84	0	44	0.0056	1	U	K.GTIQELGQNQYAVSGEIFVVDR.N
10345	918 - 939	808.4123	2422.2150	2422.2129	0.86	0	31	0.0038	1	U	K.GTIQELGQNQYAVSGEIFVVDR.N
10348	918 - 939	1212.1153	2422.2160	2422.2129	1.30	0	64	6.1e-06	1	U	K.GTIQELGQNQYAVSGEIFVVDR.N
10349	918 - 939	808.4126	2422.2161	2422.2129	1.32	0	49	0.00052	1	U	K.GTIQELGQNQYAVSGEIFVVDR.N
10354	918 - 939	808.4140	2422.2203	2422.2129	3.07	0	23	0.013	1	U	K.GTIQELGQNQYAVSGEIFVVDR.N
3705	940 - 950	635.8525	1269.6905	1269.6929	-1.88	0	36	0.0049	1	U	R.NTVEITELPVR.T
3706	940 - 950	635.8526	1269.6906	1269.6929	-1.80	0	51	0.00025	1	U	R.NTVEITELPVR.T
3708	940 - 950	635.8532	1269.6918	1269.6929	-0.81	0	67	8.3e-06	1	U	R.NTVEITELPVR.T
3710	940 - 950	635.8535	1269.6924	1269.6929	-0.37	0	73	2.4e-06	1	U	R.NTVEITELPVR.T
3711	940 - 950	635.8535	1269.6924	1269.6929	-0.34	0	36	0.0027	1	U	R.NTVEITELPVR.T
3712	940 - 950	635.8536	1269.6925	1269.6929	-0.24	0	54	0.00022	1	U	R.NTVEITELPVR.T
3714	940 - 950	635.8537	1269.6929	1269.6929	0.041	0	55	0.00017	1	U	R.NTVEITELPVR.T
3715	940 - 950	635.8538	1269.6930	1269.6929	0.10	0	52	5.3e-05	1	U	R.NTVEITELPVR.T
3717	940 - 950	424.2383	1269.6931	1269.6929	0.22	0	53	0.00028	1	U	R.NTVEITELPVR.T
3718	940 - 950	635.8540	1269.6935	1269.6929	0.53	0	29	0.013	1	U	R.NTVEITELPVR.T
3719	940 - 950	635.8541	1269.6936	1269.6929	0.62	0	34	0.0022	1	U	R.NTVEITELPVR.T
3720	940 - 950	635.8541	1269.6937	1269.6929	0.66	0	30	0.0078	1	U	R.NTVEITELPVR.T
5288	958 - 970	737.3668	1472.7190	1472.7181	0.62	0	28	0.015	1	U	K.EQVLEPMLNGTDK.T
5410	958 - 970	745.3629	1488.7113	1488.7130	-1.15	0	42	0.00073	1	U	K.EQVLEPMLNGTDK.T + Oxidation (M)
5413	958 - 970	745.3634	1488.7122	1488.7130	-0.50	0	50	0.00016	1	U	K.EQVLEPMLNGTDK.T + Oxidation (M)
10519	958 - 979	826.7526	2477.2358	2477.2359	-0.044	1	34	0.0058	1	U	K.EQVLEPMLNGTDKTPALISDYK.E + Oxidation (M)
10521	958 - 979	826.7545	2477.2417	2477.2359	2.32	1	21	0.042	1	U	K.EQVLEPMLNGTDKTPALISDYK.E + Oxidation (M)
1571	971 - 979	504.2740	1006.5335	1006.5335	-0.0050	0	31	0.01	1	U	K.TPALISDYK.E
1572	971 - 979	504.2741	1006.5337	1006.5335	0.17	0	52	0.00019	1	U	K.TPALISDYK.E
1573	971 - 979	504.2741	1006.5337	1006.5335	0.19	0	15	0.042	1	U	K.TPALISDYK.E
1574	971 - 979	504.2741	1006.5337	1006.5335	0.21	0	30	0.011	1	U	K.TPALISDYK.E
9302	971 - 988	521.2650	2081.0308	2081.0317	-0.42	1	27	0.03	1	U	K.TPALISDYKEYHTDTTVK.F
9303	971 - 988	694.6842	2081.0309	2081.0317	-0.40	1	37	0.00095	1	U	K.TPALISDYKEYHTDTTVK.F
9306	971 - 988	1041.5232	2081.0319	2081.0317	0.10	1	61	5.7e-06	1	U	K.TPALISDYKEYHTDTTVK.F
9307	971 - 988	694.6846	2081.0319	2081.0317	0.11	1	26	0.011	1	U	K.TPALISDYKEYHTDTTVK.F
9311	971 - 988	1041.5246	2081.0346	2081.0317	1.39	1	23	0.013	1	U	K.TPALISDYKEYHTDTTVK.F
2258	980 - 988	547.2616	1092.5087	1092.5088	-0.028	0	26	0.028	1		K.EYHTDTTVK.F
2261	980 - 988	547.2619	1092.5093	1092.5088	0.48	0	31	0.013	1		K.EYHTDTTVK.F
2262	980 - 988	547.2622	1092.5098	1092.5088	0.94	0	28	0.027	1		K.EYHTDTTVK.F
2374	998 - 1008	370.2084	1107.6033	1107.6036	-0.33	0	45	0.0012	1	U	K.LAQAEAAGLHK.V
2375	998 - 1008	554.8089	1107.6033	1107.6036	-0.32	0	43	0.00039	1	U	K.LAQAEAAGLHK.V
2376	998 - 1008	554.8090	1107.6034	1107.6036	-0.18	0	54	7.3e-05	1	U	K.LAQAEAAGLHK.V
2377	998 - 1008	370.2084	1107.6035	1107.6036	-0.12	0	39	0.0028	1	U	K.LAQAEAAGLHK.V
2378	998 - 1008	554.8091	1107.6036	1107.6036	0.023	0	30	0.048	1	U	K.LAQAEAAGLHK.V
2379	998 - 1008	554.8091	1107.6037	1107.6036	0.096	0	49	0.0006	1	U	K.LAQAEAAGLHK.V
2380	998 - 1008	370.2085	1107.6037	1107.6036	0.099	0	37	0.004	1	U	K.LAQAEAAGLHK.V
2381	998 - 1008	370.2086	1107.6039	1107.6036	0.23	0	36	0.0043	1	U	K.LAQAEAAGLHK.V
3401	1032 - 1041	618.8446	1235.6747	1235.6761	-1.15	1	33	0.0067	1	U	K.KYETVQDILK.E
3402	1032 - 1041	618.8453	1235.6760	1235.6761	-0.084	1	38	0.00066	1	U	K.KYETVQDILK.E
3403	1032 - 1041	618.8454	1235.6763	1235.6761	0.11	1	58	1.5e-05	1	U	K.KYETVQDILK.E
3404	1032 - 1041	412.8994	1235.6764	1235.6761	0.24	1	39	0.006	1	U	K.KYETVQDILK.E
3405	1032 - 1041	618.8455	1235.6765	1235.6761	0.32	1	48	0.00051	1	U	K.KYETVQDILK.E
3408	1032 - 1041	618.8457	1235.6769	1235.6761	0.60	1	66	2.1e-06	1	U	K.KYETVQDILK.E
2372	1033 - 1041	554.7984	1107.5823	1107.5812	1.00	0	37	0.012	1	U	K.YETVQDILK.E
727	1048 - 1054	436.2371	870.4597	870.4599	-0.26	0	31	0.021	1	U	R.LSYYGLR.K
729	1048 - 1054	436.2377	870.4609	870.4599	1.10	0	39	0.002	1	U	R.LSYYGLR.K
6081	1055 - 1068	774.9078	1547.8011	1547.8017	-0.42	1	48	0.00031	1	U	R.KEWLVGMLGAESTK.L
6082	1055 - 1068	774.9082	1547.8019	1547.8017	0.13	1	69	9.2e-06	1	U	R.KEWLVGMLGAESTK.L
6083	1055 - 1068	516.9418	1547.8036	1547.8017	1.18	1	33	0.0056	1	U	R.KEWLVGMLGAESTK.L

Query	Start - End	Observed	Mr(expt)	Mr(calc)	ppm	M	Score	Expect	Rank	U	Peptide
6216	1055 - 1068	782.9049	1563.7952	1563.7967	-0.92	1	36	0.0022	1	U	R.KEWLVGMLGAESTK.L + Oxidation (M)
6218	1055 - 1068	782.9054	1563.7962	1563.7967	-0.26	1	70	1.6e-06	1	U	R.KEWLVGMLGAESTK.L + Oxidation (M)
6219	1055 - 1068	522.2732	1563.7977	1563.7967	0.63	1	27	0.022	1	U	R.KEWLVGMLGAESTK.L + Oxidation (M)
6220	1055 - 1068	522.2735	1563.7988	1563.7967	1.34	1	25	0.02	1	U	R.KEWLVGMLGAESTK.L + Oxidation (M)
4796	1056 - 1068	710.8608	1419.7070	1419.7068	0.13	0	22	0.021	1	U	K.EWLVGMLGAESTK.L
4797	1056 - 1068	710.8609	1419.7072	1419.7068	0.32	0	50	2.3e-05	1	U	K.EWLVGMLGAESTK.L
4798	1056 - 1068	710.8614	1419.7082	1419.7068	0.99	0	38	0.00052	1	U	K.EWLVGMLGAESTK.L
4954	1056 - 1068	718.8578	1435.7010	1435.7017	-0.47	0	35	0.0096	1	U	K.EWLVGMLGAESTK.L + Oxidation (M)
3487	1092 - 1101	622.3632	1242.7118	1242.7118	0.00080	1	59	4.9e-05	1	U	K.KDLIQMLVQR.G
3488	1092 - 1101	415.2447	1242.7122	1242.7118	0.30	1	37	0.0083	1	U	K.KDLIQMLVQR.G
3619	1092 - 1101	630.3608	1258.7070	1258.7067	0.26	1	51	0.00049	1	U	K.KDLIQMLVQR.G + Oxidation (M)
3620	1092 - 1101	630.3610	1258.7075	1258.7067	0.65	1	55	0.00013	1	U	K.KDLIQMLVQR.G + Oxidation (M)
3621	1092 - 1101	420.5766	1258.7079	1258.7067	0.94	1	46	0.0011	1	U	K.KDLIQMLVQR.G + Oxidation (M)
2441	1093 - 1101	558.3156	1114.6166	1114.6169	-0.24	0	51	0.00044	1	U	K.DLIQMLVQR.G
2442	1093 - 1101	558.3158	1114.6170	1114.6169	0.10	0	45	0.0011	1	U	K.DLIQMLVQR.G
2443	1093 - 1101	558.3159	1114.6172	1114.6169	0.36	0	55	0.0002	1	U	K.DLIQMLVQR.G
2444	1093 - 1101	558.3159	1114.6173	1114.6169	0.41	0	51	0.00047	1	U	K.DLIQMLVQR.G
2589	1093 - 1101	566.3121	1130.6096	1130.6118	-1.93	0	38	0.0069	1	U	K.DLIQMLVQR.G + Oxidation (M)
2591	1093 - 1101	566.3122	1130.6099	1130.6118	-1.65	0	54	0.00025	1	U	K.DLIQMLVQR.G + Oxidation (M)
2592	1093 - 1101	566.3129	1130.6113	1130.6118	-0.41	0	38	0.0031	1	U	K.DLIQMLVQR.G + Oxidation (M)
2593	1093 - 1101	566.3130	1130.6114	1130.6118	-0.34	0	39	0.0088	1	U	K.DLIQMLVQR.G + Oxidation (M)
2594	1093 - 1101	566.3132	1130.6119	1130.6118	0.14	0	40	0.0031	1	U	K.DLIQMLVQR.G + Oxidation (M)
2595	1093 - 1101	566.3133	1130.6120	1130.6118	0.21	0	48	0.00099	1	U	K.DLIQMLVQR.G + Oxidation (M)
2596	1093 - 1101	566.3133	1130.6120	1130.6118	0.23	0	51	0.0005	1	U	K.DLIQMLVQR.G + Oxidation (M)
2597	1093 - 1101	566.3133	1130.6121	1130.6118	0.26	0	41	0.0049	1	U	K.DLIQMLVQR.G + Oxidation (M)
2598	1093 - 1101	566.3134	1130.6123	1130.6118	0.49	0	28	0.022	1	U	K.DLIQMLVQR.G + Oxidation (M)
2599	1093 - 1101	566.3135	1130.6124	1130.6118	0.58	0	51	0.00029	1	U	K.DLIQMLVQR.G + Oxidation (M)
2600	1093 - 1101	566.3136	1130.6126	1130.6118	0.70	0	43	0.0019	1	U	K.DLIQMLVQR.G + Oxidation (M)
823	1102 - 1109	447.7137	893.4129	893.4131	-0.19	0	47	8.2e-05	1	U	R.GYESDPVK.A
824	1102 - 1109	447.7138	893.4130	893.4131	-0.075	0	28	0.0076	1	U	R.GYESDPVK.A
1419	1157 - 1164	494.2898	986.5651	986.5648	0.34	1	40	0.0045	1	U	K.EKVEELIK.Q
1421	1157 - 1164	494.2899	986.5653	986.5648	0.56	1	34	0.018	2	U	K.EKVEELIK.Q
2198	1170 - 1178	543.8046	1085.5946	1085.5941	0.48	2	40	0.0053	1	U	K.GREVNDLKR.K
2199	1170 - 1178	362.8725	1085.5956	1085.5941	1.36	2	42	0.0035	1	U	K.GREVNDLKR.K
737	1172 - 1178	437.2427	872.4709	872.4716	-0.79	1	37	0.01	1	U	R.EVNDLKR.K
526	1180 - 1186	416.7138	831.4130	831.4127	0.37	0	33	0.025	1		K.SPSDLWK.E
4546	1187 - 1198	689.8403	1377.6661	1377.6664	-0.16	0	51	0.00025	1	U	K.EDLAAFVEELDK.V
4547	1187 - 1198	689.8404	1377.6662	1377.6664	-0.075	0	24	0.011	1	U	K.EDLAAFVEELDK.V
4548	1187 - 1198	689.8405	1377.6665	1377.6664	0.13	0	54	7.9e-05	1	U	K.EDLAAFVEELDK.V
4550	1187 - 1198	689.8407	1377.6668	1377.6664	0.36	0	30	0.008	1	U	K.EDLAAFVEELDK.V
9415	1187 - 1204	1054.0101	2106.0056	2106.0117	-2.86	1	72	1.3e-06	1	U	K.EDLAAFVEELDKVESQER.E
9416	1187 - 1204	703.0099	2106.0078	2106.0117	-1.81	1	49	0.00014	1	U	K.EDLAAFVEELDKVESQER.E
9417	1187 - 1204	703.0103	2106.0092	2106.0117	-1.19	1	29	0.018	1	U	K.EDLAAFVEELDKVESQER.E
9418	1187 - 1204	1054.0122	2106.0099	2106.0117	-0.82	1	77	4.6e-07	1	U	K.EDLAAFVEELDKVESQER.E
9419	1187 - 1204	1054.0124	2106.0102	2106.0117	-0.71	1	79	2.4e-07	1	U	K.EDLAAFVEELDKVESQER.E
9420	1187 - 1204	703.0108	2106.0106	2106.0117	-0.50	1	31	0.013	1	U	K.EDLAAFVEELDKVESQER.E
9421	1187 - 1204	703.0109	2106.0109	2106.0117	-0.37	1	33	0.0097	1	U	K.EDLAAFVEELDKVESQER.E
9422	1187 - 1204	1054.0127	2106.0109	2106.0117	-0.36	1	61	6.9e-06	1	U	K.EDLAAFVEELDKVESQER.E
9423	1187 - 1204	1054.0127	2106.0109	2106.0117	-0.35	1	84	5e-08	1	U	K.EDLAAFVEELDKVESQER.E
9424	1187 - 1204	703.0110	2106.0111	2106.0117	-0.25	1	33	0.007	1	U	K.EDLAAFVEELDKVESQER.E
9426	1187 - 1204	703.0112	2106.0118	2106.0117	0.068	1	32	0.012	1	U	K.EDLAAFVEELDKVESQER.E
9427	1187 - 1204	703.0113	2106.0120	2106.0117	0.15	1	33	0.02	1	U	K.EDLAAFVEELDKVESQER.E
9428	1187 - 1204	703.0113	2106.0120	2106.0117	0.18	1	40	0.0027	1	U	K.EDLAAFVEELDKVESQER.E
9429	1187 - 1204	1054.0133	2106.0121	2106.0117	0.22	1	43	0.0002	1	U	K.EDLAAFVEELDKVESQER.E
9430	1187 - 1204	703.0113	2106.0121	2106.0117	0.23	1	35	0.0077	1	U	K.EDLAAFVEELDKVESQER.E
9431	1187 - 1204	703.0114	2106.0123	2106.0117	0.30	1	38	0.0063	1	U	K.EDLAAFVEELDKVESQER.E
9432	1187 - 1204	703.0114	2106.0123	2106.0117	0.32	1	25	0.047	1	U	K.EDLAAFVEELDKVESQER.E
9433	1187 - 1204	703.0114	2106.0124	2106.0117	0.35	1	40	0.003	1	U	K.EDLAAFVEELDKVESQER.E
9434	1187 - 1204	1054.0135	2106.0124	2106.0117	0.38	1	55	5.2e-05	1	U	K.EDLAAFVEELDKVESQER.E
9435	1187 - 1204	703.0114	2106.0125	2106.0117	0.41	1	27	0.023	1	U	K.EDLAAFVEELDKVESQER.E
9436	1187 - 1204	703.0115	2106.0127	2106.0117	0.48	1	40	0.0014	1	U	K.EDLAAFVEELDKVESQER.E
9437	1187 - 1204	703.0115	2106.0127	2106.0117	0.48	1	27	0.024	1	U	K.EDLAAFVEELDKVESQER.E
9438	1187 - 1204	1054.0137	2106.0129	2106.0117	0.57	1	68	5.5e-06	1	U	K.EDLAAFVEELDKVESQER.E
9439	1187 - 1204	1054.0141	2106.0136	2106.0117	0.94	1	42	0.00014	1	U	K.EDLAAFVEELDKVESQER.E
9440	1187 - 1204	1054.0143	2106.0141	2106.0117	1.15	1	60	3.4e-06	1	U	K.EDLAAFVEELDKVESQER.E
9441	1187 - 1204	1054.0146	2106.0146	2106.0117	1.42	1	64	1.7e-06	1	U	K.EDLAAFVEELDKVESQER.E
11867	1187 - 1214	1032.1673	3093.4800	3093.4812	-0.37	2	59	3.2e-05	1	U	K.EDLAAFVEELDKVESQEREDVLAGMSGK.A
11870	1187 - 1214	1032.1680	3093.4823	3093.4812	0.36	2	49	0.00011	1	U	K.EDLAAFVEELDKVESQEREDVLAGMSGK.A
11923	1187 - 1214	1037.4980	3109.4721	3109.4761	-1.30	2	27	0.013	1	U	K.EDLAAFVEELDKVESQEREDVLAGMSGK.A + Oxidation (M)
11924	1187 - 1214	1037.4986	3109.4739	3109.4761	-0.71	2	58	4.8e-05	1	U	K.EDLAAFVEELDKVESQEREDVLAGMSGK.A

Query	Start - End	Observed	Mr(expt)	Mr(calc)	ppm	M	Score	Expect	Rank	U	Peptide
											+ Oxidation (M)
11931	1187 - 1214	1037.5003	3109.4790	3109.4761	0.94	2	32	0.0069	1	U	K.EDLAAFVEELDKVESQEREDVLAGMSGK.A + Oxidation (M)
7493	1199 - 1214	867.9199	1733.8252	1733.8254	-0.096	1	51	5.1e-05	1	U	K.VESQEREDVLAGMSGK.A
7602	1199 - 1214	584.2804	1749.8194	1749.8203	-0.52	1	30	0.011	1	U	K.VESQEREDVLAGMSGK.A + Oxidation (M)
7603	1199 - 1214	875.9179	1749.8213	1749.8203	0.57	1	65	2.4e-05	1	U	K.VESQEREDVLAGMSGK.A + Oxidation (M)
1554	1205 - 1214	503.7475	1005.4804	1005.4801	0.32	0	46	0.00031	1	U	R.EDVLAGMSGK.A
1677	1205 - 1214	511.7446	1021.4745	1021.4750	-0.45	0	52	0.00015	1	U	R.EDVLAGMSGK.A + Oxidation (M)
1678	1205 - 1214	511.7447	1021.4749	1021.4750	-0.14	0	36	0.0038	1	U	R.EDVLAGMSGK.A + Oxidation (M)
1679	1205 - 1214	511.7448	1021.4750	1021.4750	-0.018	0	39	0.0021	1	U	R.EDVLAGMSGK.A + Oxidation (M)
1680	1205 - 1214	511.7449	1021.4752	1021.4750	0.24	0	53	0.00011	1	U	R.EDVLAGMSGK.A + Oxidation (M)
1681	1205 - 1214	511.7449	1021.4753	1021.4750	0.32	0	51	0.00016	1	U	R.EDVLAGMSGK.A + Oxidation (M)
1682	1205 - 1214	511.7449	1021.4753	1021.4750	0.32	0	48	0.00033	1	U	R.EDVLAGMSGK.A + Oxidation (M)
1683	1205 - 1214	511.7451	1021.4756	1021.4750	0.59	0	49	0.00028	1	U	R.EDVLAGMSGK.A + Oxidation (M)
6849	1227 - 1240	824.9221	1647.8297	1647.8290	0.42	1	83	1.2e-07	1	U	K.KLQLEETMPSPYGR.R
6934	1227 - 1240	832.9184	1663.8223	1663.8239	-0.95	1	76	4.1e-07	1	U	K.KLQLEETMPSPYGR.R + Oxidation (M)
6935	1227 - 1240	832.9186	1663.8227	1663.8239	-0.76	1	71	1.6e-06	1	U	K.KLQLEETMPSPYGR.R + Oxidation (M)
6937	1227 - 1240	832.9187	1663.8229	1663.8239	-0.60	1	68	9.2e-07	1	U	K.KLQLEETMPSPYGR.R + Oxidation (M)
6938	1227 - 1240	832.9188	1663.8230	1663.8239	-0.57	1	58	1.2e-05	1	U	K.KLQLEETMPSPYGR.R + Oxidation (M)
6939	1227 - 1240	555.6149	1663.8230	1663.8239	-0.56	1	18	0.036	1	U	K.KLQLEETMPSPYGR.R + Oxidation (M)
6940	1227 - 1240	555.6150	1663.8231	1663.8239	-0.53	1	20	0.027	1	U	K.KLQLEETMPSPYGR.R + Oxidation (M)
6941	1227 - 1240	832.9190	1663.8234	1663.8239	-0.34	1	71	5.5e-07	1	U	K.KLQLEETMPSPYGR.R + Oxidation (M)
6942	1227 - 1240	555.6152	1663.8239	1663.8239	-0.022	1	29	0.007	1	U	K.KLQLEETMPSPYGR.R + Oxidation (M)
6943	1227 - 1240	555.6152	1663.8239	1663.8239	-0.0042	1	27	0.013	1	U	K.KLQLEETMPSPYGR.R + Oxidation (M)
6944	1227 - 1240	832.9195	1663.8245	1663.8239	0.36	1	64	2.4e-06	1	U	K.KLQLEETMPSPYGR.R + Oxidation (M)
6945	1227 - 1240	832.9196	1663.8246	1663.8239	0.38	1	51	3.9e-05	1	U	K.KLQLEETMPSPYGR.R + Oxidation (M)
6946	1227 - 1240	832.9196	1663.8246	1663.8239	0.38	1	76	1.7e-07	1	U	K.KLQLEETMPSPYGR.R + Oxidation (M)
6947	1227 - 1240	555.6155	1663.8246	1663.8239	0.39	1	21	0.026	1	U	K.KLQLEETMPSPYGR.R + Oxidation (M)
5769	1228 - 1240	507.5854	1519.7343	1519.7341	0.15	0	31	0.011	1	U	K.LQLEETMPSPYGR.R
5770	1228 - 1240	760.8745	1519.7344	1519.7341	0.22	0	60	1.1e-05	1	U	K.LQLEETMPSPYGR.R
5771	1228 - 1240	760.8746	1519.7347	1519.7341	0.40	0	55	1.4e-05	1	U	K.LQLEETMPSPYGR.R
5953	1228 - 1240	768.8716	1535.7285	1535.7290	-0.28	0	55	4e-05	1	U	K.LQLEETMPSPYGR.R + Oxidation (M)
5954	1228 - 1240	768.8717	1535.7288	1535.7290	-0.13	0	61	3.7e-06	1	U	K.LQLEETMPSPYGR.R + Oxidation (M)
5955	1228 - 1240	768.8717	1535.7288	1535.7290	-0.094	0	67	1.5e-06	1	U	K.LQLEETMPSPYGR.R + Oxidation (M)
5956	1228 - 1240	768.8717	1535.7289	1535.7290	-0.068	0	69	2.1e-06	1	U	K.LQLEETMPSPYGR.R + Oxidation (M)
5957	1228 - 1240	768.8718	1535.7290	1535.7290	0.0098	0	57	1.6e-05	1	U	K.LQLEETMPSPYGR.R + Oxidation (M)
5958	1228 - 1240	768.8718	1535.7290	1535.7290	0.036	0	56	2.3e-05	1	U	K.LQLEETMPSPYGR.R + Oxidation (M)
5959	1228 - 1240	768.8718	1535.7290	1535.7290	0.049	0	43	0.00052	1	U	K.LQLEETMPSPYGR.R + Oxidation (M)
2942	1241 - 1250	586.3470	1170.6795	1170.6794	0.10	1	45	0.00043	1	U	R.RIIPEITAMK.A
2943	1241 - 1250	391.2339	1170.6798	1170.6794	0.35	1	37	0.00087	1	U	R.RIIPEITAMK.A
3032	1241 - 1250	594.3440	1186.6735	1186.6743	-0.67	1	35	0.00065	1	U	R.RIIPEITAMK.A + Oxidation (M)
3033	1241 - 1250	396.5652	1186.6737	1186.6743	-0.59	1	32	0.0023	1	U	R.RIIPEITAMK.A + Oxidation (M)
3036	1241 - 1250	594.3446	1186.6746	1186.6743	0.19	1	49	4e-05	1	U	R.RIIPEITAMK.A + Oxidation (M)
3037	1241 - 1250	594.3446	1186.6747	1186.6743	0.27	1	38	0.00097	1	U	R.RIIPEITAMK.A + Oxidation (M)
3038	1241 - 1250	594.3446	1186.6747	1186.6743	0.32	1	39	0.00051	1	U	R.RIIPEITAMK.A + Oxidation (M)
1632	1242 - 1250	508.2957	1014.5769	1014.5783	-1.43	0	21	0.03	1	U	R.IIPEITAMK.A
1638	1242 - 1250	508.2965	1014.5785	1014.5783	0.16	0	27	0.047	1	U	R.IIPEITAMK.A
2719	1261 - 1271	573.3298	1144.6451	1144.6452	-0.021	2	66	1.4e-05	1	U	K.KKGDLDTAAVK.V
1655	1262 - 1271	509.2823	1016.5500	1016.5502	-0.21	1	45	0.0019	1	U	K.KGDLDTAAVK.V
1656	1262 - 1271	509.2823	1016.5501	1016.5502	-0.13	1	43	0.0012	1	U	K.KGDLDTAAVK.V
1657	1262 - 1271	509.2824	1016.5502	1016.5502	-0.048	1	58	8.3e-05	1	U	K.KGDLDTAAVK.V
811	1263 - 1271	445.2350	888.4554	888.4553	0.19	0	26	0.032	1	U	K.GDLDTAAVK.V
812	1263 - 1271	445.2353	888.4560	888.4553	0.80	0	50	0.00057	1	U	K.GDLDTAAVK.V
11344	1272 - 1299	973.4681	2917.3824	2917.3869	-1.56	0	25	0.018	1	U	K.VEFDEEFSGAPVEGAGEEALTPSVPINK.G
11347	1272 - 1299	973.4684	2917.3834	2917.3869	-1.19	0	47	0.00031	1	U	K.VEFDEEFSGAPVEGAGEEALTPSVPINK.G
11348	1272 - 1299	973.4686	2917.3838	2917.3869	-1.06	0	24	0.025	1	U	K.VEFDEEFSGAPVEGAGEEALTPSVPINK.G
11350	1272 - 1299	973.4690	2917.3852	2917.3869	-0.59	0	29	0.011	1	U	K.VEFDEEFSGAPVEGAGEEALTPSVPINK.G

Query	Start – End	Observed	Mr(expt)	Mr(calc)	ppm	M	Score	Expect	Rank	U	Peptide
11351	1272 – 1299	973.4690	2917.3852	2917.3869	-0.58	0	64	9.5e-06	1	U	K.VEFDEEFSGAPVEGAGEEALTPSVPINK.G
11352	1272 – 1299	973.4692	2917.3857	2917.3869	-0.43	0	27	0.012	1	U	K.VEFDEEFSGAPVEGAGEEALTPSVPINK.G
11353	1272 – 1299	973.4692	2917.3859	2917.3869	-0.36	0	35	0.0061	1	U	K.VEFDEEFSGAPVEGAGEEALTPSVPINK.G
11355	1272 – 1299	973.4693	2917.3862	2917.3869	-0.24	0	55	5.4e-05	1	U	K.VEFDEEFSGAPVEGAGEEALTPSVPINK.G
11356	1272 – 1299	973.4694	2917.3863	2917.3869	-0.20	0	30	0.013	1	U	K.VEFDEEFSGAPVEGAGEEALTPSVPINK.G
11359	1272 – 1299	973.4695	2917.3868	2917.3869	-0.038	0	22	0.027	1	U	K.VEFDEEFSGAPVEGAGEEALTPSVPINK.G
11362	1272 – 1299	973.4698	2917.3877	2917.3869	0.27	0	50	0.00015	1	U	K.VEFDEEFSGAPVEGAGEEALTPSVPINK.G
11363	1272 – 1299	973.4699	2917.3878	2917.3869	0.32	0	19	0.033	1	U	K.VEFDEEFSGAPVEGAGEEALTPSVPINK.G
11364	1272 – 1299	973.4699	2917.3880	2917.3869	0.36	0	31	0.0048	1	U	K.VEFDEEFSGAPVEGAGEEALTPSVPINK.G
11366	1272 – 1299	973.4702	2917.3887	2917.3869	0.61	0	25	0.023	1	U	K.VEFDEEFSGAPVEGAGEEALTPSVPINK.G
11367	1272 – 1299	973.4702	2917.3887	2917.3869	0.62	0	33	0.0037	1	U	K.VEFDEEFSGAPVEGAGEEALTPSVPINK.G
11370	1272 – 1299	973.4705	2917.3896	2917.3869	0.93	0	51	0.00034	1	U	K.VEFDEEFSGAPVEGAGEEALTPSVPINK.G
11372	1272 – 1299	973.4708	2917.3905	2917.3869	1.24	0	22	0.045	1	U	K.VEFDEEFSGAPVEGAGEEALTPSVPINK.G
11373	1272 – 1299	730.3550	2917.3908	2917.3869	1.34	0	45	0.0066	1	U	K.VEFDEEFSGAPVEGAGEEALTPSVPINK.G
11375	1272 – 1299	973.4730	2917.3972	2917.3869	3.53	0	38	0.0014	1	U	K.VEFDEEFSGAPVEGAGEEALTPSVPINK.G
11653	1272 – 1299	1000.1234	2997.3484	2997.3532	-1.62	0	40	0.00073	1	U	K.VEFDEEFSGAPVEGAGEEALTPSVPINK.G + Phospho (ST)
11654	1272 – 1299	1000.1254	2997.3544	2997.3532	0.39	0	34	0.0049	1	U	K.VEFDEEFSGAPVEGAGEEALTPSVPINK.G + Phospho (ST)
3053	1316 – 1327	396.8908	1187.6507	1187.6510	-0.27	1	20	0.049	1	U	R.KTPTSSGKPSAK.K
3054	1316 – 1327	594.8327	1187.6509	1187.6510	-0.083	1	60	2.5e-05	1	U	R.KTPTSSGKPSAK.K
3056	1316 – 1327	396.8909	1187.6510	1187.6510	-0.020	1	38	0.0018	1	U	R.KTPTSSGKPSAK.K
3060	1316 – 1327	594.8331	1187.6516	1187.6510	0.54	1	48	0.00022	1	U	R.KTPTSSGKPSAK.K
1966	1317 – 1327	530.7854	1059.5563	1059.5560	0.22	0	37	0.00096	1	U	K.TPTSSGKPSAK.K
11335	1332 – 1356	972.1280	2913.3623	2913.3628	-0.17	2	70	5.6e-07	1	U	K.RNPWSDDESKSESDLEETEPVVIPR.D
11336	1332 – 1356	972.1282	2913.3628	2913.3628	0.013	2	76	3.2e-07	1	U	K.RNPWSDDESKSESDLEETEPVVIPR.D
11337	1332 – 1356	729.3481	2913.3631	2913.3628	0.12	2	28	0.03	1	U	K.RNPWSDDESKSESDLEETEPVVIPR.D
11644	1332 – 1356	998.7840	2993.3303	2993.3291	0.40	2	35	0.019	1	U	K.RNPWSDDESKSESDLEETEPVVIPR.D + Phospho (ST)
2104	1333 – 1341	539.2277	1076.4408	1076.4411	-0.27	0	40	0.0013	1	U	R.NPWSDDESK.S
11079	1333 – 1356	920.0938	2757.2596	2757.2617	-0.77	1	70	2.9e-06	1	U	R.NPWSDDESKSESDLEETEPVVIPR.D
11080	1333 – 1356	920.0943	2757.2610	2757.2617	-0.23	1	34	0.0076	1	U	R.NPWSDDESKSESDLEETEPVVIPR.D
7239	1342 – 1356	850.4230	1698.8314	1698.8312	0.15	0	77	1.8e-06	1	U	K.SESDLEETEPVVIPR.D
151	1363 – 1369	371.7140	741.4134	741.4133	0.17	0	34	0.0034	1	U	R.AAAERPK.Y
12205	1370 – 1396	1076.7360	3227.1861	3227.1858	0.090	0	46	0.00027	1	U	K.YTFDFSEEEDDDADDDDDDNNDLEELK.V
12206	1370 – 1396	1076.7361	3227.1865	3227.1858	0.21	0	46	0.00022	1	U	K.YTFDFSEEEDDDADDDDDDNNDLEELK.V
10279	1397 – 1418	805.0311	2412.0714	2412.0734	-0.80	1	31	0.018	1	U	K.VKASPITNDGEDEFVPSDGLDK.D + Phospho (ST)
10280	1397 – 1418	805.0319	2412.0738	2412.0734	0.18	1	34	0.016	1	U	K.VKASPITNDGEDEFVPSDGLDK.D + Phospho (ST)
12585	1397 – 1427	860.1374	3436.5206	3436.5236	-0.86	2	56	0.00021	1	U	K.VKASPITNDGEDEFVPSDGLDKDEYTFSPGK.S + Phospho (ST)
12586	1397 – 1427	860.1376	3436.5211	3436.5236	-0.71	2	36	0.0041	1	U	K.VKASPITNDGEDEFVPSDGLDKDEYTFSPGK.S + Phospho (ST)
12587	1397 – 1427	1146.5146	3436.5219	3436.5236	-0.47	2	69	1e-05	1	U	K.VKASPITNDGEDEFVPSDGLDKDEYTFSPGK.S + Phospho (ST)
12588	1397 – 1427	860.1379	3436.5224	3436.5236	-0.34	2	46	0.00089	1	U	K.VKASPITNDGEDEFVPSDGLDKDEYTFSPGK.S + Phospho (ST)
12589	1397 – 1427	860.1379	3436.5226	3436.5236	-0.29	2	64	8.2e-05	1	U	K.VKASPITNDGEDEFVPSDGLDKDEYTFSPGK.S + Phospho (ST)
12590	1397 – 1427	860.1382	3436.5238	3436.5236	0.063	2	47	0.00046	1	U	K.VKASPITNDGEDEFVPSDGLDKDEYTFSPGK.S + Phospho (ST)
12591	1397 – 1427	1146.5154	3436.5245	3436.5236	0.28	2	68	6.9e-06	1	U	K.VKASPITNDGEDEFVPSDGLDKDEYTFSPGK.S + Phospho (ST)
12592	1397 – 1427	1146.5159	3436.5260	3436.5236	0.71	2	73	2.2e-06	1	U	K.VKASPITNDGEDEFVPSDGLDKDEYTFSPGK.S + Phospho (ST)
9402	1399 – 1418	1053.4787	2104.9428	2104.9437	-0.41	0	66	1.1e-05	1	U	K.ASPITNDGEDEFVPSDGLDK.D
11983	1399 – 1427	783.3557	3129.3937	3129.3939	-0.065	1	47	0.00033	1	U	K.ASPITNDGEDEFVPSDGLDKDEYTFSPGK.S
11985	1399 – 1427	1044.1392	3129.3958	3129.3939	0.61	1	85	2.6e-07	1	U	K.ASPITNDGEDEFVPSDGLDKDEYTFSPGK.S
11986	1399 – 1427	1044.1395	3129.3966	3129.3939	0.86	1	78	5.3e-07	1	U	K.ASPITNDGEDEFVPSDGLDKDEYTFSPGK.S
12167	1399 – 1427	1070.7929	3209.3568	3209.3602	-1.04	1	66	2e-05	1	U	K.ASPITNDGEDEFVPSDGLDKDEYTFSPGK.S + Phospho (ST)
12168	1399 – 1427	1070.7939	3209.3598	3209.3602	-0.13	1	75	1.3e-06	1	U	K.ASPITNDGEDEFVPSDGLDKDEYTFSPGK.S + Phospho (ST)
12169	1399 – 1427	1070.7946	3209.3619	3209.3602	0.54	1	45	0.0003	1	U	K.ASPITNDGEDEFVPSDGLDKDEYTFSPGK.S + Phospho (ST)
1815	1419 – 1427	522.2376	1042.4605	1042.4607	-0.18	0	36	0.00082	1	U	K.DEYTFSPGK.S
8988	1440 – 1456	990.4784	1978.9423	1978.9425	-0.11	1	78	1.5e-07	1	U	K.KSQDFGNLFSFPSYSQK.S
8361	1441 – 1456	926.4303	1850.8460	1850.8475	-0.81	0	75	1.7e-07	1	U	K.SQDFGNLFSFPSYSQK.S
8362	1441 – 1456	926.4307	1850.8468	1850.8475	-0.37	0	91	5.7e-09	1	U	K.SQDFGNLFSFPSYSQK.S
8363	1441 – 1456	926.4307	1850.8469	1850.8475	-0.36	0	63	2.3e-06	1	U	K.SQDFGNLFSFPSYSQK.S
8364	1441 – 1456	617.9563	1850.8471	1850.8475	-0.23	0	30	0.0095	1	U	K.SQDFGNLFSFPSYSQK.S
8365	1441 – 1456	926.4312	1850.8478	1850.8475	0.13	0	67	4.9e-07	1	U	K.SQDFGNLFSFPSYSQK.S
8366	1441 – 1456	926.4312	1850.8478	1850.8475	0.14	0	84	1.3e-08	1	U	K.SQDFGNLFSFPSYSQK.S
8368	1441 – 1456	926.4313	1850.8481	1850.8475	0.30	0	51	1.7e-05	1	U	K.SQDFGNLFSFPSYSQK.S
8370	1441 – 1456	617.9568	1850.8487	1850.8475	0.61	0	51	0.00028	1	U	K.SQDFGNLFSFPSYSQK.S
8371	1441 – 1456	926.4317	1850.8489	1850.8475	0.74	0	74	3.4e-07	1	U	K.SQDFGNLFSFPSYSQK.S
8372	1441 – 1456	926.4318	1850.8490	1850.8475	0.81	0	62	7.9e-06	1	U	K.SQDFGNLFSFPSYSQK.S
8373	1441 – 1456	926.4322	1850.8499	1850.8475	1.26	0	79	1e-07	1	U	K.SQDFGNLFSFPSYSQK.S
8374	1441 – 1456	926.4328	1850.8511	1850.8475	1.94	0	82	6.3e-08	1	U	K.SQDFGNLFSFPSYSQK.S
8375	1441 – 1456	926.4352	1850.8559	1850.8475	4.51	0	72	2.3e-07	1	U	K.SQDFGNLFSFPSYSQK.S
11116	1457 – 1482	932.4093	2794.2060	2794.2093	-1.21	1	76	4.2e-07	1	U	K.SEDDSAKFDSNEEDSASVFSPSFGLK.Q
11118	1457 – 1482	932.4095	2794.2066	2794.2093	-1.00	1	53	6.5e-05	1	U	K.SEDDSAKFDSNEEDSASVFSPSFGLK.Q
11119	1457 – 1482	932.4098	2794.2077	2794.2093	-0.60	1	67	3.7e-06	1	U	K.SEDDSAKFDSNEEDSASVFSPSFGLK.Q
11120	1457 – 1482	932.4104	2794.2093	2794.2093	-0.0082	1	52	7.3e-05	1	U	K.SEDDSAKFDSNEEDSASVFSPSFGLK.Q
11121	1457 – 1482	932.4117	2794.2133	2794.2093	1.41	1	62	4.9e-06	1	U	K.SEDDSAKFDSNEEDSASVFSPSFGLK.Q
11266	1457 – 1482	959.0657	2874.1754	2874.1757	-0.093	1	33	0.0075	1	U	K.SEDDSAKFDSNEEDSASVFSPSFGLK.Q + Phospho (ST)
11268	1457 – 1482	959.0661	2874.1765	2874.1757	0.30	1	38	0.0012	1	U	K.SEDDSAKFDSNEEDSASVFSPSFGLK.Q + Phospho (ST)

Query	Start - End	Observed	Mr(expt)	Mr(calc)	ppm	M	Score	Expect	Rank	U	Peptide
9245	1464 - 1482	1031.9640	2061.9133	2061.9167	-1.64	0	18	0.029	1	U	K.FDSNEEDSASVFSPSFGLK.Q
9246	1464 - 1482	1031.9642	2061.9138	2061.9167	-1.41	0	83	3.6e-08	1	U	K.FDSNEEDSASVFSPSFGLK.Q
9247	1464 - 1482	688.3126	2061.9159	2061.9167	-0.41	0	39	0.0015	1	U	K.FDSNEEDSASVFSPSFGLK.Q
9250	1464 - 1482	1031.9654	2061.9163	2061.9167	-0.19	0	105	4.8e-10	1	U	K.FDSNEEDSASVFSPSFGLK.Q
9251	1464 - 1482	1031.9655	2061.9165	2061.9167	-0.099	0	96	5.8e-09	1	U	K.FDSNEEDSASVFSPSFGLK.Q
9252	1464 - 1482	1031.9660	2061.9175	2061.9167	0.39	0	76	3.8e-07	1	U	K.FDSNEEDSASVFSPSFGLK.Q
9253	1464 - 1482	1031.9661	2061.9176	2061.9167	0.42	0	110	1.6e-10	1	U	K.FDSNEEDSASVFSPSFGLK.Q
9254	1464 - 1482	1031.9663	2061.9180	2061.9167	0.63	0	29	0.0066	1	U	K.FDSNEEDSASVFSPSFGLK.Q
9255	1464 - 1482	1031.9665	2061.9184	2061.9167	0.81	0	44	0.00044	1	U	K.FDSNEEDSASVFSPSFGLK.Q
9256	1464 - 1482	1031.9668	2061.9190	2061.9167	1.09	0	92	1.3e-08	1	U	K.FDSNEEDSASVFSPSFGLK.Q
9257	1464 - 1482	688.3137	2061.9193	2061.9167	1.23	0	24	0.037	1	U	K.FDSNEEDSASVFSPSFGLK.Q
9258	1464 - 1482	1031.9673	2061.9201	2061.9167	1.64	0	18	0.032	1	U	K.FDSNEEDSASVFSPSFGLK.Q
9259	1464 - 1482	1031.9682	2061.9218	2061.9167	2.46	0	45	0.00036	1	U	K.FDSNEEDSASVFSPSFGLK.Q
9260	1464 - 1482	1031.9695	2061.9245	2061.9167	3.76	0	60	7e-05	1	U	K.FDSNEEDSASVFSPSFGLK.Q
9542	1464 - 1482	714.9676	2141.8809	2141.8831	-1.02	0	30	0.008	1	U	K.FDSNEEDSASVFSPSFGLK.Q + Phospho (ST)
9543	1464 - 1482	1071.9478	2141.8811	2141.8831	-0.90	0	68	3e-06	1	U	K.FDSNEEDSASVFSPSFGLK.Q + Phospho (ST)
9544	1464 - 1482	1071.9479	2141.8813	2141.8831	-0.82	0	82	1.5e-07	1	U	K.FDSNEEDSASVFSPSFGLK.Q + Phospho (ST)
9545	1464 - 1482	1071.9482	2141.8817	2141.8831	-0.61	0	73	6.8e-07	1	U	K.FDSNEEDSASVFSPSFGLK.Q + Phospho (ST)
9546	1464 - 1482	1071.9482	2141.8818	2141.8831	-0.60	0	78	1.4e-06	1	U	K.FDSNEEDSASVFSPSFGLK.Q + Phospho (ST)
9547	1464 - 1482	1071.9482	2141.8818	2141.8831	-0.57	0	70	3.7e-06	1	U	K.FDSNEEDSASVFSPSFGLK.Q + Phospho (ST)
9549	1464 - 1482	1071.9484	2141.8823	2141.8831	-0.37	0	60	1.2e-05	1	U	K.FDSNEEDSASVFSPSFGLK.Q + Phospho (ST)
9550	1464 - 1482	1071.9485	2141.8824	2141.8831	-0.31	0	79	4.9e-07	1	U	K.FDSNEEDSASVFSPSFGLK.Q + Phospho (ST)
9551	1464 - 1482	1071.9486	2141.8827	2141.8831	-0.15	0	75	6.8e-07	1	U	K.FDSNEEDSASVFSPSFGLK.Q + Phospho (ST)
9553	1464 - 1482	714.9683	2141.8831	2141.8831	0.021	0	49	9.3e-05	1	U	K.FDSNEEDSASVFSPSFGLK.Q + Phospho (ST)
9554	1464 - 1482	714.9685	2141.8836	2141.8831	0.26	0	27	0.0064	1	U	K.FDSNEEDSASVFSPSFGLK.Q + Phospho (ST)
9555	1464 - 1482	714.9688	2141.8845	2141.8831	0.69	0	29	0.0021	1	U	K.FDSNEEDSASVFSPSFGLK.Q + Phospho (ST)
9556	1464 - 1482	714.9692	2141.8859	2141.8831	1.31	0	32	0.011	1	U	K.FDSNEEDSASVFSPSFGLK.Q + Phospho (ST)
871	1483 - 1490	451.7507	901.4868	901.4869	-0.072	1	48	0.001	1	U	K.QTDKVPSK.T
4466	1496 - 1508	456.9330	1367.7771	1367.7773	-0.10	1	18	0.034	1	U	K.KGKPSSDTVPKPK.R
4467	1496 - 1508	456.9330	1367.7773	1367.7773	0.051	1	46	0.00028	1	U	K.KGKPSSDTVPKPK.R
4468	1496 - 1508	684.8960	1367.7775	1367.7773	0.17	1	64	1e-05	1	U	K.KGKPSSDTVPKPK.R
5040	1496 - 1508	483.5888	1447.7447	1447.7436	0.77	1	23	0.043	1	U	K.KGKPSSDTVPKPK.R + Phospho (ST)
3451	1497 - 1508	414.2343	1239.6811	1239.6823	-0.94	0	29	0.0099	1	U	K.GKPSSDTVPKPK.R
3455	1497 - 1508	414.2346	1239.6821	1239.6823	-0.19	0	41	0.0018	1	U	K.GKPSSDTVPKPK.R
3457	1497 - 1508	620.8489	1239.6832	1239.6823	0.77	0	44	0.00016	1	U	K.GKPSSDTVPKPK.R
3458	1497 - 1508	414.2358	1239.6856	1239.6823	2.67	0	32	0.0084	1	U	K.GKPSSDTVPKPK.R
4122	1497 - 1508	440.8899	1319.6478	1319.6486	-0.66	0	24	0.04	1	U	K.GKPSSDTVPKPK.R + Phospho (ST)
8750	1515 - 1532	960.9809	1919.9472	1919.9476	-0.24	1	103	2.8e-10	1	U	K.KVVEAVNSDSDSEFGIPK.K
8751	1515 - 1532	640.9897	1919.9473	1919.9476	-0.19	1	61	1.6e-05	1	U	K.KVVEAVNSDSDSEFGIPK.K
8753	1515 - 1532	960.9813	1919.9480	1919.9476	0.20	1	68	6.5e-07	1	U	K.KVVEAVNSDSDSEFGIPK.K
9042	1515 - 1532	667.6454	1999.9144	1999.9140	0.21	1	31	0.0056	1	U	K.KVVEAVNSDSDSEFGIPK.K + Phospho (ST)
9043	1515 - 1532	1000.9645	1999.9144	1999.9140	0.24	1	84	1.9e-07	1	U	K.KVVEAVNSDSDSEFGIPK.K + Phospho (ST)
9044	1515 - 1532	1000.9646	1999.9146	1999.9140	0.31	1	56	0.00012	1	U	K.KVVEAVNSDSDSEFGIPK.K + Phospho (ST)
9045	1515 - 1532	1000.9646	1999.9146	1999.9140	0.31	1	75	9.9e-07	1	U	K.KVVEAVNSDSDSEFGIPK.K + Phospho (ST)
9046	1515 - 1532	1000.9650	1999.9155	1999.9140	0.76	1	45	0.00056	1	U	K.KVVEAVNSDSDSEFGIPK.K + Phospho (ST)
9047	1515 - 1532	667.6459	1999.9158	1999.9140	0.93	1	50	0.0014	1	U	K.KVVEAVNSDSDSEFGIPK.K + Phospho (ST)
7907	1516 - 1532	598.2916	1791.8531	1791.8527	0.23	0	20	0.044	1	U	K.VVEAVNSDSDSEFGIPK.K
7908	1516 - 1532	896.9338	1791.8531	1791.8527	0.26	0	67	6.8e-07	1	U	K.VVEAVNSDSDSEFGIPK.K
7909	1516 - 1532	896.9355	1791.8565	1791.8527	2.12	0	50	0.00016	1	U	K.VVEAVNSDSDSEFGIPK.K
8463	1516 - 1532	936.9157	1871.8168	1871.8190	-1.16	0	52	2.3e-05	1	U	K.VVEAVNSDSDSEFGIPK.K + Phospho (ST)
8464	1516 - 1532	936.9162	1871.8179	1871.8190	-0.56	0	74	7.2e-07	1	U	K.VVEAVNSDSDSEFGIPK.K + Phospho (ST)
8465	1516 - 1532	936.9169	1871.8192	1871.8190	0.090	0	58	6.1e-06	1	U	K.VVEAVNSDSDSEFGIPK.K + Phospho (ST)
8144	1547 - 1562	606.2566	1815.7478	1815.7537	-3.22	2	28	0.029	1	U	K.RKASGSENEGDYNPGR.K + Phospho (ST)
8145	1547 - 1562	606.2589	1815.7550	1815.7537	0.71	2	47	0.0023	1	U	K.RKASGSENEGDYNPGR.K + Phospho (ST)
6355	1548 - 1562	527.5690	1579.6851	1579.6862	-0.70	1	38	0.013	1	U	R.KASGSENEGDYNPGR.K
6356	1548 - 1562	527.5691	1579.6854	1579.6862	-0.57	1	47	0.00039	1	U	R.KASGSENEGDYNPGR.K
6357	1548 - 1562	790.8502	1579.6858	1579.6862	-0.30	1	35	0.00055	1	U	R.KASGSENEGDYNPGR.K
6359	1548 - 1562	527.5695	1579.6866	1579.6862	0.23	1	33	0.0049	1	U	R.KASGSENEGDYNPGR.K
6914	1548 - 1562	554.2244	1659.6515	1659.6526	-0.63	1	28	0.029	1	U	R.KASGSENEGDYNPGR.K + Phospho (ST)
6915	1548 - 1562	554.2250	1659.6532	1659.6526	0.36	1	35	0.0032	1	U	R.KASGSENEGDYNPGR.K + Phospho (ST)
6916	1548 - 1562	830.8339	1659.6533	1659.6526	0.44	1	40	0.00052	1	U	R.KASGSENEGDYNPGR.K + Phospho (ST)
7528	1548 - 1562	870.8175	1739.6205	1739.6189	0.90	1	39	0.0031	1	U	R.KASGSENEGDYNPGR.K

Query	Start – End	Observed	Mr(expt)	Mr(calc)	ppm	M	Score	Expect	Rank	U	Peptide
											+ 2 Phospho (ST)
7287	1548 – 1563	570.2676	1707.7810	1707.7812	-0.12	2	54	3.4e-05	1	U	R.KASGSENEGDYNPGRK.T
7850	1548 – 1563	596.9232	1787.7477	1787.7475	0.10	2	38	0.00076	1	U	R.KASGSENEGDYNPGRK.T + Phospho (ST)
5069	1549 – 1562	726.8021	1451.5896	1451.5913	-1.15	0	47	0.00091	1	U	K.ASGSENEGDYNPGR.K
5070	1549 – 1562	726.8026	1451.5907	1451.5913	-0.42	0	45	0.00048	1	U	K.ASGSENEGDYNPGR.K
5072	1549 – 1562	726.8030	1451.5914	1451.5913	0.093	0	29	0.016	1	U	K.ASGSENEGDYNPGR.K
5073	1549 – 1562	484.8711	1451.5915	1451.5913	0.16	0	38	0.0034	1	U	K.ASGSENEGDYNPGR.K
5074	1549 – 1562	726.8031	1451.5917	1451.5913	0.26	0	46	8.2e-05	1	U	K.ASGSENEGDYNPGR.K
5075	1549 – 1562	726.8037	1451.5928	1451.5913	1.02	0	27	0.0088	1	U	K.ASGSENEGDYNPGR.K
5076	1549 – 1562	726.8037	1451.5928	1451.5913	1.07	0	32	0.0047	1	U	K.ASGSENEGDYNPGR.K
5926	1549 – 1562	766.7857	1531.5568	1531.5576	-0.50	0	26	0.012	1	U	K.ASGSENEGDYNPGR.K + Phospho (ST)
5927	1549 – 1562	766.7857	1531.5569	1531.5576	-0.46	0	58	2.3e-05	1	U	K.ASGSENEGDYNPGR.K + Phospho (ST)
5928	1549 – 1562	766.7864	1531.5582	1531.5576	0.37	0	49	0.00015	1	U	K.ASGSENEGDYNPGR.K + Phospho (ST)
6358	1549 – 1563	790.8505	1579.6864	1579.6862	0.088	1	80	3.5e-07	1	U	K.ASGSENEGDYNPGRK.T
6917	1549 – 1563	830.8340	1659.6534	1659.6526	0.50	1	49	0.00031	1	U	K.ASGSENEGDYNPGRK.T + Phospho (ST)
11426	1574 – 1599	588.2806	2936.3664	2936.3716	-1.77	1	38	0.0037	1	U	K.KTSFDQDSDVDIFPSDFPTEPPSLPR.T
11427	1574 – 1599	979.7965	2936.3676	2936.3716	-1.39	1	35	0.0052	1	U	K.KTSFDQDSDVDIFPSDFPTEPPSLPR.T
11428	1574 – 1599	979.7966	2936.3678	2936.3716	-1.30	1	31	0.019	1	U	K.KTSFDQDSDVDIFPSDFPTEPPSLPR.T
11432	1574 – 1599	979.7971	2936.3695	2936.3716	-0.73	1	32	0.014	1	U	K.KTSFDQDSDVDIFPSDFPTEPPSLPR.T
11438	1574 – 1599	979.7977	2936.3713	2936.3716	-0.11	1	38	0.01	1	U	K.KTSFDQDSDVDIFPSDFPTEPPSLPR.T
11439	1574 – 1599	588.2816	2936.3716	2936.3716	-0.0027	1	38	0.0022	1	U	K.KTSFDQDSDVDIFPSDFPTEPPSLPR.T
11441	1574 – 1599	979.7980	2936.3722	2936.3716	0.19	1	46	0.00037	1	U	K.KTSFDQDSDVDIFPSDFPTEPPSLPR.T
11157	1575 – 1599	937.0993	2808.2761	2808.2767	-0.20	0	31	0.0074	1	U	K.TSFDQDSDVDIFPSDFPTEPPSLPR.T
11159	1575 – 1599	937.0996	2808.2770	2808.2767	0.11	0	37	0.0049	1	U	K.TSFDQDSDVDIFPSDFPTEPPSLPR.T
9643	1609 – 1626	1086.9145	2171.8144	2171.8154	-0.44	0	23	0.028	1	U	K.YFAESDEEEDDVDFAMFN.-
9644	1609 – 1626	1086.9146	2171.8147	2171.8154	-0.31	0	51	5.6e-05	1	U	K.YFAESDEEEDDVDFAMFN.-
9689	1609 – 1626	1094.9100	2187.8055	2187.8103	-2.18	0	47	4.4e-05	1	U	K.YFAESDEEEDDVDFAMFN.- + Oxidation (M)
9690	1609 – 1626	1094.9104	2187.8062	2187.8103	-1.87	0	34	0.0011	1	U	K.YFAESDEEEDDVDFAMFN.- + Oxidation (M)
9691	1609 – 1626	1094.9116	2187.8087	2187.8103	-0.73	0	48	3.2e-05	1	U	K.YFAESDEEEDDVDFAMFN.- + Oxidation (M)
9692	1609 – 1626	1094.9117	2187.8088	2187.8103	-0.65	0	33	0.0016	1	U	K.YFAESDEEEDDVDFAMFN.- + Oxidation (M)
9694	1609 – 1626	1094.9122	2187.8098	2187.8103	-0.22	0	30	0.0017	1	U	K.YFAESDEEEDDVDFAMFN.- + Oxidation (M)
9695	1609 – 1626	1094.9122	2187.8099	2187.8103	-0.16	0	42	0.00021	1	U	K.YFAESDEEEDDVDFAMFN.- + Oxidation (M)
9696	1609 – 1626	1094.9128	2187.8111	2187.8103	0.36	0	31	0.0042	1	U	K.YFAESDEEEDDVDFAMFN.- + Oxidation (M)

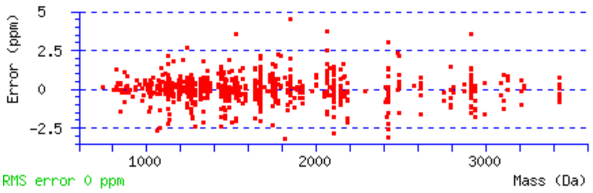

Supplement: Supplementary file 7 — Supplementary Data 4 [file 41467_2023_44089_MOESM7_ESM.pdf]
