# Supplementary material for: ERK2-topoisomerase II regulatory axis is important for gene activation in immediate early genes
Source: Nat Commun. 2023 Dec 14;14:8341. doi: 10.1038/s41467-023-44089-y (PMC10721843; doi:10.1038/s41467-023-44089-y)

MASCOT Search Results

Protein View: Q02880

DNA topoisomerase 2-beta OS=Homo sapiens OX=9606 GN=TOP2B PE=1 SV=3

**Database:**	Uniprot_Proteome_Human
**Score:**	18698
**Monoisotopic mass (M_r):**	184122
**Calculated pI:**	8.14

Sequence similarity is available as **an NCBI BLAST search of Q02880 against nr**.

Search parameters

**MS data file:**	File Name: Z:\SN22\SN221366_deTOP2B_Erk2m_1ul.raw;File Path: ;File Time: 3/1/2023 6:25:31 PM;File Size: 352168572 [Byte]
**Enzyme:**	Trypsin: cuts C-term side of KR unless next residue is P.
**Fixed modifications:**	**Carbamidomethyl (C)**
**Variable modifications:**	**Acetyl (Protein N-term)**, **Oxidation (M)**, **Phospho (ST)**, **Phospho (Y)**

Protein sequence coverage: 65%

Matched peptides shown in ***bold red***.

```
   1 MAKSGGCGAG AGVGGGNGAL TWVTLFDQNN AAKKEESETA NKNDSSKKLS
  51 VERVYQKKTQ LEHILLRPDT YIGSVEPLTQ FMWVYDEDVG MNCREVTFVP
 101 GLYKIFDEIL VNAADNKQRD KNMTCIKVSI DPESNIISIW NNGKGIPVVE
 151 HKVEKVYVPA LIFGQLLTSS NYDDDEKKVT GGRNGYGAKL CNIFSTKFTV
 201 ETACKEYKHS FKQTWMNNMM KTSEAKIKHF DGEDYTCITF QPDLSKFKME
 251 KLDKDIVALM TRRAYDLAGS CRGVKVMFNG KKLPVNGFRS YVDLYVKDKL
 301 DETGVALKVI HELANERWDV CLTLSEKGFQ QISFVNSIAT TKGGRHVDYV
 351 VDQVVGKLIE VVKKKNKAGV SVKPFQVKNH IWVFINCLIE NPTFDSQTKE
 401 NMTLQPKSFG SKCQLSEKFF KAASNCGIVE SILNWVKFKA QTQLNKKCSS
 451 VKYSKIKGIP KLDDANDAGG KHSLECTLIL TEGDSAKSLA VSGLGVIGRD
 501 RYGVFPLRGK ILNVREASHK QIMENAEINN IIKIVGLQYK KSYDDAESLK
 551 TLRYGKIMIM TDQDQDGSHI KGLLINFIHH NWPSLLKHGF LEEFITPIVK
 601 ASKNKQELSF YSIPEFDEWK KHIENQKAWK IKYYKGLGTS TAKEAKEYFA
 651 DMERHRILFR YAGPEDDAAI TLAFSKKKID DRKEWLTNFM EDRRQRRLHG
 701 LPEQFLYGTA TKHLTYNDFI NKELILFSNS DNERSIPSLV DGFKPGQRKV
 751 LFTCFKRNDK REVKVAQLAG SVAEMSAYHH GEQALMMTIV NLAQNFVGSN
 801 NINLLQPIGQ FGTRLHGGKD AASPRYIFTM LSTLARLLFP AVDDNLLKFL
 851 YDDNQRVEPE WYIPIIPMVL INGAEGIGTG WACKLPNYDA REIVNNVRRM
 901 LDGLDPHPML PNYKNFKGTI QELGQNQYAV SGEIFVVDRN TVEITELPVR
 951 TWTQVYKEQV LEPMLNGTDK TPALISDYKE YHTDTTVKFV VKMTEEKLAQ
1001 AEAAGLHKVF KLQTTLTCNS MVLFDHMGCL KKYETVQDIL KEFFDLRLSY
1051 YGLRKEWLVG MLGAESTKLN NQARFILEKI QGKITIENRS KKDLIQMLVQ
1101 RGYESDPVKA WKEAQEKAAE EDETQNQHDD SSSDSGTPSG PDFNYILNMS
1151 LWSLTKEKVE ELIKQRDAKG REVNDLKRKS PSDLWKEDLA AFVEELDKVE
1201 SQEREDVLAG MSGKAIKGKV GKPKVKKLQL EETMPSPYGR RIIPEITAMK
1251 ADASKKLLKK KKGDLDTAAV KVEFDEEFSG APVEGAGEEA LTPSVPINKG
1301 PKPKREKKEP GTRVRKTPTS SGKPSAKKVK KRNPWSDDES KSESDLEETE
1351 PVVIPRDSLL RRAAAERPKY TFDFSEEEDD DADDDDDDNN DLEELKVKAS
1401 PITNDGEDEF VPSDGLDKDE YTFSPGKSKA TPEKSLHDKK SQDFGNLFSF
1451 PSYSQKSEDD SAKFDSNEED SASVFSPSFG LKQTDKVPSK TVAAKKGKPS
1501 SDTVPKPKRA PKQKKVVEAV NSDSDSEFGI PKKTTTPKGK GRGAKKRKAS
1551 GSENEGDYNP GRKTSKTTSK KPKKTSFDQD SDVDIFPSDF PTEPPSLPRT
1601 GRARKEVKYF AESDEEEDDV DFAMFN
```

Unformatted sequence string: **1626 residues** (for pasting into other applications).

Sort by ◉ residue number ○ increasing mass ○ decreasing mass
Show ◉ matched peptides only ○ predicted peptides also

Query	Start – End	Observed	Mr(expt)	Mr(calc)	ppm	M	Score	Expect	Rank	U	Peptide
1666	34 – 42	518.2512	1034.4879	1034.4880	-0.10	1	42	0.0039	1	U	K.KEESETANK.N
5991	34 – 47	783.8662	1565.7178	1565.7169	0.58	2	28	0.0083	1	U	K.KEESETANKNDSSK.K
5992	34 – 47	783.8662	1565.7178	1565.7169	0.58	2	52	5.7e-05	1	U	K.KEESETANKNDSSK.K
6939	34 – 48	565.6116	1693.8129	1693.8118	0.65	3	25	0.017	1	U	K.KEESETANKNDSSKK.L
12986	59 – 94	1086.2690	4341.0467	4341.0545	-1.79	0	41	0.019	1	U	K.TQLEHILLRPDTYIGSVEPLTQFMWVYDEDVGMNCR.E + Oxidation (M)
2646	95 – 104	576.8185	1151.6224	1151.6227	-0.19	0	16	0.035	1		R.EVTFVPGLYK.I
2647	95 – 104	576.8186	1151.6227	1151.6227	0.055	0	16	0.035	1		R.EVTFVPGLYK.I
2653	95 – 104	576.8191	1151.6236	1151.6227	0.84	0	20	0.021	1		R.EVTFVPGLYK.I
4952	105 – 117	487.9240	1460.7501	1460.7511	-0.64	0	30	0.0069	1		K.IFDEILVNAADNK.Q
4953	105 – 117	487.9240	1460.7503	1460.7511	-0.52	0	36	0.0066	1		K.IFDEILVNAADNK.Q
4954	105 – 117	731.3825	1460.7505	1460.7511	-0.39	0	80	7.6e-07	1		K.IFDEILVNAADNK.Q
4955	105 – 117	731.3825	1460.7505	1460.7511	-0.39	0	59	2.6e-05	1		K.IFDEILVNAADNK.Q
4956	105 – 117	487.9241	1460.7506	1460.7511	-0.34	0	31	0.016	1		K.IFDEILVNAADNK.Q
4957	105 – 117	731.3827	1460.7509	1460.7511	-0.12	0	47	0.00021	1		K.IFDEILVNAADNK.Q
4958	105 – 117	731.3828	1460.7509	1460.7511	-0.092	0	70	2.8e-06	1		K.IFDEILVNAADNK.Q
4960	105 – 117	487.9243	1460.7510	1460.7511	-0.049	0	48	0.0014	1		K.IFDEILVNAADNK.Q
4962	105 – 117	487.9243	1460.7511	1460.7511	-0.0075	0	31	0.014	1		K.IFDEILVNAADNK.Q
4963	105 – 117	487.9243	1460.7511	1460.7511	-0.0075	0	40	0.004	1		K.IFDEILVNAADNK.Q

Query	Start – End	Observed	Mr(expt)	Mr(calc)	ppm	M	Score	Expect	Rank	U	Peptide
4964	105 – 117	487.9243	1460.7511	1460.7511	0.013	0	27	0.02	1		K.IFDEILVNAADNK.Q
4965	105 – 117	731.3828	1460.7511	1460.7511	0.031	0	51	0.00013	1		K.IFDEILVNAADNK.Q
4966	105 – 117	487.9244	1460.7512	1460.7511	0.095	0	41	0.00091	1		K.IFDEILVNAADNK.Q
4967	105 – 117	731.3829	1460.7512	1460.7511	0.099	0	68	9.4e-06	1		K.IFDEILVNAADNK.Q
4968	105 – 117	731.3829	1460.7512	1460.7511	0.11	0	68	4.3e-06	1		K.IFDEILVNAADNK.Q
4969	105 – 117	487.9244	1460.7513	1460.7511	0.12	0	30	0.049	1		K.IFDEILVNAADNK.Q
4971	105 – 117	731.3830	1460.7515	1460.7511	0.28	0	68	3.8e-06	1		K.IFDEILVNAADNK.Q
4972	105 – 117	487.9244	1460.7515	1460.7511	0.30	0	28	0.0071	1		K.IFDEILVNAADNK.Q
4973	105 – 117	731.3831	1460.7516	1460.7511	0.36	0	60	1.6e-05	1		K.IFDEILVNAADNK.Q
4974	105 – 117	731.3831	1460.7516	1460.7511	0.37	0	70	2.1e-06	1		K.IFDEILVNAADNK.Q
4975	105 – 117	731.3832	1460.7518	1460.7511	0.47	0	66	3.5e-06	1		K.IFDEILVNAADNK.Q
4976	105 – 117	487.9246	1460.7519	1460.7511	0.55	0	41	0.00079	1		K.IFDEILVNAADNK.Q
4977	105 – 117	731.3833	1460.7521	1460.7511	0.67	0	46	0.00032	1		K.IFDEILVNAADNK.Q
4978	105 – 117	731.3835	1460.7524	1460.7511	0.93	0	66	2.2e-05	1		K.IFDEILVNAADNK.Q
8293	128 – 144	943.4858	1884.9569	1884.9581	-0.62	0	63	3.2e-06	1	U	K.VSIDPESNIISIWNNGK.G
8294	128 – 144	629.3264	1884.9575	1884.9581	-0.31	0	58	6.3e-05	1	U	K.VSIDPESNIISIWNNGK.G
8295	128 – 144	943.4864	1884.9582	1884.9581	0.028	0	39	0.00023	1	U	K.VSIDPESNIISIWNNGK.G
10221	156 – 177	1244.1181	2486.2216	2486.2217	-0.021	0	85	1.2e-07	1	U	K.VYVPALIFGQLLTSSNYDDDEK.K
10222	156 – 177	829.7478	2486.2217	2486.2217	0.0048	0	53	3.2e-05	1	U	K.VYVPALIFGQLLTSSNYDDDEK.K
10224	156 – 177	829.7479	2486.2218	2486.2217	0.065	0	61	7.1e-06	1	U	K.VYVPALIFGQLLTSSNYDDDEK.K
10225	156 – 177	1244.1183	2486.2221	2486.2217	0.18	0	52	3.4e-05	1	U	K.VYVPALIFGQLLTSSNYDDDEK.K
10226	156 – 177	829.7482	2486.2228	2486.2217	0.46	0	32	0.0066	1	U	K.VYVPALIFGQLLTSSNYDDDEK.K
10227	156 – 177	1244.1188	2486.2231	2486.2217	0.57	0	92	2.1e-08	1	U	K.VYVPALIFGQLLTSSNYDDDEK.K
10229	156 – 177	1244.1217	2486.2289	2486.2217	2.91	0	69	1.8e-06	1	U	K.VYVPALIFGQLLTSSNYDDDEK.K
10496	156 – 178	872.4457	2614.3153	2614.3166	-0.52	1	34	0.013	1	U	K.VYVPALIFGQLLTSSNYDDDEKK.V
10498	156 – 178	654.5863	2614.3163	2614.3166	-0.14	1	34	0.0087	1	U	K.VYVPALIFGQLLTSSNYDDDEKK.V
10499	156 – 178	872.4462	2614.3168	2614.3166	0.075	1	47	0.00057	1	U	K.VYVPALIFGQLLTSSNYDDDEKK.V
10500	156 – 178	654.5865	2614.3171	2614.3166	0.16	1	46	0.0028	1	U	K.VYVPALIFGQLLTSSNYDDDEKK.V
10501	156 – 178	872.4463	2614.3171	2614.3166	0.17	1	37	0.0052	1	U	K.VYVPALIFGQLLTSSNYDDDEKK.V
10502	156 – 178	872.4463	2614.3172	2614.3166	0.20	1	41	0.0086	1	U	K.VYVPALIFGQLLTSSNYDDDEKK.V
10503	156 – 178	872.4464	2614.3173	2614.3166	0.26	1	62	8.2e-05	1	U	K.VYVPALIFGQLLTSSNYDDDEKK.V
10506	156 – 178	872.4466	2614.3180	2614.3166	0.53	1	38	0.0049	1	U	K.VYVPALIFGQLLTSSNYDDDEKK.V
10508	156 – 178	1308.1664	2614.3182	2614.3166	0.61	1	54	4.6e-05	1	U	K.VYVPALIFGQLLTSSNYDDDEKK.V
10509	156 – 178	1308.1667	2614.3189	2614.3166	0.88	1	71	7.6e-06	1	U	K.VYVPALIFGQLLTSSNYDDDEKK.V
10512	156 – 178	1308.1690	2614.3233	2614.3166	2.57	1	54	0.00027	1	U	K.VYVPALIFGQLLTSSNYDDDEKK.V
1323	190 – 197	491.7551	981.4956	981.4953	0.25	0	39	0.0013	1		K.LCNIFSTK.F
1141	198 – 205	478.2311	954.4476	954.4481	-0.49	0	25	0.017	1	U	K.FTVETACK.E
2884	213 – 221	592.2565	1182.4985	1182.4984	0.089	0	22	0.015	1	U	K.QTWMNNMMK.T
2994	213 – 221	600.2535	1198.4924	1198.4933	-0.71	0	34	0.0083	1	U	K.QTWMNNMMK.T + Oxidation (M)
2996	213 – 221	600.2539	1198.4933	1198.4933	0.023	0	33	0.0089	1	U	K.QTWMNNMMK.T + Oxidation (M)
2998	213 – 221	600.2541	1198.4937	1198.4933	0.32	0	29	0.014	1	U	K.QTWMNNMMK.T + Oxidation (M)
3097	213 – 221	608.2511	1214.4877	1214.4882	-0.41	0	21	0.015	1	U	K.QTWMNNMMK.T + 2 Oxidation (M)
3098	213 – 221	608.2512	1214.4878	1214.4882	-0.32	0	20	0.035	1	U	K.QTWMNNMMK.T + 2 Oxidation (M)
3100	213 – 221	608.2513	1214.4880	1214.4882	-0.17	0	31	0.01	1	U	K.QTWMNNMMK.T + 2 Oxidation (M)
3101	213 – 221	608.2513	1214.4880	1214.4882	-0.16	0	25	0.033	1	U	K.QTWMNNMMK.T + 2 Oxidation (M)
3102	213 – 221	608.2513	1214.4881	1214.4882	-0.11	0	18	0.028	1	U	K.QTWMNNMMK.T + 2 Oxidation (M)
3105	213 – 221	608.2520	1214.4895	1214.4882	1.09	0	33	0.01	1	U	K.QTWMNNMMK.T + 2 Oxidation (M)
9383	229 – 246	724.9898	2171.9475	2171.9470	0.21	0	33	0.0077	1	U	K.HFDGEDYTCITFQPDLSK.F
3618	252 – 262	637.8600	1273.7055	1273.7064	-0.72	1	40	0.0019	1	U	K.LDKDIVALMTR.R
3621	252 – 262	637.8603	1273.7060	1273.7064	-0.28	1	31	0.027	1	U	K.LDKDIVALMTR.R
3622	252 – 262	637.8603	1273.7060	1273.7064	-0.27	1	46	0.00048	1	U	K.LDKDIVALMTR.R
3623	252 – 262	637.8603	1273.7061	1273.7064	-0.24	1	43	0.00049	1	U	K.LDKDIVALMTR.R
3627	252 – 262	637.8606	1273.7067	1273.7064	0.23	1	62	2.9e-05	1	U	K.LDKDIVALMTR.R
3629	252 – 262	425.5763	1273.7070	1273.7064	0.49	1	31	0.035	1	U	K.LDKDIVALMTR.R
3742	252 – 262	645.8575	1289.7004	1289.7013	-0.67	1	27	0.0079	1	U	K.LDKDIVALMTR.R + Oxidation (M)
3743	252 – 262	645.8576	1289.7007	1289.7013	-0.48	1	54	0.00027	1	U	K.LDKDIVALMTR.R + Oxidation (M)
3745	252 – 262	430.9076	1289.7008	1289.7013	-0.38	1	29	0.013	1	U	K.LDKDIVALMTR.R + Oxidation (M)
3746	252 – 262	645.8577	1289.7008	1289.7013	-0.37	1	34	0.0019	1	U	K.LDKDIVALMTR.R + Oxidation (M)
3748	252 – 262	430.9076	1289.7011	1289.7013	-0.19	1	23	0.043	1	U	K.LDKDIVALMTR.R + Oxidation (M)
3749	252 – 262	430.9077	1289.7012	1289.7013	-0.12	1	35	0.0036	1	U	K.LDKDIVALMTR.R + Oxidation (M)
3750	252 – 262	645.8579	1289.7012	1289.7013	-0.078	1	32	0.0072	1	U	K.LDKDIVALMTR.R + Oxidation (M)
3751	252 – 262	430.9077	1289.7013	1289.7013	-0.029	1	41	0.0042	1	U	K.LDKDIVALMTR.R + Oxidation (M)
3752	252 – 262	645.8580	1289.7014	1289.7013	0.11	1	51	6.6e-05	1	U	K.LDKDIVALMTR.R + Oxidation (M)
3753	252 – 262	430.9078	1289.7015	1289.7013	0.11	1	33	0.0088	1	U	K.LDKDIVALMTR.R + Oxidation (M)
3754	252 – 262	645.8580	1289.7015	1289.7013	0.15	1	49	0.00016	1	U	K.LDKDIVALMTR.R + Oxidation (M)
3755	252 – 262	430.9078	1289.7015	1289.7013	0.16	1	29	0.0054	1	U	K.LDKDIVALMTR.R + Oxidation (M)
3756	252 – 262	645.8581	1289.7016	1289.7013	0.20	1	68	1.5e-06	1	U	K.LDKDIVALMTR.R + Oxidation (M)
3757	252 – 262	430.9080	1289.7021	1289.7013	0.60	1	40	0.0017	1	U	K.LDKDIVALMTR.R + Oxidation (M)
937	255 – 262	459.7572	917.4998	917.5004	-0.65	0	49	0.0002	1	U	K.DIVALMTR.R
1035	255 – 262	467.7546	933.4947	933.4953	-0.68	0	38	0.0091	1	U	K.DIVALMTR.R + Oxidation (M)

Query	Start - End	Observed	Mr(expt)	Mr(calc)	ppm	M	Score	Expect	Rank	U	Peptide
1039	255 - 262	467.7551	933.4956	933.4953	0.28	0	32	0.037	1	U	K.DIVALMTR.R + Oxidation (M)
1040	255 - 262	467.7552	933.4958	933.4953	0.47	0	49	0.00068	1	U	K.DIVALMTR.R + Oxidation (M)
1504	264 - 272	506.7295	1011.4444	1011.4444	0.022	0	30	0.0042	1	U	R.AYDLAGSCR.G
1338	290 - 297	493.7632	985.5119	985.5120	-0.092	0	30	0.021	1	U	R.SYVDLYVK.D
1339	290 - 297	493.7634	985.5122	985.5120	0.15	0	32	0.013	1	U	R.SYVDLYVK.D
1340	290 - 297	493.7635	985.5125	985.5120	0.48	0	43	0.00061	1	U	R.SYVDLYVK.D
2923	298 - 308	594.8269	1187.6392	1187.6398	-0.49	1	48	0.00032	1	U	K.DKLDETGVALK.V
2924	298 - 308	594.8269	1187.6393	1187.6398	-0.36	1	24	0.023	1	U	K.DKLDETGVALK.V
2926	298 - 308	396.8871	1187.6395	1187.6398	-0.21	1	31	0.028	1	U	K.DKLDETGVALK.V
2930	298 - 308	594.8272	1187.6398	1187.6398	0.078	1	29	0.017	1	U	K.DKLDETGVALK.V
1097	300 - 308	473.2661	944.5176	944.5179	-0.28	0	49	0.00057	1	U	K.LDETGVALK.V
2030	309 - 317	360.8641	1079.5704	1079.5723	-1.80	0	26	0.035	1	U	K.VIHELANER.W
2033	309 - 317	360.8646	1079.5719	1079.5723	-0.41	0	26	0.025	1	U	K.VIHELANER.W
2034	309 - 317	360.8646	1079.5719	1079.5723	-0.38	0	36	0.012	1	U	K.VIHELANER.W
2035	309 - 317	540.7933	1079.5720	1079.5723	-0.26	0	47	0.00065	1	U	K.VIHELANER.W
2037	309 - 317	540.7934	1079.5722	1079.5723	-0.13	0	39	0.0016	1	U	K.VIHELANER.W
2038	309 - 317	540.7934	1079.5722	1079.5723	-0.13	0	27	0.037	1	U	K.VIHELANER.W
2041	309 - 317	360.8647	1079.5723	1079.5723	-0.023	0	32	0.019	1	U	K.VIHELANER.W
2043	309 - 317	360.8647	1079.5724	1079.5723	0.032	0	27	0.033	1	U	K.VIHELANER.W
2044	309 - 317	360.8647	1079.5724	1079.5723	0.060	0	29	0.0093	1	U	K.VIHELANER.W
2045	309 - 317	540.7935	1079.5724	1079.5723	0.094	0	50	0.00029	1	U	K.VIHELANER.W
2046	309 - 317	360.8648	1079.5725	1079.5723	0.14	0	32	0.0089	1	U	K.VIHELANER.W
2047	309 - 317	540.7937	1079.5728	1079.5723	0.43	0	45	0.00072	1	U	K.VIHELANER.W
2048	309 - 317	540.7937	1079.5729	1079.5723	0.54	0	26	0.011	1	U	K.VIHELANER.W
3403	318 - 327	625.8079	1249.6013	1249.6013	0.012	0	42	0.00034	1	U	R.WDVCLTLSEK.G
6503	328 - 342	820.9348	1639.8550	1639.8570	-1.21	0	67	1.5e-06	1	U	K.GFQQISFVNSIATTK.G
6505	328 - 342	820.9350	1639.8554	1639.8570	-0.97	0	28	0.0051	1	U	K.GFQQISFVNSIATTK.G
6507	328 - 342	547.6258	1639.8557	1639.8570	-0.80	0	46	0.00013	1	U	K.GFQQISFVNSIATTK.G
6508	328 - 342	820.9351	1639.8557	1639.8570	-0.76	0	14	0.045	1	U	K.GFQQISFVNSIATTK.G
6509	328 - 342	820.9352	1639.8557	1639.8570	-0.75	0	32	0.0035	1	U	K.GFQQISFVNSIATTK.G
6510	328 - 342	820.9352	1639.8558	1639.8570	-0.74	0	56	7.4e-06	1	U	K.GFQQISFVNSIATTK.G
6513	328 - 342	820.9354	1639.8562	1639.8570	-0.46	0	53	0.00042	1	U	K.GFQQISFVNSIATTK.G
6515	328 - 342	820.9358	1639.8570	1639.8570	0.042	0	37	0.0021	1	U	K.GFQQISFVNSIATTK.G
6516	328 - 342	820.9358	1639.8571	1639.8570	0.066	0	30	0.019	1	U	K.GFQQISFVNSIATTK.G
6518	328 - 342	820.9358	1639.8571	1639.8570	0.10	0	58	0.00012	1	U	K.GFQQISFVNSIATTK.G
6519	328 - 342	820.9359	1639.8572	1639.8570	0.13	0	43	0.0006	1	U	K.GFQQISFVNSIATTK.G
6520	328 - 342	547.6264	1639.8572	1639.8570	0.15	0	42	0.0006	1	U	K.GFQQISFVNSIATTK.G
6521	328 - 342	820.9361	1639.8577	1639.8570	0.43	0	54	0.00024	1	U	K.GFQQISFVNSIATTK.G
6522	328 - 342	547.6265	1639.8577	1639.8570	0.44	0	57	9.8e-06	1	U	K.GFQQISFVNSIATTK.G
6524	328 - 342	820.9362	1639.8579	1639.8570	0.57	0	48	0.0012	1	U	K.GFQQISFVNSIATTK.G
6525	328 - 342	820.9363	1639.8581	1639.8570	0.66	0	64	3.4e-05	1	U	K.GFQQISFVNSIATTK.G
6527	328 - 342	547.6267	1639.8582	1639.8570	0.75	0	39	0.00025	1	U	K.GFQQISFVNSIATTK.G
6528	328 - 342	547.6267	1639.8583	1639.8570	0.83	0	30	0.0043	1	U	K.GFQQISFVNSIATTK.G
6529	328 - 342	820.9366	1639.8586	1639.8570	0.97	0	95	2.5e-08	1	U	K.GFQQISFVNSIATTK.G
6531	328 - 342	820.9369	1639.8592	1639.8570	1.33	0	53	0.00014	1	U	K.GFQQISFVNSIATTK.G
4212	346 - 357	679.3584	1356.7022	1356.7038	-1.14	0	36	0.00056	1	U	R.HVDYVVDQVVGK.L
4214	346 - 357	679.3587	1356.7029	1356.7038	-0.65	0	81	7.3e-08	1	U	R.HVDYVVDQVVGK.L
4215	346 - 357	453.2416	1356.7030	1356.7038	-0.59	0	40	0.0077	1	U	R.HVDYVVDQVVGK.L
4216	346 - 357	679.3588	1356.7031	1356.7038	-0.48	0	77	1.2e-07	1	U	R.HVDYVVDQVVGK.L
4217	346 - 357	679.3590	1356.7034	1356.7038	-0.30	0	46	0.00017	1	U	R.HVDYVVDQVVGK.L
4218	346 - 357	679.3590	1356.7034	1356.7038	-0.27	0	75	2.5e-07	1	U	R.HVDYVVDQVVGK.L
4219	346 - 357	679.3590	1356.7035	1356.7038	-0.23	0	47	0.00018	1	U	R.HVDYVVDQVVGK.L
4220	346 - 357	679.3592	1356.7038	1356.7038	0.038	0	77	1.5e-07	1	U	R.HVDYVVDQVVGK.L
4221	346 - 357	679.3592	1356.7039	1356.7038	0.053	0	62	4.7e-06	1	U	R.HVDYVVDQVVGK.L
4222	346 - 357	679.3592	1356.7039	1356.7038	0.068	0	43	0.00032	1	U	R.HVDYVVDQVVGK.L
4223	346 - 357	679.3593	1356.7040	1356.7038	0.16	0	61	2.1e-06	1	U	R.HVDYVVDQVVGK.L
4225	346 - 357	679.3593	1356.7041	1356.7038	0.22	0	57	1.6e-05	1	U	R.HVDYVVDQVVGK.L
4226	346 - 357	679.3594	1356.7042	1356.7038	0.33	0	79	1.5e-07	1	U	R.HVDYVVDQVVGK.L
4227	346 - 357	679.3594	1356.7043	1356.7038	0.35	0	41	0.00028	1	U	R.HVDYVVDQVVGK.L
4228	346 - 357	679.3594	1356.7043	1356.7038	0.36	0	48	0.0001	1	U	R.HVDYVVDQVVGK.L
4229	346 - 357	453.2421	1356.7046	1356.7038	0.59	0	40	0.0046	1	U	R.HVDYVVDQVVGK.L
4230	346 - 357	679.3601	1356.7057	1356.7038	1.41	0	74	2.6e-07	1	U	R.HVDYVVDQVVGK.L
482	358 - 364	414.7811	827.5476	827.5480	-0.47	1	32	0.0045	1	U	K.LIEVVKK.K
2710	368 - 378	580.3447	1158.6749	1158.6761	-1.01	0	47	0.00049	1	U	K.AGVSVKPFQVK.N
2711	368 - 378	387.2324	1158.6755	1158.6761	-0.55	0	30	0.014	1	U	K.AGVSVKPFQVK.N
2716	368 - 378	387.2326	1158.6759	1158.6761	-0.22	0	35	0.0082	1	U	K.AGVSVKPFQVK.N
2721	368 - 378	580.3454	1158.6762	1158.6761	0.074	0	23	0.037	1	U	K.AGVSVKPFQVK.N
2722	368 - 378	580.3454	1158.6763	1158.6761	0.16	0	42	0.0015	1	U	K.AGVSVKPFQVK.N
2727	368 - 378	580.3467	1158.6788	1158.6761	2.32	0	32	0.009	1	U	K.AGVSVKPFQVK.N
10414	379 - 399	859.4246	2575.2520	2575.2529	-0.38	0	52	0.00078	1	U	K.NHIWVFINCLIENPTFDSQTK.E
10415	379 - 399	859.4272	2575.2597	2575.2529	2.62	0	37	0.025	1	U	K.NHIWVFINCLIENPTFDSQTK.E
2010	438 - 446	539.3061	1076.5977	1076.5978	-0.13	1	31	0.0064	1	U	K.FKAQTQLNK.K
378	440 - 446	401.7245	801.4345	801.4344	0.025	0	32	0.028	1	U	K.AQTQLNK.K
1273	462 - 471	488.2225	974.4305	974.4305	-0.0082	0	57	9.8e-05	1	U	K.LDDANDAGGK.H
1274	462 - 471	488.2227	974.4308	974.4305	0.34	0	60	5.3e-05	1	U	K.LDDANDAGGK.H
7515	472 - 487	591.9609	1772.8608	1772.8614	-0.38	0	27	0.022	1	U	K.HSLECTLILTEGDSAK.S
7516	472 - 487	887.4381	1772.8617	1772.8614	0.12	0	112	2.6e-10	1	U	K.HSLECTLILTEGDSAK.S
2432	488 - 499	564.8401	1127.6657	1127.6663	-0.47	0	64	3.8e-06	1	U	K.SLAVSGLGVIGR.D
2434	488 - 499	376.8959	1127.6659	1127.6663	-0.29	0	34	0.0059	1	U	K.SLAVSGLGVIGR.D
2435	488 - 499	564.8403	1127.6660	1127.6663	-0.21	0	56	3.9e-05	1	U	K.SLAVSGLGVIGR.D
2436	488 - 499	564.8403	1127.6660	1127.6663	-0.19	0	42	0.00053	1	U	K.SLAVSGLGVIGR.D
2437	488 - 499	564.8403	1127.6660	1127.6663	-0.19	0	44	0.00015	1	U	K.SLAVSGLGVIGR.D
2438	488 - 499	564.8405	1127.6664	1127.6663	0.15	0	70	1.8e-06	1	U	K.SLAVSGLGVIGR.D
2439	488 - 499	564.8405	1127.6665	1127.6663	0.18	0	33	0.0013	1	U	K.SLAVSGLGVIGR.D
2440	488 - 499	564.8405	1127.6665	1127.6663	0.20	0	65	9.4e-06	1	U	K.SLAVSGLGVIGR.D
2441	488 - 499	564.8405	1127.6665	1127.6663	0.22	0	62	1.1e-05	1	U	K.SLAVSGLGVIGR.D
2442	488 - 499	564.8406	1127.6665	1127.6663	0.26	0	69	4e-06	1	U	K.SLAVSGLGVIGR.D
2443	488 - 499	564.8406	1127.6667	1127.6663	0.38	0	54	5.3e-05	1	U	K.SLAVSGLGVIGR.D
2444	488 - 499	564.8407	1127.6668	1127.6663	0.49	0	67	6.2e-06	1	U	K.SLAVSGLGVIGR.D
2445	488 - 499	564.8407	1127.6669	1127.6663	0.56	0	25	0.0045	1	U	K.SLAVSGLGVIGR.D
2446	488 - 499	564.8408	1127.6669	1127.6663	0.61	0	29	0.002	1	U	K.SLAVSGLGVIGR.D

Query	Start – End	Observed	Mr(expt)	Mr(calc)	ppm	M	Score	Expect	Rank	U	Peptide
2447	488 – 499	564.8408	1127.6670	1127.6663	0.65	0	72	1.6e-06	1	U	K.SLAVSGLGVIGR.D
2448	488 – 499	564.8409	1127.6672	1127.6663	0.88	0	51	5e-05	1	U	K.SLAVSGLGVIGR.D
2375	500 – 508	561.8063	1121.5980	1121.5982	-0.17	1	31	0.027	1	U	R.DRYGVFPLR.G
594	502 – 508	426.2423	850.4700	850.4701	-0.16	0	27	0.037	1		R.YGVFPLR.G
596	502 – 508	426.2430	850.4714	850.4701	1.46	0	28	0.0033	1		R.YGVFPLR.G
5646	521 – 533	510.6045	1528.7916	1528.7919	-0.17	0	51	0.00055	1		K.QIMENAEINNIIK.I
5647	521 – 533	510.6046	1528.7921	1528.7919	0.13	0	25	0.024	1		K.QIMENAEINNIIK.I
5812	521 – 533	773.3999	1544.7852	1544.7868	-1.04	0	62	2.2e-05	1		K.QIMENAEINNIIK.I + Oxidation (M)
5813	521 – 533	773.4005	1544.7864	1544.7868	-0.25	0	64	1.7e-05	1		K.QIMENAEINNIIK.I + Oxidation (M)
5814	521 – 533	773.4006	1544.7866	1544.7868	-0.10	0	48	0.00034	1		K.QIMENAEINNIIK.I + Oxidation (M)
5815	521 – 533	773.4007	1544.7869	1544.7868	0.077	0	57	7.9e-05	1		K.QIMENAEINNIIK.I + Oxidation (M)
5816	521 – 533	773.4012	1544.7878	1544.7868	0.67	0	60	9.1e-05	1		K.QIMENAEINNIIK.I + Oxidation (M)
440	534 – 540	410.7499	819.4852	819.4854	-0.24	0	25	0.022	1		K.IVGLQYK.K
443	534 – 540	410.7500	819.4854	819.4854	-0.024	0	24	0.017	1		K.IVGLQYK.K
445	534 – 540	410.7500	819.4855	819.4854	0.073	0	21	0.048	1		K.IVGLQYK.K
447	534 – 540	410.7501	819.4857	819.4854	0.32	0	38	0.0022	1		K.IVGLQYK.K
2666	541 – 550	578.2799	1154.5453	1154.5455	-0.15	1	32	0.0015	1	U	K.KSYDDAESLK.T
2667	541 – 550	578.2799	1154.5453	1154.5455	-0.15	1	30	0.0014	1	U	K.KSYDDAESLK.T
2669	541 – 550	578.2801	1154.5457	1154.5455	0.16	1	30	0.014	1	U	K.KSYDDAESLK.T
2671	541 – 550	578.2802	1154.5459	1154.5455	0.39	1	30	0.0022	1	U	K.KSYDDAESLK.T
1609	542 – 550	514.2325	1026.4505	1026.4505	-0.057	0	26	0.012	1	U	K.SYDDAESLK.T
1610	542 – 550	514.2326	1026.4506	1026.4505	0.041	0	21	0.043	1	U	K.SYDDAESLK.T
1611	542 – 550	514.2327	1026.4508	1026.4505	0.29	0	23	0.013	1	U	K.SYDDAESLK.T
1612	542 – 550	514.2328	1026.4511	1026.4505	0.53	0	39	0.00037	1	U	K.SYDDAESLK.T
7211	557 – 571	866.4051	1730.7956	1730.7968	-0.64	0	57	2.1e-05	1		K.IMIMTDQDQDGSHIK.G
7215	557 – 571	577.9395	1730.7968	1730.7968	0.023	0	34	0.007	1		K.IMIMTDQDQDGSHIK.G
7216	557 – 571	866.4058	1730.7970	1730.7968	0.14	0	72	1.4e-06	1		K.IMIMTDQDQDGSHIK.G
7219	557 – 571	433.7066	1730.7972	1730.7968	0.24	0	28	0.029	1		K.IMIMTDQDQDGSHIK.G
7220	557 – 571	866.4059	1730.7973	1730.7968	0.31	0	77	1.1e-06	1		K.IMIMTDQDQDGSHIK.G
7222	557 – 571	577.9397	1730.7974	1730.7968	0.37	0	30	0.035	1		K.IMIMTDQDQDGSHIK.G
7223	557 – 571	577.9400	1730.7981	1730.7968	0.77	0	29	0.012	1		K.IMIMTDQDQDGSHIK.G
7229	557 – 571	866.4070	1730.7995	1730.7968	1.59	0	38	0.00067	1		K.IMIMTDQDQDGSHIK.G
7322	557 – 571	874.4023	1746.7900	1746.7917	-0.98	0	75	3.9e-07	1		K.IMIMTDQDQDGSHIK.G + Oxidation (M)
7323	557 – 571	874.4023	1746.7901	1746.7917	-0.91	0	79	1.5e-06	1		K.IMIMTDQDQDGSHIK.G + Oxidation (M)
7326	557 – 571	874.4028	1746.7910	1746.7917	-0.37	0	60	0.00011	1		K.IMIMTDQDQDGSHIK.G + Oxidation (M)
7327	557 – 571	874.4028	1746.7911	1746.7917	-0.33	0	71	1.5e-06	1		K.IMIMTDQDQDGSHIK.G + Oxidation (M)
7328	557 – 571	874.4028	1746.7911	1746.7917	-0.31	0	84	1.7e-07	1		K.IMIMTDQDQDGSHIK.G + Oxidation (M)
7331	557 – 571	583.2711	1746.7916	1746.7917	-0.063	0	47	0.0022	1		K.IMIMTDQDQDGSHIK.G + Oxidation (M)
7332	557 – 571	583.2713	1746.7920	1746.7917	0.18	0	31	0.012	1		K.IMIMTDQDQDGSHIK.G + Oxidation (M)
7334	557 – 571	583.2714	1746.7922	1746.7917	0.31	0	49	0.00062	1		K.IMIMTDQDQDGSHIK.G + Oxidation (M)
7335	557 – 571	583.2714	1746.7923	1746.7917	0.38	0	46	0.0027	1		K.IMIMTDQDQDGSHIK.G + Oxidation (M)
7338	557 – 571	583.2717	1746.7933	1746.7917	0.93	0	33	0.028	1		K.IMIMTDQDQDGSHIK.G + Oxidation (M)
7339	557 – 571	583.2718	1746.7935	1746.7917	1.02	0	49	0.00052	1		K.IMIMTDQDQDGSHIK.G + Oxidation (M)
7441	557 – 571	588.6023	1762.7850	1762.7866	-0.91	0	61	4.2e-05	1		K.IMIMTDQDQDGSHIK.G + 2 Oxidation (M)
7442	557 – 571	588.6023	1762.7852	1762.7866	-0.79	0	24	0.027	1		K.IMIMTDQDQDGSHIK.G + 2 Oxidation (M)
7443	557 – 571	588.6025	1762.7856	1762.7866	-0.57	0	51	0.00028	1		K.IMIMTDQDQDGSHIK.G + 2 Oxidation (M)
7444	557 – 571	588.6025	1762.7857	1762.7866	-0.52	0	36	0.0032	1		K.IMIMTDQDQDGSHIK.G + 2 Oxidation (M)
7446	557 – 571	588.6026	1762.7858	1762.7866	-0.44	0	58	5.9e-05	1		K.IMIMTDQDQDGSHIK.G + 2 Oxidation (M)
7448	557 – 571	882.4002	1762.7859	1762.7866	-0.39	0	76	2.8e-06	1		K.IMIMTDQDQDGSHIK.G + 2 Oxidation (M)
7450	557 – 571	882.4004	1762.7863	1762.7866	-0.15	0	61	9.2e-05	1		K.IMIMTDQDQDGSHIK.G + 2 Oxidation (M)
7451	557 – 571	588.6028	1762.7864	1762.7866	-0.096	0	41	0.0014	1		K.IMIMTDQDQDGSHIK.G + 2 Oxidation (M)
7452	557 – 571	882.4005	1762.7865	1762.7866	-0.058	0	60	0.00011	1		K.IMIMTDQDQDGSHIK.G + 2 Oxidation (M)
7453	557 – 571	588.6029	1762.7868	1762.7866	0.11	0	35	0.0019	1		K.IMIMTDQDQDGSHIK.G + 2 Oxidation (M)
7454	557 – 571	588.6029	1762.7868	1762.7866	0.13	0	26	0.038	1		K.IMIMTDQDQDGSHIK.G + 2 Oxidation (M)
7455	557 – 571	588.6029	1762.7868	1762.7866	0.14	0	57	0.00022	1		K.IMIMTDQDQDGSHIK.G + 2 Oxidation (M)
7456	557 – 571	882.4007	1762.7868	1762.7866	0.15	0	58	0.00019	1		K.IMIMTDQDQDGSHIK.G + 2 Oxidation (M)
7457	557 – 571	588.6030	1762.7872	1762.7866	0.33	0	33	0.0044	1		K.IMIMTDQDQDGSHIK.G + 2 Oxidation (M)
7459	557 – 571	882.4011	1762.7877	1762.7866	0.61	0	61	8.4e-05	1		K.IMIMTDQDQDGSHIK.G + 2 Oxidation (M)
7460	557 – 571	588.6034	1762.7883	1762.7866	0.96	0	46	0.0003	1		K.IMIMTDQDQDGSHIK.G + 2 Oxidation (M)
8370	572 – 587	634.6956	1901.0651	1901.0676	-1.28	0	22	0.015	1	U	K.GLLINFIHHNWPSLLK.H
8372	572 – 587	634.6963	1901.0671	1901.0676	-0.22	0	22	0.015	1	U	K.GLLINFIHHNWPSLLK.H
8373	572 – 587	634.6964	1901.0673	1901.0676	-0.11	0	36	0.0036	1	U	K.GLLINFIHHNWPSLLK.H
8374	572 – 587	634.6964	1901.0674	1901.0676	-0.078	0	21	0.024	1	U	K.GLLINFIHHNWPSLLK.H

Query	Start – End	Observed	Mr(expt)	Mr(calc)	ppm	M	Score	Expect	Rank	U	Peptide
8377	572 – 587	951.5412	1901.0678	1901.0676	0.11	0	42	0.00012	1	U	K.GLLINFIHHNWPSLLK.H
8380	572 – 587	634.6968	1901.0685	1901.0676	0.47	0	27	0.0045	1	U	K.GLLINFIHHNWPSLLK.H
8381	572 – 587	951.5416	1901.0686	1901.0676	0.57	0	64	1.5e-06	1	U	K.GLLINFIHHNWPSLLK.H
5648	588 – 600	510.6162	1528.8268	1528.8290	-1.39	0	32	0.0046	1	U	K.HGFLEEFITPIVK.A
5649	588 – 600	765.4212	1528.8279	1528.8290	-0.69	0	45	0.00018	1	U	K.HGFLEEFITPIVK.A
5650	588 – 600	510.6167	1528.8282	1528.8290	-0.53	0	28	0.011	1	U	K.HGFLEEFITPIVK.A
5651	588 – 600	765.4214	1528.8282	1528.8290	-0.52	0	70	7.8e-07	1	U	K.HGFLEEFITPIVK.A
5652	588 – 600	510.6167	1528.8283	1528.8290	-0.43	0	25	0.049	1	U	K.HGFLEEFITPIVK.A
5653	588 – 600	765.4214	1528.8283	1528.8290	-0.40	0	48	3e-05	1	U	K.HGFLEEFITPIVK.A
5654	588 – 600	765.4215	1528.8285	1528.8290	-0.31	0	73	1.5e-07	1	U	K.HGFLEEFITPIVK.A
5655	588 – 600	510.6168	1528.8286	1528.8290	-0.26	0	42	0.0017	1	U	K.HGFLEEFITPIVK.A
5656	588 – 600	765.4216	1528.8286	1528.8290	-0.22	0	58	3.5e-06	1	U	K.HGFLEEFITPIVK.A
5657	588 – 600	765.4216	1528.8286	1528.8290	-0.22	0	87	2.7e-08	1	U	K.HGFLEEFITPIVK.A
5658	588 – 600	765.4216	1528.8286	1528.8290	-0.21	0	80	1.5e-07	1	U	K.HGFLEEFITPIVK.A
5660	588 – 600	510.6169	1528.8289	1528.8290	-0.041	0	33	0.0033	1	U	K.HGFLEEFITPIVK.A
5661	588 – 600	510.6169	1528.8290	1528.8290	-0.0013	0	43	0.0015	1	U	K.HGFLEEFITPIVK.A
5662	588 – 600	765.4218	1528.8290	1528.8290	0.029	0	69	7.2e-07	1	U	K.HGFLEEFITPIVK.A
5663	588 – 600	765.4218	1528.8290	1528.8290	0.029	0	69	3.4e-07	1	U	K.HGFLEEFITPIVK.A
5664	588 – 600	765.4218	1528.8290	1528.8290	0.042	0	90	6.8e-09	1	U	K.HGFLEEFITPIVK.A
5665	588 – 600	765.4218	1528.8291	1528.8290	0.081	0	68	4.4e-07	1	U	K.HGFLEEFITPIVK.A
5666	588 – 600	510.6170	1528.8291	1528.8290	0.097	0	47	0.00049	1	U	K.HGFLEEFITPIVK.A
5667	588 – 600	510.6170	1528.8291	1528.8290	0.097	0	46	0.00086	1	U	K.HGFLEEFITPIVK.A
5668	588 – 600	510.6170	1528.8291	1528.8290	0.097	0	40	0.0013	1	U	K.HGFLEEFITPIVK.A
5669	588 – 600	765.4218	1528.8291	1528.8290	0.12	0	70	4.3e-07	1	U	K.HGFLEEFITPIVK.A
5670	588 – 600	510.6170	1528.8292	1528.8290	0.18	0	48	0.00044	1	U	K.HGFLEEFITPIVK.A
5671	588 – 600	510.6170	1528.8293	1528.8290	0.19	0	36	0.012	1	U	K.HGFLEEFITPIVK.A
5672	588 – 600	765.4220	1528.8294	1528.8290	0.26	0	76	2.5e-07	1	U	K.HGFLEEFITPIVK.A
5673	588 – 600	765.4220	1528.8294	1528.8290	0.26	0	54	9.3e-06	1	U	K.HGFLEEFITPIVK.A
5674	588 – 600	765.4220	1528.8294	1528.8290	0.29	0	83	1.6e-08	1	U	K.HGFLEEFITPIVK.A
5675	588 – 600	510.6171	1528.8294	1528.8290	0.29	0	46	0.0014	1	U	K.HGFLEEFITPIVK.A
5676	588 – 600	510.6171	1528.8294	1528.8290	0.31	0	33	0.0057	1	U	K.HGFLEEFITPIVK.A
5677	588 – 600	765.4220	1528.8295	1528.8290	0.36	0	87	1.5e-08	1	U	K.HGFLEEFITPIVK.A
5678	588 – 600	765.4222	1528.8299	1528.8290	0.59	0	71	6.9e-07	1	U	K.HGFLEEFITPIVK.A
9328	604 – 620	1080.5162	2159.0178	2159.0211	-1.52	1	126	2.3e-11	1	U	K.NKQELSFYSIPEFDEWK.K
9329	604 – 620	720.6813	2159.0221	2159.0211	0.46	1	26	0.045	1	U	K.NKQELSFYSIPEFDEWK.K
9727	604 – 621	763.3795	2287.1168	2287.1161	0.32	2	41	0.00066	1	U	K.NKQELSFYSIPEFDEWKK.H
8477	606 – 620	959.4484	1916.8823	1916.8832	-0.46	0	64	1.6e-05	1	U	K.QELSFYSIPEFDEWK.K
8478	606 – 620	959.4486	1916.8826	1916.8832	-0.33	0	60	2.9e-05	1	U	K.QELSFYSIPEFDEWK.K
8481	606 – 620	959.4493	1916.8840	1916.8832	0.43	0	43	0.00078	1	U	K.QELSFYSIPEFDEWK.K
8948	606 – 621	1023.4962	2044.9779	2044.9782	-0.13	1	52	4.3e-05	1	U	K.QELSFYSIPEFDEWKK.H
8950	606 – 621	682.6668	2044.9787	2044.9782	0.25	1	36	0.0021	1	U	K.QELSFYSIPEFDEWKK.H
1859	647 – 654	530.7239	1059.4332	1059.4331	0.044	0	16	0.04	1	U	K.EYFADMER.H
1860	647 – 654	530.7239	1059.4333	1059.4331	0.14	0	15	0.039	1	U	K.EYFADMER.H
1861	647 – 654	530.7242	1059.4339	1059.4331	0.72	0	32	0.006	1	U	K.EYFADMER.H
1998	647 – 654	538.7210	1075.4273	1075.4280	-0.64	0	30	0.0088	1	U	K.EYFADMER.H + Oxidation (M)
1999	647 – 654	538.7213	1075.4280	1075.4280	-0.011	0	33	0.013	1	U	K.EYFADMER.H + Oxidation (M)
2001	647 – 654	538.7215	1075.4285	1075.4280	0.44	0	22	0.035	1	U	K.EYFADMER.H + Oxidation (M)
6706	661 – 676	834.9088	1667.8030	1667.8042	-0.75	0	64	2.5e-06	1	U	R.YAGPEDDAAITLAFSK.K
6707	661 – 676	834.9088	1667.8031	1667.8042	-0.68	0	80	2.1e-07	1	U	R.YAGPEDDAAITLAFSK.K
6708	661 – 676	834.9090	1667.8034	1667.8042	-0.51	0	78	7.5e-08	1	U	R.YAGPEDDAAITLAFSK.K
6709	661 – 676	834.9090	1667.8035	1667.8042	-0.45	0	92	3.6e-09	1	U	R.YAGPEDDAAITLAFSK.K
6710	661 – 676	834.9091	1667.8036	1667.8042	-0.37	0	90	1.4e-08	1	U	R.YAGPEDDAAITLAFSK.K
6711	661 – 676	834.9092	1667.8039	1667.8042	-0.21	0	67	2.7e-06	1	U	R.YAGPEDDAAITLAFSK.K
6712	661 – 676	834.9092	1667.8039	1667.8042	-0.19	0	92	1.7e-08	1	U	R.YAGPEDDAAITLAFSK.K
6713	661 – 676	834.9093	1667.8041	1667.8042	-0.094	0	71	6.3e-07	1	U	R.YAGPEDDAAITLAFSK.K
6714	661 – 676	834.9093	1667.8041	1667.8042	-0.082	0	70	7.7e-07	1	U	R.YAGPEDDAAITLAFSK.K
6715	661 – 676	834.9094	1667.8042	1667.8042	-0.010	0	75	1e-07	1	U	R.YAGPEDDAAITLAFSK.K
6716	661 – 676	834.9094	1667.8043	1667.8042	0.062	0	61	5e-06	1	U	R.YAGPEDDAAITLAFSK.K
6717	661 – 676	834.9095	1667.8044	1667.8042	0.074	0	59	1.3e-05	1	U	R.YAGPEDDAAITLAFSK.K
6718	661 – 676	834.9095	1667.8045	1667.8042	0.13	0	73	1.6e-07	1	U	R.YAGPEDDAAITLAFSK.K
6720	661 – 676	834.9098	1667.8049	1667.8042	0.42	0	65	3.2e-06	1	U	R.YAGPEDDAAITLAFSK.K
6721	661 – 676	834.9098	1667.8051	1667.8042	0.51	0	73	4.7e-07	1	U	R.YAGPEDDAAITLAFSK.K
6722	661 – 676	834.9099	1667.8052	1667.8042	0.57	0	47	4.4e-05	1	U	R.YAGPEDDAAITLAFSK.K
6723	661 – 676	556.9424	1667.8055	1667.8042	0.73	0	51	0.00028	1	U	R.YAGPEDDAAITLAFSK.K
6724	661 – 676	834.9101	1667.8057	1667.8042	0.87	0	79	8.8e-08	1	U	R.YAGPEDDAAITLAFSK.K
6725	661 – 676	834.9102	1667.8059	1667.8042	1.02	0	73	1.2e-06	1	U	R.YAGPEDDAAITLAFSK.K
5026	683 – 693	490.2345	1467.6817	1467.6816	0.052	1	27	0.016	1		R.KEWLTNFMEDR.R
5149	683 – 693	742.8450	1483.6755	1483.6765	-0.71	1	41	0.00016	1		R.KEWLTNFMEDR.R + Oxidation (M)
5151	683 – 693	495.5660	1483.6761	1483.6765	-0.33	1	29	0.012	1		R.KEWLTNFMEDR.R + Oxidation (M)
5152	683 – 693	495.5660	1483.6762	1483.6765	-0.21	1	25	0.017	1		R.KEWLTNFMEDR.R + Oxidation (M)
5154	683 – 693	742.8455	1483.6764	1483.6765	-0.078	1	43	0.00043	1		R.KEWLTNFMEDR.R + Oxidation (M)
5158	683 – 693	495.5663	1483.6770	1483.6765	0.30	1	31	0.0024	1		R.KEWLTNFMEDR.R + Oxidation (M)
6429	683 – 694	542.2681	1623.7824	1623.7827	-0.20	2	31	0.019	1		R.KEWLTNFMEDRR.Q
4120	684 – 693	670.7978	1339.5811	1339.5867	-4.17	0	35	0.0006	1		K.EWLTNFMEDR.R
4121	684 – 693	670.8006	1339.5867	1339.5867	0.013	0	50	0.00021	1		K.EWLTNFMEDR.R
4204	684 – 693	678.7978	1355.5811	1355.5816	-0.37	0	21	0.018	1		K.EWLTNFMEDR.R + Oxidation (M)
4205	684 – 693	678.7979	1355.5813	1355.5816	-0.19	0	26	0.043	1		K.EWLTNFMEDR.R + Oxidation (M)
7962	697 – 712	610.9992	1829.9759	1829.9788	-1.61	1	29	0.0054	1	U	R.RLHGLPEQFLYGTATK.H
7963	697 – 712	610.9996	1829.9769	1829.9788	-1.02	1	39	0.00086	1	U	R.RLHGLPEQFLYGTATK.H
7964	697 – 712	610.9998	1829.9775	1829.9788	-0.74	1	25	0.039	1	U	R.RLHGLPEQFLYGTATK.H
7966	697 – 712	611.0000	1829.9783	1829.9788	-0.30	1	29	0.0034	1	U	R.RLHGLPEQFLYGTATK.H
7967	697 – 712	611.0000	1829.9783	1829.9788	-0.30	1	56	2.9e-05	1	U	R.RLHGLPEQFLYGTATK.H
7968	697 – 712	915.9965	1829.9784	1829.9788	-0.21	1	83	1.5e-08	1	U	R.RLHGLPEQFLYGTATK.H

Query	Start – End	Observed	Mr(expt)	Mr(calc)	ppm	M	Score	Expect	Rank	U	Peptide
7969	697 – 712	611.0001	1829.9785	1829.9788	-0.18	1	21	0.011	1	U	R.RLHGLPEQFLYGTATK.H
7970	697 – 712	611.0003	1829.9791	1829.9788	0.15	1	46	0.00049	1	U	R.RLHGLPEQFLYGTATK.H
7972	697 – 712	611.0004	1829.9793	1829.9788	0.29	1	54	0.00014	1	U	R.RLHGLPEQFLYGTATK.H
7973	697 – 712	458.5021	1829.9794	1829.9788	0.31	1	49	0.00034	1	U	R.RLHGLPEQFLYGTATK.H
7976	697 – 712	611.0006	1829.9798	1829.9788	0.56	1	32	0.0061	1	U	R.RLHGLPEQFLYGTATK.H
7977	697 – 712	611.0006	1829.9801	1829.9788	0.72	1	28	0.012	1	U	R.RLHGLPEQFLYGTATK.H
6759	698 – 712	837.9450	1673.8755	1673.8777	-1.30	0	21	0.012	1	U	R.LHGLPEQFLYGTATK.H
6760	698 – 712	837.9452	1673.8759	1673.8777	-1.08	0	74	1.9e-07	1	U	R.LHGLPEQFLYGTATK.H
6761	698 – 712	558.9660	1673.8763	1673.8777	-0.84	0	39	0.0015	1	U	R.LHGLPEQFLYGTATK.H
6762	698 – 712	558.9661	1673.8764	1673.8777	-0.81	0	24	0.017	1	U	R.LHGLPEQFLYGTATK.H
6763	698 – 712	837.9455	1673.8765	1673.8777	-0.72	0	29	0.0018	1	U	R.LHGLPEQFLYGTATK.H
6764	698 – 712	837.9456	1673.8767	1673.8777	-0.58	0	33	0.00087	1	U	R.LHGLPEQFLYGTATK.H
6765	698 – 712	558.9662	1673.8769	1673.8777	-0.48	0	44	0.00096	1	U	R.LHGLPEQFLYGTATK.H
6766	698 – 712	837.9459	1673.8772	1673.8777	-0.29	0	33	0.002	1	U	R.LHGLPEQFLYGTATK.H
6768	698 – 712	837.9460	1673.8774	1673.8777	-0.15	0	37	0.00036	1	U	R.LHGLPEQFLYGTATK.H
6770	698 – 712	558.9664	1673.8775	1673.8777	-0.11	0	26	0.026	1	U	R.LHGLPEQFLYGTATK.H
6771	698 – 712	558.9665	1673.8776	1673.8777	-0.089	0	34	0.00093	1	U	R.LHGLPEQFLYGTATK.H
6772	698 – 712	837.9461	1673.8776	1673.8777	-0.079	0	33	0.00085	1	U	R.LHGLPEQFLYGTATK.H
6773	698 – 712	558.9665	1673.8776	1673.8777	-0.071	0	30	0.0076	1	U	R.LHGLPEQFLYGTATK.H
6774	698 – 712	837.9461	1673.8776	1673.8777	-0.032	0	25	0.0065	1	U	R.LHGLPEQFLYGTATK.H
6775	698 – 712	558.9665	1673.8778	1673.8777	0.036	0	32	0.0035	1	U	R.LHGLPEQFLYGTATK.H
6776	698 – 712	837.9462	1673.8779	1673.8777	0.11	0	54	9.1e-06	1	U	R.LHGLPEQFLYGTATK.H
6777	698 – 712	558.9666	1673.8779	1673.8777	0.13	0	23	0.035	1	U	R.LHGLPEQFLYGTATK.H
6778	698 – 712	419.4768	1673.8779	1673.8777	0.14	0	39	0.0084	1	U	R.LHGLPEQFLYGTATK.H
6779	698 – 712	837.9462	1673.8779	1673.8777	0.15	0	30	0.0023	1	U	R.LHGLPEQFLYGTATK.H
6780	698 – 712	558.9666	1673.8781	1673.8777	0.22	0	38	0.0045	1	U	R.LHGLPEQFLYGTATK.H
6781	698 – 712	558.9666	1673.8781	1673.8777	0.23	0	38	0.0032	1	U	R.LHGLPEQFLYGTATK.H
6782	698 – 712	558.9666	1673.8781	1673.8777	0.25	0	31	0.0062	1	U	R.LHGLPEQFLYGTATK.H
6783	698 – 712	837.9464	1673.8782	1673.8777	0.28	0	66	7.9e-07	1	U	R.LHGLPEQFLYGTATK.H
6784	698 – 712	558.9667	1673.8782	1673.8777	0.31	0	38	0.0055	1	U	R.LHGLPEQFLYGTATK.H
6785	698 – 712	558.9668	1673.8785	1673.8777	0.45	0	26	0.027	1	U	R.LHGLPEQFLYGTATK.H
6786	698 – 712	558.9668	1673.8785	1673.8777	0.48	0	30	0.022	1	U	R.LHGLPEQFLYGTATK.H
6788	698 – 712	558.9669	1673.8788	1673.8777	0.68	0	38	0.0029	1	U	R.LHGLPEQFLYGTATK.H
6790	698 – 712	837.9469	1673.8793	1673.8777	0.95	0	20	0.012	1	U	R.LHGLPEQFLYGTATK.H
6792	698 – 712	837.9470	1673.8795	1673.8777	1.10	0	23	0.0076	1	U	R.LHGLPEQFLYGTATK.H
6793	698 – 712	558.9672	1673.8797	1673.8777	1.20	0	48	0.00078	1	U	R.LHGLPEQFLYGTATK.H
6794	698 – 712	558.9674	1673.8805	1673.8777	1.67	0	26	0.028	1	U	R.LHGLPEQFLYGTATK.H
3510	713 – 722	632.8191	1263.6237	1263.6248	-0.84	0	24	0.011	1	U	K.HLTYNDFINK.E
3511	713 – 722	632.8192	1263.6239	1263.6248	-0.67	0	16	0.034	1	U	K.HLTYNDFINK.E
3512	713 – 722	632.8193	1263.6240	1263.6248	-0.64	0	39	0.0032	1	U	K.HLTYNDFINK.E
3513	713 – 722	632.8193	1263.6240	1263.6248	-0.59	0	36	0.0012	1	U	K.HLTYNDFINK.E
3515	713 – 722	632.8194	1263.6243	1263.6248	-0.38	0	35	0.0013	1	U	K.HLTYNDFINK.E
3516	713 – 722	632.8194	1263.6243	1263.6248	-0.37	0	34	0.0015	1	U	K.HLTYNDFINK.E
3517	713 – 722	632.8194	1263.6243	1263.6248	-0.37	0	27	0.0059	1	U	K.HLTYNDFINK.E
3518	713 – 722	632.8196	1263.6245	1263.6248	-0.18	0	33	0.0019	1	U	K.HLTYNDFINK.E
3520	713 – 722	632.8196	1263.6246	1263.6248	-0.11	0	40	0.0023	1	U	K.HLTYNDFINK.E
3521	713 – 722	632.8196	1263.6246	1263.6248	-0.097	0	36	0.018	1	U	K.HLTYNDFINK.E
3522	713 – 722	632.8197	1263.6248	1263.6248	0.013	0	44	0.00044	1	U	K.HLTYNDFINK.E
3523	713 – 722	632.8197	1263.6248	1263.6248	0.029	0	34	0.0016	1	U	K.HLTYNDFINK.E
3524	713 – 722	632.8197	1263.6248	1263.6248	0.029	0	24	0.012	1	U	K.HLTYNDFINK.E
3525	713 – 722	632.8197	1263.6248	1263.6248	0.045	0	23	0.026	1	U	K.HLTYNDFINK.E
3527	713 – 722	632.8197	1263.6249	1263.6248	0.11	0	16	0.033	1	U	K.HLTYNDFINK.E
3528	713 – 722	632.8197	1263.6249	1263.6248	0.11	0	24	0.028	1	U	K.HLTYNDFINK.E
3529	713 – 722	632.8197	1263.6249	1263.6248	0.12	0	26	0.011	1	U	K.HLTYNDFINK.E
3530	713 – 722	632.8198	1263.6250	1263.6248	0.16	0	34	0.0013	1	U	K.HLTYNDFINK.E
3531	713 – 722	422.2156	1263.6250	1263.6248	0.18	0	36	0.0025	1	U	K.HLTYNDFINK.E
3532	713 – 722	422.2157	1263.6253	1263.6248	0.42	0	21	0.039	1	U	K.HLTYNDFINK.E
3533	713 – 722	422.2157	1263.6253	1263.6248	0.42	0	27	0.013	1	U	K.HLTYNDFINK.E
3534	713 – 722	632.8199	1263.6253	1263.6248	0.42	0	30	0.013	1	U	K.HLTYNDFINK.E
3535	713 – 722	632.8200	1263.6254	1263.6248	0.50	0	39	0.0021	1	U	K.HLTYNDFINK.E
3536	713 – 722	632.8200	1263.6255	1263.6248	0.58	0	31	0.0018	1	U	K.HLTYNDFINK.E
3537	713 – 722	632.8200	1263.6255	1263.6248	0.60	0	25	0.019	1	U	K.HLTYNDFINK.E
3539	713 – 722	632.8202	1263.6258	1263.6248	0.80	0	28	0.013	1	U	K.HLTYNDFINK.E
3540	713 – 722	632.8204	1263.6262	1263.6248	1.17	0	30	0.028	1	U	K.HLTYNDFINK.E
4733	723 – 734	718.8534	1435.6923	1435.6943	-1.39	0	32	0.0027	1		K.ELILFSNSDNER.S
4734	723 – 734	718.8537	1435.6929	1435.6943	-0.96	0	45	0.003	1		K.ELILFSNSDNER.S
4735	723 – 734	718.8538	1435.6931	1435.6943	-0.80	0	36	0.0052	1		K.ELILFSNSDNER.S
4736	723 – 734	718.8540	1435.6935	1435.6943	-0.53	0	40	0.0009	1		K.ELILFSNSDNER.S
4737	723 – 734	718.8540	1435.6935	1435.6943	-0.53	0	52	5e-05	1		K.ELILFSNSDNER.S
4739	723 – 734	718.8541	1435.6936	1435.6943	-0.46	0	32	0.0019	1		K.ELILFSNSDNER.S
4740	723 – 734	718.8541	1435.6936	1435.6943	-0.45	0	47	0.00046	1		K.ELILFSNSDNER.S
4741	723 – 734	718.8542	1435.6937	1435.6943	-0.38	0	28	0.008	1		K.ELILFSNSDNER.S
4742	723 – 734	718.8542	1435.6939	1435.6943	-0.29	0	24	0.0079	1		K.ELILFSNSDNER.S
4743	723 – 734	718.8543	1435.6940	1435.6943	-0.20	0	52	9.1e-05	1		K.ELILFSNSDNER.S
4744	723 – 734	718.8544	1435.6942	1435.6943	-0.085	0	36	0.0054	1		K.ELILFSNSDNER.S
4746	723 – 734	718.8544	1435.6942	1435.6943	-0.029	0	39	0.0042	1		K.ELILFSNSDNER.S
4747	723 – 734	718.8544	1435.6942	1435.6943	-0.029	0	29	0.04	1		K.ELILFSNSDNER.S
4748	723 – 734	718.8544	1435.6943	1435.6943	0.040	0	40	0.0025	1		K.ELILFSNSDNER.S
4749	723 – 734	718.8545	1435.6944	1435.6943	0.068	0	37	0.0059	1		K.ELILFSNSDNER.S
4750	723 – 734	718.8546	1435.6946	1435.6943	0.19	0	31	0.023	1		K.ELILFSNSDNER.S
4751	723 – 734	718.8546	1435.6946	1435.6943	0.21	0	31	0.019	1		K.ELILFSNSDNER.S
4752	723 – 734	718.8547	1435.6949	1435.6943	0.40	0	34	0.037	1		K.ELILFSNSDNER.S
4753	723 – 734	718.8549	1435.6952	1435.6943	0.64	0	47	0.00099	1		K.ELILFSNSDNER.S
4754	723 – 734	718.8549	1435.6953	1435.6943	0.72	0	24	0.035	1		K.ELILFSNSDNER.S
4755	723 – 734	718.8550	1435.6955	1435.6943	0.82	0	30	0.028	1		K.ELILFSNSDNER.S
4756	723 – 734	718.8553	1435.6961	1435.6943	1.28	0	36	0.0089	1		K.ELILFSNSDNER.S
4757	723 – 734	718.8554	1435.6962	1435.6943	1.34	0	44	0.00047	1		K.ELILFSNSDNER.S
4758	723 – 734	718.8554	1435.6963	1435.6943	1.41	0	27	0.0076	1		K.ELILFSNSDNER.S
5301	735 – 748	750.9107	1499.8069	1499.8096	-1.82	0	40	0.0002	1	U	R.SIPSLVDGFKPGQR.K
5302	735 – 748	750.9110	1499.8073	1499.8096	-1.52	0	39	0.0014	1	U	R.SIPSLVDGFKPGQR.K
5304	735 – 748	750.9115	1499.8084	1499.8096	-0.84	0	15	0.043	1	U	R.SIPSLVDGFKPGQR.K
5316	735 – 748	750.9121	1499.8097	1499.8096	0.065	0	54	8.2e-05	1	U	R.SIPSLVDGFKPGQR.K
5319	735 – 748	750.9123	1499.8101	1499.8096	0.30	0	32	0.0016	1	U	R.SIPSLVDGFKPGQR.K

Query	Start - End	Observed	Mr(expt)	Mr(calc)	ppm	M	Score	Expect	Rank	U	Peptide
5324	735 - 748	750.9130	1499.8114	1499.8096	1.17	0	14	0.045	1	U	R.SIPSLVDGFKPGQR.K
5325	735 - 748	750.9130	1499.8115	1499.8096	1.28	0	46	5.1e-05	1	U	R.SIPSLVDGFKPGQR.K
3938	826 - 836	658.3564	1314.6983	1314.7006	-1.76	0	33	0.0068	1	U	R.YIFTMLSTLAR.L
3939	826 - 836	658.3574	1314.7002	1314.7006	-0.27	0	38	0.00032	1	U	R.YIFTMLSTLAR.L
3940	826 - 836	658.3576	1314.7006	1314.7006	-0.0068	0	35	0.0011	1	U	R.YIFTMLSTLAR.L
3941	826 - 836	658.3576	1314.7006	1314.7006	0.024	0	50	7.4e-05	1	U	R.YIFTMLSTLAR.L
3942	826 - 836	658.3576	1314.7007	1314.7006	0.100	0	32	0.0081	1	U	R.YIFTMLSTLAR.L
3943	826 - 836	658.3576	1314.7007	1314.7006	0.100	0	46	0.00023	1	U	R.YIFTMLSTLAR.L
3944	826 - 836	658.3576	1314.7007	1314.7006	0.11	0	49	0.00013	1	U	R.YIFTMLSTLAR.L
3945	826 - 836	658.3577	1314.7008	1314.7006	0.21	0	43	0.00032	1	U	R.YIFTMLSTLAR.L
3946	826 - 836	658.3577	1314.7008	1314.7006	0.21	0	45	0.00013	1	U	R.YIFTMLSTLAR.L
3947	826 - 836	658.3578	1314.7011	1314.7006	0.39	0	38	0.00069	1	U	R.YIFTMLSTLAR.L
3948	826 - 836	658.3579	1314.7013	1314.7006	0.54	0	46	0.00013	1	U	R.YIFTMLSTLAR.L
3949	826 - 836	658.3580	1314.7013	1314.7006	0.59	0	38	0.0016	1	U	R.YIFTMLSTLAR.L
4057	826 - 836	666.3548	1330.6951	1330.6955	-0.31	0	32	0.0015	1	U	R.YIFTMLSTLAR.L + Oxidation (M)
4058	826 - 836	666.3548	1330.6951	1330.6955	-0.29	0	32	0.0024	1	U	R.YIFTMLSTLAR.L + Oxidation (M)
4059	826 - 836	666.3549	1330.6952	1330.6955	-0.22	0	55	3.6e-05	1	U	R.YIFTMLSTLAR.L + Oxidation (M)
4060	826 - 836	666.3550	1330.6953	1330.6955	-0.11	0	53	3.8e-05	1	U	R.YIFTMLSTLAR.L + Oxidation (M)
4061	826 - 836	666.3550	1330.6953	1330.6955	-0.11	0	51	6e-05	1	U	R.YIFTMLSTLAR.L + Oxidation (M)
4062	826 - 836	666.3551	1330.6956	1330.6955	0.069	0	53	4.4e-05	1	U	R.YIFTMLSTLAR.L + Oxidation (M)
4063	826 - 836	666.3551	1330.6956	1330.6955	0.084	0	56	2.1e-05	1	U	R.YIFTMLSTLAR.L + Oxidation (M)
4064	826 - 836	666.3552	1330.6958	1330.6955	0.22	0	50	9.4e-05	1	U	R.YIFTMLSTLAR.L + Oxidation (M)
4066	826 - 836	666.3553	1330.6961	1330.6955	0.46	0	24	0.0064	1	U	R.YIFTMLSTLAR.L + Oxidation (M)
4067	826 - 836	666.3554	1330.6962	1330.6955	0.54	0	54	3.5e-05	1	U	R.YIFTMLSTLAR.L + Oxidation (M)
4068	826 - 836	666.3555	1330.6965	1330.6955	0.75	0	23	0.036	1	U	R.YIFTMLSTLAR.L + Oxidation (M)
4069	826 - 836	666.3556	1330.6966	1330.6955	0.81	0	25	0.005	1	U	R.YIFTMLSTLAR.L + Oxidation (M)
4231	837 - 848	679.3887	1356.7628	1356.7653	-1.84	0	31	0.0068	1	U	R.LLFPAVDDNLLK.F
4233	837 - 848	679.3896	1356.7647	1356.7653	-0.46	0	22	0.037	1	U	R.LLFPAVDDNLLK.F
4235	837 - 848	679.3897	1356.7649	1356.7653	-0.30	0	40	0.0014	1	U	R.LLFPAVDDNLLK.F
4236	837 - 848	679.3898	1356.7649	1356.7653	-0.25	0	35	0.005	1	U	R.LLFPAVDDNLLK.F
4237	837 - 848	679.3898	1356.7651	1356.7653	-0.16	0	47	0.00024	1	U	R.LLFPAVDDNLLK.F
4238	837 - 848	679.3898	1356.7651	1356.7653	-0.16	0	50	0.00021	1	U	R.LLFPAVDDNLLK.F
4239	837 - 848	679.3898	1356.7651	1356.7653	-0.13	0	32	0.0028	1	U	R.LLFPAVDDNLLK.F
4240	837 - 848	679.3899	1356.7652	1356.7653	-0.089	0	31	0.024	1	U	R.LLFPAVDDNLLK.F
4242	837 - 848	679.3899	1356.7653	1356.7653	0.014	0	48	0.00017	1	U	R.LLFPAVDDNLLK.F
4243	837 - 848	679.3899	1356.7653	1356.7653	0.014	0	32	0.0025	1	U	R.LLFPAVDDNLLK.F
4244	837 - 848	679.3900	1356.7653	1356.7653	0.043	0	32	0.0035	1	U	R.LLFPAVDDNLLK.F
4245	837 - 848	679.3900	1356.7654	1356.7653	0.073	0	30	0.028	1	U	R.LLFPAVDDNLLK.F
4246	837 - 848	679.3900	1356.7654	1356.7653	0.088	0	25	0.045	1	U	R.LLFPAVDDNLLK.F
4247	837 - 848	679.3900	1356.7654	1356.7653	0.088	0	43	0.001	1	U	R.LLFPAVDDNLLK.F
4248	837 - 848	679.3900	1356.7655	1356.7653	0.16	0	16	0.036	1	U	R.LLFPAVDDNLLK.F
4249	837 - 848	679.3900	1356.7655	1356.7653	0.18	0	26	0.0047	1	U	R.LLFPAVDDNLLK.F
4250	837 - 848	679.3901	1356.7656	1356.7653	0.26	0	43	0.0016	1	U	R.LLFPAVDDNLLK.F
4251	837 - 848	679.3901	1356.7657	1356.7653	0.28	0	63	1.2e-05	1	U	R.LLFPAVDDNLLK.F
4252	837 - 848	679.3901	1356.7657	1356.7653	0.28	0	43	0.00068	1	U	R.LLFPAVDDNLLK.F
4253	837 - 848	679.3902	1356.7657	1356.7653	0.34	0	28	0.013	1	U	R.LLFPAVDDNLLK.F
4255	837 - 848	679.3902	1356.7658	1356.7653	0.35	0	33	0.021	1	U	R.LLFPAVDDNLLK.F
1938	849 - 856	535.7487	1069.4828	1069.4829	-0.034	0	35	0.015	1		K.FLYDDNQR.V
1940	849 - 856	535.7488	1069.4830	1069.4829	0.15	0	27	0.034	1		K.FLYDDNQR.V
579	885 - 891	424.7167	847.4188	847.4188	0.065	0	39	0.0048	1	U	K.LPNYDAR.E
580	885 - 891	424.7167	847.4189	847.4188	0.089	0	27	0.019	1	U	K.LPNYDAR.E
7285	900 - 914	870.9250	1739.8354	1739.8375	-1.21	0	26	0.011	1	U	R.MLDGLDPHPMLPNYK.N
7286	900 - 914	580.9528	1739.8365	1739.8375	-0.57	0	32	0.047	1	U	R.MLDGLDPHPMLPNYK.N
7287	900 - 914	870.9260	1739.8374	1739.8375	-0.041	0	57	4.2e-05	1	U	R.MLDGLDPHPMLPNYK.N
7288	900 - 914	870.9261	1739.8377	1739.8375	0.12	0	39	0.0026	1	U	R.MLDGLDPHPMLPNYK.N
7397	900 - 914	878.9225	1755.8304	1755.8324	-1.16	0	35	0.012	1	U	R.MLDGLDPHPMLPNYK.N + Oxidation (M)
7401	900 - 914	878.9233	1755.8321	1755.8324	-0.19	0	58	2.1e-05	1	U	R.MLDGLDPHPMLPNYK.N + Oxidation (M)
7506	900 - 914	886.9205	1771.8265	1771.8273	-0.44	0	26	0.0093	1	U	R.MLDGLDPHPMLPNYK.N + 2 Oxidation (M)
7507	900 - 914	591.6163	1771.8271	1771.8273	-0.14	0	34	0.011	1	U	R.MLDGLDPHPMLPNYK.N + 2 Oxidation (M)
7511	900 - 914	886.9212	1771.8279	1771.8273	0.35	0	46	0.00018	1	U	R.MLDGLDPHPMLPNYK.N + 2 Oxidation (M)
7513	900 - 914	886.9214	1771.8282	1771.8273	0.48	0	28	0.005	1	U	R.MLDGLDPHPMLPNYK.N + 2 Oxidation (M)
9990	918 - 939	1212.1116	2422.2087	2422.2129	-1.70	0	88	1.1e-07	1	U	K.GTIQELGQNQYAVSGEIFVVDR.N
9991	918 - 939	1212.1119	2422.2092	2422.2129	-1.51	0	122	6.1e-12	1	U	K.GTIQELGQNQYAVSGEIFVVDR.N
9993	918 - 939	1212.1124	2422.2103	2422.2129	-1.04	0	79	8.7e-08	1	U	K.GTIQELGQNQYAVSGEIFVVDR.N
9994	918 - 939	808.4108	2422.2107	2422.2129	-0.88	0	37	0.0038	1	U	K.GTIQELGQNQYAVSGEIFVVDR.N
9997	918 - 939	1212.1129	2422.2112	2422.2129	-0.69	0	94	1.6e-09	1	U	K.GTIQELGQNQYAVSGEIFVVDR.N
9999	918 - 939	1212.1130	2422.2114	2422.2129	-0.60	0	82	2.4e-07	1	U	K.GTIQELGQNQYAVSGEIFVVDR.N
10001	918 - 939	808.4112	2422.2119	2422.2129	-0.40	0	27	0.0059	1	U	K.GTIQELGQNQYAVSGEIFVVDR.N
10003	918 - 939	808.4114	2422.2124	2422.2129	-0.20	0	33	0.0044	1	U	K.GTIQELGQNQYAVSGEIFVVDR.N
10008	918 - 939	808.4117	2422.2133	2422.2129	0.18	0	28	0.028	1	U	K.GTIQELGQNQYAVSGEIFVVDR.N
10009	918 - 939	808.4117	2422.2133	2422.2129	0.18	0	28	0.022	1	U	K.GTIQELGQNQYAVSGEIFVVDR.N
10010	918 - 939	1212.1140	2422.2133	2422.2129	0.20	0	90	7.7e-09	1	U	K.GTIQELGQNQYAVSGEIFVVDR.N
10012	918 - 939	606.5606	2422.2135	2422.2129	0.25	0	38	0.015	1	U	K.GTIQELGQNQYAVSGEIFVVDR.N
10013	918 - 939	808.4118	2422.2135	2422.2129	0.26	0	43	0.0004	1	U	K.GTIQELGQNQYAVSGEIFVVDR.N
10016	918 - 939	808.4118	2422.2137	2422.2129	0.33	0	31	0.011	1	U	K.GTIQELGQNQYAVSGEIFVVDR.N
10021	918 - 939	1212.1144	2422.2142	2422.2129	0.57	0	78	5.1e-07	1	U	K.GTIQELGQNQYAVSGEIFVVDR.N

Query	Start - End	Observed	Mr(expt)	Mr(calc)	ppm	M	Score	Expect	Rank	U	Peptide
10022	918 - 939	808.4120	2422.2143	2422.2129	0.59	0	22	0.036	1	U	K.GTIQELGQNQYAVSGEIFVVDR.N
10023	918 - 939	808.4121	2422.2146	2422.2129	0.70	0	43	0.0009	1	U	K.GTIQELGQNQYAVSGEIFVVDR.N
10027	918 - 939	808.4123	2422.2150	2422.2129	0.86	0	23	0.029	1	U	K.GTIQELGQNQYAVSGEIFVVDR.N
10030	918 - 939	808.4130	2422.2172	2422.2129	1.79	0	41	0.00098	1	U	K.GTIQELGQNQYAVSGEIFVVDR.N
10031	918 - 939	1212.1173	2422.2200	2422.2129	2.97	0	38	0.00046	1	U	K.GTIQELGQNQYAVSGEIFVVDR.N
12696	918 - 950	919.4807	3673.8939	3673.8952	-0.35	1	30	0.011	1	U	K.GTIQELGQNQYAVSGEIFVVDRNTVEITELPVR.T
12697	918 - 950	1225.6394	3673.8963	3673.8952	0.30	1	88	1.3e-07	1	U	K.GTIQELGQNQYAVSGEIFVVDRNTVEITELPVR.T
12698	918 - 950	919.4824	3673.9007	3673.8952	1.51	1	24	0.038	1	U	K.GTIQELGQNQYAVSGEIFVVDRNTVEITELPVR.T
3570	940 - 950	635.8532	1269.6918	1269.6929	-0.83	0	34	0.0095	1	U	R.NTVEITELPVR.T
3572	940 - 950	635.8533	1269.6920	1269.6929	-0.65	0	67	9.9e-06	1	U	R.NTVEITELPVR.T
3573	940 - 950	635.8534	1269.6922	1269.6929	-0.49	0	40	0.0027	1	U	R.NTVEITELPVR.T
3575	940 - 950	635.8534	1269.6923	1269.6929	-0.45	0	31	0.016	1	U	R.NTVEITELPVR.T
3576	940 - 950	635.8535	1269.6924	1269.6929	-0.34	0	26	0.0042	1	U	R.NTVEITELPVR.T
3578	940 - 950	635.8536	1269.6927	1269.6929	-0.15	0	52	0.00029	1	U	R.NTVEITELPVR.T
3579	940 - 950	635.8536	1269.6927	1269.6929	-0.12	0	49	0.00014	1	U	R.NTVEITELPVR.T
3581	940 - 950	635.8538	1269.6930	1269.6929	0.14	0	27	0.0028	1	U	R.NTVEITELPVR.T
3582	940 - 950	635.8538	1269.6931	1269.6929	0.23	0	35	0.0032	1	U	R.NTVEITELPVR.T
3583	940 - 950	635.8539	1269.6932	1269.6929	0.25	0	20	0.05	1	U	R.NTVEITELPVR.T
3584	940 - 950	635.8539	1269.6932	1269.6929	0.29	0	50	0.00023	1	U	R.NTVEITELPVR.T
3585	940 - 950	424.2384	1269.6933	1269.6929	0.36	0	55	0.00015	1	U	R.NTVEITELPVR.T
3586	940 - 950	635.8540	1269.6934	1269.6929	0.42	0	55	0.00018	1	U	R.NTVEITELPVR.T
971	951 - 957	463.2425	924.4704	924.4705	-0.100	0	34	0.031	1	U	R.TWTQVYK.E
5075	958 - 970	737.3661	1472.7176	1472.7181	-0.32	0	23	0.041	1	U	K.EQVLEPMLNGTDK.T
5193	958 - 970	745.3632	1488.7119	1488.7130	-0.76	0	49	0.00019	1	U	K.EQVLEPMLNGTDK.T + Oxidation (M)
5197	958 - 970	745.3639	1488.7132	1488.7130	0.11	0	41	0.0015	1	U	K.EQVLEPMLNGTDK.T + Oxidation (M)
10195	958 - 979	826.7528	2477.2367	2477.2359	0.30	1	33	0.0082	1	U	K.EQVLEPMLNGTDKTPALISDYK.E + Oxidation (M)
1471	971 - 979	504.2740	1006.5334	1006.5335	-0.045	0	24	0.029	1	U	K.TPALISDYK.E
1472	971 - 979	504.2741	1006.5337	1006.5335	0.17	0	36	0.0032	1	U	K.TPALISDYK.E
1473	971 - 979	504.2744	1006.5343	1006.5335	0.79	0	58	5.7e-05	1	U	K.TPALISDYK.E
9066	971 - 988	694.6839	2081.0299	2081.0317	-0.85	1	41	0.0011	1	U	K.TPALISDYKEYHTDTTVK.F
9070	971 - 988	1041.5226	2081.0307	2081.0317	-0.46	1	55	7.6e-06	1	U	K.TPALISDYKEYHTDTTVK.F
9074	971 - 988	1041.5232	2081.0319	2081.0317	0.12	1	61	3.3e-06	1	U	K.TPALISDYKEYHTDTTVK.F
9075	971 - 988	694.6846	2081.0320	2081.0317	0.13	1	22	0.033	1	U	K.TPALISDYKEYHTDTTVK.F
9077	971 - 988	694.6848	2081.0327	2081.0317	0.48	1	27	0.02	1	U	K.TPALISDYKEYHTDTTVK.F
9078	971 - 988	694.6866	2081.0380	2081.0317	3.03	1	20	0.03	1	U	K.TPALISDYKEYHTDTTVK.F
2158	980 - 988	547.2614	1092.5082	1092.5088	-0.50	0	21	0.016	1		K.EYHTDTTVK.F
2259	998 - 1008	370.2080	1107.6021	1107.6036	-1.34	0	29	0.014	1	U	K.LAQAEAAGLHK.V
2260	998 - 1008	554.8086	1107.6026	1107.6036	-0.95	0	60	2.5e-05	1	U	K.LAQAEAAGLHK.V
2262	998 - 1008	554.8089	1107.6033	1107.6036	-0.27	0	32	0.012	1	U	K.LAQAEAAGLHK.V
2263	998 - 1008	370.2084	1107.6033	1107.6036	-0.25	0	40	0.0038	1	U	K.LAQAEAAGLHK.V
2265	998 - 1008	370.2084	1107.6035	1107.6036	-0.14	0	33	0.003	1	U	K.LAQAEAAGLHK.V
2266	998 - 1008	370.2084	1107.6035	1107.6036	-0.14	0	29	0.024	1	U	K.LAQAEAAGLHK.V
2267	998 - 1008	370.2085	1107.6036	1107.6036	0.018	0	40	0.0022	1	U	K.LAQAEAAGLHK.V
2268	998 - 1008	370.2085	1107.6036	1107.6036	0.018	0	27	0.043	1	U	K.LAQAEAAGLHK.V
2269	998 - 1008	370.2085	1107.6037	1107.6036	0.099	0	43	0.0014	1	U	K.LAQAEAAGLHK.V
2270	998 - 1008	554.8092	1107.6038	1107.6036	0.15	0	45	0.00063	1	U	K.LAQAEAAGLHK.V
2271	998 - 1008	554.8092	1107.6039	1107.6036	0.28	0	28	0.012	1	U	K.LAQAEAAGLHK.V
3256	1032 - 1041	618.8448	1235.6751	1235.6761	-0.86	1	36	0.0011	1	U	K.KYETVQDILK.E
3258	1032 - 1041	618.8452	1235.6758	1235.6761	-0.23	1	54	5.9e-05	1	U	K.KYETVQDILK.E
3259	1032 - 1041	412.8993	1235.6760	1235.6761	-0.12	1	37	0.0065	1	U	K.KYETVQDILK.E
3260	1032 - 1041	618.8453	1235.6761	1235.6761	-0.052	1	73	4.9e-07	1	U	K.KYETVQDILK.E
3261	1032 - 1041	618.8454	1235.6761	1235.6761	0.013	1	48	7e-05	1	U	K.KYETVQDILK.E
3262	1032 - 1041	618.8454	1235.6763	1235.6761	0.16	1	50	8e-05	1	U	K.KYETVQDILK.E
3263	1032 - 1041	618.8455	1235.6764	1235.6761	0.22	1	25	0.013	1	U	K.KYETVQDILK.E
3264	1032 - 1041	618.8455	1235.6764	1235.6761	0.26	1	26	0.048	1	U	K.KYETVQDILK.E
3265	1032 - 1041	618.8455	1235.6765	1235.6761	0.27	1	30	0.0039	1	U	K.KYETVQDILK.E
2258	1033 - 1041	554.7988	1107.5831	1107.5812	1.78	0	39	0.0084	1	U	K.YETVQDILK.E
695	1048 - 1054	436.2371	870.4597	870.4599	-0.26	0	30	0.023	1	U	R.LSYYGLR.K
696	1048 - 1054	436.2372	870.4599	870.4599	-0.025	0	22	0.036	1	U	R.LSYYGLR.K
5847	1055 - 1068	774.9080	1547.8015	1547.8017	-0.16	1	67	1.7e-05	1	U	R.KEWLVGMLGAESTK.L
5848	1055 - 1068	516.9412	1547.8017	1547.8017	-0.039	1	37	0.0074	1	U	R.KEWLVGMLGAESTK.L
5969	1055 - 1068	522.2724	1563.7954	1563.7967	-0.81	1	14	0.048	1	U	R.KEWLVGMLGAESTK.L + Oxidation (M)
5970	1055 - 1068	782.9050	1563.7955	1563.7967	-0.74	1	56	6e-05	1	U	R.KEWLVGMLGAESTK.L + Oxidation (M)
5971	1055 - 1068	522.2725	1563.7956	1563.7967	-0.67	1	27	0.015	1	U	R.KEWLVGMLGAESTK.L + Oxidation (M)
5972	1055 - 1068	782.9053	1563.7960	1563.7967	-0.43	1	76	4.5e-07	1	U	R.KEWLVGMLGAESTK.L + Oxidation (M)
5974	1055 - 1068	782.9055	1563.7965	1563.7967	-0.085	1	68	1.5e-06	1	U	R.KEWLVGMLGAESTK.L + Oxidation (M)
5975	1055 - 1068	782.9057	1563.7968	1563.7967	0.068	1	68	4e-06	1	U	R.KEWLVGMLGAESTK.L + Oxidation (M)
5976	1055 - 1068	522.2730	1563.7973	1563.7967	0.38	1	20	0.013	1	U	R.KEWLVGMLGAESTK.L + Oxidation (M)
5977	1055 - 1068	522.2731	1563.7976	1563.7967	0.60	1	33	0.0025	1	U	R.KEWLVGMLGAESTK.L + Oxidation (M)
4623	1056 - 1068	710.8608	1419.7070	1419.7068	0.18	0	32	0.0022	1	U	K.EWLVGMLGAESTK.L
4625	1056 - 1068	710.8612	1419.7079	1419.7068	0.80	0	34	0.0025	1	U	K.EWLVGMLGAESTK.L
4761	1056 - 1068	718.8567	1435.6989	1435.7017	-1.96	0	33	0.043	1	U	K.EWLVGMLGAESTK.L + Oxidation (M)
4763	1056 - 1068	718.8572	1435.6999	1435.7017	-1.22	0	34	0.033	1	U	K.EWLVGMLGAESTK.L + Oxidation (M)
3340	1092 - 1101	415.2443	1242.7110	1242.7118	-0.64	1	44	0.0015	1	U	K.KDLIQMLVQR.G
3341	1092 - 1101	622.3628	1242.7111	1242.7118	-0.56	1	57	7.9e-05	1	U	K.KDLIQMLVQR.G
3482	1092 - 1101	630.3597	1258.7049	1258.7067	-1.46	1	55	0.00014	1	U	K.KDLIQMLVQR.G + Oxidation (M)
3483	1092 - 1101	630.3608	1258.7070	1258.7067	0.26	1	55	0.00014	1	U	K.KDLIQMLVQR.G + Oxidation (M)
3484	1092 - 1101	420.5766	1258.7080	1258.7067	1.01	1	38	0.0057	1	U	K.KDLIQMLVQR.G + Oxidation (M)

Query	Start – End	Observed	Mr(expt)	Mr(calc)	ppm	M	Score	Expect	Rank	U	Peptide
2326	1093 – 1101	558.3155	1114.6164	1114.6169	-0.43	0	45	0.0019	1	U	K.DLIQMLVQR.G
2327	1093 – 1101	558.3157	1114.6169	1114.6169	0.014	0	64	2.3e-05	1	U	K.DLIQMLVQR.G
2328	1093 – 1101	558.3157	1114.6169	1114.6169	0.014	0	51	0.0004	1	U	K.DLIQMLVQR.G
2329	1093 – 1101	558.3158	1114.6171	1114.6169	0.25	0	52	0.0004	1	U	K.DLIQMLVQR.G
2475	1093 – 1101	566.3125	1130.6104	1130.6118	-1.21	0	51	0.00039	1	U	K.DLIQMLVQR.G + Oxidation (M)
2476	1093 – 1101	566.3125	1130.6105	1130.6118	-1.12	0	54	0.00026	1	U	K.DLIQMLVQR.G + Oxidation (M)
2477	1093 – 1101	566.3128	1130.6110	1130.6118	-0.67	0	54	0.00025	1	U	K.DLIQMLVQR.G + Oxidation (M)
2478	1093 – 1101	566.3128	1130.6111	1130.6118	-0.57	0	47	0.00092	1	U	K.DLIQMLVQR.G + Oxidation (M)
2479	1093 – 1101	566.3130	1130.6114	1130.6118	-0.36	0	44	0.002	1	U	K.DLIQMLVQR.G + Oxidation (M)
2480	1093 – 1101	566.3130	1130.6114	1130.6118	-0.36	0	48	0.00043	1	U	K.DLIQMLVQR.G + Oxidation (M)
2481	1093 – 1101	566.3130	1130.6114	1130.6118	-0.30	0	37	0.012	1	U	K.DLIQMLVQR.G + Oxidation (M)
2482	1093 – 1101	566.3133	1130.6120	1130.6118	0.19	0	52	0.00046	1	U	K.DLIQMLVQR.G + Oxidation (M)
2483	1093 – 1101	566.3135	1130.6124	1130.6118	0.53	0	32	0.041	1	U	K.DLIQMLVQR.G + Oxidation (M)
2485	1093 – 1101	566.3135	1130.6125	1130.6118	0.65	0	33	0.0078	1	U	K.DLIQMLVQR.G + Oxidation (M)
2486	1093 – 1101	566.3140	1130.6134	1130.6118	1.41	0	52	0.00037	1	U	K.DLIQMLVQR.G + Oxidation (M)
790	1102 – 1109	447.7128	893.4111	893.4131	-2.13	0	38	0.00053	1	U	R.GYESDPVK.A
793	1102 – 1109	447.7140	893.4135	893.4131	0.46	0	41	0.00032	1	U	R.GYESDPVK.A
794	1102 – 1109	447.7142	893.4138	893.4131	0.89	0	16	0.034	1	U	R.GYESDPVK.A
1350	1157 – 1164	494.2897	986.5648	986.5648	0.034	1	33	0.022	2	U	K.EKVEELIK.Q
1351	1157 – 1164	494.2899	986.5652	986.5648	0.44	1	28	0.041	2	U	K.EKVEELIK.Q
1003	1170 – 1177	465.7541	929.4936	929.4930	0.63	1	31	0.041	1	U	K.GREVNDLK.R
2098	1170 – 1178	543.8044	1085.5943	1085.5941	0.20	2	44	0.002	1	U	K.GREVNDLKR.K
2099	1170 – 1178	362.8722	1085.5949	1085.5941	0.67	2	47	0.0012	1	U	K.GREVNDLKR.K
710	1172 – 1178	437.2432	872.4717	872.4716	0.22	1	44	0.0021	1	U	R.EVNDLKR.K
493	1180 – 1186	416.7139	831.4132	831.4127	0.68	0	35	0.016	1		K.SPSDLWK.E
4376	1187 – 1198	689.8401	1377.6657	1377.6664	-0.47	0	58	4.1e-05	1	U	K.EDLAAFVEELDK.V
4379	1187 – 1198	689.8413	1377.6681	1377.6664	1.29	0	54	1.8e-05	1	U	K.EDLAAFVEELDK.V
9170	1187 – 1204	1054.0100	2106.0054	2106.0117	-2.98	1	88	6.6e-08	1	U	K.EDLAAFVEELDKVESQER.E
9171	1187 – 1204	703.0102	2106.0089	2106.0117	-1.33	1	27	0.011	1	U	K.EDLAAFVEELDKVESQER.E
9173	1187 – 1204	703.0108	2106.0107	2106.0117	-0.47	1	44	0.00075	1	U	K.EDLAAFVEELDKVESQER.E
9174	1187 – 1204	1054.0127	2106.0108	2106.0117	-0.42	1	64	3.2e-05	1	U	K.EDLAAFVEELDKVESQER.E
9175	1187 – 1204	703.0109	2106.0108	2106.0117	-0.39	1	46	0.00065	1	U	K.EDLAAFVEELDKVESQER.E
9176	1187 – 1204	1054.0129	2106.0112	2106.0117	-0.19	1	84	5.4e-08	1	U	K.EDLAAFVEELDKVESQER.E
9177	1187 – 1204	703.0111	2106.0114	2106.0117	-0.13	1	38	0.0037	1	U	K.EDLAAFVEELDKVESQER.E
9179	1187 – 1204	703.0111	2106.0114	2106.0117	-0.12	1	50	0.00028	1	U	K.EDLAAFVEELDKVESQER.E
9180	1187 – 1204	703.0111	2106.0115	2106.0117	-0.074	1	33	0.0053	1	U	K.EDLAAFVEELDKVESQER.E
9181	1187 – 1204	703.0111	2106.0116	2106.0117	-0.046	1	44	0.0022	1	U	K.EDLAAFVEELDKVESQER.E
9182	1187 – 1204	703.0112	2106.0117	2106.0117	0.011	1	41	0.0019	1	U	K.EDLAAFVEELDKVESQER.E
9183	1187 – 1204	1054.0131	2106.0117	2106.0117	0.033	1	76	6.7e-07	1	U	K.EDLAAFVEELDKVESQER.E
9184	1187 – 1204	1054.0131	2106.0117	2106.0117	0.033	1	64	6e-06	1	U	K.EDLAAFVEELDKVESQER.E
9185	1187 – 1204	703.0112	2106.0118	2106.0117	0.054	1	44	0.0003	1	U	K.EDLAAFVEELDKVESQER.E
9186	1187 – 1204	1054.0133	2106.0120	2106.0117	0.15	1	60	3.1e-05	1	U	K.EDLAAFVEELDKVESQER.E
9187	1187 – 1204	703.0113	2106.0121	2106.0117	0.21	1	41	0.0018	1	U	K.EDLAAFVEELDKVESQER.E
9188	1187 – 1204	703.0113	2106.0121	2106.0117	0.23	1	44	0.00096	1	U	K.EDLAAFVEELDKVESQER.E
9189	1187 – 1204	703.0113	2106.0122	2106.0117	0.24	1	53	8.8e-05	1	U	K.EDLAAFVEELDKVESQER.E
9190	1187 – 1204	703.0113	2106.0122	2106.0117	0.24	1	54	0.00021	1	U	K.EDLAAFVEELDKVESQER.E
9191	1187 – 1204	703.0114	2106.0123	2106.0117	0.32	1	38	0.0017	1	U	K.EDLAAFVEELDKVESQER.E
9192	1187 – 1204	1054.0140	2106.0134	2106.0117	0.84	1	57	3.8e-05	1	U	K.EDLAAFVEELDKVESQER.E
11594	1187 – 1214	1032.1680	3093.4821	3093.4812	0.28	2	54	0.0013	1	U	K.EDLAAFVEELDKVESQEREDVLAGMSGK.A
11600	1187 – 1214	1032.1684	3093.4835	3093.4812	0.76	2	64	9.6e-06	1	U	K.EDLAAFVEELDKVESQEREDVLAGMSGK.A
11604	1187 – 1214	1032.1702	3093.4887	3093.4812	2.42	2	40	0.0025	1	U	K.EDLAAFVEELDKVESQEREDVLAGMSGK.A
11657	1187 – 1214	1037.4985	3109.4737	3109.4761	-0.78	2	48	0.00017	1	U	K.EDLAAFVEELDKVESQEREDVLAGMSGK.A + Oxidation (M)
11659	1187 – 1214	1037.4989	3109.4748	3109.4761	-0.43	2	40	0.0013	1	U	K.EDLAAFVEELDKVESQEREDVLAGMSGK.A + Oxidation (M)
11660	1187 – 1214	1037.4996	3109.4769	3109.4761	0.27	2	59	1.6e-05	1	U	K.EDLAAFVEELDKVESQEREDVLAGMSGK.A + Oxidation (M)
11668	1187 – 1214	1037.5005	3109.4796	3109.4761	1.12	2	49	0.00022	1	U	K.EDLAAFVEELDKVESQEREDVLAGMSGK.A + Oxidation (M)
7244	1199 – 1214	867.9206	1733.8267	1733.8254	0.78	1	64	3.4e-06	1	U	K.VESQEREDVLAGMSGK.A
7246	1199 – 1214	867.9225	1733.8304	1733.8254	2.88	1	52	7.2e-05	1	U	K.VESQEREDVLAGMSGK.A
7359	1199 – 1214	584.2806	1749.8200	1749.8203	-0.16	1	43	0.00095	1	U	K.VESQEREDVLAGMSGK.A + Oxidation (M)
7360	1199 – 1214	875.9176	1749.8206	1749.8203	0.19	1	64	4.9e-06	1	U	K.VESQEREDVLAGMSGK.A + Oxidation (M)
1460	1205 – 1214	503.7472	1005.4799	1005.4801	-0.16	0	46	0.00029	1	U	R.EDVLAGMSGK.A
1571	1205 – 1214	511.7444	1021.4743	1021.4750	-0.66	0	46	0.0005	1	U	R.EDVLAGMSGK.A + Oxidation (M)
1572	1205 – 1214	511.7445	1021.4744	1021.4750	-0.59	0	53	0.00011	1	U	R.EDVLAGMSGK.A + Oxidation (M)
1573	1205 – 1214	511.7445	1021.4744	1021.4750	-0.55	0	48	0.00038	1	U	R.EDVLAGMSGK.A + Oxidation (M)
1574	1205 – 1214	511.7446	1021.4747	1021.4750	-0.29	0	40	0.00075	1	U	R.EDVLAGMSGK.A + Oxidation (M)
1576	1205 – 1214	511.7448	1021.4750	1021.4750	0.022	0	40	0.0021	1	U	R.EDVLAGMSGK.A + Oxidation (M)
1577	1205 – 1214	511.7449	1021.4753	1021.4750	0.26	0	56	8.8e-05	1	U	R.EDVLAGMSGK.A + Oxidation (M)
1578	1205 – 1214	511.7450	1021.4753	1021.4750	0.33	0	39	0.0017	1	U	R.EDVLAGMSGK.A + Oxidation (M)
1579	1205 – 1214	511.7450	1021.4754	1021.4750	0.37	0	42	0.0013	1	U	R.EDVLAGMSGK.A + Oxidation (M)
6582	1227 – 1240	550.2835	1647.8287	1647.8290	-0.19	1	16	0.049	1	U	K.KLQLEETMPSPYGR.R

Query	Start - End	Observed	Mr(expt)	Mr(calc)	ppm	M	Score	Expect	Rank	U	Peptide
6583	1227 - 1240	824.9218	1647.8291	1647.8290	0.035	1	69	5.4e-07	1	U	K.KLQLEETMPSPYGR.R
6584	1227 - 1240	824.9218	1647.8291	1647.8290	0.084	1	84	6.9e-08	1	U	K.KLQLEETMPSPYGR.R
6586	1227 - 1240	550.2840	1647.8303	1647.8290	0.78	1	26	0.011	1	U	K.KLQLEETMPSPYGR.R
6672	1227 - 1240	555.6147	1663.8223	1663.8239	-0.96	1	34	0.0088	1	U	K.KLQLEETMPSPYGR.R + Oxidation (M)
6673	1227 - 1240	832.9186	1663.8227	1663.8239	-0.75	1	69	4.5e-07	1	U	K.KLQLEETMPSPYGR.R + Oxidation (M)
6674	1227 - 1240	832.9187	1663.8229	1663.8239	-0.64	1	95	3.5e-09	1	U	K.KLQLEETMPSPYGR.R + Oxidation (M)
6675	1227 - 1240	832.9188	1663.8230	1663.8239	-0.57	1	66	2e-06	1	U	K.KLQLEETMPSPYGR.R + Oxidation (M)
6678	1227 - 1240	832.9188	1663.8231	1663.8239	-0.47	1	68	1.7e-06	1	U	K.KLQLEETMPSPYGR.R + Oxidation (M)
6679	1227 - 1240	832.9190	1663.8235	1663.8239	-0.27	1	17	0.044	1	U	K.KLQLEETMPSPYGR.R + Oxidation (M)
6680	1227 - 1240	832.9190	1663.8235	1663.8239	-0.23	1	51	4.5e-05	1	U	K.KLQLEETMPSPYGR.R + Oxidation (M)
6681	1227 - 1240	832.9191	1663.8236	1663.8239	-0.17	1	52	4e-05	1	U	K.KLQLEETMPSPYGR.R + Oxidation (M)
6682	1227 - 1240	832.9192	1663.8237	1663.8239	-0.11	1	66	2.2e-06	1	U	K.KLQLEETMPSPYGR.R + Oxidation (M)
6683	1227 - 1240	832.9192	1663.8238	1663.8239	-0.097	1	75	2.5e-07	1	U	K.KLQLEETMPSPYGR.R + Oxidation (M)
6685	1227 - 1240	555.6153	1663.8242	1663.8239	0.16	1	21	0.035	1	U	K.KLQLEETMPSPYGR.R + Oxidation (M)
6686	1227 - 1240	555.6154	1663.8244	1663.8239	0.28	1	19	0.049	1	U	K.KLQLEETMPSPYGR.R + Oxidation (M)
6687	1227 - 1240	555.6155	1663.8247	1663.8239	0.46	1	30	0.023	1	U	K.KLQLEETMPSPYGR.R + Oxidation (M)
6688	1227 - 1240	832.9197	1663.8248	1663.8239	0.52	1	68	1.1e-06	1	U	K.KLQLEETMPSPYGR.R + Oxidation (M)
5524	1228 - 1240	760.8741	1519.7337	1519.7341	-0.25	0	56	2.4e-05	1	U	K.LQLEETMPSPYGR.R
5528	1228 - 1240	507.5862	1519.7367	1519.7341	1.73	0	23	0.038	1	U	K.LQLEETMPSPYGR.R
5529	1228 - 1240	760.8761	1519.7376	1519.7341	2.32	0	32	0.0021	1	U	K.LQLEETMPSPYGR.R
5729	1228 - 1240	768.8716	1535.7287	1535.7290	-0.15	0	32	0.0048	1	U	K.LQLEETMPSPYGR.R + Oxidation (M)
5731	1228 - 1240	768.8718	1535.7290	1535.7290	-0.0033	0	67	4.9e-06	1	U	K.LQLEETMPSPYGR.R + Oxidation (M)
5734	1228 - 1240	768.8718	1535.7291	1535.7290	0.10	0	63	4.7e-06	1	U	K.LQLEETMPSPYGR.R + Oxidation (M)
5735	1228 - 1240	768.8718	1535.7291	1535.7290	0.11	0	67	4.1e-06	1	U	K.LQLEETMPSPYGR.R + Oxidation (M)
5736	1228 - 1240	768.8719	1535.7293	1535.7290	0.21	0	74	5.5e-07	1	U	K.LQLEETMPSPYGR.R + Oxidation (M)
5737	1228 - 1240	768.8720	1535.7295	1535.7290	0.35	0	84	1.3e-07	1	U	K.LQLEETMPSPYGR.R + Oxidation (M)
2808	1241 - 1250	586.3469	1170.6792	1170.6794	-0.22	1	21	0.039	1	U	R.RIIPEITAMK.A
2810	1241 - 1250	586.3470	1170.6794	1170.6794	-0.034	1	38	0.00084	1	U	R.RIIPEITAMK.A
2811	1241 - 1250	391.2338	1170.6794	1170.6794	-0.0051	1	36	0.00057	1	U	R.RIIPEITAMK.A
2911	1241 - 1250	594.3439	1186.6733	1186.6743	-0.89	1	34	0.0013	1	U	R.RIIPEITAMK.A + Oxidation (M)
2912	1241 - 1250	396.5654	1186.6743	1186.6743	-0.080	1	39	0.00055	1	U	R.RIIPEITAMK.A + Oxidation (M)
2916	1241 - 1250	594.3445	1186.6745	1186.6743	0.10	1	37	0.00093	1	U	R.RIIPEITAMK.A + Oxidation (M)
2917	1241 - 1250	594.3445	1186.6745	1186.6743	0.12	1	38	0.00055	1	U	R.RIIPEITAMK.A + Oxidation (M)
2919	1241 - 1250	594.3448	1186.6749	1186.6743	0.51	1	34	0.0013	1	U	R.RIIPEITAMK.A + Oxidation (M)
1532	1242 - 1250	508.2964	1014.5782	1014.5783	-0.074	0	42	0.0018	1	U	R.IIPEITAMK.A
1642	1242 - 1250	516.2939	1030.5733	1030.5732	0.045	0	28	0.013	1	U	R.IIPEITAMK.A + Oxidation (M)
2603	1261 - 1271	573.3300	1144.6453	1144.6452	0.15	2	65	1.4e-05	1	U	K.KKGDLDTAAVK.V
1548	1262 - 1271	509.2820	1016.5495	1016.5502	-0.68	1	64	2.4e-05	1	U	K.KGDLDTAAVK.V
1549	1262 - 1271	509.2822	1016.5498	1016.5502	-0.36	1	48	0.00092	1	U	K.KGDLDTAAVK.V
1550	1262 - 1271	509.2822	1016.5498	1016.5502	-0.36	1	31	0.048	1	U	K.KGDLDTAAVK.V
1551	1262 - 1271	509.2824	1016.5502	1016.5502	-0.048	1	40	0.0056	1	U	K.KGDLDTAAVK.V
1552	1262 - 1271	509.2826	1016.5506	1016.5502	0.35	1	50	0.00058	1	U	K.KGDLDTAAVK.V
2189	1262 - 1271	549.2657	1096.5169	1096.5165	0.32	1	40	0.0052	1	U	K.KGDLDTAAVK.V + Phospho (ST)
776	1263 - 1271	445.2349	888.4552	888.4553	-0.10	0	33	0.029	1	U	K.GDLDTAAVK.V
777	1263 - 1271	445.2350	888.4553	888.4553	0.098	0	34	0.018	1	U	K.GDLDTAAVK.V
11081	1272 - 1299	973.4680	2917.3821	2917.3869	-1.64	0	31	0.0046	1	U	K.VEFDEEFSGAPVEGAGEEALTPSVPINK.G
11083	1272 - 1299	973.4688	2917.3845	2917.3869	-0.83	0	28	0.016	1	U	K.VEFDEEFSGAPVEGAGEEALTPSVPINK.G
11086	1272 - 1299	973.4688	2917.3846	2917.3869	-0.78	0	36	0.0018	1	U	K.VEFDEEFSGAPVEGAGEEALTPSVPINK.G
11087	1272 - 1299	973.4689	2917.3848	2917.3869	-0.72	0	47	0.00019	1	U	K.VEFDEEFSGAPVEGAGEEALTPSVPINK.G
11092	1272 - 1299	973.4694	2917.3865	2917.3869	-0.13	0	50	7.2e-05	1	U	K.VEFDEEFSGAPVEGAGEEALTPSVPINK.G
11096	1272 - 1299	973.4696	2917.3870	2917.3869	0.034	0	60	4.8e-05	1	U	K.VEFDEEFSGAPVEGAGEEALTPSVPINK.G
11098	1272 - 1299	973.4697	2917.3872	2917.3869	0.12	0	39	0.0021	1	U	K.VEFDEEFSGAPVEGAGEEALTPSVPINK.G
11100	1272 - 1299	973.4698	2917.3874	2917.3869	0.18	0	42	0.0003	1	U	K.VEFDEEFSGAPVEGAGEEALTPSVPINK.G
11101	1272 - 1299	973.4698	2917.3876	2917.3869	0.23	0	40	0.0026	1	U	K.VEFDEEFSGAPVEGAGEEALTPSVPINK.G
11102	1272 - 1299	973.4699	2917.3880	2917.3869	0.37	0	31	0.0071	1	U	K.VEFDEEFSGAPVEGAGEEALTPSVPINK.G
11103	1272 - 1299	973.4701	2917.3885	2917.3869	0.55	0	56	3.9e-05	1	U	K.VEFDEEFSGAPVEGAGEEALTPSVPINK.G
11107	1272 - 1299	973.4706	2917.3900	2917.3869	1.05	0	26	0.041	1	U	K.VEFDEEFSGAPVEGAGEEALTPSVPINK.G
11109	1272 - 1299	973.4715	2917.3927	2917.3869	2.00	0	34	0.0031	1	U	K.VEFDEEFSGAPVEGAGEEALTPSVPINK.G
11387	1272 - 1299	1000.1245	2997.3516	2997.3532	-0.56	0	55	7.8e-05	1	U	K.VEFDEEFSGAPVEGAGEEALTPSVPINK.G + Phospho (ST)
2932	1316 - 1327	396.8907	1187.6502	1187.6510	-0.63	1	26	0.027	1	U	R.KTPTSSGKPSAK.K
2935	1316 - 1327	594.8327	1187.6508	1187.6510	-0.13	1	31	0.009	1	U	R.KTPTSSGKPSAK.K
2936	1316 - 1327	396.8909	1187.6508	1187.6510	-0.12	1	33	0.024	1	U	R.KTPTSSGKPSAK.K
2937	1316 - 1327	594.8328	1187.6511	1187.6510	0.069	1	52	0.00042	1	U	R.KTPTSSGKPSAK.K
2940	1316 - 1327	594.8330	1187.6514	1187.6510	0.34	1	33	0.013	1	U	R.KTPTSSGKPSAK.K
1864	1317 - 1327	530.7851	1059.5557	1059.5560	-0.34	0	25	0.02	1	U	K.TPTSSGKPSAK.K
1868	1317 - 1327	530.7853	1059.5560	1059.5560	-0.0019	0	37	0.0013	1	U	K.TPTSSGKPSAK.K

Query	Start - End	Observed	Mr(expt)	Mr(calc)	ppm	M	Score	Expect	Rank	U	Peptide
11070	1332 - 1356	972.1275	2913.3608	2913.3628	-0.69	2	83	1e-07	1	U	K.RNPWSDDESKSESDLEETEPVVIPR.D
2005	1333 - 1341	539.2279	1076.4412	1076.4411	0.14	0	41	0.0005	1	U	R.NPWSDDESK.S
10784	1333 - 1356	690.3226	2757.2615	2757.2617	-0.084	1	46	0.0011	1	U	R.NPWSDDESKSESDLEETEPVVIPR.D
10785	1333 - 1356	920.0944	2757.2615	2757.2617	-0.071	1	60	9.7e-05	1	U	R.NPWSDDESKSESDLEETEPVVIPR.D
10787	1333 - 1356	690.3252	2757.2717	2757.2617	3.64	1	43	0.00074	1	U	R.NPWSDDESKSESDLEETEPVVIPR.D
10955	1333 - 1356	946.7501	2837.2284	2837.2280	0.15	1	43	0.0015	1	U	R.NPWSDDESKSESDLEETEPVVIPR.D
											+ Phospho (ST)
6980	1342 - 1356	567.2837	1698.8293	1698.8312	-1.11	0	43	0.0053	1	U	K.SESDLEETEPVVIPR.D
6982	1342 - 1356	850.4229	1698.8313	1698.8312	0.080	0	78	1.6e-06	1	U	K.SESDLEETEPVVIPR.D
7545	1342 - 1356	890.4078	1778.8010	1778.7975	1.99	0	28	0.023	1	U	K.SESDLEETEPVVIPR.D
											+ Phospho (ST)
818	1362 - 1369	449.7643	897.5141	897.5144	-0.37	1	26	0.034	1	U	R.RAAAERPK.Y
147	1363 - 1369	371.7140	741.4133	741.4133	0.065	0	32	0.0026	1	U	R.AAAERPK.Y
11992	1370 - 1396	1076.7355	3227.1848	3227.1858	-0.32	0	73	4e-07	1	U	K.YTFDFSEEEDDDADDDDDDNNDLEELK.V
9953	1397 - 1418	805.0323	2412.0751	2412.0734	0.71	1	33	0.0074	1	U	K.VKASPITNDGEDEFVPSDGLDK.D
											+ Phospho (ST)
9954	1397 - 1418	805.0326	2412.0759	2412.0734	1.02	1	35	0.011	1	U	K.VKASPITNDGEDEFVPSDGLDK.D
											+ Phospho (ST)
12420	1397 - 1427	1146.5124	3436.5154	3436.5236	-2.37	2	50	0.00041	1	U	K.VKASPITNDGEDEFVPSDGLDKDEYTFSPGK.S
											+ Phospho (ST)
12421	1397 - 1427	1146.5134	3436.5185	3436.5236	-1.47	2	78	1.3e-06	1	U	K.VKASPITNDGEDEFVPSDGLDKDEYTFSPGK.S
											+ Phospho (ST)
12422	1397 - 1427	860.1370	3436.5188	3436.5236	-1.39	2	50	0.00038	1	U	K.VKASPITNDGEDEFVPSDGLDKDEYTFSPGK.S
											+ Phospho (ST)
12423	1397 - 1427	860.1374	3436.5206	3436.5236	-0.86	2	51	0.00052	1	U	K.VKASPITNDGEDEFVPSDGLDKDEYTFSPGK.S
											+ Phospho (ST)
12424	1397 - 1427	860.1383	3436.5242	3436.5236	0.19	2	38	0.01	1	U	K.VKASPITNDGEDEFVPSDGLDKDEYTFSPGK.S
											+ Phospho (ST)
12425	1397 - 1427	860.1384	3436.5245	3436.5236	0.27	2	42	0.0036	1	U	K.VKASPITNDGEDEFVPSDGLDKDEYTFSPGK.S
											+ Phospho (ST)
12426	1397 - 1427	860.1386	3436.5253	3436.5236	0.51	2	57	0.0003	1	U	K.VKASPITNDGEDEFVPSDGLDKDEYTFSPGK.S
											+ Phospho (ST)
9160	1399 - 1418	1053.4794	2104.9443	2104.9437	0.28	0	89	8.9e-08	1	U	K.ASPITNDGEDEFVPSDGLDK.D
11738	1399 - 1427	783.3542	3129.3878	3129.3939	-1.94	1	29	0.016	1	U	K.ASPITNDGEDEFVPSDGLDKDEYTFSPGK.S
11739	1399 - 1427	1044.1377	3129.3914	3129.3939	-0.80	1	39	0.0046	1	U	K.ASPITNDGEDEFVPSDGLDKDEYTFSPGK.S
11740	1399 - 1427	1044.1379	3129.3920	3129.3939	-0.60	1	52	0.00012	1	U	K.ASPITNDGEDEFVPSDGLDKDEYTFSPGK.S
11742	1399 - 1427	1044.1387	3129.3943	3129.3939	0.15	1	52	0.00025	1	U	K.ASPITNDGEDEFVPSDGLDKDEYTFSPGK.S
11746	1399 - 1427	783.3564	3129.3967	3129.3939	0.89	1	37	0.0062	1	U	K.ASPITNDGEDEFVPSDGLDKDEYTFSPGK.S
11747	1399 - 1427	1044.1396	3129.3971	3129.3939	1.04	1	57	6e-05	1	U	K.ASPITNDGEDEFVPSDGLDKDEYTFSPGK.S
11947	1399 - 1427	1070.7931	3209.3574	3209.3602	-0.88	1	62	2.7e-05	1	U	K.ASPITNDGEDEFVPSDGLDKDEYTFSPGK.S
											+ Phospho (ST)
11948	1399 - 1427	1070.7939	3209.3599	3209.3602	-0.088	1	88	6.7e-08	1	U	K.ASPITNDGEDEFVPSDGLDKDEYTFSPGK.S
											+ Phospho (ST)
11949	1399 - 1427	1070.7948	3209.3624	3209.3602	0.70	1	40	0.0035	1	U	K.ASPITNDGEDEFVPSDGLDKDEYTFSPGK.S
											+ Phospho (ST)
1711	1419 - 1427	522.2377	1042.4608	1042.4607	0.066	0	36	0.00084	1	U	K.DEYTFSPGK.S
8760	1440 - 1456	990.4786	1978.9426	1978.9425	0.070	1	94	9e-09	1	U	K.KSQDFGNLFSFPSYSQK.S
8761	1440 - 1456	660.6550	1978.9431	1978.9425	0.33	1	24	0.028	1	U	K.KSQDFGNLFSFPSYSQK.S
8119	1441 - 1456	926.4304	1850.8463	1850.8475	-0.64	0	53	9.9e-06	1	U	K.SQDFGNLFSFPSYSQK.S
8120	1441 - 1456	926.4307	1850.8468	1850.8475	-0.38	0	91	4.9e-09	1	U	K.SQDFGNLFSFPSYSQK.S
8122	1441 - 1456	926.4308	1850.8471	1850.8475	-0.24	0	21	0.029	1	U	K.SQDFGNLFSFPSYSQK.S
8123	1441 - 1456	617.9563	1850.8472	1850.8475	-0.20	0	26	0.015	1	U	K.SQDFGNLFSFPSYSQK.S
8124	1441 - 1456	926.4309	1850.8472	1850.8475	-0.19	0	91	4.2e-09	1	U	K.SQDFGNLFSFPSYSQK.S
8125	1441 - 1456	926.4310	1850.8475	1850.8475	0.0076	0	74	1.9e-07	1	U	K.SQDFGNLFSFPSYSQK.S
8126	1441 - 1456	617.9565	1850.8476	1850.8475	0.0097	0	57	1.5e-05	1	U	K.SQDFGNLFSFPSYSQK.S
8128	1441 - 1456	926.4312	1850.8479	1850.8475	0.20	0	78	9.7e-08	1	U	K.SQDFGNLFSFPSYSQK.S
8130	1441 - 1456	926.4314	1850.8482	1850.8475	0.39	0	92	2.4e-09	1	U	K.SQDFGNLFSFPSYSQK.S
8131	1441 - 1456	926.4315	1850.8485	1850.8475	0.53	0	57	5.8e-06	1	U	K.SQDFGNLFSFPSYSQK.S
8132	1441 - 1456	926.4316	1850.8485	1850.8475	0.55	0	78	7.1e-08	1	U	K.SQDFGNLFSFPSYSQK.S
8133	1441 - 1456	926.4316	1850.8486	1850.8475	0.59	0	66	3.3e-06	1	U	K.SQDFGNLFSFPSYSQK.S
8559	1441 - 1456	966.4132	1930.8118	1930.8139	-1.07	0	66	2.6e-06	1	U	K.SQDFGNLFSFPSYSQK.S
											+ Phospho (ST)
8560	1441 - 1456	966.4138	1930.8131	1930.8139	-0.38	0	52	2.9e-05	1	U	K.SQDFGNLFSFPSYSQK.S
											+ Phospho (ST)
10836	1457 - 1482	932.4096	2794.2069	2794.2093	-0.87	1	73	8.2e-07	1	U	K.SEDDSAKFDSNEEDSASVFSPSFGLK.Q
10837	1457 - 1482	932.4097	2794.2072	2794.2093	-0.76	1	59	1.9e-05	1	U	K.SEDDSAKFDSNEEDSASVFSPSFGLK.Q
10839	1457 - 1482	932.4101	2794.2084	2794.2093	-0.34	1	35	0.003	1	U	K.SEDDSAKFDSNEEDSASVFSPSFGLK.Q
10840	1457 - 1482	932.4101	2794.2084	2794.2093	-0.34	1	82	1.7e-07	1	U	K.SEDDSAKFDSNEEDSASVFSPSFGLK.Q
10841	1457 - 1482	932.4102	2794.2089	2794.2093	-0.17	1	59	2e-05	1	U	K.SEDDSAKFDSNEEDSASVFSPSFGLK.Q
10842	1457 - 1482	932.4104	2794.2092	2794.2093	-0.040	1	47	0.00026	1	U	K.SEDDSAKFDSNEEDSASVFSPSFGLK.Q
10843	1457 - 1482	932.4104	2794.2094	2794.2093	0.024	1	63	6.5e-06	1	U	K.SEDDSAKFDSNEEDSASVFSPSFGLK.Q
11005	1457 - 1482	959.0658	2874.1756	2874.1757	-0.020	1	47	0.00013	1	U	K.SEDDSAKFDSNEEDSASVFSPSFGLK.Q
											+ Phospho (ST)
11006	1457 - 1482	959.0659	2874.1760	2874.1757	0.11	1	31	0.0072	1	U	K.SEDDSAKFDSNEEDSASVFSPSFGLK.Q
											+ Phospho (ST)
11007	1457 - 1482	959.0659	2874.1760	2874.1757	0.11	1	41	0.0015	1	U	K.SEDDSAKFDSNEEDSASVFSPSFGLK.Q
											+ Phospho (ST)
11008	1457 - 1482	959.0665	2874.1778	2874.1757	0.74	1	61	1.4e-05	1	U	K.SEDDSAKFDSNEEDSASVFSPSFGLK.Q
											+ Phospho (ST)
9009	1464 - 1482	1031.9636	2061.9126	2061.9167	-1.99	0	51	0.00013	1	U	K.FDSNEEDSASVFSPSFGLK.Q
9010	1464 - 1482	1031.9641	2061.9136	2061.9167	-1.53	0	109	8.1e-10	1	U	K.FDSNEEDSASVFSPSFGLK.Q
9012	1464 - 1482	1031.9644	2061.9143	2061.9167	-1.17	0	39	0.00096	1	U	K.FDSNEEDSASVFSPSFGLK.Q
9014	1464 - 1482	1031.9652	2061.9158	2061.9167	-0.47	0	76	3.2e-07	1	U	K.FDSNEEDSASVFSPSFGLK.Q
9015	1464 - 1482	1031.9653	2061.9160	2061.9167	-0.33	0	61	9.9e-06	1	U	K.FDSNEEDSASVFSPSFGLK.Q
9016	1464 - 1482	1031.9655	2061.9165	2061.9167	-0.12	0	100	8.3e-10	1	U	K.FDSNEEDSASVFSPSFGLK.Q
9017	1464 - 1482	688.3128	2061.9167	2061.9167	-0.034	0	52	0.00026	1	U	K.FDSNEEDSASVFSPSFGLK.Q
9018	1464 - 1482	688.3129	2061.9169	2061.9167	0.097	0	30	0.017	1	U	K.FDSNEEDSASVFSPSFGLK.Q
9270	1464 - 1482	1071.9480	2141.8813	2141.8831	-0.80	0	72	1.2e-06	1	U	K.FDSNEEDSASVFSPSFGLK.Q
											+ Phospho (ST)
9271	1464 - 1482	1071.9480	2141.8814	2141.8831	-0.79	0	75	1.2e-06	1	U	K.FDSNEEDSASVFSPSFGLK.Q
											+ Phospho (ST)
9273	1464 - 1482	1071.9482	2141.8818	2141.8831	-0.56	0	67	2.9e-06	1	U	K.FDSNEEDSASVFSPSFGLK.Q
											+ Phospho (ST)
9274	1464 - 1482	1071.9486	2141.8826	2141.8831	-0.22	0	80	6.9e-08	1	U	K.FDSNEEDSASVFSPSFGLK.Q

Query	Start – End	Observed	Mr(expt)	Mr(calc)	ppm	M	Score	Expect	Rank	U	Peptide
											+ Phospho (ST)
9275	1464 – 1482	1071.9488	2141.8831	2141.8831	0.0051	0	65	2.7e-06	1	U	K.FDSNEEDSASVFSPSFGLK.Q + Phospho (ST)
9276	1464 – 1482	1071.9488	2141.8831	2141.8831	0.0051	0	63	2.1e-06	1	U	K.FDSNEEDSASVFSPSFGLK.Q + Phospho (ST)
9277	1464 – 1482	714.9684	2141.8832	2141.8831	0.077	0	45	0.00016	1	U	K.FDSNEEDSASVFSPSFGLK.Q + Phospho (ST)
9278	1464 – 1482	714.9684	2141.8832	2141.8831	0.077	0	27	0.026	1	U	K.FDSNEEDSASVFSPSFGLK.Q + Phospho (ST)
9279	1464 – 1482	1071.9489	2141.8833	2141.8831	0.12	0	64	2.4e-06	1	U	K.FDSNEEDSASVFSPSFGLK.Q + Phospho (ST)
9280	1464 – 1482	714.9684	2141.8834	2141.8831	0.18	0	30	0.0037	1	U	K.FDSNEEDSASVFSPSFGLK.Q + Phospho (ST)
9281	1464 – 1482	714.9689	2141.8849	2141.8831	0.86	0	32	0.0064	1	U	K.FDSNEEDSASVFSPSFGLK.Q + Phospho (ST)
9283	1464 – 1482	714.9690	2141.8853	2141.8831	1.03	0	20	0.013	1	U	K.FDSNEEDSASVFSPSFGLK.Q + Phospho (ST)
9284	1464 – 1482	714.9692	2141.8857	2141.8831	1.21	0	15	0.036	1	U	K.FDSNEEDSASVFSPSFGLK.Q + Phospho (ST)
839	1483 – 1490	451.7506	901.4867	901.4869	-0.25	1	41	0.0044	1	U	K.QTDKVPSK.T
4305	1496 – 1508	684.8958	1367.7771	1367.7773	-0.091	1	59	2.6e-05	1	U	K.KGKPSSDTVPKPK.R
3311	1497 – 1508	414.2347	1239.6823	1239.6823	-0.020	0	50	0.00029	1	U	K.GKPSSDTVPKPK.R
3315	1497 – 1508	620.8492	1239.6839	1239.6823	1.33	0	47	9.5e-05	1	U	K.GKPSSDTVPKPK.R
3316	1497 – 1508	414.2357	1239.6853	1239.6823	2.45	0	30	0.0093	1	U	K.GKPSSDTVPKPK.R
8510	1515 – 1532	640.9893	1919.9462	1919.9476	-0.75	1	69	2.4e-06	1	U	K.KVVEAVNSDSDSEFGIPK.K
8511	1515 – 1532	960.9810	1919.9474	1919.9476	-0.14	1	93	2e-09	1	U	K.KVVEAVNSDSDSEFGIPK.K
8512	1515 – 1532	960.9812	1919.9479	1919.9476	0.12	1	85	1.1e-08	1	U	K.KVVEAVNSDSDSEFGIPK.K
8796	1515 – 1532	1000.9638	1999.9131	1999.9140	-0.40	1	74	1.3e-06	1	U	K.KVVEAVNSDSDSEFGIPK.K + Phospho (ST)
8797	1515 – 1532	667.6450	1999.9132	1999.9140	-0.38	1	26	0.013	1	U	K.KVVEAVNSDSDSEFGIPK.K + Phospho (ST)
8798	1515 – 1532	667.6450	1999.9133	1999.9140	-0.35	1	29	0.017	1	U	K.KVVEAVNSDSDSEFGIPK.K + Phospho (ST)
8799	1515 – 1532	1000.9639	1999.9133	1999.9140	-0.34	1	69	7e-06	1	U	K.KVVEAVNSDSDSEFGIPK.K + Phospho (ST)
8800	1515 – 1532	1000.9640	1999.9134	1999.9140	-0.25	1	42	0.00028	1	U	K.KVVEAVNSDSDSEFGIPK.K + Phospho (ST)
8802	1515 – 1532	667.6454	1999.9144	1999.9140	0.21	1	39	0.0012	1	U	K.KVVEAVNSDSDSEFGIPK.K + Phospho (ST)
8803	1515 – 1532	1000.9646	1999.9146	1999.9140	0.31	1	59	7e-06	1	U	K.KVVEAVNSDSDSEFGIPK.K + Phospho (ST)
7665	1516 – 1532	896.9336	1791.8527	1791.8527	0.040	0	77	1.2e-07	1	U	K.VVEAVNSDSDSEFGIPK.K
7666	1516 – 1532	598.2916	1791.8529	1791.8527	0.15	0	37	0.00055	1	U	K.VVEAVNSDSDSEFGIPK.K
7667	1516 – 1532	896.9340	1791.8534	1791.8527	0.42	0	58	4.6e-06	1	U	K.VVEAVNSDSDSEFGIPK.K
7668	1516 – 1532	896.9343	1791.8541	1791.8527	0.81	0	51	1.7e-05	1	U	K.VVEAVNSDSDSEFGIPK.K
7669	1516 – 1532	896.9350	1791.8555	1791.8527	1.59	0	40	0.00024	1	U	K.VVEAVNSDSDSEFGIPK.K
7671	1516 – 1532	896.9364	1791.8583	1791.8527	3.14	0	45	0.0011	1	U	K.VVEAVNSDSDSEFGIPK.K
8232	1516 – 1532	936.9158	1871.8171	1871.8190	-0.99	0	64	9.4e-07	1	U	K.VVEAVNSDSDSEFGIPK.K + Phospho (ST)
8233	1516 – 1532	936.9159	1871.8173	1871.8190	-0.90	0	56	9.9e-06	1	U	K.VVEAVNSDSDSEFGIPK.K + Phospho (ST)
8234	1516 – 1532	936.9168	1871.8190	1871.8190	0.015	0	73	3.1e-07	1	U	K.VVEAVNSDSDSEFGIPK.K + Phospho (ST)
8235	1516 – 1532	936.9168	1871.8191	1871.8190	0.068	0	81	7.7e-08	1	U	K.VVEAVNSDSDSEFGIPK.K + Phospho (ST)
8236	1516 – 1532	936.9170	1871.8194	1871.8190	0.21	0	69	3.8e-07	1	U	K.VVEAVNSDSDSEFGIPK.K + Phospho (ST)
7880	1547 – 1562	606.2587	1815.7543	1815.7537	0.36	2	53	0.00058	1	U	K.RKASGSENEGDYNPGR.K + Phospho (ST)
6105	1548 – 1562	527.5693	1579.6861	1579.6862	-0.074	1	29	0.016	1	U	R.KASGSENEGDYNPGR.K
6107	1548 – 1562	527.5694	1579.6864	1579.6862	0.078	1	52	0.00017	1	U	R.KASGSENEGDYNPGR.K
6108	1548 – 1562	790.8505	1579.6864	1579.6862	0.088	1	52	8.2e-05	1	U	R.KASGSENEGDYNPGR.K
6645	1548 – 1562	554.2248	1659.6525	1659.6526	-0.055	1	38	0.0065	1	U	R.KASGSENEGDYNPGR.K + Phospho (ST)
6646	1548 – 1562	830.8337	1659.6529	1659.6526	0.20	1	61	6.2e-06	1	U	R.KASGSENEGDYNPGR.K + Phospho (ST)
6647	1548 – 1562	554.2250	1659.6531	1659.6526	0.31	1	22	0.042	1	U	R.KASGSENEGDYNPGR.K + Phospho (ST)
6648	1548 – 1562	554.2254	1659.6544	1659.6526	1.08	1	31	0.0053	1	U	R.KASGSENEGDYNPGR.K + Phospho (ST)
7278	1548 – 1562	870.8170	1739.6195	1739.6189	0.33	1	22	0.044	1	U	R.KASGSENEGDYNPGR.K + 2 Phospho (ST)
7279	1548 – 1562	870.8174	1739.6202	1739.6189	0.74	1	19	0.025	1	U	R.KASGSENEGDYNPGR.K + 2 Phospho (ST)
7037	1548 – 1563	570.2675	1707.7807	1707.7812	-0.28	2	54	5.8e-05	1	U	R.KASGSENEGDYNPGRK.T
7039	1548 – 1563	854.8985	1707.7824	1707.7812	0.71	2	52	7.8e-05	1	U	R.KASGSENEGDYNPGRK.T
7609	1548 – 1563	596.9233	1787.7480	1787.7475	0.27	2	37	0.00099	1	U	R.KASGSENEGDYNPGRK.T + Phospho (ST)
4870	1549 – 1562	726.8008	1451.5871	1451.5913	-2.90	0	60	1.8e-05	1	U	K.ASGSENEGDYNPGR.K
4871	1549 – 1562	726.8028	1451.5911	1451.5913	-0.11	0	46	0.00014	1	U	K.ASGSENEGDYNPGR.K
4872	1549 – 1562	726.8029	1451.5912	1451.5913	-0.031	0	53	3.5e-05	1	U	K.ASGSENEGDYNPGR.K
4873	1549 – 1562	484.8712	1451.5917	1451.5913	0.25	0	42	0.0011	1	U	K.ASGSENEGDYNPGR.K
4874	1549 – 1562	726.8032	1451.5917	1451.5913	0.31	0	60	2.2e-05	1	U	K.ASGSENEGDYNPGR.K
4875	1549 – 1562	726.8032	1451.5918	1451.5913	0.35	0	37	0.0061	1	U	K.ASGSENEGDYNPGR.K
4876	1549 – 1562	484.8714	1451.5925	1451.5913	0.85	0	30	0.014	1	U	K.ASGSENEGDYNPGR.K
4877	1549 – 1562	726.8041	1451.5936	1451.5913	1.57	0	42	0.0013	1	U	K.ASGSENEGDYNPGR.K
5700	1549 – 1562	766.7839	1531.5532	1531.5576	-2.90	0	56	8.1e-05	1	U	K.ASGSENEGDYNPGR.K + Phospho (ST)
5701	1549 – 1562	766.7854	1531.5562	1531.5576	-0.92	0	67	6.9e-06	1	U	K.ASGSENEGDYNPGR.K + Phospho (ST)
5702	1549 – 1562	766.7862	1531.5579	1531.5576	0.20	0	35	0.0058	1	U	K.ASGSENEGDYNPGR.K + Phospho (ST)
5703	1549 – 1562	766.7862	1531.5579	1531.5576	0.20	0	55	0.00011	1	U	K.ASGSENEGDYNPGR.K + Phospho (ST)
6106	1549 – 1563	790.8504	1579.6863	1579.6862	0.025	1	71	3.7e-06	1	U	K.ASGSENEGDYNPGRK.T

Query	Start - End	Observed	Mr(expt)	Mr(calc)	ppm	M	Score	Expect	Rank	U	Peptide
6649	1549 - 1563	830.8345	1659.6544	1659.6526	1.11	1	33	0.019	1	U	K.ASGSENEGDYNPGRK.T + Phospho (ST)
11158	1574 - 1599	979.7967	2936.3683	2936.3716	-1.13	1	24	0.021	1	U	K.KTSFDQDSDVDIFPSDFPTEPPSLPR.T
11162	1574 - 1599	588.2813	2936.3701	2936.3716	-0.53	1	30	0.028	1	U	K.KTSFDQDSDVDIFPSDFPTEPPSLPR.T
11163	1574 - 1599	979.7974	2936.3705	2936.3716	-0.38	1	45	0.00096	1	U	K.KTSFDQDSDVDIFPSDFPTEPPSLPR.T
11164	1574 - 1599	979.7975	2936.3707	2936.3716	-0.32	1	29	0.048	1	U	K.KTSFDQDSDVDIFPSDFPTEPPSLPR.T
11167	1574 - 1599	735.1001	2936.3714	2936.3716	-0.086	1	32	0.019	1	U	K.KTSFDQDSDVDIFPSDFPTEPPSLPR.T
11168	1574 - 1599	979.7978	2936.3716	2936.3716	-0.0089	1	49	0.00057	1	U	K.KTSFDQDSDVDIFPSDFPTEPPSLPR.T
11169	1574 - 1599	979.7978	2936.3716	2936.3716	0.0014	1	41	0.00086	1	U	K.KTSFDQDSDVDIFPSDFPTEPPSLPR.T
11170	1574 - 1599	588.2816	2936.3719	2936.3716	0.082	1	32	0.019	1	U	K.KTSFDQDSDVDIFPSDFPTEPPSLPR.T
11173	1574 - 1599	979.7981	2936.3725	2936.3716	0.31	1	32	0.012	1	U	K.KTSFDQDSDVDIFPSDFPTEPPSLPR.T
11174	1574 - 1599	979.7984	2936.3733	2936.3716	0.55	1	31	0.012	1	U	K.KTSFDQDSDVDIFPSDFPTEPPSLPR.T
10874	1575 - 1599	937.0986	2808.2741	2808.2767	-0.91	0	38	0.0014	1	U	K.TSFDQDSDVDIFPSDFPTEPPSLPR.T
10875	1575 - 1599	937.0991	2808.2754	2808.2767	-0.44	0	48	0.00031	1	U	K.TSFDQDSDVDIFPSDFPTEPPSLPR.T
10877	1575 - 1599	937.0993	2808.2760	2808.2767	-0.25	0	28	0.011	1	U	K.TSFDQDSDVDIFPSDFPTEPPSLPR.T
10878	1575 - 1599	937.0994	2808.2765	2808.2767	-0.064	0	48	0.00011	1	U	K.TSFDQDSDVDIFPSDFPTEPPSLPR.T
10880	1575 - 1599	937.0996	2808.2771	2808.2767	0.14	0	26	0.015	1	U	K.TSFDQDSDVDIFPSDFPTEPPSLPR.T
10883	1575 - 1599	937.1000	2808.2782	2808.2767	0.53	0	45	0.00024	1	U	K.TSFDQDSDVDIFPSDFPTEPPSLPR.T
9376	1609 - 1626	1086.9145	2171.8145	2171.8154	-0.41	0	18	0.041	1	U	K.YFAESDEEEDDVDFAMFN.-
9379	1609 - 1626	1086.9148	2171.8150	2171.8154	-0.18	0	37	0.0004	1	U	K.YFAESDEEEDDVDFAMFN.-
9380	1609 - 1626	724.9456	2171.8151	2171.8154	-0.11	0	45	0.00052	1	U	K.YFAESDEEEDDVDFAMFN.-
9428	1609 - 1626	1094.9116	2187.8086	2187.8103	-0.78	0	28	0.0035	1	U	K.YFAESDEEEDDVDFAMFN.- + Oxidation (M)
9429	1609 - 1626	1094.9122	2187.8098	2187.8103	-0.20	0	57	1.1e-05	1	U	K.YFAESDEEEDDVDFAMFN.- + Oxidation (M)
9430	1609 - 1626	1094.9123	2187.8101	2187.8103	-0.084	0	38	0.00039	1	U	K.YFAESDEEEDDVDFAMFN.- + Oxidation (M)
9432	1609 - 1626	1094.9129	2187.8113	2187.8103	0.46	0	30	0.0045	1	U	K.YFAESDEEEDDVDFAMFN.- + Oxidation (M)
9433	1609 - 1626	1094.9130	2187.8115	2187.8103	0.56	0	56	6.3e-06	1	U	K.YFAESDEEEDDVDFAMFN.- + Oxidation (M)
9434	1609 - 1626	1094.9133	2187.8120	2187.8103	0.80	0	46	5.1e-05	1	U	K.YFAESDEEEDDVDFAMFN.- + Oxidation (M)

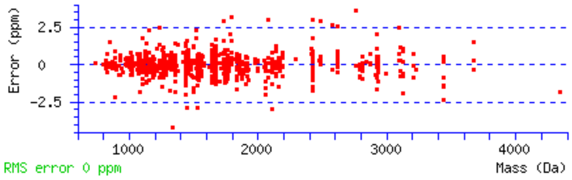

Supplement: Supplementary file 8 — Supplementary Data 5 [file 41467_2023_44089_MOESM8_ESM.pdf]
