# Supplementary material for: ERK2-topoisomerase II regulatory axis is important for gene activation in immediate early genes
Source: Nat Commun. 2023 Dec 14;14:8341. doi: 10.1038/s41467-023-44089-y (PMC10721843; doi:10.1038/s41467-023-44089-y)
Supplement: Supplementary file 9 — Supplementary Data 6 [file 41467_2023_44089_MOESM9_ESM.pdf]

Erk1_S1336, S1340, S1344

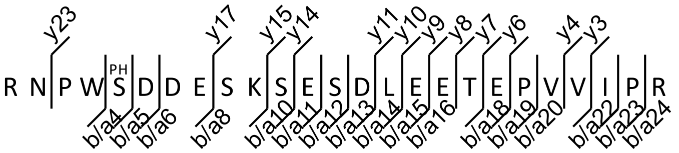

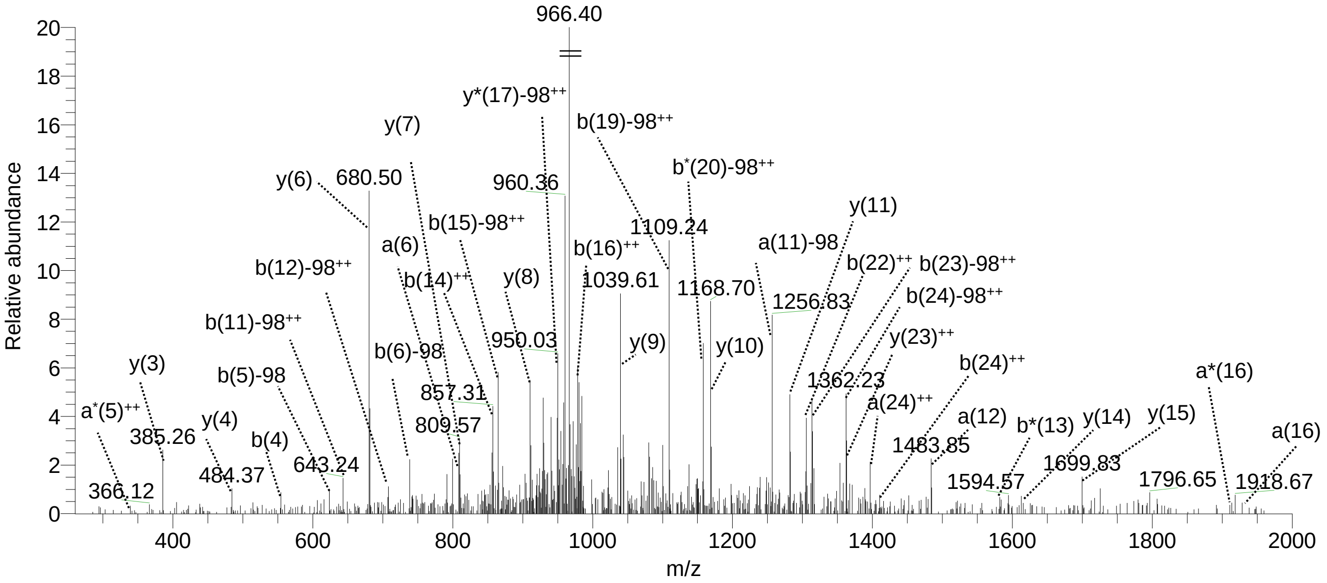

Match to Query 12003: 2993.331402 from(998.784410,3+) intensity(19394.5762) rtinseconds(2576) rawfile(SN221363_deTOP2B_Erk1_1ul.raw) scans(8614) index(6914)

Monoisotopic mass of neutral peptide Mr(calc): 2993.3291

Fixed modifications: Carbamidomethyl (C) (apply to specified residues or termini only)

Variable modifications: S5 : Phospho (ST), with neutral losses 97.9769(shown in table), 0.0000

Ions Score: 52 **Expect:** 0.00025 **Matches :** 74/464 fragment ions using 85 most intense peaks

#	a	a++	a*	a*++	b	b++	b*	b*++	Seq.	y	y++	y*	y*++	#
1	129.11	65.06	112.09	56.55	157.11	79.06	140.08	70.54	R					25
2	243.16	122.08	226.13	113.57	271.15	136.08	254.12	127.57	N	2740.26	1370.63	2723.23	1362.12	24
3	340.21	170.61	323.18	162.09	368.20	184.61	351.18	176.09	P	2626.22	1313.61	2609.19	1305.10	23
4	526.29	263.65	509.26	255.13	554.28	277.65	537.26	269.13	W	2529.16	1265.09	2512.14	1256.57	22
5	595.31	298.16	578.28	289.65	623.30	312.16	606.28	303.64	S	2343.08	1172.05	2326.06	1163.53	21
6	710.34	355.67	693.31	347.16	738.33	369.67	721.31	361.16	D	2274.06	1137.53	2257.04	1129.02	20
7	825.36	413.19	808.34	404.67	853.36	427.18	836.33	418.67	D	2159.04	1080.02	2142.01	1071.51	19
8	954.41	477.71	937.38	469.19	982.40	491.70	965.37	483.19	E	2044.01	1022.51	2026.98	1013.99	18
9	1041.44	521.22	1024.41	512.71	1069.43	535.22	1052.41	526.71	S	1914.97	957.99	1897.94	949.47	17
10	1169.53	585.27	1152.51	576.76	1197.53	599.27	1180.50	590.75	K	1827.93	914.47	1810.91	905.96	16
11	1256.57	628.79	1239.54	620.27	1284.56	642.78	1267.53	634.27	S	1699.84	850.42	1682.81	841.91	15
12	1385.61	693.31	1368.58	684.79	1413.60	707.31	1396.58	698.79	E	1612.81	806.91	1595.78	798.39	14
13	1472.64	736.82	1455.61	728.31	1500.64	750.82	1483.61	742.31	S	1483.76	742.39	1466.74	733.87	13
14	1587.67	794.34	1570.64	785.82	1615.66	808.33	1598.64	799.82	D	1396.73	698.87	1379.71	690.36	12
15	1700.75	850.88	1683.72	842.37	1728.75	864.88	1711.72	856.36	L	1281.70	641.36	1264.68	632.84	11
16	1829.79	915.40	1812.77	906.89	1857.79	929.40	1840.76	920.88	E	1168.62	584.81	1151.59	576.30	10
17	1958.84	979.92	1941.81	971.41	1986.83	993.92	1969.80	985.41	E	1039.58	520.29	1022.55	511.78	9
18	2059.88	1030.45	2042.86	1021.93	2087.88	1044.44	2070.85	1035.93	T	910.54	455.77	893.51	447.26	8
19	2188.93	1094.97	2171.90	1086.45	2216.92	1108.96	2199.89	1100.45	E	809.49	405.25	792.46	396.73	7
20	2285.98	1143.49	2268.95	1134.98	2313.97	1157.49	2296.95	1148.98	P	680.45	340.73	663.42	332.21	6
21	2385.05	1193.03	2368.02	1184.51	2413.04	1207.02	2396.02	1198.51	V	583.39	292.20	566.37	283.69	5
22	2484.12	1242.56	2467.09	1234.05	2512.11	1256.56	2495.08	1248.05	V	484.32	242.67	467.30	234.15	4
23	2597.20	1299.10	2580.17	1290.59	2625.20	1313.10	2608.17	1304.59	I	385.26	193.13	368.23	184.62	3
24	2694.25	1347.63	2677.23	1339.12	2722.25	1361.63	2705.22	1353.11	P	272.17	136.59	255.15	128.08	2
25									R	175.12	88.06	158.09	79.55	1

All matches to this query

Score	Mr(calc)	Delta	Site Analysis
52.4	2993.329	0.0023	Phospho S5 82.30%
44.9	2993.329	0.0023	Phospho S9 14.60%
37.6	2993.329	0.0023	Phospho S11 2.71%
29.1	2993.329	0.0023	Phospho S13 0.38%
6.9	2993.329	0.0023	Phospho T18 0.00%

Erk1_S1336, S1340, S1344

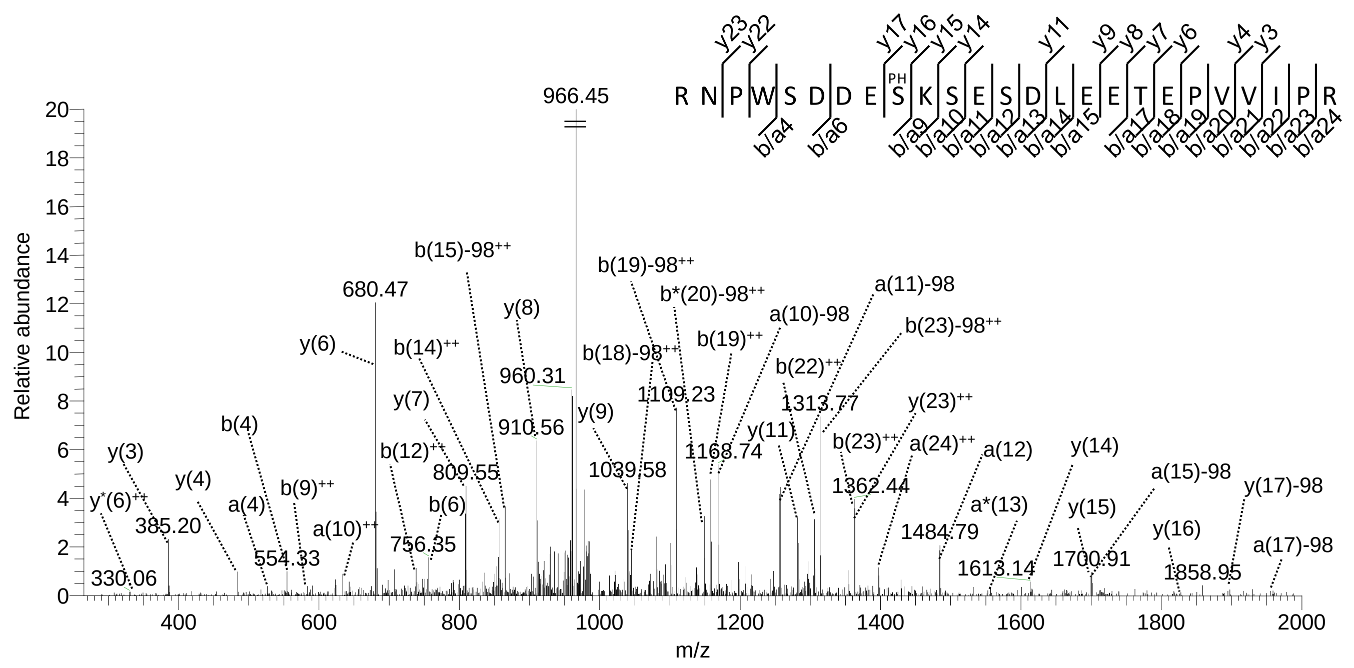

Match to Query 12001: 2993.325072 from(998.782300,3+) intensity(15240.2246) rtinseconds(2551) rawfile(SN221363_deTOP2B_Erk1_1ul.raw) scans(8507) index(6815)

Monoisotopic mass of neutral peptide Mr(calc): 2993.3291

Fixed modifications: Carbamidomethyl (C) (apply to specified residues or termini only)

Variable modifications: S9 : Phospho (ST), with neutral losses 97.9769(shown in table), 0.0000

Ions Score: 67 **Expect:** 4.3e-06 **Matches :** 59/448 fragment ions using 68 most intense peaks

#	a	a^{++}	a*	a^{*++}	b	b^{++}	b*	b^{*++}	Seq.	y	y^{++}	y*	y^{*++}	#
1	129.11	65.06	112.09	56.55	157.11	79.06	140.08	70.54	R					25
2	243.16	122.08	226.13	113.57	271.15	136.08	254.12	127.57	N	2740.26	1370.63	2723.23	1362.12	24
3	340.21	170.61	323.18	162.09	368.20	184.61	351.18	176.09	P	2626.22	1313.61	2609.19	1305.10	23
4	**526.29**	263.65	509.26	255.13	554.28	277.65	537.26	269.13	W	2529.16	1265.09	2512.14	1256.57	22
5	613.32	307.16	596.29	298.65	641.32	321.16	624.29	312.65	S	2343.08	1172.05	2326.06	1163.53	21
6	728.35	364.68	711.32	356.16	756.34	378.67	739.32	370.16	D	2256.05	1128.53	2239.02	1120.02	20
7	843.37	422.19	826.35	413.68	871.37	436.19	854.34	427.68	D	2141.02	1071.02	2124.00	1062.50	19
8	972.42	486.71	955.39	478.20	1000.41	500.71	983.39	492.20	E	2026.00	1013.50	2008.97	1004.99	18
9	**1041.44**	521.22	1024.41	512.71	1069.43	535.22	1052.41	526.71	S	***1896.95***	948.98	1879.93	940.47	17
10	**1169.53**	585.27	1152.51	576.76	1197.53	599.27	1180.50	590.75	K	***1827.93***	914.47	1810.91	905.96	16
11	**1256.57**	628.79	1239.54	620.27	1284.56	642.78	1267.53	634.27	S	***1699.84***	850.42	1682.81	841.91	15
12	1385.61	693.31	1368.58	684.79	1413.60	**707.31**	1396.58	698.79	E	***1612.81***	806.91	1595.78	798.39	14
13	1472.64	736.82	1455.61	728.31	1500.64	750.82	1483.61	742.31	S	***1483.76***	742.39	1466.74	733.87	13
14	1587.67	794.34	1570.64	785.82	1615.66	**808.33**	1598.64	799.82	D	***1396.73***	698.87	1379.71	690.36	12
15	**1700.75**	850.88	1683.72	842.37	1728.75	**864.88**	1711.72	856.36	L	***1281.70***	641.36	1264.68	632.84	11
16	1829.79	915.40	1812.77	906.89	1857.79	929.40	1840.76	920.88	E	***1168.62***	584.81	1151.59	576.30	10
17	**1958.84**	979.92	1941.81	971.41	1986.83	993.92	1969.80	985.41	E	***1039.58***	520.29	1022.55	511.78	9
18	2059.88	1030.45	2042.86	1021.93	2087.88	**1044.44**	2070.85	1035.93	T	***910.54***	455.77	893.51	447.26	8
19	2188.93	1094.97	2171.90	1086.45	2216.92	**1108.96**	2199.89	1100.45	E	***809.49***	405.25	792.46	396.73	7
20	2285.98	1143.49	2268.95	1134.98	2313.97	1157.49	2296.95	1148.98	P	***680.45***	340.73	663.42	332.21	6
21	2385.05	1193.03	2368.02	1184.51	2413.04	1207.02	2396.02	1198.51	V	***583.39***	292.20	566.37	283.69	5
22	2484.12	1242.56	2467.09	1234.05	2512.11	**1256.56**	2495.08	1248.05	V	***484.32***	242.67	467.30	234.15	4
23	2597.20	1299.10	2580.17	1290.59	2625.20	**1313.10**	2608.17	1304.59	I	***385.26***	193.13	368.23	184.62	3
24	2694.25	1347.63	2677.23	1339.12	2722.25	1361.63	2705.22	1353.11	P	272.17	136.59	255.15	128.08	2
25									R	175.12	88.06	158.09	79.55	1

All matches to this query

Score	Mr(calc)	Delta	Site Analysis
66.6	2993.329	-0.004	Phospho S9 78.41%
59.9	2993.329	-0.004	Phospho S5 16.88%
54.3	2993.329	-0.004	Phospho S11 4.62%
37.3	2993.329	-0.004	Phospho S13 0.09%
11.6	2993.329	-0.004	Phospho T18 0.00%

Erk1_S1336, S1340, S1344

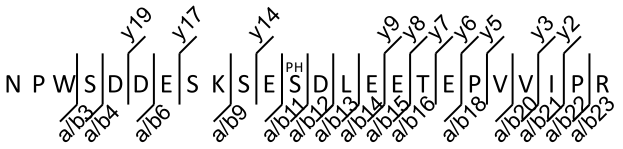

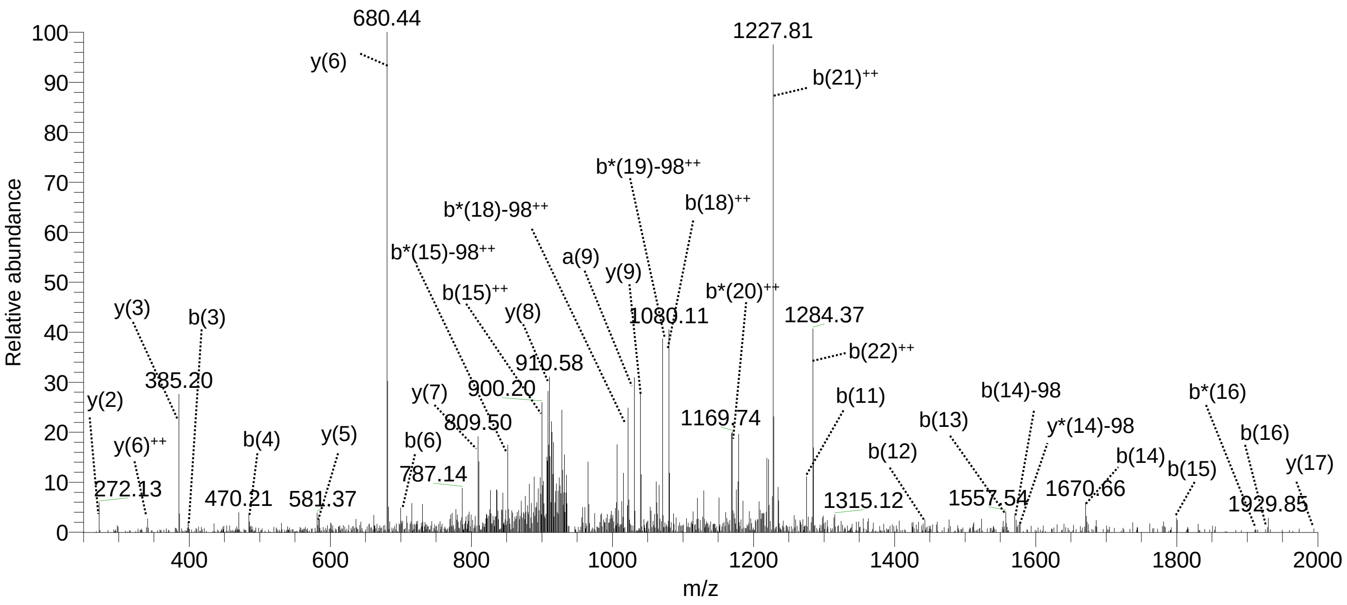

Match to Query 11588: 2837.230062 from(946.750630,3+) intensity(15514.4561) rtinseconds(2595) rawfile(SN221363_deTOP2B_Erk1_1ul.raw) scans(8693) index(6987)

Monoisotopic mass of neutral peptide Mr(calc): 2837.2280

Fixed modifications: Carbamidomethyl (C) (apply to specified residues or termini only)

Variable modifications: S12 : Phospho (ST), with neutral losses 0.0000(shown in table), 97.9769

Ions Score: 60 **Expect:** 7e-05 **Matches :** 43/416 fragment ions using 53 most intense peaks

#	a	a++	a*	a*++	b	b++	b*	b*++	Seq.	y	y++	y*	y*++	#
1	87.06	44.03	70.03	35.52	115.05	58.03	98.02	49.52	N					24
2	184.11	92.56	167.08	84.04	212.10	106.56	195.08	98.04	P	2724.19	1362.60	2707.17	1354.09	23
3	370.19	185.60	353.16	177.08	***398.18***	199.59	381.16	191.08	W	2627.14	1314.07	2610.11	1305.56	22
4	457.22	229.11	440.19	220.60	***485.21***	243.11	468.19	234.60	S	2441.06	1221.03	2424.03	1212.52	21
5	572.25	286.63	555.22	278.11	600.24	300.62	583.21	292.11	D	2354.03	1177.52	2337.00	1169.00	20
6	687.27	344.14	670.25	335.63	***715.27***	358.14	698.24	349.62	D	2239.00	1120.00	2221.97	1111.49	19
7	816.32	408.66	799.29	400.15	844.31	422.66	827.28	414.15	E	2123.97	1062.49	2106.95	1053.98	18
8	903.35	452.18	886.32	443.66	931.34	466.18	914.32	457.66	S	***1994.93***	997.97	1977.91	989.46	17
9	1031.44	516.23	1014.42	507.71	1059.44	530.22	1042.41	521.71	K	1907.90	954.45	1890.87	945.94	16
10	1118.47	559.74	1101.45	551.23	1146.47	573.74	1129.44	565.23	S	1779.80	890.41	1762.78	881.89	15
11	1247.52	624.26	1230.49	615.75	***1275.51***	638.26	1258.49	629.75	E	1692.77	846.89	1675.75	838.38	14
12	1414.52	707.76	1397.49	699.25	***1442.51***	721.76	1425.48	713.25	S	1563.73	782.37	1546.70	773.86	13
13	1529.54	765.28	1512.52	756.76	***1557.54***	779.27	1540.51	770.76	D	1396.73	698.87	1379.71	690.36	12
14	1642.63	821.82	1625.60	813.30	***1670.62***	835.81	1653.60	827.30	L	1281.70	641.36	1264.68	632.84	11
15	1771.67	886.34	1754.64	877.83	***1799.66***	**900.34**	1782.64	891.82	E	1168.62	584.81	1151.59	576.30	10
16	1900.71	950.86	1883.69	942.35	***1928.71***	964.86	1911.68	956.34	E	***1039.58***	520.29	1022.55	511.78	9
17	2001.76	1001.38	1984.73	992.87	2029.75	1015.38	2012.73	1006.87	T	***910.54***	455.77	893.51	447.26	8
18	2130.80	1065.90	2113.78	1057.39	2158.80	**1079.90**	2141.77	1071.39	E	***809.49***	405.25	792.46	396.73	7
19	2227.86	1114.43	2210.83	1105.92	2255.85	1128.43	2238.82	1119.92	P	***680.45***	340.73	663.42	332.21	6
20	2326.92	1163.97	2309.90	1155.45	2354.92	1177.96	2337.89	1169.45	V	***583.39***	292.20	566.37	283.69	5
21	2425.99	1213.50	2408.97	1204.99	2453.99	**1227.50**	2436.96	1218.98	V	***484.32***	242.67	467.30	234.15	4
22	2539.08	1270.04	2522.05	1261.53	2567.07	**1284.04**	2550.04	1275.53	I	***385.26***	193.13	368.23	184.62	3
23	2636.13	1318.57	2619.10	1310.05	2664.12	1332.57	2647.10	1324.05	P	***272.17***	136.59	255.15	128.08	2
24									R	175.12	88.06	158.09	79.55	1

All matches to this query

Score	Mr(calc)	Delta	Site Analysis
60.3	2837.228	0.002	Phospho S12 67.34%
54.1	2837.228	0.002	Phospho S10 16.15%
54.1	2837.228	0.002	Phospho S8 16.15%
37	2837.228	0.002	Phospho S4 0.31%
27.7	2837.228	0.002	Phospho T17 0.04%

Erk1m_ S1476

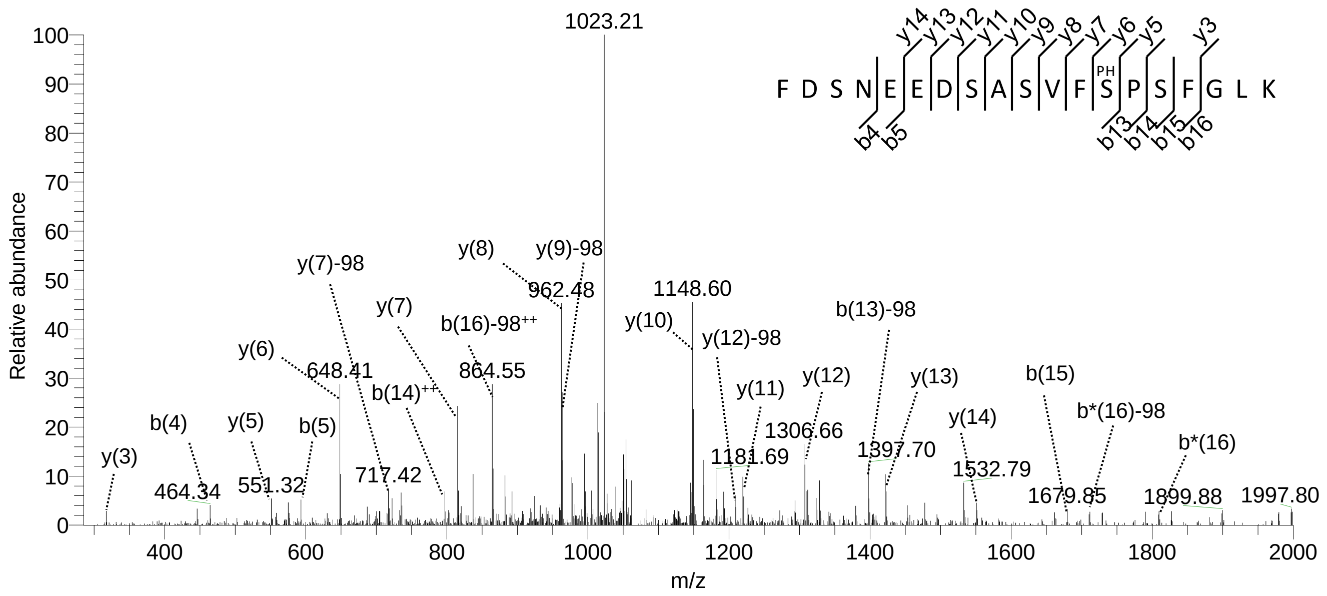

Match to Query 9544: 2141.881308 from(1071.947930,2+) intensity(22847.6758) rtinseconds(3249) rawfile(SN221365_deTOP2B_Erk1ml_1ul.raw) scans(11219) index(9272)

Monoisotopic mass of neutral peptide Mr(calc): 2141.8831
Fixed modifications: Carbamidomethyl (C) (apply to specified residues or termini only)
Variable modifications: S13 : Phospho (ST), with neutral losses 0.0000(shown in table), 97.9769
Ions Score: 82 **Expect:** 1.5e-07 **Matches :** 25/300 fragment ions using 34 most intense peaks

#	a	a++	a*	a*++	b	b++	b*	b*++	Seq.	y	y++	y*	y*++	#
1	120.1	60.5			148.1	74.5			F					19
2	235.1	118.1			263.1	132.1			D	1995.8	998.4	1978.8	989.9	18
3	322.1	161.6			350.1	175.6			S	1880.8	940.9	1863.8	932.4	17
4	436.2	218.6	419.2	210.1	**464.2**	232.6	447.2	224.1	N	1793.8	897.4	1776.7	888.9	16
5	565.2	283.1	548.2	274.6	**593.2**	297.1	576.2	288.6	E	***1679.7***	840.4	1662.7	831.9	15
6	694.3	347.6	677.2	339.1	722.3	361.6	705.2	353.1	E	***1550.7***	775.8	1533.7	767.3	14
7	809.3	405.2	792.3	396.6	837.3	419.1	820.3	410.6	D	***1421.6***	711.3	1404.6	702.8	13
8	896.3	448.7	879.3	440.2	924.3	462.7	907.3	454.2	S	***1306.6***	653.8	1289.6	645.3	12
9	967.4	484.2	950.3	475.7	995.4	498.2	978.3	489.7	A	***1219.6***	610.3	1202.5	601.8	11
10	1054.4	527.7	1037.4	519.2	1082.4	541.7	1065.4	533.2	S	***1148.5***	574.8	1131.5	566.3	10
11	1153.5	577.2	1136.4	568.7	1181.5	591.2	1164.4	582.7	V	1061.5	531.3	1044.5	522.7	9
12	1300.5	650.8	1283.5	642.3	1328.5	664.8	1311.5	656.3	F	***962.4***	481.7	945.4	473.2	8
13	1467.5	734.3	1450.5	725.8	1495.5	748.3	1478.5	739.8	S	***815.4***	408.2	798.3	399.7	7
14	1564.6	782.8	1547.6	774.3	1592.6	796.8	1575.6	788.3	P	***648.4***	324.7	631.3	316.2	6
15	1651.6	826.3	1634.6	817.8	**1679.6**	840.3	1662.6	831.8	S	***551.3***	276.2	534.3	267.6	5
16	1798.7	899.8	1781.7	891.3	1826.7	913.8	1809.7	905.3	F	***464.3***	232.6	447.3	224.1	4
17	1855.7	928.4	1838.7	919.8	1883.7	942.4	1866.7	933.8	G	***317.2***	159.1	300.2	150.6	3
18	1968.8	984.9	1951.8	976.4	1996.8	998.9	1979.8	990.4	L	260.2	130.6	243.2	122.1	2
19									K	147.1	74.1	130.1	65.5	1

All matches to this query

Score	Mr(calc)	Delta	Site Analysis
81.6	2141.8831	-0.0017	Phospho S13 99.26%
59	2141.8831	-0.0017	Phospho S10 0.55%
54.2	2141.8831	-0.0017	Phospho S15 0.18%
38.2	2141.8831	-0.0017	Phospho S8 0.00%
24.8	2141.8831	-0.0017	Phospho S3 0.00%

Erk2_S1336, S1340, S1344

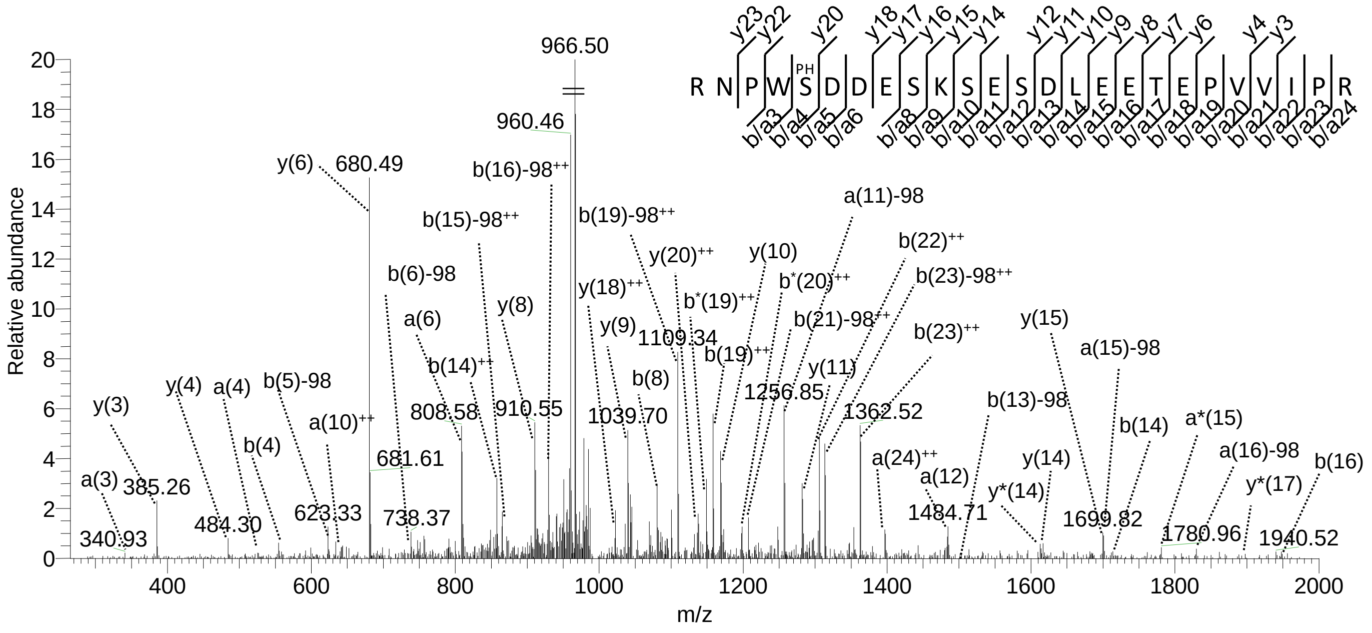

Match to Query 11699: 2993.329722 from(998.783850,3+) intensity(20973.7480) rtinseconds(2565) rawfile(SN221364_deTOP2B_Erk2_1ul.raw) scans(8366) index(6632)

Monoisotopic mass of neutral peptide Mr(calc): 2993.3291

Fixed modifications: Carbamidomethyl (C) (apply to specified residues or termini only)

Variable modifications: S5 : Phospho (ST), with neutral losses 0.0000(shown in table), 97.9769

Ions Score: 63 **Expect:** 1e-05 **Matches :** 112/464 fragment ions using 153 most intense peaks

#	a	a++	a*	a*++	b	b++	b*	b*++	Seq.	y	y++	y*	y*++	#
1	129.1	65.1	112.1	56.5	157.1	79.1	140.1	70.5	R					25
2	243.2	122.1	226.1	113.6	271.2	136.1	254.1	127.6	N	2838.2	1419.6	2821.2	1411.1	24
3	**340.2**	170.6	323.2	162.1	**368.2**	184.6	351.2	176.1	P	2724.2	***1362.6***	2707.2	1354.1	23
4	**526.3**	263.6	509.3	255.1	**554.3**	277.6	537.3	269.1	W	2627.1	***1314.1***	2610.1	1305.6	22
5	693.3	347.1	676.3	338.6	721.3	361.1	704.3	352.6	S	2441.1	1221.0	2424.0	1212.5	21
6	**808.3**	404.7	791.3	396.1	836.3	418.7	819.3	410.1	D	2274.1	***1137.5***	2257.0	1129.0	20
7	923.3	462.2	906.3	453.7	951.3	476.2	934.3	467.7	D	2159.0	***1080.0***	2142.0	1071.5	19
8	1052.4	526.7	1035.4	518.2	**1080.4**	540.7	1063.4	532.2	E	2044.0	***1022.5***	2027.0	1014.0	18
9	1139.4	570.2	1122.4	561.7	1167.4	**584.2**	1150.4	575.7	S	1915.0	***958.0***	1897.9	949.5	17
10	1267.5	634.3	1250.5	625.7	1295.5	**648.3**	1278.5	639.7	K	***1827.9***	914.5	1810.9	906.0	16
11	**1354.5**	677.8	1337.5	669.3	1382.5	691.8	1365.5	683.3	S	***1699.8***	850.4	1682.8	841.9	15
12	**1483.6**	742.3	1466.6	733.8	1511.6	**756.3**	1494.6	747.8	E	***1612.8***	806.9	1595.8	798.4	14
13	1570.6	785.8	1553.6	777.3	1598.6	799.8	1581.6	791.3	S	***1483.8***	742.4	1466.7	733.9	13
14	1685.6	843.3	1668.6	834.8	**1713.6**	**857.3**	1696.6	848.8	D	***1396.7***	***698.9***	1379.7	690.4	12
15	**1798.7**	899.9	1781.7	891.4	1826.7	913.9	1809.7	905.4	L	***1281.7***	***641.4***	1264.7	632.8	11
16	1927.8	964.4	1910.7	955.9	**1955.8**	**978.4**	1938.7	969.9	E	***1168.6***	***584.8***	1151.6	576.3	10
17	2056.8	1028.9	2039.8	1020.4	2084.8	**1042.9**	2067.8	1034.4	E	***1039.6***	520.3	1022.6	511.8	9
18	2157.9	1079.4	2140.8	1070.9	2185.9	1093.4	2168.8	1084.9	T	***910.5***	455.8	893.5	447.3	8
19	2286.9	1144.0	2269.9	1135.4	2314.9	**1158.0**	2297.9	1149.4	E	***809.5***	405.2	792.5	396.7	7
20	2384.0	1192.5	2366.9	1184.0	2412.0	1206.5	2394.9	1198.0	P	***680.4***	***340.7***	663.4	332.2	6
21	2483.0	1242.0	2466.0	1233.5	2511.0	1256.0	2494.0	1247.5	V	***583.4***	292.2	566.4	283.7	5
22	2582.1	1291.6	2565.1	1283.0	2610.1	**1305.5**	2593.1	1297.0	V	***484.3***	242.7	467.3	234.2	4
23	2695.2	1348.1	2678.2	1339.6	2723.2	**1362.1**	2706.1	1353.6	I	***385.3***	193.1	368.2	184.6	3
24	2792.2	1396.6	2775.2	1388.1	2820.2	1410.6	2803.2	1402.1	P	272.2	136.6	255.1	128.1	2
25									R	175.1	88.1	158.1	79.5	1

All matches to this query

Score	Mr(calc)	Delta	Site Analysis
63.1	2993.329	0.0006	Phospho S5 75.56%
58	2993.329	0.0006	Phospho S9 23.78%
42.2	2993.329	0.0006	Phospho S11 0.62%
30	2993.329	0.0006	Phospho S13 0.04%

Erk2_S1336, S1340, S1344

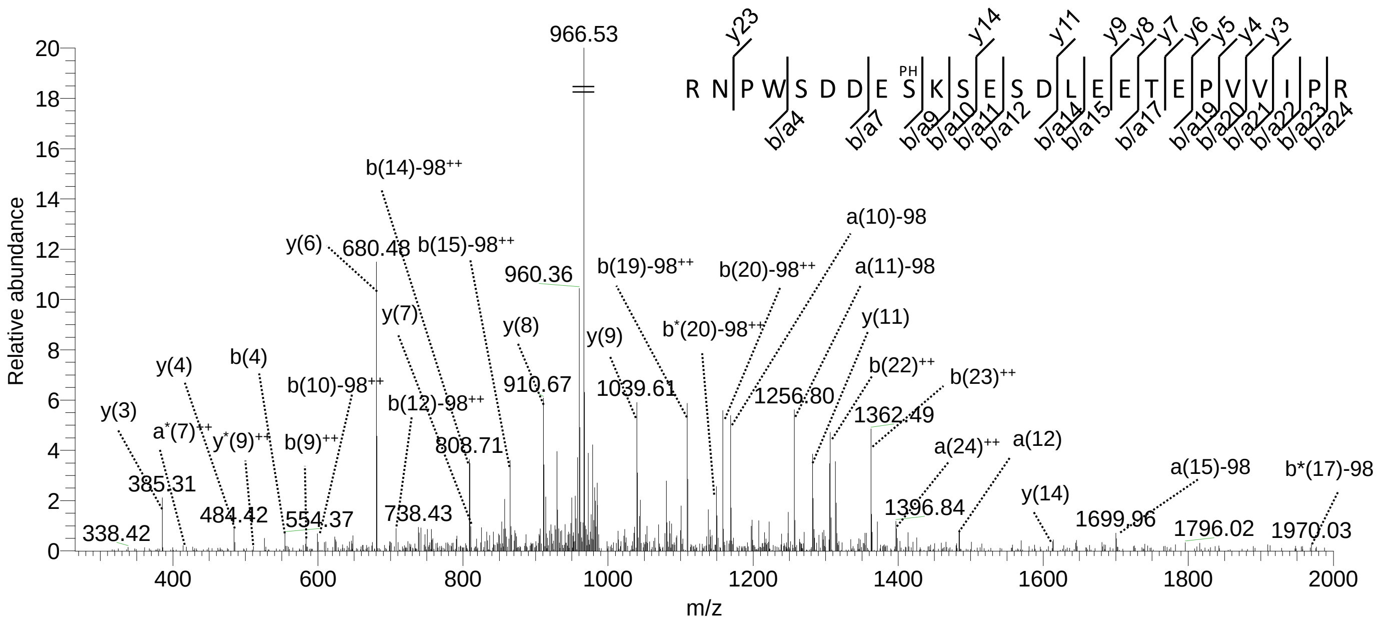

Match to Query 11698: 2993.329182 from(998.783670,3+) intensity(20568.6523) rtinseconds(2558) rawfile(SN221364_deTOP2B_Erk2_1ul.raw) scans(8339) index(6607)

Monoisotopic mass of neutral peptide Mr(calc): 2993.3291

Fixed modifications: Carbamidomethyl (C) (apply to specified residues or termini only)

Variable modifications: S9 : Phospho (ST), with neutral losses 97.9769(shown in table), 0.0000

Ions Score: 63 **Expect:** 2.4e-05 **Matches :** 44/448 fragment ions using 51 most intense peaks

#	a	a++	a*	a*++	b	b++	b*	b*++	Seq.	y	y++	y*	y*++	#
1	129.1	65.1	112.1	56.5	157.1	79.1	140.1	70.5	R					25
2	243.2	122.1	226.1	113.6	271.2	136.1	254.1	127.6	N	2740.3	1370.6	2723.2	1362.1	24
3	340.2	170.6	323.2	162.1	368.2	184.6	351.2	176.1	P	2626.2	1313.6	2609.2	1305.1	23
4	526.3	263.6	509.3	255.1	554.3	277.6	537.3	269.1	W	2529.2	1265.1	2512.1	1256.6	22
5	613.3	307.2	596.3	298.7	641.3	321.2	624.3	312.6	S	2343.1	1172.0	2326.1	1163.5	21
6	728.3	364.7	711.3	356.2	756.3	378.7	739.3	370.2	D	2256.1	1128.5	2239.0	1120.0	20
7	843.4	422.2	826.3	413.7	871.4	436.2	854.3	427.7	D	2141.0	1071.0	2124.0	1062.5	19
8	972.4	486.7	955.4	478.2	1000.4	500.7	983.4	492.2	E	2026.0	1013.5	2009.0	1005.0	18
9	**1041.4**	521.2	1024.4	512.7	1069.4	535.2	1052.4	526.7	S	1897.0	949.0	1879.9	940.5	17
10	**1169.5**	585.3	1152.5	576.8	1197.5	**599.3**	1180.5	590.8	K	1827.9	914.5	1810.9	906.0	16
11	**1256.6**	628.8	1239.5	620.3	1284.6	642.8	1267.5	634.3	S	***1699.8***	850.4	1682.8	841.9	15
12	1385.6	693.3	1368.6	684.8	1413.6	**707.3**	1396.6	698.8	E	***1612.8***	806.9	1595.8	798.4	14
13	1472.6	736.8	1455.6	728.3	1500.6	750.8	1483.6	742.3	S	***1483.8***	742.4	1466.7	733.9	13
14	1587.7	794.3	1570.6	785.8	1615.7	**808.3**	1598.6	799.8	D	***1396.7***	698.9	1379.7	690.4	12
15	**1700.8**	850.9	1683.7	842.4	1728.7	**864.9**	1711.7	856.4	L	***1281.7***	641.4	1264.7	632.8	11
16	1829.8	915.4	1812.8	906.9	1857.8	929.4	1840.8	920.9	E	***1168.6***	584.8	1151.6	576.3	10
17	1958.8	979.9	1941.8	971.4	1986.8	993.9	1969.8	985.4	E	***1039.6***	520.3	1022.6	511.8	9
18	2059.9	1030.4	2042.9	1021.9	2087.9	1044.4	2070.9	1035.9	T	***910.5***	455.8	893.5	447.3	8
19	2188.9	1095.0	2171.9	1086.5	2216.9	**1109.0**	2199.9	1100.5	E	***809.5***	405.2	792.5	396.7	7
20	2286.0	1143.5	2269.0	1135.0	2314.0	**1157.5**	2296.9	1149.0	P	***680.4***	340.7	663.4	332.2	6
21	2385.0	1193.0	2368.0	1184.5	2413.0	1207.0	2396.0	1198.5	V	***583.4***	292.2	566.4	283.7	5
22	2484.1	1242.6	2467.1	1234.0	2512.1	**1256.6**	2495.1	1248.0	V	***484.3***	242.7	467.3	234.2	4
23	2597.2	1299.1	2580.2	1290.6	2625.2	1313.1	2608.2	1304.6	I	***385.3***	193.1	368.2	184.6	3
24	2694.3	1347.6	2677.2	1339.1	2722.2	1361.6	2705.2	1353.1	P	272.2	136.6	255.1	128.1	2
25									R	175.1	88.1	158.1	79.5	1

All matches to this query

Score	Mr(calc)	Delta	Site Analysis
63	2993.3291	0.0001	Phospho S9 58.20%
61.2	2993.3291	0.0001	Phospho S5 38.90%
49.9	2993.3291	0.0001	Phospho S11 2.86%
31	2993.3291	0.0001	Phospho S13 0.04%

Erk2_S1336, S1340, S1344

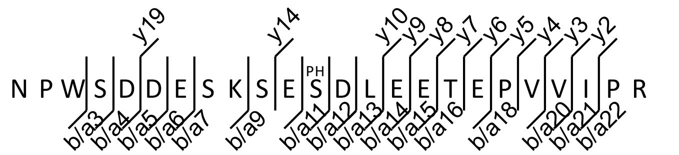

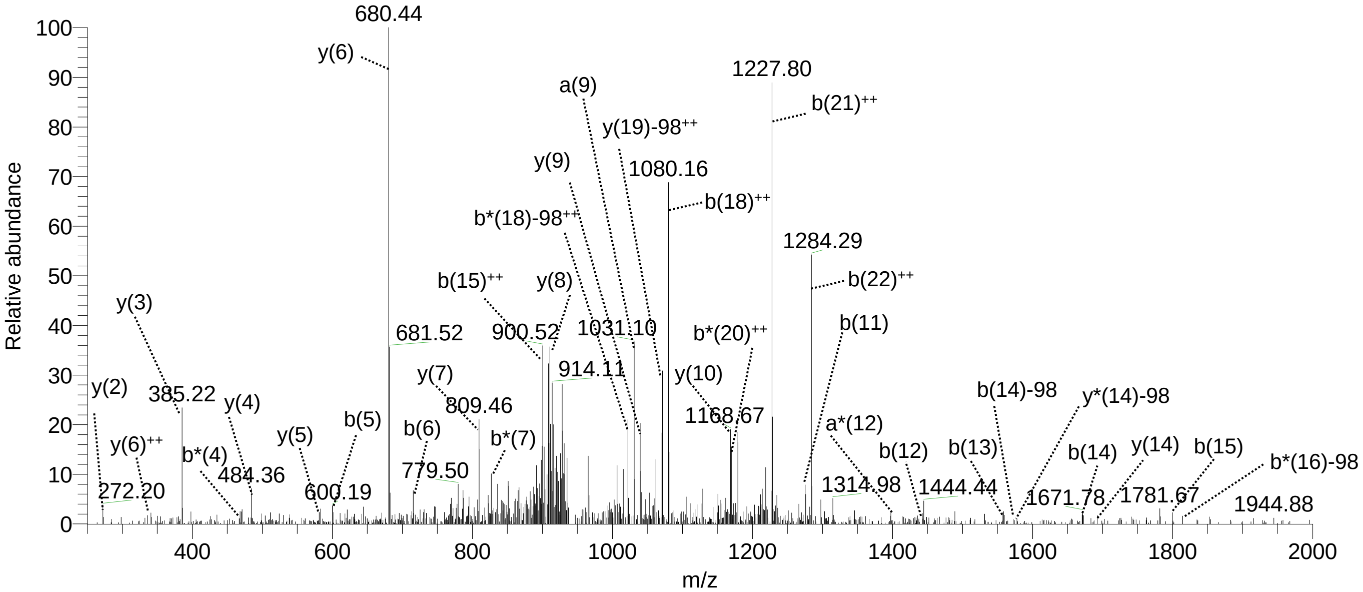

Match to Query 11292: 2837.226972 from(946.749600,3+) intensity(9996.0352) rtinseconds(2605) rawfile(SN221364_deTOP2B_Erk2_1ul.raw) scans(8539) index(6791)

Monoisotopic mass of neutral peptide Mr(calc): 2837.2280

Fixed modifications: Carbamidomethyl (C) (apply to specified residues or termini only)

Variable modifications: S12 : Phospho (ST), with neutral losses 0.0000(shown in table), 97.9769

Ions Score: 59 **Expect:** 0.00013 **Matches :** 46/416 fragment ions using 54 most intense peaks

#	a	a⁺⁺	a*	a*⁺⁺	b	b⁺⁺	b*	b*⁺⁺	Seq.	y	y⁺⁺	y*	y*⁺⁺	#
1	129.1	65.1	112.1	56.5	157.1	79.1	140.1	70.5	R					25
2	243.2	122.1	226.1	113.6	271.2	136.1	254.1	127.6	N	2838.2	1419.6	2821.2	1411.1	24
3	**340.2**	170.6	323.2	162.1	**368.2**	184.6	351.2	176.1	P	2724.2	**1362.6**	2707.2	1354.1	23
4	**526.3**	263.6	509.3	255.1	**554.3**	277.6	537.3	269.1	W	2627.1	**1314.1**	2610.1	1305.6	22
5	693.3	347.1	676.3	338.6	721.3	361.1	704.3	352.6	S	2441.1	1221.0	2424.0	1212.5	21
6	**808.3**	404.7	791.3	396.1	836.3	418.7	819.3	410.1	D	2274.1	**1137.5**	2257.0	1129.0	20
7	923.3	462.2	906.3	453.7	951.3	476.2	934.3	467.7	D	2159.0	**1080.0**	2142.0	1071.5	19
8	1052.4	526.7	1035.4	518.2	**1080.4**	540.7	1063.4	532.2	E	2044.0	**1022.5**	2027.0	1014.0	18
9	1139.4	570.2	1122.4	561.7	1167.4	**584.2**	1150.4	575.7	S	1915.0	**958.0**	1897.9	949.5	17
10	1267.5	634.3	1250.5	625.7	1295.5	**648.3**	1278.5	639.7	K	**1827.9**	914.5	1810.9	906.0	16
11	**1354.5**	677.8	1337.5	669.3	1382.5	691.8	1365.5	683.3	S	**1699.8**	850.4	1682.8	841.9	15
12	**1483.6**	742.3	1466.6	733.8	1511.6	**756.3**	1494.6	747.8	E	**1612.8**	806.9	1595.8	798.4	14
13	1570.6	785.8	1553.6	777.3	1598.6	799.8	1581.6	791.3	S	**1483.8**	742.4	1466.7	733.9	13
14	1685.6	843.3	1668.6	834.8	**1713.6**	**857.3**	1696.6	848.8	D	**1396.7**	**698.9**	1379.7	690.4	12
15	**1798.7**	899.9	1781.7	891.4	1826.7	913.9	1809.7	905.4	L	**1281.7**	**641.4**	1264.7	632.8	11
16	1927.8	964.4	1910.7	955.9	**1955.8**	**978.4**	1938.7	969.9	E	**1168.6**	**584.8**	1151.6	576.3	10
17	2056.8	1028.9	2039.8	1020.4	2084.8	**1042.9**	2067.8	1034.4	E	**1039.6**	520.3	1022.6	511.8	9
18	2157.9	1079.4	2140.8	1070.9	2185.9	1093.4	2168.8	1084.9	T	**910.5**	455.8	893.5	447.3	8
19	2286.9	1144.0	2269.9	1135.4	2314.9	**1158.0**	2297.9	1149.4	E	**809.5**	405.2	792.5	396.7	7
20	2384.0	1192.5	2366.9	1184.0	2412.0	1206.5	2394.9	1198.0	P	**680.4**	**340.7**	663.4	332.2	6
21	2483.0	1242.0	2466.0	1233.5	2511.0	1256.0	2494.0	1247.5	V	**583.4**	292.2	566.4	283.7	5
22	2582.1	1291.6	2565.1	1283.0	2610.1	**1305.5**	2593.1	1297.0	V	**484.3**	242.7	467.3	234.2	4
23	2695.2	1348.1	2678.2	1339.6	2723.2	**1362.1**	2706.1	1353.6	I	**385.3**	193.1	368.2	184.6	3
24	2792.2	1396.6	2775.2	1388.1	2820.2	1410.6	2803.2	1402.1	P	272.2	136.6	255.1	128.1	2
25									R	175.1	88.1	158.1	79.5	1

All matches to this query

Score	Mr(calc)	Delta	Site Analysis
59.4	2837.228	-0.001	Phospho S12 94.58%
42.9	2837.228	-0.001	Phospho S10 2.08%
42.9	2837.228	-0.001	Phospho S8 2.08%
40.6	2837.228	-0.001	Phospho S4 1.25%
21.5	2837.228	-0.001	Phospho T17 0.02%

Erk2m_ S1449

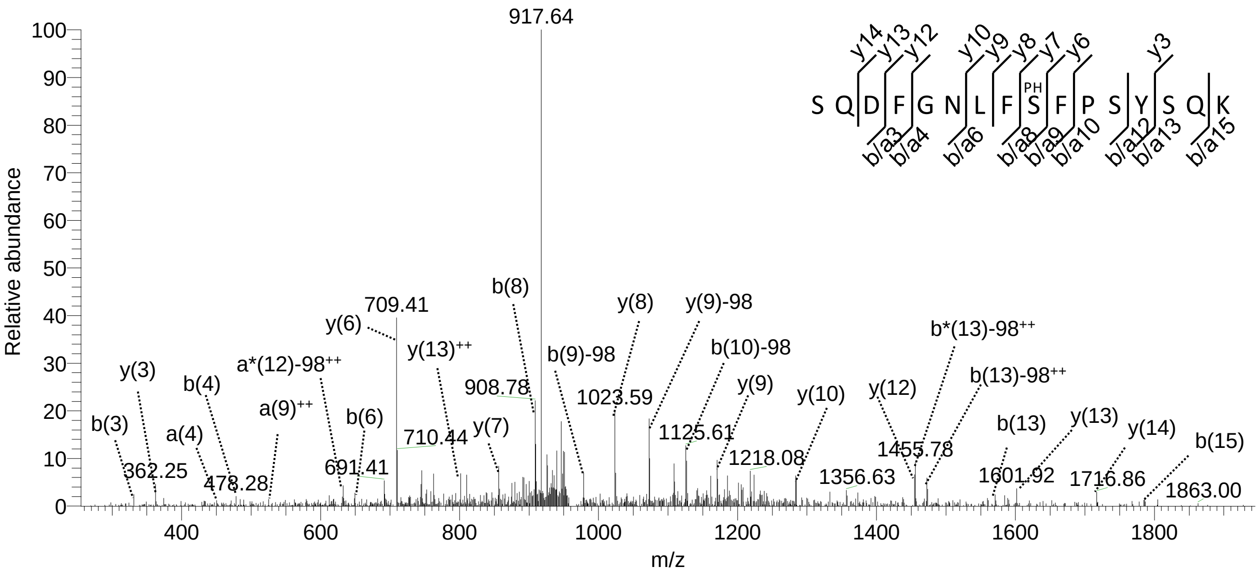

Match to Query 8559: 1930.811788 from(966.413170,2+) intensity(12163.2783) rtinseconds(3270) rawfile(SN221366_deTOP2B_Erk2m_1ul.raw) scans(11317) index(9361)

Monoisotopic mass of neutral peptide Mr(calc): 1930.8139
Fixed modifications: Carbamidomethyl (C) (apply to specified residues or termini only)
Variable modifications: S9 : Phospho (ST), with neutral losses 0.0000(shown in table), 97.9769
Ions Score: 66 **Expect:** 2.6e-06 **Matches :** 29/264 fragment ions using 34 most intense peaks

#	a	a++	a*	a*++	b	b++	b*	b*++	Seq.	y	y++	y*	y*++	#
1	60.0	30.5			88.0	44.5			S					16
2	188.1	94.6	171.1	86.0	216.1	108.6	199.1	100.0	Q	1844.8	922.9	1827.8	914.4	15
3	303.1	152.1	286.1	143.6	**331.1**	166.1	314.1	157.6	D	***1716.7***	858.9	1699.7	850.4	14
4	450.2	225.6	433.2	217.1	**478.2**	239.6	461.2	231.1	F	***1601.7***	801.4	1584.7	792.8	13
5	507.2	254.1	490.2	245.6	535.2	268.1	518.2	259.6	G	***1454.6***	727.8	1437.6	719.3	12
6	621.3	311.1	604.2	302.6	**649.3**	325.1	632.2	316.6	N	1397.6	699.3	1380.6	690.8	11
7	734.3	367.7	717.3	359.2	762.3	381.7	745.3	373.2	L	***1283.6***	642.3	1266.5	633.8	10
8	881.4	441.2	864.4	432.7	**909.4**	455.2	892.4	446.7	F	***1170.5***	585.7	1153.5	577.2	9
9	1048.4	524.7	1031.4	516.2	1076.4	538.7	1059.4	530.2	S	***1023.4***	512.2	1006.4	503.7	8
10	1195.5	598.2	1178.5	589.7	1223.5	612.2	1206.5	603.7	F	***856.4***	428.7	839.4	420.2	7
11	1292.5	646.8	1275.5	638.3	1320.5	660.8	1303.5	652.3	P	***709.4***	355.2	692.3	346.7	6
12	1379.6	690.3	1362.5	681.8	1407.6	704.3	1390.5	695.8	S	612.3	306.7	595.3	298.1	5
13	1542.6	771.8	1525.6	763.3	**1570.6**	785.8	1553.6	777.3	Y	***525.3***	263.1	508.2	254.6	4
14	1629.7	815.3	1612.6	806.8	1657.7	829.3	1640.6	820.8	S	***362.2***	181.6	345.2	173.1	3
15	1757.7	879.4	1740.7	870.9	**1785.7**	893.4	1768.7	884.8	Q	275.2	138.1	258.1	129.6	2
16									K	147.1	74.1	130.1	65.5	1

All matches to this query

Score	Mr(calc)	Delta	Site Analysis
65.8	1930.8139	-0.0021	Phospho S9 99.81%
38.3	1930.8139	-0.0021	Phospho S12 0.18%
23	1930.8139	-0.0021	Phospho Y13 0.01%
21.6	1930.8139	-0.0021	Phospho S14 0.00%
13.2	1930.8139	-0.0021	Phospho S1 0.00%

Erk2m_ S1476

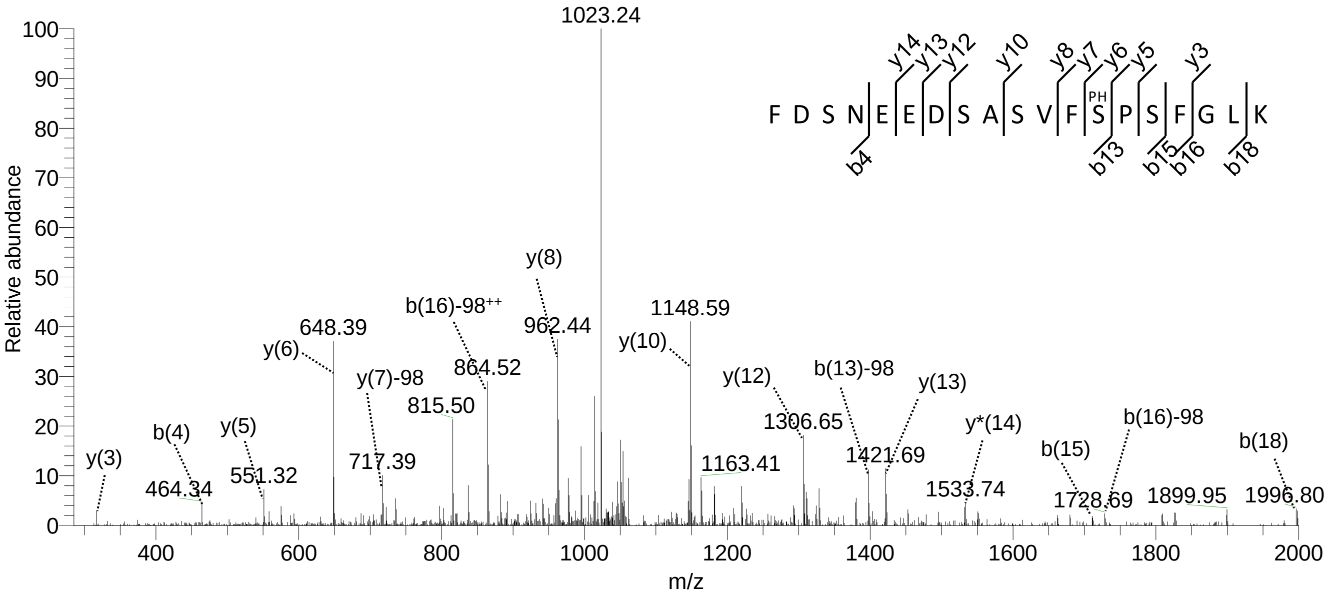

Match to Query 9274: 2141.882588 from(1071.948570,2+) intensity(21991.7324) rtinseconds(3251) rawfile(SN221366_deTOP2B_Erk2m_1ul.raw) scans(11238) index(9288)
Monoisotopic mass of neutral peptide Mr(calc): 2141.8831
Fixed modifications: Carbamidomethyl (C) (apply to specified residues or termini only)
Variable modifications: S13 : Phospho (ST), with neutral losses 0.0000(shown in table), 97.9769
Ions Score: 80 **Expect:** 6.9e-08 **Matches :** 19/300 fragment ions using 17 most intense peaks z

#	a	a++	a*	a*++	b	b++	b*	b*++	Seq.	y	y++	y*	y*++	#
1	120.1	60.5			148.1	74.5			F					19
2	235.1	118.1			263.1	132.1			D	1995.8	998.4	1978.8	989.9	18
3	322.1	161.6			350.1	175.6			S	1880.8	940.9	1863.8	932.4	17
4	436.2	218.6	419.2	210.1	464.2	232.6	447.2	224.1	N	1793.8	897.4	1776.7	888.9	16
5	565.2	283.1	548.2	274.6	593.2	297.1	576.2	288.6	E	1679.7	840.4	1662.7	831.9	15
6	694.3	347.6	677.2	339.1	722.3	361.6	705.2	353.1	E	1550.7	775.8	1533.7	767.3	14
7	809.3	405.2	792.3	396.6	837.3	419.1	820.3	410.6	D	1421.6	711.3	1404.6	702.8	13
8	896.3	448.7	879.3	440.2	924.3	462.7	907.3	454.2	S	1306.6	653.8	1289.6	645.3	12
9	967.4	484.2	950.3	475.7	995.4	498.2	978.3	489.7	A	1219.6	610.3	1202.5	601.8	11
10	1054.4	527.7	1037.4	519.2	1082.4	541.7	1065.4	533.2	S	1148.5	574.8	1131.5	566.3	10
11	1153.5	577.2	1136.4	568.7	1181.5	591.2	1164.4	582.7	V	1061.5	531.3	1044.5	522.7	9
12	1300.5	650.8	1283.5	642.3	1328.5	664.8	1311.5	656.3	F	962.4	481.7	945.4	473.2	8
13	1467.5	734.3	1450.5	725.8	1495.5	748.3	1478.5	739.8	S	815.4	408.2	798.3	399.7	7
14	1564.6	782.8	1547.6	774.3	1592.6	796.8	1575.6	788.3	P	648.4	324.7	631.3	316.2	6
15	1651.6	826.3	1634.6	817.8	1679.6	840.3	1662.6	831.8	S	551.3	276.2	534.3	267.6	5
16	1798.7	899.8	1781.7	891.3	1826.7	913.8	1809.7	905.3	F	464.3	232.6	447.3	224.1	4
17	1855.7	928.4	1838.7	919.8	1883.7	942.4	1866.7	933.8	G	317.2	159.1	300.2	150.6	3
18	1968.8	984.9	1951.8	976.4	1996.8	998.9	1979.8	990.4	L	260.2	130.6	243.2	122.1	2
19									K	147.1	74.1	130.1	65.5	1

All matches to this query

Score	Mr(calc)	Delta	Site Analysis
80.2	2141.8831	-0.0005	Phospho S13 99.06%
59.6	2141.8831	-0.0005	Phospho S10 0.86%
46.4	2141.8831	-0.0005	Phospho S15 0.04%
45.3	2141.8831	-0.0005	Phospho S8 0.03%
19.4	2141.8831	-0.0005	Phospho S3 0.00%

Erk2m_T1267

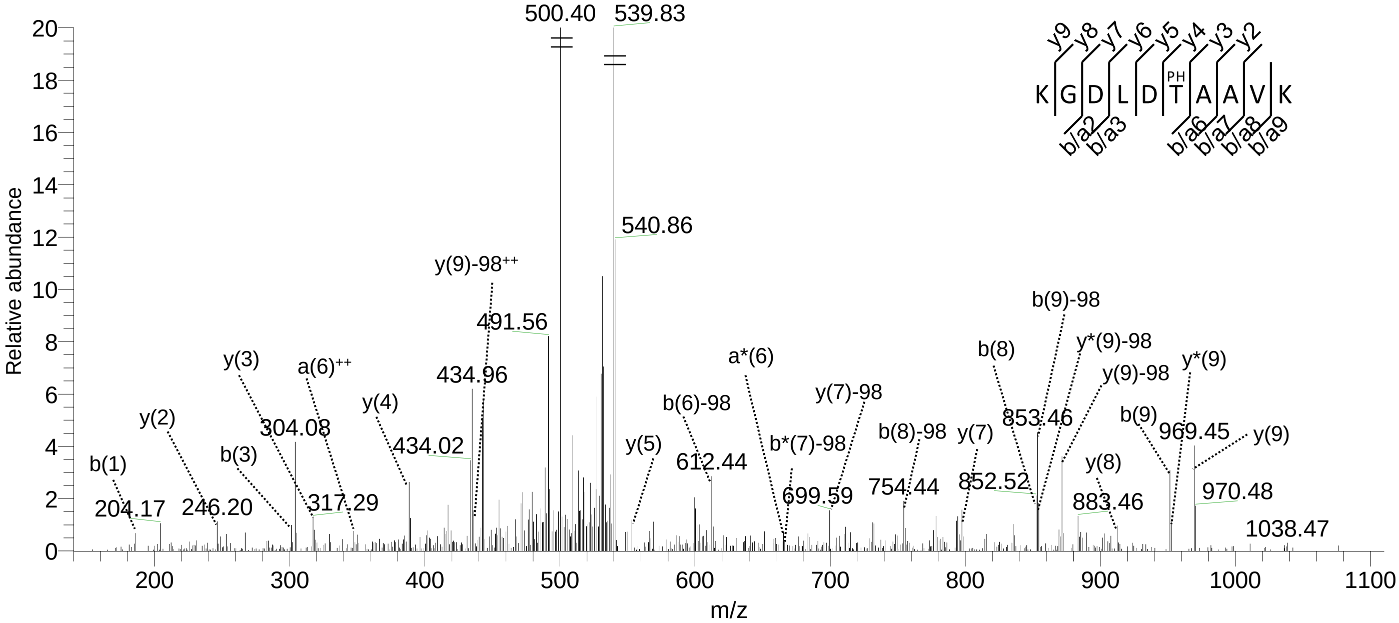

Match to Query 2189: 1096.516888 from(549.265720,2+) intensity(10601.1406) rtinseconds(1259) rawfile(SN221366_deTOP2B_Erk2m_1ul.raw) scans(2924) index(1760)

Monoisotopic mass of neutral peptide Mr(calc): 1096.5165

Fixed modifications: Carbamidomethyl (C) (apply to specified residues or termini only)

Variable modifications: T6 : Phospho (ST), with neutral losses 0.0000(shown in table), 97.9769

Ions Score: 40 **Expect:** 0.0052 **Matches :** 27/160 fragment ions using 64 most intense peaks

#	a	a++	a*	a*++	b	b++	b*	b*++	Seq.	y	y++	y*	y*++	#
1	101.1	51.1	84.1	42.5	129.1	65.1	112.1	56.5	K					10
2	158.1	79.6	141.1	71.1	**186.1**	93.6	169.1	85.1	G	***969.4***	485.2	952.4	476.7	9
3	273.2	137.1	256.1	128.6	**301.2**	151.1	284.1	142.6	D	***912.4***	456.7	895.4	448.2	8
4	386.2	193.6	369.2	185.1	414.2	207.6	397.2	199.1	L	***797.4***	399.2	780.4	390.7	7
5	501.3	251.1	484.2	242.6	529.3	265.1	512.2	256.6	D	***684.3***	342.7	667.3	334.1	6
6	682.3	341.6	665.3	333.1	710.3	355.6	693.2	347.1	T	***569.3***	285.1	552.2	276.6	5
7	753.3	377.2	736.3	368.6	781.3	391.2	764.3	382.6	A	***388.3***	194.6	371.2	186.1	4
8	824.4	412.7	807.3	404.2	**852.3**	426.7	835.3	418.2	A	***317.2***	159.1	300.2	150.6	3
9	923.4	462.2	906.4	453.7	**951.4**	476.2	934.4	467.7	V	***246.2***	123.6	229.2	115.1	2
10									K	147.1	74.1	130.1	65.5	1

Erk1m_ T1292

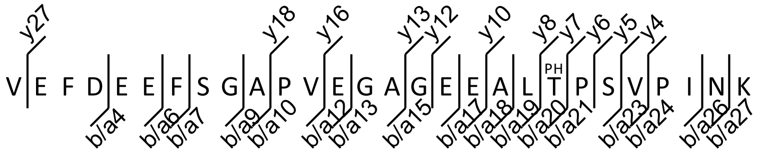

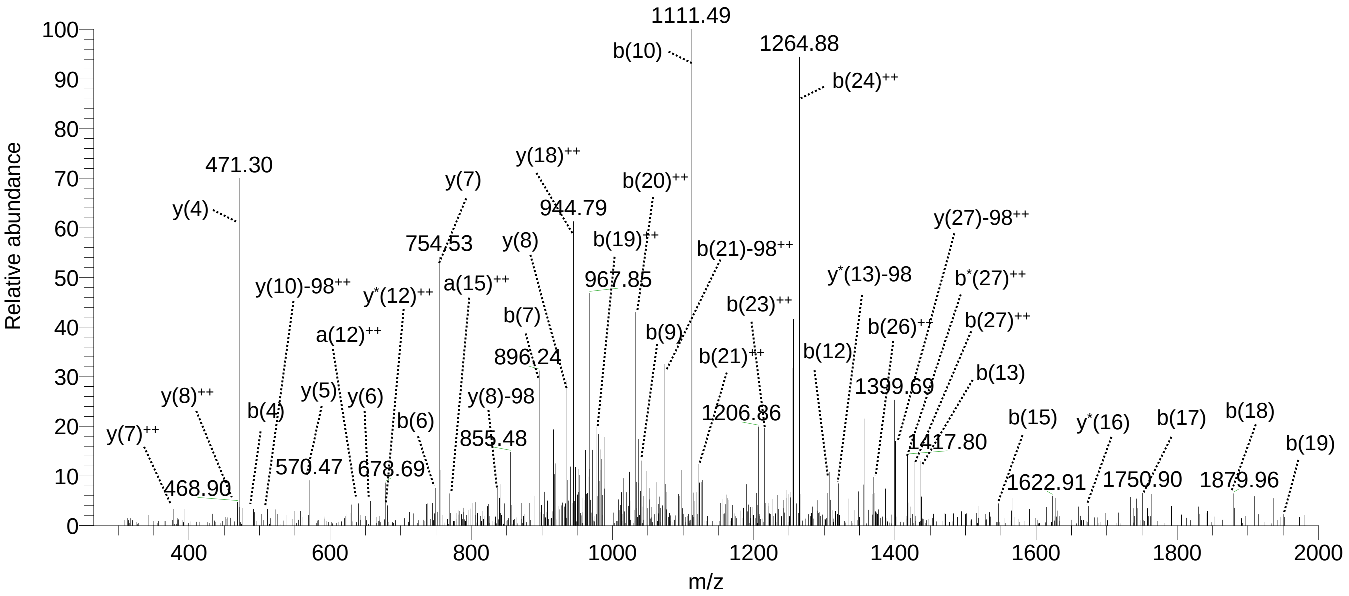

Match to Query 11653: 2997.348372 from(1000.123400,3+) intensity(71701.7266) rtinseconds(2936) rawfile(SN221365_deTOP2B_Erk1ml_1ul.raw) scans(9928) index(8092)

Monoisotopic mass of neutral peptide Mr(calc): 2997.3532
Fixed modifications: Carbamidomethyl (C) (apply to specified residues or termini only)
Variable modifications: T21 : Phospho (ST), with neutral losses 0.0000(shown in table), 97.9769
Ions Score: 40 **Expect:** 0.00073 **Matches :** 46/332 fragment ions using 85 most intense peaks

#	a	a++	a*	a*++	b	b++	b*	b*++	Seq.	y	y++	y*	y*++	#
1.0	72.1	36.5			100.1	50.5			V					28.0
2.0	201.1	101.1			229.1	115.1			E	2899.3	1450.1	2882.3	1441.6	27.0
3.0	348.2	174.6			376.2	188.6			F	2770.2	1385.6	2753.2	1377.1	26.0
4.0	463.2	232.1			491.2	246.1			D	2623.2	1312.1	2606.2	1303.6	25.0
5.0	592.3	296.6			620.3	310.6			E	2508.2	1254.6	2491.1	1246.1	24.0
6.0	721.3	361.2			749.3	375.2			E	2379.1	1190.1	2362.1	1181.5	23.0
7.0	868.4	434.7			896.4	448.7			F	2250.1	1125.5	2233.0	1117.0	22.0
8.0	955.4	478.2			983.4	492.2			S	2103.0	1052.0	2086.0	1043.5	21.0
9.0	1012.4	506.7			1040.4	520.7			G	2016.0	1008.5	1998.9	1000.0	20.0
10.0	1083.5	542.2			1111.5	556.2			A	1958.9	980.0	1941.9	971.5	19.0
11.0	1180.5	590.8			1208.5	604.8			P	1887.9	944.5	1870.9	935.9	18.0
12.0	1279.6	640.3			1307.6	654.3			V	1790.9	895.9	1773.8	887.4	17.0
13.0	1408.6	704.8			1436.6	718.8			E	1691.8	846.4	1674.8	837.9	16.0
14.0	1465.6	733.3			1493.6	747.3			G	1562.7	781.9	1545.7	773.4	15.0
15.0	1536.7	768.8			1564.7	782.8			A	1505.7	753.4	1488.7	744.9	14.0
16.0	1593.7	797.4			1621.7	811.4			G	1434.7	717.8	1417.7	709.3	13.0
17.0	1722.7	861.9			1750.7	875.9			E	1377.7	689.3	1360.6	680.8	12.0
18.0	1851.8	926.4			1879.8	940.4			E	1248.6	624.8	1231.6	616.3	11.0
19.0	1922.8	961.9			1950.8	975.9			A	1119.6	560.3	1102.6	551.8	10.0
20.0	2035.9	1018.5			2063.9	1032.5			L	1048.5	524.8	1031.5	516.3	9.0
21.0	2216.9	1109.0			2244.9	1123.0			T	935.5	468.2	918.4	459.7	8.0
22.0	2314.0	1157.5			2342.0	1171.5			P	754.4	377.7	737.4	369.2	7.0
23.0	2401.0	1201.0			2429.0	1215.0			S	657.4	329.2	640.4	320.7	6.0
24.0	2500.1	1250.5			2528.1	1264.5			V	570.4	285.7	553.3	277.2	5.0
25.0	2597.1	1299.1			2625.1	1313.1			P	471.3	236.1	454.3	227.6	4.0
26.0	2710.2	1355.6			2738.2	1369.6			I	374.2	187.6	357.2	179.1	3.0
27.0	2824.3	1412.6	2807.2	1404.1	2852.3	1426.6	2835.2	1418.1	N	261.2	131.1	244.1	122.6	2.0
28.0									K	147.1	74.1	130.1	65.5	1.0

All matches to this query

Score	Mr(calc)	Delta	Site Analysis
39.9	2997.353	-0.0049	Phospho T21 90.98%
29.8	2997.353	-0.0049	Phospho S23 8.91%
10.8	2997.353	-0.0049	Phospho S8 0.11%

Erk2m_ T1292

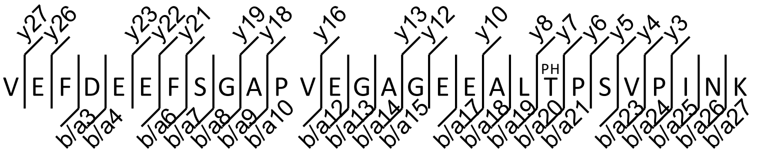

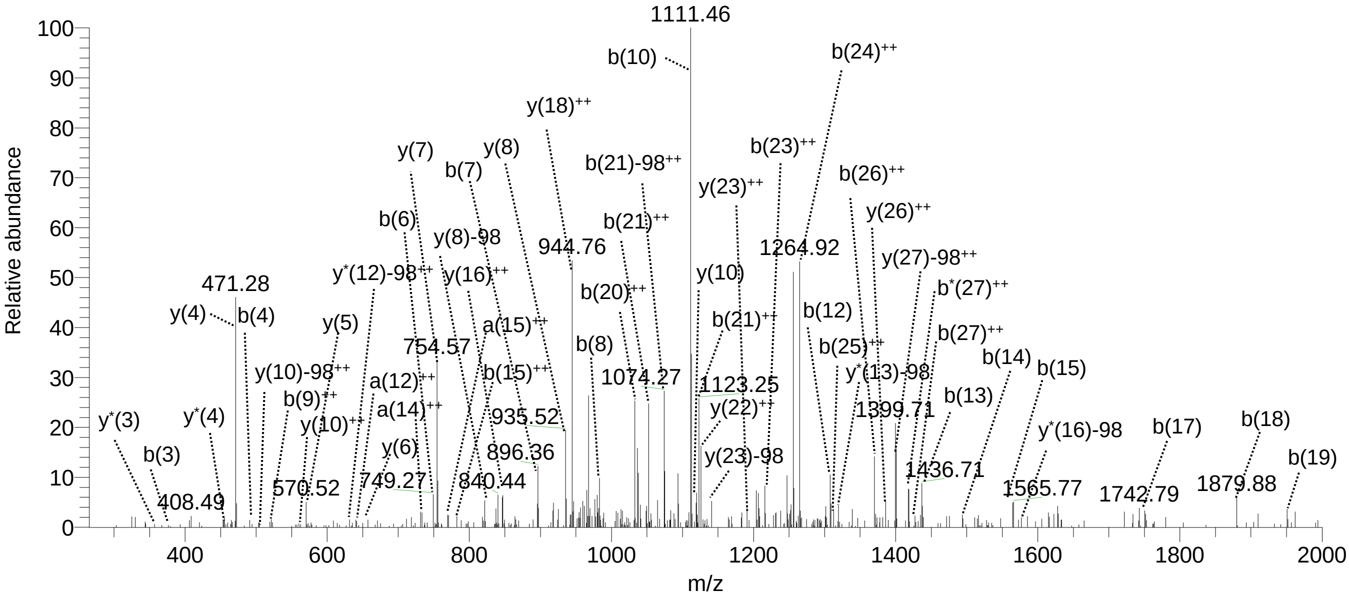

Match to Query 11387: 2997.351552 from(1000.124460,3+) intensity(543952.3750) rtinseconds(2940) rawfile(Z:¥SN22¥SN221366_deTOP2B_Erk2m_1ul.raw) scans(9948) index(8112)
Title: SpectrumID: "8131"; File: "SN221366_deTOP2B_Erk2m_1ul.raw"

Monoisotopic mass of neutral peptide Mr(calc): 2997.3532
Fixed modifications: Carbamidomethyl (C) (apply to specified residues or termini only)
Variable modifications: T21 : Phospho (ST), with neutral losses 0.0000(shown in table), 97.9769
Ions Score: 55 **Expect:** 7.8e-05 **Matches :** 65/332 fragment ions using 133 most intense peaks

#	a	a++	a*	a*++	b	b++	b*	b*++	Seq.	y	y++	y*	y*++	#
1	72.1	36.5			100.1	50.5			V					28
2	201.1	101.1			229.1	115.1			E	2899.3	1450.1	2882.3	1441.6	27
3	348.2	174.6			**376.2**	188.6			F	2770.2	**1385.6**	2753.2	1377.1	26
4	463.2	232.1			**491.2**	246.1			D	2623.2	**1312.1**	2606.2	1303.6	25
5	592.3	296.6			620.3	310.6			E	2508.2	1254.6	2491.1	1246.1	24
6	721.3	361.2			**749.3**	375.2			E	2379.1	**1190.1**	2362.1	1181.5	23
7	868.4	434.7			**896.4**	448.7			F	2250.1	**1125.5**	2233.0	1117.0	22
8	955.4	478.2			**983.4**	492.2			S	2103.0	**1052.0**	2086.0	1043.5	21
9	1012.4	506.7			1040.4	**520.7**			G	2016.0	1008.5	1998.9	1000.0	20
10	1083.5	542.2			**1111.5**	556.2			A	1958.9	**980.0**	1941.9	971.5	19
11	1180.5	590.8			1208.5	604.8			P	1887.9	**944.5**	1870.9	935.9	18
12	1279.6	640.3			**1307.6**	654.3			V	1790.9	**895.9**	1773.8	887.4	17
13	1408.6	704.8			**1436.6**	718.8			E	1691.8	**846.4**	1674.8	837.9	16
14	1465.6	733.3			**1493.6**	747.3			G	1562.7	**781.9**	1545.7	773.4	15
15	1536.7	768.8			**1564.7**	**782.8**			A	1505.7	753.4	1488.7	744.9	14
16	1593.7	797.4			1621.7	811.4			G	**1434.7**	717.8	1417.7	709.3	13
17	1722.7	861.9			**1750.7**	875.9			E	1377.7	689.3	1360.6	680.8	12
18	1851.8	926.4			**1879.8**	940.4			E	1248.6	624.8	1231.6	616.3	11
19	1922.8	961.9			**1950.8**	**975.9**			A	**1119.6**	**560.3**	1102.6	551.8	10
20	2035.9	1018.5			2063.9	**1032.5**			L	1048.5	524.8	1031.5	516.3	9
21	2216.9	1109.0			2244.9	**1123.0**			T	**935.5**	468.2	918.4	459.7	8
22	2314.0	1157.5			2342.0	1171.5			P	**754.4**	377.7	737.4	369.2	7
23	2401.0	1201.0			2429.0	**1215.0**			S	**657.4**	329.2	640.4	320.7	6
24	2500.1	1250.5			2528.1	**1264.5**			V	**570.4**	285.7	553.3	277.2	5
25	2597.1	1299.1			2625.1	**1313.1**			P	**471.3**	236.1	454.3	227.6	4
26	2710.2	1355.6			2738.2	**1369.6**			I	374.2	187.6	357.2	179.1	3
27	2824.3	1412.6	2807.2	1404.1	2852.3	**1426.6**	2835.2	1418.1	N	261.2	131.1	244.1	122.6	2
28									K	147.1	74.1	130.1	65.5	1

All matches to this query

Score	Mr(calc)	Delta	Site Analysis
54.6	2997.353	-0.0017	Phospho T21 83.30%
47.6	2997.353	-0.0017	Phospho S23 16.70%
