# Supplementary material for: ERK2-topoisomerase II regulatory axis is important for gene activation in immediate early genes
Source: Nat Commun. 2023 Dec 14;14:8341. doi: 10.1038/s41467-023-44089-y (PMC10721843; doi:10.1038/s41467-023-44089-y)
Supplement: Supplementary file 10 — Supplementary Data 7 [file 41467_2023_44089_MOESM10_ESM.pdf]

Mascot Search Results

User	: r-nakagawa
Email	:
Search title	: SN221362_deTOP2B_control_1ul_Node:2
MS data file	: File Name: Z:¥SN22¥SN221362_deTOP2B_control_1ul.raw;File Path: ;File Time: 3/1/2023 7:06:29 AM;File Size: 352609374 [Byte]
Database 1	: cRAP STRAV_proteinG_20230313 (118 sequences; 39090 residues)
Database 2	: Original_21 21_TOP2B_1-566_20210618 (1 sequences; 589 residues)
Database 3	: Uniprot_Proteome_E.coli_K12 Proteome_E.coli_K12_UP000000625_2022_01 (4448 sequences; 1358110 residues)
Timestamp	: 13 Sep 2023 at 00:22:17 GMT
Enzyme	: Trypsin
Fixed modifications	: Carbamidomethyl (C)
Variable modifications	: Acetyl (Protein N-term), Oxidation (M), Phospho (ST), Phospho (Y)
Mass values	: Monoisotopic
Protein Mass	: Unrestricted
Peptide Mass Tolerance	: ± 10 ppm
Fragment Mass Tolerance	: ± 0.8 Da
Max Missed Cleavages	: 3
Instrument type	: Default
Number of queries	: 14029

Protein hits :

2::TOP2B_1-566	TOP2B_1-566
1::sp|TRYP_PIG|	sp|TRYP_PIG|
1::sp|K2C1_HUMAN|	sp|K2C1_HUMAN|
1::sp|K1C10_HUMAN|	sp|K1C10_HUMAN|
3::P0AA25|THIO_ECOLI	Thioredoxin 1 OS=Escherichia coli (strain K12) OX=83333 GN=trxA PE=1 SV=2
1::sp|ALBU_BOVIN|	sp|ALBU_BOVIN|
1::sp|K22E_HUMAN|	sp|K22E_HUMAN|
3::P76298|FLHA_ECOLI	Flagellar biosynthesis protein FlhA OS=Escherichia coli (strain K12) OX=83333 GN=flhA PE=3 SV=1
3::P0AD96|LIVJ_ECOLI	Leu/Ile/Val-binding protein OS=Escherichia coli (strain K12) OX=83333 GN=livJ PE=1 SV=1
3::P39220|YABP_ECOLI	Protein YabP OS=Escherichia coli (strain K12) OX=83333 GN=yabP PE=4 SV=2
3::P0AFB5|NTRB_ECOLI	Sensory histidine kinase/phosphatase NtrB OS=Escherichia coli (strain K12) OX=83333 GN=glnL PE=1 SV=1
3::Q06065|ATOC_ECOLI	Regulatory protein AtoC OS=Escherichia coli (strain K12) OX=83333 GN=atoC PE=1 SV=2
3::P69741|MBHT_ECOLI	Hydrogenase-2 small chain OS=Escherichia coli (strain K12) OX=83333 GN=hybO PE=1 SV=1
3::P77439|PTFX1_ECOLI	Multiphosphoryl transfer protein 1 OS=Escherichia coli (strain K12) OX=83333 GN=fryA PE=1 SV=1
3::P06149|DLD_ECOLI	Quinone-dependent D-lactate dehydrogenase OS=Escherichia coli (strain K12) OX=83333 GN=dld PE=1 SV=3
3::P28722|YHCA_ECOLI	Uncharacterized fimbrial chaperone YhcA OS=Escherichia coli (strain K12) OX=83333 GN=yhcA PE=3 SV=2
3::Q46857|DKGA_ECOLI	2,5-diketo-D-gluconic acid reductase A OS=Escherichia coli (strain K12) OX=83333 GN=dkgA PE=1 SV=3
3::P28630|HOLA_ECOLI	DNA polymerase III subunit delta OS=Escherichia coli (strain K12) OX=83333 GN=holA PE=1 SV=1
1::sp|RS27A_HUMAN|	sp|RS27A_HUMAN|
3::Q46793|YGEN_ECOLI	Putative uncharacterized protein YgeN OS=Escherichia coli (strain K12) OX=83333 GN=ygeN PE=5 SV=3
3::P0AFS9|MEPM_ECOLI	Murein DD-endopeptidase MepM OS=Escherichia coli (strain K12) OX=83333 GN=mepM PE=1 SV=1
3::P15877|DHG_ECOLI	Quinoprotein glucose dehydrogenase OS=Escherichia coli (strain K12) OX=83333 GN=gcd PE=1 SV=3
3::P69797|PTNAB_ECOLI	PTS system mannose-specific EIIAB component OS=Escherichia coli (strain K12) OX=83333 GN=manX PE=1 SV=2
3::P75882|GFCD_ECOLI	Uncharacterized lipoprotein GfcD OS=Escherichia coli (strain K12) OX=83333 GN=gfcD PE=3 SV=1
3::P04152|UMUC_ECOLI	Protein UmuC OS=Escherichia coli (strain K12) OX=83333 GN=umuC PE=1 SV=2
3::P20966|PTFBC_ECOLI	PTS system fructose-specific EIIB'BC component OS=Escherichia coli (strain K12) OX=83333 GN=fruA PE=1 SV=1
3::P77739|KT3K_ECOLI	Probable ketoamine kinase YniA OS=Escherichia coli (strain K12) OX=83333 GN=yniA PE=3 SV=1
3::P31828|PQQL_ECOLI	Probable zinc protease PqqL OS=Escherichia coli (strain K12) OX=83333 GN=pqqL PE=1 SV=2
3::P0ACP5|GNTR_ECOLI	HTH-type transcriptional regulator GntR OS=Escherichia coli (strain K12) OX=83333 GN=gntR PE=1 SV=1
3::P0A8N3|SYK1_ECOLI	Lysine--tRNA ligase OS=Escherichia coli (strain K12) OX=83333 GN=lysS PE=1 SV=2
3::P76555|EUTQ_ECOLI	Acetate kinase EutQ OS=Escherichia coli (strain K12) OX=83333 GN=eutQ PE=1 SV=1
3::P0AEX9|MALE_ECOLI	Maltose/maltodextrin-binding periplasmic protein OS=Escherichia coli (strain K12) OX=83333 GN=malE PE=1 SV=1
3::Q46861|YGIQ_ECOLI	UPF0313 protein YgiQ OS=Escherichia coli (strain K12) OX=83333 GN=ygiQ PE=3 SV=3
3::P0ACY1|YDJA_ECOLI	Putative NAD(P)H nitroreductase YdjA OS=Escherichia coli (strain K12) OX=83333 GN=ydjA PE=1 SV=1
3::P63224|GMHA_ECOLI	Phosphoheptose isomerase OS=Escherichia coli (strain K12) OX=83333 GN=gmhA PE=1 SV=1
3::V9HVX0|YPAA_ECOLI	Uncharacterized protein YpaA OS=Escherichia coli (strain K12) OX=83333 GN=ypaA PE=4 SV=1
3::P0A6H5|HSLU_ECOLI	ATP-dependent protease ATPase subunit HslU OS=Escherichia coli (strain K12) OX=83333 GN=hslU PE=1 SV=1
3::P02943|LAMB_ECOLI	Maltoporin OS=Escherichia coli (strain K12) OX=83333 GN=lamB PE=1 SV=1
3::P52101|GLRK_ECOLI	Sensor histidine kinase GlrK OS=Escherichia coli (strain K12) OX=83333 GN=glrK PE=1 SV=3
3::P68644|FIXC_ECOLI	Protein FixC OS=Escherichia coli (strain K12) OX=83333 GN=fixC PE=3 SV=1
3::P77766|RNAAM_ECOLI	5'-3' exoribonuclease OS=Escherichia coli (strain K12) OX=83333 GN=yciV PE=1 SV=1
3::P80645|SSUD_ECOLI	Alkanesulfonate monooxygenase OS=Escherichia coli (strain K12) OX=83333 GN=ssuD PE=1 SV=3
3::P75958|LOLE_ECOLI	Lipoprotein-releasing system transmembrane protein LolE OS=Escherichia coli (strain K12) OX=83333 GN=lolE PE=1 SV=3
3::P76399|MDTC_ECOLI	Multidrug resistance protein MdtC OS=Escherichia coli (strain K12) OX=83333 GN=mdtC PE=1 SV=1
3::P0AEM0|FKBX_ECOLI	FKBP-type 16 kDa peptidyl-prolyl cis-trans isomerase OS=Escherichia coli (strain K12) OX=83333 GN=fkpB PE=1 SV=2
3::P0AC88|GM4D_ECOLI	GDP-mannose 4,6-dehydratase OS=Escherichia coli (strain K12) OX=83333 GN=gmd PE=1 SV=1
3::A0A7H2C769|A0A7H2C769_ECOLI	Putative type II secretion system C-type protein YghF OS=Escherichia coli (strain K12) OX=83333 GN=yghF PE=3 SV=1
3::P0A840|SURE_ECOLI	5'/3'-nucleotidase SurE OS=Escherichia coli (strain K12) OX=83333 GN=surE PE=1 SV=1
3::P21893|RECJ_ECOLI	Single-stranded-DNA-specific exonuclease RecJ OS=Escherichia coli (strain K12) OX=83333 GN=recJ PE=1 SV=2
3::P30852|SMF_ECOLI	Protein Smf OS=Escherichia coli (strain K12) OX=83333 GN=smf PE=3 SV=1
3::P33668|YBBC_ECOLI	Uncharacterized protein YbbC OS=Escherichia coli (strain K12) OX=83333 GN=ybbC PE=2 SV=1
3::P38035|RTCR_ECOLI	Transcriptional regulatory protein RtcR OS=Escherichia coli (strain K12) OX=83333 GN=rtcR PE=4 SV=2
3::P23871|HEMH_ECOLI	Ferrochelatase OS=Escherichia coli (strain K12) OX=83333 GN=hemH PE=1 SV=3
3::P00634|PPB_ECOLI	Alkaline phosphatase OS=Escherichia coli (strain K12) OX=83333 GN=phoA PE=1 SV=1
3::Q46864|MQSA_ECOLI	Antitoxin MqsA OS=Escherichia coli (strain K12) OX=83333 GN=mqsA PE=1 SV=1
1::sp|DHE3_BOVIN|	sp|DHE3_BOVIN|
3::P0AG24|SPOT_ECOLI	Bifunctional (p)ppGpp synthase/hydrolase SpoT OS=Escherichia coli (strain K12) OX=83333 GN=spoT PE=1 SV=1
3::P37650|BCSC_ECOLI	Cellulose synthase operon protein C OS=Escherichia coli (strain K12) OX=83333 GN=bcsC PE=1 SV=3
3::P39277|YJEH_ECOLI	L-methionine/branched-chain amino acid exporter YjeH OS=Escherichia coli (strain K12) OX=83333 GN=yjeH PE=1 SV=1
3::P77293|GTRB_ECOLI	Prophage bactoprenol glucosyl transferase homolog OS=Escherichia coli (strain K12) OX=83333 GN=yfdH PE=1 SV=1
3::P36678|GSPM_ECOLI	Putative type II secretion system protein M OS=Escherichia coli (strain K12) OX=83333 GN=gspM PE=2 SV=2
3::P16869|FHUE_ECOLI	FhuE receptor OS=Escherichia coli (strain K12) OX=83333 GN=fhuE PE=1 SV=2
3::P76161|REQ2_ECOLI	Prophage antitermination protein Q homolog QuuQ OS=Escherichia coli (strain K12) OX=83333 GN=quuQ PE=3 SV=2
3::P64570|YQGC_ECOLI	Protein YqgC OS=Escherichia coli (strain K12) OX=83333 GN=yqgC PE=1 SV=1
3::P77732|RHMR_ECOLI	Uncharacterized HTH-type transcriptional regulator RhmR OS=Escherichia coli (strain K12) OX=83333 GN=rhmR PE=1 SV=1
3::P25772|LIGB_ECOLI	DNA ligase B OS=Escherichia coli (strain K12) OX=83333 GN=ligB PE=1 SV=2
3::P75977|YMFM_ECOLI	Uncharacterized protein YmfM OS=Escherichia coli (strain K12) OX=83333 GN=ymfM PE=4 SV=1
3::P77658|YNAA_ECOLI	Protein YnaA OS=Escherichia coli (strain K12) OX=83333 GN=ynaA PE=4 SV=1
3::P0A7R9|RS11_ECOLI	30S ribosomal protein S11 OS=Escherichia coli (strain K12) OX=83333 GN=rpsK PE=1 SV=2
3::P00957|SYA_ECOLI	Alanine--tRNA ligase OS=Escherichia coli (strain K12) OX=83333 GN=alaS PE=1 SV=2
3::P07813|SYL_ECOLI	Leucine--tRNA ligase OS=Escherichia coli (strain K12) OX=83333 GN=leuS PE=1 SV=2
3::P0ACZ8|CUSR_ECOLI	Transcriptional regulatory protein CusR OS=Escherichia coli (strain K12) OX=83333 GN=cusR PE=1 SV=1
3::P24215|UXUA_ECOLI	Mannonate dehydratase OS=Escherichia coli (strain K12) OX=83333 GN=uxuA PE=1 SV=2
3::P38134|ETK_ECOLI	Tyrosine-protein kinase etk OS=Escherichia coli (strain K12) OX=83333 GN=etk PE=1 SV=2
3::P0ADT8|YGIM_ECOLI	Uncharacterized protein YgiM OS=Escherichia coli (strain K12) OX=83333 GN=ygiM PE=3 SV=1
3::P0AFA2|NARX_ECOLI	Nitrate/nitrite sensor protein NarX OS=Escherichia coli (strain K12) OX=83333 GN=narX PE=1 SV=1
3::P20099|BISC_ECOLI	Biotin sulfoxide reductase OS=Escherichia coli (strain K12) OX=83333 GN=bisC PE=1 SV=3
3::P33362|YEHZ_ECOLI	Glycine betaine-binding protein YehZ OS=Escherichia coli (strain K12) OX=83333 GN=yehZ PE=1 SV=1
3::P39358|YJHG_ECOLI	D-xylonate dehydratase YjhG OS=Escherichia coli (strain K12) OX=83333 GN=yjhG PE=1 SV=2

	Target	Decoy	False discovery rate
Peptide matches above identity threshold	462	55	11.90 %
Peptide matches above homology or identity threshold	463	59	12.74 %

Select Summary Report

Format As [Select Summary (protein hits)] Help Help

Significance threshold p< 0.05 Max. number of hits AUTO Show Percolator scores ☐

Standard scoring ○ MudPIT scoring ◉ Display non-significant matches ☐ Show sub-sets 0

Show pop-ups ◉ Suppress pop-ups ○ Require bold red ☐

Preferred taxonomy [All entries]

Re-Search ◉ All queries ○ Unassigned ○ Below homology threshold ○ Below identity threshold

1. 2::TOP2B_1-566 Mass: 66629 Score: 8456 Matches: 341(341) Sequences: 49(49) emPAI: 40.10

TOP2B_1-566

Query	Observed	Mr(expt)	Mr(calc)	ppm	Miss	Score	Expect	Rank	Unique	Peptide
303	388.7291	775.4437	775.4439	-0.27	1	17	0.02	1	U	K.TSEAKIK.H
410	401.7254	801.4362	801.4344	2.25	0	36	0.00031	1	U	K.AQTQLNK.K 408
480	410.7507	819.4868	819.4854	1.71	0	32	0.00057	1	U	K.IVGLQYK.K 476 477 479
521	414.7815	827.5485	827.5480	0.57	1	26	0.0024	1	U	K.LIEVVKK.K
640	426.2421	850.4697	850.4701	-0.46	0	35	0.00033	1	U	R.YGVFPLR.G 639 641 642 643 644 645 646 647 648
779	439.7585	877.5025	877.5022	0.38	0	28	0.002	1	U	K.GIPVVEHK.V 781
988	459.7574	917.5003	917.5004	-0.11	0	42	0.00012	1	U	K.DIVALMTR.R
1061	465.7719	929.5293	929.5294	-0.12	1	32	0.00081	1	U	K.AQTQLNKK.C 1060 1062
1093	467.7551	933.4957	933.4953	0.41	0	(32)	0.00084	1	U	K.DIVALMTR.R 1088 1089 1090 1091 1092
1154	473.2665	944.5185	944.5179	0.69	0	51	1.1e-005	1	U	K.LDETGVALK.V

1200	478.2307	954.4469	954.4481	-1.22	0	19	0.018	1	U	K.FTVETACK.E
1338	488.2227	974.4308	974.4305	0.28	0	65	3.8e-007	1	U	K.LDDANDAGGK.H 1337 1339 1340 1341 1342
1351	488.7425	975.4704	975.4695	0.96	0	21	0.0099	1	U	K.ENMTLQPK.S 1346 1348 1352
1394	491.7550	981.4955	981.4953	0.12	0	42	0.00013	1	U	K.LCNIFSTK.F
1421	493.7637	985.5128	985.5120	0.76	0	43	6.3e-005	1	U	R.SYVDLYVK.D 1419 1420
1576	505.2439	1008.4732	1008.4732	-0.05	1	(19)	0.016	1	U	R.DKNMTCIK.V
1596	506.7296	1011.4447	1011.4444	0.36	0	59	1.8e-006	1	U	R.AYDLAGSCR.G
1697	513.2414	1024.4682	1024.4681	0.03	1	28	0.003	1	U	R.DKNMTCIK.V
1716	514.2327	1026.4508	1026.4505	0.27	0	43	4.7e-005	1	U	K.SYDDAESLK.T 1713 1714 1715 1717
1779	518.2513	1034.4881	1034.4880	0.09	1	42	9.7e-005	1	U	K.KEESETANK.N
2136	539.3061	1076.5977	1076.5978	-0.14	1	26	0.0035	1	U	K.FKAQTQLNK.K
2137	359.8732	1076.5978	1076.5978	-0.05	1	(18)	0.022	1	U	K.FKAQTQLNK.K
2173	540.7937	1079.5728	1079.5723	0.41	0	51	1.3e-005	1	U	K.VIHELANER.W 2158 2163 2164 2168 2170 2175 2177
2174	360.8649	1079.5728	1079.5723	0.42	0	(44)	7.6e-005	1	U	K.VIHELANER.W 2159 2161 2162 2165 2166 2169 2171 2172 2176
2546	374.8732	1121.5978	1121.5982	-0.31	1	(21)	0.012	1	U	R.DRYGVFPLR.G
2547	561.8067	1121.5989	1121.5982	0.67	1	32	0.00097	1	U	R.DRYGVFPLR.G
2603	564.8401	1127.6657	1127.6663	-0.49	0	69	1.2e-007	1	U	K.SLAVSGLGVIGR.D 2601 2602 2605 2606 2607 2608 2609 2610 2611 2612 2614 2615 2616 2617 2618 2619 2
2613	376.8961	1127.6663	1127.6663	0.05	0	(46)	2.5e-005	1	U	K.SLAVSGLGVIGR.D
2847	576.8182	1151.6218	1151.6227	-0.73	0	15	0.049	1	U	R.EVTFVPGLYK.I
2883	578.2803	1154.5459	1154.5455	0.39	1	33	0.0011	1	U	K.KSYDDAESLK.T 2880 2884
2916	387.2321	1158.6746	1158.6761	-1.30	0	(43)	6.3e-005	1	U	K.AGVSVKPFQVK.N 2923 2926 2927
2928	580.3458	1158.6770	1158.6761	0.82	0	51	1.1e-005	1	U	K.AGVSVKPFQVK.N 2917 2922 2924 2925 2932
2994	584.7798	1167.5451	1167.5455	-0.33	1	41	0.00017	1	U	R.RAYDLAGSCR.G
3143	594.8270	1187.6394	1187.6398	-0.29	1	50	2.2e-005	1	U	K.DKLDETGVALK.V 3144 3145 3147 3148
3149	396.8873	1187.6402	1187.6398	0.37	1	(25)	0.006	1	U	K.DKLDETGVALK.V 3146
3212	600.2545	1198.4944	1198.4933	0.89	0	(32)	0.00098	1	U	K.QTWMNNMMK.T
3329	608.2515	1214.4885	1214.4882	0.22	0	(23)	0.0056	1	U	K.QTWMNNMMK.T 3328
3330	608.2516	1214.4886	1214.4882	0.32	0	33	0.0005	1	U	K.QTWMNNMMK.T
3440	615.3243	1228.6340	1228.6339	0.09	1	20	0.013	1	U	R.SYVDLYVKDK.L
3624	625.8080	1249.6014	1249.6013	0.11	0	57	4.1e-006	1	U	R.WDVCLTLSEK.G
3857	637.8604	1273.7063	1273.7064	-0.10	1	71	1.2e-007	1	U	K.LDKDIVALMTR.R 3844 3850 3853 3854 3855 3858 3860 3861 3863 3866 3867 3868
3862	425.5762	1273.7068	1273.7064	0.35	1	(39)	0.00019	1	U	K.LDKDIVALMTR.R 3847 3864
3985	430.9075	1289.7006	1289.7013	-0.52	1	(45)	7.1e-005	1	U	K.LDKDIVALMTR.R 3986 3987 3988 3991 3992 3994 3995
3990	645.8578	1289.7010	1289.7013	-0.22	1	(65)	5.7e-007	1	U	K.LDKDIVALMTR.R 3980 3983 3984 3989 3993 3997
4491	679.3590	1356.7034	1356.7038	-0.26	0	80	2.6e-008	1	U	R.HVDYVVDQVVGK.L 4486 4487 4489 4490 4492 4493 4494 4495 4496 4499 4501 4503 4504
4502	453.2421	1356.7046	1356.7038	0.59	0	(34)	0.0012	1	U	R.HVDYVVDQVVGK.L 4488 4497 4498 4500
4773	699.3490	1396.6834	1396.6834	-0.02	1	55	5.7e-006	1	U	K.SYDDAESLKTLR.Y 4774
5259	731.3818	1460.7491	1460.7511	-1.37	0	109	3e-011	1	U	K.IFDEILVNAADNK.Q 5268 5270 5271 5274 5278 5279 5283 5285 5287 5288 5293 5295 5296
5281	487.9244	1460.7513	1460.7511	0.16	0	(49)	3.7e-005	1	U	K.IFDEILVNAADNK.Q 5258 5260 5261 5262 5263 5264 5265 5266 5267 5269 5272 5273 5275 5276 5277 5280
6010	765.4036	1528.7927	1528.7919	0.52	0	90	2.5e-009	1	U	K.QIMENAEINNIIK.I
6011	510.6049	1528.7929	1528.7919	0.66	0	(47)	5.3e-005	1	U	K.QIMENAEINNIIK.I
6213	773.4009	1544.7873	1544.7868	0.34	0	(87)	4.5e-009	1	U	K.QIMENAEINNIIK.I 6209 6210 6211 6212
6436	522.9126	1565.7161	1565.7169	-0.51	2	(19)	0.041	1	U	K.KEESETANKNDSSK.K
6439	783.8661	1565.7176	1565.7169	0.47	2	32	0.0022	1	U	K.KEESETANKNDSSK.K
7035	547.6262	1639.8568	1639.8570	-0.13	0	(48)	3.7e-005	1	U	K.GFQQISFVNSIATTK.G 7036 7041 7045 7050 7054
7049	820.9363	1639.8580	1639.8570	0.60	0	78	3.2e-008	1	U	K.GFQQISFVNSIATTK.G 7018 7019 7020 7021 7022 7023 7024 7026 7027 7028 7029 7030 7031 7032 7033 70
7502	847.9132	1693.8118	1693.8118	-0.00	3	47	6.3e-005	1	U	K.KEESETANKNDSSKK.L 7504
7503	565.6114	1693.8123	1693.8118	0.28	3	(25)	0.0099	1	U	K.KEESETANKNDSSKK.L
7802	577.9397	1730.7971	1730.7968	0.21	0	(31)	0.0031	1	U	K.IMIMTDQDQDGSHIK.A 7810
7807	866.4066	1730.7987	1730.7968	1.13	0	(80)	4.2e-008	1	U	K.IMIMTDQDQDGSHIK.A 7799 7808
7934	582.6445	1744.9116	1744.9108	0.48	1	30	0.0024	1	U	K.IFDEILVNAADNKQR.D
7952	583.2712	1746.7918	1746.7917	0.06	0	(41)	0.0002	1	U	K.IMIMTDQDQDGSHIK.A 7959
7953	874.4032	1746.7919	1746.7917	0.11	0	82	1.7e-008	1	U	K.IMIMTDQDQDGSHIK.A 7946
7957	874.4033	1746.7920	1746.7917	0.22	0	(79)	3e-008	1	U	K.IMIMTDQDQDGSHIK.A 7949 7951
7961	583.2719	1746.7939	1746.7917	1.26	0	(47)	4.8e-005	1	U	K.IMIMTDQDQDGSHIK.A 7945 7950 7954 7955 7956 7958 7960 7962
8095	882.4007	1762.7868	1762.7866	0.15	0	(71)	2.9e-007	1	U	K.IMIMTDQDQDGSHIK.A 8089 8092 8093 8096 8100 8102 8104
8097	588.6029	1762.7870	1762.7866	0.23	0	(58)	6.2e-006	1	U	K.IMIMTDQDQDGSHIK.A 8086 8087 8088 8090 8091 8094 8099 8101 8103 8105 8106
8179	887.4379	1772.8612	1772.8615	-0.16	0	97	8.6e-010	1	U	K.HSLECTLILTEGDSAK.S
8180	591.9611	1772.8615	1772.8615	0.01	0	(31)	0.0034	1	U	K.HSLECTLILTEGDSAK.S
9004	943.4863	1884.9580	1884.9581	-0.04	0	69	2.8e-007	1	U	K.VSIDPESNIISIWNNGK.G 9003 9006
9005	629.3268	1884.9585	1884.9581	0.21	0	(59)	2.5e-006	1	U	K.VSIDPESNIISIWNNGK.G
10280	724.9897	2171.9472	2171.9470	0.09	0	(26)	0.01	1	U	K.HFDGEDYTCITFQPDLSK.F
10383	735.0150	2202.0231	2202.0236	-0.27	0	24	0.016	1	U	K.SGGCGAGAGVGGGNGALTWVNNAAK.K
10560	563.3082	2249.2039	2249.2015	1.04	2	24	0.0082	1	U	K.DKLDETGVALKVIHELANER.W
10569	1126.9724	2251.9302	2251.9133	7.49	0	46	8.9e-005	1	U	K.HFDGEDYTCITFQPDLSK.F
10639	761.6696	2281.9871	2281.9900	-1.26	0	(22)	0.025	1	U	K.SGGCGAGAGVGGGNGALTWVNNAAK.K
11202	829.7476	2486.2209	2486.2217	-0.33	0	(53)	2.4e-005	1	U	K.VYVPALIFGQLLTSSNYDDDEK.K 11201 11203 11205 11207
11204	1244.1186	2486.2227	2486.2217	0.40	0	95	1.5e-009	1	U	K.VYVPALIFGQLLTSSNYDDDEK.K 11199 11200 11206 11209
11429	859.4249	2575.2528	2575.2529	-0.07	0	63	2.6e-006	1	U	K.NHIWVFINCLIENPTFDSQTK.E 11430
11520	654.5867	2614.3177	2614.3166	0.42	1	(39)	0.00062	1	U	K.VYVPALIFGQLLTSSNYDDDEKK.V 11512 11526 11528
11522	1308.1667	2614.3188	2614.3166	0.85	1	(61)	4.1e-006	1	U	K.VYVPALIFGQLLTSSNYDDDEKK.V 11515
11524	872.4470	2614.3190	2614.3166	0.91	1	61	3.6e-006	1	U	K.VYVPALIFGQLLTSSNYDDDEKK.V 11509 11513 11514 11516 11521
12009	948.4822	2842.4247	2842.4276	-1.04	1	54	2.1e-005	1	U	K.VEKVYVPALIFGQLLTSSNYDDDEK.K

---

2. 1::sp|TRYP_PIG| Mass: 25078 Score: 266 Matches: 13(13) Sequences: 2(2) emPAI: 0.40

sp|TRYP_PIG|

Query	Observed	Mr(expt)	Mr(calc)	ppm	Miss	Score	Expect	Rank	Unique	Peptide
596	421.7584	841.5023	841.5022	0.14	0	54	4e-006	1	U	R.VATVSLPR.S 590 591 592 593 594 595 597 598 599 600 601
1849	523.2858	1044.5570	1044.5564	0.65	0	37	0.00036	1	U	K.LSSPATLNSR.V

---

3. 1::sp|K2C1_HUMAN| Mass: 66018 Score: 200 Matches: 8(8) Sequences: 8(8) emPAI: 0.68

sp|K2C1_HUMAN|

Query	Observed	Mr(expt)	Mr(calc)	ppm	Miss	Score	Expect	Rank	Unique	Peptide
1324	487.2693	972.5240	972.5240	0.06	0	33	0.00068	1		K.IEISELNR.V
1766	517.2619	1032.5092	1032.5087	0.43	0	28	0.0022	1	U	R.TLLEGEESR.M

2278	546.7549	1091.4953	1091.4956	-0.26	0	28	0.0015	1	U	R.GSGGGSSGGSIGGR.G
2578	563.2767	1124.5389	1124.5349	3.50	0	40	0.00021	1	U	K.AEAESLYQSK.Y
3080	590.3038	1178.5931	1178.5931	0.00	0	28	0.0038	1	U	K.YEELQITAGR.H
4096	651.8613	1301.7079	1301.7078	0.09	0	49	2.6e-005	1	U	R.SLDLDSIIAEVK.A
4138	436.8899	1307.6479	1307.6469	0.75	2	45	9e-005	1	U	R.NKYEDEINKR.T
4688	692.3493	1382.6841	1382.6830	0.76	0	54	7.3e-006	1	U	K.SLNNQFASFIDK.V

---

4. 1::sp|K1C10_HUMAN| Mass: 59711 Score: 161 Matches: 5(5) Sequences: 5(5) emPAI: 0.43

sp|K1C10_HUMAN|

Query	Observed	Mr(expt)	Mr(calc)	ppm	Miss	Score	Expect	Rank	Unique	Peptide
433	404.2033	806.3920	806.3923	-0.29	0	27	0.0027	1	U	R.LAADDFR.L
441	405.2239	808.4332	808.4330	0.18	0	14	0.037	1	U	R.LASYLDK.V
3718	631.8024	1261.5902	1261.5899	0.24	0	40	0.00022	1	U	R.SLLEGEGSSGGGGR.G
4563	683.3234	1364.6322	1364.6320	0.12	0	60	1.8e-006	1	U	R.SQYEQLAEQNR.K
4673	691.3284	1380.6422	1380.6408	1.01	0	80	2.8e-008	1	U	R.ALEESNYELEGK.I

---

5. 3::P0AA25|THIO_ECOLI Mass: 11913 Score: 127 Matches: 2(2) Sequences: 2(2) emPAI: 1.01

Thioredoxin 1 OS=Escherichia coli (strain K12) OX=83333 GN=trxA PE=1 SV=2

Query	Observed	Mr(expt)	Mr(calc)	ppm	Miss	Score	Expect	Rank	Unique	Peptide
3771	634.3357	1266.6569	1266.6568	0.10	0	67	4e-007	1	U	K.LNIDQNPGTAPK.Y
8495	903.4535	1804.8924	1804.8917	0.42	0	77	6.6e-008	1	U	K.MIAPILDEIADEYQGK.L

---

6. 1::sp|ALBU_BOVIN| Mass: 71244 Score: 125 Matches: 6(6) Sequences: 6(6) emPAI: 0.43

sp|ALBU_BOVIN|

Query	Observed	Mr(expt)	Mr(calc)	ppm	Miss	Score	Expect	Rank	Unique	Peptide
363	395.2400	788.4654	788.4644	1.37	0	18	0.017	1	U	K.LVTDLTK.V
468	409.7170	817.4195	817.4181	1.72	0	23	0.0053	1	U	K.ATEEQLK.T
1001	461.7478	921.4810	921.4807	0.31	0	27	0.0027	1	U	K.AEFVEVTK.L
4116	653.3620	1304.7094	1304.7088	0.47	0	47	3.2e-005	1	U	K.HLVDEPQNLIK.Q
6450	784.3763	1566.7381	1566.7354	1.70	0	45	9.6e-005	1	U	K.DAFLGSFLYEYSR.R
7012	547.3176	1638.9310	1638.9305	0.30	1	36	0.00024	1	U	R.KVPQVSTPTLVEVSR.S

---

7. 1::sp|K22E_HUMAN| Mass: 66110 Score: 102 Matches: 4(4) Sequences: 4(4) emPAI: 0.29

sp|K22E_HUMAN|

Query	Observed	Mr(expt)	Mr(calc)	ppm	Miss	Score	Expect	Rank	Unique	Peptide
1324	487.2693	972.5240	972.5240	0.06	0	33	0.00068	1		K.IEISELNR.V
2398	554.2752	1106.5358	1106.5356	0.19	0	27	0.0023	1	U	K.AQYEEIAQR.S
3178	398.8754	1193.6045	1193.6040	0.38	2	25	0.0046	1	U	K.KYEDEINKR.T
3653	627.8076	1253.6007	1253.6001	0.49	0	59	3e-006	1	U	R.GFSSGSAVVSGGSR.R

---

8. 3::P76298|FLHA_ECOLI Mass: 74795 Score: 95 Matches: 12(12) Sequences: 1(1) emPAI: 0.06

Flagellar biosynthesis protein FlhA OS=Escherichia coli (strain K12) OX=83333 GN=flhA PE=3 SV=1

Query	Observed	Mr(expt)	Mr(calc)	ppm	Miss	Score	Expect	Rank	Unique	Peptide
3743	632.8199	1263.6252	1263.6159	7.37	1	26	0.0045	1	U	R.HIRMTATIGGK.- 3726 3735 3742 3746 3753 3756
3755	632.8202	1263.6259	1263.6159	7.94	1	(26)	0.0041	1	U	R.HIRMTATIGGK.- 3734 3744 3748 3754

---

9. 3::P0AD96|LIVJ_ECOLI Mass: 39223 Score: 31 Matches: 1(1) Sequences: 1(1) emPAI: 0.11

Leu/Ile/Val-binding protein OS=Escherichia coli (strain K12) OX=83333 GN=livJ PE=1 SV=1

Query	Observed	Mr(expt)	Mr(calc)	ppm	Miss	Score	Expect	Rank	Unique	Peptide
397	400.7352	799.4559	799.4552	0.85	0	31	0.00082	1	U	K.QAVAVANK.V

Proteins matching the same set of peptides:

3::P04816|LIVK_ECOLI Mass: 39468 Score: 31 Matches: 1(1) Sequences: 1(1)

Leucine-specific-binding protein OS=Escherichia coli (strain K12) OX=83333 GN=livK PE=1 SV=2

---

10. 3::P39220|YABP_ECOLI Mass: 24946 Score: 26 Matches: 2(2) Sequences: 2(2) emPAI: 0.40

Protein YabP OS=Escherichia coli (strain K12) OX=83333 GN=yabP PE=4 SV=2

Query	Observed	Mr(expt)	Mr(calc)	ppm	Miss	Score	Expect	Rank	Unique	Peptide
815	443.2374	884.4602	884.4603	-0.16	0	17	0.021	1	U	K.NPDELVAK.G
4109	652.8180	1303.6214	1303.6213	0.05	0	23	0.009	1	U	K.FINSLELFNK.L

---

11. 3::P0AFB5|NTRB_ECOLI Mass: 38532 Score: 26 Matches: 1(1) Sequences: 1(1) emPAI: 0.12

Sensory histidine kinase/phosphatase NtrB OS=Escherichia coli (strain K12) OX=83333 GN=glnL PE=1 SV=1

Query	Observed	Mr(expt)	Mr(calc)	ppm	Miss	Score	Expect	Rank	Unique	Peptide
89	363.7163	725.4180	725.4184	-0.50	0	26	0.0027	1	U	K.NPLGGLR.G

---

12. 3::Q06065|ATOC_ECOLI Mass: 52428 Score: 25 Matches: 2(2) Sequences: 1(1) emPAI: 0.08

Regulatory protein AtoC OS=Escherichia coli (strain K12) OX=83333 GN=atoC PE=1 SV=2

Query	Observed	Mr(expt)	Mr(calc)	ppm	Miss	Score	Expect	Rank	Unique	Peptide
3733	422.2154	1263.6245	1263.6203	3.32	1	20	0.017	1	U	R.MPEMDGIKALK.E 3736

---

13. 3::P69741|MBHT_ECOLI Mass: 40368 Score: 24 Matches: 1(1) Sequences: 1(1) emPAI: 0.11

Hydrogenase-2 small chain OS=Escherichia coli (strain K12) OX=83333 GN=hybO PE=1 SV=1

Query	Observed	Mr(expt)	Mr(calc)	ppm	Miss	Score	Expect	Rank	Unique	Peptide
9983	697.6294	2089.8665	2089.8689	-1.13	1	24	0.014	1	U	R.DFMKLCAALAATMGLSSK.A

---

14. 3::P77439|PTFX1_ECOLI Mass: 92757 Score: 24 Matches: 1(1) Sequences: 1(1) emPAI: 0.05

Multiphosphoryl transfer protein 1 OS=Escherichia coli (strain K12) OX=83333 GN=fryA PE=1 SV=1

Query	Observed	Mr(expt)	Mr(calc)	ppm	Miss	Score	Expect	Rank	Unique	Peptide
11359	849.0838	2544.2297	2544.2134	6.39	0	24	0.025	1	U	R.LMTEQHQGLGAAIISNMEQVCAK.L

---

15. 3::P06149|DLD_ECOLI Mass: 64914 Score: 24 Matches: 2(2) Sequences: 1(1) emPAI: 0.07

Quinone-dependent D-lactate dehydrogenase OS=Escherichia coli (strain K12) OX=83333 GN=dld PE=1 SV=3

Query	Observed	Mr(expt)	Mr(calc)	ppm	Miss	Score	Expect	Rank	Unique	Peptide
758	437.7350	873.4555	873.4556	-0.11	1	23	0.008	1	U	K.LDDDRIK.D 759

---

16. 3::P28722|YHCA_ECOLI Mass: 25323 Score: 23 Matches: 1(1) Sequences: 1(1) emPAI: 0.18
Uncharacterized fimbrial chaperone YhcA OS=Escherichia coli (strain K12) OX=83333 GN=yhcA PE=3 SV=2

Query	Observed	Mr(expt)	Mr(calc)	ppm	Miss	Score	Expect	Rank	Unique	Peptide
14028	999.8786	4994.3566	4994.3418	2.97	3	23	0.04	1	U	M.LRHITFIVFITTSMNTLATGMVPETSVLLVDEKRGEASINIK.N

17. 3::Q46857|DKGA_ECOLI Mass: 31147 Score: 23 Matches: 1(1) Sequences: 1(1) emPAI: 0.15
2,5-diketo-D-gluconic acid reductase A OS=Escherichia coli (strain K12) OX=83333 GN=dkgA PE=1 SV=3

Query	Observed	Mr(expt)	Mr(calc)	ppm	Miss	Score	Expect	Rank	Unique	Peptide
1066	466.2461	930.4777	930.4844	-7.23	0	23	0.0054	1	U	-.MANPTVIK.L

18. 3::P28630|HOLA_ECOLI Mass: 38965 Score: 23 Matches: 1(1) Sequences: 1(1) emPAI: 0.12
DNA polymerase III subunit delta OS=Escherichia coli (strain K12) OX=83333 GN=holA PE=1 SV=1

Query	Observed	Mr(expt)	Mr(calc)	ppm	Miss	Score	Expect	Rank	Unique	Peptide
1049	464.7828	927.5509	927.5502	0.85	0	23	0.0059	1	U	R.QAVQLLTR.T

19. 1::sp|RS27A_HUMAN| Mass: 18296 Score: 22 Matches: 1(1) Sequences: 1(1) emPAI: 0.26
sp|RS27A_HUMAN|

Query	Observed	Mr(expt)	Mr(calc)	ppm	Miss	Score	Expect	Rank	Unique	Peptide
8272	894.4670	1786.9194	1786.9200	-0.32	0	22	0.022	1	U	K.TITLEVEPSDTIENVK.A

20. 3::Q46793|YGEN_ECOLI Mass: 27575 Score: 21 Matches: 1(1) Sequences: 1(1) emPAI: 0.17
Putative uncharacterized protein YgeN OS=Escherichia coli (strain K12) OX=83333 GN=ygeN PE=5 SV=3

Query	Observed	Mr(expt)	Mr(calc)	ppm	Miss	Score	Expect	Rank	Unique	Peptide
6063	765.8946	1529.7746	1529.7710	2.36	3	21	0.021	1	U	-.MRKKIEMSLIK.S

21. 3::P0AFS9|MEPM_ECOLI Mass: 49027 Score: 20 Matches: 1(1) Sequences: 1(1) emPAI: 0.09
Murein DD-endopeptidase MepM OS=Escherichia coli (strain K12) OX=83333 GN=mepM PE=1 SV=1

Query	Observed	Mr(expt)	Mr(calc)	ppm	Miss	Score	Expect	Rank	Unique	Peptide
8884	620.6257	1858.8553	1858.8703	-8.07	2	20	0.039	1	U	R.EQSQLLGVRLRSEGK.D

22. 3::P15877|DHG_ECOLI Mass: 87091 Score: 20 Matches: 1(1) Sequences: 1(1) emPAI: 0.05
Quinoprotein glucose dehydrogenase OS=Escherichia coli (strain K12) OX=83333 GN=gcd PE=1 SV=3

Query	Observed	Mr(expt)	Mr(calc)	ppm	Miss	Score	Expect	Rank	Unique	Peptide
1603	507.2217	1012.4289	1012.4284	0.54	0	20	0.0099	1	U	R.AYNMSNGEK.L

23. 3::P69797|PTNAB_ECOLI Mass: 35026 Score: 20 Matches: 1(1) Sequences: 1(1) emPAI: 0.13
PTS system mannose-specific EIIAB component OS=Escherichia coli (strain K12) OX=83333 GN=manX PE=1 SV=2

Query	Observed	Mr(expt)	Mr(calc)	ppm	Miss	Score	Expect	Rank	Unique	Peptide
6062	765.8940	1529.7735	1529.7598	8.98	2	20	0.028	1	U	K.LKMMDLISKIDK.-

24. 3::P75882|GFCD_ECOLI Mass: 78923 Score: 20 Matches: 2(2) Sequences: 1(1) emPAI: 0.06
Uncharacterized lipoprotein GfcD OS=Escherichia coli (strain K12) OX=83333 GN=gfcD PE=3 SV=1

Query	Observed	Mr(expt)	Mr(calc)	ppm	Miss	Score	Expect	Rank	Unique	Peptide
1642	509.2826	1016.5505	1016.5502	0.33	1	19	0.019	1	U	K.DKGATIDVAK.R 1643

25. 3::P04152|UMUC_ECOLI Mass: 48276 Score: 20 Matches: 1(1) Sequences: 1(1) emPAI: 0.09
Protein UmuC OS=Escherichia coli (strain K12) OX=83333 GN=umuC PE=1 SV=2

Query	Observed	Mr(expt)	Mr(calc)	ppm	Miss	Score	Expect	Rank	Unique	Peptide
755	437.2432	872.4719	872.4716	0.42	0	20	0.017	1	U	R.DIINAATR.S

26. 3::P20966|PTFBC_ECOLI Mass: 57824 Score: 19 Matches: 1(1) Sequences: 1(1) emPAI: 0.08
PTS system fructose-specific EIIB'BC component OS=Escherichia coli (strain K12) OX=83333 GN=fruA PE=1 SV=1

Query	Observed	Mr(expt)	Mr(calc)	ppm	Miss	Score	Expect	Rank	Unique	Peptide
419	402.7089	803.4032	803.4025	0.90	0	19	0.011	1	U	K.TAQELDK.A

27. 3::P77739|KT3K_ECOLI Mass: 32666 Score: 19 Matches: 1(1) Sequences: 1(1) emPAI: 0.14
Probable ketoamine kinase YniA OS=Escherichia coli (strain K12) OX=83333 GN=yniA PE=3 SV=1

Query	Observed	Mr(expt)	Mr(calc)	ppm	Miss	Score	Expect	Rank	Unique	Peptide
1083	467.2354	932.4562	932.4538	2.57	0	19	0.011	1	U	-.MWQAISR.L

28. 3::P31828|PQQL_ECOLI Mass: 104706 Score: 19 Matches: 1(1) Sequences: 1(1) emPAI: 0.04
Probable zinc protease PqqL OS=Escherichia coli (strain K12) OX=83333 GN=pqqL PE=1 SV=2

Query	Observed	Mr(expt)	Mr(calc)	ppm	Miss	Score	Expect	Rank	Unique	Peptide
4857	707.3304	1412.6462	1412.6371	6.48	0	19	0.023	1	U	K.QMTVENVNTAVK.Q

29. 3::P0ACP5|GNTR_ECOLI Mass: 36570 Score: 19 Matches: 1(1) Sequences: 1(1) emPAI: 0.12
HTH-type transcriptional regulator GntR OS=Escherichia coli (strain K12) OX=83333 GN=gntR PE=1 SV=1

Query	Observed	Mr(expt)	Mr(calc)	ppm	Miss	Score	Expect	Rank	Unique	Peptide
664	428.7663	855.5179	855.5178	0.18	0	19	0.012	1	U	R.LASVLTPR.E

30. 3::P0A8N3|SYK1_ECOLI Mass: 57624 Score: 19 Matches: 2(2) Sequences: 2(2) emPAI: 0.16
Lysine--tRNA ligase OS=Escherichia coli (strain K12) OX=83333 GN=lysS PE=1 SV=2

Query	Observed	Mr(expt)	Mr(calc)	ppm	Miss	Score	Expect	Rank	Unique	Peptide
2234	363.2009	1086.5810	1086.5710	9.24	0	19	0.02	1	U	K.WDLGDILGAK.G
2613	376.8961	1127.6663	1127.6775	-9.89	1	14	0.044	2	U	K.VRSQILSGIR.Q

31. 3::P76555|EUTQ_ECOLI Mass: 25516 Score: 19 Matches: 1(1) Sequences: 1(1) emPAI: 0.18
Acetate kinase EutQ OS=Escherichia coli (strain K12) OX=83333 GN=eutQ PE=1 SV=1

Query	Observed	Mr(expt)	Mr(calc)	ppm	Miss	Score	Expect	Rank	Unique	Peptide
8326	896.4427	1790.8708	1790.8580	7.13	2	19	0.048	1	U	K.SVTGKGGIKVIDGSSVK.F

32. 3::P0AEX9|MALE_ECOLI Mass: 43360 Score: 19 Matches: 1(1) Sequences: 1(1) emPAI: 0.10
Maltose/maltodextrin-binding periplasmic protein OS=Escherichia coli (strain K12) OX=83333 GN=malE PE=1 SV=1

Query	Observed	Mr(expt)	Mr(calc)	ppm	Miss	Score	Expect	Rank	Unique	Peptide
900	452.2428	902.4711	902.4709	0.19	0	19	0.026	1	U	R.QTVDEALK.D

33. 3::Q46861|YGIQ_ECOLI Mass: 84223 Score: 19 Matches: 1(1) Sequences: 1(1) emPAI: 0.05
UPF0313 protein YgiQ OS=Escherichia coli (strain K12) OX=83333 GN=ygiQ PE=3 SV=3

Query	Observed	Mr(expt)	Mr(calc)	ppm	Miss	Score	Expect	Rank	Unique	Peptide
6614	528.5888	1582.7445	1582.7449	-0.22	1	19	0.04	1	U	K.TYVLLPSFEKVK.G

34. 3::P0ACY1|YDJA_ECOLI Mass: 20217 Score: 19 Matches: 1(1) Sequences: 1(1) emPAI: 0.23
Putative NAD(P)H nitroreductase YdjA OS=Escherichia coli (strain K12) OX=83333 GN=ydjA PE=1 SV=1

Query	Observed	Mr(expt)	Mr(calc)	ppm	Miss	Score	Expect	Rank	Unique	Peptide
8068	880.9706	1759.9266	1759.9362	-5.49	2	19	0.035	1	U	-.MDALELLINRRSASR.L

35. 3::P63224|GMHA_ECOLI Mass: 20973 Score: 18 Matches: 1(1) Sequences: 1(1) emPAI: 0.22
Phosphoheptose isomerase OS=Escherichia coli (strain K12) OX=83333 GN=gmhA PE=1 SV=1

Query	Observed	Mr(expt)	Mr(calc)	ppm	Miss	Score	Expect	Rank	Unique	Peptide
5154	725.3482	1448.6818	1448.6929	-7.71	1	18	0.033	1	U	K.DGGKMAGTADIEIR.V

36. 3::V9HVX0|YPAA_ECOLI Mass: 6876 Score: 18 Matches: 1(1) Sequences: 1(1) emPAI: 0.80
Uncharacterized protein YpaA OS=Escherichia coli (strain K12) OX=83333 GN=ypaA PE=4 SV=1

Query	Observed	Mr(expt)	Mr(calc)	ppm	Miss	Score	Expect	Rank	Unique	Peptide
5318	732.8890	1463.7635	1463.7555	5.51	1	18	0.043	1	U	K.IALRMLEQGFDR.D

37. 3::P0A6H5|HSLU_ECOLI Mass: 49677 Score: 18 Matches: 1(1) Sequences: 1(1) emPAI: 0.09
ATP-dependent protease ATPase subunit HslU OS=Escherichia coli (strain K12) OX=83333 GN=hslU PE=1 SV=1

Query	Observed	Mr(expt)	Mr(calc)	ppm	Miss	Score	Expect	Rank	Unique	Peptide
535	416.7247	831.4348	831.4338	1.27	0	18	0.017	1	U	R.DLTDAAVK.M

38. 3::P02943|LAMB_ECOLI Mass: 49995 Score: 18 Matches: 1(1) Sequences: 1(1) emPAI: 0.09
Maltoporin OS=Escherichia coli (strain K12) OX=83333 GN=lamB PE=1 SV=1

Query	Observed	Mr(expt)	Mr(calc)	ppm	Miss	Score	Expect	Rank	Unique	Peptide
113	366.2242	730.4338	730.4337	0.15	0	18	0.017	1	U	K.LSLAATR.S

39. 3::P52101|GLRK_ECOLI Mass: 53639 Score: 18 Matches: 1(1) Sequences: 1(1) emPAI: 0.08
Sensor histidine kinase GlrK OS=Escherichia coli (strain K12) OX=83333 GN=glrK PE=1 SV=3

Query	Observed	Mr(expt)	Mr(calc)	ppm	Miss	Score	Expect	Rank	Unique	Peptide
94	364.1959	726.3772	726.3773	-0.04	0	18	0.017	1	U	R.SSLHGAR.V

40. 3::P68644|FIXC_ECOLI Mass: 45902 Score: 18 Matches: 1(1) Sequences: 1(1) emPAI: 0.10
Protein FixC OS=Escherichia coli (strain K12) OX=83333 GN=fixC PE=3 SV=1

Query	Observed	Mr(expt)	Mr(calc)	ppm	Miss	Score	Expect	Rank	Unique	Peptide
8291	447.9621	1787.8193	1787.8165	1.57	1	18	0.037	1	U	R.DLFTIDGSAPELMRK.K

41. 3::P77766|RNAAM_ECOLI Mass: 32902 Score: 17 Matches: 1(1) Sequences: 1(1) emPAI: 0.14
5'-3' exoribonuclease OS=Escherichia coli (strain K12) OX=83333 GN=yciV PE=1 SV=1

Query	Observed	Mr(expt)	Mr(calc)	ppm	Miss	Score	Expect	Rank	Unique	Peptide
397	400.7352	799.4559	799.4552	0.87	0	17	0.02	2	U	R.AQLIAER.L

42. 3::P80645|SSUD_ECOLI Mass: 41768 Score: 17 Matches: 1(1) Sequences: 1(1) emPAI: 0.11
Alkanesulfonate monooxygenase OS=Escherichia coli (strain K12) OX=83333 GN=ssuD PE=1 SV=3

Query	Observed	Mr(expt)	Mr(calc)	ppm	Miss	Score	Expect	Rank	Unique	Peptide
296	387.7090	773.4034	773.4032	0.33	0	17	0.018	1	U	R.QAATLDR.L

43. 3::P75958|LOLE_ECOLI Mass: 45429 Score: 17 Matches: 1(1) Sequences: 1(1) emPAI: 0.10
Lipoprotein-releasing system transmembrane protein LolE OS=Escherichia coli (strain K12) OX=83333 GN=lolE PE=1 SV=3

Query	Observed	Mr(expt)	Mr(calc)	ppm	Miss	Score	Expect	Rank	Unique	Peptide
4299	443.2331	1326.6775	1326.6892	-8.81	1	17	0.04	1	U	R.ASNIDPARVLSGQ.-

44. 3::P76399|MDTC_ECOLI Mass: 111168 Score: 17 Matches: 1(1) Sequences: 1(1) emPAI: 0.04
Multidrug resistance protein MdtC OS=Escherichia coli (strain K12) OX=83333 GN=mdtC PE=1 SV=1

Query	Observed	Mr(expt)	Mr(calc)	ppm	Miss	Score	Expect	Rank	Unique	Peptide
13433	1163.2966	3486.8679	3486.8455	6.41	2	17	0.043	1	U	R.NAGMTNAKPAILLMIRKLPEANIIQTVDSIR.A

45. 3::P0AEM0|FKBX_ECOLI Mass: 16071 Score: 17 Matches: 1(1) Sequences: 1(1) emPAI: 0.30
FKBP-type 16 kDa peptidyl-prolyl cis-trans isomerase OS=Escherichia coli (strain K12) OX=83333 GN=fkpB PE=1 SV=2

Query	Observed	Mr(expt)	Mr(calc)	ppm	Miss	Score	Expect	Rank	Unique	Peptide
13461	881.5934	3522.3444	3522.3506	-1.76	1	17	0.039	1	U	-.MSESVQSNSAVLVHFTLKLDDGTTAESTR.N

46. 3::P0AC88|GM4D_ECOLI Mass: 42191 Score: 17 Matches: 1(1) Sequences: 1(1) emPAI: 0.11
GDP-mannose 4,6-dehydratase OS=Escherichia coli (strain K12) OX=83333 GN=gmd PE=1 SV=1

Query	Observed	Mr(expt)	Mr(calc)	ppm	Miss	Score	Expect	Rank	Unique	Peptide
134	368.2053	734.3961	734.3963	-0.19	0	17	0.019	1	U	R.SPYAVAK.L

47. 3::A0A7H2C769|A0A7H2C769_ECOLI Mass: 37383 Score: 17 Matches: 1(1) Sequences: 1(1) emPAI: 0.12
Putative type II secretion system C-type protein YghF OS=Escherichia coli (strain K12) OX=83333 GN=yghF PE=3 SV=1

Query	Observed	Mr(expt)	Mr(calc)	ppm	Miss	Score	Expect	Rank	Unique	Peptide
5116	722.3780	1442.7413	1442.7323	6.28	0	17	0.046	1	U	K.IFNYIQLTPVR.K

Proteins matching the same set of peptides:
3::Q46834|YGHF_ECOLI Mass: 37383 Score: 17 Matches: 1(1) Sequences: 1(1)
Putative type II secretion system C-type protein YghF OS=Escherichia coli (strain K12) OX=83333 GN=yghF PE=3 SV=2

48. 3::P0A840|SURE_ECOLI Mass: 27169 Score: 17 Matches: 1(1) Sequences: 1(1) emPAI: 0.17
5'/3'-nucleotidase SurE OS=Escherichia coli (strain K12) OX=83333 GN=surE PE=1 SV=1

Query	Observed	Mr(expt)	Mr(calc)	ppm	Miss	Score	Expect	Rank	Unique	Peptide
10700	577.5629	2306.2224	2306.2052	7.45	1	17	0.05	1	U	-.MRILLSNDDGVHAPGIQTLAK.A

49. 3::P21893|RECJ_ECOLI Mass: 63634 Score: 17 Matches: 1(1) Sequences: 1(1) emPAI: 0.07
Single-stranded-DNA-specific exonuclease RecJ OS=Escherichia coli (strain K12) OX=83333 GN=recJ PE=1 SV=2

Query	Observed	Mr(expt)	Mr(calc)	ppm	Miss	Score	Expect	Rank	Unique	Peptide
2228	543.8046	1085.5947	1085.6015	-6.31	1	17	0.037	1	U	-.MKQQIQLR.R

50. 3::P30852|SMF_ECOLI Mass: 41158 Score: 17 Matches: 1(1) Sequences: 1(1) emPAI: 0.11
Protein Smf OS=Escherichia coli (strain K12) OX=83333 GN=smf PE=3 SV=1

Query	Observed	Mr(expt)	Mr(calc)	ppm	Miss	Score	Expect	Rank	Unique	Peptide
730	435.7076	869.4007	869.4008	-0.14	0	17	0.025	1	U	R.SGSLVTAR.C

51. 3::P33668|YBBC_ECOLI Mass: 14257 Score: 17 Matches: 1(1) Sequences: 1(1) emPAI: 0.34
Uncharacterized protein YbbC OS=Escherichia coli (strain K12) OX=83333 GN=ybbC PE=2 SV=1

Query	Observed	Mr(expt)	Mr(calc)	ppm	Miss	Score	Expect	Rank	Unique	Peptide
1649	509.7495	1017.4845	1017.4896	-5.04	1	17	0.032	1	U	K.YSVKNTVK.T

52. 3::P38035|RTCR_ECOLI Mass: 60604 Score: 16 Matches: 1(1) Sequences: 1(1) emPAI: 0.07
Transcriptional regulatory protein RtcR OS=Escherichia coli (strain K12) OX=83333 GN=rtcR PE=4 SV=2

Query	Observed	Mr(expt)	Mr(calc)	ppm	Miss	Score	Expect	Rank	Unique	Peptide
1216	479.2299	956.4453	956.4386	7.03	0	16	0.035	1	U	R.MATFATSGR.I

53. 3::P23871|HEMH_ECOLI Mass: 36089 Score: 16 Matches: 1(1) Sequences: 1(1) emPAI: 0.12
Ferrochelatase OS=Escherichia coli (strain K12) OX=83333 GN=hemH PE=1 SV=3

Query	Observed	Mr(expt)	Mr(calc)	ppm	Miss	Score	Expect	Rank	Unique	Peptide
4465	451.9139	1352.7199	1352.7329	-9.64	2	16	0.034	1	U	R.KRSIPGISFIR.D

54. 3::P00634|PPB_ECOLI Mass: 49636 Score: 16 Matches: 1(1) Sequences: 1(1) emPAI: 0.09
Alkaline phosphatase OS=Escherichia coli (strain K12) OX=83333 GN=phoA PE=1 SV=1

Query	Observed	Mr(expt)	Mr(calc)	ppm	Miss	Score	Expect	Rank	Unique	Peptide
3426	614.2892	1226.5638	1226.5601	2.99	1	16	0.046	1	U	R.KCYGPSATSEK.C

55. 3::Q46864|MQSA_ECOLI Mass: 14922 Score: 16 Matches: 1(1) Sequences: 1(1) emPAI: 0.32
Antitoxin MqsA OS=Escherichia coli (strain K12) OX=83333 GN=mqsA PE=1 SV=1

Query	Observed	Mr(expt)	Mr(calc)	ppm	Miss	Score	Expect	Rank	Unique	Peptide
862	449.2430	896.4714	896.4732	-2.03	1	16	0.025	1	U	K.KLSLTQK.E

56. 1::sp|DHE3_BOVIN| Mass: 61815 Score: 16 Matches: 1(1) Sequences: 1(1) emPAI: 0.07
sp|DHE3_BOVIN|

Query	Observed	Mr(expt)	Mr(calc)	ppm	Miss	Score	Expect	Rank	Unique	Peptide
282	386.1642	770.3139	770.3211	-9.31	0	16	0.025	1	U	R.ISGASEK.D

57. 3::P0AG24|SPOT_ECOLI Mass: 79748 Score: 16 Matches: 2(2) Sequences: 1(1) emPAI: 0.06
Bifunctional (p)ppGpp synthase/hydrolase SpoT OS=Escherichia coli (strain K12) OX=83333 GN=spoT PE=1 SV=1

Query	Observed	Mr(expt)	Mr(calc)	ppm	Miss	Score	Expect	Rank	Unique	Peptide
874	450.2146	898.4147	898.4201	-6.03	0	15	0.033	1	U	K.DYIAIPK.A 873

58. 3::P37650|BCSC_ECOLI Mass: 127760 Score: 16 Matches: 1(1) Sequences: 1(1) emPAI: 0.03
Cellulose synthase operon protein C OS=Escherichia coli (strain K12) OX=83333 GN=bcsC PE=1 SV=3

Query	Observed	Mr(expt)	Mr(calc)	ppm	Miss	Score	Expect	Rank	Unique	Peptide
1350	488.7422	975.4698	975.4733	-3.58	1	16	0.037	1	U	K.SNAGREGASK.I

59. 3::P39277|YJEH_ECOLI Mass: 45261 Score: 15 Matches: 1(1) Sequences: 1(1) emPAI: 0.10
L-methionine/branched-chain amino acid exporter YjeH OS=Escherichia coli (strain K12) OX=83333 GN=yjeH PE=1 SV=1

Query	Observed	Mr(expt)	Mr(calc)	ppm	Miss	Score	Expect	Rank	Unique	Peptide
1251	480.7538	959.4929	959.4924	0.60	1	15	0.044	1	U	R.KTPENGITT.-

60. 3::P77293|GTRB_ECOLI Mass: 34614 Score: 15 Matches: 1(1) Sequences: 1(1) emPAI: 0.13
Prophage bactoprenol glucosyl transferase homolog OS=Escherichia coli (strain K12) OX=83333 GN=yfdH PE=1 SV=1

Query	Observed	Mr(expt)	Mr(calc)	ppm	Miss	Score	Expect	Rank	Unique	Peptide
457	408.7265	815.4385	815.4389	-0.42	0	15	0.031	1	U	R.DVVENIK.L

61. 3::P36678|GSPM_ECOLI Mass: 17280 Score: 15 Matches: 1(1) Sequences: 1(1) emPAI: 0.27
Putative type II secretion system protein M OS=Escherichia coli (strain K12) OX=83333 GN=gspM PE=2 SV=2

Query	Observed	Mr(expt)	Mr(calc)	ppm	Miss	Score	Expect	Rank	Unique	Peptide
2219	543.3199	1084.6252	1084.6240	1.09	1	15	0.042	1	U	K.NILLEEAKR.E

62. 3::P16869|FHUE_ECOLI Mass: 81239 Score: 15 Matches: 1(1) Sequences: 1(1) emPAI: 0.05
FhuE receptor OS=Escherichia coli (strain K12) OX=83333 GN=fhuE PE=1 SV=2

Query	Observed	Mr(expt)	Mr(calc)	ppm	Miss	Score	Expect	Rank	Unique	Peptide
662	428.6925	855.3704	855.3739	-4.08	0	15	0.033	1	U	K.AVDGTVSK.G

63. 3::P76161|REQ2_ECOLI Mass: 28478 Score: 15 Matches: 1(1) Sequences: 1(1) emPAI: 0.16
Prophage antitermination protein Q homolog QuuQ OS=Escherichia coli (strain K12) OX=83333 GN=quuQ PE=3 SV=2

Query	Observed	Mr(expt)	Mr(calc)	ppm	Miss	Score	Expect	Rank	Unique	Peptide
1066	466.2461	930.4777	930.4844	-7.21	0	15	0.033	2	U	-.MNLEALPK.Y

64. 3::P64570|YQGC_ECOLI Mass: 8166 Score: 15 Matches: 1(1) Sequences: 1(1) emPAI: 0.64
Protein YqgC OS=Escherichia coli (strain K12) OX=83333 GN=yqgC PE=1 SV=1

Query	Observed	Mr(expt)	Mr(calc)	ppm	Miss	Score	Expect	Rank	Unique	Peptide
3472	617.7462	1233.4779	1233.4883	-8.45	0	15	0.033	1	U	-.MGITSAGMQSR.D

65. 3::P77732|RHMR_ECOLI Mass: 29228 Score: 15 Matches: 1(1) Sequences: 1(1) emPAI: 0.16
Uncharacterized HTH-type transcriptional regulator RhmR OS=Escherichia coli (strain K12) OX=83333 GN=rhmR PE=1 SV=1

Query	Observed	Mr(expt)	Mr(calc)	ppm	Miss	Score	Expect	Rank	Unique	Peptide
706	432.7089	863.4033	863.4025	0.89	0	15	0.034	1	U	K.SPFDLQAS.-

66. 3::P25772|LIGB_ECOLI Mass: 63425 Score: 14 Matches: 1(1) Sequences: 1(1) emPAI: 0.07
DNA ligase B OS=Escherichia coli (strain K12) OX=83333 GN=ligB PE=1 SV=2

Query	Observed	Mr(expt)	Mr(calc)	ppm	Miss	Score	Expect	Rank	Unique	Peptide
150	372.7215	743.4285	743.4290	-0.62	0	14	0.036	1	U	R.VNIGSVR.R

---

67. 3::P75977|YMFM_ECOLI Mass: 12319 Score: 14 Matches: 1(1) Sequences: 1(1) emPAI: 0.40

Uncharacterized protein YmfM OS=Escherichia coli (strain K12) OX=83333 GN=ymfM PE=4 SV=1

Query	Observed	Mr(expt)	Mr(calc)	ppm	Miss	Score	Expect	Rank	Unique	Peptide
2462	371.8297	1112.4672	1112.4652	1.87	0	14	0.045	1	U	M.NSLTTHYR.R

---

68. 3::P77658|YNAA_ECOLI Mass: 37094 Score: 14 Matches: 1(1) Sequences: 1(1) emPAI: 0.12

Protein YnaA OS=Escherichia coli (strain K12) OX=83333 GN=ynaA PE=4 SV=1

Query	Observed	Mr(expt)	Mr(calc)	ppm	Miss	Score	Expect	Rank	Unique	Peptide
1063	465.7797	929.5448	929.5406	4.53	2	14	0.037	1	U	R.KIQETRR.E

---

69. 3::P0A7R9|RS11_ECOLI Mass: 13950 Score: 14 Matches: 1(1) Sequences: 1(1) emPAI: 0.35

30S ribosomal protein S11 OS=Escherichia coli (strain K12) OX=83333 GN=rpsK PE=1 SV=2

Query	Observed	Mr(expt)	Mr(calc)	ppm	Miss	Score	Expect	Rank	Unique	Peptide
350	393.7395	785.4644	785.4581	7.92	1	14	0.038	1	U	-.MAKAPIR.A

---

70. 3::P00957|SYA_ECOLI Mass: 96315 Score: 14 Matches: 1(1) Sequences: 1(1) emPAI: 0.05

Alanine--tRNA ligase OS=Escherichia coli (strain K12) OX=83333 GN=alaS PE=1 SV=2

Query	Observed	Mr(expt)	Mr(calc)	ppm	Miss	Score	Expect	Rank	Unique	Peptide
491	412.2393	822.4640	822.4712	-8.69	0	14	0.039	1	U	R.VIADHIR.S

---

71. 3::P07813|SYL_ECOLI Mass: 97799 Score: 14 Matches: 1(1) Sequences: 1(1) emPAI: 0.04

Leucine--tRNA ligase OS=Escherichia coli (strain K12) OX=83333 GN=leuS PE=1 SV=2

Query	Observed	Mr(expt)	Mr(calc)	ppm	Miss	Score	Expect	Rank	Unique	Peptide
712	433.2287	864.4428	864.4388	4.61	0	14	0.04	1	U	R.LHMGHVR.N

---

72. 3::P0ACZ8|CUSR_ECOLI Mass: 25378 Score: 14 Matches: 1(1) Sequences: 1(1) emPAI: 0.18

Transcriptional regulatory protein CusR OS=Escherichia coli (strain K12) OX=83333 GN=cusR PE=1 SV=1

Query	Observed	Mr(expt)	Mr(calc)	ppm	Miss	Score	Expect	Rank	Unique	Peptide
1357	489.2346	976.4547	976.4502	4.63	0	14	0.04	1	U	K.IDNDFEPK.L

---

73. 3::P24215|UXUA_ECOLI Mass: 44981 Score: 14 Matches: 1(1) Sequences: 1(1) emPAI: 0.10

Mannonate dehydratase OS=Escherichia coli (strain K12) OX=83333 GN=uxuA PE=1 SV=2

Query	Observed	Mr(expt)	Mr(calc)	ppm	Miss	Score	Expect	Rank	Unique	Peptide
818	443.2834	884.5523	884.5443	9.02	1	14	0.041	1	U	R.LKGLAEVR.G

---

74. 3::P38134|ETK_ECOLI Mass: 81191 Score: 14 Matches: 1(1) Sequences: 1(1) emPAI: 0.05

Tyrosine-protein kinase etk OS=Escherichia coli (strain K12) OX=83333 GN=etk PE=1 SV=2

Query	Observed	Mr(expt)	Mr(calc)	ppm	Miss	Score	Expect	Rank	Unique	Peptide
2422	555.2141	1108.4135	1108.4025	9.97	0	14	0.042	1	U	K.EVSLSMQR.L

---

75. 3::P0ADT8|YGIM_ECOLI Mass: 23062 Score: 14 Matches: 1(1) Sequences: 1(1) emPAI: 0.20

Uncharacterized protein YgiM OS=Escherichia coli (strain K12) OX=83333 GN=ygiM PE=3 SV=1

Query	Observed	Mr(expt)	Mr(calc)	ppm	Miss	Score	Expect	Rank	Unique	Peptide
2937	580.7940	1159.5734	1159.5721	1.13	0	14	0.049	1	U	K.VDAASVQLDDK.Q

---

76. 3::P0AFA2|NARX_ECOLI Mass: 67669 Score: 13 Matches: 1(1) Sequences: 1(1) emPAI: 0.07

Nitrate/nitrite sensor protein NarX OS=Escherichia coli (strain K12) OX=83333 GN=narX PE=1 SV=1

Query	Observed	Mr(expt)	Mr(calc)	ppm	Miss	Score	Expect	Rank	Unique	Peptide
196	378.2061	754.3976	754.3973	0.36	0	13	0.047	1	U	K.TAGLEHK.N

---

77. 3::P20099|BISC_ECOLI Mass: 86366 Score: 13 Matches: 1(1) Sequences: 1(1) emPAI: 0.05

Biotin sulfoxide reductase OS=Escherichia coli (strain K12) OX=83333 GN=bisC PE=1 SV=3

Query	Observed	Mr(expt)	Mr(calc)	ppm	Miss	Score	Expect	Rank	Unique	Peptide
254	384.6712	767.3278	767.3232	6.08	0	13	0.047	1	U	-.MANSSSR.Y

---

78. 3::P33362|YEHZ_ECOLI Score: 13 Matches: 1(1) Sequences: 1(1)

Glycine betaine-binding protein YehZ OS=Escherichia coli (strain K12) OX=83333 GN=yehZ PE=1 SV=1

Query	Observed	Mr(expt)	Mr(calc)	ppm	Miss	Score	Expect	Rank	Unique	Peptide
1066	466.2461	930.4777	930.4770	0.72	1	13	0.048	3	U	R.QDVAEKNK.L

---

79. 3::P39358|YJHG_ECOLI Score: 13 Matches: 1(1) Sequences: 1(1)

D-xylonate dehydratase YjhG OS=Escherichia coli (strain K12) OX=83333 GN=yjhG PE=1 SV=2

Query	Observed	Mr(expt)	Mr(calc)	ppm	Miss	Score	Expect	Rank	Unique	Peptide
150	372.7215	743.4285	743.4290	-0.62	0	13	0.049	2	U	K.VQTIGAR.F

Proteins matching the same set of peptides:

3::P77596|YAGF_ECOLI Mass: 69926 Score: 13 Matches: 1(1) Sequences: 1(1)

D-xylonate dehydratase YagF OS=Escherichia coli (strain K12) OX=83333 GN=yagF PE=1 SV=1
