# Supplementary material for: ERK2-topoisomerase II regulatory axis is important for gene activation in immediate early genes
Source: Nat Commun. 2023 Dec 14;14:8341. doi: 10.1038/s41467-023-44089-y (PMC10721843; doi:10.1038/s41467-023-44089-y)
Supplement: Supplementary file 11 — Supplementary Data 8 [file 41467_2023_44089_MOESM11_ESM.pdf]

Mascot Search Results

User	r-nakagawa
Email	
Search title	SN221365_deTOP2B_Erk1ml_1ul_Node:2
MS data file	File Name: Z:¥SN22¥SN221365_deTOP2B_Erk1ml_1ul.raw;File Path: ;File Time: 3/1/2023 3:35:46 PM;File Size: 351320790 [Byte]
Database 1	cRAP STRAV_proteinG_20230313 (118 sequences; 39090 residues)
Database 2	Original_21 21_TOP2B_1-566_20210618 (1 sequences; 589 residues)
Database 3	Uniprot_Proteome_E.coli_K12 Proteome_E.coli_K12_UP000000625_2022_01 (4448 sequences; 1358110 residues)
Timestamp	13 Sep 2023 at 00:28:03 GMT
Enzyme	Trypsin
Fixed modifications	Carbamidomethyl (C)
Variable modifications	Acetyl (Protein N-term), Oxidation (M), Phospho (ST), Phospho (Y)
Mass values	Monoisotopic
Protein Mass	Unrestricted
Peptide Mass Tolerance	± 10 ppm
Fragment Mass Tolerance	± 0.8 Da
Max Missed Cleavages	3
Instrument type	Default
Number of queries	13219

Protein hits:

2::TOP2B_1-566	TOP2B_1-566
1::sp|K1C10_HUMAN|	sp|K1C10_HUMAN|
1::sp|K2C1_HUMAN|	sp|K2C1_HUMAN|
1::sp|K22E_HUMAN|	sp|K22E_HUMAN|
1::sp|K1C9_HUMAN|	sp|K1C9_HUMAN|
1::sp|HBB_HUMAN|	sp|HBB_HUMAN|
1::sp|K1C15_SHEEP|	sp|K1C15_SHEEP|
3::P0AA25|THIO_ECOLI	Thioredoxin 1 OS=Escherichia coli (strain K12) OX=83333 GN=trxA PE=1 SV=2
1::sp|HBA_HUMAN|	sp|HBA_HUMAN|
1::sp|TRYP_PIG|	sp|TRYP_PIG|
1::sp|ALBU_BOVIN|	sp|ALBU_BOVIN|
3::P76298|FLHA_ECOLI	Flagellar biosynthesis protein FlhA OS=Escherichia coli (strain K12) OX=83333 GN=flhA PE=3 SV=1
1::sp|KRHB4_HUMAN|	sp|KRHB4_HUMAN|
1::sp|CASB_BOVIN|	sp|CASB_BOVIN|
1::sp|RS27A_HUMAN|	sp|RS27A_HUMAN|
3::P0AD96|LIVJ_ECOLI	Leu/Ile/Val-binding protein OS=Escherichia coli (strain K12) OX=83333 GN=livJ PE=1 SV=1
3::P77211|CUSC_ECOLI	Cation efflux system protein CusC OS=Escherichia coli (strain K12) OX=83333 GN=cusC PE=1 SV=1
3::P75882|GFCD_ECOLI	Uncharacterized lipoprotein GfcD OS=Escherichia coli (strain K12) OX=83333 GN=gfcD PE=3 SV=1
3::Q06065|ATOC_ECOLI	Regulatory protein AtoC OS=Escherichia coli (strain K12) OX=83333 GN=atoC PE=1 SV=2
3::P62601|TREF_ECOLI	Cytoplasmic trehalase OS=Escherichia coli (strain K12) OX=83333 GN=treF PE=1 SV=1
3::A0A7H2C767|A0A7H2C767_ECOLI	Chaperone protein ClpB OS=Escherichia coli (strain K12) OX=83333 GN=clpB PE=3 SV=1
3::P32719|ALSE_ECOLI	D-allulose-6-phosphate 3-epimerase OS=Escherichia coli (strain K12) OX=83333 GN=alsE PE=1 SV=1
3::P76092|YNBC_ECOLI	Uncharacterized protein YnbC OS=Escherichia coli (strain K12) OX=83333 GN=ynbC PE=1 SV=1
3::Q46857|DKGA_ECOLI	2,5-diketo-D-gluconic acid reductase A OS=Escherichia coli (strain K12) OX=83333 GN=dkgA PE=1 SV=3
3::P0ABH0|FTSA_ECOLI	Cell division protein FtsA OS=Escherichia coli (strain K12) OX=83333 GN=ftsA PE=1 SV=1
3::P12996|BIOB_ECOLI	Biotin synthase OS=Escherichia coli (strain K12) OX=83333 GN=bioB PE=1 SV=2
3::P39358|YJHG_ECOLI	D-xylonate dehydratase YjhG OS=Escherichia coli (strain K12) OX=83333 GN=yjhG PE=1 SV=2
3::P21361|YCIG_ECOLI	Uncharacterized protein YciG OS=Escherichia coli (strain K12) OX=83333 GN=yciG PE=3 SV=1
3::P12008|AROC_ECOLI	Chorismate synthase OS=Escherichia coli (strain K12) OX=83333 GN=aroC PE=1 SV=4
3::P71242|WCAK_ECOLI	Colanic acid biosynthesis protein WcaK OS=Escherichia coli (strain K12) OX=83333 GN=wcaK PE=3 SV=2
3::P46891|COF_ECOLI	HMP-PP phosphatase OS=Escherichia coli (strain K12) OX=83333 GN=cof PE=1 SV=2
1::sp|CYC_HUMAN|	sp|CYC_HUMAN|
3::P0A759|NAGB_ECOLI	Glucosamine-6-phosphate deaminase OS=Escherichia coli (strain K12) OX=83333 GN=nagB PE=1 SV=1
3::P0C018|RL18_ECOLI	50S ribosomal protein L18 OS=Escherichia coli (strain K12) OX=83333 GN=rplR PE=1 SV=1
3::P0A7V0|RS2_ECOLI	30S ribosomal protein S2 OS=Escherichia coli (strain K12) OX=83333 GN=rpsB PE=1 SV=2
3::P0ACP5|GNTR_ECOLI	HTH-type transcriptional regulator GntR OS=Escherichia coli (strain K12) OX=83333 GN=gntR PE=1 SV=1
3::P32053|INTA_ECOLI	Prophage integrase IntA OS=Escherichia coli (strain K12) OX=83333 GN=intA PE=1 SV=2
3::P06149|DLD_ECOLI	Quinone-dependent D-lactate dehydrogenase OS=Escherichia coli (strain K12) OX=83333 GN=dld PE=1 SV=3
3::P0ADT8|YGIM_ECOLI	Uncharacterized protein YgiM OS=Escherichia coli (strain K12) OX=83333 GN=ygiM PE=3 SV=1
3::P63201|GADW_ECOLI	HTH-type transcriptional regulator GadW OS=Escherichia coli (strain K12) OX=83333 GN=gadW PE=1 SV=1
3::P22106|ASNB_ECOLI	Asparagine synthetase B [glutamine-hydrolyzing] OS=Escherichia coli (strain K12) OX=83333 GN=asnB PE=1 SV=3
3::P15029|FECD_ECOLI	Fe(3+) dicitrate transport system permease protein FecD OS=Escherichia coli (strain K12) OX=83333 GN=fecD PE=1 SV=2
3::P21889|SYD_ECOLI	Aspartate--tRNA ligase OS=Escherichia coli (strain K12) OX=83333 GN=aspS PE=1 SV=1
3::P76104|RLHA_ECOLI	23S rRNA 5-hydroxycytidine C2501 synthase OS=Escherichia coli (strain K12) OX=83333 GN=rlhA PE=1 SV=2
3::P21893|RECJ_ECOLI	Single-stranded-DNA-specific exonuclease RecJ OS=Escherichia coli (strain K12) OX=83333 GN=recJ PE=1 SV=2
3::P38035|RTCR_ECOLI	Transcriptional regulatory protein RtcR OS=Escherichia coli (strain K12) OX=83333 GN=rtcR PE=4 SV=2
3::P80645|SSUD_ECOLI	Alkanesulfonate monooxygenase OS=Escherichia coli (strain K12) OX=83333 GN=ssuD PE=1 SV=3
3::P0AAJ8|HYBA_ECOLI	Hydrogenase-2 operon protein HybA OS=Escherichia coli (strain K12) OX=83333 GN=hybA PE=3 SV=1
3::P77330|BORD_ECOLI	Prophage lipoprotein Bor homolog OS=Escherichia coli (strain K12) OX=83333 GN=borD PE=3 SV=1
3::Q46901|CSE1_ECOLI	CRISPR system Cascade subunit CasA OS=Escherichia coli (strain K12) OX=83333 GN=casA PE=1 SV=1
3::P0AA43|RSUA_ECOLI	Ribosomal small subunit pseudouridine synthase A OS=Escherichia coli (strain K12) OX=83333 GN=rsuA PE=1 SV=1
3::P45522|KEFB_ECOLI	Glutathione-regulated potassium-efflux system protein KefB OS=Escherichia coli (strain K12) OX=83333 GN=kefB PE=3 SV=1
3::P77337|YDIS_ECOLI	Probable electron transfer flavoprotein-quinone oxidoreductase YdiS OS=Escherichia coli (strain K12) OX=83333 GN=ydiS PE=3 SV=1
3::P0AEX9|MALE_ECOLI	Maltose/maltodextrin-binding periplasmic protein OS=Escherichia coli (strain K12) OX=83333 GN=malE PE=1 SV=1
3::P33668|YBBC_ECOLI	Uncharacterized protein YbbC OS=Escherichia coli (strain K12) OX=83333 GN=ybbC PE=2 SV=1
3::P02921|MELB_ECOLI	Melibiose carrier protein OS=Escherichia coli (strain K12) OX=83333 GN=melB PE=1 SV=2
3::P64622|YHDV_ECOLI	Uncharacterized protein YhdV OS=Escherichia coli (strain K12) OX=83333 GN=yhdV PE=3 SV=1
3::P20966|PTFBC_ECOLI	PTS system fructose-specific EIIB'BC component OS=Escherichia coli (strain K12) OX=83333 GN=fruA PE=1 SV=1
3::P08331|CPDB_ECOLI	2',3'-cyclic-nucleotide 2'-phosphodiesterase/3'-nucleotidase OS=Escherichia coli (strain K12) OX=83333 GN=cpdB PE=1 SV=2
3::P12999|BIOC_ECOLI	Malonyl-[acyl-carrier protein] O-methyltransferase OS=Escherichia coli (strain K12) OX=83333 GN=bioC PE=1 SV=2
3::P0AG96|SECE_ECOLI	Protein translocase subunit SecE OS=Escherichia coli (strain K12) OX=83333 GN=secE PE=1 SV=1
3::P14900|MURD_ECOLI	UDP-N-acetylmuramoylalanine--D-glutamate ligase OS=Escherichia coli (strain K12) OX=83333 GN=murD PE=1 SV=3
3::P28249|ASMA_ECOLI	Protein AsmA OS=Escherichia coli (strain K12) OX=83333 GN=asmA PE=3 SV=2
3::P0ADF6|EDD_ECOLI	Phosphogluconate dehydratase OS=Escherichia coli (strain K12) OX=83333 GN=edd PE=1 SV=1
3::P0AD65|MRDA_ECOLI	Peptidoglycan D,D-transpeptidase MrdA OS=Escherichia coli (strain K12) OX=83333 GN=mrdA PE=1 SV=1
3::P0A6N8|EFPL_ECOLI	Elongation factor P-like protein OS=Escherichia coli (strain K12) OX=83333 GN=yeiP PE=1 SV=1
3::P43329|HRPA_ECOLI	ATP-dependent RNA helicase HrpA OS=Escherichia coli (strain K12) OX=83333 GN=hrpA PE=1 SV=3
3::P0AGD7|SRP54_ECOLI	Signal recognition particle protein OS=Escherichia coli (strain K12) OX=83333 GN=ffh PE=1 SV=1
3::P0AFA2|NARX_ECOLI	Nitrate/nitrite sensor protein NarX OS=Escherichia coli (strain K12) OX=83333 GN=narX PE=1 SV=1
3::P0ACL0|GLPR_ECOLI	Glycerol-3-phosphate regulon repressor OS=Escherichia coli (strain K12) OX=83333 GN=glpR PE=1 SV=1
3::P33136|OPGG_ECOLI	Glucans biosynthesis protein G OS=Escherichia coli (strain K12) OX=83333 GN=mdoG PE=1 SV=1
3::P0A7R9|RS11_ECOLI	30S ribosomal protein S11 OS=Escherichia coli (strain K12) OX=83333 GN=rpsK PE=1 SV=2
3::P25772|LIGB_ECOLI	DNA ligase B OS=Escherichia coli (strain K12) OX=83333 GN=ligB PE=1 SV=2
1::sp|UB2E1_HUMAN|	sp|UB2E1_HUMAN|
3::P75830|MACA_ECOLI	Macrolide export protein MacA OS=Escherichia coli (strain K12) OX=83333 GN=macA PE=1 SV=2
3::P17169|GLMS_ECOLI	Glutamine--fructose-6-phosphate aminotransferase [isomerizing] OS=Escherichia coli (strain K12) OX=83333 GN=glmS PE=1 SV=4
3::P0AAH4|SAPD_ECOLI	Putrescine export system ATP-binding protein SapD OS=Escherichia coli (strain K12) OX=83333 GN=sapD PE=1 SV=1
3::P76015|DHAK_ECOLI	PEP-dependent dihydroxyacetone kinase, dihydroxyacetone-binding subunit DhaK OS=Escherichia coli (strain K12) OX=83333 GN=dhaK PE
3::P77766|RNAAM_ECOLI	5'-3' exoribonuclease OS=Escherichia coli (strain K12) OX=83333 GN=yciV PE=1 SV=1
3::Q7DFV4|YMDE_ECOLI	Putative uncharacterized protein YmdE OS=Escherichia coli (strain K12) OX=83333 GN=ymdE PE=5 SV=3
3::P77739|KT3K_ECOLI	Probable ketoamine kinase YniA OS=Escherichia coli (strain K12) OX=83333 GN=yniA PE=3 SV=1
3::P16869|FHUE_ECOLI	FhuE receptor OS=Escherichia coli (strain K12) OX=83333 GN=fhuE PE=1 SV=2
3::P0A9M0|LON_ECOLI	Lon protease OS=Escherichia coli (strain K12) OX=83333 GN=lon PE=1 SV=1
3::P09831|GLTB_ECOLI	Glutamate synthase [NADPH] large chain OS=Escherichia coli (strain K12) OX=83333 GN=gltB PE=1 SV=3
3::P0A7V3|RS3_ECOLI	30S ribosomal protein S3 OS=Escherichia coli (strain K12) OX=83333 GN=rpsC PE=1 SV=2
3::P02918|PBPA_ECOLI	Penicillin-binding protein 1A OS=Escherichia coli (strain K12) OX=83333 GN=mrcA PE=1 SV=1
3::P37013|NORR_ECOLI	Anaerobic nitric oxide reductase transcription regulator NorR OS=Escherichia coli (strain K12) OX=83333 GN=norR PE=1 SV=2
3::P71229|HYFR_ECOLI	DNA-binding transcriptional activator HyfR OS=Escherichia coli (strain K12) OX=83333 GN=hyfR PE=1 SV=1
3::P38134|ETK_ECOLI	Tyrosine-protein kinase etk OS=Escherichia coli (strain K12) OX=83333 GN=etk PE=1 SV=2
3::P55140|YGCG_ECOLI	UPF0603 protein YgcG OS=Escherichia coli (strain K12) OX=83333 GN=ygcG PE=3 SV=2
3::P00582|DPO1_ECOLI	DNA polymerase I OS=Escherichia coli (strain K12) OX=83333 GN=polA PE=1 SV=1
3::P76352|YEEO_ECOLI	Probable FMN/FAD exporter YeeO OS=Escherichia coli (strain K12) OX=83333 GN=yeeO PE=1 SV=4
3::P0AGF6|TDCB_ECOLI	L-threonine dehydratase catabolic TdcB OS=Escherichia coli (strain K12) OX=83333 GN=tdcB PE=1 SV=1

	Target	Decoy	False discovery rate
Peptide matches above identity threshold	574	64	11.15 %
Peptide matches above homology or identity threshold	581	66	11.36 %

Select Summary Report

Format As [Select Summary (protein hits)] Help Help

Significance threshold p< 0.05 Max. number of hits AUTO Show Percolator scores

Standard scoring MudPIT scoring Display non-significant matches Show sub-sets 0

Show pop-ups Suppress pop-ups Require bold red

Preferred taxonomy [All entries]

Re-Search All queries Unassigned Below homology threshold Below identity threshold

1. 2::TOP2B_1-566 Mass: 66629 Score: 7506 Matches: 303(303) Sequences: 40(40) emPAI: 20.66

TOP2B_1-566

Query	Observed	Mr(expt)	Mr(calc)	ppm	Miss	Score	Expect	Rank	Unique	Peptide
397	401.7246	801.4346	801.4344	0.17	0	37	0.00029	1	U	K.AQTQLNK.K
473	410.7507	819.4867	819.4854	1.61	0	32	0.00067	1	U	K.IVGLQYK.K 464 471

516	414.7813	827.5480	827.5480	0.01	1	28	0.0016	1	U	K.LIEVVKK.K
623	426.2422	850.4698	850.4701	-0.35	0	29	0.0012	1	U	R.YGVFPLR.G 624 626 627
758	439.7584	877.5023	877.5022	0.22	0	34	0.00051	1	U	K.GIPVVEHK.V 759
975	459.7572	917.4999	917.5004	-0.59	0	49	2e-005	1	U	K.DIVALMTR.R
1054	465.7721	929.5297	929.5294	0.33	1	27	0.0025	1	U	K.AQTQLNKK.C 1053
1085	467.7551	933.4956	933.4953	0.24	0	(40)	0.00015	1	U	K.DIVALMTR.R 1082 1083 1084 1086
1149	473.2666	944.5187	944.5179	0.91	0	50	1.5e-005	1	U	K.LDETGVALK.V
1201	478.2316	954.4486	954.4481	0.54	0	26	0.0051	1	U	K.FTVETACK.E
1252	480.7449	959.4752	959.4746	0.64	0	(20)	0.016	1	U	K.ENMTLQPK.S 1250
1341	488.2222	974.4299	974.4305	-0.62	0	54	4.3e-006	1	U	K.LDDANDAGGK.H 1342 1343
1348	488.7413	975.4681	975.4695	-1.46	0	22	0.0088	1	U	K.ENMTLQPK.S 1352 1353
1393	491.7548	981.4951	981.4953	-0.26	0	39	0.00024	1	U	K.LCNIFSTK.F
1412	493.7636	985.5126	985.5120	0.54	0	43	5.7e-005	1	U	R.SYVDLYVK.D 1410 1411 1413
1585	505.2440	1008.4735	1008.4732	0.26	1	(20)	0.012	1	U	R.DKNMTCIK.V
1604	506.7296	1011.4446	1011.4444	0.20	0	43	8.1e-005	1	U	R.AYDLAGSCR.G
1704	513.2408	1024.4671	1024.4681	-1.01	1	30	0.002	1	U	R.DKNMTCIK.V
1714	514.2324	1026.4503	1026.4505	-0.23	0	46	2.7e-005	1	U	K.SYDDAESLK.T 1716 1717
1771	518.2516	1034.4887	1034.4880	0.73	1	38	0.00025	1	U	K.KEESETANK.N
2142	360.8647	1079.5724	1079.5723	0.03	0	(33)	0.00077	1	U	K.VIHELANER.W 2132 2134 2137 2140 2145 2147 2150 2152
2144	540.7935	1079.5725	1079.5723	0.19	0	57	3.5e-006	1	U	K.VIHELANER.W 2131 2135 2139 2143 2146 2148 2149
2492	374.8732	1121.5976	1121.5982	-0.49	1	(20)	0.017	1	U	R.DRYGVFPLR.G
2493	561.8066	1121.5986	1121.5982	0.35	1	28	0.0021	1	U	R.DRYGVFPLR.G
2561	376.8962	1127.6668	1127.6663	0.48	0	(36)	0.00027	1	U	K.SLAVSGLGVIGR.D
2563	564.8409	1127.6672	1127.6663	0.81	0	74	4.4e-008	1	U	K.SLAVSGLGVIGR.D 2549 2550 2551 2552 2554 2555 2556 2557 2558 2559 2560 2562 2564 2565 2566
2786	576.8188	1151.6230	1151.6227	0.32	0	28	0.0021	1	U	R.EVTFVPGLYK.I 2774 2777 2779 2783 2784
2814	578.2802	1154.5458	1154.5455	0.30	1	34	0.0008	1	U	K.KSYDDAESLK.T 2812
2853	387.2326	1158.6760	1158.6761	-0.11	0	(37)	0.00028	1	U	K.AGVSVKPFQVK.N 2858 2859
2857	580.3454	1158.6762	1158.6761	0.06	0	48	2e-005	1	U	K.AGVSVKPFQVK.N 2849 2856 2861 2862
3045	396.8872	1187.6396	1187.6398	-0.11	1	(30)	0.0022	1	U	K.DKLDETGVALK.V 3046 3052
3050	594.8272	1187.6398	1187.6398	0.04	1	50	2.2e-005	1	U	K.DKLDETGVALK.V 3047 3049 3051
3118	600.2537	1198.4929	1198.4933	-0.33	0	(28)	0.0018	1	U	K.QTWMNNMMK.T 3120
3121	600.2544	1198.4942	1198.4933	0.72	0	32	0.0009	1	U	K.QTWMNNMMK.T 3117 3119 3122
3223	608.2517	1214.4888	1214.4882	0.48	0	(31)	0.00074	1	U	K.QTWMNNMMK.T 3219 3221 3225
3224	608.2518	1214.4889	1214.4882	0.60	0	(27)	0.0019	1	U	K.QTWMNNMMK.T 3222
3226	608.2526	1214.4905	1214.4882	1.92	0	(30)	0.00091	1	U	K.QTWMNNMMK.T
3542	625.8076	1249.6007	1249.6013	-0.47	0	44	8.5e-005	1	U	R.WDVCLTLSEK.G
3760	637.8605	1273.7065	1273.7064	0.08	1	77	2.7e-008	1	U	K.LDKDIVALMTR.R 3750 3752 3755 3759 3761 3762 3764 3765
3766	425.5762	1273.7068	1273.7064	0.35	1	(33)	0.00078	1	U	K.LDKDIVALMTR.R 3753 3768
3890	430.9077	1289.7012	1289.7013	-0.12	1	(33)	0.001	1	U	K.LDKDIVALMTR.R 3881 3884 3886 3888 3895 3896
3894	645.8581	1289.7016	1289.7013	0.26	1	(62)	1.4e-006	1	U	K.LDKDIVALMTR.R 3882 3883 3885 3887 3889 3892 3893
4374	453.2417	1356.7032	1356.7038	-0.45	0	(44)	0.00011	1	U	R.HVDYVVDQVVGK.L 4380 4390
4388	679.3593	1356.7041	1356.7038	0.24	0	81	2.5e-008	1	U	R.HVDYVVDQVVGK.L 4375 4376 4377 4378 4381 4382 4383 4384 4385 4386 4387 4389 4391 4392 4394
5153	731.3817	1460.7488	1460.7511	-1.58	0	82	1.6e-008	1	U	K.IFDEILVNAADNK.Q 5157 5158 5163 5169 5170 5174 5176 5180 5181 5182 5183 5185 5187
5160	487.9241	1460.7505	1460.7511	-0.40	0	(49)	3.2e-005	1	U	K.IFDEILVNAADNK.Q 5151 5152 5155 5156 5159 5161 5162 5164 5165 5166 5167 5168 5171 5172 5173 5175
5875	765.4037	1528.7928	1528.7919	0.61	0	96	6e-010	1	U	K.QIMENAEINNIIK.I 5877
5876	510.6049	1528.7928	1528.7919	0.64	0	(32)	0.0017	1	U	K.QIMENAEINNIIK.I
6055	773.4005	1544.7865	1544.7868	-0.18	0	(75)	8e-008	1	U	K.QIMENAEINNIIK.I 6052 6053 6054 6056
6238	522.9128	1565.7167	1565.7169	-0.11	2	20	0.04	1	U	K.KEESETANKNDSSK.K 6239
6773	820.9358	1639.8571	1639.8570	0.10	0	81	1.6e-008	1	U	K.GFQQISFVNSIATTK.G 6758 6759 6761 6762 6763 6764 6765 6769 6770 6771 6772 6775 6776 6777 6778 67
6774	547.6264	1639.8573	1639.8570	0.17	0	(56)	5.1e-006	1	U	K.GFQQISFVNSIATTK.G 6756 6760 6766 6767 6782
7469	866.4067	1730.7989	1730.7968	1.23	0	(69)	4.4e-007	1	U	K.IMIMTDQDQDGSHIK.A 7466
7470	577.9414	1730.8024	1730.7968	3.25	0	(23)	0.02	1	U	K.IMIMTDQDQDGSHIK.A 7468
7565	583.2707	1746.7902	1746.7917	-0.84	0	(32)	0.0016	1	U	K.IMIMTDQDQDGSHIK.A 7580
7567	874.4030	1746.7915	1746.7917	-0.11	0	(68)	3.8e-007	1	U	K.IMIMTDQDQDGSHIK.A 7569 7576
7570	874.4031	1746.7916	1746.7917	-0.06	0	77	6.1e-008	1	U	K.IMIMTDQDQDGSHIK.A 7575
7578	583.2716	1746.7930	1746.7917	0.78	0	(48)	4.3e-005	1	U	K.IMIMTDQDQDGSHIK.A 7564 7568 7571 7572 7573 7574 7579
7688	588.6029	1762.7869	1762.7866	0.18	0	(62)	2.5e-006	1	U	K.IMIMTDQDQDGSHIK.A 7679 7680 7681 7682 7686 7687 7689 7691 7693 7694 7700
7697	882.4014	1762.7882	1762.7866	0.91	0	(72)	3e-007	1	U	K.IMIMTDQDQDGSHIK.A 7678 7684 7685 7692 7695 7696 7698 7699
7762	591.9606	1772.8600	1772.8615	-0.82	0	(24)	0.014	1	U	K.HSLECTLILTEGDSAK.S
7763	887.4382	1772.8618	1772.8615	0.22	0	93	1.8e-009	1	U	K.HSLECTLILTEGDSAK.S
8521	629.3266	1884.9580	1884.9581	-0.04	0	(59)	2.5e-006	1	U	K.VSIDPESNIISIWNNGK.G
8522	943.4864	1884.9583	1884.9581	0.11	0	77	4.3e-008	1	U	K.VSIDPESNIISIWNNGK.G
9649	724.9902	2171.9488	2171.9470	0.83	0	40	0.00043	1	U	K.HFDGEDYTCITFQPDLSK.F
10285	805.3811	2413.1216	2413.1260	-1.85	1	55	1.5e-005	1	U	K.IKHFDGEDYTCITFQPDLSK.F 10286
10546	1244.1186	2486.2226	2486.2217	0.36	0	86	1.3e-008	1	U	K.VYVPALIFGQLLTSSNYDDDEK.K 10538 10543 10548 10550 10551
10547	829.7482	2486.2227	2486.2217	0.40	0	(54)	2.1e-005	1	U	K.VYVPALIFGQLLTSSNYDDDEK.K 10541
10737	859.4251	2575.2534	2575.2529	0.19	0	39	0.00073	1	U	K.NHIWVFINCLIENPTFDSQTK.E
10815	872.4458	2614.3155	2614.3166	-0.43	1	(54)	1.9e-005	1	U	K.VYVPALIFGQLLTSSNYDDDEKK.V 10813 10817 10818 10824 10829 10830
10819	654.5865	2614.3169	2614.3166	0.10	1	(47)	9.7e-005	1	U	K.VYVPALIFGQLLTSSNYDDDEKK.V 10820
10827	1308.1665	2614.3185	2614.3166	0.72	1	64	2.1e-006	1	U	K.VYVPALIFGQLLTSSNYDDDEKK.V 10812

---

2. 1::sp|K1C10_HUMAN| Mass: 59711 Score: 1630 Matches: 47(47) Sequences: 26(26) emPAI: 7.52

sp|K1C10_HUMAN|

Query	Observed	Mr(expt)	Mr(calc)	ppm	Miss	Score	Expect	Rank	Unique	Peptide
419	404.2030	806.3915	806.3923	-0.98	0	42	8.9e-005	1		R.LAADDFR.L
428	405.2240	808.4334	808.4330	0.45	0	30	0.00091	1		R.LASYLDK.V
602	424.2297	846.4449	846.4447	0.30	0	25	0.0049	1	U	K.SEITELR.R
959	458.6957	915.3769	915.3756	1.39	0	25	0.0042	1		K.NHEEEMK.D
1464	497.2533	992.4920	992.4927	-0.71	0	45	4.9e-005	1	U	K.YENEVALR.Q
1476	498.2636	994.5126	994.5123	0.27	1	27	0.0057	1	U	K.IKEWYEK.H
1537	502.2799	1002.5453	1002.5458	-0.47	1	23	0.0079	1	U	K.SEITELRR.N
1751	516.3022	1030.5899	1030.5910	-1.10	0	55	3.4e-006	1		R.VLDELTLTK.A
2223	545.7691	1089.5236	1089.5237	-0.10	0	(46)	3.8e-005	1	U	K.VTMQNLNDR.L
2354	553.7668	1105.5191	1105.5186	0.44	0	59	2e-006	1	U	K.VTMQNLNDR.L 2352
3133	601.3123	1200.6100	1200.6098	0.13	0	62	1.5e-006	1		R.QSVEADINGLR.R

Query	Observed	Mr(expt)	Mr(calc)	ppm	Miss	Score	Expect	Rank	Unique	Peptide
3387	617.8431	1233.6717	1233.6717	-0.00	1	34	0.00052	1	U	R.LKYENEVALR.Q
3388	412.2313	1233.6720	1233.6717	0.24	1	(34)	0.00056	1	U	R.LKYENEVALR.Q
3634	631.8023	1261.5901	1261.5899	0.21	0	65	5.7e-007	1	U	R.SLLEGEGSSGGGGR.G
3950	434.2033	1299.5880	1299.5877	0.23	1	34	0.00094	1	U	K.NHEEEMKDLR.N
4452	683.3235	1364.6324	1364.6320	0.30	0	66	4.1e-007	1	U	R.SQYEQLAEQNR.K
4567	691.3281	1380.6417	1380.6408	0.64	0	81	2.3e-008	1	U	R.ALEESNYELEGK.I
4903	717.8887	1433.7628	1433.7626	0.15	1	60	2.1e-006	1	U	K.IRLENEIQTYR.S
5458	747.3708	1492.7269	1492.7270	-0.02	1	49	4e-005	1	U	R.SQYEQLAEQNRK.D
5459	498.5832	1492.7278	1492.7270	0.53	1	(31)	0.0023	1	U	R.SQYEQLAEQNRK.D
7283	854.3888	1706.7630	1706.7649	-1.10	0	110	2.2e-011	1	U	K.GSLGGGFSSGGFSGGSFSR.G
7969	599.6753	1796.0040	1796.0043	-0.15	0	(90)	1.1e-009	1	U	R.NVQALEIELQSQLALK.Q
7971	899.0096	1796.0047	1796.0043	0.19	0	117	1.8e-012	1	U	R.NVQALEIELQSQLALK.Q 7970
9028	666.3284	1995.9634	1995.9636	-0.12	0	(41)	0.00034	1	U	K.ELTTEIDNNIEQISSYK.S
9029	998.9900	1995.9653	1995.9636	0.86	0	81	3.4e-008	1	U	K.ELTTEIDNNIEQISSYK.S
9370	1048.5271	2095.0397	2095.0394	0.12	0	(75)	1.6e-007	1	U	K.ADLEMQIESLTEELAYLK.K 9369 9372
9371	699.3545	2095.0416	2095.0394	1.06	0	(49)	5.1e-005	1	U	K.ADLEMQIESLTEELAYLK.K
9458	704.6854	2111.0344	2111.0343	0.03	0	(49)	4.4e-005	1	U	K.ADLEMQIESLTEELAYLK.K
9459	1056.5249	2111.0353	2111.0343	0.45	0	77	6.9e-008	1	U	K.ADLEMQIESLTEELAYLK.K
9788	738.0386	2211.0940	2211.0906	1.53	1	44	0.0002	1	U	K.SKELTTEIDNNIEQISSYK.S 9787
9830	742.0526	2223.1358	2223.1344	0.65	1	21	0.027	1	U	K.ADLEMQIESLTEELAYLKK.N
10190	1184.1328	2366.2509	2366.2553	-1.86	0	61	1.9e-006	1	U	K.NQILNLTTDNANILLQIDNAR.L 10191
10192	789.7587	2366.2542	2366.2553	-0.49	0	(60)	2.3e-006	1	U	K.NQILNLTTDNANILLQIDNAR.L 10193
11262	958.1358	2871.3856	2871.3855	0.05	0	(45)	0.00023	1	U	R.NVSTGDVNVEMNAAPGVDLTQLLNNMR.S
11283	963.4665	2887.3776	2887.3804	-0.99	0	(39)	0.00083	1	U	R.NVSTGDVNVEMNAAPGVDLTQLLNNMR.S 11282
11285	963.4687	2887.3843	2887.3804	1.34	0	65	2.2e-006	1	U	R.NVSTGDVNVEMNAAPGVDLTQLLNNMR.S
11322	968.7992	2903.3757	2903.3753	0.12	0	(48)	9.2e-005	1	U	R.NVSTGDVNVEMNAAPGVDLTQLLNNMR.S
11763	1018.2139	3051.6200	3051.6200	-0.00	1	60	2.4e-006	1	U	K.TIDDLKNQILNLTTDNANILLQIDNAR.L 11762

---

3. 1::sp|K2C1_HUMAN| Mass: 66018 Score: 1517 Matches: 45(45) Sequences: 29(29) emPAI: 7.43

sp|K2C1_HUMAN|

Query	Observed	Mr(expt)	Mr(calc)	ppm	Miss	Score	Expect	Rank	Unique	Peptide
406	402.7085	803.4024	803.4025	-0.12	0	28	0.0015	1	U	R.SEIDNVK.K
742	437.7535	873.4924	873.4920	0.47	0	45	4.4e-005	1	U	R.SLVNLGGSK.S
1069	466.7559	931.4973	931.4974	-0.16	1	37	0.00045	1	U	R.SEIDNVKK.Q
1329	487.2696	972.5246	972.5240	0.67	0	41	8.9e-005	1		K.IEISELNR.V
1506	500.2263	998.4381	998.4379	0.23	0	45	5e-005	1	U	K.DVDGAYMTK.V
1624	508.2235	1014.4325	1014.4328	-0.30	0	(41)	7.3e-005	1	U	K.DVDGAYMTK.V
1760	517.2620	1032.5094	1032.5087	0.66	0	35	0.00042	1	U	R.TLLEGEESR.M
2015	533.7621	1065.5097	1065.5090	0.62	1	29	0.0019	1		K.YEDEINKR.T
2016	356.1772	1065.5099	1065.5090	0.76	1	(23)	0.0082	1		K.YEDEINKR.T
2076	537.3011	1072.5877	1072.5876	0.08	1	35	0.00093	1	U	R.LRSEIDNVK.K
2250	546.7552	1091.4959	1091.4956	0.27	0	59	1.4e-006	1	U	R.GSGGGSSGGSIGGR.G
2523	563.2746	1124.5346	1124.5349	-0.25	0	64	7.4e-007	1	U	K.AEAESLYQSK.Y
2538	564.2737	1126.5329	1126.5329	0.01	1	(31)	0.0016	1	U	K.KDVDGAYMTK.V
2692	572.2676	1142.5207	1142.5278	-6.16	1	40	0.00014	1	U	K.KDVDGAYMTK.V
2769	576.7801	1151.5457	1151.5458	-0.12	1	27	0.0046	1	U	R.NKYEDEINK.R
2825	579.2612	1156.5079	1156.5070	0.79	0	42	0.00012	1	U	R.DYQELMNTK.L
2986	590.3039	1178.5932	1178.5931	0.11	0	67	4.8e-007	1	U	K.YEELQITAGR.H 2985
3136	401.2345	1200.6816	1200.6826	-0.85	2	23	0.0062	1	U	R.LRSEIDNVKK.Q
3137	601.3488	1200.6831	1200.6826	0.44	2	(17)	0.028	1	U	R.LRSEIDNVKK.Q
3791	639.3587	1276.7029	1276.7027	0.17	0	70	1.7e-007	1	U	K.LALDLEIATYR.T
3947	650.7680	1299.5215	1299.5224	-0.64	0	(58)	2.4e-006	1	U	K.NMQDMVEDYR.N
3986	651.8612	1301.7079	1301.7078	0.08	0	65	6.4e-007	1	U	R.SLDLDSIIAEVK.A
4028	436.8896	1307.6470	1307.6469	0.08	2	37	0.00052	1	U	R.NKYEDEINKR.T
4029	654.8309	1307.6472	1307.6469	0.23	2	(33)	0.0013	1	U	R.NKYEDEINKR.T
4087	658.7659	1315.5172	1315.5173	-0.03	0	(46)	2.3e-005	1	U	K.NMQDMVEDYR.N
4088	658.7664	1315.5183	1315.5173	0.77	0	61	7.9e-007	1	U	K.NMQDMVEDYR.N
4230	666.7634	1331.5123	1331.5122	0.09	0	(48)	1.5e-005	1	U	K.NMQDMVEDYR.N 4231 4232 4233
4283	670.8385	1339.6625	1339.6619	0.46	1	60	2.1e-006	1	U	K.SKAEAESLYQSK.Y
4373	679.3517	1356.6888	1356.6885	0.27	0	72	1.9e-007	1	U	K.LNDLEDALQQAK.E
4587	692.3493	1382.6841	1382.6830	0.76	0	64	7.2e-007	1	U	K.SLNNQFASFIDK.V
5311	738.3940	1474.7734	1474.7780	-3.10	0	83	1.1e-008	1		R.FLEQQNQVLQTK.W
6898	829.4002	1656.7859	1656.7856	0.16	0	74	1.6e-007	1	U	R.SGGGFSSGSAGIINYQR.R 6899
6900	553.2700	1656.7882	1656.7856	1.55	0	(20)	0.034	1	U	R.SGGGFSSGSAGIINYQR.R
7358	858.9290	1715.8435	1715.8438	-0.15	0	81	2.7e-008	1	U	K.QISNLQQSISDAEQR.G
7359	572.9553	1715.8440	1715.8438	0.08	0	(41)	0.00031	1	U	K.QISNLQQSISDAEQR.G
8306	615.6538	1843.9396	1843.9388	0.47	1	18	0.042	1	U	K.KQISNLQQSISDAEQR.G
9022	665.3300	1992.9683	1992.9693	-0.53	0	45	0.00013	1	U	R.THNLEPYFESFINNLR.R
10213	1192.4786	2382.9426	2382.9447	-0.87	0	103	9e-011	1	U	R.GGGGGGYGSGGSSYGSGGGSYGSGGGGGGGR.G
10214	795.3222	2382.9447	2382.9447	0.02	0	(76)	4.6e-008	1	U	R.GGGGGGYGSGGSSYGSGGGSYGSGGGGGGGR.G
12365	1104.7747	3311.3023	3311.3009	0.42	0	95	9.9e-010	1	U	R.GSYGSGGSSYGSGGGSYGSGGGGGGHGSYGSGSSSGGYR.G

---

4. 1::sp|K22E_HUMAN| Mass: 66110 Score: 1232 Matches: 35(35) Sequences: 28(28) emPAI: 5.92

sp|K22E_HUMAN|

Query	Observed	Mr(expt)	Mr(calc)	ppm	Miss	Score	Expect	Rank	Unique	Peptide
514	414.2188	826.4230	826.4225	0.61	0	33	0.00055	1		R.FASFIDK.V
523	416.2506	830.4867	830.4862	0.62	0	30	0.00097	1	U	R.SLVGLGGTK.S
1329	487.2696	972.5246	972.5240	0.67	0	41	8.9e-005	1		K.IEISELNR.V
1471	497.7876	993.5607	993.5607	-0.03	0	49	1.4e-005	1	U	R.LQGEIAHVK.K
1776	519.2675	1036.5204	1036.5189	1.41	0	54	8.1e-006	1	U	R.YLDGLTAER.T
1780	519.7591	1037.5037	1037.5029	0.78	1	35	0.00045	1	U	K.KYEDEINK.R
1798	521.2823	1040.5500	1040.5502	-0.22	0	46	2.4e-005	1	U	K.VDPEIQNVK.A
2015	533.7621	1065.5097	1065.5090	0.62	1	29	0.0019	1		K.YEDEINKR.T
2016	356.1772	1065.5099	1065.5090	0.76	1	(23)	0.0082	1		K.YEDEINKR.T
2180	542.7526	1083.4907	1083.4906	0.04	0	38	0.00022	1	U	K.DVDNAYMIK.V

2359	554.2752	1106.5358	1106.5356	0.21	0	33	0.00056	1	U	K.AQYEEIAQR.S	
3077	597.3118	1192.6091	1192.6088	0.28	0	62	1.8e-006	1	U	K.YEELQVTVGR.H	
3081	398.8752	1193.6039	1193.6040	-0.12	2	52	1e-005	1	U	K.KYEDEINKR.T	
3105	599.2782	1196.5419	1196.5422	-0.25	0	50	2.1e-005	1	U	K.GGSISGGGYGSGGGK.H	
3179	604.8117	1207.6089	1207.6085	0.37	0	69	1.9e-007	1	U	R.TAAENDFVTLK.K	
3574	627.8074	1253.6002	1253.6001	0.12	0	87	4.9e-009	1	U	R.GFSSGSAVVSGGSR.R	
3637	632.3506	1262.6865	1262.6870	-0.38	0	70	2.4e-007	1	U	K.LALDVEIATYR.K	
4119	660.7949	1319.5752	1319.5756	-0.30	0	66	5e-007	1	U	R.HGGGGGGFGGGGFGSR.S	
4120	440.8659	1319.5760	1319.5756	0.29	0	(37)	0.00038	1	U	R.HGGGGGGFGGGGFGSR.S	
4195	665.3235	1328.6325	1328.6320	0.33	0	75	5.6e-008	1	U	K.NVQDAIADAEQR.G	
4196	665.3667	1328.7188	1328.7187	0.05	0	68	4.6e-007	1	U	R.NLDLDSIIAEVK.A	
4261	668.8601	1335.7057	1335.7034	1.70	1	61	1.5e-006	1	U	R.TAAENDFVTLKK.D	
4489	686.3592	1370.7037	1370.7041	-0.27	0	77	4.2e-008	1	U	K.LNDLEEALQQAK.E	
4631	696.3438	1390.6730	1390.6728	0.13	1	65	5.6e-007	1	U	R.SKEEAEALYHSK.Y	
4632	464.5649	1390.6730	1390.6728	0.13	1	(34)	0.00074	1	U	R.SKEEAEALYHSK.Y	
5150	730.9034	1459.7922	1459.7922	-0.01	0	76	4.4e-008	1	U	K.VDLLNQEIEFLK.V	
5311	738.3940	1474.7734	1474.7780	-3.10	0	83	1.1e-008	1		R.FLEQQNQVLQTK.W	
8274	919.9613	1837.9080	1837.9072	0.48	0	53	1.8e-005	1	U	K.SISISVAGGGGGFGAAGGFGGR.G	
9947	752.6867	2255.0382	2255.0376	0.29	1	(32)	0.0029	1	U	R.TSQNSELNNMQDLVEDYKK.K	
9995	758.0187	2271.0343	2271.0325	0.79	1	43	0.00021	1	U	R.TSQNSELNNMQDLVEDYKK.K	
11747	1016.8360	3047.4860	3047.4870	-0.31	0	56	1.7e-005	1	U	K.VLYDAEISQIHQSVTDTNVILSMDNSR.N	11748
11778	1022.1687	3063.4842	3063.4819	0.74	0	(47)	0.00012	1	U	K.VLYDAEISQIHQSVTDTNVILSMDNSR.N	
13033	1023.7731	4091.0633	4091.0654	-0.53	0	29	0.0075	1	U	R.FGGFGGPGGVGGLGGPGGFGPGGYPGGIHEVSVNQSLLQPLNVK.V	13034

5. 1::sp|K1C9_HUMAN| Mass: 62320 Score: 1205 Matches: 35(35) Sequences: 20(20) emPAI: 3.83

sp|K1C9_HUMAN|

Query	Observed	Mr(expt)	Mr(calc)	ppm	Miss	Score	Expect	Rank	Unique	Peptide	
428	405.2240	808.4334	808.4330	0.45	0	30	0.00091	1		R.LASYLDK.V	
432	406.7533	811.4920	811.4916	0.52	1	57	1.8e-006	1	U	K.KGPAAIQK.N	
841	449.2103	896.4061	896.4062	-0.08	0	(19)	0.026	1	U	R.MTLDDFR.I	
944	457.2079	912.4012	912.4011	0.12	0	26	0.0029	1	U	R.MTLDDFR.I	
1967	530.7857	1059.5568	1059.5560	0.73	0	42	0.00011	1	U	K.TLLDIDNTR.M	
2002	533.2535	1064.4924	1064.4920	0.37	0	53	5.9e-006	1	U	K.STMQELNSR.L	
2157	541.2513	1080.4881	1080.4870	1.05	0	(20)	0.017	1	U	K.STMQELNSR.L	
2276	365.8537	1094.5394	1094.5396	-0.20	1	22	0.014	1	U	K.IQDWYDKK.G	
2277	548.2772	1094.5398	1094.5396	0.19	1	(17)	0.043	1	U	K.IQDWYDKK.G	
2485	561.2961	1120.5777	1120.5764	1.17	0	35	0.00063	1	U	R.QEYEQLIAK.N	
3176	603.8054	1205.5962	1205.5962	-0.01	0	41	0.00017	1	U	R.QVLDNLTMEK.S	
3374	616.8026	1231.5907	1231.5906	0.12	0	77	4.4e-008	1	U	R.SGGGGGGGLGSGGSIR.S	
3395	618.2678	1234.5210	1234.5215	-0.36	0	63	9.9e-007	1	U	R.FSSSSGYGGGSSR.V	
4018	436.5639	1306.6697	1306.6703	-0.46	1	(26)	0.0065	1	U	R.IKFEMEQNLR.Q	
4148	662.3401	1322.6656	1322.6652	0.27	1	41	0.00022	1	U	R.IKFEMEQNLR.Q	
4149	441.8960	1322.6661	1322.6652	0.64	1	(32)	0.002	1	U	R.IKFEMEQNLR.Q	4150
6440	529.5936	1585.7589	1585.7583	0.33	0	(35)	0.00099	1	U	K.VQALEEANNDLENK.I	
6441	793.8869	1585.7592	1585.7583	0.57	0	80	2.9e-008	1	U	K.VQALEEANNDLENK.I	
7877	896.3674	1790.7203	1790.7205	-0.08	0	97	3.7e-010	1	U	R.GGSGGSYGGGGSGGGYGGGSGSR.G	7875
8272	613.3267	1836.9582	1836.9581	0.07	0	39	0.00024	1	U	R.HGVQELEIELQSQLSK.K	8273
9752	1102.0074	2202.0002	2202.0072	-3.14	0	99	5.8e-010	1	U	K.SDLEMQYETLQEELMALK.K	9753
9754	735.0093	2202.0060	2202.0072	-0.51	0	(28)	0.0068	1	U	K.SDLEMQYETLQEELMALK.K	9755
10589	837.3814	2509.1223	2509.1245	-0.88	0	74	2.2e-007	1	U	K.EIETYHNLLEGGQEDFESSGAGK.I	10590
11296	965.8013	2894.3820	2894.3828	-0.30	1	55	1.8e-005	1	U	K.NHKEEMSQLTGQNSGDVNVEINVAPGK.D	
11315	726.3582	2901.4039	2901.4032	0.22	1	(22)	0.049	1	U	K.NYSPYYNTIDDLKDQIVDLTVGNNK.T	
11316	968.1420	2901.4042	2901.4032	0.35	1	40	0.00075	1	U	K.NYSPYYNTIDDLKDQIVDLTVGNNK.T	
12188	1075.0977	3222.2712	3222.2744	-0.99	0	89	4.3e-009	1	U	R.GGSGGSHGGGSGFGGESGGSYGGGEEASGSGGGYGGGSGK.S	12189
12291	1088.8422	3263.5047	3263.5066	-0.58	0	92	5.2e-009	1	U	K.DIENQYETQITQIEHEVSSSGQEVQSSAK.E	

6. 1::sp|HBB_HUMAN| Mass: 15971 Score: 317 Matches: 10(10) Sequences: 7(7) emPAI: 7.10

sp|HBB_HUMAN|

Query	Observed	Mr(expt)	Mr(calc)	ppm	Miss	Score	Expect	Rank	Unique	Peptide
1070	466.7639	931.5132	931.5127	0.49	0	31	0.00097	1	U	K.SAVTALWGK.V
1187	476.7583	951.5021	951.5025	-0.45	0	22	0.0085	1	U	-.VHLTPEEK.S
2532	563.7859	1125.5572	1125.5567	0.47	0	29	0.0025	1	U	K.LHVDPENFR.L
2534	376.1931	1125.5576	1125.5567	0.76	0	(21)	0.016	1	U	K.LHVDPENFR.L
2745	383.8961	1148.6664	1148.6666	-0.15	0	(35)	0.00029	1	U	K.VVAGVANALAHK.Y
2746	575.3408	1148.6671	1148.6666	0.48	0	72	6.3e-008	1	U	K.VVAGVANALAHK.Y
3769	637.8674	1273.7202	1273.7183	1.52	0	40	0.00011	1	U	R.LLVVYPWTQR.F
4552	689.8543	1377.6941	1377.6929	0.91	0	25	0.0065	1	U	K.EFTPPVQAAYQK.V
9218	1029.9782	2057.9418	2057.9405	0.65	0	95	1.6e-009	1	U	R.FFESFGDLSTPDAVMGNPK.V
9282	1037.9748	2073.9350	2073.9354	-0.20	0	(82)	3e-008	1	U	R.FFESFGDLSTPDAVMGNPK.V

7. 1::sp|K1C15_SHEEP| Mass: 48740 Score: 203 Matches: 6(6) Sequences: 6(6) emPAI: 0.69

sp|K1C15_SHEEP|

Query	Observed	Mr(expt)	Mr(calc)	ppm	Miss	Score	Expect	Rank	Unique	Peptide
419	404.2030	806.3915	806.3923	-0.98	0	42	8.9e-005	1		R.LAADDFR.L
959	458.6957	915.3769	915.3756	1.39	0	25	0.0042	1		K.NHEEEMK.E
1436	495.2725	988.5305	988.5301	0.34	1	23	0.0093	1	U	K.SEITDLRR.T
1751	516.3022	1030.5899	1030.5910	-1.10	0	55	3.4e-006	1		R.VLDELTLTK.T
3133	601.3123	1200.6100	1200.6098	0.13	0	62	1.5e-006	1		R.QSVEADINGLR.R
3974	651.3328	1300.6511	1300.6510	0.09	0	68	3.3e-007	1	U	R.ALEEANADLEVK.I

8. 3::P0AA25|THIO_ECOLI Mass: 11913 Score: 181 Matches: 3(3) Sequences: 2(2) emPAI: 1.01

Thioredoxin 1 OS=Escherichia coli (strain K12) OX=83333 GN=trxA PE=1 SV=2

Query	Observed	Mr(expt)	Mr(calc)	ppm	Miss	Score	Expect	Rank	Unique	Peptide	
3688	634.3356	1266.6566	1266.6568	-0.15	0	82	1.3e-008	1	U	K.LNIDQNPGTAPK.Y	
8057	903.4532	1804.8919	1804.8917	0.14	0	70	3.1e-007	1	U	K.MIAPILDEIADEYQGK.L	8056

9. 1::sp|HBA_HUMAN| Mass: 15174 Score: 172 Matches: 7(7) Sequences: 5(5) emPAI: 4.19

sp|HBA_HUMAN|

Query	Observed	Mr(expt)	Mr(calc)	ppm	Miss	Score	Expect	Rank	Unique	Peptide
107	365.2106	728.4067	728.4068	-0.16	0	24	0.0037	1	U	-.VLSPADK.T
2067	536.2806	1070.5466	1070.5471	-0.42	0	27	0.0026	1	U	R.MFLSFPTTK.T
2206	544.2784	1086.5422	1086.5420	0.18	0	(16)	0.031	1	U	R.MFLSFPTTK.T
3564	626.8609	1251.7073	1251.7075	-0.12	0	57	2.3e-006	1	U	K.FLASVSTVLTSK.Y
5872	765.3710	1528.7274	1528.7270	0.29	0	61	2.5e-006	1	U	K.VGAHAGEYGAEALER.M
5873	510.5832	1528.7277	1528.7270	0.45	0	(54)	1.1e-005	1	U	K.VGAHAGEYGAEALER.M
11687	603.3031	3011.4789	3011.4771	0.62	0	25	0.018	1	U	K.VADALTNAVAHVDDMPNALSALSDLHAHK.L

10. 1::sp|TRYP_PIG| Mass: 25078 Score: 169 Matches: 7(7) Sequences: 2(2) emPAI: 0.40

sp|TRYP_PIG|

Query	Observed	Mr(expt)	Mr(calc)	ppm	Miss	Score	Expect	Rank	Unique	Peptide
585	421.7587	841.5029	841.5022	0.92	0	49	1.3e-005	1	U	R.VATVSLPR.S 582 583 584 586 587
1838	523.2856	1044.5567	1044.5564	0.30	0	40	0.00019	1	U	K.LSSPATLNSR.V

11. 1::sp|ALBU_BOVIN| Mass: 71244 Score: 91 Matches: 6(6) Sequences: 4(4) emPAI: 0.27

sp|ALBU_BOVIN|

Query	Observed	Mr(expt)	Mr(calc)	ppm	Miss	Score	Expect	Rank	Unique	Peptide
357	395.2398	788.4649	788.4644	0.73	0	16	0.024	1	U	K.LVTDLTK.V
454	409.7165	817.4184	817.4181	0.30	0	24	0.0041	1	U	K.ATEEQLK.T
991	461.7480	921.4815	921.4807	0.87	0	28	0.0021	1	U	K.AEFVEVTK.L 990
6751	547.3176	1638.9311	1638.9305	0.35	1	33	0.00049	1	U	R.KVPQVSTPTLVEVSR.S 6750

12. 3::P76298|FLHA_ECOLI Mass: 74795 Score: 61 Matches: 8(8) Sequences: 1(1) emPAI: 0.06

Flagellar biosynthesis protein FlhA OS=Escherichia coli (strain K12) OX=83333 GN=flhA PE=3 SV=1

Query	Observed	Mr(expt)	Mr(calc)	ppm	Miss	Score	Expect	Rank	Unique	Peptide
3650	632.8196	1263.6245	1263.6159	6.86	1	(17)	0.038	1	U	R.HIRMTATIGGK.- 3645
3666	632.8199	1263.6253	1263.6159	7.43	1	25	0.0052	1	U	R.HIRMTATIGGK.- 3653 3658 3660 3662 3668

13. 1::sp|KRHB4_HUMAN| Mass: 65938 Score: 44 Matches: 2(2) Sequences: 2(2) emPAI: 0.14

sp|KRHB4_HUMAN|

Query	Observed	Mr(expt)	Mr(calc)	ppm	Miss	Score	Expect	Rank	Unique	Peptide
514	414.2188	826.4230	826.4225	0.61	0	33	0.00055	1		K.FASFIDK.V
3637	632.3506	1262.6865	1262.6870	-0.38	0	26	0.0059	2	U	K.LGLDIEIATYR.R

14. 1::sp|CASB_BOVIN| Mass: 25148 Score: 34 Matches: 2(2) Sequences: 1(1) emPAI: 0.18

sp|CASB_BOVIN|

Query	Observed	Mr(expt)	Mr(calc)	ppm	Miss	Score	Expect	Rank	Unique	Peptide
9749	734.7257	2201.1552	2201.1555	-0.11	0	26	0.0079	1	U	R.DMPIQAFLLYQEPVLGPVR.G 9750

15. 1::sp|RS27A_HUMAN| Mass: 18296 Score: 33 Matches: 1(1) Sequences: 1(1) emPAI: 0.26

sp|RS27A_HUMAN|

Query	Observed	Mr(expt)	Mr(calc)	ppm	Miss	Score	Expect	Rank	Unique	Peptide
7847	894.4674	1786.9203	1786.9200	0.18	0	33	0.0017	1	U	K.TITLEVEPSDTIENVK.A

16. 3::P0AD96|LIVJ_ECOLI Mass: 39223 Score: 31 Matches: 1(1) Sequences: 1(1) emPAI: 0.11

Leu/Ile/Val-binding protein OS=Escherichia coli (strain K12) OX=83333 GN=livJ PE=1 SV=1

Query	Observed	Mr(expt)	Mr(calc)	ppm	Miss	Score	Expect	Rank	Unique	Peptide
389	400.7350	799.4555	799.4552	0.38	0	31	0.00082	1	U	K.QAVAVANK.V

Proteins matching the same set of peptides:

3::P04816|LIVK_ECOLI Mass: 39468 Score: 31 Matches: 1(1) Sequences: 1(1)

Leucine-specific-binding protein OS=Escherichia coli (strain K12) OX=83333 GN=livK PE=1 SV=2

17. 3::P77211|CUSC_ECOLI Mass: 50410 Score: 29 Matches: 2(2) Sequences: 1(1) emPAI: 0.09

Cation efflux system protein CusC OS=Escherichia coli (strain K12) OX=83333 GN=cusC PE=1 SV=1

Query	Observed	Mr(expt)	Mr(calc)	ppm	Miss	Score	Expect	Rank	Unique	Peptide
10192	789.7587	2366.2542	2366.2706	-6.93	1	25	0.0067	2	U	K.IEIPIFNAGRNQANLDIAEIR.Q 10193

18. 3::P75882|GFCD_ECOLI Mass: 78923 Score: 28 Matches: 2(2) Sequences: 1(1) emPAI: 0.06

Uncharacterized lipoprotein GfcD OS=Escherichia coli (strain K12) OX=83333 GN=gfcD PE=3 SV=1

Query	Observed	Mr(expt)	Mr(calc)	ppm	Miss	Score	Expect	Rank	Unique	Peptide
1654	509.2822	1016.5498	1016.5502	-0.42	1	21	0.012	1	U	K.DKGATIDVAK.R 1657

19. 3::Q06065|ATOC_ECOLI Mass: 52428 Score: 25 Matches: 2(2) Sequences: 1(1) emPAI: 0.08

Regulatory protein AtoC OS=Escherichia coli (strain K12) OX=83333 GN=atoC PE=1 SV=2

Query	Observed	Mr(expt)	Mr(calc)	ppm	Miss	Score	Expect	Rank	Unique	Peptide
3665	422.2156	1263.6251	1263.6203	3.84	1	22	0.011	1	U	R.MPEMDGIKALK.E 3640

20. 3::P62601|TREF_ECOLI Mass: 63828 Score: 24 Matches: 1(1) Sequences: 1(1) emPAI: 0.07

Cytoplasmic trehalase OS=Escherichia coli (strain K12) OX=83333 GN=treF PE=1 SV=1

Query	Observed	Mr(expt)	Mr(calc)	ppm	Miss	Score	Expect	Rank	Unique	Peptide
6667	812.4225	1622.8304	1622.8280	1.49	1	24	0.0088	1	U	K.LESAIANISALKGEK.E

21. 3::A0A7H2C767|A0A7H2C767_ECOLI Mass: 79935 Score: 24 Matches: 1(1) Sequences: 1(1) emPAI: 0.05

Chaperone protein ClpB OS=Escherichia coli (strain K12) OX=83333 GN=clpB PE=3 SV=1

Query	Observed	Mr(expt)	Mr(calc)	ppm	Miss	Score	Expect	Rank	Unique	Peptide
349	394.7112	787.4078	787.4075	0.28	0	24	0.0038	1	U	K.AELEQAK.I

Proteins matching the same set of peptides:

3::P63284|CLPB_ECOLI Mass: 95697 Score: 24 Matches: 1(1) Sequences: 1(1)

Chaperone protein ClpB OS=Escherichia coli (strain K12) OX=83333 GN=clpB PE=1 SV=1

22. 3::P32719|ALSE_ECOLI Mass: 26263 Score: 24 Matches: 1(1) Sequences: 1(1) emPAI: 0.17

D-allulose-6-phosphate 3-epimerase OS=Escherichia coli (strain K12) OX=83333 GN=alsE PE=1 SV=1

Query	Observed	Mr(expt)	Mr(calc)	ppm	Miss	Score	Expect	Rank	Unique	Peptide
6102	776.3944	1550.7743	1550.7870	-8.22	1	24	0.011	1	U	M.KISPSLMCMDLLK.F

---

23. 3::P76092|YNBC_ECOLI Mass: 65529 Score: 23 Matches: 1(1) Sequences: 1(1) emPAI: 0.07

Uncharacterized protein YnbC OS=Escherichia coli (strain K12) OX=83333 GN=ynbC PE=1 SV=1

Query	Observed	Mr(expt)	Mr(calc)	ppm	Miss	Score	Expect	Rank	Unique	Peptide
1025	464.2502	926.4859	926.4830	3.20	2	23	0.0053	1	U	R.FMRKAMK.L

---

24. 3::Q46857|DKGA_ECOLI Mass: 31147 Score: 23 Matches: 1(1) Sequences: 1(1) emPAI: 0.15

2,5-diketo-D-gluconic acid reductase A OS=Escherichia coli (strain K12) OX=83333 GN=dkgA PE=1 SV=3

Query	Observed	Mr(expt)	Mr(calc)	ppm	Miss	Score	Expect	Rank	Unique	Peptide
1058	466.2459	930.4771	930.4844	-7.83	0	23	0.0056	1	U	-.MANPTVIK.L

---

25. 3::P0ABH0|FTSA_ECOLI Mass: 45815 Score: 22 Matches: 1(1) Sequences: 1(1) emPAI: 0.10

Cell division protein FtsA OS=Escherichia coli (strain K12) OX=83333 GN=ftsA PE=1 SV=1

Query	Observed	Mr(expt)	Mr(calc)	ppm	Miss	Score	Expect	Rank	Unique	Peptide
6076	774.3977	1546.7808	1546.7661	9.51	1	22	0.014	1	U	R.GMDKGGVNDLESVVK.C

---

26. 3::P12996|BIOB_ECOLI Mass: 39079 Score: 22 Matches: 1(1) Sequences: 1(1) emPAI: 0.11

Biotin synthase OS=Escherichia coli (strain K12) OX=83333 GN=bioB PE=1 SV=2

Query	Observed	Mr(expt)	Mr(calc)	ppm	Miss	Score	Expect	Rank	Unique	Peptide
221	379.7297	757.4448	757.4446	0.21	1	22	0.0074	1	U	K.VRDAGIK.V

---

27. 3::P39358|YJHG_ECOLI Mass: 70486 Score: 22 Matches: 1(1) Sequences: 1(1) emPAI: 0.06

D-xylonate dehydratase YjhG OS=Escherichia coli (strain K12) OX=83333 GN=yjhG PE=1 SV=2

Query	Observed	Mr(expt)	Mr(calc)	ppm	Miss	Score	Expect	Rank	Unique	Peptide
155	372.7219	743.4292	743.4290	0.30	0	22	0.0063	1	U	K.VQTIGAR.F

Proteins matching the same set of peptides:
3::P77596|YAGF_ECOLI Mass: 69926 Score: 22 Matches: 1(1) Sequences: 1(1)
D-xylonate dehydratase YagF OS=Escherichia coli (strain K12) OX=83333 GN=yagF PE=1 SV=1

---

28. 3::P21361|YCIG_ECOLI Mass: 5999 Score: 21 Matches: 1(1) Sequences: 1(1) emPAI: 0.95

Uncharacterized protein YciG OS=Escherichia coli (strain K12) OX=83333 GN=yciG PE=3 SV=1

Query	Observed	Mr(expt)	Mr(calc)	ppm	Miss	Score	Expect	Rank	Unique	Peptide
1251	480.7448	959.4751	959.4785	-3.53	1	21	0.013	1	U	K.KGGQQSGGNK.S

---

29. 3::P12008|AROC_ECOLI Mass: 39341 Score: 20 Matches: 1(1) Sequences: 1(1) emPAI: 0.11

Chorismate synthase OS=Escherichia coli (strain K12) OX=83333 GN=aroC PE=1 SV=4

Query	Observed	Mr(expt)	Mr(calc)	ppm	Miss	Score	Expect	Rank	Unique	Peptide
726	436.2313	870.4480	870.4559	-9.06	1	20	0.0094	1	U	R.REPDQVK.I

---

30. 3::P71242|WCAK_ECOLI Mass: 47598 Score: 20 Matches: 1(1) Sequences: 1(1) emPAI: 0.09

Colanic acid biosynthesis protein WcaK OS=Escherichia coli (strain K12) OX=83333 GN=wcaK PE=3 SV=2

Query	Observed	Mr(expt)	Mr(calc)	ppm	Miss	Score	Expect	Rank	Unique	Peptide
813	445.7563	889.4980	889.4981	-0.07	1	20	0.02	1	U	K.RSNITTAK.V

---

31. 3::P46891|COF_ECOLI Mass: 30750 Score: 20 Matches: 1(1) Sequences: 1(1) emPAI: 0.15

HMP-PP phosphatase OS=Escherichia coli (strain K12) OX=83333 GN=cof PE=1 SV=2

Query	Observed	Mr(expt)	Mr(calc)	ppm	Miss	Score	Expect	Rank	Unique	Peptide
1234	479.7369	957.4593	957.4572	2.21	0	20	0.024	1	U	R.YQIIDVK.K

---

32. 1::sp|CYC_HUMAN| Mass: 11724 Score: 19 Matches: 1(1) Sequences: 1(1) emPAI: 0.42

sp|CYC_HUMAN|

Query	Observed	Mr(expt)	Mr(calc)	ppm	Miss	Score	Expect	Rank	Unique	Peptide
5865	764.8877	1527.7609	1527.7697	-5.76	2	19	0.026	1	U	K.EERADLIAYLKK.A

---

33. 3::P0A759|NAGB_ECOLI Mass: 29983 Score: 19 Matches: 1(1) Sequences: 1(1) emPAI: 0.15

Glucosamine-6-phosphate deaminase OS=Escherichia coli (strain K12) OX=83333 GN=nagB PE=1 SV=1

Query	Observed	Mr(expt)	Mr(calc)	ppm	Miss	Score	Expect	Rank	Unique	Peptide
6044	386.9645	1543.8290	1543.8181	7.09	1	19	0.031	1	U	K.ALVEMHKAGQVSFK.H

---

34. 3::P0C018|RL18_ECOLI Score: 19 Matches: 1(1) Sequences: 1(1)

50S ribosomal protein L18 OS=Escherichia coli (strain K12) OX=83333 GN=rplR PE=1 SV=1

Query	Observed	Mr(expt)	Mr(calc)	ppm	Miss	Score	Expect	Rank	Unique	Peptide
2276	365.8537	1094.5394	1094.5485	-8.29	1	19	0.029	2	U	R.KLQELGATR.L

---

35. 3::P0A7V0|RS2_ECOLI Mass: 26784 Score: 19 Matches: 1(1) Sequences: 1(1) emPAI: 0.17

30S ribosomal protein S2 OS=Escherichia coli (strain K12) OX=83333 GN=rpsB PE=1 SV=2

Query	Observed	Mr(expt)	Mr(calc)	ppm	Miss	Score	Expect	Rank	Unique	Peptide
3937	649.8182	1297.6219	1297.6192	2.08	1	19	0.025	1	U	-.MATVSMRDMLK.A

---

36. 3::P0ACP5|GNTR_ECOLI Mass: 36570 Score: 19 Matches: 1(1) Sequences: 1(1) emPAI: 0.12

HTH-type transcriptional regulator GntR OS=Escherichia coli (strain K12) OX=83333 GN=gntR PE=1 SV=1

Query	Observed	Mr(expt)	Mr(calc)	ppm	Miss	Score	Expect	Rank	Unique	Peptide
647	428.7662	855.5179	855.5178	0.09	0	19	0.014	1	U	R.LASVLTPR.E

---

37. 3::P32053|INTA_ECOLI Mass: 46736 Score: 19 Matches: 1(1) Sequences: 1(1) emPAI: 0.10

Prophage integrase IntA OS=Escherichia coli (strain K12) OX=83333 GN=intA PE=1 SV=2

Query	Observed	Mr(expt)	Mr(calc)	ppm	Miss	Score	Expect	Rank	Unique	Peptide
705	434.7535	867.4924	867.4926	-0.32	0	19	0.014	1	U	K.AVQPVQAR.G

---

38. 3::P06149|DLD_ECOLI Mass: 64914 Score: 18 Matches: 1(1) Sequences: 1(1) emPAI: 0.07

Quinone-dependent D-lactate dehydrogenase OS=Escherichia coli (strain K12) OX=83333 GN=dld PE=1 SV=3

Query	Observed	Mr(expt)	Mr(calc)	ppm	Miss	Score	Expect	Rank	Unique	Peptide

Query	Observed	Mr(expt)	Mr(calc)	ppm	Miss	Score	Expect	Rank	Unique	Peptide
740	437.7351	873.4557	873.4556	0.12	1	18	0.022	1	U	K.LDDDRIK.D

---

39. 3::P0ADT8|YGIM_ECOLI Mass: 23062 Score: 18 Matches: 2(2) Sequences: 2(2) emPAI: 0.44

Uncharacterized protein YgiM OS=Escherichia coli (strain K12) OX=83333 GN=ygiM PE=3 SV=1

Query	Observed	Mr(expt)	Mr(calc)	ppm	Miss	Score	Expect	Rank	Unique	Peptide
1798	521.2823	1040.5500	1040.5502	-0.22	0	15	0.03	2	U	R.VPDLENQVK.T
2864	580.7947	1159.5748	1159.5721	2.38	0	16	0.03	2	U	K.VDAASVQLDDK.Q

---

40. 3::P63201|GADW_ECOLI Mass: 28353 Score: 18 Matches: 1(1) Sequences: 1(1) emPAI: 0.16

HTH-type transcriptional regulator GadW OS=Escherichia coli (strain K12) OX=83333 GN=gadW PE=1 SV=1

Query	Observed	Mr(expt)	Mr(calc)	ppm	Miss	Score	Expect	Rank	Unique	Peptide
2909	389.8357	1166.4852	1166.4930	-6.66	0	18	0.03	1	U	R.MYTSESLIK.K

---

41. 3::P22106|ASNB_ECOLI Mass: 63075 Score: 18 Matches: 1(1) Sequences: 1(1) emPAI: 0.07

Asparagine synthetase B [glutamine-hydrolyzing] OS=Escherichia coli (strain K12) OX=83333 GN=asnB PE=1 SV=3

Query	Observed	Mr(expt)	Mr(calc)	ppm	Miss	Score	Expect	Rank	Unique	Peptide
5635	753.8779	1505.7413	1505.7483	-4.62	1	18	0.04	1	U	R.KLLALHMYDCAR.A

---

42. 3::P15029|FECD_ECOLI Mass: 34337 Score: 18 Matches: 1(1) Sequences: 1(1) emPAI: 0.13

Fe(3+) dicitrate transport system permease protein FecD OS=Escherichia coli (strain K12) OX=83333 GN=fecD PE=1 SV=2

Query	Observed	Mr(expt)	Mr(calc)	ppm	Miss	Score	Expect	Rank	Unique	Peptide
3234	406.2104	1215.6092	1215.6104	-0.95	1	18	0.036	1	U	K.MLAKTHQPMK.L

---

43. 3::P21889|SYD_ECOLI Mass: 66157 Score: 18 Matches: 1(1) Sequences: 1(1) emPAI: 0.07

Aspartate--tRNA ligase OS=Escherichia coli (strain K12) OX=83333 GN=aspS PE=1 SV=1

Query	Observed	Mr(expt)	Mr(calc)	ppm	Miss	Score	Expect	Rank	Unique	Peptide
5028	724.3921	1446.7696	1446.7708	-0.84	1	18	0.024	1	U	R.VAALRVPGGASLTR.K

---

44. 3::P76104|RLHA_ECOLI Mass: 73170 Score: 17 Matches: 1(1) Sequences: 1(1) emPAI: 0.06

23S rRNA 5-hydroxycytidine C2501 synthase OS=Escherichia coli (strain K12) OX=83333 GN=rlhA PE=1 SV=2

Query	Observed	Mr(expt)	Mr(calc)	ppm	Miss	Score	Expect	Rank	Unique	Peptide
221	379.7297	757.4448	757.4446	0.23	1	17	0.021	2	U	K.QAKGNIK.S

---

45. 3::P21893|RECJ_ECOLI Mass: 63634 Score: 17 Matches: 1(1) Sequences: 1(1) emPAI: 0.07

Single-stranded-DNA-specific exonuclease RecJ OS=Escherichia coli (strain K12) OX=83333 GN=recJ PE=1 SV=2

Query	Observed	Mr(expt)	Mr(calc)	ppm	Miss	Score	Expect	Rank	Unique	Peptide
2198	543.8047	1085.5947	1085.6015	-6.24	1	17	0.034	1	U	-.MKQQIQLR.R

---

46. 3::P38035|RTCR_ECOLI Mass: 60604 Score: 17 Matches: 1(1) Sequences: 1(1) emPAI: 0.07

Transcriptional regulatory protein RtcR OS=Escherichia coli (strain K12) OX=83333 GN=rtcR PE=4 SV=2

Query	Observed	Mr(expt)	Mr(calc)	ppm	Miss	Score	Expect	Rank	Unique	Peptide
2864	580.7947	1159.5748	1159.5638	9.52	1	17	0.024	1	U	R.SRSLFETLK.R

---

47. 3::P80645|SSUD_ECOLI Mass: 41768 Score: 17 Matches: 1(1) Sequences: 1(1) emPAI: 0.11

Alkanesulfonate monooxygenase OS=Escherichia coli (strain K12) OX=83333 GN=ssuD PE=1 SV=3

Query	Observed	Mr(expt)	Mr(calc)	ppm	Miss	Score	Expect	Rank	Unique	Peptide
292	387.7093	773.4040	773.4032	1.16	0	17	0.02	1	U	R.QAATLDR.L

---

48. 3::P0AAJ8|HYBA_ECOLI Mass: 36949 Score: 17 Matches: 1(1) Sequences: 1(1) emPAI: 0.12

Hydrogenase-2 operon protein HybA OS=Escherichia coli (strain K12) OX=83333 GN=hybA PE=3 SV=1

Query	Observed	Mr(expt)	Mr(calc)	ppm	Miss	Score	Expect	Rank	Unique	Peptide
1068	466.7434	931.4723	931.4763	-4.37	0	17	0.028	1	U	K.TGIVHYDK.D

---

49. 3::P77330|BORD_ECOLI Mass: 10612 Score: 17 Matches: 1(1) Sequences: 1(1) emPAI: 0.47

Prophage lipoprotein Bor homolog OS=Escherichia coli (strain K12) OX=83333 GN=borD PE=3 SV=1

Query	Observed	Mr(expt)	Mr(calc)	ppm	Miss	Score	Expect	Rank	Unique	Peptide
128	366.7161	731.4177	731.4177	-0.10	1	17	0.022	1	U	K.KTVDAAK.I

---

50. 3::Q46901|CSE1_ECOLI Mass: 56493 Score: 17 Matches: 1(1) Sequences: 1(1) emPAI: 0.08

CRISPR system Cascade subunit CasA OS=Escherichia coli (strain K12) OX=83333 GN=casA PE=1 SV=1

Query	Observed	Mr(expt)	Mr(calc)	ppm	Miss	Score	Expect	Rank	Unique	Peptide
8915	654.0380	1959.0921	1959.0918	0.16	1	17	0.023	1	U	R.GIDLRSTVLLNVLTLPR.L

---

51. 3::P0AA43|RSUA_ECOLI Mass: 25963 Score: 17 Matches: 1(1) Sequences: 1(1) emPAI: 0.18

Ribosomal small subunit pseudouridine synthase A OS=Escherichia coli (strain K12) OX=83333 GN=rsuA PE=1 SV=1

Query	Observed	Mr(expt)	Mr(calc)	ppm	Miss	Score	Expect	Rank	Unique	Peptide
295	388.2185	774.4225	774.4236	-1.35	0	17	0.022	1	U	R.LTISEGR.Y

---

52. 3::P45522|KEFB_ECOLI Mass: 66482 Score: 17 Matches: 1(1) Sequences: 1(1) emPAI: 0.07

Glutathione-regulated potassium-efflux system protein KefB OS=Escherichia coli (strain K12) OX=83333 GN=kefB PE=3 SV=1

Query	Observed	Mr(expt)	Mr(calc)	ppm	Miss	Score	Expect	Rank	Unique	Peptide
2418	557.2803	1112.5461	1112.5413	4.32	1	17	0.049	1	U	K.MRITVLER.D

---

53. 3::P77337|YDIS_ECOLI Score: 16 Matches: 1(1) Sequences: 1(1)

Probable electron transfer flavoprotein-quinone oxidoreductase YdiS OS=Escherichia coli (strain K12) OX=83333 GN=ydiS PE=3 SV=1

Query	Observed	Mr(expt)	Mr(calc)	ppm	Miss	Score	Expect	Rank	Unique	Peptide
1058	466.2459	930.4771	930.4779	-0.81	1	16	0.023	2	U	K.MIMGHAKK.I

---

54. 3::P0AEX9|MALE_ECOLI Mass: 43360 Score: 16 Matches: 1(1) Sequences: 1(1) emPAI: 0.10

Maltose/maltodextrin-binding periplasmic protein OS=Escherichia coli (strain K12) OX=83333 GN=malE PE=1 SV=1

Query	Observed	Mr(expt)	Mr(calc)	ppm	Miss	Score	Expect	Rank	Unique	Peptide
879	452.2427	902.4709	902.4709	-0.01	0	16	0.045	1	U	R.QTVDEALK.D

---

55. 3::P33668|YBBC_ECOLI Mass: 14257 Score: 16 Matches: 1(1) Sequences: 1(1) emPAI: 0.34

Uncharacterized protein YbbC OS=Escherichia coli (strain K12) OX=83333 GN=ybbC PE=2 SV=1

Query Observed Mr(expt) Mr(calc) ppm Miss Score Expect Rank Unique Peptide
1662 509.7492 1017.4839 1017.4896 -5.57 1 16 0.035 1 U K.YSVKNIVK.T

---

56. 3::P02921|MELB_ECOLI Mass: 52830 Score: 16 Matches: 1(1) Sequences: 1(1) emPAI: 0.08
Melibiose carrier protein OS=Escherichia coli (strain K12) OX=83333 GN=melB PE=1 SV=2
Query Observed Mr(expt) Mr(calc) ppm Miss Score Expect Rank Unique Peptide
1207 478.7239 955.4331 955.4354 -2.40 0 16 0.035 1 U -.MSISMTTK.L

---

57. 3::P64622|YHDV_ECOLI Score: 16 Matches: 1(1) Sequences: 1(1)
Uncharacterized protein YhdV OS=Escherichia coli (strain K12) OX=83333 GN=yhdV PE=3 SV=1
Query Observed Mr(expt) Mr(calc) ppm Miss Score Expect Rank Unique Peptide
3121 600.2544 1198.4942 1198.4876 5.50 0 16 0.037 4 U R.LVHINTCMK.G

---

58. 3::P20966|PTFBC_ECOLI Mass: 57824 Score: 16 Matches: 1(1) Sequences: 1(1) emPAI: 0.08
PTS system fructose-specific EIIB'BC component OS=Escherichia coli (strain K12) OX=83333 GN=fruA PE=1 SV=1
Query Observed Mr(expt) Mr(calc) ppm Miss Score Expect Rank Unique Peptide
407 402.7088 803.4029 803.4025 0.58 0 16 0.025 1 U K.TAQELDK.A

---

59. 3::P08331|CPDB_ECOLI Mass: 70902 Score: 16 Matches: 1(1) Sequences: 1(1) emPAI: 0.06
2',3'-cyclic-nucleotide 2'-phosphodiesterase/3'-nucleotidase OS=Escherichia coli (strain K12) OX=83333 GN=cpdB PE=1 SV=2
Query Observed Mr(expt) Mr(calc) ppm Miss Score Expect Rank Unique Peptide
3570 627.3347 1252.6548 1252.6526 1.82 0 16 0.038 1 U K.QPMFTPYLIK.D

---

60. 3::P12999|BIOC_ECOLI Mass: 28373 Score: 16 Matches: 1(1) Sequences: 1(1) emPAI: 0.16
Malonyl-[acyl-carrier protein] O-methyltransferase OS=Escherichia coli (strain K12) OX=83333 GN=bioC PE=1 SV=2
Query Observed Mr(expt) Mr(calc) ppm Miss Score Expect Rank Unique Peptide
6002 514.6168 1540.8286 1540.8395 -7.10 1 16 0.041 1 U R.QSADALLAMLPQRK.Y

---

61. 3::P0AG96|SECE_ECOLI Mass: 13635 Score: 16 Matches: 1(1) Sequences: 1(1) emPAI: 0.36
Protein translocase subunit SecE OS=Escherichia coli (strain K12) OX=83333 GN=secE PE=1 SV=1
Query Observed Mr(expt) Mr(calc) ppm Miss Score Expect Rank Unique Peptide
1550 503.3085 1004.6024 1004.6019 0.56 0 16 0.026 1 U R.LVSFITGLR.F

---

62. 3::P14900|MURD_ECOLI Mass: 47343 Score: 16 Matches: 1(1) Sequences: 1(1) emPAI: 0.09
UDP-N-acetylmuramoylalanine--D-glutamate ligase OS=Escherichia coli (strain K12) OX=83333 GN=murD PE=1 SV=3
Query Observed Mr(expt) Mr(calc) ppm Miss Score Expect Rank Unique Peptide
2584 566.3040 1130.5934 1130.5866 5.95 1 16 0.042 1 U R.GVTPRVMDTR.M

---

63. 3::P28249|ASMA_ECOLI Mass: 68977 Score: 16 Matches: 1(1) Sequences: 1(1) emPAI: 0.06
Protein AsmA OS=Escherichia coli (strain K12) OX=83333 GN=asmA PE=3 SV=2
Query Observed Mr(expt) Mr(calc) ppm Miss Score Expect Rank Unique Peptide
4032 654.8453 1307.6760 1307.6738 1.71 0 16 0.05 1 U R.LENVEIGTILK.A

---

64. 3::P0ADF6|EDD_ECOLI Mass: 65054 Score: 16 Matches: 1(1) Sequences: 1(1) emPAI: 0.07
Phosphogluconate dehydratase OS=Escherichia coli (strain K12) OX=83333 GN=edd PE=1 SV=1
Query Observed Mr(expt) Mr(calc) ppm Miss Score Expect Rank Unique Peptide
2 350.1560 698.2974 698.3000 -3.72 0 16 0.027 1 U R.LSGASGK.V

---

65. 3::P0AD65|MRDA_ECOLI Mass: 70812 Score: 16 Matches: 2(2) Sequences: 1(1) emPAI: 0.06
Peptidoglycan D,D-transpeptidase MrdA OS=Escherichia coli (strain K12) OX=83333 GN=mrdA PE=1 SV=1
Query Observed Mr(expt) Mr(calc) ppm Miss Score Expect Rank Unique Peptide
296 388.2198 774.4250 774.4276 -3.29 0 14 0.037 1 U R.FPGVEVK.G 295

---

66. 3::P0A6N8|EFPL_ECOLI Mass: 21519 Score: 16 Matches: 1(1) Sequences: 1(1) emPAI: 0.22
Elongation factor P-like protein OS=Escherichia coli (strain K12) OX=83333 GN=yeiP PE=1 SV=1
Query Observed Mr(expt) Mr(calc) ppm Miss Score Expect Rank Unique Peptide
2199 362.8725 1085.5957 1085.6015 -5.34 2 16 0.048 1 U -.MPRANEIKK.G

---

67. 3::P43329|HRPA_ECOLI Mass: 149448 Score: 16 Matches: 1(1) Sequences: 1(1) emPAI: 0.03
ATP-dependent RNA helicase HrpA OS=Escherichia coli (strain K12) OX=83333 GN=hrpA PE=1 SV=3
Query Observed Mr(expt) Mr(calc) ppm Miss Score Expect Rank Unique Peptide
2485 561.2961 1120.5777 1120.5798 -1.84 1 16 0.05 2 U K.SMLIKEGAEK.I

---

68. 3::P0AGD7|SRP54_ECOLI Mass: 49812 Score: 16 Matches: 1(1) Sequences: 1(1) emPAI: 0.09
Signal recognition particle protein OS=Escherichia coli (strain K12) OX=83333 GN=ffh PE=1 SV=1
Query Observed Mr(expt) Mr(calc) ppm Miss Score Expect Rank Unique Peptide
1595 505.7787 1009.5428 1009.5412 1.58 3 16 0.032 1 U K.KMKKGGMAK.M

---

69. 3::P0AFA2|NARX_ECOLI Mass: 67669 Score: 15 Matches: 1(1) Sequences: 1(1) emPAI: 0.07
Nitrate/nitrite sensor protein NarX OS=Escherichia coli (strain K12) OX=83333 GN=narX PE=1 SV=1
Query Observed Mr(expt) Mr(calc) ppm Miss Score Expect Rank Unique Peptide
202 378.2058 754.3971 754.3973 -0.36 0 15 0.029 1 U K.TAGLEHK.N

---

70. 3::P0ACL0|GLPR_ECOLI Mass: 28087 Score: 15 Matches: 1(1) Sequences: 1(1) emPAI: 0.16
Glycerol-3-phosphate regulon repressor OS=Escherichia coli (strain K12) OX=83333 GN=glpR PE=1 SV=1
Query Observed Mr(expt) Mr(calc) ppm Miss Score Expect Rank Unique Peptide
416 403.7019 805.3892 805.3817 9.29 0 15 0.03 1 U K.ATQTEEK.E

Proteins matching the same set of peptides:
3::P23840|DIND_ECOLI Mass: 31173 Score: 15 Matches: 1(1) Sequences: 1(1)
DNA damage-inducible protein D OS=Escherichia coli (strain K12) OX=83333 GN=dinD PE=4 SV=1

---

71. 3::P33136|OPGG_ECOLI Mass: 57876 Score: 15 Matches: 1(1) Sequences: 1(1) emPAI: 0.08
Glucans biosynthesis protein G OS=Escherichia coli (strain K12) OX=83333 GN=mdoG PE=1 SV=1
Query Observed Mr(expt) Mr(calc) ppm Miss Score Expect Rank Unique Peptide
399 401.7250 801.4355 801.4344 1.27 0 15 0.042 1 U K.QAQSLAGK.G

72. 3::P0A7R9|RS11_ECOLI Mass: 13950 Score: 15 Matches: 1(1) Sequences: 1(1) emPAI: 0.35
30S ribosomal protein S11 OS=Escherichia coli (strain K12) OX=83333 GN=rpsK PE=1 SV=2

Query	Observed	Mr(expt)	Mr(calc)	ppm	Miss	Score	Expect	Rank	Unique	Peptide
340	393.7397	785.4648	785.4581	8.53	1	15	0.032	1	U	-.MAKAPIR.A

73. 3::P25772|LIGB_ECOLI Mass: 63425 Score: 15 Matches: 1(1) Sequences: 1(1) emPAI: 0.07
DNA ligase B OS=Escherichia coli (strain K12) OX=83333 GN=ligB PE=1 SV=2

Query	Observed	Mr(expt)	Mr(calc)	ppm	Miss	Score	Expect	Rank	Unique	Peptide
155	372.7219	743.4292	743.4290	0.30	0	15	0.034	2	U	R.VNIGSVR.R

74. 1::sp|UB2E1_HUMAN| Mass: 21676 Score: 15 Matches: 1(1) Sequences: 1(1) emPAI: 0.21
sp|UB2E1_HUMAN|

Query	Observed	Mr(expt)	Mr(calc)	ppm	Miss	Score	Expect	Rank	Unique	Peptide
1202	478.2539	954.4932	954.4899	3.47	1	15	0.034	1	U	K.LLSTSAKR.I

75. 3::P75830|MACA_ECOLI Mass: 40599 Score: 15 Matches: 1(1) Sequences: 1(1) emPAI: 0.11
Macrolide export protein MacA OS=Escherichia coli (strain K12) OX=83333 GN=macA PE=1 SV=2

Query	Observed	Mr(expt)	Mr(calc)	ppm	Miss	Score	Expect	Rank	Unique	Peptide
161	373.2138	744.4129	744.4130	-0.06	0	15	0.035	1	U	R.EVTIGAR.N

76. 3::P17169|GLMS_ECOLI Mass: 67081 Score: 14 Matches: 1(1) Sequences: 1(1) emPAI: 0.07
Glutamine--fructose-6-phosphate aminotransferase [isomerizing] OS=Escherichia coli (strain K12) OX=83333 GN=glmS PE=1 SV=4

Query	Observed	Mr(expt)	Mr(calc)	ppm	Miss	Score	Expect	Rank	Unique	Peptide
3029	594.2587	1186.5028	1186.4941	7.37	0	14	0.042	1	U	R.IEQMLSQDK.R

77. 3::P0AAH4|SAPD_ECOLI Mass: 38093 Score: 14 Matches: 1(1) Sequences: 1(1) emPAI: 0.12
Putrescine export system ATP-binding protein SapD OS=Escherichia coli (strain K12) OX=83333 GN=sapD PE=1 SV=1

Query	Observed	Mr(expt)	Mr(calc)	ppm	Miss	Score	Expect	Rank	Unique	Peptide
653	429.2455	856.4765	856.4840	-8.75	0	14	0.048	1	U	-.MPLLDIR.N

78. 3::P76015|DHAK_ECOLI Mass: 38590 Score: 14 Matches: 1(1) Sequences: 1(1) emPAI: 0.12
PEP-dependent dihydroxyacetone kinase, dihydroxyacetone-binding subunit DhaK OS=Escherichia coli (strain K12) OX=83333 GN=dhaK PE=1 SV=2

Query	Observed	Mr(expt)	Mr(calc)	ppm	Miss	Score	Expect	Rank	Unique	Peptide
103	364.7007	727.3867	727.3864	0.42	0	14	0.038	1	U	R.ADAPVAGK.V

79. 3::P77766|RNAAM_ECOLI Mass: 32902 Score: 14 Matches: 1(1) Sequences: 1(1) emPAI: 0.14
5'-3' exoribonuclease OS=Escherichia coli (strain K12) OX=83333 GN=yciV PE=1 SV=1

Query	Observed	Mr(expt)	Mr(calc)	ppm	Miss	Score	Expect	Rank	Unique	Peptide
389	400.7350	799.4555	799.4552	0.40	0	14	0.043	2	U	R.AQLIAER.L

80. 3::Q7DFV4|YMDE_ECOLI Mass: 12057 Score: 14 Matches: 1(1) Sequences: 1(1) emPAI: 0.41
Putative uncharacterized protein YmdE OS=Escherichia coli (strain K12) OX=83333 GN=ymdE PE=5 SV=3

Query	Observed	Mr(expt)	Mr(calc)	ppm	Miss	Score	Expect	Rank	Unique	Peptide
2265	547.7330	1093.4515	1093.4532	-1.54	0	14	0.04	1	U	-.MEDGVQAMR.D

81. 3::P77739|KT3K_ECOLI Mass: 32666 Score: 14 Matches: 1(1) Sequences: 1(1) emPAI: 0.14
Probable ketoamine kinase YniA OS=Escherichia coli (strain K12) OX=83333 GN=yniA PE=3 SV=1

Query	Observed	Mr(expt)	Mr(calc)	ppm	Miss	Score	Expect	Rank	Unique	Peptide
1076	467.2353	932.4560	932.4538	2.39	0	14	0.041	1	U	-.MWQAISR.L

82. 3::P16869|FHUE_ECOLI Mass: 81239 Score: 14 Matches: 1(1) Sequences: 1(1) emPAI: 0.05
FhuE receptor OS=Escherichia coli (strain K12) OX=83333 GN=fhuE PE=1 SV=2

Query	Observed	Mr(expt)	Mr(calc)	ppm	Miss	Score	Expect	Rank	Unique	Peptide
3771	638.2576	1274.5006	1274.5114	-8.43	0	14	0.043	1	U	R.LTTTLAIFR.I

83. 3::P0A9M0|LON_ECOLI Mass: 87725 Score: 14 Matches: 1(1) Sequences: 1(1) emPAI: 0.05
Lon protease OS=Escherichia coli (strain K12) OX=83333 GN=lon PE=1 SV=1

Query	Observed	Mr(expt)	Mr(calc)	ppm	Miss	Score	Expect	Rank	Unique	Peptide
349	394.7112	787.4078	787.4075	0.28	0	14	0.041	2	U	K.AEAELQK.L

84. 3::P09831|GLTB_ECOLI Mass: 164335 Score: 14 Matches: 1(1) Sequences: 1(1) emPAI: 0.03
Glutamate synthase [NADPH] large chain OS=Escherichia coli (strain K12) OX=83333 GN=gltB PE=1 SV=3

Query	Observed	Mr(expt)	Mr(calc)	ppm	Miss	Score	Expect	Rank	Unique	Peptide
1158	473.7351	945.4556	945.4572	-1.69	1	14	0.044	1	U	R.YVITKDK.L

85. 3::P0A7V3|RS3_ECOLI Mass: 25967 Score: 14 Matches: 1(1) Sequences: 1(1) emPAI: 0.18
30S ribosomal protein S3 OS=Escherichia coli (strain K12) OX=83333 GN=rpsC PE=1 SV=2

Query	Observed	Mr(expt)	Mr(calc)	ppm	Miss	Score	Expect	Rank	Unique	Peptide
411	403.2065	804.3985	804.3912	9.08	0	14	0.043	1	U	R.AVQNAMR.L

86. 3::P02918|PBPA_ECOLI Mass: 93806 Score: 14 Matches: 1(1) Sequences: 1(1) emPAI: 0.05
Penicillin-binding protein 1A OS=Escherichia coli (strain K12) OX=83333 GN=mrcA PE=1 SV=1

Query	Observed	Mr(expt)	Mr(calc)	ppm	Miss	Score	Expect	Rank	Unique	Peptide
2305	549.8038	1097.5930	1097.5941	-1.00	0	14	0.043	1	U	K.VQQAAQQAVR.N

87. 3::P37013|NORR_ECOLI Mass: 55601 Score: 14 Matches: 1(1) Sequences: 1(1) emPAI: 0.08
Anaerobic nitric oxide reductase transcription regulator NorR OS=Escherichia coli (strain K12) OX=83333 GN=norR PE=1 SV=2

Query	Observed	Mr(expt)	Mr(calc)	ppm	Miss	Score	Expect	Rank	Unique	Peptide
155	372.7219	743.4292	743.4290	0.32	0	14	0.043	3	U	R.VLAATNR.D

88. 3::P71229|HYFR_ECOLI Mass: 75771 Score: 14 Matches: 1(1) Sequences: 1(1) emPAI: 0.06
DNA-binding transcriptional activator HyfR OS=Escherichia coli (strain K12) OX=83333 GN=hyfR PE=1 SV=1

Query	Observed	Mr(expt)	Mr(calc)	ppm	Miss	Score	Expect	Rank	Unique	Peptide
155	372.7219	743.4292	743.4290	0.32	0	14	0.043	3	U	R.VIAATNR.D

89. 3::P38134|ETK_ECOLI Mass: 81191 Score: 14 Matches: 1(1) Sequences: 1(1) emPAI: 0.05

Tyrosine-protein kinase etk OS=Escherichia coli (strain K12) OX=83333 GN=etk PE=1 SV=2

Query	Observed	Mr(expt)	Mr(calc)	ppm	Miss	Score	Expect	Rank	Unique	Peptide
2384	555.2137	1108.4128	1108.4025	9.34	0	14	0.044	1	U	K.EVSLSMQR.L

---

90. 3::P55140|YGCG_ECOLI Mass: 32527 Score: 13 Matches: 1(1) Sequences: 1(1) emPAI: 0.14

UPF0603 protein YgcG OS=Escherichia coli (strain K12) OX=83333 GN=ygcG PE=3 SV=2

Query	Observed	Mr(expt)	Mr(calc)	ppm	Miss	Score	Expect	Rank	Unique	Peptide
12831	914.3105	3653.2130	3653.2305	-4.81	2	13	0.045	1	U	R.ASGRSYHSDNSGSAGGSDSGGFSGGGGSSGGGGASGRW.-

---

91. 3::P00582|DPO1_ECOLI Mass: 103168 Score: 13 Matches: 1(1) Sequences: 1(1) emPAI: 0.04

DNA polymerase I OS=Escherichia coli (strain K12) OX=83333 GN=polA PE=1 SV=1

Query	Observed	Mr(expt)	Mr(calc)	ppm	Miss	Score	Expect	Rank	Unique	Peptide
718	435.7561	869.4976	869.4971	0.59	1	13	0.046	1	U	R.NGVKIDPK.V

---

92. 3::P76352|YEEO_ECOLI Mass: 60477 Score: 13 Matches: 1(1) Sequences: 1(1) emPAI: 0.07

Probable FMN/FAD exporter YeeO OS=Escherichia coli (strain K12) OX=83333 GN=yeeO PE=1 SV=4

Query	Observed	Mr(expt)	Mr(calc)	ppm	Miss	Score	Expect	Rank	Unique	Peptide
13155	1157.1393	4624.5282	4624.5243	0.86	0	13	0.049	1	U	K.IPLLINGSLNILNIIISGILIYGLFSWPGLGFVGAGLGLTISR.Y

---

93. 3::P0AGF6|TDCB_ECOLI Mass: 35552 Score: 13 Matches: 1(1) Sequences: 1(1) emPAI: 0.13

L-threonine dehydratase catabolic TdcB OS=Escherichia coli (strain K12) OX=83333 GN=tdcB PE=1 SV=1

Query	Observed	Mr(expt)	Mr(calc)	ppm	Miss	Score	Expect	Rank	Unique	Peptide
2500	562.2236	1122.4327	1122.4247	7.13	0	13	0.05	1	U	K.LSSLTDAEK.R
