# Supplementary material for: ERK2-topoisomerase II regulatory axis is important for gene activation in immediate early genes
Source: Nat Commun. 2023 Dec 14;14:8341. doi: 10.1038/s41467-023-44089-y (PMC10721843; doi:10.1038/s41467-023-44089-y)
Supplement: Supplementary file 12 — Supplementary Data 9 [file 41467_2023_44089_MOESM12_ESM.pdf]

Mascot Search Results

User	: r-nakagawa
Email	:
Search title	: SN221366_deTOP2B_Erk2m_1ul_Node:2
MS data file	: File Name: Z:¥SN22¥SN221366_deTOP2B_Erk2m_1ul.raw;File Path: ;File Time: 3/1/2023 6:25:31 PM;File Size: 352168572 [Byte]
Database 1	: cRAP STRAV_proteinG_20230313 (118 sequences; 39090 residues)
Database 2	: Original_21 21_TOP2B_1-566_20210618 (1 sequences; 589 residues)
Database 3	: Uniprot_Proteome_E.coli_K12 Proteome_E.coli_K12_UP000000625_2022_01 (4448 sequences; 1358110 residues)
Timestamp	: 13 Sep 2023 at 00:33:54 GMT
Enzyme	: Trypsin
Fixed modifications	: Carbamidomethyl (C)
Variable modifications	: Acetyl (Protein N-term), Oxidation (M), Phospho (ST), Phospho (Y)
Mass values	: Monoisotopic
Protein Mass	: Unrestricted
Peptide Mass Tolerance	: ± 10 ppm
Fragment Mass Tolerance	: ± 0.8 Da
Max Missed Cleavages	: 3
Instrument type	: Default
Number of queries	: 13249

Protein hits :

2::TOP2B_1-566	TOP2B_1-566
1::sp|K2C1_HUMAN|	sp|K2C1_HUMAN|
1::sp|K1C10_HUMAN|	sp|K1C10_HUMAN|
1::sp|K22E_HUMAN|	sp|K22E_HUMAN|
1::sp|TRYP_PIG|	sp|TRYP_PIG|
1::sp|K1C9_HUMAN|	sp|K1C9_HUMAN|
3::P0AA25|THIO_ECOLI	Thioredoxin 1 OS=Escherichia coli (strain K12) OX=83333 GN=trxA PE=1 SV=2
1::sp|ALBU_BOVIN|	sp|ALBU_BOVIN|
3::P76298|FLHA_ECOLI	Flagellar biosynthesis protein FlhA OS=Escherichia coli (strain K12) OX=83333 GN=flhA PE=3 SV=1
3::Q46857|DKGA_ECOLI	2,5-diketo-D-gluconic acid reductase A OS=Escherichia coli (strain K12) OX=83333 GN=dkgA PE=1 SV=3
3::P0AD96|LIVJ_ECOLI	Leu/Ile/Val-binding protein OS=Escherichia coli (strain K12) OX=83333 GN=livJ PE=1 SV=1
3::P75882|GFCD_ECOLI	Uncharacterized lipoprotein GfcD OS=Escherichia coli (strain K12) OX=83333 GN=gfcD PE=3 SV=1
3::P0AAB4|UBID_ECOLI	3-octaprenyl-4-hydroxybenzoate carboxy-lyase OS=Escherichia coli (strain K12) OX=83333 GN=ubiD PE=1 SV=1
3::P13009|METH_ECOLI	Methionine synthase OS=Escherichia coli (strain K12) OX=83333 GN=metH PE=1 SV=5
3::P62601|TREF_ECOLI	Cytoplasmic trehalase OS=Escherichia coli (strain K12) OX=83333 GN=treF PE=1 SV=1
3::P0ACP5|GNTR_ECOLI	HTH-type transcriptional regulator GntR OS=Escherichia coli (strain K12) OX=83333 GN=gntR PE=1 SV=1
1::sp|RS27A_HUMAN|	sp|RS27A_HUMAN|
3::P12996|BIOB_ECOLI	Biotin synthase OS=Escherichia coli (strain K12) OX=83333 GN=bioB PE=1 SV=2
3::P77221|YAHG_ECOLI	Uncharacterized protein YahG OS=Escherichia coli (strain K12) OX=83333 GN=yahG PE=4 SV=1
3::P67660|YHAJ_ECOLI	Probable HTH-type transcriptional regulator YhaJ OS=Escherichia coli (strain K12) OX=83333 GN=yhaJ PE=3 SV=1
3::P39358|YJHG_ECOLI	D-xylonate dehydratase YjhG OS=Escherichia coli (strain K12) OX=83333 GN=yjhG PE=1 SV=2
3::P24177|ACRD_ECOLI	Probable aminoglycoside efflux pump OS=Escherichia coli (strain K12) OX=83333 GN=acrD PE=3 SV=3
3::P38035|RTCR_ECOLI	Transcriptional regulatory protein RtcR OS=Escherichia coli (strain K12) OX=83333 GN=rtcR PE=4 SV=2
3::P71242|WCAK_ECOLI	Colanic acid biosynthesis protein WcaK OS=Escherichia coli (strain K12) OX=83333 GN=wcaK PE=3 SV=2
3::P77682|GTRA_ECOLI	Prophage bactoprenol-linked glucose translocase homolog OS=Escherichia coli (strain K12) OX=83333 GN=yfdG PE=1 SV=1
3::P0AFS9|MEPM_ECOLI	Murein DD-endopeptidase MepM OS=Escherichia coli (strain K12) OX=83333 GN=mepM PE=1 SV=1
3::Q46861|YGIQ_ECOLI	UPF0313 protein YgiQ OS=Escherichia coli (strain K12) OX=83333 GN=ygiQ PE=3 SV=3
3::P0CE47|EFTU1_ECOLI	Elongation factor Tu 1 OS=Escherichia coli (strain K12) OX=83333 GN=tufA PE=1 SV=1
3::P0ABD5|ACCA_ECOLI	Acetyl-coenzyme A carboxylase carboxyl transferase subunit alpha OS=Escherichia coli (strain K12) OX=83333 GN=accA PE=1 SV=2
3::Q46793|YGEN_ECOLI	Putative uncharacterized protein YgeN OS=Escherichia coli (strain K12) OX=83333 GN=ygeN PE=5 SV=3
3::P38134|ETK_ECOLI	Tyrosine-protein kinase etk OS=Escherichia coli (strain K12) OX=83333 GN=etk PE=1 SV=2
3::P37327|YFDC_ECOLI	Inner membrane protein YfdC OS=Escherichia coli (strain K12) OX=83333 GN=yfdC PE=1 SV=1
3::V9HVX0|YPAA_ECOLI	Uncharacterized protein YpaA OS=Escherichia coli (strain K12) OX=83333 GN=ypaA PE=4 SV=1
3::P16384|MIAA_ECOLI	tRNA dimethylallyltransferase OS=Escherichia coli (strain K12) OX=83333 GN=miaA PE=1 SV=2
3::P63224|GMHA_ECOLI	Phosphoheptose isomerase OS=Escherichia coli (strain K12) OX=83333 GN=gmhA PE=1 SV=1
3::P0A8Z3|YBGC_ECOLI	Acyl-CoA thioester hydrolase YbgC OS=Escherichia coli (strain K12) OX=83333 GN=ybgC PE=1 SV=1
1::sp|KRHB1_HUMAN|	sp|KRHB1_HUMAN|
3::P25772|LIGB_ECOLI	DNA ligase B OS=Escherichia coli (strain K12) OX=83333 GN=ligB PE=1 SV=2
3::P0A759|NAGB_ECOLI	Glucosamine-6-phosphate deaminase OS=Escherichia coli (strain K12) OX=83333 GN=nagB PE=1 SV=1
3::P0ADT8|YGIM_ECOLI	Uncharacterized protein YgiM OS=Escherichia coli (strain K12) OX=83333 GN=ygiM PE=3 SV=1
3::P0AGD7|SRP54_ECOLI	Signal recognition particle protein OS=Escherichia coli (strain K12) OX=83333 GN=ffh PE=1 SV=1
1::sp|KRHB4_HUMAN|	sp|KRHB4_HUMAN|
3::P77211|CUSC_ECOLI	Cation efflux system protein CusC OS=Escherichia coli (strain K12) OX=83333 GN=cusC PE=1 SV=1
3::P69811|PTFAH_ECOLI	Multiphosphoryl transfer protein OS=Escherichia coli (strain K12) OX=83333 GN=fruB PE=1 SV=1
3::P0AEE5|DGAL_ECOLI	D-galactose-binding periplasmic protein OS=Escherichia coli (strain K12) OX=83333 GN=mglB PE=1 SV=1
3::P15028|FECB_ECOLI	Fe(3+) dicitrate-binding periplasmic protein OS=Escherichia coli (strain K12) OX=83333 GN=fecB PE=2 SV=3
3::Q06065|ATOC_ECOLI	Regulatory protein AtoC OS=Escherichia coli (strain K12) OX=83333 GN=atoC PE=1 SV=2
3::P00634|PPB_ECOLI	Alkaline phosphatase OS=Escherichia coli (strain K12) OX=83333 GN=phoA PE=1 SV=1
3::P06992|RSMA_ECOLI	Ribosomal RNA small subunit methyltransferase A OS=Escherichia coli (strain K12) OX=83333 GN=rsmA PE=1 SV=1
3::P77377|WZXC_ECOLI	Lipopolysaccharide biosynthesis protein WzxC OS=Escherichia coli (strain K12) OX=83333 GN=wzxC PE=1 SV=1
3::P33668|YBBC_ECOLI	Uncharacterized protein YbbC OS=Escherichia coli (strain K12) OX=83333 GN=ybbC PE=2 SV=1
3::P80645|SSUD_ECOLI	Alkanesulfonate monooxygenase OS=Escherichia coli (strain K12) OX=83333 GN=ssuD PE=1 SV=3
3::P13738|NHAA_ECOLI	Na(+)/H(+) antiporter NhaA OS=Escherichia coli (strain K12) OX=83333 GN=nhaA PE=1 SV=3
3::P31434|XYLS_ECOLI	Alpha-xylosidase OS=Escherichia coli (strain K12) OX=83333 GN=yicI PE=1 SV=2
3::P76104|RLHA_ECOLI	23S rRNA 5-hydroxycytidine C2501 synthase OS=Escherichia coli (strain K12) OX=83333 GN=rlhA PE=1 SV=2
3::P21893|RECJ_ECOLI	Single-stranded-DNA-specific exonuclease RecJ OS=Escherichia coli (strain K12) OX=83333 GN=recJ PE=1 SV=2
3::P76016|DHAR_ECOLI	PTS-dependent dihydroxyacetone kinase operon regulatory protein OS=Escherichia coli (strain K12) OX=83333 GN=dhaR PE=1 SV=2
3::P0AFE8|NUOM_ECOLI	NADH-quinone oxidoreductase subunit M OS=Escherichia coli (strain K12) OX=83333 GN=nuoM PE=1 SV=1
3::P09372|GRPE_ECOLI	Protein GrpE OS=Escherichia coli (strain K12) OX=83333 GN=grpE PE=1 SV=1
3::P28917|YDCC_ECOLI	H repeat-associated putative transposase YdcC OS=Escherichia coli (strain K12) OX=83333 GN=ydcC PE=3 SV=1
3::P37007|YAGA_ECOLI	Uncharacterized protein YagA OS=Escherichia coli (strain K12) OX=83333 GN=yagA PE=2 SV=3
3::P28912|YHHI_ECOLI	H repeat-associated putative transposase YhhI OS=Escherichia coli (strain K12) OX=83333 GN=yhhI PE=3 SV=1
3::P28630|HOLA_ECOLI	DNA polymerase III subunit delta OS=Escherichia coli (strain K12) OX=83333 GN=holA PE=1 SV=1
3::P0AAK7|NRFC_ECOLI	Protein NrfC OS=Escherichia coli (strain K12) OX=83333 GN=nrfC PE=3 SV=1
3::P37650|BCSC_ECOLI	Cellulose synthase operon protein C OS=Escherichia coli (strain K12) OX=83333 GN=bcsC PE=1 SV=3
3::P0AFB8|NTRC_ECOLI	DNA-binding transcriptional regulator NtrC OS=Escherichia coli (strain K12) OX=83333 GN=glnG PE=1 SV=1
3::P0AG96|SECE_ECOLI	Protein translocase subunit SecE OS=Escherichia coli (strain K12) OX=83333 GN=secE PE=1 SV=1
3::P02925|RBSB_ECOLI	Ribose import binding protein RbsB OS=Escherichia coli (strain K12) OX=83333 GN=rbsB PE=1 SV=1
3::P0AEG4|DSBA_ECOLI	Thiol:disulfide interchange protein DsbA OS=Escherichia coli (strain K12) OX=83333 GN=dsbA PE=1 SV=1
3::P77766|RNAAM_ECOLI	5'-3' exoribonuclease OS=Escherichia coli (strain K12) OX=83333 GN=yciV PE=1 SV=1
3::P39347|INTB_ECOLI	Putative prophage integrase IntB OS=Escherichia coli (strain K12) OX=83333 GN=intB PE=5 SV=1
3::P00957|SYA_ECOLI	Alanine--tRNA ligase OS=Escherichia coli (strain K12) OX=83333 GN=alaS PE=1 SV=2
3::P16869|FHUE_ECOLI	FhuE receptor OS=Escherichia coli (strain K12) OX=83333 GN=fhuE PE=1 SV=2
3::P77658|YNAA_ECOLI	Protein YnaA OS=Escherichia coli (strain K12) OX=83333 GN=ynaA PE=4 SV=1
3::P50456|MLC_ECOLI	Protein mlc OS=Escherichia coli (strain K12) OX=83333 GN=mlc PE=1 SV=2
3::P02943|LAMB_ECOLI	Maltoporin OS=Escherichia coli (strain K12) OX=83333 GN=lamB PE=1 SV=1
3::P0ADF6|EDD_ECOLI	Phosphogluconate dehydratase OS=Escherichia coli (strain K12) OX=83333 GN=edd PE=1 SV=1
3::P37902|GLTI_ECOLI	Glutamate/aspartate import solute-binding protein OS=Escherichia coli (strain K12) OX=83333 GN=gltI PE=1 SV=2
3::P28635|METQ_ECOLI	D-methionine-binding lipoprotein MetQ OS=Escherichia coli (strain K12) OX=83333 GN=metQ PE=1 SV=2
3::P23842|PDEA_ECOLI	Probable cyclic di-GMP phosphodiesterase PdeA OS=Escherichia coli (strain K12) OX=83333 GN=pdeA PE=2 SV=2
3::P56258|WECF_ECOLI	TDP-N-acetylfucosamine:lipid II N-acetylfucosaminyltransferase OS=Escherichia coli (strain K12) OX=83333 GN=wecF PE=1 SV=2
3::P31224|ACRB_ECOLI	Multidrug efflux pump subunit AcrB OS=Escherichia coli (strain K12) OX=83333 GN=acrB PE=1 SV=1

	Target	Decoy	False discovery rate
Peptide matches above identity threshold	487	57	11.70 %
Peptide matches above homology or identity threshold	488	60	12.30 %

Select Summary Report

Format As [Select Summary (protein hits)] Help Help

Significance threshold p< 0.05 Max. number of hits AUTO Show Percolator scores ☐

Standard scoring ○ MudPIT scoring ◉ Display non-significant matches ☐ Show sub-sets 0

Show pop-ups ◉ Suppress pop-ups ○ Require bold red ☐

Preferred taxonomy [All entries]

Re-Search ◉ All queries ○ Unassigned ○ Below homology threshold ○ Below identity threshold

1. 2::TOP2B_1-566 Mass: 66629 Score: 7213 Matches: 298(298) Sequences: 44(44) emPAI: 30.81

TOP2B_1-566

Query	Observed	Mr(expt)	Mr(calc)	ppm	Miss	Score	Expect	Rank	Unique	Peptide
377	401.7245	801.4344	801.4344	-0.07	0	32	0.00087	1	U	K.AQTQLNK.K 376
447	410.7502	819.4858	819.4854	0.51	0	38	0.00018	1	U	K.IVGLQYK.K 441 445 446
482	414.7811	827.5477	827.5480	-0.40	1	32	0.00059	1	U	K.LIEVVKK.K
596	426.2430	850.4714	850.4701	1.51	0	28	0.0014	1	U	R.YGVFPLR.G 592 594 595
730	439.7585	877.5024	877.5022	0.32	0	23	0.0057	1	U	K.GIPVVEHK.V 729

937	459.7572	917.4999	917.5004	-0.61	0	(49)	2e-005	1	U	K.DIVALMTR.R
1006	465.7717	929.5287	929.5294	-0.71	1	27	0.0028	1	U	K.AQTQLNKK.C 1008
1040	467.7553	933.4960	933.4953	0.67	0	49	1.7e-005	1	U	K.DIVALMTR.R 1035 1036 1038 1039
1097	473.2661	944.5177	944.5179	-0.13	0	49	2e-005	1	U	K.LDETGVALK.V
1141	478.2311	954.4477	954.4481	-0.38	0	25	0.0046	1	U	K.FTVETACK.E
1186	480.7448	959.4749	959.4746	0.37	0	(20)	0.014	1	U	K.ENMTLQPK.S 1184 1187
1274	488.2226	974.4307	974.4305	0.26	0	60	1.3e-006	1	U	K.LDDANDAGGK.H 1273
1282	488.7422	975.4699	975.4695	0.40	0	22	0.0093	1	U	K.ENMTLQPK.S 1278 1280 1281 1283
1323	491.7551	981.4957	981.4953	0.39	0	39	0.00022	1	U	K.LCNIFSTK.F
1340	493.7636	985.5126	985.5120	0.60	0	43	5.8e-005	1	U	R.SYVDLYVK.D 1338 1339
1486	505.2439	1008.4732	1008.4732	0.03	1	(21)	0.0097	1	U	R.DKNMTCIK.V
1504	506.7295	1011.4445	1011.4444	0.10	0	30	0.0015	1	U	R.AYDLAGSCR.G
1601	513.2413	1024.4680	1024.4681	-0.09	1	25	0.0061	1	U	R.DKNMTCIK.V
1612	514.2329	1026.4512	1026.4505	0.61	0	39	0.00012	1	U	K.SYDDAESLK.T 1609 1610 1611
1666	518.2512	1034.4879	1034.4880	-0.10	1	42	0.00011	1	U	K.KEESETANK.N
2010	539.3061	1076.5977	1076.5978	-0.09	1	31	0.0011	1	U	K.FKAQTQLNK.K
2033	360.8646	1079.5720	1079.5723	-0.33	0	(36)	0.00041	1	U	K.VIHELANER.W 2030 2031 2034 2038 2039 2040 2041 2043 2045 2046
2044	540.7935	1079.5725	1079.5723	0.15	0	50	1.6e-005	1	U	K.VIHELANER.W 2035 2036 2037 2042 2047 2048 2049
2374	374.8733	1121.5980	1121.5982	-0.15	1	(16)	0.039	1	U	R.DRYGVFPLR.G
2375	561.8063	1121.5981	1121.5982	-0.10	1	31	0.0011	1	U	R.DRYGVFPLR.G
2436	376.8959	1127.6660	1127.6663	-0.24	0	(34)	0.00036	1	U	K.SLAVSGLGVIGR.D
2445	564.8406	1127.6666	1127.6663	0.33	0	72	6.6e-008	1	U	K.SLAVSGLGVIGR.D 2432 2433 2434 2437 2438 2439 2440 2441 2442 2443 2444 2446 2447 2448
2654	576.8192	1151.6237	1151.6227	0.94	0	20	0.012	1	U	R.EVTFVPGLYK.I 2646 2647 2651 2655
2666	578.2800	1154.5454	1154.5455	-0.12	1	32	0.0015	1	U	K.KSYDDAESLK.T 2667 2670 2671
2710	580.3448	1158.6751	1158.6761	-0.84	0	47	2.6e-005	1	U	K.AGVSVKPFQVK.N 2715 2721 2722 2723 2727
2716	387.2326	1158.6760	1158.6761	-0.06	0	(35)	0.00043	1	U	K.AGVSVKPFQVK.N 2711 2718 2719 2725 2726
2884	592.2566	1182.4986	1182.4984	0.17	0	(22)	0.0072	1	U	K.QTWMNNMMK.T
2923	594.8270	1187.6394	1187.6398	-0.33	1	48	3e-005	1	U	K.DKLDETGVALK.V 2924 2928 2930
2925	396.8872	1187.6397	1187.6398	-0.09	1	(31)	0.0015	1	U	K.DKLDETGVALK.V 2926
2994	600.2536	1198.4927	1198.4933	-0.51	0	34	0.00046	1	U	K.QTWMNNMMK.T 2997
2996	600.2540	1198.4935	1198.4933	0.16	0	(33)	0.00078	1	U	K.QTWMNNMMK.T 2998
3101	608.2514	1214.4882	1214.4882	0.02	0	(31)	0.00088	1	U	K.QTWMNNMMK.T 3099 3103 3105
3102	608.2514	1214.4882	1214.4882	0.02	0	(25)	0.0029	1	U	K.QTWMNNMMK.T 3097 3098 3100
3104	608.2521	1214.4896	1214.4882	1.16	0	(33)	0.00051	1	U	K.QTWMNNMMK.T
3403	625.8080	1249.6014	1249.6013	0.12	0	42	0.00013	1	U	R.WDVCLTLSEK.G
3627	637.8606	1273.7067	1273.7064	0.28	1	(62)	9.9e-007	1	U	K.LDKDIVALMTR.R 3618 3620 3621 3623
3629	425.5763	1273.7071	1273.7064	0.54	1	(31)	0.0012	1	U	K.LDKDIVALMTR.R 3616 3624 3625
3750	430.9077	1289.7013	1289.7013	0.02	1	(41)	0.00017	1	U	K.LDKDIVALMTR.R 3744 3745 3747 3749 3753 3755 3757
3756	645.8582	1289.7018	1289.7013	0.40	1	68	3.1e-007	1	U	K.LDKDIVALMTR.R 3742 3743 3746 3751 3752 3754
4214	679.3588	1356.7031	1356.7038	-0.51	0	81	2.4e-008	1	U	R.HVDYVVDQVVGK.L 4212 4216 4217 4218 4219 4220 4221 4222 4223 4224 4225 4227 4228 4230
4215	453.2417	1356.7032	1356.7038	-0.43	0	(40)	0.00029	1	U	R.HVDYVVDQVVGK.L 4211 4213 4226 4229
4957	731.3826	1460.7507	1460.7511	-0.28	0	80	2.6e-008	1	U	K.IFDEILVNAADNK.Q 4956 4958 4960 4961 4964 4970 4972 4973 4974 4975 4976 4977
4965	487.9243	1460.7512	1460.7511	0.07	0	(48)	5e-005	1	U	K.IFDEILVNAADNK.Q 4951 4952 4953 4954 4955 4959 4962 4963 4966 4967 4968 4969 4971 4978
5646	510.6045	1528.7917	1528.7919	-0.11	0	(51)	1.9e-005	1	U	K.QIMENAEINNIK.I 5647
5813	773.4005	1544.7865	1544.7868	-0.21	0	64	1e-006	1	U	K.QIMENAEINNIK.I 5812 5814 5815 5816
5992	783.8661	1565.7177	1565.7169	0.53	2	52	2.7e-005	1	U	K.KEESETANKNDSSK.K 5991
6525	547.6266	1639.8579	1639.8570	0.55	0	(57)	4.1e-006	1	U	K.GFQQISFVNSIATTK.G 6509 6520 6527 6528
6529	820.9366	1639.8587	1639.8570	1.08	0	95	7.1e-010	1	U	K.GFQQISFVNSIATTK.G 6503 6504 6505 6511 6514 6515 6516 6517 6518 6519 6521 6522 6524 6531
6939	565.6116	1693.8129	1693.8118	0.61	3	25	0.012	1	U	K.KEESETANKNDSSKK.L
7215	577.9397	1730.7971	1730.7968	0.21	0	(34)	0.0016	1	U	K.IMIMTDQDQDGSHIK.A 7214 7222 7223
7219	433.7066	1730.7974	1730.7968	0.38	0	(28)	0.0056	1	U	K.IMIMTDQDQDGSHIK.A
7221	866.4061	1730.7976	1730.7968	0.51	0	(77)	7.4e-008	1	U	K.IMIMTDQDQDGSHIK.A 7211 7218 7229
7322	874.4024	1746.7902	1746.7917	-0.83	0	(75)	8.6e-008	1	U	K.IMIMTDQDQDGSHIK.A 7326
7328	874.4030	1746.7914	1746.7917	-0.13	0	84	1.2e-008	1	U	K.IMIMTDQDQDGSHIK.A 7323 7327
7334	583.2715	1746.7926	1746.7917	0.52	0	(49)	3e-005	1	U	K.IMIMTDQDQDGSHIK.A 7324 7331 7333 7335 7336 7338 7339
7441	588.6024	1762.7852	1762.7866	-0.78	0	(61)	2.9e-006	1	U	K.IMIMTDQDQDGSHIK.A 7442 7443 7444 7446 7451 7453 7454 7456 7457 7460
7448	882.4004	1762.7862	1762.7866	-0.22	0	(76)	9.5e-008	1	U	K.IMIMTDQDQDGSHIK.A 7450 7452 7455 7459
7515	591.9609	1772.8609	1772.8615	-0.31	0	(27)	0.0078	1	U	K.HSLECTLILTEGDSAK.S
7516	887.4382	1772.8618	1772.8615	0.19	0	112	2.5e-011	1	U	K.HSLECTLILTEGDSAK.S
8293	943.4856	1884.9567	1884.9581	-0.76	0	63	1.1e-006	1	U	K.VSIDPESNIISIWNNGK.G 8295
8294	629.3264	1884.9573	1884.9581	-0.44	0	(58)	3.2e-006	1	U	K.VSIDPESNIISIWNNGK.G
9383	724.9898	2171.9476	2171.9470	0.29	0	33	0.0022	1	U	K.HFDGEDYTCITFQPDLSK.F
9481	735.0160	2202.0261	2202.0236	1.12	0	23	0.025	1	U	K.SGGCGAGAGVGGGNGALTWVNNAAK.K
10220	829.7476	2486.2210	2486.2217	-0.26	0	(61)	4.1e-006	1	U	K.VYVPALIFGQLLTSSNYDDDEK.K 10221 10222 10227
10226	1244.1185	2486.2225	2486.2217	0.32	0	92	3.2e-009	1	U	K.VYVPALIFGQLLTSSNYDDDEK.K 10223 10225 10229
10414	859.4247	2575.2522	2575.2529	-0.31	0	52	3.5e-005	1	U	K.NHIWVFINCLIENPTFDSQTK.E 10415
10503	872.4463	2614.3172	2614.3166	0.21	1	(62)	3.4e-006	1	U	K.VYVPALIFGQLLTSSNYDDDEKK.V 10496 10498 10499 10500 10502 10505 10506
10504	654.5866	2614.3172	2614.3166	0.22	1	(46)	0.00012	1	U	K.VYVPALIFGQLLTSSNYDDDEKK.V 10497
10510	1308.1668	2614.3191	2614.3166	0.94	1	71	3.6e-007	1	U	K.VYVPALIFGQLLTSSNYDDDEKK.V 10509 10512
11576	772.1536	3084.5853	3084.5768	2.77	2	22	0.025	1	U	K.VYVPALIFGQLLTSSNYDDDEKKVTGGR.N
12981	1082.2728	4325.0620	4325.0595	0.56	0	(29)	0.012	1	U	K.TQLEHILLRPDTYIGSVEPLTQFMWVYDEDVGMNCR.E
12986	1086.2689	4341.0466	4341.0545	-1.81	0	41	0.00073	1	U	K.TQLEHILLRPDTYIGSVEPLTQFMWVYDEDVGMNCR.E

---

2. 1::sp|K2C1_HUMAN| Mass: 66018 Score: 1215 Matches: 31(31) Sequences: 22(22) emPAI: 3.71

sp|K2C1_HUMAN|

Query	Observed	Mr(expt)	Mr(calc)	ppm	Miss	Score	Expect	Rank	Unique	Peptide
714	437.7535	873.4924	873.4920	0.47	0	36	0.00032	1	U	R.SLVNLGGSK.S
1261	487.2695	972.5245	972.5240	0.53	0	34	0.00056	1		K.IEISELNR.V
1415	500.2264	998.4382	998.4379	0.29	0	45	5.4e-005	1	U	K.DVDGAYMTK.V
1655	517.2617	1032.5088	1032.5087	0.08	0	41	0.0001	1	U	R.TLLEGEESR.M
1966	537.3015	1072.5885	1072.5876	0.77	1	27	0.0061	1	U	R.LRSEIDNVK.K
2151	546.7555	1091.4964	1091.4956	0.71	0	62	7e-007	1	U	R.GSGGGSSGGSIGGR.G

Query	Observed	Mr(expt)	Mr(calc)	ppm	Miss	Score	Expect	Rank	Unique	Peptide
2405	563.2749	1124.5353	1124.5349	0.32	0	56	5.1e-006	1	U	K.AEAESLYQSK.Y
2681	579.2608	1156.5070	1156.5070	-0.04	0	28	0.0026	1	U	R.DYQELMNTK.L
2860	590.3041	1178.5936	1178.5931	0.44	0	65	6.8e-007	1	U	K.YEELQITAGR.H
3653	639.3584	1276.7023	1276.7027	-0.25	0	70	1.7e-007	1	U	K.LALDLEIATYR.T
3817	650.7681	1299.5216	1299.5224	-0.59	0	(44)	5.3e-005	1	U	K.NMQDMVEDYR.N
3847	651.8611	1301.7077	1301.7078	-0.06	0	69	2.8e-007	1	U	R.SLDLDSIIAEVK.A
3887	436.8899	1307.6478	1307.6469	0.65	2	38	0.00042	1	U	R.NKYEDEINKR.T
3953	658.7659	1315.5172	1315.5173	-0.05	0	58	1.6e-006	1	U	K.NMQDMVEDYR.N
3954	658.7660	1315.5175	1315.5173	0.21	0	(39)	0.00012	1	U	K.NMQDMVEDYR.N
4080	666.7642	1331.5138	1331.5122	1.21	0	(37)	0.00019	1	U	K.NMQDMVEDYR.N
4122	670.8383	1339.6621	1339.6619	0.15	1	60	2.1e-006	1	U	K.SKAEAESLYQSK.Y
4411	692.3492	1382.6839	1382.6830	0.68	0	64	7.2e-007	1	U	K.SLNNQFASFIDK.V
5100	738.3784	1474.7422	1474.7416	0.39	0	56	7.4e-006	1	U	K.WELLQQVDTSTR.T
6630	829.3996	1656.7846	1656.7856	-0.57	0	74	1.7e-007	1	U	R.SGGGFSSGSAGIINYQR.R
7104	858.9293	1715.8441	1715.8438	0.18	0	85	1.1e-008	1	U	K.QISNLQQSISDAEQR.G 7103
8055	615.6531	1843.9375	1843.9388	-0.67	1	23	0.015	1	U	K.KQISNLQQSISDAEQR.G
8786	665.3296	1992.9670	1992.9693	-1.19	0	46	9.1e-005	1	U	R.THNLEPYFESFINNLR.R
9881	1192.4787	2382.9428	2382.9447	-0.79	0	87	3.7e-009	1	U	R.GGGGGGYGSGGSSYGSGGGSYGSGGGGGGGR.G 9880
9883	795.3223	2382.9451	2382.9447	0.17	0	(61)	1.3e-006	1	U	R.GGGGGGYGSGGSSYGSGGGSYGSGGGGGGGR.G 9882 9884
12161	1104.7745	3311.3016	3311.3009	0.20	0	87	6.9e-009	1	U	R.GSYGSGGSSYGSGGGSYGSGGGGGGHGSYGSGSSSGGYR.G 12160

3. 1::sp|K1C10_HUMAN| Mass: 59711 Score: 1015 Matches: 32(32) Sequences: 19(19) emPAI: 3.81

sp|K1C10_HUMAN|

Query	Observed	Mr(expt)	Mr(calc)	ppm	Miss	Score	Expect	Rank	Unique	Peptide
398	404.2036	806.3926	806.3923	0.41	0	38	0.00019	1	U	R.LAADDFR.L
405	405.2239	808.4332	808.4330	0.15	0	20	0.0095	1		R.LASYLDK.V
569	424.2297	846.4448	846.4447	0.11	0	22	0.01	1	U	K.SEITELR.R
924	458.6957	915.3768	915.3756	1.35	0	29	0.0015	1	U	K.NHEEEMK.D
1384	497.2531	992.4916	992.4927	-1.08	0	46	4.1e-005	1	U	K.YENEVALR.Q
1445	502.2800	1002.5454	1002.5458	-0.33	1	23	0.0074	1	U	K.SEITELRR.N
2125	545.7691	1089.5236	1089.5237	-0.09	0	(45)	4.8e-005	1	U	K.VTMQNLNDR.L
2237	553.7668	1105.5191	1105.5186	0.46	0	48	3.1e-005	1	U	K.VTMQNLNDR.L
3010	601.3123	1200.6101	1200.6098	0.22	0	52	1.5e-005	1	U	R.QSVEADINGLR.R
3249	617.8429	1233.6713	1233.6717	-0.28	1	(29)	0.0016	1	U	R.LKYENEVALR.Q
3250	412.2312	1233.6717	1233.6717	-0.03	1	39	0.00018	1	U	R.LKYENEVALR.Q
3499	631.8026	1261.5906	1261.5899	0.61	0	68	3.2e-007	1	U	R.SLLEGEGSSGGGGR.G
4288	683.3232	1364.6318	1364.6320	-0.17	0	78	2.8e-008	1	U	R.SQYEQLAEQNR.K 4289
4397	691.3281	1380.6416	1380.6408	0.54	0	100	3.2e-010	1	U	R.ALEESNYELEGK.I
4719	717.8885	1433.7624	1433.7626	-0.17	1	60	2.2e-006	1	U	K.IRLENEIQTYR.S
4720	478.9284	1433.7635	1433.7626	0.60	1	(16)	0.046	1	U	K.IRLENEIQTYR.S
5247	498.5833	1492.7279	1492.7270	0.64	1	(25)	0.0082	1	U	R.SQYEQLAEQNRK.D
5248	747.3715	1492.7284	1492.7270	0.97	1	46	6.5e-005	1	U	R.SQYEQLAEQNRK.D 5246
7035	854.3902	1706.7658	1706.7649	0.55	0	138	3.6e-014	1	U	K.GSLGGGFSSGGFSGGSFSR.G
9126	699.3524	2095.0354	2095.0394	-1.94	0	35	0.0013	1	U	K.ADLEMQIESLTEELAYLK.K 9127
9863	789.7597	2366.2573	2366.2553	0.83	0	(65)	7.7e-007	1	U	K.NQILNLTTDNANILLQIDNAR.L
9864	1184.1365	2366.2585	2366.2553	1.35	0	67	3.9e-007	1	U	K.NQILNLTTDNANILLQIDNAR.L
11001	958.1358	2871.3855	2871.3855	-0.00	0	66	1.7e-006	1	U	R.NVSTGDVNVEMNAAPGVDLTQLLNNMR.S
11021	963.4680	2887.3821	2887.3804	0.59	0	(50)	7.4e-005	1	U	R.NVSTGDVNVEMNAAPGVDLTQLLNNMR.S 11019
11022	963.4681	2887.3824	2887.3804	0.71	0	(55)	1.9e-005	1	U	R.NVSTGDVNVEMNAAPGVDLTQLLNNMR.S 11020
11055	968.8000	2903.3782	2903.3753	0.98	0	(21)	0.048	1	U	R.NVSTGDVNVEMNAAPGVDLTQLLNNMR.S
11494	1018.2141	3051.6204	3051.6200	0.12	1	30	0.0027	1	U	K.TIDDLKNQILNLTTDNANILLQIDNAR.L

4. 1::sp|K22E_HUMAN| Mass: 66110 Score: 813 Matches: 20(20) Sequences: 19(19) emPAI: 2.41

sp|K22E_HUMAN|

Query	Observed	Mr(expt)	Mr(calc)	ppm	Miss	Score	Expect	Rank	Unique	Peptide
491	416.2501	830.4857	830.4862	-0.54	0	32	0.00056	1	U	R.SLVGLGGTK.S
1261	487.2695	972.5245	972.5240	0.53	0	34	0.00056	1		K.IEISELNR.V
1675	519.2672	1036.5198	1036.5189	0.85	0	48	3.4e-005	1	U	R.YLDGLTAER.T
1698	521.2824	1040.5503	1040.5502	0.11	0	41	7.4e-005	1	U	K.VDPEIQNVK.A
2244	554.2753	1106.5360	1106.5356	0.39	0	47	2.6e-005	1	U	K.AQYEEIAQR.S
2958	597.3118	1192.6091	1192.6088	0.28	0	57	4.6e-006	1	U	K.YEELQVTVGR.H
2962	398.8752	1193.6037	1193.6040	-0.24	2	31	0.0012	1	U	K.KYEDEINKR.T
2980	599.2788	1196.5430	1196.5422	0.69	0	42	0.00012	1	U	K.GGSISGGGYGSGGGK.H
3057	604.8117	1207.6088	1207.6085	0.32	0	65	5e-007	1	U	R.TAAENDFVTLK.K
3433	627.8074	1253.6003	1253.6001	0.19	0	77	4.9e-008	1	U	R.GFSSGSAVVSGGSR.R
3988	660.7948	1319.5750	1319.5756	-0.45	0	79	2.5e-008	1	U	R.HGGGGGGFGGGGFGSR.S
3989	440.8658	1319.5755	1319.5756	-0.05	0	(34)	0.00072	1	U	R.HGGGGGGFGGGGFGSR.S
4051	665.3234	1328.6321	1328.6320	0.09	0	72	1.1e-007	1	U	K.NVQDAIADAEQR.G
4328	686.3595	1370.7044	1370.7041	0.23	0	78	3e-008	1	U	K.LNDLEEALQQAK.E
4456	464.5649	1390.6728	1390.6728	-0.04	1	45	6.1e-005	1	U	R.SKEEAEALYHSK.Y
6215	794.8454	1587.6762	1587.6761	0.05	0	77	4e-008	1	U	R.GGSSSGGGYGSGGGGSSSVK.G
7280	870.8569	1739.6992	1739.6983	0.51	0	102	1.1e-010	1	U	R.GSSSGGGYSSGSSSYGSGGR.Q
7293	871.3780	1740.7413	1740.7412	0.07	0	84	1.3e-008	1	U	R.GGSGGGGSISGGGYGSGGGSGGR.Y
11482	1016.8350	3047.4830	3047.4870	-1.30	0	28	0.012	1	U	K.VLYDAEISQIHQSVTDTNVILSMDNSR.N
12926	1023.7731	4091.0635	4091.0654	-0.49	0	31	0.0051	1	U	R.FGGFGGPGGVGGLGGPGGFGPGGYPGGIHEVSVNQSLLQPLNVK.V

5. 1::sp|TRYP_PIG| Mass: 25078 Score: 271 Matches: 11(11) Sequences: 2(2) emPAI: 0.40

sp|TRYP_PIG|

Query	Observed	Mr(expt)	Mr(calc)	ppm	Miss	Score	Expect	Rank	Unique	Peptide
550	421.7587	841.5029	841.5022	0.92	0	54	4.1e-006	1	U	R.VATVSLPR.S 540 542 543 544 545 546 547 548 551
1733	523.2855	1044.5565	1044.5564	0.19	0	36	0.00057	1	U	K.LSSPATLNSR.V

6. 1::sp|K1C9_HUMAN| Mass: 62320 Score: 174 Matches: 6(6) Sequences: 6(6) emPAI: 0.51

sp|K1C9_HUMAN|

Query	Observed	Mr(expt)	Mr(calc)	ppm	Miss	Score	Expect	Rank	Unique	Peptide
405	405.2239	808.4332	808.4330	0.15	0	20	0.0095	1		R.LASYLDK.V
1870	530.7864	1059.5583	1059.5560	2.13	0	19	0.02	1	U	K.TLLDIDNTR.M
1903	533.2532	1064.4919	1064.4920	-0.10	0	44	4.9e-005	1	U	K.STMQELNSR.L
6188	793.8866	1585.7586	1585.7583	0.15	0	74	1.2e-007	1	U	K.VQALEEANNDLENK.I
7637	896.3673	1790.7200	1790.7205	-0.29	0	64	8e-007	1	U	R.GGSGGSYGGGGSGGGYGGGSGSR.G
12070	1088.8423	3263.5050	3263.5066	-0.48	0	31	0.0065	1	U	K.DIENQYETQITQIEHEVSSSGQEVQSSAK.E

7. 3::P0AA25|THIO_ECOLI Mass: 11913 Score: 137 Matches: 2(2) Sequences: 2(2) emPAI: 1.01

Thioredoxin 1 OS=Escherichia coli (strain K12) OX=83333 GN=trxA PE=1 SV=2

Query	Observed	Mr(expt)	Mr(calc)	ppm	Miss	Score	Expect	Rank	Unique	Peptide
3557	634.3358	1266.6571	1266.6568	0.21	0	69	2.9e-007	1	U	K.LNIDQNPGTAPK.Y
7808	903.4531	1804.8916	1804.8917	-0.04	0	85	1.1e-008	1	U	K.MIAPILDEIADEYQGK.L

8. 1::sp|ALBU_BOVIN| Mass: 71244 Score: 123 Matches: 5(5) Sequences: 4(4) emPAI: 0.27

sp|ALBU_BOVIN|

Query	Observed	Mr(expt)	Mr(calc)	ppm	Miss	Score	Expect	Rank	Unique	Peptide
952	461.7476	921.4807	921.4807	-0.06	0	36	0.00036	1	U	K.AEFVEVTK.L
2758	582.3191	1162.6237	1162.6234	0.31	0	30	0.0014	1	U	K.LVNELTEFAK.T
3867	653.3625	1304.7105	1304.7088	1.25	0	43	7.9e-005	1	U	K.HLVDEPQNLIK.Q 3866
6495	547.3181	1638.9324	1638.9305	1.20	1	35	0.00029	1	U	R.KVPQVSTPTLVEVSR.S

9. 3::P76298|FLHA_ECOLI Mass: 74795 Score: 92 Matches: 14(14) Sequences: 1(1) emPAI: 0.06

Flagellar biosynthesis protein FlhA OS=Escherichia coli (strain K12) OX=83333 GN=flhA PE=3 SV=1

Query	Observed	Mr(expt)	Mr(calc)	ppm	Miss	Score	Expect	Rank	Unique	Peptide
3513	632.8193	1263.6241	1263.6159	6.55	1	(19)	0.021	1	U	R.HIRMTATIGGK.- 3530 3538
3525	632.8197	1263.6249	1263.6159	7.12	1	26	0.0047	1	U	R.HIRMTATIGGK.- 3514 3515 3516 3519 3524 3527 3528 3534 3536 3540

10. 3::Q46857|DKGA_ECOLI Mass: 31147 Score: 34 Matches: 2(2) Sequences: 1(1) emPAI: 0.15

2,5-diketo-D-gluconic acid reductase A OS=Escherichia coli (strain K12) OX=83333 GN=dkgA PE=1 SV=3

Query	Observed	Mr(expt)	Mr(calc)	ppm	Miss	Score	Expect	Rank	Unique	Peptide
1011	466.2456	930.4766	930.4844	-8.41	0	25	0.0034	1	U	-.MANPTVIK.L 1012

11. 3::P0AD96|LIVJ_ECOLI Mass: 39223 Score: 31 Matches: 1(1) Sequences: 1(1) emPAI: 0.11

Leu/Ile/Val-binding protein OS=Escherichia coli (strain K12) OX=83333 GN=livJ PE=1 SV=1

Query	Observed	Mr(expt)	Mr(calc)	ppm	Miss	Score	Expect	Rank	Unique	Peptide
369	400.7352	799.4558	799.4552	0.75	0	31	0.00082	1	U	K.QAVAVANK.V

Proteins matching the same set of peptides:

3::P04816|LIVK_ECOLI Mass: 39468 Score: 31 Matches: 1(1) Sequences: 1(1)

Leucine-specific-binding protein OS=Escherichia coli (strain K12) OX=83333 GN=livK PE=1 SV=2

12. 3::P75882|GFCD_ECOLI Mass: 78923 Score: 31 Matches: 3(3) Sequences: 1(1) emPAI: 0.06

Uncharacterized lipoprotein GfcD OS=Escherichia coli (strain K12) OX=83333 GN=gfcD PE=3 SV=1

Query	Observed	Mr(expt)	Mr(calc)	ppm	Miss	Score	Expect	Rank	Unique	Peptide
1552	509.2826	1016.5506	1016.5502	0.40	1	22	0.0089	1	U	K.DKGATIDVAK.R 1548 1550

13. 3::P0AAB4|UBID_ECOLI Mass: 55967 Score: 28 Matches: 1(1) Sequences: 1(1) emPAI: 0.08

3-octaprenyl-4-hydroxybenzoate carboxy-lyase OS=Escherichia coli (strain K12) OX=83333 GN=ubiD PE=1 SV=1

Query	Observed	Mr(expt)	Mr(calc)	ppm	Miss	Score	Expect	Rank	Unique	Peptide
12975	1078.2271	4308.8795	4308.8650	3.37	2	28	0.011	1	U	R.MDPARDTVLVENTPIDYLDFASPVSGLGSKMGLDATNK.W

14. 3::P13009|METH_ECOLI Mass: 136653 Score: 28 Matches: 2(2) Sequences: 2(2) emPAI: 0.06

Methionine synthase OS=Escherichia coli (strain K12) OX=83333 GN=metH PE=1 SV=5

Query	Observed	Mr(expt)	Mr(calc)	ppm	Miss	Score	Expect	Rank	Unique	Peptide
414	406.7352	811.4559	811.4552	0.84	0	22	0.0061	1	U	R.AVEGLAPR.K
3663	427.1728	1278.4965	1278.5080	-8.99	1	19	0.017	1	U	-.MSSKVEQLR.A

15. 3::P62601|TREF_ECOLI Mass: 63828 Score: 27 Matches: 1(1) Sequences: 1(1) emPAI: 0.07

Cytoplasmic trehalase OS=Escherichia coli (strain K12) OX=83333 GN=treF PE=1 SV=1

Query	Observed	Mr(expt)	Mr(calc)	ppm	Miss	Score	Expect	Rank	Unique	Peptide
6426	812.4229	1622.8312	1622.8280	1.98	1	27	0.0044	1	U	K.LESAIANISALKGEK.E

16. 3::P0ACP5|GNTR_ECOLI Mass: 36570 Score: 26 Matches: 1(1) Sequences: 1(1) emPAI: 0.12

HTH-type transcriptional regulator GntR OS=Escherichia coli (strain K12) OX=83333 GN=gntR PE=1 SV=1

Query	Observed	Mr(expt)	Mr(calc)	ppm	Miss	Score	Expect	Rank	Unique	Peptide
612	428.7661	855.5176	855.5178	-0.24	0	26	0.0025	1	U	R.LASVLTPR.E

17. 1::sp|RS27A_HUMAN| Mass: 18296 Score: 25 Matches: 1(1) Sequences: 1(1) emPAI: 0.26

sp|RS27A_HUMAN|

Query	Observed	Mr(expt)	Mr(calc)	ppm	Miss	Score	Expect	Rank	Unique	Peptide
7607	894.4668	1786.9191	1786.9200	-0.51	0	25	0.0093	1	U	K.TITLEVEPSDTIENVK.A

18. 3::P12996|BIOB_ECOLI Mass: 39079 Score: 25 Matches: 1(1) Sequences: 1(1) emPAI: 0.11

Biotin synthase OS=Escherichia coli (strain K12) OX=83333 GN=bioB PE=1 SV=2

Query	Observed	Mr(expt)	Mr(calc)	ppm	Miss	Score	Expect	Rank	Unique	Peptide
217	379.7296	757.4446	757.4446	0.03	1	25	0.0041	1	U	K.VRDAGIK.V

19. 3::P77221|YAHG_ECOLI Mass: 50740 Score: 24 Matches: 1(1) Sequences: 1(1) emPAI: 0.09

Uncharacterized protein YahG OS=Escherichia coli (strain K12) OX=83333 GN=yahG PE=4 SV=1

Query	Observed	Mr(expt)	Mr(calc)	ppm	Miss	Score	Expect	Rank	Unique	Peptide

Query	Observed	Mr(expt)	Mr(calc)	ppm	Miss	Score	Expect	Rank	Unique	Peptide
11466	1012.7716	3035.2930	3035.2863	2.18	0	24	0.019	1	U	-.MSQSLFSQPLNVINVGIAMFSDDLK.K

20. 3::P67660|YHAJ_ECOLI Mass: 33293 Score: 24 Matches: 1(1) Sequences: 1(1) emPAI: 0.14
Probable HTH-type transcriptional regulator YhaJ OS=Escherichia coli (strain K12) OX=83333 GN=yhaJ PE=3 SV=1

Query	Observed	Mr(expt)	Mr(calc)	ppm	Miss	Score	Expect	Rank	Unique	Peptide
11542	767.8403	3067.3323	3067.3025	9.73	2	24	0.025	1	U	R.LEQGRADIVIAPDMHFRSSSEINSR.K

21. 3::P39358|YJHG_ECOLI Mass: 70486 Score: 23 Matches: 1(1) Sequences: 1(1) emPAI: 0.06
D-xylonate dehydratase YjhG OS=Escherichia coli (strain K12) OX=83333 GN=yjhG PE=1 SV=2

Query	Observed	Mr(expt)	Mr(calc)	ppm	Miss	Score	Expect	Rank	Unique	Peptide
150	372.7220	743.4294	743.4290	0.57	0	23	0.0051	1	U	K.VQTIGAR.F

Proteins matching the same set of peptides:
3::P77596|YAGF_ECOLI Mass: 69926 Score: 23 Matches: 1(1) Sequences: 1(1)
D-xylonate dehydratase YagF OS=Escherichia coli (strain K12) OX=83333 GN=yagF PE=1 SV=1

22. 3::P24177|ACRD_ECOLI Mass: 113203 Score: 22 Matches: 1(1) Sequences: 1(1) emPAI: 0.04
Probable aminoglycoside efflux pump OS=Escherichia coli (strain K12) OX=83333 GN=acrD PE=3 SV=3

Query	Observed	Mr(expt)	Mr(calc)	ppm	Miss	Score	Expect	Rank	Unique	Peptide
13075	1105.2711	4417.0552	4417.0516	0.80	2	22	0.05	1	U	R.EATRKSMGQIQGALVGIAMVLSAVFVPMAFFGGTTGAIYR.Q

23. 3::P38035|RTCR_ECOLI Mass: 60604 Score: 22 Matches: 1(1) Sequences: 1(1) emPAI: 0.07
Transcriptional regulatory protein RtcR OS=Escherichia coli (strain K12) OX=83333 GN=rtcR PE=4 SV=2

Query	Observed	Mr(expt)	Mr(calc)	ppm	Miss	Score	Expect	Rank	Unique	Peptide
2732	580.7935	1159.5725	1159.5638	7.46	1	22	0.0075	1	U	R.SRSLFETLK.R

24. 3::P71242|WCAK_ECOLI Mass: 47598 Score: 22 Matches: 1(1) Sequences: 1(1) emPAI: 0.09
Colanic acid biosynthesis protein WcaK OS=Escherichia coli (strain K12) OX=83333 GN=wcaK PE=3 SV=2

Query	Observed	Mr(expt)	Mr(calc)	ppm	Miss	Score	Expect	Rank	Unique	Peptide
781	445.7564	889.4982	889.4981	0.16	1	22	0.012	1	U	K.RSNITTAK.V

25. 3::P77682|GTRA_ECOLI Mass: 13388 Score: 21 Matches: 1(1) Sequences: 1(1) emPAI: 0.36
Prophage bactoprenol-linked glucose translocase homolog OS=Escherichia coli (strain K12) OX=83333 GN=yfdG PE=1 SV=1

Query	Observed	Mr(expt)	Mr(calc)	ppm	Miss	Score	Expect	Rank	Unique	Peptide
4860	484.1993	1449.5762	1449.5765	-0.17	1	21	0.012	1	U	K.FTFKASTTTMR.Y

26. 3::P0AFS9|MEPM_ECOLI Mass: 49027 Score: 20 Matches: 1(1) Sequences: 1(1) emPAI: 0.09
Murein DD-endopeptidase MepM OS=Escherichia coli (strain K12) OX=83333 GN=mepM PE=1 SV=1

Query	Observed	Mr(expt)	Mr(calc)	ppm	Miss	Score	Expect	Rank	Unique	Peptide
8174	620.6276	1858.8610	1858.8703	-5.02	2	20	0.037	1	U	R.EQSQLLGVRLRSEGK.D

27. 3::Q46861|YGIQ_ECOLI Mass: 84223 Score: 20 Matches: 1(1) Sequences: 1(1) emPAI: 0.05
UPF0313 protein YgiQ OS=Escherichia coli (strain K12) OX=83333 GN=ygiQ PE=3 SV=3

Query	Observed	Mr(expt)	Mr(calc)	ppm	Miss	Score	Expect	Rank	Unique	Peptide
6156	528.5885	1582.7436	1582.7449	-0.83	1	20	0.029	1	U	K.TYVLLPSFEKVK.G

28. 3::P0CE47|EFTU1_ECOLI Mass: 43427 Score: 19 Matches: 1(1) Sequences: 1(1) emPAI: 0.10
Elongation factor Tu 1 OS=Escherichia coli (strain K12) OX=83333 GN=tufA PE=1 SV=1

Query	Observed	Mr(expt)	Mr(calc)	ppm	Miss	Score	Expect	Rank	Unique	Peptide
2658	577.2888	1152.5631	1152.5652	-1.84	1	19	0.031	1	U	R.GTVVTGRVER.G

Proteins matching the same set of peptides:
3::P0CE48|EFTU2_ECOLI Mass: 43457 Score: 19 Matches: 1(1) Sequences: 1(1)
Elongation factor Tu 2 OS=Escherichia coli (strain K12) OX=83333 GN=tufB PE=1 SV=1

29. 3::P0ABD5|ACCA_ECOLI Mass: 35333 Score: 19 Matches: 1(1) Sequences: 1(1) emPAI: 0.13
Acetyl-coenzyme A carboxylase carboxyl transferase subunit alpha OS=Escherichia coli (strain K12) OX=83333 GN=accA PE=1 SV=2

Query	Observed	Mr(expt)	Mr(calc)	ppm	Miss	Score	Expect	Rank	Unique	Peptide
11987	807.7025	3226.7809	3226.7747	1.92	3	19	0.02	1	U	R.LKELKLIDSIIPEPLGGAHRNPEAMAASLK.A

30. 3::Q46793|YGEN_ECOLI Mass: 27575 Score: 19 Matches: 1(1) Sequences: 1(1) emPAI: 0.17
Putative uncharacterized protein YgeN OS=Escherichia coli (strain K12) OX=83333 GN=ygeN PE=5 SV=3

Query	Observed	Mr(expt)	Mr(calc)	ppm	Miss	Score	Expect	Rank	Unique	Peptide
5685	765.8949	1529.7752	1529.7710	2.76	3	19	0.034	1	U	-.MRKKIEMSLIK.S

31. 3::P38134|ETK_ECOLI Mass: 81191 Score: 18 Matches: 1(1) Sequences: 1(1) emPAI: 0.05
Tyrosine-protein kinase etk OS=Escherichia coli (strain K12) OX=83333 GN=etk PE=1 SV=2

Query	Observed	Mr(expt)	Mr(calc)	ppm	Miss	Score	Expect	Rank	Unique	Peptide
4508	467.8997	1400.6773	1400.6754	1.35	1	18	0.038	1	U	K.YFPIVGRGWAR.L

32. 3::P37327|YFDC_ECOLI Mass: 34538 Score: 18 Matches: 1(1) Sequences: 1(1) emPAI: 0.13
Inner membrane protein YfdC OS=Escherichia coli (strain K12) OX=83333 GN=yfdC PE=1 SV=1

Query	Observed	Mr(expt)	Mr(calc)	ppm	Miss	Score	Expect	Rank	Unique	Peptide
1003	465.7541	929.4936	929.4930	0.62	1	18	0.016	1	U	K.AERAENIK.K

33. 3::V9HVX0|YPAA_ECOLI Mass: 6876 Score: 18 Matches: 1(1) Sequences: 1(1) emPAI: 0.80
Uncharacterized protein YpaA OS=Escherichia coli (strain K12) OX=83333 GN=ypaA PE=4 SV=1

Query	Observed	Mr(expt)	Mr(calc)	ppm	Miss	Score	Expect	Rank	Unique	Peptide
5000	732.8884	1463.7623	1463.7555	4.66	1	18	0.039	1	U	K.IALRMLEQGFDR.D

34. 3::P16384|MIAA_ECOLI Mass: 35157 Score: 18 Matches: 2(2) Sequences: 1(1) emPAI: 0.13
tRNA dimethylallyltransferase OS=Escherichia coli (strain K12) OX=83333 GN=miaA PE=1 SV=2

Query	Observed	Mr(expt)	Mr(calc)	ppm	Miss	Score	Expect	Rank	Unique	Peptide
2792	390.8832	1169.6278	1169.6292	-1.23	0	17	0.025	1	U	R.DEVLQVVGAIAG.-

2793	585.8216	1169.6285	1169.6292	-0.57	0	(15)	0.04	1	U	R.DEVLQVVGAIAG.-

35. 3::P63224|GMHA_ECOLI Mass: 20973 Score: 18 Matches: 1(1) Sequences: 1(1) emPAI: 0.22
Phosphoheptose isomerase OS=Escherichia coli (strain K12) OX=83333 GN=gmhA PE=1 SV=1

Query	Observed	Mr(expt)	Mr(calc)	ppm	Miss	Score	Expect	Rank	Unique	Peptide
4854	725.3482	1448.6817	1448.6929	-7.72	1	18	0.035	1	U	K.DGGKMAGTADIEIR.V

36. 3::P0A8Z3|YBGC_ECOLI Mass: 15609 Score: 18 Matches: 1(1) Sequences: 1(1) emPAI: 0.31
Acyl-CoA thioester hydrolase YbgC OS=Escherichia coli (strain K12) OX=83333 GN=ybgC PE=1 SV=1

Query	Observed	Mr(expt)	Mr(calc)	ppm	Miss	Score	Expect	Rank	Unique	Peptide
3110	406.1946	1215.5620	1215.5594	2.15	0	18	0.032	1	U	K.MTVEYYAPAR.L

37. 1::sp|KRHB1_HUMAN| Mass: 56875 Score: 18 Matches: 1(1) Sequences: 1(1) emPAI: 0.08
sp|KRHB1_HUMAN|

Query	Observed	Mr(expt)	Mr(calc)	ppm	Miss	Score	Expect	Rank	Unique	Peptide
410	406.2213	810.4280	810.4276	0.48	0	18	0.017	1	U	R.FAAFIDK.V

Proteins matching the same set of peptides:
1::sp|K2M2_SHEEP| Mass: 55472 Score: 18 Matches: 1(1) Sequences: 1(1)
sp|K2M2_SHEEP|
1::sp|K2M3_SHEEP| Mass: 56418 Score: 18 Matches: 1(1) Sequences: 1(1)
sp|K2M3_SHEEP|
1::sp|KRHB3_HUMAN| Mass: 56004 Score: 18 Matches: 1(1) Sequences: 1(1)
sp|KRHB3_HUMAN|
1::sp|KRHB5_HUMAN| Mass: 57306 Score: 18 Matches: 1(1) Sequences: 1(1)
sp|KRHB5_HUMAN|
1::sp|KRHB6_HUMAN| Mass: 55120 Score: 18 Matches: 1(1) Sequences: 1(1)
sp|KRHB6_HUMAN|

38. 3::P25772|LIGB_ECOLI Mass: 63425 Score: 17 Matches: 1(1) Sequences: 1(1) emPAI: 0.07
DNA ligase B OS=Escherichia coli (strain K12) OX=83333 GN=ligB PE=1 SV=2

Query	Observed	Mr(expt)	Mr(calc)	ppm	Miss	Score	Expect	Rank	Unique	Peptide
150	372.7220	743.4294	743.4290	0.57	0	17	0.018	2	U	R.VNIGSVR.R

39. 3::P0A759|NAGB_ECOLI Mass: 29983 Score: 17 Matches: 1(1) Sequences: 1(1) emPAI: 0.15
Glucosamine-6-phosphate deaminase OS=Escherichia coli (strain K12) OX=83333 GN=nagB PE=1 SV=1

Query	Observed	Mr(expt)	Mr(calc)	ppm	Miss	Score	Expect	Rank	Unique	Peptide
1221	483.2577	964.5008	964.4978	3.20	1	17	0.039	1	U	R.QYEEKIR.S

40. 3::P0ADT8|YGIM_ECOLI Mass: 23062 Score: 17 Matches: 2(2) Sequences: 2(2) emPAI: 0.44
Uncharacterized protein YgiM OS=Escherichia coli (strain K12) OX=83333 GN=ygiM PE=3 SV=1

Query	Observed	Mr(expt)	Mr(calc)	ppm	Miss	Score	Expect	Rank	Unique	Peptide
1698	521.2824	1040.5503	1040.5502	0.11	0	14	0.038	2	U	R.VPDLENQVK.T
2732	580.7935	1159.5725	1159.5721	0.33	0	16	0.028	2	U	K.VDAASVQLDDK.Q

41. 3::P0AGD7|SRP54_ECOLI Mass: 49812 Score: 17 Matches: 1(1) Sequences: 1(1) emPAI: 0.09
Signal recognition particle protein OS=Escherichia coli (strain K12) OX=83333 GN=ffh PE=1 SV=1

Query	Observed	Mr(expt)	Mr(calc)	ppm	Miss	Score	Expect	Rank	Unique	Peptide
1496	505.7783	1009.5421	1009.5412	0.83	3	17	0.028	1	U	K.KMKKGGMAK.M

42. 1::sp|KRHB4_HUMAN| Mass: 65938 Score: 17 Matches: 1(1) Sequences: 1(1) emPAI: 0.07
sp|KRHB4_HUMAN|

Query	Observed	Mr(expt)	Mr(calc)	ppm	Miss	Score	Expect	Rank	Unique	Peptide
1552	509.2826	1016.5506	1016.5502	0.43	2	17	0.028	2	U	K.KDEKEQIK.T

43. 3::P77211|CUSC_ECOLI Mass: 50410 Score: 17 Matches: 1(1) Sequences: 1(1) emPAI: 0.09
Cation efflux system protein CusC OS=Escherichia coli (strain K12) OX=83333 GN=cusC PE=1 SV=1

Query	Observed	Mr(expt)	Mr(calc)	ppm	Miss	Score	Expect	Rank	Unique	Peptide
9863	789.7597	2366.2573	2366.2706	-5.61	1	17	0.047	2	U	K.IEIPIFNAGRNQANLDIAEIR.Q

44. 3::P69811|PTFAH_ECOLI Mass: 39624 Score: 17 Matches: 1(1) Sequences: 1(1) emPAI: 0.11
Multiphosphoryl transfer protein OS=Escherichia coli (strain K12) OX=83333 GN=fruB PE=1 SV=1

Query	Observed	Mr(expt)	Mr(calc)	ppm	Miss	Score	Expect	Rank	Unique	Peptide
6393	540.2932	1617.8577	1617.8474	6.31	1	17	0.04	1	U	R.LRFTAQGADAEQALK.A

45. 3::P0AEE5|DGAL_ECOLI Mass: 35690 Score: 17 Matches: 1(1) Sequences: 1(1) emPAI: 0.13
D-galactose-binding periplasmic protein OS=Escherichia coli (strain K12) OX=83333 GN=mglB PE=1 SV=1

Query	Observed	Mr(expt)	Mr(calc)	ppm	Miss	Score	Expect	Rank	Unique	Peptide
839	451.7506	901.4866	901.4869	-0.30	1	17	0.04	1	U	R.KAIEQDAK.A

46. 3::P15028|FECB_ECOLI Mass: 33126 Score: 17 Matches: 1(1) Sequences: 1(1) emPAI: 0.14
Fe(3+) dicitrate-binding periplasmic protein OS=Escherichia coli (strain K12) OX=83333 GN=fecB PE=2 SV=3

Query	Observed	Mr(expt)	Mr(calc)	ppm	Miss	Score	Expect	Rank	Unique	Peptide
75	359.2105	716.4065	716.4068	-0.44	0	17	0.021	1	U	R.IAADTVK.I

47. 3::Q06065|ATOC_ECOLI Mass: 52428 Score: 17 Matches: 2(2) Sequences: 1(1) emPAI: 0.08
Regulatory protein AtoC OS=Escherichia coli (strain K12) OX=83333 GN=atoC PE=1 SV=2

Query	Observed	Mr(expt)	Mr(calc)	ppm	Miss	Score	Expect	Rank	Unique	Peptide
3531	422.2157	1263.6252	1263.6203	3.93	1	16	0.046	1	U	R.MPEMDGIKALK.E 3532

48. 3::P00634|PPB_ECOLI Mass: 49636 Score: 17 Matches: 1(1) Sequences: 1(1) emPAI: 0.09
Alkaline phosphatase OS=Escherichia coli (strain K12) OX=83333 GN=phoA PE=1 SV=1

Query	Observed	Mr(expt)	Mr(calc)	ppm	Miss	Score	Expect	Rank	Unique	Peptide
3209	614.2891	1226.5636	1226.5601	2.86	1	17	0.042	1	U	R.KCYGPSATSEK.C

49. 3::P06992|RSMA_ECOLI Mass: 30515 Score: 17 Matches: 1(1) Sequences: 1(1) emPAI: 0.15
Ribosomal RNA small subunit methyltransferase A OS=Escherichia coli (strain K12) OX=83333 GN=rsmA PE=1 SV=1

Query	Observed	Mr(expt)	Mr(calc)	ppm	Miss	Score	Expect	Rank	Unique	Peptide
156	373.2140	744.4135	744.4130	0.62	0	17	0.022	1	U	K.VDSAVVR.L

50. 3::P77377|WZXC_ECOLI Mass: 53886 Score: 17 Matches: 1(1) Sequences: 1(1) emPAI: 0.08
Lipopolysaccharide biosynthesis protein WzxC OS=Escherichia coli (strain K12) OX=83333 GN=wzxC PE=1 SV=1

Query	Observed	Mr(expt)	Mr(calc)	ppm	Miss	Score	Expect	Rank	Unique	Peptide
5672	510.6170	1528.8292	1528.8395	-6.75	1	17	0.04	1	U	R.SVGNPIGSLLMAKAR.V

51. 3::P33668|YBBC_ECOLI Mass: 14257 Score: 16 Matches: 1(1) Sequences: 1(1) emPAI: 0.34
Uncharacterized protein YbbC OS=Escherichia coli (strain K12) OX=83333 GN=ybbC PE=2 SV=1

Query	Observed	Mr(expt)	Mr(calc)	ppm	Miss	Score	Expect	Rank	Unique	Peptide
1556	509.7492	1017.4839	1017.4896	-5.57	1	16	0.035	1	U	K.YSVKNTVK.T

52. 3::P80645|SSUD_ECOLI Mass: 41768 Score: 16 Matches: 1(1) Sequences: 1(1) emPAI: 0.11
Alkanesulfonate monooxygenase OS=Escherichia coli (strain K12) OX=83333 GN=ssuD PE=1 SV=3

Query	Observed	Mr(expt)	Mr(calc)	ppm	Miss	Score	Expect	Rank	Unique	Peptide
285	387.7088	773.4031	773.4032	-0.03	0	16	0.024	1	U	R.QAATLDR.L

53. 3::P13738|NHAA_ECOLI Mass: 41500 Score: 16 Matches: 1(1) Sequences: 1(1) emPAI: 0.11
Na(+)/H(+) antiporter NhaA OS=Escherichia coli (strain K12) OX=83333 GN=nhaA PE=1 SV=3

Query	Observed	Mr(expt)	Mr(calc)	ppm	Miss	Score	Expect	Rank	Unique	Peptide
12915	1014.3229	4053.2625	4053.2390	5.80	2	16	0.024	1	U	R.RTGVYILVGVVLWTAVLKSGVHATLAGVIVGFFIPLK.E

54. 3::P31434|XYLS_ECOLI Mass: 88764 Score: 16 Matches: 1(1) Sequences: 1(1) emPAI: 0.05
Alpha-xylosidase OS=Escherichia coli (strain K12) OX=83333 GN=yicI PE=1 SV=2

Query	Observed	Mr(expt)	Mr(calc)	ppm	Miss	Score	Expect	Rank	Unique	Peptide
1613	514.2439	1026.4733	1026.4648	8.30	1	16	0.05	1	U	R.LHGSKSYR.V

55. 3::P76104|RLHA_ECOLI Mass: 73170 Score: 16 Matches: 1(1) Sequences: 1(1) emPAI: 0.06
23S rRNA 5-hydroxycytidine C2501 synthase OS=Escherichia coli (strain K12) OX=83333 GN=rlhA PE=1 SV=2

Query	Observed	Mr(expt)	Mr(calc)	ppm	Miss	Score	Expect	Rank	Unique	Peptide
217	379.7296	757.4446	757.4446	0.05	1	16	0.03	2	U	K.QAKGNIK.S

56. 3::P21893|RECJ_ECOLI Mass: 63634 Score: 16 Matches: 1(1) Sequences: 1(1) emPAI: 0.07
Single-stranded-DNA-specific exonuclease RecJ OS=Escherichia coli (strain K12) OX=83333 GN=recJ PE=1 SV=2

Query	Observed	Mr(expt)	Mr(calc)	ppm	Miss	Score	Expect	Rank	Unique	Peptide
2098	543.8044	1085.5943	1085.6015	-6.68	1	16	0.043	1	U	-.MKQQIQLR.R

57. 3::P76016|DHAR_ECOLI Mass: 71088 Score: 16 Matches: 1(1) Sequences: 1(1) emPAI: 0.06
PTS-dependent dihydroxyacetone kinase operon regulatory protein OS=Escherichia coli (strain K12) OX=83333 GN=dhaR PE=1 SV=2

Query	Observed	Mr(expt)	Mr(calc)	ppm	Miss	Score	Expect	Rank	Unique	Peptide
8370	634.6957	1901.0653	1901.0638	0.79	0	16	0.036	1	U	R.AITELLTLPAVLQQAIK.Q

58. 3::P0AFE8|NUOM_ECOLI Mass: 56658 Score: 16 Matches: 1(1) Sequences: 1(1) emPAI: 0.08
NADH-quinone oxidoreductase subunit M OS=Escherichia coli (strain K12) OX=83333 GN=nuoM PE=1 SV=1

Query	Observed	Mr(expt)	Mr(calc)	ppm	Miss	Score	Expect	Rank	Unique	Peptide
1065	471.2400	940.4654	940.4743	-9.45	1	16	0.029	1	U	K.TRITAATK.F

59. 3::P09372|GRPE_ECOLI Mass: 21784 Score: 16 Matches: 1(1) Sequences: 1(1) emPAI: 0.21
Protein GrpE OS=Escherichia coli (strain K12) OX=83333 GN=grpE PE=1 SV=1

Query	Observed	Mr(expt)	Mr(calc)	ppm	Miss	Score	Expect	Rank	Unique	Peptide
2088	543.3196	1084.6246	1084.6353	-9.87	2	16	0.038	1	U	R.ERDGILRVK.A

60. 3::P28917|YDCC_ECOLI Mass: 43507 Score: 15 Matches: 1(1) Sequences: 1(1) emPAI: 0.10
H repeat-associated putative transposase YdcC OS=Escherichia coli (strain K12) OX=83333 GN=ydcC PE=3 SV=1

Query	Observed	Mr(expt)	Mr(calc)	ppm	Miss	Score	Expect	Rank	Unique	Peptide
723	439.2244	876.4342	876.4375	-3.72	0	15	0.028	1	U	K.ELEMTVR.Y

61. 3::P37007|YAGA_ECOLI Mass: 44026 Score: 15 Matches: 1(1) Sequences: 1(1) emPAI: 0.10
Uncharacterized protein YagA OS=Escherichia coli (strain K12) OX=83333 GN=yagA PE=2 SV=3

Query	Observed	Mr(expt)	Mr(calc)	ppm	Miss	Score	Expect	Rank	Unique	Peptide
217	379.7296	757.4446	757.4446	0.03	1	15	0.034	3	U	R.GERVGLK.E

62. 3::P28912|YHHI_ECOLI Mass: 43574 Score: 15 Matches: 1(1) Sequences: 1(1) emPAI: 0.10
H repeat-associated putative transposase YhhI OS=Escherichia coli (strain K12) OX=83333 GN=yhhI PE=3 SV=1

Query	Observed	Mr(expt)	Mr(calc)	ppm	Miss	Score	Expect	Rank	Unique	Peptide
2081	362.4979	1084.4720	1084.4624	8.86	1	15	0.034	1	U	K.KEPEMTVR.Y

Proteins matching the same set of peptides:
3::P75741|YBFL_ECOLI Mass: 43532 Score: 15 Matches: 1(1) Sequences: 1(1)
Putative protein YbfL OS=Escherichia coli (strain K12) OX=83333 GN=ybfL PE=5 SV=3

63. 3::P28630|HOLA_ECOLI Mass: 38965 Score: 15 Matches: 1(1) Sequences: 1(1) emPAI: 0.12
DNA polymerase III subunit delta OS=Escherichia coli (strain K12) OX=83333 GN=holA PE=1 SV=1

Query	Observed	Mr(expt)	Mr(calc)	ppm	Miss	Score	Expect	Rank	Unique	Peptide
995	464.7822	927.5499	927.5502	-0.29	0	15	0.031	1	U	R.QAVQLLTR.T

64. 3::P0AAK7|NRFC_ECOLI Mass: 25464 Score: 15 Matches: 1(1) Sequences: 1(1) emPAI: 0.18
Protein NrfC OS=Escherichia coli (strain K12) OX=83333 GN=nrfC PE=3 SV=1

Query Observed Mr(expt) Mr(calc) ppm Miss Score Expect Rank Unique Peptide

628 430.2534 858.4923 858.4923 0.02 1 15 0.048 1 U R.KTNLQAGK.L

65. 3::P37650|BCSC_ECOLI Mass: 127760 Score: 15 Matches: 1(1) Sequences: 1(1) emPAI: 0.03
Cellulose synthase operon protein C OS=Escherichia coli (strain K12) OX=83333 GN=bcsC PE=1 SV=3

Query	Observed	Mr(expt)	Mr(calc)	ppm	Miss	Score	Expect	Rank	Unique	Peptide
1282	488.7422	975.4699	975.4733	-3.52	1	15	0.043	2	U	K.SNAGREGASK.I

66. 3::P0AFB8|NTRC_ECOLI Mass: 52336 Score: 15 Matches: 1(1) Sequences: 1(1) emPAI: 0.08
DNA-binding transcriptional regulator NtrC OS=Escherichia coli (strain K12) OX=83333 GN=glnG PE=1 SV=1

Query	Observed	Mr(expt)	Mr(calc)	ppm	Miss	Score	Expect	Rank	Unique	Peptide
681	435.7076	869.4006	869.4048	-4.84	0	15	0.04	1	U	R.VGGYAPVK.V

67. 3::P0AG96|SECE_ECOLI Mass: 13635 Score: 15 Matches: 1(1) Sequences: 1(1) emPAI: 0.36
Protein translocase subunit SecE OS=Escherichia coli (strain K12) OX=83333 GN=secE PE=1 SV=1

Query	Observed	Mr(expt)	Mr(calc)	ppm	Miss	Score	Expect	Rank	Unique	Peptide
1455	503.3082	1004.6019	1004.6019	0.08	0	15	0.035	1	U	R.LVSFITGLR.F

68. 3::P02925|RBSB_ECOLI Mass: 30931 Score: 14 Matches: 1(1) Sequences: 1(1) emPAI: 0.15
Ribose import binding protein RbsB OS=Escherichia coli (strain K12) OX=83333 GN=rbsB PE=1 SV=1

Query	Observed	Mr(expt)	Mr(calc)	ppm	Miss	Score	Expect	Rank	Unique	Peptide
1196	481.2367	960.4588	960.4512	7.90	1	14	0.037	1	U	K.DGAQKEADK.L

69. 3::P0AEG4|DSBA_ECOLI Mass: 23204 Score: 14 Matches: 1(1) Sequences: 1(1) emPAI: 0.20
Thiol:disulfide interchange protein DsbA OS=Escherichia coli (strain K12) OX=83333 GN=dsbA PE=1 SV=1

Query	Observed	Mr(expt)	Mr(calc)	ppm	Miss	Score	Expect	Rank	Unique	Peptide
3068	605.8174	1209.6203	1209.6192	0.92	2	14	0.05	1	U	K.KLPEGVKMIK.Y

70. 3::P77766|RNAAM_ECOLI Score: 14 Matches: 1(1) Sequences: 1(1)
5'-3' exoribonuclease OS=Escherichia coli (strain K12) OX=83333 GN=yciV PE=1 SV=1

Query	Observed	Mr(expt)	Mr(calc)	ppm	Miss	Score	Expect	Rank	Unique	Peptide
369	400.7352	799.4558	799.4552	0.77	0	14	0.042	2	U	R.AQLIAER.L

71. 3::P39347|INTB_ECOLI Mass: 45725 Score: 14 Matches: 1(1) Sequences: 1(1) emPAI: 0.10
Putative protein IntB OS=Escherichia coli (strain K12) OX=83333 GN=intB PE=5 SV=1

Query	Observed	Mr(expt)	Mr(calc)	ppm	Miss	Score	Expect	Rank	Unique	Peptide
2577	381.8837	1142.6292	1142.6196	8.33	0	14	0.048	1	U	M_HLLVHPNGSK.Y

72. 3::P00957|SYA_ECOLI Mass: 96315 Score: 14 Matches: 1(1) Sequences: 1(1) emPAI: 0.05
Alanine--tRNA ligase OS=Escherichia coli (strain K12) OX=83333 GN=alaS PE=1 SV=2

Query	Observed	Mr(expt)	Mr(calc)	ppm	Miss	Score	Expect	Rank	Unique	Peptide
455	412.2393	822.4640	822.4712	-8.74	0	14	0.04	1	U	R.VIADHIR.S

73. 3::P16869|FHUE_ECOLI Mass: 81239 Score: 14 Matches: 1(1) Sequences: 1(1) emPAI: 0.05
FhuE receptor OS=Escherichia coli (strain K12) OX=83333 GN=fhuE PE=1 SV=2

Query	Observed	Mr(expt)	Mr(calc)	ppm	Miss	Score	Expect	Rank	Unique	Peptide
3632	638.2583	1274.5021	1274.5114	-7.30	0	14	0.043	1	U	R.LTTTLAIFR.I

74. 3::P77658|YNAA_ECOLI Mass: 37094 Score: 14 Matches: 1(1) Sequences: 1(1) emPAI: 0.12
Protein YnaA OS=Escherichia coli (strain K12) OX=83333 GN=ynaA PE=4 SV=1

Query	Observed	Mr(expt)	Mr(calc)	ppm	Miss	Score	Expect	Rank	Unique	Peptide
1009	465.7793	929.5440	929.5406	3.59	2	14	0.042	1	U	R.KIQETRR.E

75. 3::P50456|MLC_ECOLI Mass: 44516 Score: 14 Matches: 1(1) Sequences: 1(1) emPAI: 0.10
Protein mlc OS=Escherichia coli (strain K12) OX=83333 GN=mlc PE=1 SV=2

Query	Observed	Mr(expt)	Mr(calc)	ppm	Miss	Score	Expect	Rank	Unique	Peptide
1724	522.7363	1043.4580	1043.4634	-5.19	0	14	0.045	1	U	R.MPFYEDVK.E

76. 3::P02943|LAMB_ECOLI Mass: 49995 Score: 14 Matches: 1(1) Sequences: 1(1) emPAI: 0.09
Maltoporin OS=Escherichia coli (strain K12) OX=83333 GN=lamB PE=1 SV=1

Query	Observed	Mr(expt)	Mr(calc)	ppm	Miss	Score	Expect	Rank	Unique	Peptide
113	366.2239	730.4332	730.4337	-0.75	0	14	0.043	1	U	K.LSLAATR.S

77. 3::P0ADF6|EDD_ECOLI Mass: 65054 Score: 14 Matches: 1(1) Sequences: 1(1) emPAI: 0.07
Phosphogluconate dehydratase OS=Escherichia coli (strain K12) OX=83333 GN=edd PE=1 SV=1

Query	Observed	Mr(expt)	Mr(calc)	ppm	Miss	Score	Expect	Rank	Unique	Peptide
2	350.1556	698.2967	698.3000	-4.78	0	14	0.043	1	U	R.LSGASGK.V

78. 3::P37902|GLTI_ECOLI Score: 14 Matches: 1(1) Sequences: 1(1)
Glutamate/aspartate import solute-binding protein OS=Escherichia coli (strain K12) OX=83333 GN=gltI PE=1 SV=2

Query	Observed	Mr(expt)	Mr(calc)	ppm	Miss	Score	Expect	Rank	Unique	Peptide
723	439.2244	876.4342	876.4341	0.11	1	14	0.045	2	U	R.KDDPQFK.K

79. 3::P28635|METQ_ECOLI Mass: 29471 Score: 13 Matches: 1(1) Sequences: 1(1) emPAI: 0.15
D-methionine-binding lipoprotein MetQ OS=Escherichia coli (strain K12) OX=83333 GN=metQ PE=1 SV=2

Query	Observed	Mr(expt)	Mr(calc)	ppm	Miss	Score	Expect	Rank	Unique	Peptide
350	396.2241	790.4336	790.4337	-0.17	0	13	0.046	1	U	K.VFNGGAVK.G

80. 3::P23842|PDEA_ECOLI Mass: 84325 Score: 13 Matches: 1(1) Sequences: 1(1) emPAI: 0.05
Probable cyclic di-GMP phosphodiesterase PdeA OS=Escherichia coli (strain K12) OX=83333 GN=pdeA PE=2 SV=2

Query	Observed	Mr(expt)	Mr(calc)	ppm	Miss	Score	Expect	Rank	Unique	Peptide
11012	577.1268	2880.5974	2880.6176	-7.03	3	13	0.047	1	U	R.FSVNLMPLILLQKNIAGRIIRLFK.R

81. 3::P56258|WECF_ECOLI Mass: 40899 Score: 13 Matches: 1(1) Sequences: 1(1) emPAI: 0.11

TDP-N-acetylfucosamine:lipid II N-acetylfucosaminyltransferase OS=Escherichia coli (strain K12) OX=83333 GN=wecF PE=1 SV=2

Query	Observed	Mr(expt)	Mr(calc)	ppm	Miss	Score	Expect	Rank	Unique	Peptide
529	420.6980	839.3815	839.3790	2.98	0	13	0.047	1	U	R.QLASVDK.N

82. 3::P31224|ACRB_ECOLI Mass: 113615 Score: 13 Matches: 1(1) Sequences: 1(1) emPAI: 0.04

Multidrug efflux pump subunit AcrB OS=Escherichia coli (strain K12) OX=83333 GN=acrB PE=1 SV=1

Query	Observed	Mr(expt)	Mr(calc)	ppm	Miss	Score	Expect	Rank	Unique	Peptide
632	430.7217	859.4288	859.4287	0.11	0	13	0.05	1	U	K.IDIDQEK.A
