# Supplementary material for: ERK2-topoisomerase II regulatory axis is important for gene activation in immediate early genes
Source: Nat Commun. 2023 Dec 14;14:8341. doi: 10.1038/s41467-023-44089-y (PMC10721843; doi:10.1038/s41467-023-44089-y)

Figure 1e : Raw data.

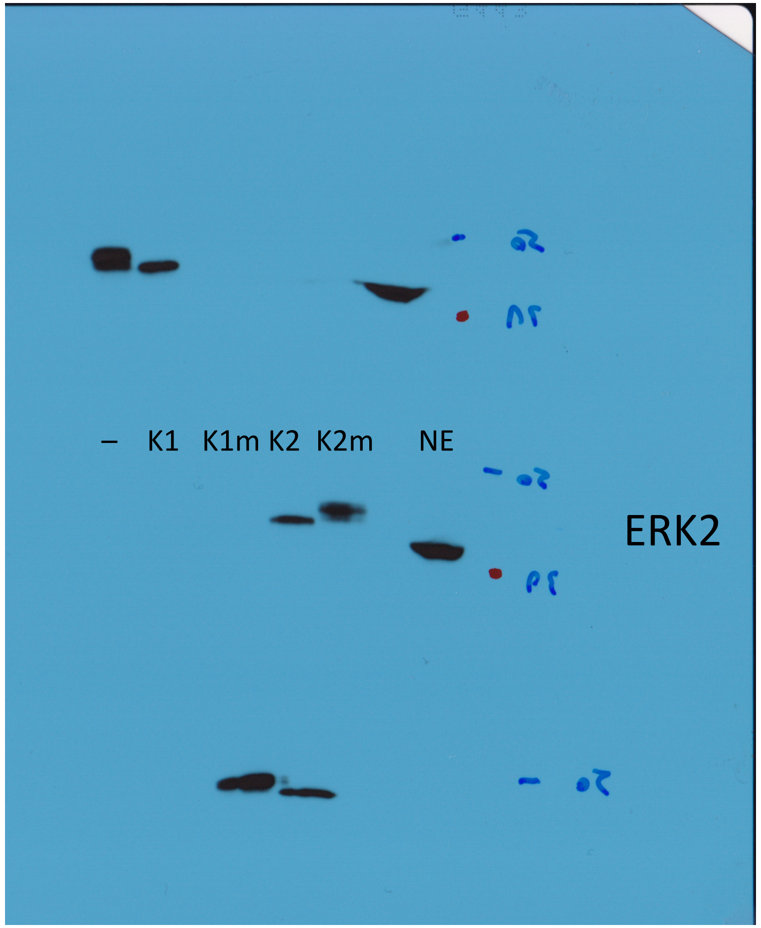

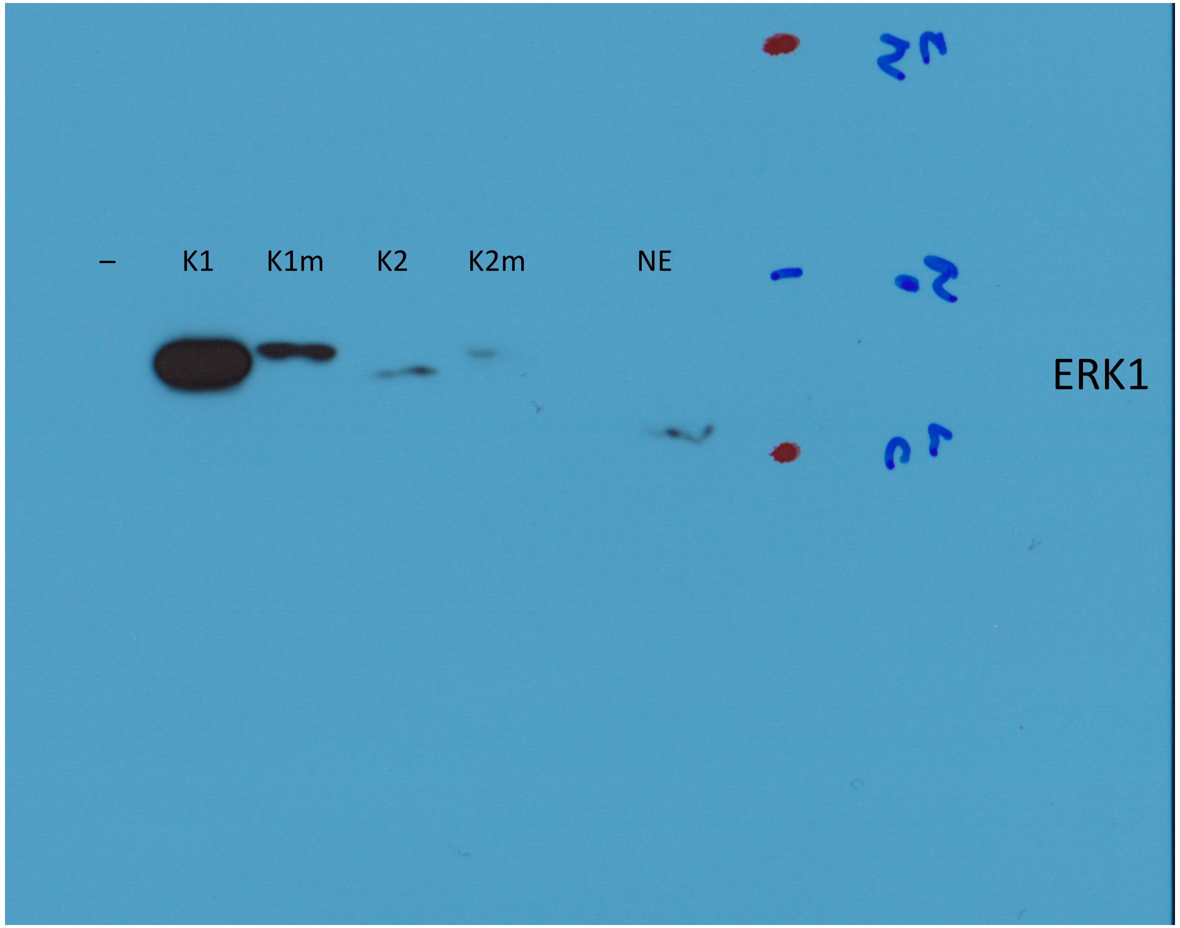

Figure 1f: Raw data.

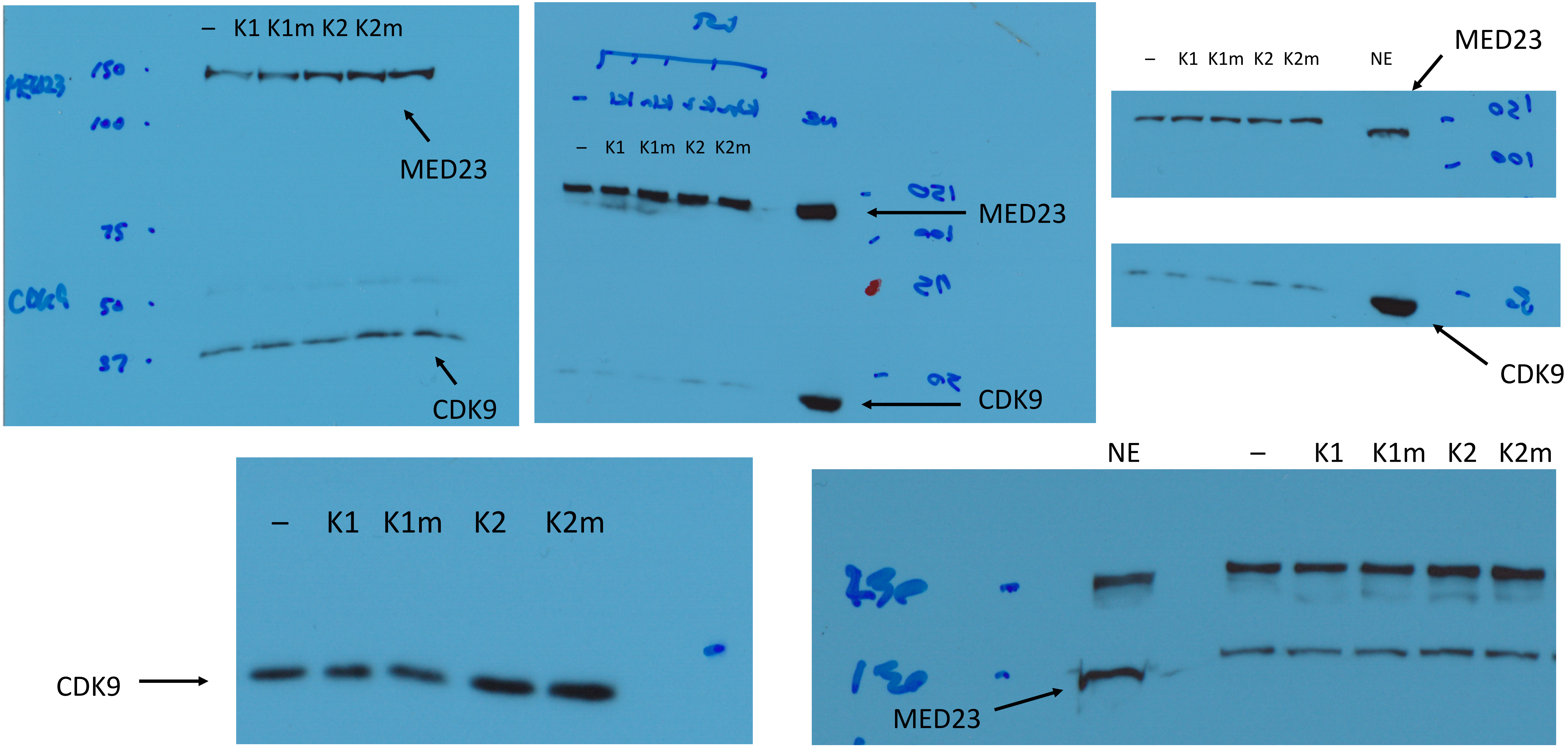

Figure 2b : Raw data.

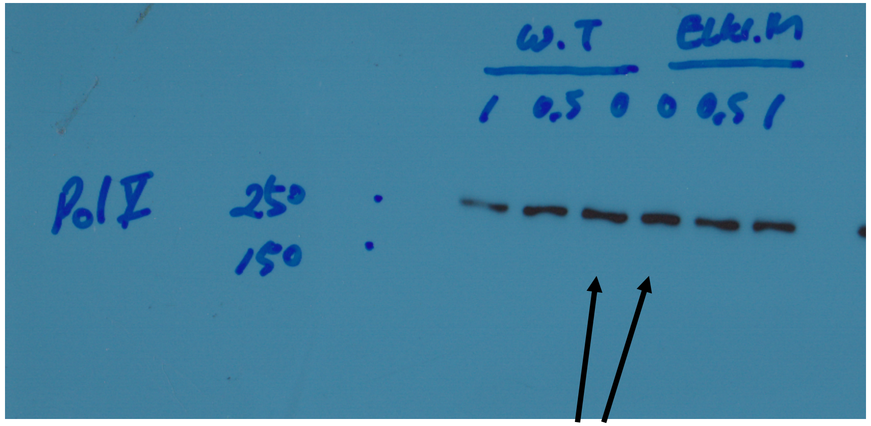

Pol II

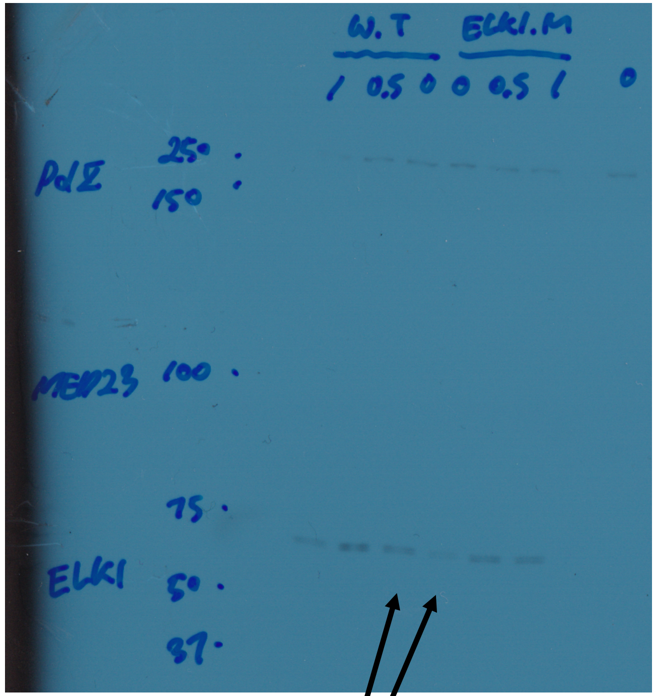

ELK1

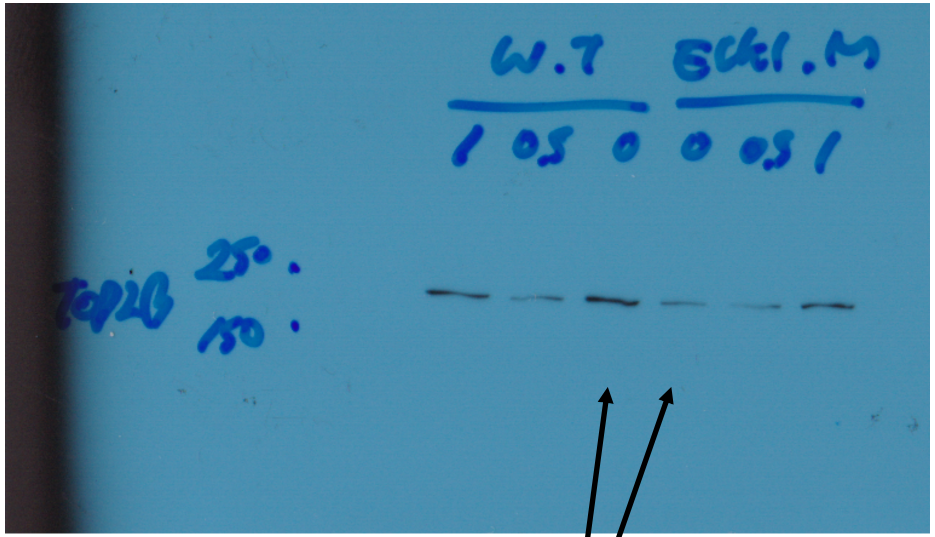

TOP2B

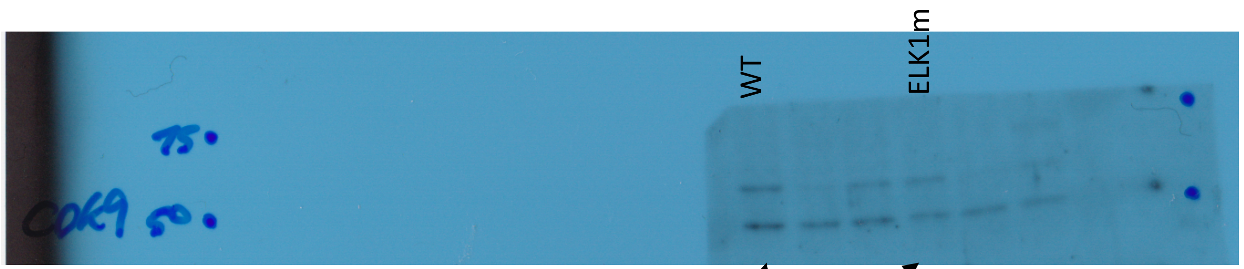

CDK9

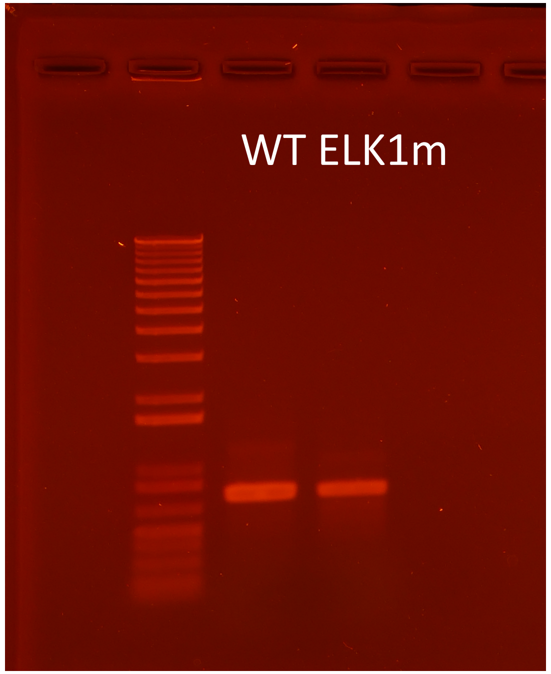

WT and ELK1m EGR1 template: 50 ng/uL per each

WT: WT EGR1

M or ELK1m: ELK1mut template

Figure 2d : Raw data.

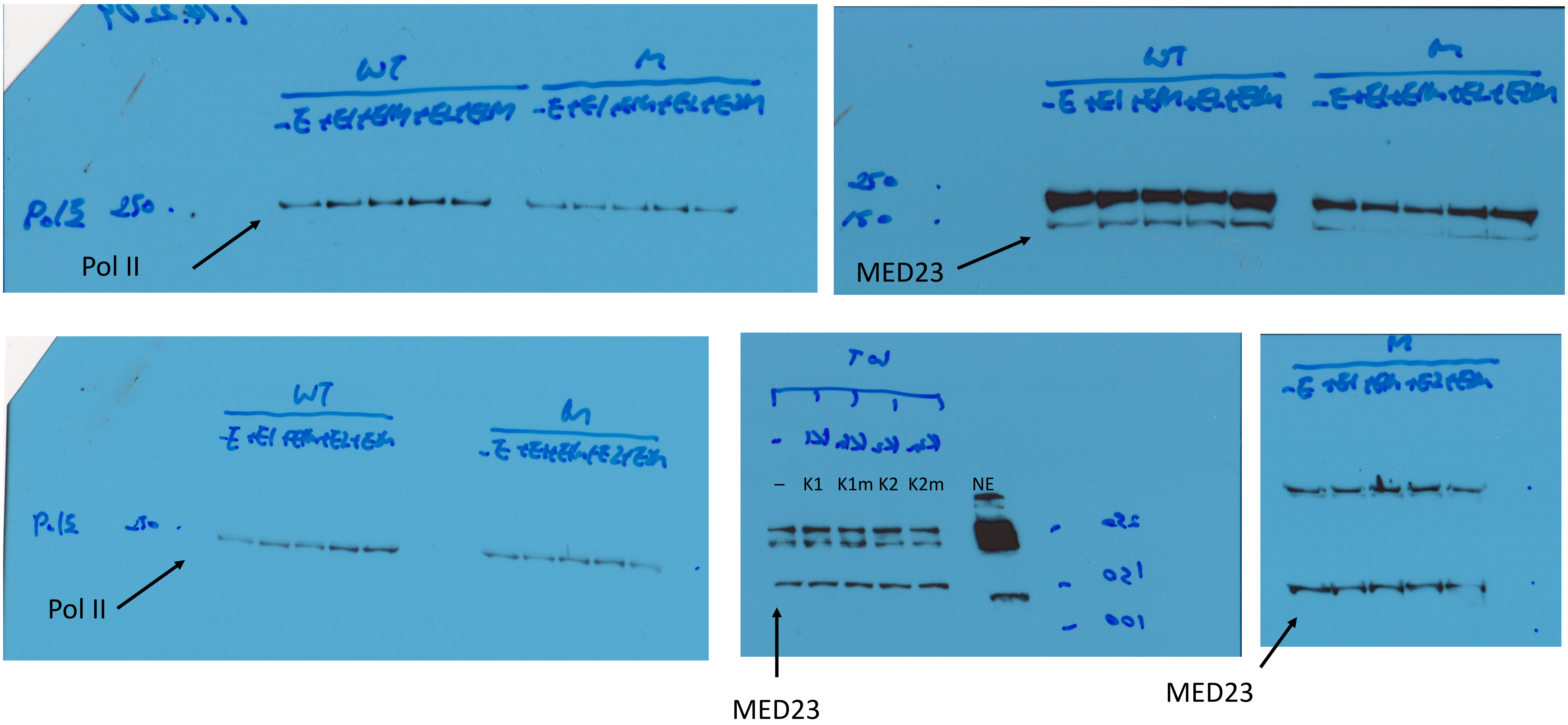

WT: WT EGR1; M or ELK1m: ELK1mut template

Figure 3a: Raw data.

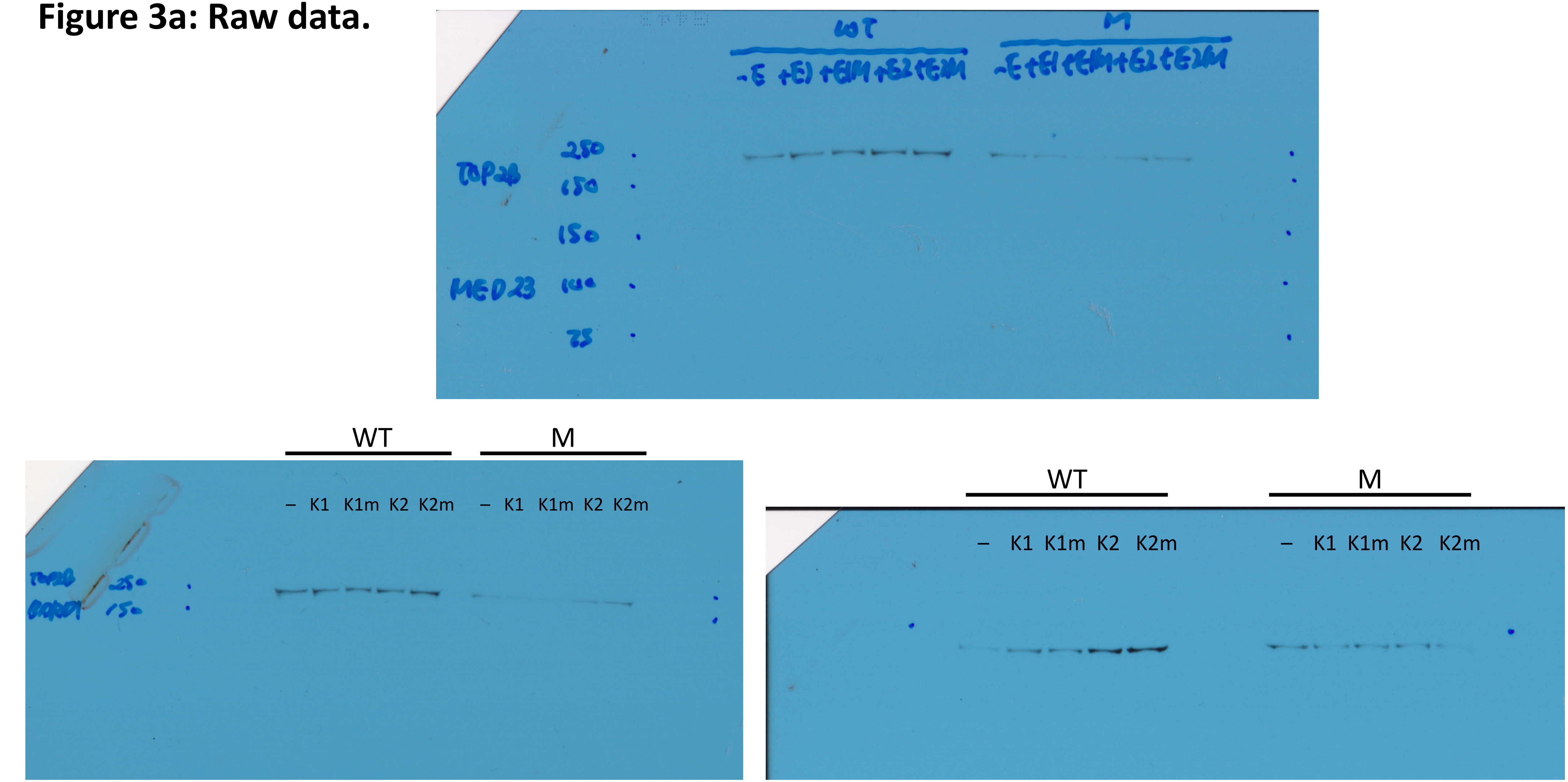

WT: WT EGR1

M or ELK1m: ELK1mut template

Figure 5b: Raw data.

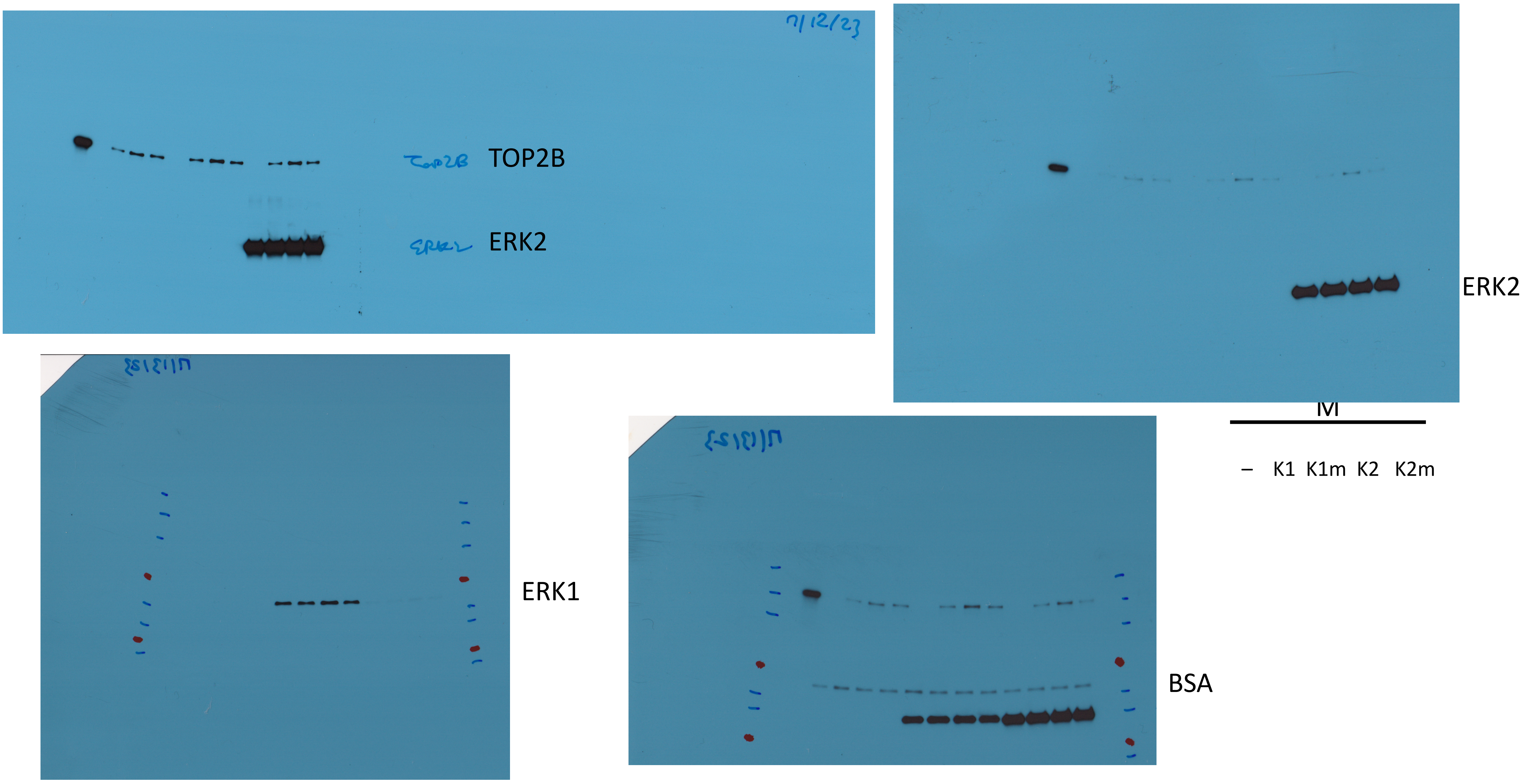

Figure 6e: Raw data.

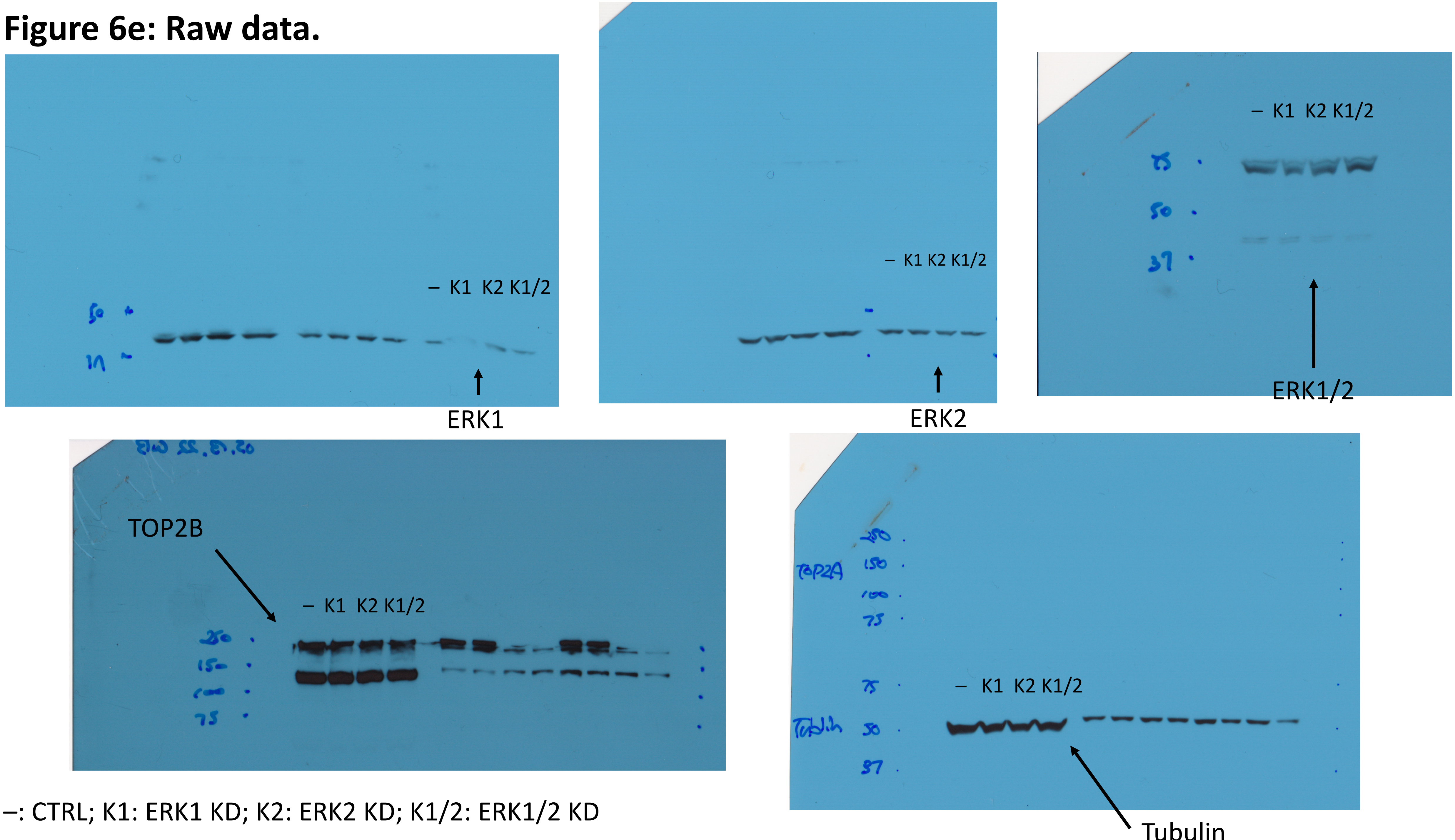

–: CTRL; K1: ERK1 KD; K2: ERK2 KD; K1/2: ERK1/2 KD

Supplementary Figure 1A: Raw data.

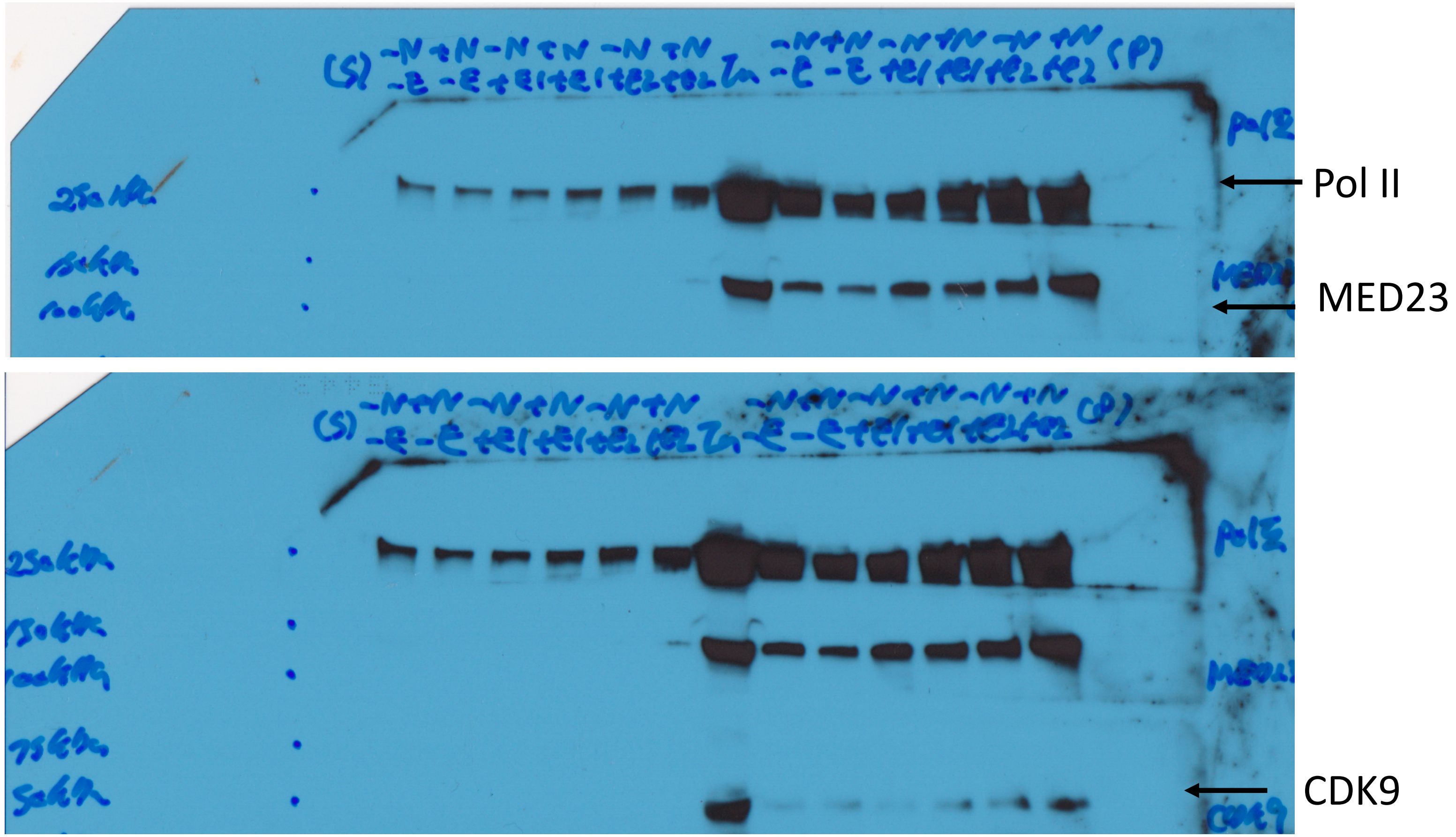

Supplementary Figure 1B: Raw data.

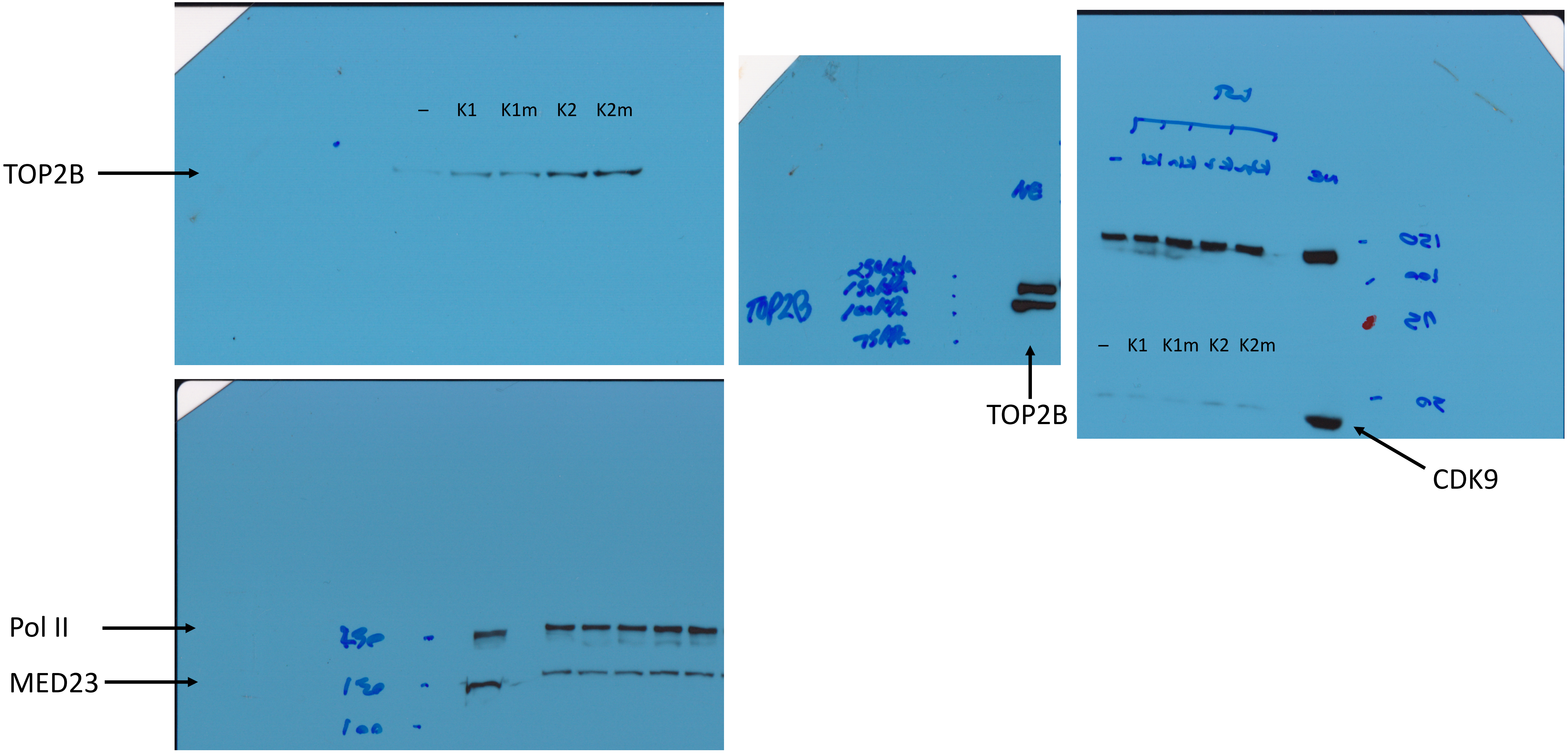

Supplementary Figure 1C: Raw data.

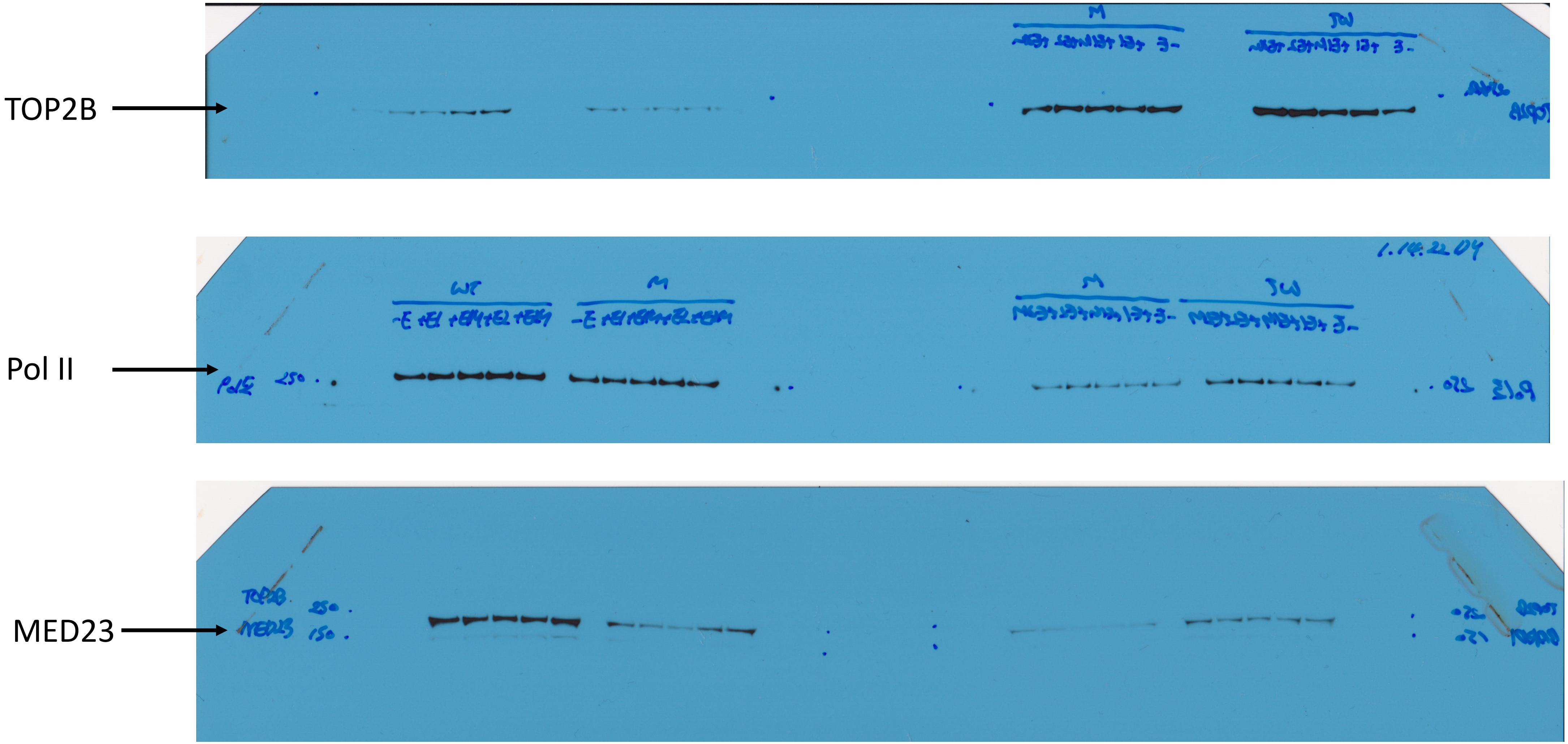

Supplementary Figure 2A: Raw data.

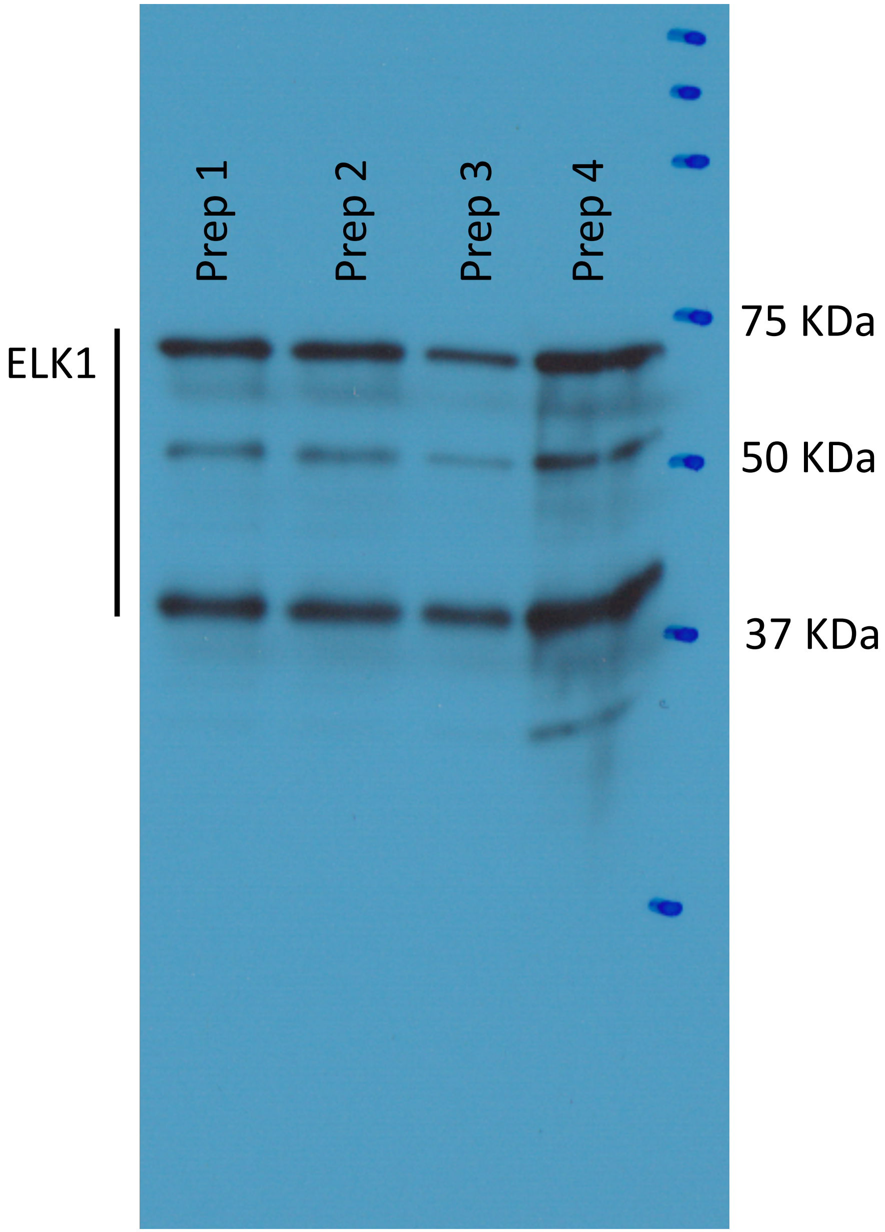

Prep 3 was shown in Fig. S2A and used in this study

Supplementary Figure 2B: Raw data.

TOP2B phosphorylated by ERK1 and ERK2

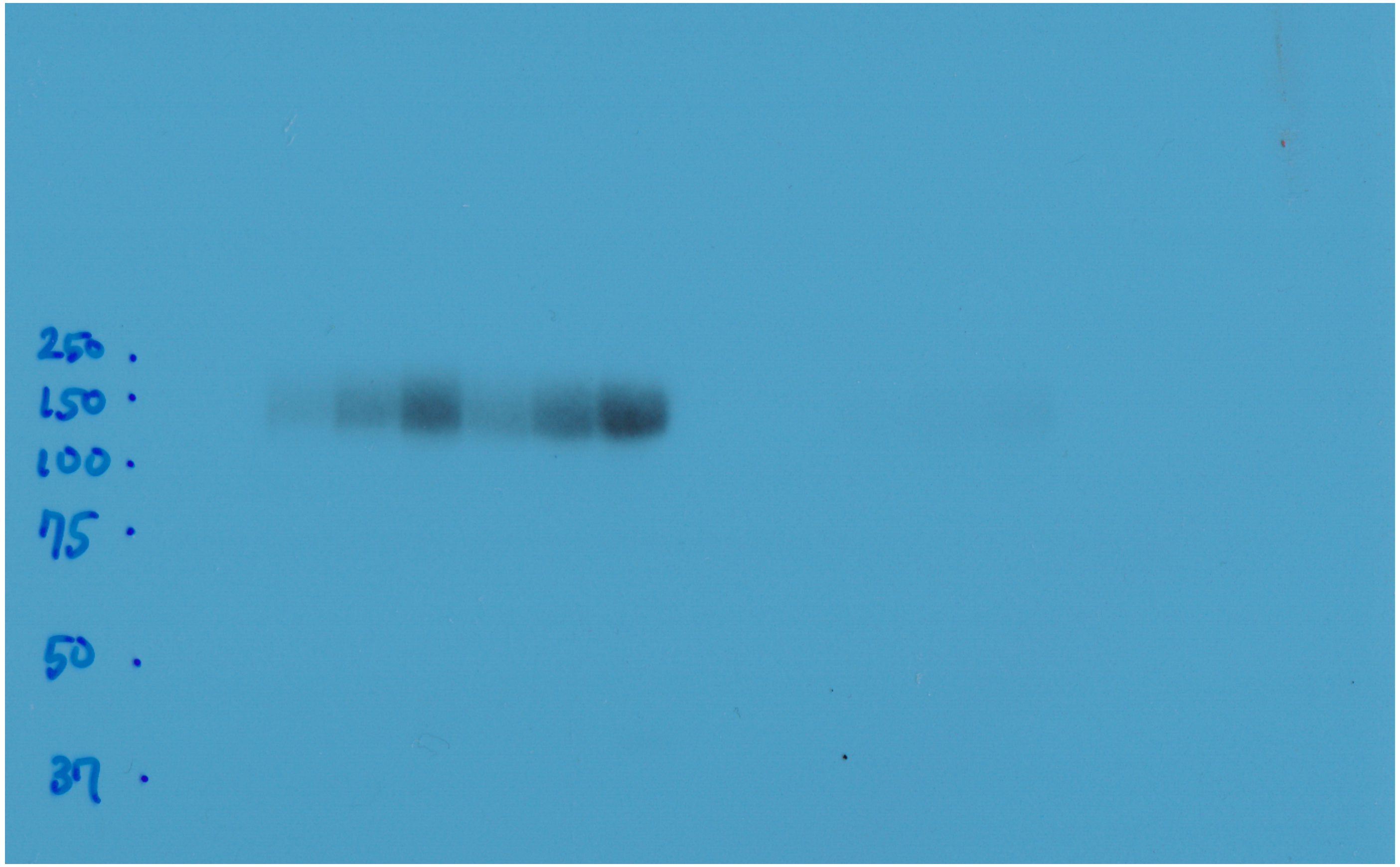

Supplementary Figure 7A: Raw data.

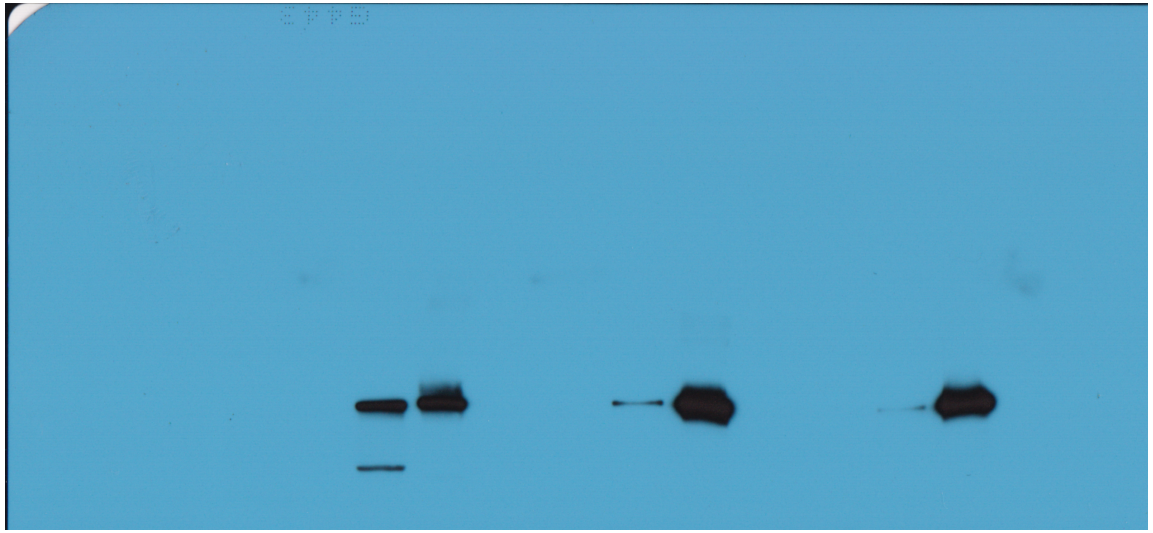

TOP2B

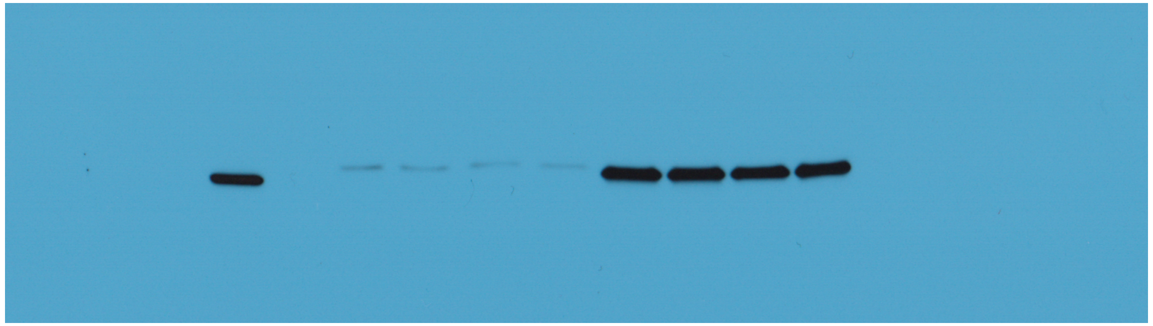

ERK2

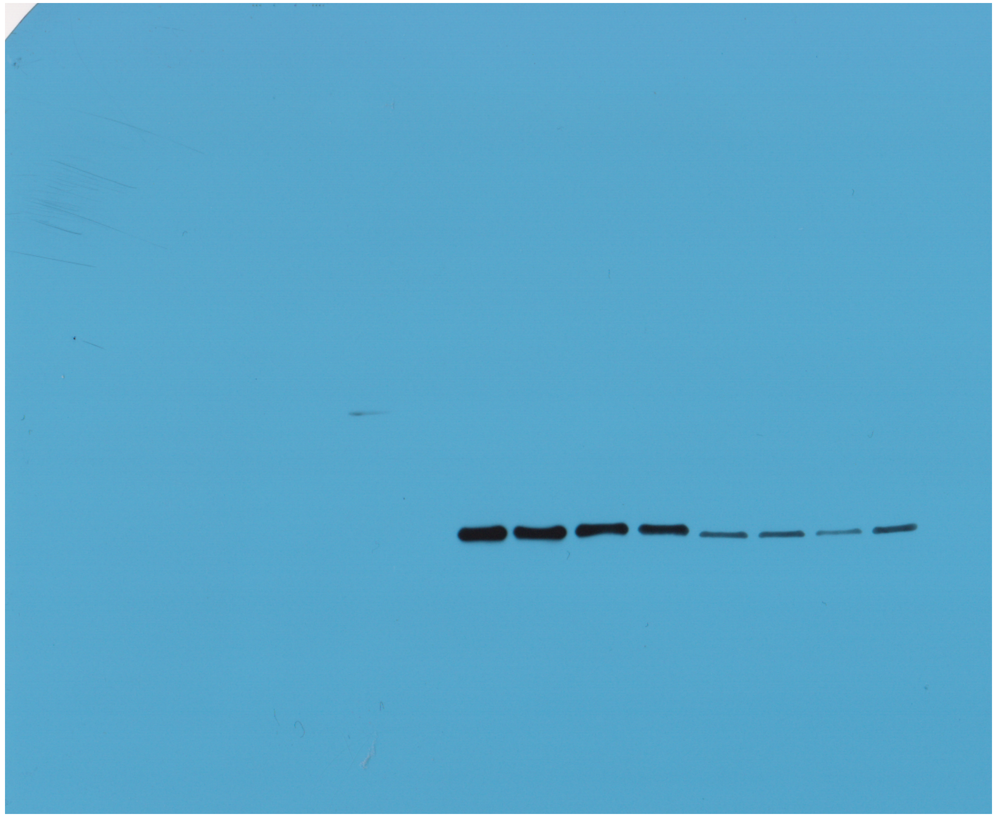

ERK1

Supplementary Figure 7B: Raw data.

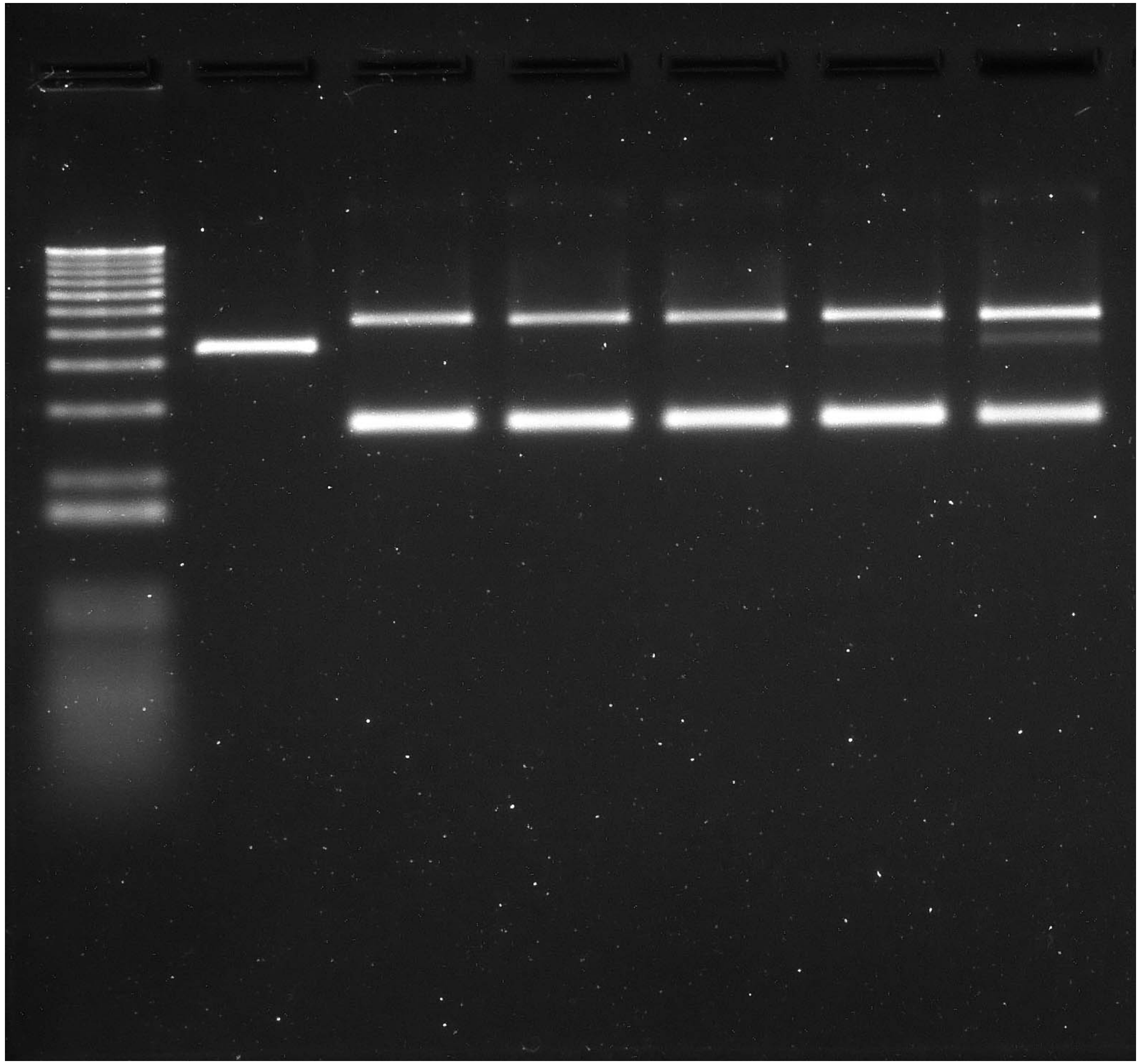

Supplement: Supplementary file 14 — Source Data [file 41467_2023_44089_MOESM14_ESM.zip › Source Data File-Bunch.pdf]
